# Comparative Study of Single-stranded Oligonucleotides Secondary Structure Prediction Tools

**DOI:** 10.1186/s12859-023-05532-5

**Published:** 2023-11-08

**Authors:** Thomas Binet, Séverine Padiolleau-Lefèvre, Stéphane Octave, Bérangère Avalle, Irene Maffucci

**Affiliations:** Université de technologie de Compiègne, UPJV, CNRS, Enzyme and Cell Engineering, Centre de recherche Royallieu - CS 60 319, 60203 Compiègne Cedex, France

**Keywords:** Single-stranded oligonucleotides, Secondary structure, Prediction, Benchmark

## Abstract

**Background:**

Single-stranded nucleic acids (ssNAs) have important biological roles and a high biotechnological potential linked to their ability to bind to numerous molecular targets. This depends on the different spatial conformations they can assume. The first level of ssNAs spatial organisation corresponds to their base pairs pattern, i.e. their secondary structure. Many computational tools have been developed to predict the ssNAs secondary structures, making the choice of the appropriate tool difficult, and an up-to-date guide on the limits and applicability of current secondary structure prediction tools is missing. Therefore, we performed a comparative study of the performances of 9 freely available tools (mfold, RNAfold, CentroidFold, CONTRAfold, MC-Fold, LinearFold, UFold, SPOT-RNA, and MXfold2) on a dataset of 538 ssNAs with known experimental secondary structure.

**Results:**

The minimum free energy-based tools, namely mfold and RNAfold, and some tools based on artificial intelligence, namely CONTRAfold and MXfold2, provided the best results, with $\sim 50\%$ of exact predictions, whilst MC-fold seemed to be the worst performing tool, with only $\sim 11\%$ of exact predictions. In addition, UFold and SPOT-RNA are the only options for pseudoknots prediction. Including in the analysis of mfold and RNAfold results 5–10 suboptimal solutions further improved the performances of these tools. Nevertheless, we could observe issues in predicting particular motifs, such as multiple-ways junctions and mini-dumbbells, or the ssNAs whose structure has been determined in complex with a protein. In addition, our benchmark shows that some effort has to be paid for ssDNA secondary structure predictions.

**Conclusions:**

In general, Mfold, RNAfold, and MXfold2 seem to currently be the best choice for the ssNAs secondary structure prediction, although they still show some limits linked to specific structural motifs. Nevertheless, actual trends suggest that artificial intelligence has a high potential to overcome these remaining issues, for example the recently developed UFold and SPOT-RNA have a high success rate in predicting pseudoknots.

**Supplementary Information:**

The online version contains supplementary material available at 10.1186/s12859-023-05532-5.

## Introduction

Single-stranded nucleic acids (ssNAs) play pivotal roles in living organisms and, as a consequence, present a high biotechnological potential. Indeed, both RNA and ssDNA have a natural ability to bind a wide range of targets with high specificity and dissociation constants in the nano- to picomolar range, which makes them interesting for therapeutic or diagnostic applications [1–3].

**Fig. 1:**
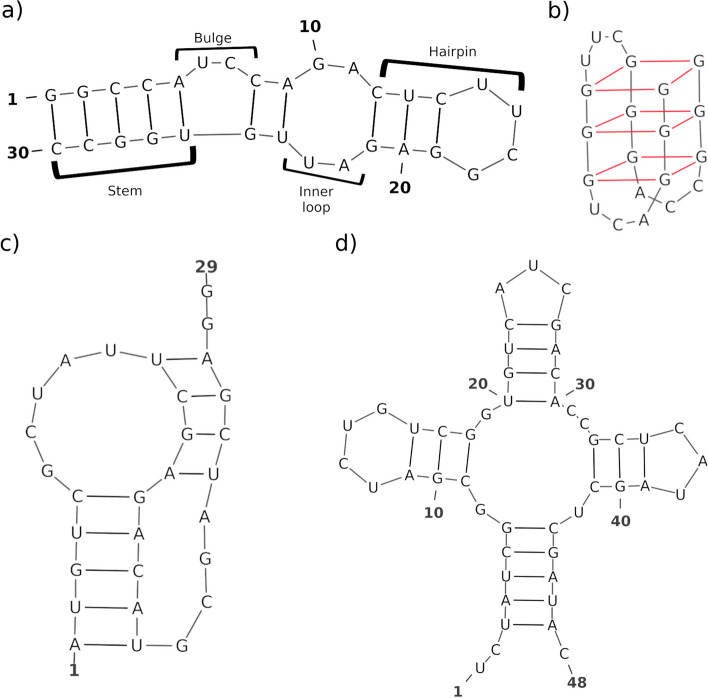
Main ssNA structural motifs. Stem, bulge, inner loop, and hairpin (**a**); G-quadruplex (**b**); pseudoknot (**c**) and multiple ways junction (**d**)

The ssNAs binding properties, and therefore their function and biological impact, mostly depend on their spatial conformation, which can be essentially described by the base pairs pattern formed within a nucleic acid sequence, namely its secondary structure. The simplest ssNA secondary structure elements are stems, inner loops, bulges, and hairpins (Fig. 1a). In addition, more complex motifs, such as G-quadruplexes (Fig. 1b), pseudoknots (Fig. 1c), and multiple-ways junctions (Fig. 1d), have been characterized.

The knowledge of ssNA structures and functions benefits from the increase of the experimental data available in public databases, such as the Protein Data Bank and the Nucleic Acid Database. However, all the diversity of ssNAs structures has not been yet experimentally described, because the interest in this kind of molecules has arisen quite recently, and their structural characterization is hampered by their intrinsic high flexibility. SsNA structure prediction methods can be an interesting and powerful tool to help in the structural investigation of ssNAs in order to better understand the ssNAs functioning and to rationally design new ssNAs for therapeutic or diagnostic applications.

So far, many algorithms have been developed for the prediction of the ssNAs secondary structures. Two of the most commonly used tools at this scope are mfold [4] and RNAfold [5], which are based on the computation of the folding(s) with the minimum free energy (MFE) by relying on sets of thermodynamics parameters and a dynamic programming approach. Alternatively to MFE-based methods, other algorithms and tools have been developed, which, for example, implement different probabilistic models, such as CentroidFold [6], or make use of computational techniques as an alternative to the dynamic programming (Linearfold [7]). Additionally, newly available tools, such as MXfold2 [8], UFold [9], and SPOT-RNA [10], exploit modern computational techniques such as Machine Learning and Deep Learning.

Because of the large number of available ssNAs secondary structure prediction tools, the choice of the one to use might be non-trivial and might depend on many factors, such as the ssNA type (RNA or DNA), length and features. In addition, most of the available tools have been developed and tested for the prediction of RNA secondary structures, though much interest is rising toward ssDNA [11]. Indeed, as compared to RNA, ssDNA is more stable, due to the removal of the $2^\prime$-hydroxyl group present in RNA and replacement with a $2^\prime$-proton, making it highly interesting for biotechnological applications [12]. Besides the type of ssNA, the possibility of handling complex structures or of taking into account the ssNA experimental environment can be critical for the choice of the prediction algorithm. Therefore, it is fundamental to fully understand the applicability and the limits of the available tools, in order to determine the progress that need to be done in the field. In light of this, in the present study, we extensively compared the ability of several prediction tools, namely mfold, RNAfold, CentroidFold, CONTRAfold, MC-Fold, LinearFold, UFold, SPOT-RNA, and MXfold2, in correctly predicting the ssNAs secondary structures (Table 1). To do so, we retrieved 538 unique ssDNA and RNA sequences with known 3D structures, both free and in complex with proteins from public databases (namely PDB and NDB) [13, 14], and we extracted their secondary structure using x3dna [15]. To assess the performance of the selected prediction tools, we compared the predicted and the experimental ssNAs secondary structures using AptaMat as a metric. AptaMat is a secondary structure comparison metric we recently developed [16] able to provide a sensitive estimation of the impact of base pair variations between two structures. We showed that MFE-based approaches, such as RNAfold or mfold, still represent a good choice to predict ssNAs secondary structures. In addition, the option they offer to consider suboptimal predictions brings a substantial improvement in the prediction quality. Nevertheless, artificial intelligence might be extremely helpful for predicting ssNAs secondary structures, if coupled with thermodynamics models, as shown by the good performances of MXfold2.

**Table 1:** SsNA secondary structure prediction tools included in the present study

Method	Year	Prediction approach	ssDNA parameter	Pseudoknots	Speed
Mfold	2003	MFE-based	Yes	No	$O(n^3)$
RNAfold	2008	MFE-based	Yes	No	$O(n^3)$
CentroidFold	2009	MFE-based or knowledge-based with $\gamma$-centroid estimator	No	No	$O(n^3)$
LinearFold	2019	MFE-based or knowledge-based with runtime linearization and heuristic beam search	No	No	*O*(*n*)
CONTRAfold	2006	Machine learning	No	No	$O(n^3)$
MC-fold	2008	Machine learning	No	Yes	$O(n^{{}^{15}/{}_{2}})$
MXfold2	2021	Deep learning & thermodynamic	No	No	0.31 s (GPU)^a^
UFold	2022	Deep learning	No	Yes	0.16 s (GPU)^a^
SPOT-RNA	2019	Deep learning	No	Yes	77.80 s (GPU)^a^

^a^Average run-time measured for sequences of 1000–1500 nucleotides

## Materials and methods

### ssNAs dataset

For the benchmark of the selected ssNAs secondary structure prediction tools, we retrieved the sequences of ssNAs with available 3D structures from the Protein Data Bank [14] and the Nucleic Acid Database [13]. We excluded the sequences containing non-natural nucleotides and/or G-quadruplexes and those in complex with a small molecule. The resulting dataset is made of 538 ssNAs, including 67 ssDNAs and 471 RNAs (Additional file 2). For each ssNA, we recovered the experimental secondary structure in the dot-bracket notation by using x3DNA-dssr [15] from its 3D structure.

### Secondary structure prediction tools

We made the choice of including in this benchmark only ssNAs secondary structure prediction tools freely available as standalone programs for Unix/Linux distribution. The selected tools are summarized in Table 1.

It has to be mentioned that, although all the selected tools can take DNA sequences as input, most of them have been developed to predict RNA secondary structures and, therefore, they preliminary convert DNA to RNA sequences. Only mfold and RNAfold can use distinct prediction parameters for DNA and RNA sequences.

#### MFE-based prediction tools

The most commonly used MFE-based prediction tool is mfold, which was the first one to include dynamic programming to predict the minimum free energy (MFE) structures [4, 17]. When submitting a ssNA sequence to mfold, the user can additionally set multiple parameters, among which the most interesting are: (1) the type of ssNA (RNA by default); (2) its shape, either linear or circular (linear by default); (3) the simulation temperature (between 0 and 100 $^{\circ }$C, 37 $^{\circ }$C by default); (4) the Na$^+$ (1 M by default) or Mg$^{2+}$ (0 M by default) ions concentration, (5) the potential constraints to force or forbid the formation of specific base pairs; (6) the maximum number of foldings (100 by default); and (7) the threshold to compute suboptimal foldings (5 % by default). This is defined as a percent suboptimality, *p*, which corresponds to the percentage from the MFE that will be considered when computing the suboptimal folding.

The second MFE-based prediction tool included in the benchmark is RNAfold, which is part of the ViennaRNA tool suite [5]. The main difference between RNAfold and mfold is the thermodynamics parameters applied to calculate MFE. In RNAfold, four energy models are included: (1) the Turner model of 1999 [18] which works with a nearest-neighbour energy estimation at 37 $^{\circ }$C; (2) the Turner model of 2004 [19], which considers enthalpy changes to predict secondary structures at arbitrary temperatures; (3) the optimized Turner model by Andronescu et al. 2007 [20], which has been trained on an experimental dataset including structural and thermodynamics data; (4) the Mathews model (2004) [19], the only one developed explicitly for DNA sequences. In addition, RNAfold allows setting the same parameters as mfold, except for the choice of an energy threshold to output suboptimal foldings, which, in the ViennaRNA suite is handled by RNAsubopt.

For both mfold and RNAfold, default parameters were kept, with the thermodynamics models being the Turner (2004) and Mathews (1999) models for RNAfold and mfold, respectively. Subsequently, we investigated whether the prediction of the ssDNA secondary structures improved when using the thermodynamics models developed for DNA sequences (the SantaLucia (1998) and Mathews (2004) models for mfold and RNAfold, respectively), as compared to the default models. Finally, we extended our analysis by considering suboptimal foldings.

#### Machine learning or deep learning prediction tools

Recently, thanks to the increased availability of experimental ssNAs structures, methods based on machine learning (ML) or deep learning have been developed to tackle the secondary structure prediction problem. In this context, the knowledge-based approach implemented within CONTRAfold exploits conditional log-linear models (CLLMs) as a stochastic context-free grammar approach (SCFG) coupled with a simplification of the traditional energy-based scoring scheme [21]. CONTRAfold allows setting alternative model parameters for secondary structures prediction and the parameter $\gamma$ for the maximum expected accuracy (MEA) function involved in the ranking of the predicted secondary structures.

Like CONTRAfold, MC-Fold [22] implements a knowledge-based algorithm. Within MC-Fold, a pseudo-potential energy function is derived by using statistics from the PDB database on a library representing nucleotide relationships in structured RNAs, which includes all base pair types. Dynamic programming is then used to enumerate the obtained sub-optimal solutions. MC-Fold can handle H-type pseudoknots, by specifying the “pseudoknot” keyword when launching the simulation. We tested MC-fold with or without this option, in an attempt to better estimate the secondary structures of ssNAs with pseudoknots.

We included in our benchmark another very recent tool, MXfold2 [8], which estimates the most probable secondary structure by integrating ssNA folding scores learnt using a deep neural network with Turner’s nearest-neighbor free energy parameters [23]. In addition, we selected two deep learning-based tools which have the advantage of handling pseudoknots: SPOT-RNA [10] and UFold [9]. The former implements an algorithm making use of deep contextual learning for the secondary structure prediction, while the latter uses an image-like representation of RNA sequences which then are processed by full convolutional networks. Both tools have been developed and extensively tested only on RNA sequences.

#### Other prediction tools

Several software implemented modifications to either MFE-based or knowledge-based algorithms for the ssNA secondary structure prediction. For example, LinearFold implements an algorithm applicable to both MFE-based (LinearFold-V) and alternative approaches, such as CONTRAfold, (LinearFold-C) to linearize the runtime of the dynamic programming (1) by iterating over the sequence from $5^\prime$ to $3^\prime$ incrementally tagging each nucleotide using the dot-bracket notation, and (2) by using the heuristic beam search method to prune the search space. In this benchmark, both LinearFold-C (default) and LinearFold-V were tested.

CentroidFold [6] makes use of the $\gamma$-centroid estimator to find the most probable secondary structure as an alternative to the MFE or MEA approaches. It can be coupled to different probability distributions, including the CONTRAfold one or the Vienna RNAfold McCaskill [20] model. Sato et al. showed that CentroidFold provided the best results coupled with Vienna RNAfold McCaskill model, thus only this combination was included in the benchmark.

### Comparison metrics

To assess the accuracy of the selected tools, we compared the predicted secondary structure in the dot-bracket notation to the one retrieved from the experimental structure (i.e. the reference) for each sequence of the previously described dataset and each selected tool. For this purpose, we used AptaMat [16] as a metric, which we developed to specifically compare ssNAs secondary structures by representing them as matrices and making use of a metric built upon the Manhattan distance in the plane. The python code implementing AptaMat is publicly available at https://github.com/GEC-git/AptaMat.git. We showed that AptaMat is able to determine the difference between secondary structures with the highest possible sensitivity. Indeed, it showed to be able to distinguish and correctly rank highly similar secondary structures, where other commonly used metrics (i.e. F1 score, Matthews correlation coefficient (MCC), the Hamming distance, and the RNAdistance) failed as showed in the example reported in the Additional file 1, where AptaMat is the only one capable to highlight the differences between the proposed structures from the reference.

In addition, this metric can handle extended dot-bracket notations describing motifs such as pseudoknots. We defined an AptaMat distance ($Apta_D$) threshold of 1.5 to discriminate between close and far structures. This threshold was chosen by analyzing the results of the RNA families clustering study we recently performed [16]: indeed, we could observe a $Apta_D < 1.5$ within each RNA family, and, therefore, structures with an $Apta_D \le 1.5$ can be considered similar. Thus, structures showing an $Apta_D$ from the experimental structure of 0 are correctly predicted, structures showing an $Apta_D \le 1.5$ are considered close to the reference, and structures showing an $Apta_D > 1.5$ are considered incorrectly predicted.

Although the following discussion will be based on AptaMat, we also carried out the comparison of the efficiency of the different selected tools using the F1 score and the MCC as metrics, which are the most commonly used metrics for this type of study (Additional files 11 and 12). As indicated in the literature [24, 25], predictions with F1 score and MCC equal to 1 are exact and a threshold of 0.5 for both the F1 score and the MCC, has been fixed to discriminate predicted structures close to the reference from the far ones. The obtained results using these metrics are globally comparable to those obtained with AptaMat (Additional files 11 and 12).

## Results and discussion

The benchmark ssNAs dataset was built by retrieving all the ssNA sequences with available 3D structures from the Protein Data Bank [14] and the Nucleic Acid Database [13], excluding the sequences containing non-natural nucleotides and/or G-quadruplexes. Indeed, although recent works described promising methods to allow secondary structure prediction for nucleic acid analogues [26], the inclusion of non-natural nucleotides in our dataset may result in issues while predicting secondary structures as current software cannot directly handle unconventional nucleotides. G-quadruplexes were also discarded, since for these peculiar motifs recent studies have already shown the limits of the available prediction tools [27, 28]. In addition, we excluded from the dataset the ssNAs experimentally characterized in complex with small molecules, because the interaction with the molecular target usually drastically alters the ssNAs base pairing [29, 30]. However, we kept the ssNA sequences whose structure has been determined in complex with proteins: this might also affect, but only to a lower extent, the ssNA secondary structure [31]. This choice allowed us to investigate how the prediction tools deal with this situation. The resulting dataset is one of the largest datasets containing structures retrieved from the PDB and NDB databases, and it is made of 538 ssNAs, including 67 ssDNAs and 471 RNAs (Additional file 2), with a length ranging from 7 to 1509 nucleotides. As compared to other datasets used in this kind of studies [27, 32], the one we built allows us to fully challenge the selected prediction tools, by testing their efficiency on two types of ssNAs (ssDNA and RNA), on a wide ssNAs length and structure complexity range, and on ssNAs whose structure can be affected by the surrounding environment.

The following discussion is based on the use of the AptaMat distance from the reference as the only metric. However, we also performed the analysis with the F1 score and the MCC as metrics (Additional files 11 and 12). Independently from the metrics, we decided to set a threshold (see “Materials and methods” section for the choice of the threshold) to discriminate predictions close to and far from the experimental references, although all of them are continuous metrics. Indeed, the aim of this work is to verify the performances of some of the existing secondary structure prediction tools in reproducing experimental structure, and, ultimately, to provide to the potential user information about (1) the best suited tool as a function of the type of ssNAs, (2) the parameters to be included during the calculation (if available), and, most importantly, (3) the possibility of obtaining an incorrect prediction (i.e. far from the experimental) even when using the most appropriate tool. Therefore, the choice of a threshold allows us to provide easily interpretable results, together with taking into account small base pairs variations. Comparing the obtained results some differences can be observed in the percentage of good predictions ($Apta_D \le 1.5$, F1 score and MCC $\ge 0.5$) (Fig. 5, Additional files 11 and 12). In particular, the F1 score indicates a higher percentage of good predictions as compared to both AptaMat and the MCC, probably because it is less accurate than the MCC, which gives a high score only if the prediction obtained good results in all the confusion matrix categories [33]. However, the MCC sometimes fails in discriminating good from wrong predictions. The classification of some of the predictions for the ssNA with PDB code 6NOA (Additional file 13) offers an example of these issues. The experimental structure of this ssNA corresponds to a hairpin/stem loop interrupted by two bulges and two internal loops. CONTRAfold predicts a structure highly close to the experimental one, with one bulge becoming an internal loop because of the loss of a single base pair. This structural proximity to the reference is correctly captured by the three metrics. Ufold, on the contrary, provides a predicted structure farther from the experimental one as compared to the CONTRAfold one, since one bulge and two internal loops fusions into a wide internal loop. Although the evaluations provided by the three metrics are close to the chosen threshold, both AptaMat ($Apta_D = 1.52$) and the MCC (MCC = 0.47) capture this difference from the reference, while the F1 score associated to this structure is $\ge 0.5$ (0.59), wrongly indicating that this prediction is close to the reference. Finally, the structure predicted by SPOT-RNA is even more distant from the experimental one, since only one bulge is still present and 8 base pairs are lost. The distance of this prediction from the reference is correctly indicated by AptaMat ($Apta_D = 5.31$), while both the F1 score and the MCC (0.69 and 0.65, respectively) wrongly classify this prediction as close to the reference. Globally, the observed differences in the classification of good and wrong predictions can be ascribed to the fact that AptaMat has been specifically developed for the comparison of ssNA secondary structures, and it gives a high weight to the relative position of the base pairs, being, therefore, more sensitive for the classification of the predictions and more adapted for the scope of this particular study.

### Accuracy of MFE-based prediction tools

**Fig. 2:**
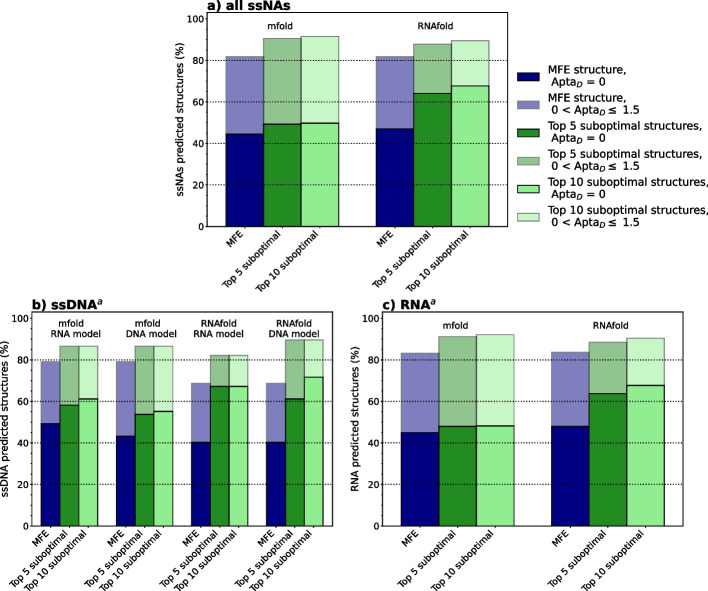
Percentages of ssNAs (**a**), ssDNA (**b**) and RNA (**c**) secondary structures predicted as identical (AptaD = 0, full-color bars) and close ($0 < Apta_D \le 1.5$, bars with transparency) to the experimental structure by mfold and RNAfold. The results obtained by considering only the MFE structure or either the top 5 or top 10 suboptimal solutions are included. For ssDNA sequences, the results obtained by using also the Santa-Lucia DNA model (mfold) and the Mathews 2004 DNA model (RNAfold) are included.$^a$ The reported percentages have been calculated on the ssDNA and RNA subsets

We initially tested the mfold and RNAfold performances under the default parameters, which implies using the Mathews 1999 model for mfold and Turner 2004 model for RNAfold, and outputting only the optimal solution. Overall, under these conditions, mfold and RNAfold provided comparable results, with $46\%$ and $47\%$ of exact predictions and $83\%$ and $82\%$ of good predictions (AptaMat distance from the experimental structure $\le 1.5$), respectively (Fig. 2a). We also analyzed the prediction tools performance as a function of the type of ssNA. We could only observe a minor difference in terms of quality of prediction between ssDNA and RNA sequences for mfold (Fig. 2). Indeed, this tool can correctly predict $49\%$ and $45\%$ of the ssDNA and RNA sequences, respectively. These percentages increase to $79\%$ and $83\%$, respectively, when we include the predictions with an $Apta_D \le 1.5$. Concerning RNAfold, we observed a slightly better accuracy for the prediction of RNA secondary structures against ssDNA structures. Indeed, it correctly predicts $48\%$ and $40\%$ of the RNA and ssDNA sequences, respectively. When predictions with an $Apta_D \le 1.5$ are included in the analysis, RNAfold showed a success percentage of $84\%$ and $69\%$ for RNA and ssDNA structures, respectively.

It is important to emphasize that both mfold and RNAfold algorithms impose 3 nucleotides as the minimum hairpin loop size, while, in the considered dataset, we observed the presence of loops composed of 2 nucleotides (PDB ID: 1EZN, 1SNJ, 2N8A, 4ER8, 4F41, 4F43, 1RNG, 2L6I, 2B6G, 2JYM, 2ES5, 2PJP, 2UWM, 1EKZ, 2MTJ, 2M3Q, 6U8D, 4ZT0, 5VW1, and 5XBL). As a consequence, these ssNAs cannot be exactly predicted by default. Moreover, both tools have some limits in correctly predicting base pairs involving the $5^\prime$ and $3^\prime$ ends. This issue might be considered negligible since the impact of terminal base pairs on global folding is limited. These two intrinsic limits of both mfold and RNAfold can explain $\sim 10\%$ of the predicted ssNAs secondary structures showing $0 < Apta_D \le 1.5$.

**Fig. 3:**
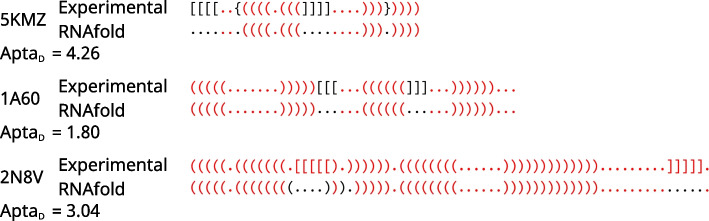
Examples of RNAfold predicted secondary structures aligned to the experimental ones. AptaMat distance value is high for the 3 examples as a result of missing pseudoknots prediction, despite a large pro-portion of well-predicted base pairs

In addition, mfold and RNAfold algorithms cannot predict pseudoknots: first, they do not make use of the extended dot-bracket notation, and, second, they do not contain the thermodynamics parameters for this kind of motif. This highly affects the estimation of the global accuracy of RNAfold and mfold since oligonucleotides with pseudoknots contribute to 14% of the dataset. Most of the related predicted structures showed an $Apta_D \le 1.5$, with less than 1% of ssNAs belonging to this group having $Apta_D > 1.5$. For this kind of structure, mfold and RNAfold usually indicate the nucleotides involved in the pseudoknot formation as unpaired, while they correctly detect the standard base pairs (Fig. 3). Unfortunately, since MFE-based algorithms are based on the local neighborhood to assign the corresponding energy, the possibility of implementing the pseudoknot prediction is limited by the necessity of significant computational time and resources, since pseudoknots require an extended overview of the whole secondary structure. In addition, specific thermodynamics parameters and weighting for pseudoknots are yet to be determined, although interesting approaches have been proposed [34].

Another potential bias in the MFE-based algorithms is represented by the prediction of multiple-ways junctions, present in $17\%$ of the ssNAs dataset. Indeed, mfold and RNAfold failed in correctly predicting the secondary structure of 43 and 46, respectively, out of 94 structures of the dataset with this structural organization. Most of these ssNAs structures have been experimentally resolved in complex with a protein or contain pseudoknots. Both situations might affect the secondary structure prediction. Indeed, the presence of pseudoknots might be responsible for the formation and stabilization of the multiple-ways junction, although we already pointed out the mfold and RNAfold failures in handling this kind of motif.

Furthermore, as previously mentioned, the presence of a binding partner might influence the ssNA folding, potentially stabilizing a conformation that is metastable for the free ssNA. For the same reason, in some cases (notably, PDB ID 2XXA, 1P5P, and 5TF6), both mfold and RNAfold predict the presence of multiple-ways junctions, while the experimental structure returns a unique hairpin/stem-loop as output. However, given the length of these 3 sequences (100 nucleotides on average) and the number of nucleotides involved in base pairs (36, 27 and 17 base pairs, respectively), the stability of the experimental structure in the free state is questionable.

#### Role of the thermodynamics model on ssDNA secondary structures prediction

As previously mentioned, both mfold and RNAfold offer the possibility of choosing the thermodynamic model depending on the oligonucleotide type, although the parameters originally determined for RNA are used by default. Therefore, we investigated whether the predictions for ssDNA secondary structures improved when using the adequate model. The results are summarized in Fig. 2b.

**Fig. 4:**
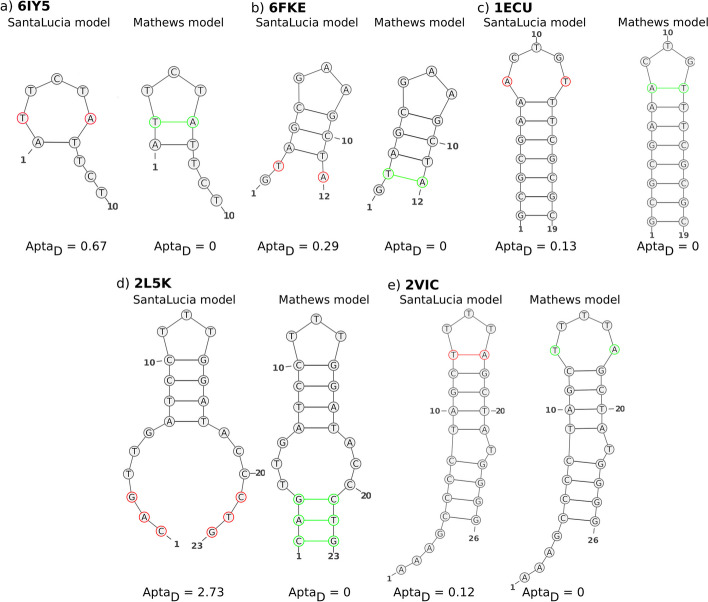
Mfold prediction of **a** 6IY5, **b** 6FKE, **c** 1ECU, **d** 2L5K, and **e** 2VIC secondary structures with SantaLucia DNA and Mathews RNA models. Nucleotides wrongly predicted as unpaired with the SantaLucia model are colored in red, while they are colored in green when the corresponding base pairs are correctly predicted with the Mathews model. AptaMat distances from the experimental structures are reported

Surprisingly, the AptaMat distances of the predicted from the experimental secondary structures of ssDNA sequences show a slightly lower prediction accuracy using the SantaLucia DNA model, implemented in mfold: $43\%$ of predictions are identical to experimental structures against $49\%$ obtained when using the RNA parameters. This might be partly due to the energetic penalties included in the SantaLucia DNA model—but not in the Mathews RNA model—for A-T pairing positioned at the extremity of a hairpin stem or for terminal mismatches [35]. The ssDNA structures with PDB ID 6IY5, 6FKE, 1ECU, 2VIC, and 2L5K are a striking example of this issue within the DNA model implemented in mfold (Fig. 4).

RNAfold, on the contrary, does not show any difference in the percentage of identical predictions as a function of the used model. In this case, the absence of improvement might be due to penalties for terminal mismatches, or to difficulties linked to predicting the secondary structures of short oligonucleotides ($\le 15$ nucleotides) using the Mathews DNA model.

If we consider all the ssDNA secondary structures with an $Apta_D \le 1.5$ as well-predicted, we could not observe any discrepancy between the DNA and RNA models, both giving $\sim 80\%$ and $70\%$ of good predictions with mfold and RNAfold, respectively (Fig. 2b). Indeed, except for structures such as 2L5K, where the global structure is significantly modified, the above-mentioned penalties affect the prediction of a limited number of base pairs, poorly affecting the final predicted structure as compared to experiments (Fig. 4).

#### Inclusion of suboptimal solutions

It is known that a single-stranded oligonucleotide sequence can adopt distinct but similar and equiprobable conformations [36, 37]. This is reflected by the alternative foldings found in some of the dataset structures obtained by NMR (PDB ID: 1M82, 1SCL, 1MFY, 1JO7, 5UZT, 2FEY, 2N6W), and it questions the default mfold and RNAfold prediction of one unique, namely the MFE, secondary structure. Therefore, since mfold and RNAfold can also generate suboptimal foldings, we extended the analysis of these two tools by allowing the generation of suboptimal structures, to verify if this might further improve the performances of the MFE-based algorithm in providing the expected secondary structure. As described in the “Materials and methods” section, we set the mfold and RNAfold parameters to retrieve all the possible suboptimal structures. Nevertheless, since the number of the obtained suboptimal solutions was system-dependent, we decided to focus on the top 5 and 10 suboptimal solutions (Additional files 4 and 5).

If we consider the whole dataset, the inclusion of suboptimal solutions in the analysis overall increased the percentage of identical or similar secondary structures compared to the reference. Nevertheless, we can observe that RNAfold predictions benefit at a higher extent from this as compared to the mfold. Indeed, using the default thermodynamics models, considering the top 5 or 10 suboptimal solutions provides an increase of $\sim 7\%$ for mfold, while RNAfold correct predictions increase by $\sim 20\%$, leading to an excellent percentage of correctly predicted structures of $\sim 65\%$ (Fig. 2a, Additional files 4 and 5). Nevertheless, this difference disappears if we include in the analysis the predicted structures with an $Apta_D \le 1.5$ from the experimental ones, with both tools reaching $\sim 90\%$ of success.

According to the ssNA type, the RNA sequences dataset benefits at a lower extent from the inclusion of suboptimal predictions as compared to the ssDNA sequences dataset (Fig. 2, Additional files 4 and 5). For what concerns this latter, we observed an improvement of $\sim 10\%$ and $\sim 25\%$ in the percentage of correctly predicted structures ($Apta_D = 0$) for mfold and RNAfold, respectively, when considering the top 5 suboptimal predictions as compared to the single MFE structure. Extending the analysis to the top 10 predicted structures has no clear impact, except when using RNAfold under the Mathews DNA model that leads to a further improvement of $\sim 11\%$. It is interesting to observe that, when considering only the MFE structure, the prediction accuracy of mfold and RNAfold are equivalent; conversely, when suboptimal solutions are included in the analysis, RNAfold can correctly predict a higher number of ssDNA secondary structures ($72\%$) as compared to mfold ($55\%$) (Fig. 2b). When the close to experiments predictions are included ($Apta_D \le 1.5$), mfold poorly benefits from the computation of suboptimal solutions, with an improvement $< 10\%$. Conversely, the percentage of RNAfold predicted structures close to the experimental ones increases by $21\%$, reaching an excellent percentage of $90\%$ when using the Mathews DNA model. A closer look at the experimental structures can explain the remaining wrongly predicted structures. For example, the structures extracted from 5GWL, 5GWQ, 6J37, 6M0B, and 6M0C PDB IDs correspond to a recently characterized DNA folding, called mini-dumbbell [38]. This folding is hardly detected as a possible folding by mfold or RNAfold because its thermodynamics parameters have been determined in the late ’90/early 2000. In addition, this folding is characterized by loops with a length of 2 nucleotides, which are not allowed by both mfold and RNAfold, as previously mentioned. Finally, the interaction with proteins can have a significant impact on the ssNA secondary structure to such an extent that this cannot be predicted even when considering suboptimal solutions.

Also, for RNA sequences, mfold benefits at a lower extent from the computation of suboptimal solutions: we observed an increase of $< 5\%$ in terms of correctly predicted secondary structures, and of $< 10\%$ when including also the predicted structures with an $Apta_D \le 1.5$, independently from the number of suboptimal folding included in the analysis (Fig. 2c). Conversely, with RNAfold, the number of correctly predicted secondary structures increased by about $20\%$, when considering either the top 5 or top 10 suboptimal solutions, reaching a success percentage $> 67\%$. When including the structures predicted close to the reference ($Apta_D \le 1.5$) in the analysis, this increase is less notable ($< 10\%$) and the RNAfold performances are equivalent to those of mfold.

To conclude on the two most commonly used MFE-based tools, it is noteworthy that mfold and RNAfold provide comparable performances for the prediction of ssNAs secondary structures, although mfold seems to be a slightly better choice when dealing with ssDNA sequences. In addition, the results suggest that the thermodynamics models developed for DNA sequences do not positively affect the prediction of their secondary structures. The computation of suboptimal solutions seems to benefit the secondary structure prediction of ssDNA sequences more than RNA sequences, although this might depend on the higher number of ssDNA sequences in complex with proteins as compared to RNA sequences. As previously mentioned, the binding to a molecular target can cause the stabilization of ssNA conformations which are metastable in the free state. Finally, the improvement given by the inclusion of suboptimal solutions of the predictions made by RNAfold is greater than that observed for mfold, probably because of the way the suboptimal solutions are computed, with mfold requiring a suboptimality percentage and RNAsubopt a $\Delta G$ threshold. Overall the obtained results suggest that the thermodynamics parameters could be improved and revised to take into account the recently acquired knowledge on ssNAs secondary structures.

### Accuracy of machine learning and deep learning approaches

**Fig. 5:**
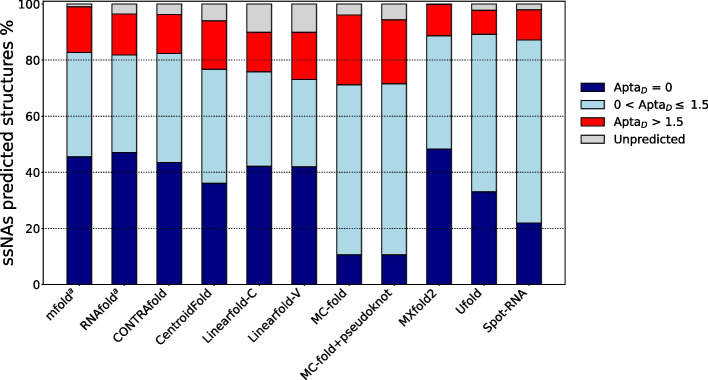
Percentages of correctly predicted ($Apta_D = 0$, blue bars), acceptably predicted ($Apta_D \le 1.5$, light blue bars), and incorrectly predicted ($Apta_D > 1.5$, red bars) ssNA structures by the considered secondary structures prediction tools. Structures predicted as unfolded or unpredicted are represented with grey bars. $^a$Under the RNA model, without suboptimal solutions

Recently artificial intelligence has been intensively exploited to solve biological problems, including the prediction of ssNAs secondary structures. In this context, CONTRAfold implements a knowledge-based algorithm (see “Materials and methods” section), that provides performances comparable to those of the MFE-based methods, in particular RNAfold. Indeed, CONTRAfold correctly predicted $43.5 \%$ of the secondary structures, with this percentage increasing to $82\%$ if the secondary structures predicted with an $Apta_D \le 1.5$ (Fig. 5) are included. As for the MFE-based methods, we did not observe a significant difference between ssDNA or RNA sequences, with 40 and $44\%$ of correctly predicted structures, respectively, increasing to 72% and $84\%$ when including the predictions with an $Apta_D \le 1.5$ (Fig. 5, Additional file 3). CONTRAfold is based on a machine learning model tuned on experimental data. It is therefore not surprising that it struggles in correctly predicting the secondary structures of short sequences ($\le 15$ nucleotides), which are much less frequently characterized. In addition, we found 32 predictions with consecutive open and closed brackets, which is normally penalized by a thermodynamics algorithm by attributing a high energetic score penalty. This is not taken into account by CONTRAfold, and results in predictions with this illegal base pairing pattern, heavily affecting the secondary structure prediction of 5 of the sequences included in the dataset. In addition, like MFE-based methods, CONTRAfold fails in some cases (58, among which 3KTW, 3HXO, 3WC1, 5FJ4, and 6D12) to correctly predict the secondary structure of ssNAs whose experimental secondary structure corresponds to the protein-bound one. Moreover, CONTRAfold does not make use of the extended dot-bracket notation and, therefore, cannot correctly predict structures containing pseudoknots.

This is also a limit of MXfold2 [8], which implements a deep neural network algorithm combined to Turner’s nearest-neighbor free energy parameters. Indeed, it does not implement the extended dot-bracket notation, leading to the incorrect prediction of pseudoknots, and it incorrectly predicted the mini-dumbbell motif. In addition, it sometimes fails in correctly predicting the secondary structures of ssNAs in complex with proteins. Nevertheless, it is the tool providing the best performances, with $\sim 48\%$ of predictions identical to the reference ($Apta_D = 0$) and only $\sim 11\%$ incorrectly predicted ssNAs ($Apta_D > 1.5$) (Fig. 5). Interestingly, MXfold2 is the only tool capable to output a folding for all the ssNA sequences included in the dataset, making it a good option regardless of the length of the ssNA.

Among the selected tools, 3 are able to handle pseudoknots, namely MC-fold, Ufold, and SPOT-RNA. Nevertheless, MC-fold can predict only H-type pseudoknots. In our dataset, this type of structure is present in 17 out of the 77 structures containing pseudoknots. Without specifying the “pseudoknot” keyword (i.e. under the default parameters), none of the H-type pseudoknots was predicted with an $Apta_D \le 1.5$, whilst 6 of the other classes of pseudoknots structures were predicted as close to the reference one. The inclusion of the “pseudoknot” keyword allowed to improve the prediction of 8 of the 17 H-type pseudoknots ($Apta_D \le 1.5$), but it reduced to 3 the other types of pseudoknots predicted with an acceptable distance from the reference. Moreover, it is worth mentioning that many structures have not been predicted by MC-fold due to errors during execution. Finally, MC fold appears to be the least performing tool among the considered ones in terms of both secondary structures predictions accuracy and computational time. Indeed, it correctly predicted only $11\%$ of the structures, and $\sim 30\%$ of the structures were predicted with an $Apta_D > 1.5$ (Fig. 5), and has a runtime of $O(n^{{}^{15}/{}_{2}})$, making it hardly usable for ssNA longer than 100 nucleotides.

Conversely, the performances of UFold and SPOT-RNA in dealing with the prediction of structures containing pseudoknots are better than those of MC-fold. Indeed, UFold showed the best results by predicting 4 out of the 77 pseudoknots with $Apta_D = 0$, and 61 out of 77 pseudoknots with $Apta_D \le 1.5$. SPOT-RNA could not exactly predict any pseudoknot, but 59 out of 77 pseudoknots were predicted as close to the reference ($Apta_D \le 1.5$). In particular, in both cases most of the predictions resulted in an $Apta_D \le 1.0$ (Additional files 13 and 14), or even $\le 0.5$ for UFold, indicating that a very few base pairs were incorrectly predicted. For example, the oligonucleotide with PDB ID 3WC2: the UFold predicted structure has an $Apta_D = 0.091$, because the bases involved in the pseudoknot are shifted by only one position. The SPOT-RNA prediction has a slightly higher $Apta_D$ of 0.915, because the experimentally detected pseudoknot is shifted by 4 positions and an additional pseudoknot between positions 8 and 14 is predicted (Additional file 15). The slightly better performances of UFold in dealing with pseudoknots, as compared to SPOT-RNA, were somehow expected since it already proved its accuracy for this kind on structures [9]. This is observable also on the whole dataset since Ufold was able to correctly predict 33$\%$ of the ssNAs, while SPOT-RNA provided 21.9$\%$ of predictions with $Apta_D = 0$ (Fig. 5). Therefore, if we focus only on the exact predictions, these two tools are globally less performing than MFE-based tools and MXfold2. This is probably due to the fact that these latter methods make use at different extent of thermodynamics parameters, which can take into account relevant factors, such as temperature and salt concentration, known to be fundamental in ssNA folding. In addition, deep learning methods strongly depend on the training dataset, and, therefore, on the available data. Nevertheless, when including predictions close to the reference ($Apta_D \le 1.5$), the UFold and SPOT-RNA performance increases to 89 and 87$\%$, respectively, making them comparable to mfold, RNAfold and MXfold2 (Fig. 5). These results are comparable to those obtained by Fu and coworkers [9] on the dataset taken from the PDB database, although it is important to underline that our dataset includes also ssDNAs and ssNAs in complex with proteins, for which the prediction tools might behave differently, as previously showed. Therefore we believe that there still is space for improvement, thanks also to the constantly increasing number of available experimental data.

### Accuracy of approaches applicable to both MFE- and knowledge-based methods

In addition to MFE-based and knowledge-based approaches, algorithms implementing modifications in the original approaches exist. For example, Linearfold [7] implements an algorithm with a linear time complexity, which makes it suitable for the prediction of long ssNA structures. It is built on both MFE-based and knowledge-based models and it has been explicitly developed for long sequences, for which traditional tools, such as mfold or CONTRAfold, might be time-consuming. Indeed, both Linearfold-C and Linearfold-V equally succeed to find structures close to the reference ($Apta_D \le 1.5$) for long ssNAs (PDB ID 1GRZ (247 nucleotides) and 2R8S (159 nucleotides)), where others have failed, although they have low predictive performance on short sequences ( $\le 15$ nucleotides, $\sim 10\%$ of the dataset), which are predicted as completely unstructured. Therefore, they can represent a good alternative to either mfold/RNAfold or CONTRAfold when dealing with long sequences.

Like Linearfold, CentroidFold [6] can be associated with both MFE-based and knowledge-based models, although, as recommended by the developers, we only tested it under the model implemented by RNAfold. The coupling of this model with the $\gamma$-centroid estimator was able to correctly predict 36 of the ssNA structures contained in the dataset. This percentage increase to $\sim 77\%$ if we include the predictions showing an $Apta_D \le 1.5$. This suggests that the CentroidFold $\gamma$-centroid estimator is slightly less performing compared to the MFE or MEA estimators in finding the optimal solution (i.e. identical to the experiments), although it is as able to provide a solution close to the experimental one ($Apta_D \le 1.5$) as the other estimators.

## Conclusion

Single-stranded nucleic acids (ssNAs) play an important role in cells and, in the form of aptamers binding to different molecular targets, they have potential biotechnological applications and their properties essentially come from their 3D structure or folding. Therefore, being able to predict this latter can be useful to better understand their role within the cells and to exploit them for therapeutic and diagnostic applications. The first level of ssNAs organisation is their secondary structure. Its prediction benefited from the development of numerous approaches over the last decades, going from minimum free energy (MFE)-based approaches to statistical-based or machine learning-based approaches.

In this study, we assessed the performances of 9 free prediction tools, namely mfold [17], RNAfold [5], MC-fold [22], CONTRAfold [21], CentroidFold [6], LinearFold [7], MXfold2 [8], Ufold [9] and SPOT-RNA [10]. To conduct this evaluation, we considered 538 ssNAs secondary structures retrieved from the PDB and NDB databases. The dataset included both ssDNA and RNA sequences, with a length range from 7 to 1509 nucleotides, and a wide range of structural motifs spanning from simple hairpins to pseudoknots. We also included ssNAs whose structure has been determined in complex with their target protein. We used as comparison metrics AptaMat, a ssNA secondary structure comparison algorithm we have recently developed. We observed that only $4.3\%$ of the considered ssNAs secondary structures (PDB IDs: 2F87, 1ESH, 1I46, 1JZC, 6FK5, 1IK1, 2Y95, 1UUT, 2LPA, 1JWC, 4QIL, 1ATV, 3Q0A, 1A1T, 1K6G, 4A4S, 1JVE, 1YTB, 2LUP, 4OOG, 6U82, 3THW) are correctly predicted by all the aforementioned prediction tools. These ssNAs share a common secondary structure, which includes a hairpin/stem-loop, with a long hairpin stem of 4 to 14 base pairs, and a hairpin loop of 3 to 5 nucleotides. When considering predictions close to the experimental structure ($Apta_D \le 1.5$), this percentage increases to $56.9\%$. In addition, at least one of the secondary structure prediction tools can provide a prediction identical and close to the experimental secondary structure in $63.9\%$ and $98.7\%$ of the cases, respectively.

The MFE-based tools, namely mfold and RNAfold, remain among the best performing prediction tools, with $\sim 46\%$ and $47\%$ of exact predictions for mfold and RNAfold, respectively. These percentages increase to $83\%$ and $82\%$, when including predictions close to the experiments ($Apta_D \le 1.5$). Nevertheless, a knowledge-based tool, CONTRAfold, and a recently published software, MXfold2, that is based on deep learning coupled with thermodynamics models, showed similar results. This indicates that, as it has been shown for many other bioinformatics challenges [39–41], artificial intelligence has a high potential to solve the question of ssNAs folding prediction. This is also shown by two recent tools exploiting deep learning methods, namely UFold and SPOT-RNA: although for our dataset they provided a low percentage of exact predictions (33 $\%$ and 21.9$\%$, respectively), they predicted 89$\%$ and 87$\%$, respectively, of structures close to the experimental one ($Apta_D \le 1.5$), performing slightly better than the MFE-based methods.

Overall, particular structural motifs are a major limitation to the performance of the prediction tools. In particular, most of them do not implement the extended dot-bracket notation and models capable to describe pseudoknots. UFold, and at a slighter minor extent SPOT-RNA, are the only tools providing acceptable predictions for this kind of structures, with 65 out of 77 and 59 out of 77 predictions with an $Apta_D \le 1.5$, respectively. Conversely, MC-fold could not provide any significant improvement in the predictions of the pseudoknotted structures of the dataset, and it has a high runtime, making it the least performing prediction tool. For MFE-based methods, including the possibility of handling pseudoknots would require a huge effort. Therefore, in our opinion the only way to deal with this kind of structure is to use artificial intelligence, thanks to the increasing number of experimentally available ssNAs structures. Mini-dumbbell, a quite recently characterized ssNA motif, is also hard to correctly predict since the number of mini-dumbell structures is limited and adequate parameters are yet to be determined. Moreover, among the tools developed for the prediction of RNA secondary structures, only mfold and RNAfold propose parameters specific to ssDNA sequences. Nevertheless, we have shown that the use of DNA thermodynamics parameters did not allow the improvement of the prediction of ssDNA secondary structures. Therefore, there is still work to be done to accurately handle this type of ssNA with a high biotechnological potential. For the time being, our study suggests to use mfold under the default parameters when predicting ssDNA secondary structures, since it is the tool providing the highest percentage of correctly predicted ssDNA secondary structures.

Another limit of the herein considered tools is the prediction of secondary structures of the ssNA in complex with proteins. This was somehow expected, since the most stable/probable ssNA conformation in the free state might be very different from the one of the bound state, thanks to the intrinsic ssNAs flexibility. A possible option to address this issue is given by the computation of suboptimal solutions, which is possible when using mfold and RNAfold. In particular, including the top 5 suboptimal solutions in our analysis brought the percentage of correctly predicted structures to $49\%$ and $64\%$ with mfold and RNAfold, respectively. If we consider the structures predicted as close to the reference ($Apta_D \le 1.5$), these percentages increase to $90\%$ and $88\%$ with mfold and RNAfold, respectively. Nevertheless, when dealing with ssNAs in complex with a molecular target, working on the 3D structure rather than the 2D structure is highly recommended; indeed, molecular recognition occurs at a 3D level. Therefore, in this case, a possible approach would be the computation of multiple suboptimal solutions in order to determine the most probable base pairs patterns and, then, use them to predict the ssNA 3D structure in complex with the molecular target using advanced techniques, such as enhanced sampling molecular dynamics simulations (article in preparation).

In conclusion, in most cases MXfold2 currently represents the best tool for RNA secondary structure prediction, since it provided the highest number of exact predictions, and RNAfold including 5 suboptimal solutions might confirm or help the prediction. Nevertheless, Ufold and SPOT-RNA, with 89$\%$ and 87$\%$ of prediction with an $Apta_D \le 1.5$, respectively, can represent a good alternative, since only a few base pairs would be incorrectly predicted. When pseudoknots are expected to occur, UFold would be the best option. Conversely, when dealing with ssDNA sequences mfold under the default parameters should be chosen. Future work needs to be dedicated to the development of a new tool based on both deep learning and thermodynamics overcoming the highlighted issues and providing a correct prediction regardless of the ssNAs properties.

### Supplementary Information

**Additional file 1.** Example of the performance of AptaMat distance in discriminating and correctly ranking close ssNA secondary structures as compared to commonly used metrics, namely F1 score, MCC and RNAdistance.

**Additional file 2.** SsNAs dataset. The PDB code, the type of ssNA, the experimental obtention method, the presence of a binding protein in the original structure, the ssNAs size, and the experimental secondary structures are reported.

**Additional file 3.** Comparison between predicted and experimental secondary structure using the AptaMat distance as a metric for CONTRAfold, CentroidFold, Linearfold, MC-fold, MXfold2, UFold, and SPOT-RNA. The PDB code is reported in the first column. $Apta_D$ values are reported for each PDB and software prediction. "/" characters indicate either structures predicted as unfolded or software failure during the computation.

**Additional file 4.** Comparison between predicted and experimental secondary structure using the AptaMat distance as a metric for RNAfold under RNA (Turner (2004)) and DNA (Mathews (2004)) model. The PDB code is reported in the first column.$Apta_D$ values are reported for each PDB and associated optimal/suboptimal prediction. "/" characters indicate either structures predicted as unfolded or software failure during the computation.

**Additional file 5.** Comparison between predicted and experimental secondary structure using the AptaMat distance as a metric for mfold under RNA (Mathews (1999)) and DNA (SantaLucia (1998)) model. The PDB code is reported in the first column. $Apta_D$ values are reported for each PDB and associated optimal/suboptimal prediction. "/" characters indicate either structures predicted as unfolded or software failure during the computation.

**Additional file 6.** Predicted secondary structure for RNAfold under RNA (Turner (2004)) and DNA (Mathews (2004)) model in the dot-bracket notation. The PDB code is reported in the first column. "/" characters indicate either structures predicted as unfolded or software failure during the computation or sequences for which the parameters were not applied.

**Additional file 7.** Predicted secondary structure for mfold under RNA (Mathews (1999)) and DNA (SantaLucia (1998)) model in the dot-bracket notation. The PDB code is reported in the first column. "/" characters indicate either structures predicted as unfolded or software failure during the computation or sequences for which the parameters were not applied.

**Additional file 8.** Predicted secondary structure for CONTRAfold, CentroidFold, Linearfold, MC-fold, MXfold2, UFold and SPOT-RNA in the dot-bracket notation. The PDB code is reported in the first column. "/" characters indicate either structures predicted as unfolded or software failure during the computation or sequences for which the parameters were not applied.

**Additional file 9.** Percentages of correctly predicted ($Apta_D = 0$ , blue bars), acceptably predicted ($Apta_D \leq 1.5$, light blue bars), and incorrectly predicted ($Apta_D > 1.5$, red bars) ssDNA structures by the considered secondary structures prediction tools. Structures predicted as unfolded or unpredicted are represented with grey bars.

**Additional file 10.** Percentages of correctly predicted ($Apta_D = 0$, blue bars), acceptably predicted ($Apta_D \leq 1.5$, light blue bars), and incorrectly predicted ($Apta_D > 1.5$, red bars) RNA structures by the considered secondary structures prediction tools. Structures predicted as unfolded or unpredicted are represented with grey bars.

**Additional file 11.** Percentages of correctly predicted (MCC = 1, blue bars), acceptably predicted ($MCC \geq 0.5$, light blue bars), and incorrectly predicted (MCC < 0.5, red bars) ssNA structures by the considered secondary structure prediction tools. Structures predicted as unfolded or unpredicted are represented with grey bars.

**Additional file 12.** Percentages of correctly predicted (F1 score = 1, blue bars), acceptably predicted ($F1 \, score \geq 0.5$, light blue bars), and incorrectly predicted (F1 score < 0.5,  red bars) ssNA structures by the considered secondary structure prediction tools. Structures predicted as unfolded or unpredicted are represented with grey bars.

**Additional file 13.** Example of the difference in the classification of the predicted structures using AptaMat, F1 score and MCC as metrics. The experimental structure corresponds to the 2NC1 PDB code, the predicted structure are those obtained by MXfold2, MC-fold, and SPOT-RNA, respectively.

**Additional file 14.** Histogram of the AptaD obtained for the UFold predictions of the structures containing pseudoknots (the 4 predictions with AptaD > 5 were discarded to facilitate the plot reading).

**Additional file 15.** Histogram of the AptaD obtained for the SPOT-RNA predictions of the structures containing pseudoknots (the 6 predictions with AptaD > 5 were discarded to facilitate the plot reading).

**Additional file 16.** Example of pseudoknot-containing secondary structure predicted by UFold and SPOT-RNA and aligned to the experimental one (code PDB 3WC2).

## Data Availability

All data generated or analyzed during this study are available upon reasonable request from the corresponding author.

## Abbreviations

ssNA: Single stranded oligonucleotides
MFE: Minimum free energy
