# Supplementary material for: Comparative Study of Single-stranded Oligonucleotides Secondary Structure Prediction Tools
Source: BMC Bioinformatics. 2023 Nov 8;24:422. doi: 10.1186/s12859-023-05532-5 (PMC10634105; doi:10.1186/s12859-023-05532-5)
Supplement: Supplementary file 1 — Additional file 1. Example of the performance of AptaMat distance in discriminating and correctly ranking close ssNA secondary structures as compared to commonly used metrics, namely F1 score, MCC and RNAdistance. [file 12859_2023_5532_MOESM1_ESM.pdf]

Additional File 1. Example of the performance of AptaMat distance in discriminating and correctly ranking close ssNA secondary structures as compared to commonly used metrics, namely F1 score, MCC, and RNAdistance.

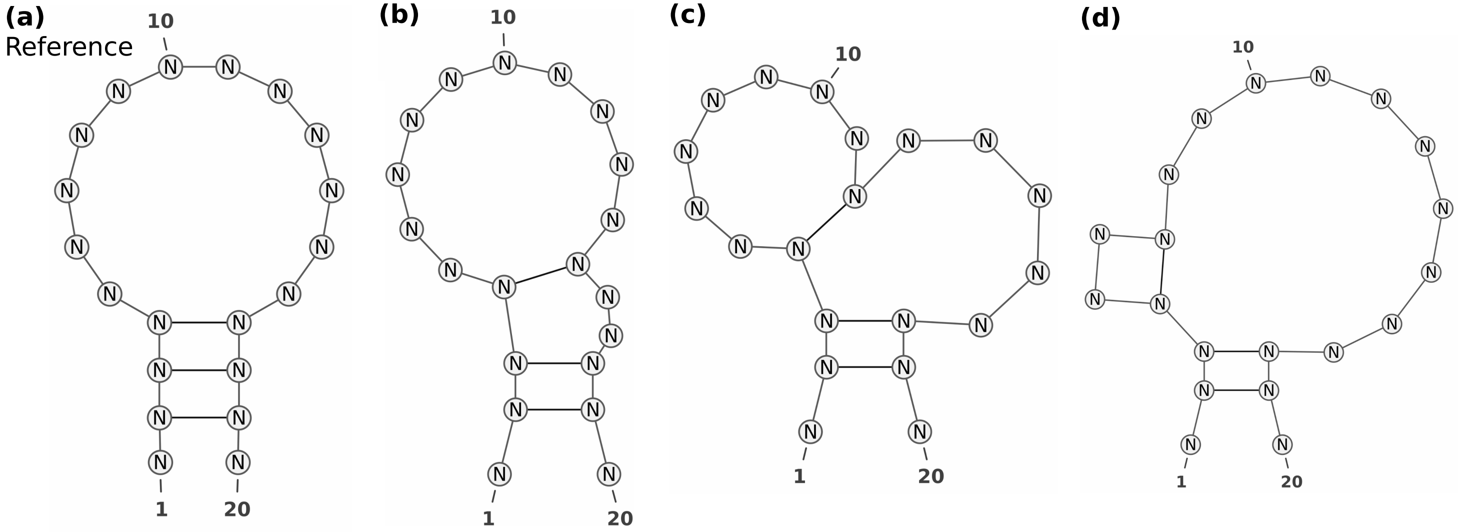

	(a) .(((............))).	(b) .(((..........)..)).	(c) .(((.......).....)).	(d) .(((..)..........)).
Hamming distance		0.10	0.10	0.10
RNAdistance		4	4	4
F1 score		0.67	0.67	0.67
MCC		0.60	0.60	0.60
AptaMat		0.67	1.17	2.00
