# Supplementary material for: Comparative Study of Single-stranded Oligonucleotides Secondary Structure Prediction Tools
Source: BMC Bioinformatics. 2023 Nov 8;24:422. doi: 10.1186/s12859-023-05532-5 (PMC10634105; doi:10.1186/s12859-023-05532-5)
Supplement: Supplementary file 2 — Additional file 2. SsNAs dataset. The PDB code, the type of ssNA, the experimental obtention method, the presence of a binding protein in the original structure, the ssNAs size, and the experimental secondary structures are reported. [file 12859_2023_5532_MOESM2_ESM.pdf]

**Additional File 2**. SsNAs dataset. The PDB code, the type of ssNA, the experimental obtention method, the presence of a binding protein in the original structure, the ssNAs size, and the experimental secondary structures are reported.					
**PDB**	**NA**	**Method**	**Complex**	**Size (nt)**	**Experimental secondary structure**
1PQT	DNA	NMR	No	7	((...))
2K71	DNA	NMR	No	8	((....))
5GWL	DNA	NMR	No	8	(..)(..)
5GWQ	DNA	NMR	No	8	(..)(..)
6J37	DNA	NMR	No	8	(..)(..)
6M0B	DNA	NMR	No	8	(..)(..)
6M0C	DNA	NMR	No	8	(..)(..)
5OND	DNA	X-RAY	Yes	9	((....).)
1ZHU	DNA	NMR	No	10	..((...)).
2A0I	DNA	X-RAY	Yes	10	....(..)..
2LO8	DNA	NMR	No	10	(((...).))
3WPD	DNA	X-RAY	Yes	10	((.....)).
6IY5	DNA	NMR	No	10	((...))...
1BJH	DNA	NMR	No	11	((((...))))
3WPG	DNA	X-RAY	Yes	11	((.....))..
2LO5	DNA	NMR	No	12	((((...).)))
3WPH	DNA	X-RAY	Yes	12	((.....))...
6FKE	DNA	X-RAY	Yes	12	.((((...))))
1LA8	DNA	NMR	No	13	(((((...)))))
1P0U	DNA	NMR	No	13	(((((...)))))
2EXF	DNA	NMR	Yes	14	..(((.....))).
2JZW	DNA	NMR	Yes	14	..(((.....))).
5F55	DNA	X-RAY	Yes	14	......(....)..
6FK5	DNA	X-RAY	Yes	14	(((((...))))).
1UUT	DNA	X-RAY	Yes	15	((((((...))))))
2M8Y	DNA	NMR	No	15	((((((...))))))
1AC7	DNA	NMR	No	16	((((((....))))))
6FK4	DNA	X-RAY	Yes	16	..(((((...))))).
1XUE	DNA	NMR	No	17	((...((...))...))
1EN1	DNA	NMR	No	18	(.(((.....))))....

PDB	NA	Method	Complex	Size (nt)	Experimental secondary structure
4KB0	DNA	X-RAY	Yes	18	((((((....)))))..)
4KB1	DNA	X-RAY	Yes	18	((((((....)))))..)
1ECU	DNA	NMR	No	19	((((((((...))))))))
3Q0A	DNA	X-RAY	Yes	20	.......(((((...)))))
4FF1	DNA	X-RAY	Yes	20	.......(((((...)))))
3C46	DNA	X-RAY	Yes	21	.......(((((...))))).
3Q23	DNA	X-RAY	Yes	21	.......(((((...))))).
2A6O	DNA	X-RAY	Yes	22	((((((((....)))).)))).
3Q24	DNA	X-RAY	Yes	22	........(((((...))))).
2L5K	DNA	NMR	No	23	(((...((((...))))...)))
3DSD	DNA	X-RAY	Yes	23	.(((((((....)))))))....
2VHG	DNA	X-RAY	Yes	24	((((((((....)))).))))...
1OSB	DNA	X-RAY	Yes	25	..(((((....)))))).........
1ZM5	DNA	X-RAY	Yes	25	..(((((....)))))).........
2CDM	DNA	X-RAY	Yes	25	.(((((....))))).....(..).
2VIC	DNA	X-RAY	Yes	26	....((((((((.....)))).))))
5N2Q	DNA	X-RAY	Yes	26	(((((((....)))))))........
1JVE	DNA	NMR	No	27	((((((((((((...))))))))))))
1NGO	DNA	NMR	No	27	(((((((((((.....)))))))))))
1NGU	DNA	NMR	No	27	((((..(((((.....)))))..))))
3ZH2	DNA	X-RAY	Yes	27	(((((.......((....))..)))))
4HT4	DNA	X-RAY	Yes	28	.(((((((...)))))))..........
1YTB	DNA	X-RAY	Yes	29	((((((((((((.....))))))))))))
1B4Y	DNA	NMR	No	30	..........((((((....))))))....
4ER8	DNA	X-RAY	Yes	32	......(((((..(((((..)))))..)))))
4F41	DNA	X-RAY	Yes	32	(((((((((((((((..)))))))))))))))
4F43	DNA	X-RAY	Yes	32	(((((((((((((((..)))))))))))))))
5HRU	DNA	X-RAY	Yes	32	((((.....[[..(((((]]...)))))))))
6SEI	DNA	X-RAY	Yes	32	.(((.(((((((((...)))))))..)).)))
5HTO	DNA	X-RAY	Yes	34	(((((.....[[..(((((]]...))))))))))
2VJU	DNA	X-RAY	Yes	35	((..((((((((....)))).))))......))..
1EZN	DNA	NMR	No	36	(((((((((((..))))((((..))))..)))))))

PDB	NA	Method	Complex	Size (nt)	Experimental secondary structure
1SNJ	DNA	NMR	No	36	(((((((((((..))))((((..))))..)))))))
6U82	DNA	X-RAY	Yes	38	((((((((((((((((...))))))))))...))))))
3HXO	DNA	X-RAY	Yes	40	.(((((..(.(((...))))...(((.....))).)))))
2N8A	DNA	NMR	Yes	45	(((((((((....))))))))).((((((((((..))))))))))
3THW	DNA	X-RAY	Yes	53	(((((((((((((((((((((((...)))))))))....))))))))))))))
2IXZ	RNA	NMR	No	8	((...).)
2OJ7	RNA	NMR	No	8	((....))
1IDV	RNA	NMR	No	10	(((....)))
1R4H	RNA	NMR	No	10	.(((..))).
2MXJ	RNA	NMR	No	11	((......)).
1AFX	RNA	NMR	No	12	((((....))))
1RNG	RNA	NMR	No	12	(((((..)))))
1ZIF	RNA	NMR	No	12	((((....))))
1ZIG	RNA	NMR	No	12	((((....))))
1ZIH	RNA	NMR	No	12	((((....))))
2F87	RNA	NMR	No	12	((((....))))
5FMZ	RNA	X-RAY	Yes	12	.((....))...
1ESH	RNA	NMR	No	13	(((((...)))))
1HS1	RNA	NMR	No	13	(((......))).
1HS2	RNA	NMR	No	13	(((......))).
1HS3	RNA	NMR	No	13	(((......))).
1HS4	RNA	NMR	No	13	(((......))).
1HS8	RNA	NMR	No	13	(((......))).
1I46	RNA	NMR	No	13	(((((...)))))
1I4B	RNA	NMR	No	13	(((((...)))))
1JZC	RNA	NMR	No	13	(((((...)))))
1VOP	RNA	NMR	No	13	((((.....))))
4Z0C	RNA	X-RAY	Yes	13	...(......)..
6FQ3	RNA	X-RAY	Yes	13	(((((...)))))
6FQL	RNA	X-RAY	Yes	13	(((((...)))))
1F85	RNA	NMR	No	14	(((((....)))))
1FHK	RNA	NMR	No	14	(((........)))

PDB	NA	Method	Complex	Size (nt)	Experimental secondary structure
1IK1	RNA	NMR	No	14	(((((...))))).
1K4A	RNA	NMR	No	14	(((((....)))))
1K4B	RNA	NMR	No	14	(((((....)))))
1ROQ	RNA	NMR	No	14	(((((....)))))
2EVY	RNA	NMR	No	14	((((......))))
2KOC	RNA	NMR	No	14	(((((....)))))
2Y95	RNA	NMR	No	14	(((((....)))))
4Z7L	RNA	X-RAY	Yes	14	((..........))
1A4T	RNA	NMR	Yes	15	(((((.....)))))
1ATW	RNA	NMR	No	15	(((((....))))).
1OQ0	RNA	NMR	No	15	((((((...))))))
1Q75	RNA	NMR	No	15	(((((.....)))))
1QFQ	RNA	NMR	Yes	15	(((((.....)))))
1XWP	RNA	NMR	No	15	((((......)))).
2LPA	RNA	NMR	No	15	((((((...))))))
4AL7	RNA	X-RAY	Yes	15	(((((.....)))))
1JTW	RNA	NMR	No	16	.(((((....))))).
1JWC	RNA	NMR	No	16	((((((....))))))
1XWU	RNA	NMR	No	16	((((.(...).)))).
2L6I	RNA	NMR	No	16	((((((..).))))).
2LP9	RNA	NMR	No	16	((((((...).)))))
2MNC	RNA	NMR	No	16	((((.(.....)))))
4AL5	RNA	X-RAY	Yes	16	.(((((.....)))))
4ILM	RNA	X-RAY	Yes	16	.....(((.....)))
4QIL	RNA	X-RAY	Yes	16	.((((((...))))))
1ATV	RNA	NMR	No	17	((((((....)))))).
1BZ2	RNA	NMR	No	17	(((((.......)))))
1BZ3	RNA	NMR	No	17	(((((.(...).)))))
1KKA	RNA	NMR	No	17	((((((.....))))))
1WKS	RNA	NMR	No	17	((((((.....))))))
1YN1	RNA	NMR	No	17	((((((.....))))))
2JR4	RNA	NMR	No	17	(((((.......)))))

PDB	NA	Method	Complex	Size (nt)	Experimental secondary structure
2KPC	RNA	NMR	No	17	((((((.....))))))
2KPD	RNA	NMR	No	17	(((((.......)))))
2KRP	RNA	NMR	No	17	(((((.......)))))
2KVN	RNA	NMR	No	17	(((((......))))).
2LAC	RNA	NMR	No	17	(((((.......)))))
2LBJ	RNA	NMR	No	17	(((((((...)))))))
2LBK	RNA	NMR	No	17	((((((.....))))))
2LBL	RNA	NMR	No	17	(((((.......)))))
2M4W	RNA	NMR	No	17	((..((....))...))
4ZLD	RNA	X-RAY	Yes	17	.((((((...)))))).
6CYT	RNA	X-RAY	Yes	17	......(((...).)).
1Z30	RNA	NMR	No	18	(((((((....)))))))
2GVO	RNA	NMR	No	18	((((((......))))))
2QH4	RNA	NMR	No	18	((((((......))))))
2Y9H	RNA	X-RAY	Yes	18	(((((((....)))))))
1ATO	RNA	NMR	No	19	((((((.......))))))
1ESY	RNA	NMR	No	19	((((.((....))..))))
1I3X	RNA	NMR	No	19	(((((((.....)))))))
1SLP	RNA	NMR	No	19	(((((((....)).)))))
1UUU	RNA	NMR	No	19	.((((((.....)))))).
2B6G	RNA	NMR	Yes	19	((((((((..).)))))))
2B7G	RNA	NMR	No	19	(((((((.....)))))))
2MEQ	RNA	NMR	No	19	((((((.......))))))
2MFD	RNA	NMR	No	19	(((((((.....)))))))
2RLU	RNA	NMR	No	19	(((((((.....)))))))
2Y8Y	RNA	X-RAY	Yes	19	.((((((....))))))..
4QI2	RNA	X-RAY	Yes	19	..((((((...))))))..
5N5C	RNA	NMR	No	19	.((((((.....)))))).
6TQB	RNA	X-RAY	Yes	19	((((((((...))))))))
1A1T	RNA	NMR	Yes	20	((((((((....))))))))
1HLX	RNA	NMR	No	20	((((((((....))))))))
1MFJ	RNA	NMR	No	20	..(((((((....)))))))

PDB	NA	Method	Complex	Size (nt)	Experimental secondary structure
1U2A	RNA	NMR	No	20	(((((((......)))))))
2JPP	RNA	NMR	Yes	20	(((((((......)))))))
2O33	RNA	NMR	No	20	(((..(((....)))..)))
2RPK	RNA	NMR	No	20	(((((((......)))))))
2RPT	RNA	NMR	No	20	((((.(((....))).))))
2Y8W	RNA	X-RAY	Yes	20	.((((((....))))))...
4L8H	RNA	X-RAY	Yes	20	((((.((((...))))))))
5F5F	RNA	X-RAY	Yes	20	(((((.(......).)))))
6PK9	RNA	NMR	No	20	(((((((......)))))))
17RA	RNA	NMR	No	21	(((((..((....)).)))))
1D0U	RNA	NMR	No	21	((((((.((....))))))))
1JOX	RNA	NMR	No	21	(((((.((......)))))))
1QWA	RNA	NMR	No	21	((((((((....)).))))))
1RKJ	RNA	NMR	Yes	21	(((((((......).))))))
1SZY	RNA	NMR	No	21	(((((((.......)))))))
2FY1	RNA	NMR	Yes	21	((((((((.....))))))))
2M21	RNA	NMR	No	21	(((((((.......)))))))
2MFF	RNA	NMR	Yes	21	((((((((.....))))))))
2MFG	RNA	NMR	Yes	21	((((((((.....))))))))
5F5H	RNA	X-RAY	Yes	21	(((((((......)))).)))
5ID6	RNA	X-RAY	Yes	21	....(((((.....)))))..
5L1Z	RNA	X-RAY	Yes	21	......((........))...
6XWJ	RNA	NMR	No	21	(((((((((...)))))))))
1F9L	RNA	NMR	No	22	((((..(((....)))..))))
1FJE	RNA	NMR	Yes	22	((((..............))))
1IKD	RNA	NMR	No	22	(((((((....)))))))....
1JUR	RNA	NMR	No	22	((((((((....)))))..)))
1K2G	RNA	NMR	No	22	(.(((....))))((....)).
1K6G	RNA	NMR	No	22	(((((((((....)))))))))
1K6H	RNA	NMR	No	22	(((((((((....)))))))))
1N66	RNA	NMR	No	22	((((..(((....)))..))))
1OSW	RNA	NMR	No	22	((((.(((....)))...))))

PDB	NA	Method	Complex	Size (nt)	Experimental secondary structure
1PJY	RNA	NMR	No	22	(((((((((....)))))))))
1TJZ	RNA	NMR	No	22	(((..((((....))))..)))
2G1W	RNA	NMR	No	22	((((.[[[.)))).....]]].
2GRW	RNA	NMR	No	22	((((.((((....)))).))))
2GV3	RNA	NMR	No	22	(((((((.(.....))))))))
2GV4	RNA	NMR	No	22	(((((.(((....))).)))))
2HNS	RNA	NMR	No	22	(((((((((....)))))))))
2JSE	RNA	NMR	No	22	((((..(((....)))..))))
2JYM	RNA	NMR	No	22	(((((.((((..).))))))))
2K66	RNA	NMR	No	22	(((((((((....)))))))))
2KD8	RNA	NMR	No	22	(((((((((....)))))))))
2M5U	RNA	NMR	No	22	(((((((((....)))))))))
2MFC	RNA	NMR	Yes	22	(((((((........)))))))
2MFE	RNA	NMR	Yes	22	((((((((......))))))))
2W2H	RNA	X-RAY	Yes	22	..((((.(......).))))..
4A4S	RNA	NMR	No	22	(((((((((....)))))))))
6F4H	RNA	X-RAY	Yes	22	((((((..........))))))
6KYV	RNA	X-RAY	Yes	22	(((((((((....)))))))))
1BGZ	RNA	NMR	No	23	((((..((((....)).))))))
1BVJ	RNA	NMR	No	23	(((((.(((......))))))))
1JTJ	RNA	NMR	No	23	(((((((.........)))))))
1K5I	RNA	NMR	No	23	(((((((((.....)))))))))
1MFK	RNA	NMR	No	23	((((((.(((....)))))))))
1OW9	RNA	NMR	No	23	((((...(((....)))..))))
1S2F	RNA	NMR	No	23	((((((.(((....)))))))))
1TLR	RNA	NMR	No	23	((((...(((....)))..))))
2ANN	RNA	X-RAY	Yes	23	(((...............)))..
2ES5	RNA	NMR	No	23	((((((((((..).)))))))))
2M12	RNA	NMR	No	23	((((((..(.......)))))))
2M22	RNA	NMR	No	23	(((((.(((.....))).)))))
2N0R	RNA	NMR	No	23	(...((((....))))......)
2N2O	RNA	NMR	No	23	....((((((...))))))....

PDB	NA	Method	Complex	Size (nt)	Experimental secondary structure
2N2P	RNA	NMR	No	23	....((((((...))))))....
2N3O	RNA	NMR	Yes	23	(((((((((.....)))))))))
2N7X	RNA	NMR	No	23	(((((..((.....))..)))))
2N82	RNA	NMR	Yes	23	.((((.............)))).
2PJP	RNA	X-RAY	Yes	23	(((((.(((((..))))))))))
2QH3	RNA	NMR	No	23	((((((((.......))))))))
2RO2	RNA	NMR	No	23	(((((((((.....)))))))))
2UWM	RNA	X-RAY	Yes	23	((((.(((((..).)))).))))
3PHP	RNA	NMR	No	23	((((((((......)))))))).
5UF3	RNA	NMR	No	23	(((((((.((....)))))))))
5WQ1	RNA	NMR	No	23	(((((((((.....)))))))))
6GBM	RNA	NMR	Yes	23	(((((((.(......))))))))
1A9N	RNA	X-RAY	Yes	24	(((((.............))))).
1E4P	RNA	NMR	No	24	((((...(((....)))...))))
1KKS	RNA	NMR	No	24	.(..((((((....))))))..).
1MT4	RNA	NMR	No	24	((((((((........))))))))
1NC0	RNA	NMR	No	24	((((((((.......)).))))))
1NYB	RNA	NMR	Yes	24	(((((((((......)))))))))
1RHT	RNA	NMR	No	24	.((.(((.((....))))).))..
1SYZ	RNA	NMR	No	24	(((((.(((.....)))..)))))
1TFN	RNA	NMR	No	24	..(((((.((....)))))))...
2HEM	RNA	NMR	No	24	(((..(((((....)))))..)))
2LK3	RNA	NMR	No	24	((((..(((((....)))))))))
2LV0	RNA	NMR	No	24	((((((((........))))))))
2QH2	RNA	NMR	No	24	(((((((((.....).))))))))
3NVK	RNA	X-RAY	Yes	24	..(.(...(......)......).
5F9F	RNA	X-RAY	Yes	24	((((((((((....))))))))))
5NG6	RNA	X-RAY	Yes	24	....(((((....)))))......
5UDZ	RNA	X-RAY	Yes	24	((((((.......)))))).....
1M82	RNA	NMR	No	25	((((((.(((....)))..))))))
1QC8	RNA	NMR	No	25	(.(.((((((......))))))).)
6DU5	RNA	X-RAY	Yes	25	..(.(((.............))).)

PDB	NA	Method	Complex	Size (nt)	Experimental secondary structure
6F4G	RNA	X-RAY	Yes	25	((((((((...........))))))
1QWB	RNA	NMR	No	26	((((((..............))))))
2L5Z	RNA	NMR	No	26	((((((...(((....))).))))))
4BW0	RNA	X-RAY	Yes	26	(((...(((....)))......))).
4QOZ	RNA	X-RAY	Yes	26	.....((((((....))))))).....
4TV0	RNA	X-RAY	Yes	26	.......((((((....))))))...
1F7F	RNA	NMR	No	27	(((((.((((((....)))))))))))
1FQZ	RNA	NMR	No	27	(((.....(((......)))....)))
1FYO	RNA	NMR	No	27	((((.(.((((....))))..).))))
1XSG	RNA	NMR	No	27	(((((.((((((....)))))))))))
1XSH	RNA	NMR	No	27	((((((.(((((....)))))))))))
1YSV	RNA	NMR	No	27	(((((((((((.....)))))))))))
2AHT	RNA	NMR	No	27	((((((.((((....))))..))))))
2IXY	RNA	NMR	No	27	(((((((((((...).)))))).))))
2LDL	RNA	NMR	No	27	((((((.(((.......))).))))))
2LJJ	RNA	NMR	No	27	((((...(((((.....))))).))))
2LQZ	RNA	NMR	No	27	(((((....((((....)))).)))))
484D	RNA	NMR	Yes	27	(((((((..((.......)))))))))
6XH0	RNA	X-RAY	Yes	27	(((((...(((((...).)))))))))
1ZBN	RNA	NMR	Yes	28	((((((.(.((((....)))))))))))
28SP	RNA	NMR	No	28	(((((....(((....)))....)))))
2GIP	RNA	NMR	No	28	(((((..(((((....)))))..)))))
2KMJ	RNA	NMR	No	28	(((((((..((((....)))))))))))
2LUN	RNA	NMR	No	28	((((.(..((((....))))..).))))
2NC0	RNA	NMR	No	28	(((((...(((((....))))).)))))
2NCI	RNA	NMR	No	28	(((((......(((....)))..)))))
5M0I	RNA	X-RAY	Yes	28	((((......((....))...))))...
6AAS	RNA	NMR	No	28	((((((((((((....))))))))))))
6SNJ	RNA	NMR	Yes	28	((((((((((.......)))))).))))
6VZC	RNA	NMR	No	28	((((((..((((....))))..))))))
1ANR	RNA	NMR	No	29	(((((....((((......)))).)))))
1EBS	RNA	NMR	No	29	((((..((((((....))))))...))))

PDB	NA	Method	Complex	Size (nt)	Experimental secondary structure
1F84	RNA	NMR	No	29	.(((.....(((......)))....))).
1JBT	RNA	X-RAY	Yes	29	((((((......(....).....))))))
1L1C	RNA	NMR	Yes	29	((.((((..((((....))))))))..))
1L1W	RNA	NMR	No	29	((((..((((((....))))))..)))).
1NBR	RNA	NMR	No	29	((((((.((((((...).)))))))))))
1OOA	RNA	X-RAY	Yes	29	(((((....((((....))))...)))))
1SCL	RNA	NMR	No	29	((((((......(....).....))))))
2GIO	RNA	NMR	No	29	(((((...(.(((....))).)..)))))
2JWV	RNA	NMR	No	29	(((((....((((....))))...)))))
2K5Z	RNA	NMR	No	29	(((((((((.((....)).))))).))))
2M24	RNA	NMR	No	29	(((((((((...........)))))))))
3SN2	RNA	X-RAY	Yes	29	((((((.((((((...).)))))))))))
5LM7	RNA	X-RAY	Yes	29	..............(((((.....)))))
5LSN	RNA	NMR	Yes	29	((.((((((((((...)))))))))).))
6DU4	RNA	X-RAY	Yes	29	(((((((...............)))))))
1AUD	RNA	NMR	Yes	30	(((((.((((....)))).......)))))
1EBR	RNA	NMR	No	30	((((..((((((....)))).))...))))
1EKZ	RNA	NMR	Yes	30	((((((((((((((..))))))))))))))
1HVU	RNA	X-RAY	Yes	30	...[.[[[..((((((]]].]...))))))
1HWQ	RNA	NMR	No	30	(((((...((((......))))...)))))
1KP7	RNA	NMR	No	30	((((.((....(((....)))..)).))))
1LDZ	RNA	NMR	No	30	(((((....(((((....)))))..)))))
1NA2	RNA	NMR	No	30	((((((....((.....))....)))))).
1RFR	RNA	NMR	No	30	(((((...(((((....)))))...)))))
5Y58	RNA	X-RAY	Yes	30	((((((..((((.......)))))))))).
6MCE	RNA	NMR	Yes	30	(((((((..(((((...).)))))))))))
1JO7	RNA	NMR	No	31	((.((((.(..(((....))).).)))).))
1MFY	RNA	NMR	No	31	..(.((((..((((....))))..)))))..
1YNC	RNA	NMR	No	31	((((((.((((........))))..))))))
1YNG	RNA	NMR	No	31	.(((((.((((........))))..))))).
2LDT	RNA	NMR	No	31	(((((((..((((....))))...)))))))
5KMZ	RNA	NMR	No	31	[[[[..{((((.(((]]]]....)))}))))

PDB	NA	Method	Complex	Size (nt)	Experimental secondary structure
5UZT	RNA	NMR	No	31	(((((((.((............)))))))))
6HYK	RNA	NMR	No	31	(((((......((((....))))...)))))
1G70	RNA	NMR	Yes	32	((((((..(((((....))).))...))))))
1KAJ	RNA	NMR	No	32	(((((...[[[[..)))))........]]]].
1KPD	RNA	NMR	No	32	(((((...[[[[[.))))).......]]]]].
1XHP	RNA	NMR	No	32	((((.((((.(((.....)))..)))).))))
1Z31	RNA	NMR	No	32	(((((.(((..(((....)))...))))))))
2LBS	RNA	NMR	Yes	32	((((((((((((((....))))))))))))))
2LI4	RNA	NMR	No	32	((((((((((((((....))))))))))))))
2LUP	RNA	NMR	Yes	32	((((((((((((((....))))))))))))))
5A18	RNA	NMR	No	32	((((((((((..(.....)..)).))))))))
1EXY	RNA	NMR	Yes	33	((((((((((((....))))..)))..))))).
2JXV	RNA	NMR	No	33	((((((((..((((....))))...))))))))
3ID5	RNA	X-RAY	Yes	33	..............(((....)))..........
1ETF	RNA	NMR	Yes	34	((((((..((((((....)))).))...))))))
1P5N	RNA	NMR	No	34	((((....(((((.......))))).....))))
1R2P	RNA	NMR	No	34	(((((((..(((((....)))))....)))))))
1R7W	RNA	NMR	No	34	(((((((......(((((....))))))))))))
1R7Z	RNA	NMR	No	34	(((((((......(((((....))))))))))))
1RNK	RNA	NMR	No	34	[[[[[..((((((.]]]]]........)))))).
1T28	RNA	NMR	No	34	(((((((((................)))))))))
2EUY	RNA	NMR	No	34	((((((..(..((((....))))..)..))))))
2F88	RNA	NMR	No	34	((((((((.(((((....)))))...))))))))
2JTP	RNA	NMR	No	34	(((((((((((...((...))..)))))))))))
2KPV	RNA	NMR	No	34	((((((((.(((((....)))).).)))).))))
2L3C	RNA	NMR	Yes	34	((((.((((((((((....)))))))))).))))
2RVO	RNA	NMR	No	34	((((((((...(((((....))))).))))))))
4OOG	RNA	X-RAY	Yes	34	((((((((((((((....))))))))))))))..
4X4O	RNA	X-RAY	Yes	34	((((((((((((.......)))))))))...)))
6SDY	RNA	NMR	Yes	34	(((((((((((((((....)))))))))))))))
1ULL	RNA	NMR	Yes	35	(((((..(((((((....))))..)))...)))))
2DRB	RNA	X-RAY	Yes	35	((((((((((((.......))))))))))))....

PDB	NA	Method	Complex	Size (nt)	Experimental secondary structure
2L3E	RNA	NMR	No	35	(((((((.....((((((....)))))))))))))
2M57	RNA	NMR	No	35	((..((((..((((.....))))....))))..))
2PCV	RNA	NMR	No	35	(((((......((((....)))).......)))))
4C4W	RNA	X-RAY	Yes	35	(((...(((((..........)))))......)))
5FJ4	RNA	X-RAY	Yes	35	(((...(((((..........)))))......)))
6BHJ	RNA	X-RAY	Yes	35	..((((((((((((.(...).))))))))))))))
1N8X	RNA	X-RAY	No	36	(((((((.(((((((....)))))))...)))))))
2FDT	RNA	NMR	No	36	(((((((.(.(((((.......))))).))))))))
2HW8	RNA	X-RAY	Yes	36	(((((((((..(((....)))......)))))))))
2N6S	RNA	NMR	No	36	((((((((((((((.(....).))))))))))))))
2TPK	RNA	NMR	No	36	..[[[[[.(((((((]]]]].......)))))))..
4X4P	RNA	X-RAY	Yes	36	((((((((((((.......)))))))))...)))..
5KQE	RNA	NMR	No	36	((((((.....(.((((((....)))))))))))))
6SY6	RNA	X-RAY	Yes	36	..((...........(((....)))........)).
2LHP	RNA	NMR	No	37	(((((((((.((((((....))))))..)))))))))
2LUB	RNA	NMR	No	37	(((((((((.((((((....))))))..)))))))))
6DTD	RNA	X-RAY	Yes	37	.((((..(.(((((((.....).)))))).)..))))
6U79	RNA	NMR	No	37	(((((((((((((((.....))).)).))))))))))
1B36	RNA	NMR	No	38	(((((.......((((....)))).........)))))
1M5L	RNA	NMR	No	38	((((....((((.((((....))))...))))..))))
1TXS	RNA	NMR	No	38	((((..((((...(((((....)))))...))))))))
2A9L	RNA	NMR	No	38	((((((((((...((((....))))))))...))))))
2KHY	RNA	NMR	No	38	(.((((.......((((....)))).......)))).)
4PDB	RNA	X-RAY	Yes	38	(((((((((...(((((....)))..)).)))))))))
6D12	RNA	X-RAY	Yes	38	(((((((.....)))))))..(((((.....)))))..
2MXL	RNA	NMR	No	39	..(((((((..((((((......)).))))..)))))))
2NBY	RNA	NMR	No	39	(((((((.((.(.(((((...))))).)))..)))))))
4KR7	RNA	X-RAY	Yes	39	((((((((.......((((....))))))))))))....
4KR9	RNA	X-RAY	Yes	39	((((((((.(....)((((....))))))))))))....
2HUA	RNA	NMR	No	40	(((((((.((...(.((....)).)..))....)))))))
2NBZ	RNA	NMR	No	40	((((((((.(((((((.....)))))))....))))))))
4PMI	RNA	X-RAY	Yes	40	((((((...((..(((((....))).))...)).))))))

PDB	NA	Method	Complex	Size (nt)	Experimental secondary structure
1A51	RNA	NMR	No	41	((((.......((((((((...)))))))).......))))
1ZC5	RNA	NMR	No	41	(((((((((((((((((....)))))))))))...))))))
4M6D	RNA	X-RAY	Yes	41	..(.((.......((((.(.[.(..])..).)))))).)..
5V17	RNA	NMR	No	41	((((((.....(((((((((......)))))))))))))))
5W1H	RNA	X-RAY	Yes	41	.....(((((........))))..)................
6W3M	RNA	NMR	No	41	(((((((((...((((((.....))))))...)))))))))
1MNX	RNA	NMR	No	42	((((.......((((((((....)))))))).......))))
2L2J	RNA	NMR	No	42	((((.(((((((((.((((....)))).))))))))).))))
2N6T	RNA	NMR	No	42	((((...(((((...((((....))))....)))))..))))
5WLH	RNA	X-RAY	Yes	42	......(((((........))))..)................
1CQ5	RNA	NMR	No	43	(((((......(((....(((....)))....)))...)))))
1CQL	RNA	NMR	No	43	((((((.....(((....(((....)))....)))..))))))
2ADT	RNA	NMR	No	43	(((((..((((((((((((....))))))))))))...)))))
2FEY	RNA	NMR	No	43	(((((...(..(((..((((.......)))).))))..)))))
2N6X	RNA	NMR	No	43	((((((((.....(((((((....)))))))....))))))))
1A60	RNA	NMR	No	44	(((((.......)))))[[[...((((((]]]...))))))...
1P6V	RNA	X-RAY	Yes	45	.......(((((((....)))))))...(((((.......)))))
1Z2J	RNA	NMR	No	45	(((((((((((((((((((....)))))))))))...))))))))
1S03	RNA	X-RAY	Yes	47	((((((.((.((..((((((((......)))))).))))))))))))
1YMO	RNA	NMR	No	47	[[[[[[........(((((((((]]]]]]........))))))))).
2MTJ	RNA	NMR	No	47	(((((..((((((..))))))..((((....))))......))))).
2PXL	RNA	X-RAY	Yes	47	..((((((((....(((....(((....)))....))).))))))))
5KH8	RNA	NMR	No	47	[[[[..((((((]]]]......((((....))))....))))))...
2K95	RNA	NMR	No	48	[[[[[[........(((((((((]]]]]]........))).)))))).
2KE6	RNA	NMR	No	48	(((((.(((((((((((((........))))))).))).))).)))))
2KUR	RNA	NMR	No	48	(((((((((((((((((((........))))))).))).)))))))))
2KUU	RNA	NMR	No	48	(((((((((((((((((((........))))))).))).)))))))))
2KUV	RNA	NMR	No	48	(((((((((((((((((((........))))))).))).)))))))))
2KUW	RNA	NMR	No	48	(((((((((((.(((((((........)))))))..)).)))))))))
2M8K	RNA	NMR	No	48	[[[[[......(.((((((((((]]]]].....).).)))))))).).
2VPL	RNA	X-RAY	Yes	48	(((((((((..(((((((......))).))))......))))))))).
4C7O	RNA	X-RAY	Yes	48	((((((((((((((((((((....)))).))).)))).).))))))))

PDB	NA	Method	Complex	Size (nt)	Experimental secondary structure
1U63	RNA	X-RAY	Yes	49	.(((((((((..((((((((....)))).))))......))))))))).
2LU0	RNA	NMR	No	49	(((((..((((.((....))...(((((....)))))))))...)))))
2PXB	RNA	X-RAY	Yes	49	(((.((((((....(((....(((....)))....))).)))))).)))
2PXD	RNA	X-RAY	Yes	49	((((((((((....(((....(((....)))....))).))))))))))
2PXE	RNA	X-RAY	Yes	49	((((((((((....(((....(((....)))....))).))))))))))
2PXF	RNA	X-RAY	Yes	49	((((((((((....(((....(((....)))....))).))))))))))
2PXK	RNA	X-RAY	Yes	49	((((((((((....(((....(((....)))....))).))))))))))
2PXP	RNA	X-RAY	Yes	49	((((((((((....(((....(((....)))....))).))))))))))
2PXQ	RNA	X-RAY	Yes	49	((((((((((....(((....(((....)))....))).))))))))))
2PXT	RNA	X-RAY	Yes	49	((((((((((....(((....(((....)))....))).))))))))))
2PXU	RNA	X-RAY	Yes	49	((((((((((....(((....(((....)))....))).))))))))))
2PXV	RNA	X-RAY	Yes	49	((((((((((....(((....(((....)))....))).))))))))))
6MXQ	RNA	NMR	No	49	((((((.(((...(((.((.(((....)))))..)))...)))))))))
6IV9	RNA	X-RAY	Yes	50	.(((((((((....))))))))..).........................
6IV8	RNA	X-RAY	Yes	51	.(((((((((....))))))))..)..........................
2MHI	RNA	NMR	No	53	(((((((..((((.((((...))))))))(..(((.....))))..)))))))
2N4L	RNA	NMR	No	53	((((((((((((((..(((..((((.....)))))))..))))))))))))))
1P5M	RNA	NMR	No	55	(((((.((((.....(((((..(((.((....)).)))..).)))))))))))))
2HGH	RNA	NMR	Yes	55	((((....((....))(((((((.....((.(....).))....)))))))))))
2KZL	RNA	NMR	No	55	((((((......((((.......((((....)))).......))))...))))))
2LC8	RNA	NMR	No	56	.(((.((((((((.[[[[[[[)))).))))..))).............]]]]]]].
6NOA	RNA	NMR	No	56	((((((.(((...(((.((.(((...........)))))..)))...)))))))))
5IEM	RNA	NMR	No	57	((((((((((.(((((..(((((((....))).)))))))..))...))))))))))
6MCF	RNA	NMR	Yes	57	((((.(((((.(((((..(((((((....))).)))))))..))...))))).))))
4M4O	RNA	X-RAY	Yes	59	((((((.(((((.((.......((((.(.[.(..])..).)))))).))))).))))))
6DB8	RNA	X-RAY	Yes	60	(((((((((..(.....[).(((.....)))(((((......].)))))..)))))))))
1UN6	RNA	X-RAY	Yes	61	((((((....((....))(((((((.....((..(....)..))....)))))))))))))
4U7U	RNA	X-RAY	Yes	61	....((((((....))))))
2N3Q	RNA	NMR	No	62	(((((.(((..(((.(((..)))..)))(((..((((....))))))).....)))))))).
3EGZ	RNA	X-RAY	Yes	65	(((((...((((...........))))((((((((..........))))))))......)))))
2NC1	RNA	NMR	No	67	((((((...(((((((((((....))))))(((((((....)))))))(.....)))))).))))))
5WT1	RNA	X-RAY	Yes	67	((((..((((......[..))))((((((.......))))).)...(((((..]....)))))))))

PDB	NA	Method	Complex	Size (nt)	Experimental secondary structure
2MQT	RNA	NMR	No	68	(((((.((((((((.....((((..((((.((((....))))))))..))))..)))))))).)))))
2N6W	RNA	NMR	No	68	((((((((((((((......(((((...((((....))))....)))))......))))))))))))))
5HR6	RNA	X-RAY	Yes	68	(((((..((((....[....))))((((((.......))))))...(((((..]....))))))))))
6U8D	RNA	X-RAY	Yes	68	.((((((((((..((((((....))))))(((((((....))))))).((((..))))))))))))))
3EPJ	RNA	X-RAY	Yes	69	((((.(..((((....[..))))..((((.......))))......((.((..]....)).))).))))
1KXK	RNA	X-RAY	No	70	.....(((..(((((...(((((((((((((((....)))))..))))))))))..))))))))......
2DET	RNA	X-RAY	Yes	70	..(((....((((....[....))))((((((.......))))))....((((..]....))))..))).
2N8V	RNA	NMR	No	70	(((((.(((((((.[[[[[).)))))).((((((((......))))))))))))).........]]]]].
5HR7	RNA	X-RAY	Yes	70	(((((..((((....[....))))((((((.......))))))...(((((..]....)))))))))))..
5V6X	RNA	X-RAY	Yes	70	(((((((.((((...[.))))(((((((.......)))))))..(((((..]....))))))))))))..
2DU3	RNA	X-RAY	Yes	71	(((((((..(((.....[...)))..((((.......))))......(((((..]....))))))))))))
2DU5	RNA	X-RAY	Yes	71	(((((((..(((.....[...))).(((((.......))))).....(((((..]....))))))))))))
2DU6	RNA	X-RAY	Yes	71	(((((((..(((.....[...))).(((((.......))))).....(((((..]....))))))))))))
2L3J	RNA	NMR	Yes	71	(((((((.(((((((((.(((.(((((((((((.....))))))))))).))).))))))))).)))))))
2MS0	RNA	NMR	Yes	71	(((((....(((.....[...)))((((((.......))))))....(((((..]....)))))..)))))
2ZZN	RNA	X-RAY	Yes	71	(((((((..((((....[..)))).(((((.......))))).....(((((..]....))))))))))))
4YVI	RNA	X-RAY	Yes	71	.((((((..((((....[..))))((((((.......))))))...(((((..]....)))))))))))..
4YVJ	RNA	X-RAY	Yes	71	.((((((..((((....[..))))((((((.......))))))...(((((..]....)))))))))))..
4YVK	RNA	X-RAY	Yes	71	.((((((..((((....[..))))((((((.......))))))...(((((..]....)))))))))))..
5TF6	RNA	X-RAY	Yes	71	.(((..((((..................(((.((((.(((.....)))..)))).)))...))))..)))
1DRZ	RNA	X-RAY	Yes	72	(((((((...[[[[[[((((.[[.....)))))))))))]]....((((..........)))).....]]]]]]
1EUQ	RNA	X-RAY	Yes	72	((((((..(((.....[...))).(((((.......)))))...(((((..]....))))))))))).....
2AKE	RNA	X-RAY	Yes	72	(((((((..((((.......)))).(((((.......))))).....(((((.......)))))))))))).
2MF0	RNA	NMR	Yes	72	((((........))))..((((((......)))))).......((((.....))))..((((.....)))).
2ZNI	RNA	X-RAY	Yes	72	((((.((.((((.....)))).((((.(.......).))))...((((((.......))))))).))))....
4ZT0	RNA	X-RAY	Yes	72	..........((((((..((((....))))....))))))..(((..).)).......((((....))))..
3WC1	RNA	X-RAY	Yes	73	(((((((..(((..........))).(((((.......)))))....(((((.......))))))))))))..
3WFQ	RNA	X-RAY	Yes	73	(((((((.....(.....[..)....(((((.......))))).....(((((..]....)))))))))))).
4X0A	RNA	X-RAY	Yes	73	(((.((..((((.....[..)))).((((.........)))).....(((((..]....))))))).)))...
5VW1	RNA	X-RAY	Yes	73	..........((((((..((((....))))....))))))..(((..).)).......((((....))))....
5WT3	RNA	X-RAY	Yes	73	(((((((..((((......[..))))((((((.......))))).)...(((((..]....))))))))))))
1GTR	RNA	X-RAY	Yes	74	((((((..(((.....[...))).(((((.......))))).....(((((..]....))))))))))).....

PDB	NA	Method	Complex	Size (nt)	Experimental secondary structure
2DER	RNA	X-RAY	Yes	74	....(.(..((((....[....))))((((((.......))))))...(((((..]....)))))).)......
2ZM5	RNA	X-RAY	Yes	74	(((((((..((((.....[..)))).(((((.......))))).....(((((..]....))))))))))))..
3AKZ	RNA	X-RAY	Yes	74	(((((((..((((....[..)))).(((((.......)))))....(((((..]....)))))))))))))....
3FOZ	RNA	X-RAY	Yes	74	(((((((..((((.....[..)))).(((((.......))))).....(((((..]....))))))))))))..
3TUP	RNA	X-RAY	Yes	74	(((((((..((((.....[..)))).(((((.......)))))......((.(..]....).)).)))))))..
3WC2	RNA	X-RAY	Yes	74	(((((((..((((.....[..)))).(((((.......))))).....(((((..]....))))))))))))..
3WFS	RNA	X-RAY	Yes	74	(((((((..((((........)))).(((((.......))))).....(((((.......))))))))))))..
4YCO	RNA	X-RAY	Yes	74	(((((((..((((.....[..)))).(((((.......))))).....(((((..]....))))))))))))..
4YYE	RNA	X-RAY	Yes	74	(((((((..((((.....[..)))).((((.........)))).....(((((..]....))))))))))))..
5D6G	RNA	X-RAY	Yes	74	(((.......(((((.(((..(((.........)))..)))..........((......))...)))))...)))
1FFY	RNA	X-RAY	Yes	75	(((((((..((((.....[...)))).(((((.......))))).....(((((..]....))))))))))))..
1N77	RNA	NMR	Yes	75	(((((((..((((....[...)))).(((((.......)))))....(((((..]....))))))))))))....
2DR2	RNA	X-RAY	Yes	75	(((((((....((.......))...(((((.......))))).....(((((.......))))))))))))....
2IHX	RNA	NMR	Yes	75	((((((((...((((.(((((.......)))))))))....((((.....))))(((....)))...))))))))
2ZUE	RNA	X-RAY	Yes	75	(((((((..((((......[...)))).(((((.......))))).....(((((..]....))))))))))))
3WQY	RNA	X-RAY	Yes	75	(((((((..((((....[..)))).((((((.....)))))).....(((((..]....))))))))))))....
3WQZ	RNA	X-RAY	Yes	75	(((((((..((((....[..)))).(((((((...))))))).....(((((..]....))))))))))))....
4TZV	RNA	X-RAY	Yes	75	(((..((((....[..)))).(((((.......))))).....(((((..]....))))))))((((....))))
4WC2	RNA	X-RAY	Yes	75	((((.((..((((.....[..)))).(((((.......))))).....(((((..]....))))))).))))...
5X6B	RNA	X-RAY	Yes	75	(((((((....((....[..))...(((((.......))))).....(((((..]....))))))))))))....
1EIY	RNA	X-RAY	Yes	76	(((((((..((((.....[..)))).(((((.......))))).....(((((..]....))))))))))))....
2K4C	RNA	NMR	No	76	(((((((..((((.....[..)))).(((((.......))))).....(((((..]....))))))))))))....
4WC3	RNA	X-RAY	Yes	76	((((.((..((((.....[..)))).(((((.......))))).....(((((..]....))))))).))))....
4WJ3	RNA	X-RAY	Yes	76	(((((((..(((......[...))).(((((.......))))).....(((((..]....))))))))))))....
1P5P	RNA	NMR	No	77	(((((.((((.....(((((..(((.((....(((((.......))))).....)).)))..).)))))))))))))
3A2K	RNA	X-RAY	Yes	77	(((((((..((((.....[...)))).(((((.......))))).....(((((..]....))))))))))))....
4X0B	RNA	X-RAY	Yes	77	.((((.((..((((........))))...((.........)).......(((((.......))))))).))))....
5CCB	RNA	X-RAY	Yes	77	((((((((..((((........)))).(((((.(...).))))).....(((((.......))))))))))))..)
3AMT	RNA	X-RAY	Yes	78	(((((((..((((......[...))))(((((.........)))))....(((((..]....))))))))))))....
3U4M	RNA	X-RAY	Yes	80	((((.(......[..(((((..(((......((((((((....))))))))...)))..........].)))))).))))
6B14	RNA	X-RAY	Yes	83	((((((((((..((((.(..[[.(((.(((((((((.....)))))))..))]..].).).)..).))))..).)))))))))
6B3K	RNA	X-RAY	Yes	83	((((((((((..((((.(..[[.(((.(((((((((.....)))))))..))]..].).).)..).))))..).)))))))))

PDB	NA	Method	Complex	Size (nt)	Experimental secondary structure
2ZZM	RNA	X-RAY	Yes	84	....(((..(((.......[.....)))((((((.......))))))(((....)))...(.(((..]....))).))))....
3A3A	RNA	X-RAY	No	86	(((((((.(..((((((..[.))))))((((((.......)))))).(((((....))))).((((..]....))))).)))))))
3K0J	RNA	X-RAY	Yes	87	((((((((((..(((.(((..........))))))).........)))).....(((...((((......))))...)))..))))))
1WZ2	RNA	X-RAY	Yes	88	(((((((..(((.......[.....))).(((((.......))))).(((....)))...(((((..]....))))))))))))....
5XBL	RNA	X-RAY	Yes	88	..........(((((...(((......))).....)))))..(.(..)..).......((((....)))).....(.....)......
2N7M	RNA	NMR	No	92	(((((((((((.(((((..(((((.(((.....(((....)))..))))))))...(((((((....))))))).))))).)))))))))))
3ADB	RNA	X-RAY	Yes	92	(((((((((..((((((..[.))))))((((((.......))))))(((((((....)))))))((((..]....)))))))))))))....
3W1K	RNA	X-RAY	Yes	92	(((((((...((((((..[.))))))((((((.......)))).))(((((((.....))))))).(((((..]....))))).))))))).
2V3C	RNA	X-RAY	Yes	96	((((..(((((.(((((((((....)))))))))..))))).....(((((.....(((.....(((....))).....)))..)))))..)))).
3KTW	RNA	X-RAY	Yes	96	.(((...((((((((((((((((....)))))))))))).))))).....((((.....(((.....(((....))).....)))..))))...)))
1LNG	RNA	X-RAY	Yes	97	..(.((..(((((.(((((((((....)))))))))..)))))....((((((.....(((.....(((....))).....)))..)))))).)).)
6JXM	RNA	X-RAY	No	97	(((((((..((((....[..)))).((((((((((...(((......)))..)..)))))))))).....(((((..]....))))))))))))....
3W3S	RNA	X-RAY	Yes	98	((((((((..((.((((..[.))))))((((((.......))))).)((((.((((....)))).)))).(((((..]....))))))))))))...
1S9S	RNA	NMR	No	101	.((((..((((((....))))))..)))).....((((..(((.(((((((((....)))))....)))))))))))((((((((((....))))))))))
6MJ0	RNA	X-RAY	No	101	[[[....((((]]]....))))...[((((((..[...)))))((((((....))))))...((((..]....))))[[[.....(((.]]].]..)))...
2KRL	RNA	NMR	No	102	.((((((((....))))))).......(((((((((..[[[[...)))))))))...((((.((((((......(........)......))))).))))]]]]
2XXA	RNA	X-RAY	Yes	102	((((((((((.((((((((.(((((((((((.....((((....(((....)))....))))...))).)).))))))..)).))))))...))))))))))
7K1Z	RNA	NMR	No	103	(((((((((..(((((((((((.(.(((.......))).).).))...)))))))).(.(..((((((.....))).((....))))..).)...)))))))))

PDB	NA	Method	Complex	Size (nt)	Experimental secondary structure
2NBX	RNA	NMR	No	108	((((((...((((((((((.((.(.(((((...))))).)))..))))))(((((((.(((((((.....)))))))....)))))))(.....)))))).))))<br>))
2LKR	RNA	NMR	No	111	(((((((.....(((((((.((((.(((.....)))..)))).......((((((((((((....)))))))))))).......)))..))))).......)))).)<br>)))
4P3E	RNA	X-RAY	Yes	124	(.((..(.((((((((((.(((((..((((((....))))))..))))).)))).......(.(((((.....((((....(((....)))....))))...))))))<br>))))))).)...)).)
3IVK	RNA	X-RAY	Yes	128	((((((((...[[[[[[.)))))))).............[[[[[(...).(.((((((((((((.....)))))..((((.]]]]]))))((.((((......)))<br>).))))))))))).]]]]].
3NDB	RNA	X-RAY	Yes	136	((((((..(((((.(((.(((((..(((((.(((((((((....)))))))))..)))))....((((((.....(((.....(((....))).....)))..)))))<br>).))))))).).)))))......))))))
2N1Q	RNA	NMR	No	155	((((((((((.(((((((.(((((((....)))))))...(((..(((.(((....((((.(((((((....)))))))...))))..))).)))..)))...)))<br>)).)......(((..(((((....)))))..))).).)))))))))))).
2R8S	RNA	X-RAY	Yes	159	.....((((.(...((((((.....(((.((((.(((..(((((((((....)))))))))..((.......))....)))......)))))))....))))))..).)<br>)))((...((((...((((((((.......))))))))..))))...))...
4P8Z	RNA	X-RAY	No	188	(((((..(((((..(((((((((...[[[.))))))))))..((((.(((.((((......(((((...((((..(((...]]])))).......))))((....))...<br>..[[[[[...))))).(((.....)))))))).)))....]]]]].((((....))))....))))...)))))..)))))
1GRZ	RNA	X-RAY	No	247	(((((.((...((((((...((((((.....(((.((((.(((...((((((((....))))))))...((.......))....)))......)))))))....)))))<br>)..)).))))).(...((((..((((((((.(....).)))))))).)))))...)....[.[.[[[..)).))))).(.((((((.....)))))).)........]]].]]((<br>.(.((((((....))))))..)......)).

PDB	NA	Method	Complex	Size (nt)	Experimental secondary structure
5IWA	RNA	X-RAY	Yes	1509	........((((..[.[[[..)))).((((.(((((..(((((((((....(((.(((..(((..((.((((((((((.....)))))))))).)))))......(((<br>......(((((((..((...((((((.(((((....((((((....))))))......)))))....((((.(((((....))))).))))...((((...)))).)<br>))))))..))))))))))(((....(((..((((((((.......)))))))))))......)))..((((((((....))))...))))))).(((((.........<br>...))))).((((....))))...))))))).)......(.(((...(((((....)))).))))).)).))))))..((((......((((....)))).....))))..<br>..(((((...(....((((.....))))......)....)))))......((((([[[...(((((.....((.]]]))........))))))))))..))))))))).....<br>......((([[...(.((((...(((.((((((((.((((((((((......((((((.....))))))....))))))))..)))))))))..((((((((...((<br>((((((....((((((....((.........))........))))))....)........((....)).))))))))..))))).)))..))))...))))....((((((...<br>((...((((.........))))...))))))))))......{...((((((..((((((((((...))))))))))...((..]]))......))))))))))).(((.....<br>.((((....))))....)))...]]].]((((((.(((((((.((..(((((..((((((((((......((.........))..........(((((((..(...(((((<br>(((..(((((((....)))))))...((((....))))..))))))))).((.(((...((((((.(....(((((((((....)))...(((......)))...)))<br>)))......((((.(((((((..(((..(((.....))))))...)))))))..(..(((((....)))))......)..)))).....).).)))...)).)))))....<br>))))))))..[)).)))))))))).(...(((((((.....(((..(((.((((....))))).))....)))......)))))))......(....(((((((........)<br>))))))....)..)..)))))......(((((((......]...)))))))......))...)))))))))).))..(.(..((...((((.(((...((((((((((((<br>...(.((((....(((....))).)))).)..)))))))))))...))).))))....))...)..)..((((((((((....)))))))))}...
