# Supplementary material for: Comparative Study of Single-stranded Oligonucleotides Secondary Structure Prediction Tools
Source: BMC Bioinformatics. 2023 Nov 8;24:422. doi: 10.1186/s12859-023-05532-5 (PMC10634105; doi:10.1186/s12859-023-05532-5)
Supplement: Supplementary file 3 — Additional file 3. Comparison between predicted and experimental secondary structure using the AptaMat distance as a metric for CONTRAfold, CentroidFold, Linearfold, MC-fold, MXfold2, UFold, and SPOT-RNA. The PDB code is reported in the first column. $Apta_D$AptaD values are reported for each PDB and software prediction. "/" characters indicate either structures predicted as unfolded or software failure during the computation. [file 12859_2023_5532_MOESM3_ESM.pdf]

Additional File 3. Comparison between predicted and experimental secondary structure using the AptaMat distance as a metric for CONTRAfold, CentroidFold, Linearfold, MC-fold, MXfold2, Ufold and SPOT-RNA. The PDB code is reported in the first column. AptaD values are reported for each PDB and software prediction. "/" characters indicate either structures predicted as unfolded or software failure during the computation.

PDB	CONTRAfold	CentroidFold	Linearfold-C	Linearfold-V	MC-fold pseudoknot	MC-fold	MXfold2	Ufold	SPOT-RNA
1PQT	/	/	/	/	/	/	0.00	/	0.00
2K71	0.00	0.00	/	/	0.40	0.40	0.00	0.00	0.40
5GWL	/	/	/	/	/	/	4.00	/	/
5GWQ	/	/	/	/	/	/	4.00	/	/
6J37	/	/	/	/	/	/	4.00	3.67	/
6M0B	/	/	/	/	4.00	4.00	4.00	4.00	/
6M0C	/	/	/	/	4.00	4.00	4.00	/	/
5OND	/	/	/	/	0.80	0.80	0.50	2.33	1.00
1ZHU	/	/	/	/	1.33	1.33	3.67	1.67	/
2A0I	/	/	/	/	/	/	3.33	0.60	1.25
2LO8	/	/	/	/	0.00	0.00	0.60	1.00	1.33
3WPD	1.00	1.00	/	/	1.33	1.33	1.00	/	/
6IY5	/	/	/	/	/	/	3.67	0	0
1BJH	0.00	/	/	/	0.00	0.00	0.00	1	1
3WPG	1.00	/	/	/	1.33	1.33	1.00	/	/
2LO5	0.43	0.43	/	/	0.00	0.00	0.43	0.43	0.00
3WPH	1.00	/	/	/	1.33	1.33	1.00	1.00	0.50
6FKE	0.00	0.29	/	/	0.00	0.00	0.00	0.00	0.00
1LA8	0.00	0.22	0.00	0.00	0.00	0.00	0.00	0.00	0.00
1P0U	0.18	0.00	/	/	0.00	0.00	0.00	0.00	0.00
2EXF	1.29	1.00	/	/	0.63	0.63	1.29	1.29	0.43
2JZW	1.29	1.00	/	/	0.63	0.63	1.29	1.29	0.43
5F55	/	/	/	/	5.67	5.67	6.67	/	/
6FK5	0.00	0.00	0.00	0.00	0.00	0.00	0.00	0.00	0.00
1UUT	0.00	0.00	0.00	0.00	0.00	0.00	0.00	0.00	0.00
2M8Y	0.00	0.18	0.00	0.00	0.00	0.00	0.00	0.00	0.00
1AC7	0.00	0.18	0.00	0.00	0.15	0.15	0.00	0.00	0.00
6FK4	0.00	0.00	0.00	0.00	0.18	0.18	0.00	0.50	0.18
1XUE	/	/	/	/	0.73	0.73	3.00	9.00	0.22
1EN1	0.00	/	0.75	0.75	1.82	1.82	0.75	0.75	0.00
4KB0	0.33	0.33	0.33	0.33	0.33	0.33	0.33	0.33	0.33
4KB1	0.33	0.33	0.33	0.33	0.33	0.33	0.33	0.33	0.33
1ECU	0.12	0.00	0.13	0.13	0.00	0.00	0.00	0.00	0.12
3Q0A	0.00	0.00	0.00	0.00	0.00	0.00	0.00	0.00	0.00
4FF1	0.00	0.00	0.00	0.00	6.50	6.50	0.00	0.00	1.55
3C46	0.18	0.00	0.18	0.18	0.18	0.18	0.18	0.18	0.18
3Q23	0.18	0.00	0.18	0.18	0.18	0.18	0.18	0.18	0.18
2A6O	0.00	0.00	0.00	0.00	0.12	0.12	0.00	0.12	0.12
3Q24	0.18	0.00	0.18	0.18	0.18	0.18	0.18	0.18	0.18
2L5K	0.00	0.00	/	/	0.47	0.47	0.00	0.00	0.13
3DSD	0.00	0.00	0.00	0.00	0.13	0.13	0.00	0.00	0.00
2VHG	0.00	0.00	0.00	0.00	0.12	0.12	0.00	0.12	0.12
1OSB	0.50	0.18	0.50	0.50	0.62	0.62	0.50	0.50	0.62
1ZM5	0.50	0.18	0.50	0.50	0.62	0.62	0.50	0.50	0.62
2CDM	2.50	2.55	2.50	2.50	1.13	1.13	2.50	2.50	2.46
2VIC	0.12	0.13	0.00	0.00	0.12	0.12	0.12	0.12	0.12
5N2Q	0.00	0.15	0.00	0.00	0.13	0.13	0.00	0.00	0.13
1JVE	0.00	0.00	0.00	0.00	0.00	0.00	0.00	0.00	0.00
1NGO	0.00	0.09	0.00	0.00	0.22	0.22	0.00	0.00	0.00
1NGU	0.00	0.00	0.00	0.00	0.29	0.29	0.00	0.00	0.20
3ZH2	2.00	2.00	2.00	2.00	2.44	2.44	2.00	1.46	2.00
4HT4	0.00	0.00	0.00	0.00	10.11	10.11	0.00	2.13	0.00
1YTB	0.00	0.00	0.00	0.00	0.00	0.00	0.00	0.00	0.00
1B4Y	0.00	/	0.00	0.00	1.29	1.29	6.00	0.15	0.15
4ER8	0.11	0.22	0.11	0.11	0.18	0.18	0.11	0.00	0.09
4F41	0.07	0.07	0.07	0.07	0.00	0.00	0.07	0.00	0.00
4F43	0.00	0.07	0.07	0.07	0.00	0.00	0.07	0.00	0.00
5HRU	4.11	5.00	4.11	4.11	5.12	6.04	1.20	1.30	1.24

PDB	CONTRAfold	CentroidFold	Linearfold-C	Linearfold-V	MC-fold pseudoknot	MC-fold	MXfold2	Ufold	SPOT-RNA
6SEI	0.00	0.86	2.42	2.42	0.08	0.08	0.00	0.00	0.08
5HTO	2.19	5.82	5.82	5.82	6.81	6.81	1.09	1.09	1.52
2VJU	1.05	1.22	1.05	1.05	1.04	1.04	1.05	0.86	1.05
1EZN	0.29	6.57	2.81	2.81	0.00	0.00	0.14	3.18	3.38
1SNJ	4.14	3.22	4.16	4.16	0.00	0.00	0.14	3.07	2.62
6U82	0.00	0.00	0.00	0.00	0.00	0.00	0.00	0.00	0.00
3HXO	6.08	0.70	11.79	11.79	6.21	0.46	9.96	4.27	0.13
2N8A	0.05	0.11	0.05	0.05	0.05	0.05	0.05	0.00	0.05
3THW	0.00	0.00	0.00	0.00	0.00	0.00	0.00	0.00	0.00
2IXZ	/	/	/	/	/	/	0.50	0.00	1.00
2OJ7	/	/	/	/	0.40	0.40	0.00	0.00	0.00
1IDV	0.75	0.00	0.00	0.00	0.29	0.29	0.00	0.29	0.29
1R4H	0.67	0.67	0.67	0.67	0.29	0.29	0.67	0.29	0.29
2MXJ	/	/	/	/	1.00	1.00	0.00	0.00	0.40
1AFX	0.00	0.00	0.00	0.00	0.22	0.22	0.00	0.00	0.22
1RNG	0.22	0.50	/	/	0.00	0.00	0.22	0.00	0.00
1ZIF	0.00	0.29	0.00	0.00	0.22	0.22	0.00	0.00	0.00
1ZIG	0.00	0.29	0.00	0.00	0.22	0.22	0.00	0.00	0.00
1ZIH	0.00	0.29	0.00	0.00	0.22	0.22	0.00	0.00	0.00
2F87	0.00	0.00	0.00	0.00	0.00	0.00	0.00	0.00	0.00
5FMZ	/	/	/	/	3.00	3.00	0.00	0.67	/
1ESH	0.00	0.00	0.00	0.00	0.00	0.00	0.00	0.00	0.00
1HS1	0.00	0.00	/	/	0.29	0.29	0.00	0.00	0.29
1HS2	0.00	0.00	/	/	0.75	0.75	0.00	0.00	0.00
1HS3	0.00	0.00	/	/	0.29	0.29	0.00	0.00	0.29
1HS4	0.00	0.00	/	/	0.75	0.75	0.00	0.57	0.00
1HS8	0.00	0.00	/	/	0.29	0.29	0.00	0.00	0.00
1I46	0.00	0.00	0.00	0.00	0.00	0.00	0.00	0.00	0.00
1I4B	0.00	0.00	0.00	0.00	0.00	0.00	0.00	0.18	0.18
1JZC	0.00	0.00	0.00	0.00	0.00	0.00	0.00	0.00	0.00
1VOP	0.22	0.00	/	/	0.22	0.22	0.22	0.22	0.00
4Z0C	/	/	/	/	1.60	1.60	0.67	/	/
6FQ3	0.00	0.22	0.22	0.22	0.00	0.00	0.00	0.00	0.00
6FQL	0.00	0.50	/	/	0.00	0.00	0.00	0.00	0.18
1F85	0.00	0.00	0.00	0.00	0.18	0.18	0.00	0.00	0.00
1FHK	0.29	0.29	/	/	1.33	1.33	0.29	0.29	0.29
1IK1	0.00	0.00	0.00	0.00	0.00	0.00	0.00	0.00	0.00
1K4A	0.00	0.00	0.00	0.00	0.00	0.00	0.00	0.00	0.18
1K4B	0.50	0.00	0.00	0.00	0.18	0.18	0.00	0.50	0.18
1ROQ	0.00	0.00	0.00	0.00	0.18	0.18	0.00	0.18	0.18
2EVY	0.22	0.22	0.22	0.22	0.60	0.60	0.22	0.22	0.22
2KOC	0.00	0.00	0.00	0.00	0.18	0.18	0.00	0.18	0.18
2Y95	0.00	0.00	0.00	0.00	0.00	0.00	0.00	0.00	0.00
4Z7L	/	/	/	/	0.40	0.40	0.00	/	0.00
1A4T	0.00	0.00	0.00	0.00	0.18	0.18	0.00	0.22	0.00
1ATW	0.00	0.00	0.00	0.00	0.18	0.18	0.00	0.18	0.18
1OQ0	0.00	0.18	0.18	0.18	0.00	0.00	0.00	0.00	0.18
1Q75	0.00	0.00	0.00	0.00	0.18	0.18	0.00	0.27	0.00
1QFQ	0.00	0.20	0.00	0.00	0.18	0.18	0.00	0.20	0.00
1XWP	0.00	0.00	0.00	0.00	0.33	0.33	0.00	0.00	0.00
2LPA	0.00	0.00	0.00	0.00	0.00	0.00	0.00	0.00	0.00
4AL7	0.00	0.00	0.00	0.00	0.18	0.18	0.00	0.27	0.00
1JTW	0.00	0.00	0.00	0.00	0.33	0.33	0.00	0.00	0.00
1JWC	0.00	0.00	0.00	0.00	0.00	0.00	0.00	0.00	0.00
1XWU	0.44	0.75	/	/	0.18	0.18	0.44	0.00	0.18
2L6I	0.27	0.27	0.27	0.27	0.00	0.00	0.27	0.00	0.27
2LP9	0.27	0.27	/	/	0.00	0.00	0.27	0.00	0.17
2MNC	0.33	0.33	0.33	0.33	0.18	0.18	0.18	0.40	0.33
4AL5	0.00	0.00	0.00	0.00	0.18	0.18	0.00	0.00	0.00
4ILM	/	/	/	/	5.00	5.00	5.00	/	3.00
4QIL	0.00	0.00	0.00	0.00	0.00	0.00	0.00	0.00	0.00
1ATV	0.00	0.00	0.00	0.00	0.00	0.00	0.00	0.00	0.00
1BZ2	0.00	0.22	0.00	0.00	0.67	0.67	0.00	0.36	0.18
1BZ3	0.36	0.60	0.36	0.36	0.23	0.23	0.36	0.00	0.33

PDB	CONTRAfold	CentroidFold	Linearfold-C	Linearfold-V	MC-fold pseudoknot	MC-fold	MXfold2	Ufold	SPOT-RNA
1KKA	0.15	0.17	0.15	0.15	0.15	0.15	0.15	0.23	0.15
1WKS	0.00	0.00	0.00	0.00	0.15	0.15	0.00	0.00	0.00
1YN1	0.00	0.00	0.00	0.00	0.15	0.15	0.00	0.00	0.00
2JR4	0.00	0.00	0.00	0.00	1.00	1.00	0.00	0.00	0.18
2KPC	0.00	0.18	0.00	0.00	0.00	0.00	0.00	0.00	0.00
2KPD	0.00	0.22	0.00	0.00	0.18	0.18	0.00	0.00	0.00
2KRP	0.00	0.00	0.00	0.00	0.36	0.36	0.00	0.00	0.18
2KVN	0.00	0.00	0.00	0.00	0.50	0.50	0.00	0.36	0.00
2LAC	0.00	0.00	0.00	0.00	0.67	0.67	0.00	0.36	0.18
2LBJ	0.00	0.15	0.15	0.15	0.00	0.00	0.00	0.00	0.00
2LBK	0.00	0.00	0.00	0.00	0.15	0.15	0.00	0.00	0.00
2LBL	0.00	0.00	0.00	0.00	0.67	0.67	0.00	0.00	0.18
2M4W	0.40	0.40	/	/	0.55	0.55	0.40	1.40	0.40
4ZLD	0.15	0.00	/	/	0.15	0.15	0.15	0.15	0.15
6CYT	0.60	/	/	/	1.89	1.89	6.57	/	0.83
1Z30	0.00	0.00	0.00	0.00	0.13	0.13	0.00	0.00	0.00
2GVO	0.00	0.00	0.00	0.00	0.43	0.43	0.00	0.00	0.00
2QH4	0.00	0.00	0.00	0.00	0.43	0.43	0.00	0.00	0.00
2Y9H	0.38	0.15	0.00	0.00	0.13	0.13	0.00	0.00	0.00
1ATO	0.00	0.00	0.00	0.00	0.43	0.43	0.00	0.00	0.00
1ESY	0.15	0.15	0.15	0.15	0.15	0.15	0.15	0.15	0.15
1I3X	0.00	0.00	0.00	0.00	0.13	0.13	0.00	0.00	0.00
1SLP	0.00	0.91	0.67	0.67	0.13	0.13	0.00	0.00	0.00
1UUU	0.29	0.29	0.29	0.29	0.29	0.29	0.29	0.29	0.29
2B6G	0.20	0.20	0.20	0.20	0.13	0.13	0.20	0.20	0.20
2B7G	0.00	0.00	0.00	0.00	0.13	0.13	0.00	0.00	0.00
2MEQ	0.00	0.00	0.00	0.00	0.43	0.43	0.00	0.00	0.23
2MFD	0.00	0.00	0.00	0.00	0.13	0.13	0.00	0.00	0.00
2RLU	0.38	0.00	0.00	0.00	0.13	0.13	0.13	0.13	0.00
2Y8Y	0.53	0.00	0.15	0.15	0.29	0.29	0.15	0.15	0.15
4QI2	0.15	7.00	/	/	0.43	0.43	0.15	0.15	0.15
5N5C	0.29	0.18	0.15	0.15	0.29	0.29	0.29	0.29	0.29
6TQB	0.00	0.13	0.00	0.00	0.00	0.00	0.00	0.00	0.13
1A1T	0.00	0.00	0.00	0.00	0.00	0.00	0.00	0.00	0.00
1HLX	0.00	0.00	0.00	0.00	0.12	0.12	0.00	0.12	0.12
1MFJ	0.00	0.00	0.00	0.00	0.13	0.13	0.00	0.13	0.00
1U2A	0.00	0.00	0.00	0.00	0.13	0.13	0.00	0.00	0.20
2JPP	0.13	0.13	0.13	0.13	0.38	0.38	0.13	0.13	0.13
2O33	0.00	0.00	0.00	0.00	0.29	0.29	0.00	0.00	0.40
2RPK	0.00	0.00	0.00	0.00	0.20	0.20	0.00	0.00	0.00
2RPT	0.00	0.00	0.00	0.00	0.25	0.25	0.00	0.00	0.25
2Y8W	0.53	0.00	0.15	0.15	0.29	0.29	0.15	0.15	0.15
4L8H	0.00	0.13	0.00	0.00	0.00	0.00	0.00	0.00	0.12
5F5F	0.18	1.11	/	/	0.53	0.53	0.00	0.00	0.29
6PK9	0.00	0.00	0.00	0.00	0.38	0.38	0.00	0.00	0.00
17RA	0.13	0.00	0.13	0.13	0.13	0.13	0.13	0.13	0.13
1D0U	0.00	0.00	0.00	0.00	0.00	0.00	0.00	0.00	0.12
1JOX	0.00	0.00	0.00	0.00	0.38	0.38	0.00	0.00	0.00
1QWA	0.00	0.57	0.57	0.57	0.12	0.12	0.00	0.12	0.13
1RKJ	0.13	0.23	0.23	0.23	0.38	0.38	0.13	0.38	0.29
1SZY	0.00	0.00	0.00	0.00	0.38	0.38	0.00	0.00	0.00
2FY1	0.00	0.00	0.00	0.00	0.12	0.12	0.00	0.00	0.00
2M21	0.00	0.00	0.00	0.00	0.38	0.38	0.00	0.00	0.00
2MFF	0.00	0.00	0.00	0.00	0.12	0.12	0.00	0.00	0.00
2MFG	0.00	0.00	0.00	0.00	0.18	0.18	0.00	0.00	0.00
5F5H	0.00	0.00	0.00	0.00	0.38	0.38	0.00	0.00	0.00
5ID6	0.00	0.22	0.00	0.00	2.43	2.43	0.00	0.00	0.18
5L1Z	0.67	0.67	0.67	0.67	3.91	3.91	2.50	/	1.63
6XWJ	0.00	0.12	0.00	0.00	0.00	0.00	0.00	0.00	0.00
1F9L	0.00	0.00	0.00	0.00	0.35	0.35	0.00	0.00	0.13
1FJE	0.00	0.00	0.00	0.00	2.69	2.69	0.00	0.44	1.20
1IKD	0.00	0.00	0.00	0.00	0.13	0.13	0.00	0.13	0.00
1JUR	0.00	0.00	0.00	0.00	0.12	0.12	0.00	0.00	0.12
1K2G	4.78	0.27	/	/	0.43	0.43	0.27	0.54	4.00

PDB	CONTRAfold	CentroidFold	Linearfold-C	Linearfold-V	MC-fold pseudoknot	MC-fold	MXfold2	Ufold	SPOT-RNA
1K6G	0.00	0.00	0.00	0.00	0.00	0.00	0.00	0.00	0.00
1K6H	0.00	0.00	0.00	0.00	0.00	0.00	0.00	0.00	0.00
1N66	0.00	0.00	0.00	0.00	0.35	0.35	0.00	0.00	0.25
1OSW	0.00	0.00	0.00	0.00	0.25	0.25	0.00	0.13	0.13
1PJY	0.00	0.00	0.00	0.00	0.00	0.00	0.12	0.00	0.00
1TJZ	0.00	4.36	0.00	0.00	0.35	0.35	0.00	0.00	0.25
2G1W	3.55	4.10	3.55	3.55	0.13	5.53	3.55	0.13	3.55
2GRW	0.00	0.00	0.00	0.00	0.22	0.22	0.00	0.00	0.22
2GV3	0.13	0.20	0.20	0.20	0.33	0.33	0.20	0.13	0.20
2GV4	0.00	0.00	0.00	0.00	0.22	0.22	0.00	0.00	0.22
2HNS	0.00	0.00	0.00	0.00	0.11	0.11	0.00	0.11	0.11
2JSE	0.00	0.00	0.00	0.00	0.35	0.35	0.00	0.20	0.13
2JYM	0.18	0.18	0.18	0.18	0.00	0.00	0.18	0.00	0.00
2K66	0.00	0.00	0.00	0.00	0.11	0.11	0.00	0.00	0.00
2KD8	0.00	0.38	0.00	0.00	0.11	0.11	0.00	0.00	0.12
2M5U	0.00	0.00	0.00	0.00	0.11	0.11	0.00	0.00	0.00
2MFC	0.00	0.00	0.00	0.00	0.71	0.71	0.00	0.00	0.00
2MFE	0.00	0.00	0.00	0.00	0.24	0.24	0.00	0.24	0.00
2W2H	0.71	0.71	0.71	0.71	0.71	0.71	0.71	0.71	0.62
4A4S	0.00	0.00	0.00	0.00	0.00	0.00	0.00	0.00	0.00
6F4H	0.00	0.00	0.00	0.00	1.25	1.25	0.00	0.00	0.00
6KYV	0.00	0.00	0.00	0.00	0.11	0.11	0.00	0.11	0.11
1BGZ	1.44	0.47	1.85	1.85	0.56	0.56	0.38	0.47	0.47
1BVJ	0.00	0.13	0.00	0.00	0.33	0.33	0.00	0.00	0.13
1JTJ	0.33	0.00	0.00	0.00	0.82	0.82	0.00	0.75	0.75
1K5I	0.00	0.00	0.00	0.00	0.11	0.11	0.00	0.00	0.00
1MFK	0.30	0.12	0.00	0.00	0.11	0.11	0.00	0.30	0.11
1OW9	0.00	0.00	0.00	0.00	0.35	0.35	0.00	0.00	0.13
1S2F	0.00	1.00	1.00	1.00	0.11	0.11	0.00	0.00	0.12
1TLR	0.00	0.00	2.45	2.45	0.41	0.41	0.00	0.13	0.13
2ANN	2.00	2.00	/	/	4.31	4.31	2.00	2.00	2.00
2ES5	0.16	0.16	0.16	0.16	0.10	0.10	0.16	0.00	0.16
2M12	0.38	0.43	0.31	0.31	0.65	0.65	0.31	0.38	0.38
2M22	0.00	0.00	0.00	0.00	0.22	0.22	0.12	0.12	0.22
2N0R	1.22	1.22	1.22	1.22	1.36	1.36	0.00	0.60	1.18
2N2O	0.15	7.00	/	/	1.00	1.00	0.93	0.93	0.15
2N2P	0.15	0.00	0.00	0.00	1.00	1.00	0.93	0.93	0.15
2N3O	0.00	0.00	0.00	0.00	0.11	0.11	0.00	0.00	0.00
2N7X	0.57	1.17	1.17	1.17	0.35	0.35	1.17	0.13	1.17
2N82	1.09	0.22	0.22	0.22	2.29	2.29	0.22	2.17	0.22
2PJP	0.19	0.22	0.21	0.21	0.10	0.10	0.21	0.19	0.10
2QH3	0.00	0.00	0.00	0.00	0.33	0.33	0.00	0.24	0.00
2RO2	0.11	0.12	0.12	0.12	0.11	0.11	0.00	0.11	0.00
2UWM	0.25	0.28	0.28	0.28	0.21	0.21	0.21	0.25	0.28
3PHP	0.00	0.00	0.00	0.00	0.33	0.33	0.00	0.56	0.18
5UF3	0.00	0.00	0.11	0.11	0.11	0.11	0.00	0.00	0.11
5WQ1	0.00	0.00	0.00	0.00	0.11	0.11	0.00	0.00	0.00
6GBM	0.20	0.20	0.20	0.20	0.33	0.33	0.20	0.00	0.20
1A9N	3.00	0.00	0.00	0.00	3.07	3.07	0.00	2.25	1.62
1E4P	0.00	0.00	0.00	0.00	0.56	0.56	0.00	0.00	0.13
1KKS	0.13	0.46	0.13	0.13	0.35	0.35	0.13	0.13	0.43
1MT4	0.00	0.24	0.00	0.00	0.63	0.63	0.00	0.24	0.00
1NC0	0.12	0.12	0.12	0.12	0.33	0.33	0.12	0.12	0.12
1NYB	0.00	0.12	0.00	0.00	0.11	0.11	0.00	0.00	0.00
1RHT	0.13	0.77	0.13	0.13	0.25	0.25	0.29	0.13	0.13
1SYZ	0.00	0.00	0.00	0.00	0.22	0.22	0.00	0.00	0.00
1TFN	0.13	0.77	0.13	0.13	0.38	0.38	0.29	0.13	0.13
2HEM	0.00	0.00	0.00	0.00	0.32	0.32	0.00	0.12	0.32
2LK3	0.00	0.00	0.00	0.00	0.11	0.11	0.00	0.00	0.11
2LV0	0.00	0.47	0.00	0.00	0.63	0.63	0.00	0.56	0.29
2QH2	0.18	0.18	0.18	0.18	0.30	0.30	0.18	0.16	0.18
3NVK	3.25	3.25	/	/	3.55	3.55	1.83	1.50	2.33
5F9F	0.00	0.00	0.00	0.00	0.10	0.10	0.00	0.00	0.00
5NG6	0.00	0.00	0.00	0.00	2.29	2.29	2.00	0.36	2.00

PDB	CONTRAfold	CentroidFold	Linearfold-C	Linearfold-V	MC-fold pseudoknot	MC-fold	MXfold2	Ufold	SPOT-RNA
5UDZ	0.15	0.42	0.00	0.00	3.50	0.43	0.15	0.15	0.00
1M82	0.11	0.00	0.11	0.11	0.20	0.20	0.11	0.20	0.20
1QC8	0.22	0.80	0.22	0.22	0.22	0.22	0.22	0.22	0.22
6DU5	0.22	/	0.22	0.22	4.86	4.86	5.09	1.45	0.22
6F4G	0.86	0.00	0.00	0.00	3.71	3.71	0.00	1.40	2.00
1QWB	0.00	0.00	0.00	0.00	2.06	2.06	0.00	0.31	0.86
2L5Z	0.00	0.00	0.00	0.00	0.20	0.20	0.00	0.00	0.20
4BW0	0.00	0.50	0.00	0.00	0.88	0.88	0.00	0.50	0.15
4QOZ	0.00	0.00	0.00	0.00	1.25	1.25	0.00	0.00	0.15
4TV0	0.00	0.00	0.00	0.00	1.60	1.60	0.00	0.00	0.00
1F7F	0.00	0.00	0.00	0.00	0.09	0.09	0.00	0.09	0.09
1FQZ	0.00	17.29	0.00	0.00	1.00	1.00	0.00	0.23	0.15
1FYO	0.00	0.00	0.00	0.00	0.29	0.29	0.24	0.11	0.20
1XSG	0.00	0.00	0.00	0.00	0.09	0.09	0.00	0.09	0.09
1XSH	0.00	0.00	0.00	0.00	0.09	0.09	0.00	0.09	0.09
1YSV	0.00	0.00	0.00	0.00	0.09	0.09	0.00	0.00	0.00
2AHT	0.10	0.10	0.10	0.10	0.18	0.18	0.10	0.10	0.10
2IXY	0.14	0.14	0.14	0.14	0.13	0.13	0.14	0.00	0.09
2LDL	0.11	0.00	0.00	0.00	0.38	0.38	0.00	0.11	0.20
2LJJ	0.11	0.11	0.11	0.11	0.11	0.11	0.11	0.11	0.11
2LQZ	0.11	0.11	0.11	0.11	0.20	0.20	0.11	0.20	0.20
484D	0.00	0.63	0.00	0.00	0.30	0.30	0.00	0.22	0.56
6XH0	0.16	0.16	0.16	0.16	0.00	0.00	0.16	0.16	0.00
1ZBN	0.00	0.14	0.14	0.14	0.00	0.00	0.00	0.00	0.00
28SP	0.00	0.00	0.00	0.00	0.70	0.70	0.00	0.29	0.22
2GIP	1.05	0.00	0.00	0.00	0.26	0.26	0.00	0.10	0.26
2KMJ	0.00	0.00	0.00	0.00	0.09	0.09	0.00	0.09	0.09
2LUN	0.00	0.38	0.00	0.00	0.36	0.36	0.00	0.25	0.40
2NC0	0.00	0.00	0.00	0.00	0.18	0.18	0.00	0.00	0.10
2NCI	0.22	0.22	0.22	0.22	0.32	0.32	0.22	0.32	0.32
5M0I	0.15	0.73	/	/	0.81	0.81	0.73	0.73	0.69
6AAS	0.00	0.00	0.00	0.00	0.08	0.08	0.00	0.00	0.00
6SNJ	0.00	0.00	0.00	0.00	0.36	0.36	0.00	0.14	0.14
6VZC	0.00	0.11	0.00	0.00	0.26	0.26	0.00	0.23	0.10
1ANR	0.11	0.11	0.11	0.11	0.25	0.25	0.11	0.11	0.25
1EBS	0.14	0.00	0.00	0.00	0.30	0.30	0.00	0.23	0.10
1F84	0.15	6.40	0.15	0.15	1.05	1.05	0.15	1.29	0.29
1JBT	1.40	0.15	1.00	1.00	1.00	1.00	0.69	1.00	1.40
1L1C	0.00	1.06	4.29	4.29	0.96	0.96	0.00	0.10	0.18
1L1W	0.00	0.00	0.00	0.00	0.18	0.18	0.00	0.00	0.20
1NBR	0.13	0.13	0.13	0.13	0.00	0.00	0.13	0.00	0.08
1OOA	0.00	0.00	0.00	0.00	0.82	0.82	0.00	0.21	0.11
1SCL	0.69	0.00	1.00	1.00	1.00	1.00	0.00	0.27	0.83
2GIO	0.47	0.24	0.24	0.24	0.36	0.36	0.00	0.25	0.50
2JWV	0.00	0.00	0.00	0.00	0.82	0.82	0.00	0.21	0.11
2K5Z	0.00	1.24	0.00	0.00	0.09	0.09	0.00	0.00	0.67
2M24	0.40	0.00	0.00	0.00	1.09	1.09	0.00	0.60	0.40
3SN2	0.13	0.13	0.13	0.13	0.44	0.44	0.13	0.00	0.08
5LM7	0.00	0.00	0.00	0.00	6.80	6.80	0.00	0.00	0.00
5LSN	0.00	0.09	0.09	0.09	0.08	0.08	0.00	0.00	0.26
6DU4	0.00	0.00	0.00	0.00	2.25	2.25	0.00	0.27	1.94
1AUD	0.00	3.18	0.00	0.00	0.20	0.20	0.00	0.25	0.11
1EBR	0.14	0.00	0.00	0.00	0.39	0.39	0.00	0.23	0.30
1EKZ	0.07	0.07	0.07	0.07	0.00	0.00	0.07	0.00	0.00
1HVU	4.00	7.69	4.00	4.00	5.00	5.00	6.53	0.32	0.20
1HWQ	0.11	0.11	0.11	0.11	0.61	0.61	0.11	0.11	0.11
1KP7	0.00	0.00	0.00	0.00	0.33	0.33	0.00	0.25	0.50
1LDZ	0.00	0.00	0.00	0.00	0.23	0.23	0.00	0.00	0.18
1NA2	0.00	1.57	1.57	1.57	0.67	0.67	0.00	0.00	0.25
1RFR	0.00	0.00	0.00	0.00	0.42	0.42	0.00	0.10	0.18
5Y58	0.00	0.00	0.00	0.00	0.27	0.27	0.20	0.20	0.20
6MCE	0.13	0.13	0.13	0.13	0.81	0.81	0.13	0.13	0.00
1JO7	0.18	0.45	0.18	0.18	0.33	0.33	0.33	0.35	0.26
1MFY	0.30	0.18	0.30	0.30	0.52	0.52	0.30	0.50	0.57

PDB	CONTRAfold	CentroidFold	Linearfold-C	Linearfold-V	MC-fold pseudoknot	MC-fold	MXfold2	Ufold	SPOT-RNA
1YNC	0.19	0.10	0.19	0.19	0.43	0.43	0.10	0.43	0.43
1YNG	0.30	0.20	0.30	0.30	0.70	0.70	0.20	0.20	0.29
2LDT	0.00	0.00	0.00	0.00	0.36	0.36	0.10	0.00	0.09
5KMZ	8.76	/	/	/	0.46	4.13	8.76	0.00	8.76
5UZT	0.62	0.30	0.30	0.30	1.48	1.48	1.00	0.68	0.21
6HYK	0.00	0.00	0.00	0.00	0.50	0.50	0.00	0.11	0.11
1G70	0.13	0.00	0.00	0.00	0.36	0.36	0.00	0.21	0.28
1KAJ	8.16	/	5.71	5.71	0.38	8.19	5.71	0.20	1.81
1KPD	6.35	/	6.67	6.67	0.18	8.50	6.67	0.10	2.10
1XHP	0.00	2.06	0.00	0.00	0.24	0.24	0.00	0.00	0.09
1Z31	0.00	0.00	0.00	0.00	0.28	0.28	0.00	0.09	0.17
2LBS	0.00	0.00	0.00	0.00	0.07	0.07	0.00	0.07	0.07
2LI4	0.00	0.00	0.00	0.00	0.07	0.07	0.00	0.00	0.00
2LUP	0.00	0.00	0.00	0.00	0.00	0.00	0.00	0.00	0.00
5A18	0.52	0.52	0.52	0.52	0.24	0.24	0.52	0.00	0.65
1EXY	0.00	0.00	0.00	0.00	0.08	0.08	0.00	0.17	0.24
2JXV	0.12	0.00	0.00	0.00	0.26	0.26	0.00	0.12	0.08
3ID5	11.83	17.00	17.00	17.00	7.93	7.93	8.82	2.80	0.50
1ETF	0.12	0.00	0.00	0.00	0.33	0.33	0.00	0.12	0.15
1P5N	0.11	0.11	0.11	0.11	1.00	1.00	0.00	0.30	0.20
1R2P	0.15	0.08	0.15	0.15	0.22	0.22	0.23	0.15	0.30
1R7W	0.00	0.00	0.00	0.00	2.79	2.79	0.00	0.00	0.00
1R7Z	0.00	0.00	0.00	0.00	0.08	0.08	0.00	0.17	0.00
1RNK	7.44	/	7.88	7.88	1.36	8.14	7.88	0.09	0.78
1T28	0.35	0.11	0.11	0.11	2.96	2.96	0.11	0.11	0.11
2EUY	0.13	18.57	0.13	0.13	0.37	0.37	0.13	0.21	0.21
2F88	0.07	0.07	0.07	0.07	0.14	0.14	0.07	0.07	0.14
2JTP	0.00	0.67	0.67	0.67	0.14	0.14	0.00	0.00	0.07
2KPV	0.00	0.12	0.12	0.12	0.14	0.14	0.12	0.00	0.19
2L3C	0.00	0.00	0.00	0.00	0.13	0.13	0.00	0.07	0.07
2RVO	0.00	0.17	0.00	0.00	0.43	0.43	0.00	0.15	0.14
4OOG	0.00	0.00	0.00	0.00	0.00	0.00	0.00	0.00	0.00
4X4O	0.00	0.00	0.00	0.00	0.16	0.16	0.00	0.75	0.00
6SDY	0.00	0.00	0.00	0.00	0.06	0.06	0.00	0.06	0.06
1ULL	0.00	0.00	0.00	0.00	0.22	0.22	0.00	0.08	0.22
2DRB	0.00	0.00	0.00	0.00	0.16	0.16	0.00	0.16	0.16
2L3E	0.00	0.00	0.00	0.00	0.07	0.07	0.00	0.07	0.07
2M57	0.26	0.29	0.26	0.26	0.33	0.33	0.26	0.26	0.42
2PCV	1.00	6.31	0.16	0.16	1.16	1.16	0.76	0.50	0.40
4C4W	16.69	16.69	/	/	8.05	8.05	0.50	0.53	1.33
5FJ4	9.50	11.25	9.50	9.50	7.81	7.81	0.00	5.33	5.18
6BHJ	0.07	0.07	0.07	0.07	0.07	0.07	0.07	0.07	0.07
1N8X	0.00	0.00	0.00	0.00	0.13	0.13	0.00	0.00	0.00
2FDT	0.07	0.07	0.07	0.07	0.28	0.28	0.07	0.07	0.19
2HW8	0.16	0.00	0.00	0.00	0.30	0.30	0.00	0.23	0.22
2N6S	0.00	0.00	0.14	0.14	0.13	0.13	0.10	0.06	0.10
2TPK	5.00	5.20	5.00	5.00	9.56	9.56	5.20	0.00	0.08
4X4P	0.00	0.00	0.00	0.00	0.16	0.16	0.00	0.79	0.00
5KQE	0.19	0.19	0.19	0.19	0.26	0.26	0.19	0.07	0.26
6SY6	5.50	5.50	5.50	5.50	2.60	2.60	0.18	1.94	0.33
2LHP	0.00	0.00	0.00	0.00	0.13	0.13	0.00	0.06	0.06
2LUB	0.00	0.00	0.00	0.00	0.13	0.13	0.00	0.06	0.06
6DTD	0.32	0.89	0.52	0.52	0.41	0.41	0.17	0.13	0.15
6U79	0.25	0.07	0.00	0.00	0.97	0.97	0.00	0.25	0.56
1B36	0.52	2.10	0.00	0.00	1.52	1.52	0.00	0.77	0.11
1M5L	0.00	0.00	0.00	0.00	0.29	0.29	0.00	0.08	0.26
1TXS	0.00	0.00	0.00	0.00	0.33	0.33	0.00	0.07	0.07
2A9L	0.00	0.00	0.00	0.00	0.07	0.07	0.00	0.00	0.07
2KHY	0.95	0.11	0.11	0.11	1.33	1.33	0.11	0.52	0.52
4PDB	0.29	0.38	0.29	0.29	0.50	0.50	0.29	0.43	0.13
6D12	18.63	19.00	0.23	0.23	19.00	19.00	19.00	18.62	0.23
2MXL	0.00	0.08	0.08	0.08	0.40	0.40	0.08	0.00	0.39
2NBY	0.29	0.37	0.37	0.37	0.27	0.27	0.29	0.00	0.59
4KR7	0.17	0.17	0.17	0.17	3.48	3.48	0.17	1.30	0.00

PDB	CONTRAfold	CentroidFold	Linearfold-C	Linearfold-V	MC-fold pseudoknot	MC-fold	MXfold2	Ufold	SPOT-RNA
4KR9	0.84	0.84	0.84	0.84	3.68	3.68	0.84	1.57	0.60
2HUA	0.12	0.95	13.70	13.70	0.38	0.38	0.17	0.19	0.96
2NBZ	0.06	0.06	0.06	0.06	0.13	0.13	0.00	0.06	0.06
4PMI	0.00	0.17	0.54	0.54	0.37	0.37	0.44	0.28	0.27
1A51	0.38	0.00	0.38	0.38	1.03	1.03	0.38	0.52	1.00
1ZC5	0.12	0.00	0.12	0.12	0.00	0.00	0.06	0.00	0.12
4M6D	1.15	2.41	2.75	2.75	4.48	4.48	1.63	1.63	0.63
5V17	0.00	0.00	0.13	0.13	0.19	0.19	0.00	0.00	0.00
5W1H	8.79	0.44	0.44	0.44	10.26	10.26	10.08	10.08	0.60
6W3M	0.00	0.00	0.00	0.00	0.29	0.29	0.00	0.00	0.06
1MNX	0.31	5.24	0.00	0.00	1.06	1.06	0.16	0.31	1.06
2L2J	0.00	0.00	0.00	0.00	0.16	0.16	0.00	0.06	0.11
2N6T	0.24	0.21	0.56	0.56	0.44	0.44	0.24	0.30	0.27
5WLH	8.79	0.44	0.44	0.44	10.75	10.75	10.08	8.79	0.60
1CQ5	0.25	2.80	0.25	0.25	10.52	10.52	0.25	0.25	0.54
1CQL	0.08	2.57	0.08	0.08	10.27	10.27	0.08	0.08	0.38
2ADT	0.00	0.06	0.00	0.00	0.46	0.46	0.00	0.09	0.06
2FEY	0.31	0.15	0.31	0.31	0.42	0.42	0.54	0.34	0.14
2N6X	0.33	0.28	0.06	0.06	0.46	0.46	0.33	0.38	0.13
1A60	1.80	4.30	1.80	1.80	0.38	2.06	4.08	0.00	1.80
1P6V	0.00	0.00	0.00	0.00	1.55	1.55	0.00	0.08	1.14
1Z2J	0.11	0.00	0.11	0.11	0.00	0.00	0.05	0.00	0.15
1S03	0.39	0.44	0.50	0.50	0.37	0.37	0.22	0.11	0.42
1YMO	7.48	7.75	7.75	7.75	14.32	14.32	13.09	0.88	0.06
2MTJ	0.44	0.13	0.13	0.13	0.51	0.51	0.13	0.06	0.13
2PXL	0.00	15.29	0.00	0.00	11.58	11.58	0.07	0.00	3.08
5KH8	5.57	7.91	5.57	5.57	0.82	5.63	5.57	0.00	0.07
2K95	13.35	/	13.57	13.57	3.54	14.38	13.35	0.88	0.56
2KE6	0.05	0.11	0.05	0.05	0.21	0.21	0.05	0.11	0.16
2KUR	0.00	0.05	0.00	0.00	0.15	0.15	0.00	0.05	0.10
2KUU	0.00	0.05	0.00	0.00	0.15	0.15	0.00	0.05	0.10
2KUV	0.00	0.05	0.00	0.00	0.15	0.15	0.00	0.00	0.10
2KUW	0.05	0.11	0.05	0.05	0.21	0.21	0.11	0.13	0.16
2M8K	5.13	5.23	5.13	5.13	1.72	4.97	4.88	0.12	5.64
2VPL	0.12	0.06	0.12	0.12	0.33	0.33	0.06	0.24	0.28
4C7O	0.05	0.18	0.76	0.76	0.05	0.05	0.50	0.38	0.05
1U63	0.11	0.18	0.11	0.11	0.26	0.26	0.06	0.17	0.21
2LU0	0.00	0.06	1.40	1.40	0.30	0.30	0.00	0.06	1.42
2PXB	0.06	1.46	0.06	0.06	11.91	11.91	0.06	0.06	0.39
2PXD	0.00	3.44	0.00	0.00	13.08	7.38	0.00	1.18	0.32
2PXE	0.00	1.33	0.00	0.00	7.38	7.38	0.00	0.00	0.32
2PXF	0.00	1.14	0.00	0.00	7.38	7.38	0.00	1.18	0.31
2PXK	0.00	1.14	0.00	0.00	7.38	7.38	0.00	0.00	0.35
2PXP	0.00	2.00	0.00	0.00	7.38	7.38	0.00	1.18	0.32
2PXQ	0.00	1.33	0.00	0.00	7.38	7.38	0.00	0.00	0.42
2PXT	0.00	1.14	0.00	0.00	7.38	7.38	0.00	1.18	0.32
2PXU	0.00	1.14	0.00	0.00	7.38	7.38	0.00	1.18	0.32
2PXV	0.00	1.14	0.00	0.00	7.38	7.38	0.00	0.00	0.32
6MXQ	4.26	0.25	4.26	4.26	0.43	0.43	0.00	5.93	5.39
6IV9	0.50	0.24	0.22	0.22	17.13	16.68	0.22	0.53	2.10
6IV8	0.50	0.24	0.22	0.22	18.34	19.10	0.22	0.30	0.32
2MHI	1.62	0.21	1.62	1.62	0.27	0.27	0.15	0.34	0.26
2N4L	0.00	0.05	0.45	0.45	0.13	0.13	0.68	0.07	0.10
1P5M	0.00	1.27	0.08	0.08	0.19	0.19	0.08	0.05	0.35
2HGH	0.76	2.80	0.00	0.00	0.46	0.46	0.64	5.28	0.38
2KZL	0.75	0.07	0.00	0.00	1.15	1.15	0.00	0.50	0.45
2LC8	14.90	21.62	10.00	10.00	5.29	8.91	11.55	1.52	5.12
6NOA	0.21	5.60	5.31	5.31	0.95	0.95	0.00	1.52	5.31
5IEM	0.51	0.82	0.57	0.57	0.67	0.67	0.00	0.16	0.62
6MCF	0.57	0.89	0.63	0.63	0.72	0.72	0.05	0.21	0.68
4M4O	2.37	1.67	0.68	0.68	2.63	2.63	0.68	0.45	0.26
6DB8	1.29	0.69	1.00	1.00	2.16	2.16	1.57	1.22	1.06
1UN6	0.49	0.05	0.05	0.05	0.69	0.69	0.05	2.57	0.45
4U7U	29.42	23.67	26.67	26.67	36.93	36.93	26.67	34.33	0.15

PDB	CONTRAfold	CentroidFold	Linearfold-C	Linearfold-V	MC-fold pseudoknot	MC-fold	MXfold2	Ufold	SPOT-RNA
2N3Q	1.88	4.85	17.31	17.31	7.37	7.37	0.10	0.62	1.68
3EGZ	0.20	0.06	0.00	0.00	9.98	9.98	0.00	0.84	0.06
2NC1	0.51	0.56	0.51	0.51	1.50	1.50	0.51	0.51	1.72
5WT1	0.63	4.94	13.73	13.73	/	12.40	0.63	1.50	0.95
2MQT	0.12	0.24	0.12	0.12	0.25	0.25	0.16	0.08	0.25
2N6W	0.35	0.28	0.16	0.16	0.53	0.53	0.16	0.31	0.48
5HR6	0.96	0.62	0.49	0.49	2.60	2.60	0.49	0.49	0.57
6U8D	0.04	0.04	0.04	0.04	0.04	0.04	0.04	1.13	0.04
3EPJ	1.10	1.00	9.83	9.83	2.41	2.41	0.78	1.70	0.88
1KXK	0.65	0.20	0.26	0.26	0.98	0.98	0.67	0.49	0.79
2DET	0.98	0.86	0.84	0.84	6.00	2.64	0.84	0.39	1.00
2N8V	3.00	3.02	3.16	3.16	0.28	12.85	3.04	0.04	3.47
5HR7	0.91	7.06	0.49	0.49	6.37	2.60	0.49	0.49	0.60
5V6X	0.34	0.34	0.34	0.34	8.23	8.23	0.34	0.74	0.45
2DU3	6.89	0.84	6.89	6.89	7.25	7.25	0.58	0.64	0.84
2DU5	0.82	0.73	0.73	0.73	7.23	7.23	0.51	0.53	0.77
2DU6	1.08	0.73	0.73	0.73	7.23	7.23	0.51	0.53	0.74
2L3J	0.00	0.00	0.00	0.00	0.13	0.13	0.00	0.00	0.13
2MS0	3.49	3.54	3.79	3.79	4.14	4.14	0.61	0.61	0.76
2ZZN	0.88	0.52	0.60	0.60	1.92	1.92	0.49	0.49	0.64
4YVI	10.20	1.20	0.59	0.59	9.67	10.08	0.59	0.50	0.38
4YVJ	9.92	1.20	0.59	0.59	1.62	1.62	0.59	0.22	0.38
4YVK	9.81	1.20	10.04	10.04	1.62	1.62	0.59	0.22	0.42
5TF6	15.53	11.23	26.00	26.00	5.71	5.71	29.83	2.22	6.81
1DRZ	10.62	9.57	16.62	16.62	4.14	12.00	7.22	8.14	3.20
1EUQ	1.83	3.51	0.59	0.59	4.22	10.68	0.49	0.59	0.86
2AKE	0.00	0.15	0.00	0.00	10.10	2.27	0.00	0.00	0.18
2MF0	0.11	11.79	0.11	0.11	7.65	6.09	0.16	0.23	0.11
2ZNI	0.10	0.15	0.10	0.10	9.51	1.00	0.10	0.15	0.27
4ZT0	4.20	5.74	25.33	25.33	6.35	6.31	8.00	0.95	5.64
3WC1	7.35	5.71	7.00	7.00	/	7.57	6.61	0.00	0.14
3WFQ	0.80	0.88	0.83	0.83	/	1.21	0.83	0.83	1.00
4X0A	0.63	0.75	0.64	0.64	7.37	1.15	0.64	0.64	1.16
5VW1	0.12	7.36	25.33	25.33	12.23	14.33	7.36	0.89	6.22
5WT3	0.81	0.55	0.55	0.55	/	/	0.55	0.55	0.83
1GTR	0.54	0.75	0.54	0.54	14.17	2.59	0.54	0.54	0.95
2DER	1.48	1.13	1.05	1.05	3.13	3.13	1.05	0.75	1.14
2ZM5	8.36	0.60	0.60	0.60	10.17	3.94	0.52	0.49	0.70
3AKZ	9.78	1.34	0.50	0.50	9.33	9.66	0.50	0.47	0.38
3FOZ	8.36	0.67	9.96	9.96	10.21	3.94	0.60	0.84	0.76
3TUP	8.42	0.73	8.77	8.77	10.94	10.94	0.70	0.61	0.79
3WC2	9.59	9.22	9.40	9.40	/	7.08	0.49	0.09	0.91
3WFS	0.07	0.07	0.00	0.00	0.53	0.53	0.00	0.00	1.63
4YCO	8.36	0.60	0.60	0.60	3.94	3.94	0.52	0.40	0.70
4YYE	0.79	13.52	18.16	18.16	2.74	2.74	0.88	0.51	0.64
5D6G	0.92	0.87	0.68	0.68	2.02	2.02	0.41	1.49	2.74
1FFY	0.57	5.94	9.78	9.78	10.22	4.94	0.51	0.51	0.71
1N77	0.52	0.61	0.52	0.52	12.76	0.88	0.52	0.49	0.83
2DR2	0.15	0.44	0.15	0.15	3.84	11.18	0.15	0.15	0.33
2IHX	0.27	14.29	1.76	1.76	15.36	15.36	0.00	2.04	1.61
2ZUE	2.87	4.44	2.78	2.78	15.71	2.98	0.51	0.51	0.61
3WQY	9.92	3.83	11.11	11.11	/	11.20	0.52	0.52	0.77
3WQZ	9.84	3.79	11.19	11.19	/	4.10	0.60	0.60	0.80
4TZV	3.52	3.65	3.78	3.78	8.82	2.50	3.53	1.79	0.71
4WC2	0.53	0.60	0.55	0.55	0.96	0.96	0.55	0.55	0.81
5X6B	1.04	0.69	0.77	0.77	/	1.80	0.66	0.66	0.54
1EIY	0.57	0.60	0.49	0.49	11.61	11.15	0.57	0.49	0.70
2K4C	3.13	2.98	3.13	3.13	13.11	5.33	0.49	0.65	0.94
4WC3	0.53	0.60	0.55	0.55	0.96	0.96	0.55	0.55	0.81
4WJ3	0.55	5.86	2.27	2.27	6.64	6.64	0.55	0.77	0.70
1P5P	0.18	12.88	0.10	0.10	0.53	0.53	9.38	0.16	11.33
3A2K	7.73	12.65	7.73	7.73	8.13	8.13	0.51	0.51	0.67
4X0B	0.48	0.43	0.31	0.31	1.00	1.00	0.31	0.31	2.26
5CCB	2.38	5.95	2.35	2.35	2.85	2.85	0.18	0.23	1.20

PDB	CONTRAfold	CentroidFold	Linearfold-C	Linearfold-V	MC-fold pseudoknot	MC-fold	MXfold2	Ufold	SPOT-RNA
3AMT	1.57	4.15	4.14	4.14	8.09	8.09	0.50	0.50	1.00
3U4M	1.34	13.02	3.02	3.02	/	2.25	1.37	3.51	3.21
6B14	3.65	6.31	5.06	26.51	13.45	13.45	6.06	5.96	1.33
6B3K	10.25	9.77	10.16	10.98	14.72	14.72	10.16	6.57	2.51
2ZZM	9.87	6.70	15.36	13.65	/	/	1.16	1.31	3.23
3A3A	0.48	0.53	0.48	10.58	0.55	0.55	0.38	0.24	0.55
3K0J	5.13	5.76	14.54	14.54	19.30	15.60	0.20	5.13	0.29
1WZ2	1.73	8.36	6.26	13.56	/	/	1.36	0.52	0.23
5XBL	1.05	5.69	11.88	31.05	14.34	5.92	5.93	5.25	1.28
2N7M	0.09	2.36	0.03	2.69	0.35	0.35	0.00	0.11	0.60
3ADB	0.31	0.31	0.31	11.29	/	/	0.34	0.53	0.39
3W1K	0.37	2.23	0.37	15.98	/	/	0.37	0.42	0.53
2V3C	0.10	0.28	3.45	8.29	/	/	0.03	3.60	3.73
3KTW	5.94	4.18	0.13	8.48	/	8.63	0.23	4.63	0.52
1LNG	0.10	0.28	2.55	8.53	0.55	0.55	0.07	2.78	3.00
6JXM	0.43	7.45	0.43	15.02	1.99	1.99	0.43	0.51	0.52
3W3S	0.33	0.61	0.53	17.09	/	/	0.45	0.39	0.50
1S9S	0.00	3.23	0.00	10.32	/	/	0.00	0.00	1.76
6MJ0	10.98	10.27	9.15	13.67	/	/	10.52	3.46	1.38
2KRL	1.49	4.54	1.49	21.11	/	/	1.49	0.17	3.61
2XXA	30.91	5.51	0.21	8.31	8.88	8.88	0.00	0.00	3.45
7K1Z	1.53	2.32	1.72	11.18	/	/	2.16	2.03	2.46
2NBX	0.69	0.76	0.73	11.98	/	/	0.62	0.05	1.58
2LKR	1.50	3.89	22.35	22.35	0.95	0.95	2.09	0.13	7.70
4P3E	0.88	3.75	1.23	20.91	1.47	1.47	1.31	0.56	6.31
3IVK	4.92	5.79	4.92	25.80	2.80	4.70	4.92	4.90	2.23
3NDB	0.13	0.22	0.12	31.19	15.68	15.68	0.06	0.31	4.40
2N1Q	7.18	8.41	11.06	40.97	6.03	6.03	9.10	0.08	6.80
2R8S	2.36	9.19	0.47	48.15	2.07	17.23	3.95	2.40	19.63
4P8Z	7.91	15.97	11.73	113.59	134.65	134.65	6.03	0.79	17.67
1GRZ	3.66	12.10	1.29	129.03	/	/	2.67	1.72	15.75
5IWA	21.29	27.60	29.69	1335.81	/	/	12.79	/	51.14
