# Supplementary material for: Comparative Study of Single-stranded Oligonucleotides Secondary Structure Prediction Tools
Source: BMC Bioinformatics. 2023 Nov 8;24:422. doi: 10.1186/s12859-023-05532-5 (PMC10634105; doi:10.1186/s12859-023-05532-5)
Supplement: Supplementary file 4 — Additional file 4. Comparison between predicted and experimental secondary structure using the AptaMat distance as a metric for RNAfold under RNA (Turner (2004)) and DNA (Mathews (2004)) model. The PDB code is reported in the first column.$Apta_D$AptaD values are reported for each PDB and associated optimal/suboptimal prediction. "/" characters indicate either structures predicted as unfolded or software failure during the computation. [file 12859_2023_5532_MOESM4_ESM.pdf]

Additional File 4. Comparison between predicted and experimental secondary structure using the AptaMat distance as a metric for RNAfold under RNA (Turner (2004)) and DNA (Mathews (2004)) model. The PDB code is reported in the first column. AptaD values are reported for each PDB and associated optimal/suboptimal prediction. "/" characters indicate either structures predicted as unfolded or software failure during the computation.

	DNA model									
PDB	RNAfold MFE	subopt #2	subopt #3	subopt #4	subopt #5	subopt #6	subopt #7	subopt #8	subopt #9	subopt #10
1PQT	/	0.00	2.00	0.67	0.67	/	/	/	/	/
2K71	/	0.00	2.00	0.67	0.67	/	/	/	/	/
5GWL	/	3.67	3.33	4.00	/	/	/	/	/	/
5GWQ	/	3.67	3.33	4.00	/	/	/	/	/	/
6J37	/	3.33	3.67	4.00	/	/	/	/	/	/
6M0B	/	3.33	3.33	3.67	3.67	4.00	/	/	/	/
6M0C	/	3.33	3.67	3.67	4.00	3.50	/	/	/	/
5OND	/	1.00	0.50	3.33	2.33	3.33	2.33	2.50	0.00	1.33
1ZHU	/	0.00	2.00	4.00	0.67	2.00	2.00	6.00	0.67	1.67
2A0I	/	2.00	3.00	5.00	5.00	1.00	7.00	3.67	3.00	5.00
2LO8	/	3.20	0.60	2.00	1.60	1.75	0.00	4.50	1.80	3.00
3WPD	/	0.00	1.00	1.00	1.67	2.00	2.00	1.00	0.67	2.00
6IY5	/	0.00	8.00	2.00	0.67	0.67	1.67	3.67	/	/
1BJH	0.00	/	0.29	0.29	1.00	3.40	2.40	1.00	0.67	4.40
3WPG	/	1.00	0.00	1.00	2.00	1.67	2.00	1.00	0.67	2.00
2LO5	0.43	/	0.00	3.50	5.60	0.83	0.57	2.00	2.80	0.50
3WPH	/	1.00	0.00	1.00	2.00	1.67	2.00	9.00	1.00	0.67
6FKE	0.00	0.29	/	0.29	1.00	0.29	0.67	3.00	8.00	5.00
1LA8	0.00	0.22	0.22	0.33	0.33	0.50	0.75	0.75	0.22	/
1P0U	0.00	0.22	0.22	/	0.75	0.22	0.22	0.50	0.75	3.29
2EXF	0.00	1.00	0.43	/	1.00	1.00	1.00	0.40	1.29	2.50
2JZW	0.00	1.00	0.43	/	1.00	1.00	1.00	0.40	1.29	2.50
5F55	/	3.00	11.00	0.00	6.00	1.00	5.00	8.00	3.67	3.00
6FK5	0.00	0.22	0.22	0.75	/	0.22	0.22	1.71	4.00	0.50
1UUT	0.00	0.18	0.18	0.60	0.40	0.60	0.18	0.18	0.89	/
2M8Y	0.00	0.18	0.18	0.60	0.27	0.18	0.40	0.27	0.60	0.18
1AC7	0.00	0.18	0.18	0.60	0.18	0.40	/	0.18	0.60	0.40
6FK4	0.00	4.00	4.00	0.22	0.22	0.75	/	0.22	0.22	4.00
1XUE	/	6.33	8.00	7.00	7.00	3.00	5.00	8.40	8.00	8.00
1EN1	0.43	0.00	1.43	/	0.75	1.38	12.17	0.50	1.50	1.14
4KB0	0.33	0.36	0.55	0.17	0.33	0.55	1.00	0.80	0.00	0.55
4KB1	0.33	0.55	0.36	0.55	1.00	0.55	0.45	0.80	0.00	0.17
1ECU	0.00	0.13	0.13	0.43	0.13	0.13	0.29	0.33	0.20	0.20
3Q0A	0.00	0.22	0.22	1.73	1.82	0.75	/	0.22	0.22	4.00
4FF1	0.00	0.22	3.33	0.22	1.73	17.00	0.75	2.00	1.91	/
3C46	0.18	0.00	0.40	1.92	0.27	0.22	2.00	0.40	0.40	0.36
3Q23	0.18	0.00	0.40	1.92	0.27	0.22	2.00	0.40	0.40	0.36
2A6O	0.00	0.13	0.47	0.13	0.13	0.47	0.67	0.13	2.00	0.13
3Q24	0.18	0.00	1.92	0.40	2.00	0.27	0.22	0.40	0.40	0.36
2L5K	2.73	0.00	1.38	0.20	11.67	/	2.00	3.80	3.20	0.20
3DSD	0.00	0.15	0.27	0.14	0.15	0.15	0.15	0.15	0.15	0.50
2VHG	0.00	0.13	0.47	0.13	0.13	0.47	0.67	0.13	2.00	0.13
1OSB	0.18	0.50	1.92	2.08	2.25	2.23	2.38	0.67	0.00	0.75
1ZM5	0.18	0.50	1.92	2.08	2.25	2.23	2.38	0.67	0.00	0.75
2CDM	2.50	0.93	2.55	0.46	0.62	1.23	0.92	0.62	2.91	0.92

	RNA model									
PDB	RNAfold MFE	subopt #2	subopt #3	subopt #4	subopt #5	subopt #6	subopt #7	subopt #8	subopt #9	subopt #10
1PQT	/	0.00	0.67	0.67	/	/	/	/	/	/
2K71	0.00	/	0.67	2.00	0.67	/	/	/	/	/
5GWL	/	3.67	4.00	/	/	/	/	/	/	/
5GWQ	/	4.00	/	/	/	/	/	/	/	/
6J37	/	4.00	/	/	/	/	/	/	/	/
6M0B	/	4.00	3.33	/	/	/	/	/	/	/
6M0C	/	4.00	/	/	/	/	/	/	/	/
5OND	/	0.50	1.00	0.00	2.33	3.33	2.50	2.33	/	/
1ZHU	/	2.00	3.67	0.00	1.67	6.00	0.67	/	/	/
2A0I	/	2.00	3.33	3.00	3.67	6.00	/	/	/	/
2LO8	/	0.60	3.20	1.60	0.00	2.00	1.25	1.75	4.50	1.80
3WPD	0.00	1.00	/	1.00	1.00	0.67	2.00	1.00	1.00	0.67
6IY5	/	1.67	/	/	/	/	/	/	/	/
1BJH	0.00	/	0.29	0.29	1.00	1.00	0.67	2.40	0.29	0.29
3WPG	1.00	0.00	1.00	/	2.00	1.00	0.67	1.00	0.50	1.00
2LO5	0.43	0.00	/	0.83	3.50	1.17	0.57	0.29	0.50	0.75
3WPH	1.00	0.00	1.00	/	2.00	1.00	0.67	1.00	0.50	1.00
6FKE	0.00	0.29	/	1.00	3.00	0.29	0.67	3.40	0.29	1.60
1LA8	0.00	0.22	0.22	0.50	0.75	0.75	/	/	/	/
1P0U	0.00	0.22	0.22	/	0.75	0.22	0.22	0.50	/	/
2EXF	1.29	0.00	0.43	1.00	1.00	1.00	0.40	/	1.00	1.67
2JZW	1.29	0.00	0.43	1.00	1.00	1.00	0.40	/	1.00	1.67
5F55	/	0.00	3.00	1.00	11.00	3.67	2.00	6.00	9.00	3.50
6FK5	0.00	0.22	0.22	0.75	0.22	0.22	/	/	/	/
1UUT	0.00	0.18	0.18	0.60	0.60	0.40	0.18	0.18	/	/
2M8Y	0.00	0.18	0.18	0.40	0.60	0.89	0.27	0.27	0.18	1.33
1AC7	0.00	0.18	0.60	0.18	0.40	0.60	0.89	0.89	0.18	0.18
6FK4	0.00	4.00	0.22	0.22	4.00	4.00	0.75	0.22	0.22	/
1XUE	/	6.33	8.00	8.00	7.20	5.00	7.00	7.00	3.00	8.00
1EN1	0.75	0.00	0.67	0.43	1.33	0.75	1.00	1.38	0.50	0.25
4KB0	0.33	0.55	0.36	0.17	0.33	0.60	1.00	0.36	0.55	/
4KB1	0.33	0.55	0.36	1.00	0.60	/	/	/	/	/
1ECU	0.00	0.13	0.43	0.13	0.29	0.92	0.13	0.62	0.33	0.20
3Q0A	0.00	0.22	0.22	0.75	/	0.22	0.22	3.25	1.71	15.00
4FF1	0.00	0.22	0.22	0.75	/	17.00	0.22	4.00	0.22	3.25
3C46	0.18	0.00	0.40	0.22	0.27	0.22	12.00	0.40	0.40	0.75
3Q23	0.18	0.00	0.40	0.22	0.27	0.22	12.00	0.40	0.40	0.75
2A6O	0.00	0.13	0.47	0.13	0.13	0.36	0.67	0.47	0.13	0.13
3Q24	0.18	0.00	0.40	0.22	0.27	0.22	12.00	0.40	0.40	0.75
2L5K	0.00	2.73	0.20	0.15	0.15	0.15	1.38	2.00	0.15	0.50
3DSD	0.00	0.15	0.14	0.15	0.15	0.15	0.27	0.50	0.15	/
2VHG	0.00	0.13	0.47	0.13	0.13	0.36	0.67	0.47	0.13	0.13
1OSB	0.50	0.18	0.00	0.73	0.73	/	/	/	/	/
1ZM5	0.50	0.18	0.00	0.73	0.73	/	/	/	/	/
2CDM	2.50	2.55	1.00	0.93	2.91	2.91	0.62	/	/	/

	DNA model											RNA model									
PDB	RNAfold MFE	subopt #2	subopt #3	subopt #4	subopt #5	subopt #6	subopt #7	subopt #8	subopt #9	subopt #10		RNAfold MFE	subopt #2	subopt #3	subopt #4	subopt #5	subopt #6	subopt #7	subopt #8	subopt #9	subopt #10
2VIC	0.12	0.25	0.56	0.00	0.25	0.25	0.13	0.47	0.56	0.25		0.12	0.00	0.56	0.25	0.25	0.47	0.13	0.13	0.73	0.40
5N2Q	0.00	0.15	0.15	1.67	0.15	0.15	1.53	1.67	1.80	1.80		0.00	0.15	0.15	0.50	0.33	0.15	0.50	0.15	3.13	0.15
1JVE	0.00	0.09	0.09	0.09	0.09	0.09	0.09	0.09	0.09	0.09		0.00	0.09	0.09	0.27	0.09	0.09	0.09	0.09	0.09	0.18
1NGO	0.00	0.10	0.10	1.00	0.10	0.20	0.10	2.63	0.20	0.10		0.00	0.10	0.10	0.09	0.10	0.30	0.20	0.30	0.10	0.10
1NGU	0.00	0.12	2.57	0.12	12.15	0.12	0.82	0.38	0.12	3.54		0.00	0.12	0.12	0.12	0.12	0.82	0.38	0.25	0.38	0.25
3ZH2	2.00	3.31	1.73	2.00	1.57	2.27	2.29	1.62	1.93	3.13		2.00	2.27	1.73	1.80	2.71	2.29	1.57	3.31	1.73	2.00
4HT4	0.00	2.07	0.15	1.67	6.93	1.53	0.15	0.40	0.15	0.15		0.00	0.15	0.15	2.07	0.15	5.00	0.40	0.15	3.71	1.80
1YTB	0.00	0.09	0.27	0.09	0.09	0.09	0.09	0.09	0.08	0.57		0.00	0.09	0.27	0.09	0.08	0.09	0.09	0.09	0.09	0.09
1B4Y	0.00	0.18	6.00	0.18	0.18	0.18	6.60	0.60	6.18	6.00		6.00	0.00	0.18	6.00	0.18	6.00	5.54	6.00	6.00	6.00
4ER8	0.11	0.22	1.44	0.33	0.22	0.22	1.65	0.22	0.47	0.32		0.11	0.22	0.33	0.22	0.47	0.35	3.30	0.47	0.47	1.84
4F41	0.07	0.14	0.21	0.44	0.30	0.14	0.14	0.14	0.14	0.14		0.07	0.21	0.14	0.44	0.30	0.30	0.14	0.14	0.10	0.54
4F43	0.07	0.14	0.21	0.44	0.30	0.14	0.14	0.14	0.14	0.14		0.07	0.21	0.14	0.44	0.30	0.30	0.14	0.14	0.10	0.54
5HRU	1.20	5.00	4.00	4.12	4.11	1.37	5.47	1.37	4.35	1.67		4.11	4.00	5.62	4.12	1.20	5.60	5.00	1.37	5.79	4.90
6SEI	0.00	0.09	0.86	0.09	2.42	0.09	0.27	0.09	0.09	0.09		0.00	0.09	0.09	0.18	0.86	0.27	1.00	0.38	0.09	0.09
5HTO	1.09	1.24	1.24	5.82	1.50	4.47	4.72	4.45	1.30	1.17		4.45	5.86	1.24	1.09	4.47	6.61	4.72	5.95	5.19	5.82
2VJU	1.05	0.80	1.22	0.95	2.30	1.22	0.95	1.22	2.55	0.38		1.05	1.22	0.80	1.22	0.10	0.95	2.30	0.85	0.55	0.20
1EZN	0.14	1.44	1.50	0.82	0.30	0.74	2.80	0.29	0.22	2.81		3.44	3.43	3.65	3.63	3.25	2.81	3.24	3.38	3.93	3.96
1SNJ	0.14	9.31	10.32	0.22	0.30	0.22	9.48	10.04	10.46	0.30		4.16	9.31	3.78	4.42	10.32	9.48	10.04	10.46	4.00	4.00
6U82	0.00	0.06	0.06	0.06	0.06	2.31	0.06	0.06	0.06	0.06		0.00	0.06	0.06	0.06	0.20	0.06	0.06	0.06	0.06	0.06
3HXO	10.17	10.00	9.96	9.95	5.18	8.05	0.44	9.78	9.74	0.21		8.91	9.00	10.17	9.00	9.04	8.39	9.95	8.90	7.43	7.71
2N8A	0.05	0.17	0.11	0.11	0.11	0.11	0.11	0.34	0.11	0.11		0.05	0.11	0.17	0.23	0.11	0.34	0.11	0.11	0.11	0.17
3THW	0.00	0.04	0.04	0.04	0.04	0.14	0.14	0.04	0.04	0.04		0.00	0.04	0.04	0.04	0.14	0.09	0.04	0.04	0.04	0.04
2IXZ	/	/	/	/	/	/	/	/	/	/		/	0.50	1.33	1.00	1.00	/	/	/	/	/
2OJ7	/	/	/	/	/	/	/	/	/	/		/	0.00	0.67	/	/	/	/	/	/	/
1R4H	/	/	/	/	/	/	/	/	/	/		0.67	1.40	/	0.40	1.60	1.40	2.50	1.50	1.50	/
1IDV	/	/	/	/	/	/	/	/	/	/		0.00	1.00	/	0.40	0.40	1.00	2.00	1.50	1.00	/
2MXJ	/	/	/	/	/	/	/	/	/	/		0.00	/	0.60	0.67	1.00	0.67	1.00	3.67	1.50	/
5FMZ	/	/	/	/	/	/	/	/	/	/		/	0.00	0.67	3.00	4.00	2.00	6.00	0.67	/	/
1RNG	/	/	/	/	/	/	/	/	/	/		0.22	2.29	0.50	0.75	/	1.14	1.14	2.29	3.33	0.63
2F87	/	/	/	/	/	/	/	/	/	/		0.00	0.29	0.29	0.67	/	1.00	/	/	/	/
1ZIF	/	/	/	/	/	/	/	/	/	/		0.00	0.29	1.00	0.71	0.43	1.33	0.29	0.67	/	/
1ZIG	/	/	/	/	/	/	/	/	/	/		0.00	0.29	1.00	0.71	0.43	1.33	0.29	0.67	/	/
1ZIH	/	/	/	/	/	/	/	/	/	/		0.00	0.29	1.00	0.71	0.43	1.33	0.29	0.67	/	/
1AFX	/	/	/	/	/	/	/	/	/	/		0.00	0.29	0.29	/	2.33	1.00	1.57	0.29	3.00	0.67
4Z0C	/	/	/	/	/	/	/	/	/	/		0.67	/	1.00	1.67	2.00	5.00	2.00	0.00	1.00	1.00
1VOP	/	/	/	/	/	/	/	/	/	/		0.22	0.00	0.50	/	0.29	1.14	0.29	0.50	1.00	/
1HS8	/	/	/	/	/	/	/	/	/	/		0.00	0.40	/	0.40	2.00	/	/	/	/	/
1HS4	/	/	/	/	/	/	/	/	/	/		0.00	0.40	0.43	/	0.40	2.00	/	/	/	/
1HS1	/	/	/	/	/	/	/	/	/	/		0.00	0.40	/	0.40	2.00	/	/	/	/	/
1HS2	/	/	/	/	/	/	/	/	/	/		0.00	0.40	0.43	/	0.40	3.00	2.00	/	/	/
1HS3	/	/	/	/	/	/	/	/	/	/		0.00	0.40	/	0.40	2.00	/	/	/	/	/
6FQ3	/	/	/	/	/	/	/	/	/	/		0.00	0.22	0.22	0.50	0.75	/	6.00	0.22	/	/
1ESH	/	/	/	/	/	/	/	/	/	/		0.00	0.22	0.22	0.22	0.50	/	/	/	/	/
1JZC	/	/	/	/	/	/	/	/	/	/		0.00	0.22	0.22	0.22	0.50	/	/	/	/	/
1I46	/	/	/	/	/	/	/	/	/	/		0.00	0.22	0.22	0.22	0.50	/	/	/	/	/
1I4B	/	/	/	/	/	/	/	/	/	/		0.00	0.22	0.22	0.22	0.50	/	/	/	/	/
6FQL	/	/	/	/	/	/	/	/	/	/		0.00	0.22	0.22	0.50	0.75	1.14	/	0.75	0.22	0.50
4Z7L	/	/	/	/	/	/	/	/	/	/		/	0.00	4.00	0.67	6.67	/	/	/	/	/

	DNA model											RNA model									
PDB	RNAfold MFE	subopt #2	subopt #3	subopt #4	subopt #5	subopt #6	subopt #7	subopt #8	subopt #9	subopt #10		RNAfold MFE	subopt #2	subopt #3	subopt #4	subopt #5	subopt #6	subopt #7	subopt #8	subopt #9	subopt #10
2KOC	/	/	/	/	/	/	/	/	/	/		0.00	0.22	0.22	0.75	0.22	/	/	/	/	/
1F85	/	/	/	/	/	/	/	/	/	/		0.00	0.22	0.22	0.75	0.50	1.75	1.00	0.33	0.22	1.86
2Y95	/	/	/	/	/	/	/	/	/	/		0.00	0.22	0.22	0.20	/	/	/	/	/	/
1FHK	/	/	/	/	/	/	/	/	/	/		0.29	0.00	0.67	3.00	0.40	/	3.29	0.88	1.00	0.43
1IK1	/	/	/	/	/	/	/	/	/	/		0.00	0.22	0.75	0.22	0.22	0.22	/	/	/	/
1ROQ	/	/	/	/	/	/	/	/	/	/		0.00	0.22	0.22	0.75	/	0.20	0.50	6.00	0.20	0.22
2EVY	/	/	/	/	/	/	/	/	/	/		0.22	0.00	0.50	0.29	/	0.29	0.50	0.50	/	/
1K4A	/	/	/	/	/	/	/	/	/	/		0.00	0.22	0.22	0.22	0.22	0.75	/	/	/	/
1K4B	/	/	/	/	/	/	/	/	/	/		0.00	0.22	0.22	0.22	0.22	0.75	/	/	/	/
4AL7	/	/	/	/	/	/	/	/	/	/		0.00	0.22	0.75	0.22	0.22	/	/	/	/	/
1OQ0	/	/	/	/	/	/	/	/	/	/		0.00	0.18	0.18	0.40	0.60	0.60	2.22	0.89	0.18	0.18
2LPA	/	/	/	/	/	/	/	/	/	/		0.00	0.18	0.18	0.60	0.60	0.18	1.33	0.18	/	0.40
1QFQ	/	/	/	/	/	/	/	/	/	/		0.00	0.22	0.22	0.20	/	/	/	/	/	/
1A4T	/	/	/	/	/	/	/	/	/	/		0.00	0.22	0.22	0.20	0.75	/	/	/	/	/
1ATW	/	/	/	/	/	/	/	/	/	/		0.00	0.22	0.22	0.22	/	/	/	/	/	/
1XWP	/	/	/	/	/	/	/	/	/	/		0.00	0.29	0.33	0.29	0.29	/	/	/	/	/
1Q75	/	/	/	/	/	/	/	/	/	/		0.00	0.22	0.22	0.22	/	/	/	/	/	/
4AL5	/	/	/	/	/	/	/	/	/	/		0.00	0.22	0.75	0.22	0.22	/	/	/	/	/
2MNC	/	/	/	/	/	/	/	/	/	/		0.18	0.36	0.33	0.00	0.20	0.20	0.40	0.60	0.63	0.40
1XWU	/	/	/	/	/	/	/	/	/	/		0.44	0.00	1.00	/	0.75	0.22	2.00	0.22	1.43	5.00
2LP9	/	/	/	/	/	/	/	/	/	/		0.27	0.70	0.00	0.50	/	1.00	1.00	3.38	1.30	0.50
2L6I	/	/	/	/	/	/	/	/	/	/		0.27	0.70	0.50	/	0.50	0.50	1.44	/	/	/
4ILM	/	/	/	/	/	/	/	/	/	/		/	0.29	5.00	0.67	0.00	0.40	5.00	0.40	11.00	7.00
1JWC	/	/	/	/	/	/	/	/	/	/		0.00	0.18	0.18	0.40	0.17	0.60	/	/	/	/
1JTW	/	/	/	/	/	/	/	/	/	/		0.00	0.22	0.22	0.33	0.75	1.71	0.67	2.25	0.22	0.44
4QIL	/	/	/	/	/	/	/	/	/	/		0.00	0.18	0.18	0.60	0.18	0.40	0.18	/	/	/
6CYT	/	/	/	/	/	/	/	/	/	/		0.83	0.60	/	6.50	6.43	0.29	6.40	7.40	0.00	6.33
2JR4	/	/	/	/	/	/	/	/	/	/		0.00	0.22	0.36	0.27	0.45	0.64	0.20	0.40	0.22	0.60
2KRP	/	/	/	/	/	/	/	/	/	/		0.00	0.22	0.27	0.36	0.75	5.25	0.36	0.22	0.50	0.60
1YN1	/	/	/	/	/	/	/	/	/	/		0.00	0.18	0.18	0.17	0.60	0.40	0.18	/	/	/
2M4W	/	/	/	/	/	/	/	/	/	/		0.40	0.22	0.22	2.00	0.67	0.67	0.63	/	2.00	0.50
1WKS	/	/	/	/	/	/	/	/	/	/		0.00	0.18	0.18	0.18	0.60	0.18	0.27	0.45	0.40	0.50
1ATV	/	/	/	/	/	/	/	/	/	/		0.00	0.18	0.18	/	/	/	/	/	/	/
2LBL	/	/	/	/	/	/	/	/	/	/		0.00	0.22	7.00	6.25	0.36	0.27	0.45	7.00	0.75	6.29
2LBK	/	/	/	/	/	/	/	/	/	/		0.00	0.18	6.00	0.60	6.00	0.18	0.18	0.27	/	/
2LBJ	/	/	/	/	/	/	/	/	/	/		0.00	0.15	0.50	0.15	0.33	0.23	0.15	0.23	0.38	0.73
2LAC	/	/	/	/	/	/	/	/	/	/		0.00	0.27	0.22	0.36	0.22	1.00	0.50	0.50	0.50	1.25
1KKA	/	/	/	/	/	/	/	/	/	/		0.15	0.00	0.18	0.33	0.18	0.17	0.60	0.33	0.17	0.40
2KVN	/	/	/	/	/	/	/	/	/	/		0.00	0.22	0.22	0.45	0.27	0.22	/	/	/	/
4ZLD	/	/	/	/	/	/	/	/	/	/		0.15	0.00	0.18	0.33	0.60	0.18	/	0.73	0.40	0.33
1BZ2	/	/	/	/	/	/	/	/	/	/		0.00	0.22	0.22	0.50	0.36	0.27	0.60	0.50	/	0.75
1BZ3	/	/	/	/	/	/	/	/	/	/		0.36	0.60	0.80	1.11	0.00	0.17	0.18	0.36	/	1.11
2KPC	/	/	/	/	/	/	/	/	/	/		0.00	0.18	0.18	0.40	0.17	0.36	0.60	0.89	0.18	/
2KPD	/	/	/	/	/	/	/	/	/	/		0.00	0.22	0.75	0.22	0.50	0.22	/	/	/	/
2GVO	/	/	/	/	/	/	/	/	/	/		0.00	0.18	0.60	0.18	0.18	0.17	0.18	0.18	/	/
2QH4	/	/	/	/	/	/	/	/	/	/		0.00	0.18	0.18	0.40	0.18	0.23	/	/	/	/
1Z30	/	/	/	/	/	/	/	/	/	/		0.00	0.15	0.50	0.15	0.15	0.23	0.15	0.15	1.09	/
2Y9H	/	/	/	/	/	/	/	/	/	/		0.00	0.15	0.15	0.50	0.33	0.50	0.73	0.15	/	/

	DNA model											RNA model									
PDB	RNAfold MFE	subopt #2	subopt #3	subopt #4	subopt #5	subopt #6	subopt #7	subopt #8	subopt #9	subopt #10		RNAfold MFE	subopt #2	subopt #3	subopt #4	subopt #5	subopt #6	subopt #7	subopt #8	subopt #9	subopt #10
4QI2	/	/	/	/	/	/	/	/	/	/		0.15	0.00	7.00	0.33	0.18	7.00	/	0.18	0.73	0.33
5N5C	/	/	/	/	/	/	/	/	/	/		0.29	0.15	0.15	0.33	0.00	0.18	0.33	0.73	0.18	0.40
6TQB	/	/	/	/	/	/	/	/	/	/		0.00	0.13	0.43	0.13	0.29	0.92	0.13	0.13	0.13	0.13
2B7G	/	/	/	/	/	/	/	/	/	/		0.00	0.15	0.15	0.50	0.33	1.09	0.15	0.15	0.15	0.73
2B6G	/	/	/	/	/	/	/	/	/	/		0.20	0.36	0.50	0.69	0.69	1.25	0.36	0.36	0.36	1.08
1ATO	/	/	/	/	/	/	/	/	/	/		0.00	0.31	0.18	0.23	0.50	0.18	0.18	0.42	/	/
2MEQ	/	/	/	/	/	/	/	/	/	/		0.00	0.18	0.18	0.38	0.23	0.31	0.60	0.40	0.17	0.58
1ESY	/	/	/	/	/	/	/	/	/	/		0.15	0.33	0.33	0.33	0.00	1.20	0.55	/	/	/
1UUU	/	/	/	/	/	/	/	/	/	/		0.29	0.15	0.15	0.00	0.46	0.46	/	/	/	/
2MFD	/	/	/	/	/	/	/	/	/	/		0.00	0.15	0.50	0.15	0.23	0.15	0.15	0.15	1.09	/
1I3X	/	/	/	/	/	/	/	/	/	/		0.00	0.15	0.15	0.15	/	/	/	/	/	/
2RLU	/	/	/	/	/	/	/	/	/	/		0.00	0.13	0.15	0.29	0.15	0.14	0.15	0.15	0.29	0.29
2Y8Y	/	/	/	/	/	/	/	/	/	/		0.15	0.00	0.33	0.73	0.18	0.18	0.33	/	/	/
1SLP	/	/	/	/	/	/	/	/	/	/		0.15	0.00	0.33	0.15	0.50	0.91	0.67	0.73	1.09	0.58
4L8H	/	/	/	/	/	/	/	/	/	/		0.00	0.13	0.43	0.13	0.13	0.29	0.29	0.13	0.62	0.62
1MFJ	/	/	/	/	/	/	/	/	/	/		0.00	0.15	0.50	0.15	0.15	0.15	/	/	/	/
1A1T	/	/	/	/	/	/	/	/	/	/		0.00	0.13	0.13	0.13	0.13	/	/	/	/	/
6PK9	/	/	/	/	/	/	/	/	/	/		0.00	0.15	0.15	0.20	0.20	0.36	0.15	0.33	2.00	2.18
2RPT	/	/	/	/	/	/	/	/	/	/		0.00	0.15	0.15	0.15	0.15	0.14	0.14	0.29	0.29	0.43
1HLX	/	/	/	/	/	/	/	/	/	/		0.00	0.13	0.13	0.13	0.13	0.13	0.43	0.13	/	/
2RPK	/	/	/	/	/	/	/	/	/	/		0.00	0.15	0.20	0.15	0.15	0.15	/	/	/	/
2JPP	/	/	/	/	/	/	/	/	/	/		0.13	0.00	0.29	0.15	0.29	0.15	0.29	0.15	0.15	/
1U2A	/	/	/	/	/	/	/	/	/	/		0.00	0.15	0.15	0.33	0.20	0.15	0.20	0.33	/	/
2O33	/	/	/	/	/	/	/	/	/	/		0.00	0.18	0.18	2.67	0.18	/	/	/	/	/
2Y8W	/	/	/	/	/	/	/	/	/	/		0.15	0.00	0.33	0.73	0.18	0.18	0.33	/	/	/
5F5F	/	/	/	/	/	/	/	/	/	/		0.00	0.18	0.60	1.33	0.18	0.17	0.36	0.40	0.36	0.60
5ID6	/	/	/	/	/	/	/	/	/	/		0.00	0.22	0.20	0.36	0.75	0.22	0.22	0.73	0.22	/
5F5H	/	/	/	/	/	/	/	/	/	/		0.00	0.15	1.36	0.15	0.50	0.15	0.20	0.33	0.15	0.15
5L1Z	/	/	/	/	/	/	/	/	/	/		0.67	1.29	2.50	1.29	1.43	0.40	0.40	2.78	0.83	1.57
2FY1	/	/	/	/	/	/	/	/	/	/		0.00	0.13	0.13	0.13	0.13	/	/	/	/	/
1RKJ	/	/	/	/	/	/	/	/	/	/		0.13	0.00	0.23	0.29	0.29	0.14	0.31	0.15	0.29	0.43
1QWA	/	/	/	/	/	/	/	/	/	/		0.00	0.13	0.57	0.40	0.13	0.20	0.57	0.29	0.40	0.60
1SZY	/	/	/	/	/	/	/	/	/	/		0.00	0.20	0.27	0.15	0.15	0.36	0.43	0.15	/	/
2M21	/	/	/	/	/	/	/	/	/	/		0.00	0.15	0.27	0.15	0.20	0.50	0.14	0.33	0.43	0.15
1JOX	/	/	/	/	/	/	/	/	/	/		0.00	0.15	0.50	0.20	0.23	0.15	0.15	1.09	0.20	/
17RA	/	/	/	/	/	/	/	/	/	/		0.13	0.13	0.36	0.29	0.29	0.00	0.29	0.29	0.29	0.77
1D0U	/	/	/	/	/	/	/	/	/	/		0.00	0.13	0.43	0.13	0.57	0.13	0.20	0.13	0.13	0.20
2MFF	/	/	/	/	/	/	/	/	/	/		0.00	0.13	0.13	0.13	0.13	/	/	/	/	/
2MFG	/	/	/	/	/	/	/	/	/	/		0.00	0.13	0.13	0.13	0.13	/	/	/	/	/
6XWJ	/	/	/	/	/	/	/	/	/	/		0.00	0.12	0.38	0.12	0.25	0.18	0.80	0.18	0.12	0.53
1K2G	/	/	/	/	/	/	/	/	/	/		4.78	9.30	0.27	9.56	8.67	/	4.00	0.50	8.90	9.56
2W2H	/	/	/	/	/	/	/	/	/	/		0.71	0.62	0.83	0.46	0.92	0.33	0.79	0.93	0.92	0.91
4A4S	/	/	/	/	/	/	/	/	/	/		0.00	0.12	0.12	0.12	/	/	/	/	/	/
2GRW	/	/	/	/	/	/	/	/	/	/		0.00	0.13	0.13	0.13	1.00	0.13	0.13	0.29	0.13	/
2GV4	/	/	/	/	/	/	/	/	/	/		0.00	0.13	0.13	0.13	0.13	0.13	/	/	/	/
1N66	/	/	/	/	/	/	/	/	/	/		0.00	0.15	0.93	0.86	0.15	0.15	0.15	0.86	1.14	0.15
1OSW	/	/	/	/	/	/	/	/	/	/		0.00	0.29	0.77	0.15	0.15	1.17	0.15	0.46	0.46	0.36
2K66	/	/	/	/	/	/	/	/	/	/		0.00	0.12	0.12	0.12	0.12	0.25	0.12	0.12	/	/

	DNA model											RNA model									
PDB	RNAfold MFE	subopt #2	subopt #3	subopt #4	subopt #5	subopt #6	subopt #7	subopt #8	subopt #9	subopt #10		RNAfold MFE	subopt #2	subopt #3	subopt #4	subopt #5	subopt #6	subopt #7	subopt #8	subopt #9	subopt #10
2JSE	/	/	/	/	/	/	/	/	/	/		0.00	0.15	0.33	0.20	0.20	0.15	0.15	0.57	0.14	0.15
2KD8	/	/	/	/	/	/	/	/	/	/		0.00	0.12	0.38	0.12	0.25	0.12	0.53	0.12	0.80	0.12
2M5U	/	/	/	/	/	/	/	/	/	/		0.00	0.12	0.12	0.12	0.12	0.12	0.12	/	/	/
2GV3	/	/	/	/	/	/	/	/	/	/		0.20	0.13	0.50	0.38	0.36	0.00	0.53	0.27	0.25	0.27
1FJE	/	/	/	/	/	/	/	/	/	/		0.00	1.40	1.00	0.29	1.00	0.44	1.11	0.56	0.89	0.89
6F4H	/	/	/	/	/	/	/	/	/	/		0.00	0.18	0.23	0.54	0.46	0.38	0.18	0.42	0.75	/
1JUR	/	/	/	/	/	/	/	/	/	/		0.00	0.13	0.13	1.38	0.13	0.13	0.43	0.29	0.13	0.13
1PJY	/	/	/	/	/	/	/	/	/	/		0.00	0.12	0.12	0.12	0.12	/	/	/	/	/
1F9L	/	/	/	/	/	/	/	/	/	/		0.00	0.15	0.15	0.15	0.15	0.15	/	/	/	/
1K6G	/	/	/	/	/	/	/	/	/	/		0.00	0.12	0.12	0.12	0.12	0.38	0.12	/	/	/
2HNS	/	/	/	/	/	/	/	/	/	/		0.00	0.12	0.12	0.12	0.12	0.12	0.38	/	/	/
1K6H	/	/	/	/	/	/	/	/	/	/		0.00	0.12	0.12	0.12	0.12	0.12	0.38	/	/	/
2JYM	/	/	/	/	/	/	/	/	/	/		0.18	0.31	0.44	1.79	0.60	0.31	0.60	0.31	0.87	1.31
2MFE	/	/	/	/	/	/	/	/	/	/		0.00	0.13	0.13	0.13	0.33	0.13	0.43	0.13	/	/
1IKD	/	/	/	/	/	/	/	/	/	/		0.00	0.15	0.15	0.15	0.38	0.15	/	/	/	/
2G1W	/	/	/	/	/	/	/	/	/	/		3.55	4.91	4.00	4.67	5.82	4.55	4.90	4.10	4.00	4.10
2MFC	/	/	/	/	/	/	/	/	/	/		0.00	0.15	0.27	0.15	/	/	/	/	/	/
6KYV	/	/	/	/	/	/	/	/	/	/		0.00	0.12	0.12	0.12	0.12	0.12	0.12	/	/	/
1TJZ	/	/	/	/	/	/	/	/	/	/		0.00	0.57	0.33	0.20	0.15	1.00	0.15	0.15	0.50	1.50
2ANN	/	/	/	/	/	/	/	/	/	/		2.00	2.00	1.50	1.25	3.13	0.00	2.50	3.25	3.00	3.00
2N0R	/	/	/	/	/	/	/	/	/	/		1.22	0.60	1.10	1.10	1.18	0.91	1.88	1.00	0.60	1.10
1JTJ	/	/	/	/	/	/	/	/	/	/		0.00	0.15	0.33	0.15	0.50	0.50	1.09	0.50	0.15	0.50
1OW9	/	/	/	/	/	/	/	/	/	/		0.00	0.15	0.50	0.15	0.15	0.15	0.15	/	/	/
2N2P	/	/	/	/	/	/	/	/	/	/		0.93	0.15	0.00	0.86	0.50	0.38	1.14	0.33	0.18	1.08
2N2O	/	/	/	/	/	/	/	/	/	/		0.93	0.15	0.00	0.86	0.50	7.00	0.38	1.14	0.50	0.57
5UF3	/	/	/	/	/	/	/	/	/	/		0.00	0.11	0.18	0.24	0.12	0.24	0.12	0.12	0.24	0.50
5WQ1	/	/	/	/	/	/	/	/	/	/		0.00	0.12	0.12	0.11	0.12	0.12	0.12	0.38	/	/
1K5I	/	/	/	/	/	/	/	/	/	/		0.00	0.12	0.29	0.12	0.12	/	/	/	/	/
2ES5	/	/	/	/	/	/	/	/	/	/		0.16	0.39	0.28	0.28	0.28	0.53	0.28	0.28	/	/
2QH3	/	/	/	/	/	/	/	/	/	/		0.00	0.13	0.18	0.24	0.13	0.13	0.31	0.13	/	/
2M22	/	/	/	/	/	/	/	/	/	/		0.12	0.00	0.13	0.25	0.25	0.13	0.13	0.25	0.13	1.38
6GBM	/	/	/	/	/	/	/	/	/	/		0.20	0.50	0.00	0.13	0.36	0.25	1.00	0.27	0.25	0.27
1TLR	/	/	/	/	/	/	/	/	/	/		0.00	0.20	1.07	0.15	2.45	0.36	0.15	0.47	0.20	0.92
1BVJ	/	/	/	/	/	/	/	/	/	/		0.00	0.13	0.13	0.13	0.13	1.15	0.43	0.13	0.13	0.57
2PJP	/	/	/	/	/	/	/	/	/	/		0.21	0.11	0.44	0.33	0.33	0.22	0.22	0.59	0.47	0.47
1MFK	/	/	/	/	/	/	/	/	/	/		0.00	0.11	0.12	0.24	0.12	0.24	0.12	0.25	0.38	0.25
2UWM	/	/	/	/	/	/	/	/	/	/		0.28	0.21	0.41	0.33	0.53	0.41	0.18	0.33	0.11	/
2N3O	/	/	/	/	/	/	/	/	/	/		0.00	0.12	0.12	0.12	/	/	/	/	/	/
2RO2	/	/	/	/	/	/	/	/	/	/		0.00	0.11	0.12	0.12	0.22	0.22	0.29	0.18	0.12	0.25
1BGZ	/	/	/	/	/	/	/	/	/	/		1.53	1.76	0.38	1.63	1.44	1.44	1.69	0.47	2.00	0.33
1S2F	/	/	/	/	/	/	/	/	/	/		0.00	0.12	0.38	0.12	1.13	0.65	1.00	1.20	0.38	0.75
2M12	/	/	/	/	/	/	/	/	/	/		0.31	0.38	0.20	0.29	1.00	0.43	0.53	0.80	0.36	0.67
2N82	/	/	/	/	/	/	/	/	/	/		1.45	0.22	1.27	2.17	1.45	1.82	0.80	0.90	0.90	1.45
2N7X	/	/	/	/	/	/	/	/	/	/		0.00	1.17	0.29	0.13	1.14	1.14	0.15	0.69	0.23	0.86
3PHP	/	/	/	/	/	/	/	/	/	/		0.00	0.13	0.18	0.13	0.13	0.13	/	/	/	/
5NG6	/	/	/	/	/	/	/	/	/	/		0.00	0.22	2.00	1.38	0.36	1.85	2.29	1.17	0.83	0.27
3NVK	/	/	/	/	/	/	/	/	/	/		2.38	6.17	2.22	1.83	2.67	6.83	2.60	3.25	2.00	2.29
1A9N	/	/	/	/	/	/	/	/	/	/		0.00	2.46	1.62	2.00	1.50	0.73	5.40	0.64	0.73	1.92

	DNA model											RNA model									
**PDB**	**RNAfold MFE**	**subopt #2**	**subopt #3**	**subopt #4**	**subopt #5**	**subopt #6**	**subopt #7**	**subopt #8**	**subopt #9**	**subopt #10**		**RNAfold MFE**	**subopt #2**	**subopt #3**	**subopt #4**	**subopt #5**	**subopt #6**	**subopt #7**	**subopt #8**	**subopt #9**	**subopt #10**
5F9F	/	/	/	/	/	/	/	/	/	/		0.00	0.11	0.11	0.33	0.33	0.11	0.11	0.11	0.22	0.11
1KKS	/	/	/	/	/	/	/	/	/	/		0.13	0.20	0.46	0.29	0.36	0.67	0.00	0.15	0.29	0.14
2QH2	/	/	/	/	/	/	/	/	/	/		0.18	0.31	0.00	0.22	0.33	0.11	0.22	0.31	0.31	/
1MT4	/	/	/	/	/	/	/	/	/	/		0.00	0.13	0.24	0.44	0.18	0.13	0.24	0.13	0.29	0.29
2LK3	/	/	/	/	/	/	/	/	/	/		0.00	0.12	0.12	0.11	0.12	0.25	0.12	0.25	0.24	0.25
2HEM	/	/	/	/	/	/	/	/	/	/		0.00	0.13	0.33	0.13	0.13	0.13	0.13	/	/	/
1TFN	/	/	/	/	/	/	/	/	/	/		0.13	0.00	0.29	0.77	0.15	0.33	0.20	0.29	0.67	0.44
1RHT	/	/	/	/	/	/	/	/	/	/		0.13	0.29	0.29	0.77	0.46	0.40	0.27	0.29	1.00	0.31
2LV0	/	/	/	/	/	/	/	/	/	/		0.00	0.13	0.24	0.13	0.71	0.44	0.24	0.13	0.47	0.13
5UDZ	/	/	/	/	/	/	/	/	/	/		0.15	0.33	0.00	0.18	0.18	0.38	0.23	0.33	1.36	0.17
1NYB	/	/	/	/	/	/	/	/	/	/		0.00	0.12	0.11	0.12	0.25	0.24	0.12	0.12	0.22	0.33
1NC0	/	/	/	/	/	/	/	/	/	/		0.12	0.25	0.00	0.25	0.25	0.25	0.18	/	/	/
1SYZ	/	/	/	/	/	/	/	/	/	/		0.00	0.13	0.33	0.47	0.13	0.13	0.13	0.25	0.13	0.13
1E4P	/	/	/	/	/	/	/	/	/	/		0.00	0.15	0.50	1.08	0.14	0.15	1.29	0.15	0.15	0.27
6F4G	/	/	/	/	/	/	/	/	/	/		0.00	3.43	1.00	0.46	0.46	0.86	0.38	0.18	0.38	3.15
1M82	/	/	/	/	/	/	/	/	/	/		0.11	0.11	0.22	0.22	0.21	0.32	0.00	0.22	0.22	0.50
1QC8	/	/	/	/	/	/	/	/	/	/		0.22	0.12	0.80	0.35	0.71	0.25	0.25	0.39	0.28	0.38
6DU5	/	/	/	/	/	/	/	/	/	/		4.91	5.09	5.00	4.60	4.00	4.40	4.00	0.22	5.00	4.33
4QOZ	/	/	/	/	/	/	/	/	/	/		0.00	0.18	0.18	0.17	0.60	0.40	0.18	/	/	/
2L5Z	/	/	/	/	/	/	/	/	/	/		0.00	0.59	0.12	0.12	1.00	0.78	0.75	0.72	0.28	0.38
1QWB	/	/	/	/	/	/	/	/	/	/		0.00	1.00	0.69	0.18	0.69	0.31	0.77	0.38	0.62	0.62
4TV0	/	/	/	/	/	/	/	/	/	/		0.00	0.18	1.29	1.54	0.18	0.17	0.60	0.40	0.62	/
4BW0	/	/	/	/	/	/	/	/	/	/		0.00	0.50	1.00	0.45	0.18	0.17	0.33	0.18	0.18	0.64
6XH0	/	/	/	/	/	/	/	/	/	/		0.16	0.00	0.28	0.28	0.20	0.30	0.39	0.11	0.28	0.11
1FQZ	/	/	/	/	/	/	/	/	/	/		0.00	12.23	0.23	12.58	0.18	0.31	11.70	0.18	0.23	11.46
1XSG	/	/	/	/	/	/	/	/	/	/		0.00	0.10	0.10	0.10	0.10	0.10	/	/	/	/
1XSH	/	/	/	/	/	/	/	/	/	/		0.00	0.09	0.10	0.09	0.10	0.10	0.10	0.10	0.19	0.19
1F7F	/	/	/	/	/	/	/	/	/	/		0.00	0.10	0.18	0.09	0.10	0.10	0.10	0.10	0.29	0.19
2LQZ	/	/	/	/	/	/	/	/	/	/		0.11	0.22	0.00	0.22	2.18	0.12	0.47	0.12	0.22	0.28
2AHT	/	/	/	/	/	/	/	/	/	/		0.10	0.00	0.20	0.20	0.20	0.11	0.11	0.11	0.32	0.47
2LDL	/	/	/	/	/	/	/	/	/	/		0.11	0.00	0.22	0.22	0.12	0.12	0.16	1.20	0.22	0.47
2LJJ	/	/	/	/	/	/	/	/	/	/		0.11	0.00	0.22	0.22	0.22	0.12	0.21	0.47	0.12	0.35
2IXY	/	/	/	/	/	/	/	/	/	/		0.14	0.09	0.00	0.25	0.25	0.35	0.19	0.10	0.19	0.19
1FYO	/	/	/	/	/	/	/	/	/	/		0.00	0.24	0.11	0.12	0.38	0.12	1.05	0.24	0.44	2.28
1YSV	/	/	/	/	/	/	/	/	/	/		0.00	0.10	0.10	0.09	0.10	0.30	0.10	0.10	0.10	0.10
484D	/	/	/	/	/	/	/	/	/	/		0.00	0.22	0.33	1.26	0.63	0.12	1.58	0.35	1.06	0.29
5M0I	/	/	/	/	/	/	/	/	/	/		0.73	2.00	1.62	0.93	0.15	0.64	2.00	1.21	13.67	0.43
2NCI	/	/	/	/	/	/	/	/	/	/		0.22	0.35	0.12	0.12	0.28	0.28	0.39	0.44	0.35	0.00
2KMJ	/	/	/	/	/	/	/	/	/	/		0.00	0.10	0.10	0.20	0.10	0.30	0.10	0.20	0.42	0.14
2GIP	/	/	/	/	/	/	/	/	/	/		0.00	1.05	1.24	0.11	0.11	1.00	1.20	1.20	0.11	1.00
28SP	/	/	/	/	/	/	/	/	/	/		0.00	0.29	0.13	0.13	0.41	0.13	0.13	0.41	0.47	0.53
1ZBN	/	/	/	/	/	/	/	/	/	/		0.00	0.10	0.14	0.25	0.10	0.10	0.20	0.20	0.25	0.25
6SNJ	/	/	/	/	/	/	/	/	/	/		0.14	0.00	0.25	0.70	0.25	0.14	0.24	0.33	0.43	0.11
2LUN	/	/	/	/	/	/	/	/	/	/		0.00	0.24	0.12	0.53	0.37	0.26	0.12	0.74	0.38	0.32
2NC0	/	/	/	/	/	/	/	/	/	/		0.00	0.11	0.11	0.11	0.20	0.10	0.11	0.20	0.22	0.11
6VZC	/	/	/	/	/	/	/	/	/	/		0.00	0.11	0.11	0.24	0.14	0.11	0.11	0.11	0.14	0.33
6AAS	/	/	/	/	/	/	/	/	/	/		0.00	0.09	0.09	0.09	0.09	0.09	0.09	0.09	0.18	0.09
1OOA	/	/	/	/	/	/	/	/	/	/		0.00	0.21	7.47	0.42	0.32	0.12	0.12	0.72	0.12	0.21

	DNA model											RNA model									
PDB	RNAfold MFE	subopt #2	subopt #3	subopt #4	subopt #5	subopt #6	subopt #7	subopt #8	subopt #9	subopt #10		RNAfold MFE	subopt #2	subopt #3	subopt #4	subopt #5	subopt #6	subopt #7	subopt #8	subopt #9	subopt #10
5LSN	/	/	/	/	/	/	/	/	/	/		0.00	0.09	0.09	0.27	0.18	0.09	0.45	0.13	0.18	0.57
1JBT	/	/	/	/	/	/	/	/	/	/		0.81	0.69	0.00	1.00	1.00	1.07	0.87	1.13	0.15	1.25
5LM7	/	/	/	/	/	/	/	/	/	/		0.00	5.77	0.22	0.22	4.17	4.33	4.17	0.20	6.67	2.45
2JWV	/	/	/	/	/	/	/	/	/	/		0.00	0.21	0.42	0.32	0.12	0.72	0.12	0.12	0.21	0.63
3SN2	/	/	/	/	/	/	/	/	/	/		0.13	0.08	0.00	0.23	0.32	0.23	0.17	0.17	0.09	0.43
2M24	/	/	/	/	/	/	/	/	/	/		0.00	0.40	0.21	0.71	0.42	0.53	0.12	0.21	0.60	0.12
1NBR	/	/	/	/	/	/	/	/	/	/		0.13	0.23	0.23	0.32	0.23	0.38	0.23	0.00	0.23	0.41
1L1C	/	/	/	/	/	/	/	/	/	/		0.16	0.00	0.67	1.06	0.61	0.89	4.29	0.28	0.11	0.11
1ANR	/	/	/	/	/	/	/	/	/	/		0.11	0.25	0.22	0.00	0.25	0.35	0.22	0.37	0.22	0.16
2GIO	/	/	/	/	/	/	/	/	/	/		0.00	0.24	1.42	0.16	1.63	0.11	0.12	1.61	1.33	1.25
1F84	/	/	/	/	/	/	/	/	/	/		0.15	0.36	0.00	0.43	11.36	0.33	11.38	11.42	0.36	1.08
1SCL	/	/	/	/	/	/	/	/	/	/		0.81	0.69	0.00	1.00	1.07	1.13	0.53	1.00	1.80	1.40
2K5Z	/	/	/	/	/	/	/	/	/	/		0.00	0.10	0.10	0.10	0.10	0.86	0.09	0.18	0.50	0.20
1L1W	/	/	/	/	/	/	/	/	/	/		0.00	0.20	0.11	0.11	0.11	0.11	0.11	0.70	0.30	0.11
1EBS	/	/	/	/	/	/	/	/	/	/		0.00	0.14	0.14	0.24	0.10	0.11	0.25	0.25	0.11	0.11
6DU4	/	/	/	/	/	/	/	/	/	/		0.00	1.25	0.73	0.73	0.60	1.76	0.15	0.27	0.80	1.47
1HVU	/	/	/	/	/	/	/	/	/	/		6.53	4.00	4.40	7.14	7.69	8.71	6.38	7.14	5.00	5.76
1LDZ	/	/	/	/	/	/	/	/	/	/		0.00	0.14	0.11	0.10	0.11	0.25	0.11	0.11	0.33	0.11
1EKZ	/	/	/	/	/	/	/	/	/	/		0.07	0.15	0.23	0.15	0.15	/	/	/	/	/
5Y58	/	/	/	/	/	/	/	/	/	/		0.00	0.20	0.20	0.11	0.11	0.32	2.10	0.25	1.29	0.11
1RFR	/	/	/	/	/	/	/	/	/	/		0.00	0.11	0.11	0.33	0.22	0.33	0.10	0.11	0.47	0.11
1AUD	/	/	/	/	/	/	/	/	/	/		3.18	2.89	0.00	3.13	0.16	2.82	2.56	2.44	0.21	0.53
1KP7	/	/	/	/	/	/	/	/	/	/		0.00	0.16	0.63	0.16	0.12	0.12	0.72	4.38	0.42	0.59
6MCE	/	/	/	/	/	/	/	/	/	/		0.13	0.23	0.00	0.23	0.23	0.09	0.17	0.25	0.32	0.33
1NA2	/	/	/	/	/	/	/	/	/	/		0.00	0.13	1.57	0.13	0.50	0.25	0.50	0.13	0.75	0.13
1HWQ	/	/	/	/	/	/	/	/	/	/		0.11	0.00	0.22	0.21	0.32	0.42	0.22	0.22	0.30	0.12
1EBR	/	/	/	/	/	/	/	/	/	/		0.00	0.14	0.14	0.24	0.40	0.10	0.11	0.25	0.25	0.26
5KMZ	/	/	/	/	/	/	/	/	/	/		4.26	8.76	4.61	9.44	4.61	4.61	9.67	5.00	5.00	5.89
1MFY	/	/	/	/	/	/	/	/	/	/		0.30	0.18	0.11	0.52	0.43	0.42	0.42	0.44	0.33	0.31
1JO7	/	/	/	/	/	/	/	/	/	/		0.18	0.18	0.33	0.29	0.29	0.45	0.45	0.29	0.29	0.58
1YNC	/	/	/	/	/	/	/	/	/	/		0.10	0.43	0.19	0.52	0.23	0.32	0.00	0.36	0.14	1.15
1YNG	/	/	/	/	/	/	/	/	/	/		0.20	0.30	0.11	0.43	0.33	0.11	0.52	0.43	0.55	0.32
6HYK	/	/	/	/	/	/	/	/	/	/		0.00	0.16	0.35	0.26	0.12	0.45	0.21	0.21	0.28	0.26
2LDT	/	/	/	/	/	/	/	/	/	/		0.00	0.10	0.10	0.10	0.20	0.09	0.30	0.14	0.20	0.19
5UZT	/	/	/	/	/	/	/	/	/	/		1.00	1.05	0.30	1.00	0.62	0.67	0.60	0.47	0.67	1.53
5A18	/	/	/	/	/	/	/	/	/	/		0.09	0.00	0.52	0.50	1.64	0.70	0.38	0.29	1.05	0.45
1XHP	/	/	/	/	/	/	/	/	/	/		0.00	0.10	0.24	0.30	0.33	0.10	0.10	0.10	0.10	1.56
1Z31	/	/	/	/	/	/	/	/	/	/		0.00	1.09	0.10	0.10	0.95	1.24	13.38	0.10	0.09	0.10
2LI4	/	/	/	/	/	/	/	/	/	/		0.00	0.07	0.07	0.23	0.15	0.07	0.07	0.07	0.07	0.07
1KAJ	/	/	/	/	/	/	/	/	/	/		5.50	8.41	5.71	7.67	8.44	8.50	8.56	8.22	7.74	7.79
1KPD	/	/	/	/	/	/	/	/	/	/		6.35	8.56	6.67	7.74	8.58	8.63	8.68	8.16	8.37	7.80
2LBS	/	/	/	/	/	/	/	/	/	/		0.00	0.07	0.23	0.07	0.07	0.07	0.11	0.07	0.07	0.07
2LUP	/	/	/	/	/	/	/	/	/	/		0.00	0.07	0.23	0.07	0.07	0.07	0.11	0.07	0.07	0.07
1G70	/	/	/	/	/	/	/	/	/	/		0.00	0.13	0.13	0.22	0.36	0.09	0.10	0.23	0.23	0.24
2JXV	/	/	/	/	/	/	/	/	/	/		0.00	0.12	0.09	0.12	0.12	0.21	0.09	0.09	0.21	0.28
1EXY	/	/	/	/	/	/	/	/	/	/		0.00	0.17	0.17	0.09	0.09	0.33	0.09	0.26	0.09	0.26
3ID5	/	/	/	/	/	/	/	/	/	/		8.92	17.00	10.00	17.10	16.60	8.90	11.80	11.89	8.82	16.73
4OOG	/	/	/	/	/	/	/	/	/	/		0.00	0.07	0.07	0.07	0.07	0.07	0.07	0.07	0.07	0.23

	DNA model											RNA model									
PDB	RNAfold MFE	subopt #2	subopt #3	subopt #4	subopt #5	subopt #6	subopt #7	subopt #8	subopt #9	subopt #10		RNAfold MFE	subopt #2	subopt #3	subopt #4	subopt #5	subopt #6	subopt #7	subopt #8	subopt #9	subopt #10
1R2P	/	/	/	/	/	/	/	/	/	/		0.15	0.23	0.08	0.08	0.38	0.24	0.32	0.24	0.32	0.42
2F88	/	/	/	/	/	/	/	/	/	/		0.07	0.07	0.00	0.15	0.15	0.15	0.11	0.15	0.32	0.15
1R7W	/	/	/	/	/	/	/	/	/	/		0.00	0.17	0.09	0.21	0.09	0.09	0.42	0.42	0.26	0.09
1R7Z	/	/	/	/	/	/	/	/	/	/		0.00	0.17	0.09	0.21	0.09	0.09	0.42	0.26	0.09	0.21
2KPV	/	/	/	/	/	/	/	/	/	/		0.12	0.00	0.21	0.21	0.08	0.21	0.21	0.08	0.25	0.21
2JTP	/	/	/	/	/	/	/	/	/	/		0.00	0.67	0.08	0.08	0.24	0.08	0.96	0.44	0.25	0.08
1P5N	/	/	/	/	/	/	/	/	/	/		0.11	16.65	0.00	0.45	16.68	0.25	0.22	0.30	16.42	0.37
6SDY	/	/	/	/	/	/	/	/	/	/		0.00	0.07	0.07	0.07	0.07	0.07	0.07	0.07	0.07	/
4X4O	/	/	/	/	/	/	/	/	/	/		0.00	0.65	1.00	0.16	0.09	0.12	0.55	0.09	0.09	0.79
2EUY	/	/	/	/	/	/	/	/	/	/		0.13	0.00	0.23	0.23	0.10	0.29	0.23	0.33	0.43	0.23
1RNK	/	/	/	/	/	/	/	/	/	/		7.44	7.88	8.58	5.94	7.70	5.20	8.15	8.60	8.65	8.70
2RVO	/	/	/	/	/	/	/	/	/	/		0.00	0.08	0.92	0.08	1.04	0.25	0.08	1.13	0.87	0.17
2L3C	/	/	/	/	/	/	/	/	/	/		0.00	0.07	0.23	0.07	0.15	0.07	0.07	0.15	0.15	0.48
1T28	/	/	/	/	/	/	/	/	/	/		0.11	0.45	0.50	0.40	0.35	0.70	0.00	0.22	0.60	0.12
1ETF	/	/	/	/	/	/	/	/	/	/		0.00	0.12	0.12	0.20	0.33	0.08	0.09	0.21	0.21	0.22
4C4W	/	/	/	/	/	/	/	/	/	/		16.69	16.18	14.94	16.87	16.33	14.83	12.60	6.60	16.31	16.59
5FJ4	/	/	/	/	/	/	/	/	/	/		11.06	6.39	6.53	9.50	10.94	6.61	11.47	11.67	9.00	9.93
2PCV	/	/	/	/	/	/	/	/	/	/		0.16	0.35	0.40	0.00	0.50	0.60	0.28	0.28	0.57	0.67
2M57	/	/	/	/	/	/	/	/	/	/		0.26	0.35	0.36	0.45	0.18	0.18	0.27	0.18	0.90	0.29
2DRB	/	/	/	/	/	/	/	/	/	/		0.00	0.09	0.09	0.09	0.16	/	/	/	/	/
1ULL	/	/	/	/	/	/	/	/	/	/		0.00	0.43	0.09	0.08	0.09	0.09	0.09	0.17	0.30	0.27
2L3E	/	/	/	/	/	/	/	/	/	/		0.00	0.08	0.08	0.15	0.08	0.50	0.25	0.08	0.08	0.19
6BHJ	/	/	/	/	/	/	/	/	/	/		0.07	0.14	0.14	0.14	0.14	0.14	0.22	/	/	/
6SY6	/	/	/	/	/	/	/	/	/	/		0.18	1.79	5.50	1.23	0.92	1.54	15.40	2.27	1.38	0.00
2TPK	/	/	/	/	/	/	/	/	/	/		5.20	5.00	5.26	4.48	5.05	5.58	4.90	4.76	5.33	5.58
2N6S	/	/	/	/	/	/	/	/	/	/		0.10	0.00	0.14	0.17	0.07	0.17	0.07	0.07	0.29	0.17
1N8X	/	/	/	/	/	/	/	/	/	/		0.00	0.29	0.43	0.14	0.37	0.22	0.07	0.37	0.07	0.07
4X4P	/	/	/	/	/	/	/	/	/	/		0.00	0.12	0.16	0.09	0.65	1.00	0.09	0.09	0.09	0.55
2HW8	/	/	/	/	/	/	/	/	/	/		0.00	0.16	0.65	0.09	2.24	0.25	2.00	0.09	0.09	0.25
5KQE	/	/	/	/	/	/	/	/	/	/		0.19	0.28	0.12	0.00	0.28	0.28	0.36	0.21	0.28	0.28
2FDT	/	/	/	/	/	/	/	/	/	/		0.07	0.19	0.15	0.15	0.15	0.00	0.32	0.15	0.15	0.08
2LUB	/	/	/	/	/	/	/	/	/	/		0.00	0.07	0.07	0.07	0.07	0.07	0.07	0.07	0.21	0.07
2LHP	/	/	/	/	/	/	/	/	/	/		0.00	0.07	0.07	0.21	0.07	0.07	0.07	0.07	0.07	0.41
6U79	/	/	/	/	/	/	/	/	/	/		0.00	0.07	0.07	0.07	0.07	0.76	0.21	0.07	0.07	0.07
6DTD	/	/	/	/	/	/	/	/	/	/		0.35	0.26	0.17	0.32	0.17	0.08	0.00	1.39	1.30	1.22
1M5L	/	/	/	/	/	/	/	/	/	/		0.00	1.08	0.09	0.09	0.08	0.70	1.44	1.00	0.09	0.17
2KHY	/	/	/	/	/	/	/	/	/	/		0.11	0.22	0.50	0.30	0.67	0.95	0.24	0.63	0.42	0.22
4PDB	/	/	/	/	/	/	/	/	/	/		0.29	0.30	0.37	0.07	0.37	0.52	0.45	0.30	0.38	0.21
1TXS	/	/	/	/	/	/	/	/	/	/		0.00	0.08	0.08	0.25	0.08	0.08	0.17	0.08	0.56	0.08
6D12	/	/	/	/	/	/	/	/	/	/		0.23	0.08	18.54	18.57	18.57	18.61	19.00	0.00	0.32	0.31
2A9L	/	/	/	/	/	/	/	/	/	/		0.00	0.07	0.07	1.00	0.14	0.07	0.07	0.07	0.54	0.14
1B36	/	/	/	/	/	/	/	/	/	/		0.00	0.70	1.00	0.40	0.60	0.91	3.00	19.29	0.32	0.89
4KR7	/	/	/	/	/	/	/	/	/	/		0.17	3.08	0.00	2.83	0.35	2.88	2.74	0.09	3.29	0.21
4KR9	/	/	/	/	/	/	/	/	/	/		0.84	3.31	0.60	3.08	1.08	3.12	3.00	0.71	3.52	0.88
2MXL	/	/	/	/	/	/	/	/	/	/		0.00	0.08	0.25	0.08	0.52	0.11	0.08	0.17	0.08	0.91
2NBY	/	/	/	/	/	/	/	/	/	/		0.29	0.79	0.10	0.37	0.00	0.23	0.27	0.59	0.79	0.63
2HUA	/	/	/	/	/	/	/	/	/	/		0.17	0.12	0.38	0.00	0.70	0.39	0.48	0.08	0.58	0.95
4PMI	/	/	/	/	/	/	/	/	/	/		0.44	0.54	0.00	0.54	0.11	1.58	0.08	0.65	0.19	1.72

	DNA model											RNA model									
PDB	RNAfold MFE	subopt #2	subopt #3	subopt #4	subopt #5	subopt #6	subopt #7	subopt #8	subopt #9	subopt #10		RNAfold MFE	subopt #2	subopt #3	subopt #4	subopt #5	subopt #6	subopt #7	subopt #8	subopt #9	subopt #10
2NBZ	/	/	/	/	/	/	/	/	/	/		0.00	0.06	0.07	0.10	0.17	0.13	0.07	0.23	0.17	0.07
5W1H	/	/	/	/	/	/	/	/	/	/		10.08	0.44	8.79	0.55	7.45	7.33	9.31	0.40	9.31	0.40
4M6D	/	/	/	/	/	/	/	/	/	/		10.26	10.10	1.63	2.39	1.35	10.20	10.17	1.10	2.53	3.44
5V17	/	/	/	/	/	/	/	/	/	/		0.00	0.13	0.07	0.07	0.07	0.21	0.07	0.07	0.07	0.07
1A51	/	/	/	/	/	/	/	/	/	/		0.38	0.00	0.52	0.24	0.16	0.28	0.48	0.09	0.48	0.09
1ZC5	/	/	/	/	/	/	/	/	/	/		0.00	0.12	0.06	0.18	0.06	0.34	0.06	0.21	0.18	0.18
6W3M	/	/	/	/	/	/	/	/	/	/		0.00	0.07	0.07	0.07	0.07	0.07	0.07	0.21	0.07	0.21
5WLH	/	/	/	/	/	/	/	/	/	/		10.08	0.44	8.79	0.55	1.33	7.45	7.33	1.40	9.31	0.40
2N6T	/	/	/	/	/	/	/	/	/	/		0.24	0.21	0.24	0.18	0.14	0.18	0.56	0.32	0.29	0.32
1MNX	/	/	/	/	/	/	/	/	/	/		0.00	0.16	0.09	0.12	0.16	0.09	0.09	0.27	0.20	0.25
2L2J	/	/	/	/	/	/	/	/	/	/		0.00	0.06	0.06	0.06	0.13	0.06	0.13	0.06	0.18	0.12
2FEY	/	/	/	/	/	/	/	/	/	/		0.54	0.59	0.24	0.24	0.31	0.63	0.44	0.69	0.50	0.59
1CQ5	/	/	/	/	/	/	/	/	/	/		0.25	0.09	0.22	0.00	0.48	0.44	0.35	0.35	0.52	0.33
1CQL	/	/	/	/	/	/	/	/	/	/		0.08	0.00	0.21	0.09	0.58	0.27	0.17	0.17	0.35	0.16
2ADT	/	/	/	/	/	/	/	/	/	/		0.00	0.09	0.06	0.06	0.15	0.31	0.13	0.09	0.20	0.15
2N6X	/	/	/	/	/	/	/	/	/	/		0.33	0.36	0.42	0.06	0.22	0.25	0.28	0.31	0.41	0.44
1A60	/	/	/	/	/	/	/	/	/	/		1.80	1.96	4.08	2.22	2.60	1.96	1.96	2.79	2.13	2.40
1P6V	/	/	/	/	/	/	/	/	/	/		0.00	0.20	0.38	0.56	0.09	0.09	0.16	1.44	0.74	0.40
1Z2J	/	/	/	/	/	/	/	/	/	/		0.00	0.11	0.05	0.05	0.16	0.19	0.16	0.11	0.26	0.21
2PXL	/	/	/	/	/	/	/	/	/	/		0.00	0.07	0.07	9.04	0.15	10.04	8.04	8.59	0.30	0.23
2MTJ	/	/	/	/	/	/	/	/	/	/		0.13	0.44	0.07	0.32	0.29	0.21	0.39	0.28	0.39	0.14
1S03	/	/	/	/	/	/	/	/	/	/		0.19	0.26	0.14	0.11	0.05	0.39	0.33	0.22	0.16	0.21
5KH8	/	/	/	/	/	/	/	/	/	/		5.57	5.73	4.71	6.42	3.28	3.92	7.04	6.45	3.67	4.08
1YMO	/	/	/	/	/	/	/	/	/	/		13.09	5.00	7.75	5.27	7.84	8.17	5.07	13.23	13.29	7.48
2VPL	/	/	/	/	/	/	/	/	/	/		0.12	0.06	0.06	0.00	0.13	0.19	0.13	0.13	0.19	0.13
2KUV	/	/	/	/	/	/	/	/	/	/		0.11	0.05	0.00	0.16	0.11	0.05	0.16	0.11	0.05	0.11
2KUU	/	/	/	/	/	/	/	/	/	/		0.11	0.05	0.00	0.16	0.11	0.05	0.11	0.05	0.16	0.11
2KUR	/	/	/	/	/	/	/	/	/	/		0.11	0.05	0.00	0.16	0.11	0.05	0.16	0.11	0.05	0.11
2KE6	/	/	/	/	/	/	/	/	/	/		0.16	0.11	0.05	0.22	0.17	0.11	0.22	0.17	0.11	0.17
2K95	/	/	/	/	/	/	/	/	/	/		13.35	8.23	7.78	13.50	13.57	7.96	8.40	12.88	6.25	8.16
2KUW	/	/	/	/	/	/	/	/	/	/		0.11	0.05	0.17	0.11	0.17	0.11	0.17	0.06	0.00	0.17
2M8K	/	/	/	/	/	/	/	/	/	/		4.88	4.84	4.97	5.13	4.93	4.87	5.20	5.10	5.23	5.17
4C7O	/	/	/	/	/	/	/	/	/	/		0.59	0.66	0.00	0.05	0.05	0.10	0.68	0.40	0.45	0.45
2PXT	/	/	/	/	/	/	/	/	/	/		0.00	0.06	0.26	0.20	0.58	0.15	0.06	0.06	0.21	0.06
2LU0	/	/	/	/	/	/	/	/	/	/		0.00	1.40	0.09	1.45	0.06	0.06	1.52	0.06	0.16	5.48
6MXQ	/	/	/	/	/	/	/	/	/	/		2.38	0.00	0.25	3.00	4.26	2.81	2.29	0.06	2.81	2.75
1U63	/	/	/	/	/	/	/	/	/	/		0.11	0.06	0.18	0.12	0.06	0.00	0.12	0.12	0.18	0.12
2PXQ	/	/	/	/	/	/	/	/	/	/		0.00	0.06	0.26	0.20	0.58	0.15	0.06	0.06	0.21	0.06
2PXP	/	/	/	/	/	/	/	/	/	/		0.00	0.06	0.26	0.20	0.58	0.15	0.06	0.06	0.21	0.06
2PXD	/	/	/	/	/	/	/	/	/	/		0.00	0.06	0.26	0.20	0.58	0.15	0.06	0.06	0.21	10.14
2PXF	/	/	/	/	/	/	/	/	/	/		0.00	0.06	0.26	0.20	0.58	0.15	0.06	0.06	0.21	0.06
2PXE	/	/	/	/	/	/	/	/	/	/		0.00	0.06	0.26	0.06	0.06	0.20	0.16	0.58	0.15	0.06
2PXK	/	/	/	/	/	/	/	/	/	/		0.00	0.06	0.06	0.26	0.06	0.20	0.16	0.58	0.15	0.06
2PXU	/	/	/	/	/	/	/	/	/	/		0.00	0.06	0.26	0.20	0.58	0.15	0.06	0.06	0.21	0.06
2PXB	/	/	/	/	/	/	/	/	/	/		0.06	0.13	0.33	0.28	0.67	0.22	0.13	0.13	0.28	0.13
2PXV	/	/	/	/	/	/	/	/	/	/		0.00	0.06	0.26	0.20	0.58	0.15	0.06	0.06	0.21	0.06
6IV9	/	/	/	/	/	/	/	/	/	/		0.22	0.35	0.24	0.38	4.30	3.05	2.84	2.26	4.63	1.95
6IV8	/	/	/	/	/	/	/	/	/	/		0.22	0.35	0.24	0.38	4.30	3.05	2.84	2.26	4.63	1.95

	DNA model											RNA model									
PDB	RNAfold MFE	subopt #2	subopt #3	subopt #4	subopt #5	subopt #6	subopt #7	subopt #8	subopt #9	subopt #10		RNAfold MFE	subopt #2	subopt #3	subopt #4	subopt #5	subopt #6	subopt #7	subopt #8	subopt #9	subopt #10
2MHI	/	/	/	/	/	/	/	/	/	/		0.15	0.21	0.28	0.23	1.62	0.16	0.22	0.21	0.26	0.21
2N4L	/	/	/	/	/	/	/	/	/	/		0.10	0.68	0.00	0.83	0.45	0.29	0.54	0.05	0.10	0.56
1P5M	/	/	/	/	/	/	/	/	/	/		0.00	0.08	0.05	0.05	0.11	0.19	0.05	0.14	1.06	0.05
2KZL	/	/	/	/	/	/	/	/	/	/		0.00	0.17	0.17	0.10	0.28	0.14	0.14	0.07	0.40	0.61
2HGH	/	/	/	/	/	/	/	/	/	/		0.64	0.13	0.00	3.32	0.72	4.50	0.22	0.56	0.28	0.72
6NOA	/	/	/	/	/	/	/	/	/	/		0.00	0.18	0.25	1.66	0.06	2.14	0.15	0.48	5.31	0.06
2LC8	/	/	/	/	/	/	/	/	/	/		7.65	7.59	9.27	7.54	7.49	7.88	7.82	7.51	7.46	11.55
6MCF	/	/	/	/	/	/	/	/	/	/		0.05	0.63	0.11	0.10	0.40	0.67	0.62	0.57	0.09	0.55
5IEM	/	/	/	/	/	/	/	/	/	/		0.00	0.57	0.07	0.05	0.34	0.61	0.56	0.51	0.05	0.49
4M4O	/	/	/	/	/	/	/	/	/	/		0.68	1.03	0.55	0.44	1.33	0.34	0.68	0.45	0.78	2.51
6DB8	/	/	/	/	/	/	/	/	/	/		1.00	1.06	1.24	1.22	1.09	1.57	1.05	2.24	2.21	1.42
4U7U	/	/	/	/	/	/	/	/	/	/		26.67	31.16	29.42	29.37	29.32	28.96	31.20	23.53	27.96	29.64
1UN6	/	/	/	/	/	/	/	/	/	/		0.49	0.28	0.05	10.58	10.54	0.35	0.40	0.40	0.55	0.38
2N3Q	/	/	/	/	/	/	/	/	/	/		9.05	8.86	9.05	9.03	8.86	8.83	0.10	9.00	8.97	9.12
3EGZ	/	/	/	/	/	/	/	/	/	/		0.12	0.00	0.31	0.20	0.24	0.06	0.64	0.20	0.18	0.29
5WT1	/	/	/	/	/	/	/	/	/	/		0.63	0.77	0.69	0.83	0.69	0.83	13.73	13.85	0.95	0.70
2NC1	/	/	/	/	/	/	/	/	/	/		0.51	0.56	0.56	0.56	0.56	0.62	0.56	0.59	0.56	0.56
5HR6	/	/	/	/	/	/	/	/	/	/		0.49	10.00	9.97	0.57	9.95	10.08	9.83	9.93	9.80	0.55
2N6W	/	/	/	/	/	/	/	/	/	/		0.35	0.37	0.39	0.32	0.34	0.16	0.36	0.13	0.40	0.28
2MQT	/	/	/	/	/	/	/	/	/	/		0.12	0.16	0.67	0.16	0.04	0.23	0.00	0.16	0.16	0.31
6U8D	/	/	/	/	/	/	/	/	/	/		0.04	7.45	0.08	0.11	7.73	7.57	7.70	0.16	0.15	7.71
3EPJ	/	/	/	/	/	/	/	/	/	/		2.61	2.58	0.85	2.58	2.46	0.78	1.00	9.14	2.54	0.83
5HR7	/	/	/	/	/	/	/	/	/	/		0.49	10.00	9.97	0.57	9.95	10.08	9.83	9.93	9.80	0.55
5V6X	/	/	/	/	/	/	/	/	/	/		0.34	0.39	0.43	0.42	0.40	0.48	0.39	0.47	0.39	0.50
2N8V	/	/	/	/	/	/	/	/	/	/		3.04	3.00	3.07	3.02	3.16	3.07	3.16	3.81	3.77	3.11
2DET	/	/	/	/	/	/	/	/	/	/		0.84	9.94	9.92	0.92	9.97	10.03	9.86	9.95	9.84	0.78
1KXK	/	/	/	/	/	/	/	/	/	/		0.26	0.22	0.17	0.13	0.31	0.28	0.26	0.23	0.19	0.17
2DU6	/	/	/	/	/	/	/	/	/	/		0.73	0.78	0.82	0.87	0.75	0.80	0.80	0.80	0.61	0.80
2DU3	/	/	/	/	/	/	/	/	/	/		6.89	6.70	7.00	6.40	6.83	6.80	6.61	7.09	6.98	0.84
2DU5	/	/	/	/	/	/	/	/	/	/		0.73	0.78	0.82	0.87	0.75	0.80	0.80	0.80	0.61	0.80
2ZZN	/	/	/	/	/	/	/	/	/	/		0.60	0.65	0.65	0.66	0.67	0.52	0.58	0.67	0.66	1.50
2L3J	/	/	/	/	/	/	/	/	/	/		0.00	0.03	0.03	0.03	0.03	0.03	0.03	0.07	0.03	0.03
2MS0	/	/	/	/	/	/	/	/	/	/		3.86	3.54	3.27	3.29	0.61	4.00	3.70	3.26	4.05	3.88
5TF6	/	/	/	/	/	/	/	/	/	/		17.03	17.97	22.97	20.55	16.29	17.86	30.27	19.00	31.14	17.24
4YVK	/	/	/	/	/	/	/	/	/	/		13.23	13.48	13.17	10.00	13.20	13.33	0.64	9.98	0.59	10.02
4YVI	/	/	/	/	/	/	/	/	/	/		10.06	10.45	10.24	10.04	9.89	0.59	10.02	10.27	0.64	10.63
4YVJ	/	/	/	/	/	/	/	/	/	/		0.64	13.23	1.89	0.59	1.76	0.61	0.51	0.76	13.48	0.84
2AKE	/	/	/	/	/	/	/	/	/	/		1.19	0.00	0.07	1.27	1.10	3.11	1.07	1.27	1.51	0.05
4ZT0	/	/	/	/	/	/	/	/	/	/		6.52	8.21	5.93	7.61	6.22	7.92	6.88	7.02	6.63	6.73
1DRZ	/	/	/	/	/	/	/	/	/	/		9.93	9.98	9.89	9.91	9.93	9.96	10.04	10.09	9.96	9.66
2ZNI	/	/	/	/	/	/	/	/	/	/		0.10	0.19	0.19	0.15	0.15	0.71	0.28	0.19	0.15	0.15
1EUQ	/	/	/	/	/	/	/	/	/	/		2.12	2.41	0.59	0.49	1.41	2.28	1.85	1.98	1.36	0.59
2MF0	/	/	/	/	/	/	/	/	/	/		0.11	0.05	0.16	0.05	0.11	0.95	0.23	0.16	0.16	0.00
3WC1	/	/	/	/	/	/	/	/	/	/		7.00	7.30	7.21	7.09	6.72	7.51	6.38	7.39	6.61	7.04
4X0A	/	/	/	/	/	/	/	/	/	/		0.64	0.75	0.61	0.78	0.70	0.71	0.63	0.68	1.18	0.70
3WFQ	/	/	/	/	/	/	/	/	/	/		0.83	0.88	0.90	0.90	0.88	0.90	0.80	0.85	1.34	1.02
5WT3	/	/	/	/	/	/	/	/	/	/		0.55	0.67	0.60	0.72	0.60	0.72	0.82	0.60	0.93	0.73
5VW1	/	/	/	/	/	/	/	/	/	/		3.38	5.41	0.60	0.15	10.97	0.53	10.50	17.22	3.42	3.88

	DNA model											RNA model									
PDB	RNAfold MFE	subopt #2	subopt #3	subopt #4	subopt #5	subopt #6	subopt #7	subopt #8	subopt #9	subopt #10		RNAfold MFE	subopt #2	subopt #3	subopt #4	subopt #5	subopt #6	subopt #7	subopt #8	subopt #9	subopt #10
2ZM5	/	/	/	/	/	/	/	/	/	/		0.60	0.52	0.67	0.66	0.59	8.52	8.42	0.58	8.56	1.59
3FOZ	/	/	/	/	/	/	/	/	/	/		0.60	0.52	0.67	0.66	0.59	8.52	8.42	0.58	8.56	1.59
3TUP	/	/	/	/	/	/	/	/	/	/		8.77	8.76	8.84	8.84	8.87	8.86	9.02	10.84	8.91	8.86
5D6G	/	/	/	/	/	/	/	/	/	/		0.68	1.08	0.77	0.79	0.92	1.30	0.65	1.05	0.74	1.33
4YCO	/	/	/	/	/	/	/	/	/	/		0.60	0.52	0.67	0.66	0.59	8.52	8.42	0.58	8.56	1.59
3WC2	/	/	/	/	/	/	/	/	/	/		9.87	9.73	9.56	10.13	9.50	10.00	9.36	10.00	9.82	9.18
3WFS	/	/	/	/	/	/	/	/	/	/		0.00	0.07	0.05	0.09	0.07	0.05	0.07	0.12	0.51	0.07
1GTR	/	/	/	/	/	/	/	/	/	/		0.54	0.59	2.18	2.48	11.09	10.98	0.61	1.93	1.31	1.54
2DER	/	/	/	/	/	/	/	/	/	/		1.05	10.84	10.82	1.12	10.77	10.95	10.64	10.74	10.62	1.13
4YYE	/	/	/	/	/	/	/	/	/	/		1.96	1.98	10.21	1.93	0.88	1.95	1.95	1.96	1.89	1.98
3AKZ	/	/	/	/	/	/	/	/	/	/		9.60	9.98	9.85	9.57	9.43	0.50	9.54	9.81	0.56	10.24
2IHX	/	/	/	/	/	/	/	/	/	/		0.00	1.46	0.21	0.04	0.20	0.16	0.04	0.38	0.34	0.20
2DR2	/	/	/	/	/	/	/	/	/	/		1.40	0.15	0.22	1.49	1.31	3.40	1.28	1.49	1.73	0.21
4TZV	/	/	/	/	/	/	/	/	/	/		3.53	3.83	3.52	0.60	3.82	0.52	3.56	3.66	3.52	3.84
5X6B	/	/	/	/	/	/	/	/	/	/		0.77	0.82	0.82	0.83	0.84	0.69	0.74	0.84	1.33	5.91
2ZUE	/	/	/	/	/	/	/	/	/	/		1.98	1.91	1.89	1.83	4.88	3.20	1.98	2.19	1.91	2.13
3WQZ	/	/	/	/	/	/	/	/	/	/		9.84	9.86	9.82	9.90	9.90	9.84	9.90	10.21	10.02	9.96
4WC2	/	/	/	/	/	/	/	/	/	/		0.55	0.60	0.61	0.63	0.60	0.61	0.53	0.58	1.05	0.60
1N77	/	/	/	/	/	/	/	/	/	/		0.52	0.58	0.49	0.60	0.58	0.75	0.62	0.67	0.58	0.60
3WQY	/	/	/	/	/	/	/	/	/	/		9.92	9.94	9.90	9.98	9.98	9.92	9.98	10.30	10.10	10.04
1FFY	/	/	/	/	/	/	/	/	/	/		10.90	11.19	11.04	11.04	10.98	11.28	11.06	10.84	1.87	11.38
1EIY	/	/	/	/	/	/	/	/	/	/		0.56	0.49	0.61	0.62	0.55	0.57	0.62	0.55	0.55	0.67
4WC3	/	/	/	/	/	/	/	/	/	/		0.55	0.60	0.61	0.63	0.60	0.61	0.53	0.58	1.05	0.60
2K4C	/	/	/	/	/	/	/	/	/	/		8.40	8.39	8.67	8.67	8.91	8.91	8.39	8.38	9.17	9.18
4WJ3	/	/	/	/	/	/	/	/	/	/		6.91	7.00	2.27	7.11	6.83	7.11	6.67	0.55	2.37	6.83
3A2K	/	/	/	/	/	/	/	/	/	/		7.62	7.83	7.73	7.71	7.60	7.62	7.50	7.60	7.39	7.94
5CCB	/	/	/	/	/	/	/	/	/	/		2.85	2.47	2.74	2.35	2.57	2.76	2.55	2.38	2.72	2.44
1P5P	/	/	/	/	/	/	/	/	/	/		0.04	0.10	9.90	0.00	0.06	9.38	0.08	10.27	0.08	0.18
4X0B	/	/	/	/	/	/	/	/	/	/		0.31	0.43	0.26	0.45	0.38	0.37	0.48	0.50	0.85	0.38
3AMT	/	/	/	/	/	/	/	/	/	/		1.66	1.57	4.14	2.13	1.55	1.61	1.75	1.47	1.74	1.73
3U4M	/	/	/	/	/	/	/	/	/	/		3.02	3.28	10.46	1.93	3.32	10.35	11.39	6.32	4.56	4.53
6B14	/	/	/	/	/	/	/	/	/	/		0.57	0.65	0.60	0.68	0.47	0.51	0.54	0.45	9.88	0.60
6B3K	/	/	/	/	/	/	/	/	/	/		11.41	9.79	11.42	9.82	11.82	10.98	10.16	11.82	11.00	11.79
2ZZM	/	/	/	/	/	/	/	/	/	/		1.85	1.82	1.89	6.57	1.98	2.15	1.93	15.22	6.82	1.87
3A3A	/	/	/	/	/	/	/	/	/	/		0.34	0.48	0.38	0.53	0.60	0.38	0.75	0.38	0.53	0.40
3K0J	/	/	/	/	/	/	/	/	/	/		7.42	5.09	6.20	14.47	14.08	5.86	5.90	6.76	14.26	6.46
1WZ2	/	/	/	/	/	/	/	/	/	/		10.79	10.65	10.36	10.58	10.49	1.27	1.31	10.96	10.67	10.65
5XBL	/	/	/	/	/	/	/	/	/	/		4.80	4.61	4.68	4.86	4.80	4.78	5.09	5.23	4.41	4.39
3ADB	/	/	/	/	/	/	/	/	/	/		0.31	0.35	0.34	0.36	0.35	0.43	0.47	1.15	1.34	1.18
2N7M	/	/	/	/	/	/	/	/	/	/		0.03	0.00	0.34	9.88	0.06	0.32	0.06	0.06	0.03	0.29
3W1K	/	/	/	/	/	/	/	/	/	/		0.37	0.40	0.40	0.44	0.40	0.42	0.40	0.42	0.51	0.41
3KTW	/	/	/	/	/	/	/	/	/	/		6.09	6.13	6.03	6.06	7.21	7.24	5.21	5.24	7.05	5.32
2V3C	/	/	/	/	/	/	/	/	/	/		0.03	0.41	0.31	0.89	0.19	0.22	0.22	0.57	0.59	0.47
6JXM	/	/	/	/	/	/	/	/	/	/		1.73	0.43	1.93	0.64	1.64	1.84	1.63	0.36	0.47	0.68
1LNG	/	/	/	/	/	/	/	/	/	/		0.03	0.41	0.31	5.43	6.82	6.85	6.88	6.86	0.89	0.19
3W3S	/	/	/	/	/	/	/	/	/	/		0.45	0.50	0.42	0.47	0.53	0.36	0.41	0.33	0.38	0.44
6MJ0	/	/	/	/	/	/	/	/	/	/		10.52	10.45	10.41	10.35	10.56	10.49	10.46	10.56	10.39	10.49
1S9S	/	/	/	/	/	/	/	/	/	/		0.00	0.03	0.06	0.03	0.10	0.03	0.09	0.08	0.06	0.42

	DNA model											RNA model									
PDB	RNAfold MFE	subopt #2	subopt #3	subopt #4	subopt #5	subopt #6	subopt #7	subopt #8	subopt #9	subopt #10		RNAfold MFE	subopt #2	subopt #3	subopt #4	subopt #5	subopt #6	subopt #7	subopt #8	subopt #9	subopt #10
2XXA	/	/	/	/	/	/	/	/	/	/		8.66	0.03	0.00	8.58	9.07	0.06	0.03	2.82	2.79	8.74
2KRL	/	/	/	/	/	/	/	/	/	/		1.49	6.24	1.55	1.55	1.48	6.03	1.55	6.38	5.69	6.38
7K1Z	/	/	/	/	/	/	/	/	/	/		1.72	1.59	1.68	1.53	1.71	2.37	2.29	2.25	1.62	1.72
2NBX	/	/	/	/	/	/	/	/	/	/		0.68	0.87	0.69	0.71	0.89	0.91	0.62	0.71	0.70	0.57
2LKR	/	/	/	/	/	/	/	/	/	/		2.09	2.01	1.58	2.18	1.49	2.15	5.53	2.10	2.08	4.64
4P3E	/	/	/	/	/	/	/	/	/	/		1.33	1.31	7.30	7.26	1.37	1.35	1.35	1.34	1.37	1.35
3IVK	/	/	/	/	/	/	/	/	/	/		4.92	5.52	5.01	5.01	5.17	5.61	5.61	5.76	5.01	4.96
3NDB	/	/	/	/	/	/	/	/	/	/		0.06	0.31	0.24	0.61	0.09	0.16	0.18	0.18	0.07	0.33
2N1Q	/	/	/	/	/	/	/	/	/	/		7.39	9.10	7.47	9.07	9.18	9.15	7.85	7.51	9.22	11.90
2R8S	/	/	/	/	/	/	/	/	/	/		4.29	4.27	0.64	0.62	3.73	3.71	0.63	0.61	3.84	3.82
4P8Z	/	/	/	/	/	/	/	/	/	/		13.32	9.79	9.80	9.85	9.86	8.64	8.69	13.40	13.34	13.41
1GRZ	/	/	/	/	/	/	/	/	/	/		2.85	2.76	2.84	2.75	2.82	2.73	2.81	2.71	2.83	2.73
5IWA	/	/	/	/	/	/	/	/	/	/		57.02	/	/	/	/	/	/	/	/	/
