# Supplementary material for: Comparative Study of Single-stranded Oligonucleotides Secondary Structure Prediction Tools
Source: BMC Bioinformatics. 2023 Nov 8;24:422. doi: 10.1186/s12859-023-05532-5 (PMC10634105; doi:10.1186/s12859-023-05532-5)
Supplement: Supplementary file 5 — Additional file 5. Comparison between predicted and experimental secondary structure using the AptaMat distance as a metric for mfold under RNA (Mathews (1999)) and DNA (SantaLucia (1998)) model. The PDB code is reported in the first column. $Apta_D$AptaD values are reported for each PDB and associated optimal/suboptimal prediction. "/" characters indicate either structures predicted as unfolded or software failure during the computation. [file 12859_2023_5532_MOESM5_ESM.pdf]

Additional File 5. Comparison between predicted and experimental secondary structure using the AptaMat distance as a metric for mfold under RNA (Mathews (1999)) and DNA (SantaLucia (1998)) model. The PDB code is reported in the first column. AptaD values are reported for each PDB and associated optimal/suboptimal prediction. "/" characters indicate either structures predicted as unfolded or software failure during the computation.

	DNA model											RNA model									
PDB	mfold MFE	subopt #2	subopt #3	subopt #4	subopt #5	subopt #6	subopt #7	subopt #8	subopt #9	subopt #10		mfold MFE	subopt #2	subopt #3	subopt #4	subopt #5	subopt #6	subopt #7	subopt #8	subopt #9	subopt #10
1PQT	0.00	/	/	/	/	/	/	/	/	/		0.00	/	/	/	/	/	/	/	/	/
2K71	0.00	/	/	/	/	/	/	/	/	/		0.00	/	/	/	/	/	/	/	/	/
5GWL	/	/	/	/	/	/	/	/	/	/		/	/	/	/	/	/	/	/	/	/
5GWQ	/	/	/	/	/	/	/	/	/	/		/	/	/	/	/	/	/	/	/	/
6J37	/	/	/	/	/	/	/	/	/	/		/	/	/	/	/	/	/	/	/	/
6M0B	/	/	/	/	/	/	/	/	/	/		/	/	/	/	/	/	/	/	/	/
6M0C	/	/	/	/	/	/	/	/	/	/		/	/	/	/	/	/	/	/	/	/
5OND	1.00	0.50	2.33	/	/	/	/	/	/	/		0.50	/	/	/	/	/	/	/	/	/
1ZHU	0.00	/	/	/	/	/	/	/	/	/		2.00	2.00	0.00	2.00	/	/	/	/	/	/
2A0I	3.00	5.00	3.67	5.00	/	/	/	/	/	/		5.00	5.00	5.00	/	/	/	/	/	/	/
2LO8	3.20	0.60	1.75	2.00	/	/	/	/	/	/		0.60	3.20	1.60	/	/	/	/	/	/	/
3WPD	0.00	/	/	/	/	/	/	/	/	/		0.00	1.00	/	/	/	/	/	/	/	/
6IY5	0.67	0.00	/	/	/	/	/	/	/	/		0.00	/	/	/	/	/	/	/	/	/
1BJH	0.00	/	/	/	/	/	/	/	/	/		0.00	/	/	/	/	/	/	/	/	/
3WPG	1.00	/	/	/	/	/	/	/	/	/		1.00	0.00	/	/	/	/	/	/	/	/
2LO5	0.43	/	/	/	/	/	/	/	/	/		0.43	/	/	/	/	/	/	/	/	/
3WPH	1.00	/	/	/	/	/	/	/	/	/		1.00	0.00	/	/	/	/	/	/	/	/
6FKE	0.29	0.00	/	/	/	/	/	/	/	/		0.00	/	/	/	/	/	/	/	/	/
1LA8	0.00	/	/	/	/	/	/	/	/	/		0.00	/	/	/	/	/	/	/	/	/
1P0U	0.00	/	/	/	/	/	/	/	/	/		0.00	/	/	/	/	/	/	/	/	/
2EXF	0.00	1.00	/	/	/	/	/	/	/	/		0.00	1.29	1.00	/	/	/	/	/	/	/
2JZW	0.00	1.00	/	/	/	/	/	/	/	/		0.00	1.29	1.00	/	/	/	/	/	/	/
5F55	3.00	/	/	/	/	/	/	/	/	/		3.00	3.67	5.00	/	/	/	/	/	/	/
6FK5	0.00	/	/	/	/	/	/	/	/	/		0.00	/	/	/	/	/	/	/	/	/
1UUT	0.00	/	/	/	/	/	/	/	/	/		0.00	/	/	/	/	/	/	/	/	/
2M8Y	0.00	6.25	0.27	5.25	/	/	/	/	/	/		0.27	5.25	0.00	/	/	/	/	/	/	/
1AC7	0.00	/	/	/	/	/	/	/	/	/		0.00	/	/	/	/	/	/	/	/	/
6FK4	0.00	4.00	4.00	/	/	/	/	/	/	/		0.00	4.00	/	/	/	/	/	/	/	/
1XUE	3.00	6.33	8.00	/	/	/	/	/	/	/		8.00	/	/	/	/	/	/	/	/	/
1EN1	0.43	1.43	/	/	/	/	/	/	/	/		0.75	0.00	0.67	1.33	/	/	/	/	/	/
4KB0	0.33	0.33	0.00	0.33	0.91	/	/	/	/	/		0.33	0.33	0.91	1.00	0.00	0.33	1.20	1.64	1.50	1.09
4KB1	0.33	0.00	0.45	0.91	0.36	/	/	/	/	/		0.33	0.91	0.33	1.00	0.00	1.20	1.27	1.64	0.33	1.09
1ECU	0.13	0.00	0.33	0.33	0.57	3.09	0.47	3.09	0.86	/		0.33	0.33	0.47	2.00	3.09	3.08	0.00	0.57	3.54	/
3Q0A	0.00	2.56	4.00	/	/	/	/	/	/	/		0.00	/	/	/	/	/	/	/	/	/
4FF1	0.00	1.82	3.33	1.73	2.56	4.00	2.00	6.30	/	/		0.00	/	/	/	/	/	/	/	/	/
3C46	0.18	2.67	4.20	/	/	/	/	/	/	/		0.18	12.00	/	/	/	/	/	/	/	/
3Q23	0.18	2.67	4.20	/	/	/	/	/	/	/		0.18	12.00	/	/	/	/	/	/	/	/
2A6O	0.00	0.47	0.47	0.67	0.86	0.93	0.86	1.14	1.64	2.14		0.00	0.47	0.67	0.93	0.86	1.14	1.64	2.50	2.38	7.38
3Q24	0.18	4.20	12.00	/	/	/	/	/	/	/		0.18	12.00	/	/	/	/	/	/	/	/
2L5K	2.73	11.67	0.00	/	/	/	/	/	/	/		0.00	11.67	1.38	/	/	/	/	/	/	/
3DSD	0.00	1.00	0.46	1.00	4.60	12.00	6.00	/	/	/		0.00	1.08	0.23	1.00	6.00	12.00	/	/	/	/
2VHG	0.00	0.47	0.47	0.67	0.86	0.93	0.86	1.14	1.64	2.14		0.00	0.47	0.86	2.50	7.38	0.93	1.64	2.38	9.31	0.67
1OSB	0.18	0.50	1.92	0.92	0.64	1.91	1.73	2.10	1.27	0.80		1.60	2.10	0.50	1.33	0.80	1.20	1.91	1.92	1.92	1.73
1ZM5	0.18	0.50	1.92	0.92	0.64	1.91	1.73	2.10	1.27	0.80		1.60	2.10	0.50	1.33	0.80	1.20	1.91	1.92	1.92	1.73
2CDM	2.50	0.62	0.93	1.23	1.17	2.50	1.50	1.82	3.08	3.00		2.50	2.50	1.82	4.20	0.93	1.17	3.08	3.40	3.80	1.23
2VIC	0.12	0.56	14.31	0.56	0.93	0.75	13.79	14.00	11.86	13.73		0.00	0.47	0.86	0.12	0.93	14.31	2.60	2.54	7.36	13.79
5N2Q	0.00	1.80	1.93	1.80	1.79	3.47	1.79	3.47	1.64	1.86		0.00	3.13	1.80	3.38	5.00	/	/	/	/	/
1JVE	0.00	0.13	0.32	0.36	0.36	0.67	/	/	/	/		0.00	0.13	0.22	0.95	1.10	1.10	/	/	/	/
1NGO	0.00	1.00	1.20	12.43	12.13	1.40	0.95	1.88	/	/		0.00	2.00	0.33	0.24	0.43	0.43	12.13	1.40	0.79	1.00
1NGU	0.00	0.82	12.50	12.15	12.00	1.73	/	/	/	/		0.00	0.82	0.22	12.15	0.75	1.06	12.00	1.73	1.29	/
3ZH2	2.00	3.46	3.31	1.31	0.00	11.78	11.20	/	/	/		2.00	2.80	1.87	2.80	0.00	3.46	1.43	1.14	0.69	3.46
4HT4	0.00	6.93	0.53	2.13	1.64	3.07	1.93	3.00	14.69	1.07		0.00	5.00	0.53	2.13	23.00	14.69	10.17	1.73	4.00	0.23
1YTB	0.00	0.36	1.10	1.10	0.95	1.30	1.30	0.91	1.10	1.47		0.64	1.00	1.00	1.36	1.48	1.95	1.50	0.00	0.30	0.30
1B4Y	0.00	6.00	10.20	7.00	/	/	/	/	/	/		0.00	6.00	7.00	4.08	10.18	/	/	/	/	/
4ER8	0.11	1.44	2.12	6.38	6.67	15.00	4.38	15.00	/	/		0.11	2.12	15.18	9.29	9.31	1.44	6.38	4.38	5.65	/

	DNA model											RNA model									
PDB	mfold MFE	subopt #2	subopt #3	subopt #4	subopt #5	subopt #6	subopt #7	subopt #8	subopt #9	subopt #10		mfold MFE	subopt #2	subopt #3	subopt #4	subopt #5	subopt #6	subopt #7	subopt #8	subopt #9	subopt #10
4F41	0.07	1.08	1.08	6.55	2.28	/	/	/	/	/		0.07	1.32	1.32	6.08	14.30	3.88	/	/	/	/
4F43	0.07	1.08	6.55	1.08	6.52	3.92	10.57	10.80	/	/		0.07	6.55	1.32	1.32	2.04	/	/	/	/	/
5HRU	5.00	1.37	4.00	3.22	/	/	/	/	/	/		3.30	4.11	1.20	5.79	3.22	7.20	5.89	3.28	4.94	4.47
6SEI	0.00	17.80	7.11	2.30	9.40	/	/	/	/	/		7.59	10.78	14.57	14.65	0.00	2.00	5.00	2.30	/	/
5HTO	1.09	4.47	3.19	5.57	2.32	/	/	/	/	/		4.64	4.45	7.76	3.24	5.00	4.55	3.65	1.24	5.86	2.90
2VJU	1.05	4.16	2.53	7.87	/	/	/	/	/	/		0.10	4.16	4.00	1.05	9.93	16.08	2.53	3.20	/	/
1EZN	0.14	0.74	4.17	1.44	3.38	3.70	13.00	7.08	1.76	8.38		0.14	0.82	1.76	3.44	3.40	7.28	8.09	13.38	12.50	10.29
1SNJ	0.14	9.31	10.32	4.16	3.85	3.56	3.83	13.27	3.37	9.13		0.14	9.31	4.16	3.85	10.32	12.12	3.60	4.22	4.83	12.75
6U82	0.00	2.63	5.62	3.74	1.85	3.27	5.30	8.26	/	/		5.30	6.92	16.27	21.36	5.00	15.36	2.27	0.00	4.81	2.27
3HXO	10.17	5.18	6.05	8.00	8.91	8.57	7.71	6.67	7.43	6.75		10.17	7.86	9.00	5.57	7.56	8.22	9.82	9.35	7.88	6.18
2N8A	0.05	0.47	1.21	1.58	4.88	0.97	2.87	2.70	2.87	3.93		0.05	4.88	1.94	3.13	2.87	2.97	3.37	11.52	22.36	20.77
3THW	0.00	3.27	5.27	8.40	8.86	/	/	/	/	/		8.08	2.73	0.00	2.08	10.57	/	/	/	/	/
2IXZ	/	/	/	/	/	/	/	/	/	/		0.50	/	/	/	/	/	/	/	/	/
2OJ7	/	/	/	/	/	/	/	/	/	/		0.00	/	/	/	/	/	/	/	/	/
1R4H	/	/	/	/	/	/	/	/	/	/		0.67	/	/	/	/	/	/	/	/	/
1IDV	/	/	/	/	/	/	/	/	/	/		0.00	/	/	/	/	/	/	/	/	/
2MXJ	/	/	/	/	/	/	/	/	/	/		0.00	/	/	/	/	/	/	/	/	/
5FMZ	/	/	/	/	/	/	/	/	/	/		0.00	/	/	/	/	/	/	/	/	/
1RNG	/	/	/	/	/	/	/	/	/	/		0.22	2.29	/	/	/	/	/	/	/	/
2F87	/	/	/	/	/	/	/	/	/	/		0.00	/	/	/	/	/	/	/	/	/
1ZIF	/	/	/	/	/	/	/	/	/	/		0.00	0.71	0.71	/	/	/	/	/	/	/
1ZIG	/	/	/	/	/	/	/	/	/	/		0.00	0.71	0.71	/	/	/	/	/	/	/
1ZIH	/	/	/	/	/	/	/	/	/	/		0.00	0.71	0.71	/	/	/	/	/	/	/
1AFX	/	/	/	/	/	/	/	/	/	/		0.00	/	/	/	/	/	/	/	/	/
4Z0C	/	/	/	/	/	/	/	/	/	/		0.67	/	/	/	/	/	/	/	/	/
1VOP	/	/	/	/	/	/	/	/	/	/		0.22	/	/	/	/	/	/	/	/	/
1HS8	/	/	/	/	/	/	/	/	/	/		0.00	/	/	/	/	/	/	/	/	/
1HS4	/	/	/	/	/	/	/	/	/	/		0.00	/	/	/	/	/	/	/	/	/
1HS1	/	/	/	/	/	/	/	/	/	/		0.00	/	/	/	/	/	/	/	/	/
1HS2	/	/	/	/	/	/	/	/	/	/		0.00	0.43	/	/	/	/	/	/	/	/
1HS3	/	/	/	/	/	/	/	/	/	/		0.00	/	/	/	/	/	/	/	/	/
6FQ3	/	/	/	/	/	/	/	/	/	/		0.22	0.00	/	/	/	/	/	/	/	/
1ESH	/	/	/	/	/	/	/	/	/	/		0.00	/	/	/	/	/	/	/	/	/
1JZC	/	/	/	/	/	/	/	/	/	/		0.00	/	/	/	/	/	/	/	/	/
1I46	/	/	/	/	/	/	/	/	/	/		0.00	/	/	/	/	/	/	/	/	/
1I4B	/	/	/	/	/	/	/	/	/	/		0.00	/	/	/	/	/	/	/	/	/
6FQL	/	/	/	/	/	/	/	/	/	/		0.22	0.00	/	/	/	/	/	/	/	/
4Z7L	/	/	/	/	/	/	/	/	/	/		0.00	/	/	/	/	/	/	/	/	/
2KOC	/	/	/	/	/	/	/	/	/	/		0.00	/	/	/	/	/	/	/	/	/
1F85	/	/	/	/	/	/	/	/	/	/		0.00	1.86	1.75	/	/	/	/	/	/	/
2Y95	/	/	/	/	/	/	/	/	/	/		0.00	2.29	2.29	/	/	/	/	/	/	/
1FHK	/	/	/	/	/	/	/	/	/	/		0.29	3.00	/	/	/	/	/	/	/	/
1IK1	/	/	/	/	/	/	/	/	/	/		0.00	/	/	/	/	/	/	/	/	/
1ROQ	/	/	/	/	/	/	/	/	/	/		0.00	/	/	/	/	/	/	/	/	/
2EVY	/	/	/	/	/	/	/	/	/	/		0.22	/	/	/	/	/	/	/	/	/
1K4A	/	/	/	/	/	/	/	/	/	/		0.00	/	/	/	/	/	/	/	/	/
1K4B	/	/	/	/	/	/	/	/	/	/		0.00	/	/	/	/	/	/	/	/	/
4AL7	/	/	/	/	/	/	/	/	/	/		0.00	/	/	/	/	/	/	/	/	/
1OQ0	/	/	/	/	/	/	/	/	/	/		0.18	0.00	2.22	2.22	/	/	/	/	/	/
2LPA	/	/	/	/	/	/	/	/	/	/		0.00	/	/	/	/	/	/	/	/	/
1QFQ	/	/	/	/	/	/	/	/	/	/		0.00	1.25	1.57	/	/	/	/	/	/	/
1A4T	/	/	/	/	/	/	/	/	/	/		0.00	2.29	/	/	/	/	/	/	/	/
1ATW	/	/	/	/	/	/	/	/	/	/		0.00	0.33	0.56	/	/	/	/	/	/	/
1XWP	/	/	/	/	/	/	/	/	/	/		0.00	/	/	/	/	/	/	/	/	/
1Q75	/	/	/	/	/	/	/	/	/	/		0.00	0.80	/	/	/	/	/	/	/	/
4AL5	/	/	/	/	/	/	/	/	/	/		0.00	/	/	/	/	/	/	/	/	/
2MNC	/	/	/	/	/	/	/	/	/	/		0.00	0.20	0.36	/	/	/	/	/	/	/
1XWU	/	/	/	/	/	/	/	/	/	/		0.44	/	/	/	/	/	/	/	/	/
2LP9	/	/	/	/	/	/	/	/	/	/		0.27	/	/	/	/	/	/	/	/	/

	DNA model											RNA model									
**PDB**	**mfold MFE**	**subopt #2**	**subopt #3**	**subopt #4**	**subopt #5**	**subopt #6**	**subopt #7**	**subopt #8**	**subopt #9**	**subopt #10**		**mfold MFE**	**subopt #2**	**subopt #3**	**subopt #4**	**subopt #5**	**subopt #6**	**subopt #7**	**subopt #8**	**subopt #9**	**subopt #10**
2L6I	/	/	/	/	/	/	/	/	/	/		0.27	/	/	/	/	/	/	/	/	/
4ILM	/	/	/	/	/	/	/	/	/	/		5.00	0.29	/	/	/	/	/	/	/	/
1JWC	/	/	/	/	/	/	/	/	/	/		0.00	0.17	0.64	0.55	0.80	0.73	/	/	/	/
1JTW	/	/	/	/	/	/	/	/	/	/		0.50	0.00	0.40	2.25	/	/	/	/	/	/
4QIL	/	/	/	/	/	/	/	/	/	/		0.00	1.20	0.45	0.45	0.50	/	/	/	/	/
6CYT	/	/	/	/	/	/	/	/	/	/		0.83	/	/	/	/	/	/	/	/	/
2JR4	/	/	/	/	/	/	/	/	/	/		0.00	0.64	4.00	1.44	0.36	1.20	3.00	1.89	1.70	2.56
2KRP	/	/	/	/	/	/	/	/	/	/		0.00	5.25	/	/	/	/	/	/	/	/
1YN1	/	/	/	/	/	/	/	/	/	/		0.00	0.73	1.27	/	/	/	/	/	/	/
2M4W	/	/	/	/	/	/	/	/	/	/		0.40	0.63	0.67	0.33	/	/	/	/	/	/
1WKS	/	/	/	/	/	/	/	/	/	/		0.00	0.45	0.50	0.55	/	/	/	/	/	/
1ATV	/	/	/	/	/	/	/	/	/	/		0.00	0.45	0.45	0.64	/	/	/	/	/	/
2LBL	/	/	/	/	/	/	/	/	/	/		7.00	0.00	6.29	6.25	0.45	/	/	/	/	/
2LBK	/	/	/	/	/	/	/	/	/	/		0.00	6.00	6.00	0.27	7.00	0.45	5.25	0.33	0.64	0.45
2LBJ	/	/	/	/	/	/	/	/	/	/		0.00	0.23	0.38	6.20	0.54	0.38	7.00	/	/	/
2LAC	/	/	/	/	/	/	/	/	/	/		0.00	1.00	2.00	1.25	/	/	/	/	/	/
1KKA	/	/	/	/	/	/	/	/	/	/		0.15	1.20	2.22	1.67	/	/	/	/	/	/
2KVN	/	/	/	/	/	/	/	/	/	/		0.00	0.45	0.64	/	/	/	/	/	/	/
4ZLD	/	/	/	/	/	/	/	/	/	/		0.15	/	/	/	/	/	/	/	/	/
1BZ2	/	/	/	/	/	/	/	/	/	/		0.00	/	/	/	/	/	/	/	/	/
1BZ3	/	/	/	/	/	/	/	/	/	/		0.36	/	/	/	/	/	/	/	/	/
2KPC	/	/	/	/	/	/	/	/	/	/		0.18	0.00	0.55	/	/	/	/	/	/	/
2KPD	/	/	/	/	/	/	/	/	/	/		0.22	0.00	0.60	/	/	/	/	/	/	/
2GVO	/	/	/	/	/	/	/	/	/	/		0.00	0.17	0.38	6.00	/	/	/	/	/	/
2QH4	/	/	/	/	/	/	/	/	/	/		0.00	/	/	/	/	/	/	/	/	/
1Z30	/	/	/	/	/	/	/	/	/	/		0.00	0.38	4.60	/	/	/	/	/	/	/
2Y9H	/	/	/	/	/	/	/	/	/	/		0.00	1.31	1.50	2.00	2.18	/	/	/	/	/
4QI2	/	/	/	/	/	/	/	/	/	/		0.15	7.00	/	/	/	/	/	/	/	/
5N5C	/	/	/	/	/	/	/	/	/	/		0.29	0.42	0.42	/	/	/	/	/	/	/
6TQB	/	/	/	/	/	/	/	/	/	/		0.00	/	/	/	/	/	/	/	/	/
2B7G	/	/	/	/	/	/	/	/	/	/		0.00	3.00	0.54	1.62	/	/	/	/	/	/
2B6G	/	/	/	/	/	/	/	/	/	/		0.20	2.83	0.71	1.57	/	/	/	/	/	/
1ATO	/	/	/	/	/	/	/	/	/	/		0.00	0.92	0.33	0.33	0.67	5.25	0.91	0.50	/	/
2MEQ	/	/	/	/	/	/	/	/	/	/		0.00	0.38	0.42	/	/	/	/	/	/	/
1ESY	/	/	/	/	/	/	/	/	/	/		0.15	7.20	1.00	/	/	/	/	/	/	/
1UUU	/	/	/	/	/	/	/	/	/	/		0.29	0.46	0.67	4.00	4.22	0.91	/	/	/	/
2MFD	/	/	/	/	/	/	/	/	/	/		0.00	9.00	0.38	/	/	/	/	/	/	/
1I3X	/	/	/	/	/	/	/	/	/	/		0.00	4.00	4.00	3.58	4.00	4.00	3.42	/	/	/
2RLU	/	/	/	/	/	/	/	/	/	/		0.00	0.13	7.00	/	/	/	/	/	/	/
2Y8Y	/	/	/	/	/	/	/	/	/	/		0.15	1.33	0.67	1.36	2.00	2.00	/	/	/	/
1SLP	/	/	/	/	/	/	/	/	/	/		0.15	0.00	2.78	/	/	/	/	/	/	/
4L8H	/	/	/	/	/	/	/	/	/	/		0.00	3.58	/	/	/	/	/	/	/	/
1MFJ	/	/	/	/	/	/	/	/	/	/		0.00	0.14	0.23	0.46	1.08	0.46	1.08	/	/	/
1A1T	/	/	/	/	/	/	/	/	/	/		0.00	0.20	0.33	0.93	8.00	/	/	/	/	/
6PK9	/	/	/	/	/	/	/	/	/	/		0.00	2.23	6.20	2.00	0.50	1.38	1.08	5.20	4.00	0.38
2RPT	/	/	/	/	/	/	/	/	/	/		0.00	0.43	4.00	4.18	3.45	12.00	/	/	/	/
1HLX	/	/	/	/	/	/	/	/	/	/		0.00	0.33	0.33	0.92	0.57	0.86	1.08	1.54	9.00	8.20
2RPK	/	/	/	/	/	/	/	/	/	/		0.00	0.38	0.54	0.38	0.23	0.38	1.62	0.67	1.00	/
2JPP	/	/	/	/	/	/	/	/	/	/		0.13	0.50	0.50	0.79	0.38	0.77	0.36	1.38	0.50	0.85
1U2A	/	/	/	/	/	/	/	/	/	/		0.00	0.33	0.57	0.62	0.79	/	/	/	/	/
2O33	/	/	/	/	/	/	/	/	/	/		0.00	12.00	0.27	0.33	1.36	1.40	/	/	/	/
2Y8W	/	/	/	/	/	/	/	/	/	/		0.15	1.33	0.67	1.36	2.00	2.00	/	/	/	/
5F5F	/	/	/	/	/	/	/	/	/	/		0.36	/	/	/	/	/	/	/	/	/
5ID6	/	/	/	/	/	/	/	/	/	/		0.00	0.20	0.36	/	/	/	/	/	/	/
5F5H	/	/	/	/	/	/	/	/	/	/		0.00	1.09	1.55	/	/	/	/	/	/	/
5L1Z	/	/	/	/	/	/	/	/	/	/		0.67	2.50	0.83	1.43	8.00	/	/	/	/	/
2FY1	/	/	/	/	/	/	/	/	/	/		0.00	0.20	0.33	1.86	12.00	12.00	/	/	/	/
1RKJ	/	/	/	/	/	/	/	/	/	/		0.13	0.36	0.50	0.31	10.60	0.62	9.67	/	/	/
1QWA	/	/	/	/	/	/	/	/	/	/		0.00	0.20	0.33	0.50	10.55	0.79	9.60	/	/	/

	DNA model											RNA model									
PDB	mfold MFE	subopt #2	subopt #3	subopt #4	subopt #5	subopt #6	subopt #7	subopt #8	subopt #9	subopt #10		mfold MFE	subopt #2	subopt #3	subopt #4	subopt #5	subopt #6	subopt #7	subopt #8	subopt #9	subopt #10
1SZY	/	/	/	/	/	/	/	/	/	/		0.00	0.38	0.38	0.54	5.00	/	/	/	/	/
2M21	/	/	/	/	/	/	/	/	/	/		0.00	1.00	1.57	0.92	2.14	/	/	/	/	/
1JOX	/	/	/	/	/	/	/	/	/	/		0.00	0.20	0.77	4.18	0.14	0.86	3.83	1.91	12.20	/
17RA	/	/	/	/	/	/	/	/	/	/		0.13	0.13	0.29	1.54	2.43	4.69	/	/	/	/
1D0U	/	/	/	/	/	/	/	/	/	/		0.00	0.20	0.40	0.33	0.47	12.20	0.57	0.33	0.71	11.40
2MFF	/	/	/	/	/	/	/	/	/	/		0.00	0.33	0.47	0.33	0.57	0.86	1.86	8.18	/	/
2MFG	/	/	/	/	/	/	/	/	/	/		0.00	0.33	0.33	0.79	1.64	6.55	1.54	2.23	/	/
6XWJ	/	/	/	/	/	/	/	/	/	/		0.00	0.87	0.80	/	/	/	/	/	/	/
1K2G	/	/	/	/	/	/	/	/	/	/		4.78	0.27	4.09	9.56	/	/	/	/	/	/
2W2H	/	/	/	/	/	/	/	/	/	/		0.62	0.71	0.77	0.93	9.00	6.27	/	/	/	/
4A4S	/	/	/	/	/	/	/	/	/	/		0.00	0.18	0.29	0.69	1.06	0.29	0.29	5.14	5.13	5.36
2GRW	/	/	/	/	/	/	/	/	/	/		0.00	0.20	0.33	1.00	0.86	1.71	/	/	/	/
2GV4	/	/	/	/	/	/	/	/	/	/		0.00	0.20	0.33	0.25	0.75	1.00	0.40	0.40	1.14	1.14
1N66	/	/	/	/	/	/	/	/	/	/		0.00	0.93	0.23	0.38	2.08	1.54	2.00	/	/	/
1OSW	/	/	/	/	/	/	/	/	/	/		0.00	1.00	0.50	0.64	0.75	0.67	1.71	1.50	0.77	3.83
2K66	/	/	/	/	/	/	/	/	/	/		0.00	0.18	0.29	0.82	1.06	0.50	0.75	0.50	/	/
2JSE	/	/	/	/	/	/	/	/	/	/		0.00	0.33	0.47	1.00	1.08	1.00	0.29	1.50	0.29	2.00
2KD8	/	/	/	/	/	/	/	/	/	/		0.00	0.18	0.29	4.00	0.88	8.00	4.77	4.38	9.00	3.43
2M5U	/	/	/	/	/	/	/	/	/	/		0.00	0.29	0.88	1.38	10.00	1.60	10.00	2.40	9.50	/
2GV3	/	/	/	/	/	/	/	/	/	/		0.20	8.18	10.10	/	/	/	/	/	/	/
1FJE	/	/	/	/	/	/	/	/	/	/		0.00	1.40	0.89	9.00	1.78	10.00	/	/	/	/
6F4H	/	/	/	/	/	/	/	/	/	/		0.00	0.38	1.33	0.77	1.67	2.17	3.73	5.60	0.55	7.60
1JUR	/	/	/	/	/	/	/	/	/	/		0.00	0.67	0.13	0.25	1.75	1.85	1.38	/	/	/
1PJY	/	/	/	/	/	/	/	/	/	/		0.00	0.29	0.29	0.53	0.18	0.18	0.41	0.75	1.00	0.50
1F9L	/	/	/	/	/	/	/	/	/	/		0.00	1.17	1.64	1.91	0.54	10.67	13.00	/	/	/
1K6G	/	/	/	/	/	/	/	/	/	/		0.00	0.50	1.07	0.80	1.07	9.00	8.08	1.73	3.36	/
2HNS	/	/	/	/	/	/	/	/	/	/		0.00	0.47	0.82	1.07	1.33	1.07	1.20	2.53	1.33	10.17
1K6H	/	/	/	/	/	/	/	/	/	/		0.00	0.29	0.35	0.59	0.47	2.62	0.82	1.60	/	/
2JYM	/	/	/	/	/	/	/	/	/	/		0.18	1.31	1.19	7.23	6.77	11.45	11.42	/	/	/
2MFE	/	/	/	/	/	/	/	/	/	/		0.00	0.33	0.33	0.69	10.00	10.43	10.57	0.79	1.21	8.20
1IKD	/	/	/	/	/	/	/	/	/	/		0.00	0.38	0.79	0.54	1.00	1.29	1.38	0.23	0.54	0.14
2G1W	/	/	/	/	/	/	/	/	/	/		3.55	4.55	4.91	4.00	4.67	5.82	5.90	5.55	5.23	2.83
2MFC	/	/	/	/	/	/	/	/	/	/		0.00	0.38	1.00	0.38	1.57	2.14	0.50	0.85	2.21	/
6KYV	/	/	/	/	/	/	/	/	/	/		0.00	0.29	0.18	0.29	0.93	1.33	12.00	1.57	0.75	0.47
1TJZ	/	/	/	/	/	/	/	/	/	/		0.00	0.33	1.17	1.58	2.08	13.00	13.00	0.77	1.50	5.18
2ANN	/	/	/	/	/	/	/	/	/	/		2.00	1.25	1.50	3.78	/	/	/	/	/	/
2N0R	/	/	/	/	/	/	/	/	/	/		1.22	0.80	1.10	1.10	1.50	0.60	2.80	0.45	4.70	2.78
1JTJ	/	/	/	/	/	/	/	/	/	/		0.00	0.57	0.43	9.60	5.60	1.00	9.67	7.33	4.33	5.67
1OW9	/	/	/	/	/	/	/	/	/	/		0.00	0.79	2.00	1.15	0.29	1.29	2.45	/	/	/
2N2P	/	/	/	/	/	/	/	/	/	/		0.15	0.93	10.00	0.57	5.00	/	/	/	/	/
2N2O	/	/	/	/	/	/	/	/	/	/		7.00	0.15	0.93	0.57	/	/	/	/	/	/
5UF3	/	/	/	/	/	/	/	/	/	/		0.00	0.11	0.35	0.18	0.47	0.29	0.71	0.94	/	/
5WQ1	/	/	/	/	/	/	/	/	/	/		0.00	0.18	0.29	1.00	1.63	8.00	2.25	8.00	1.57	7.17
1K5I	/	/	/	/	/	/	/	/	/	/		0.00	0.29	0.18	0.29	0.29	6.00	0.63	6.00	0.29	6.07
2ES5	/	/	/	/	/	/	/	/	/	/		0.16	1.44	0.37	2.88	0.50	0.44	1.19	0.56	11.64	1.44
2QH3	/	/	/	/	/	/	/	/	/	/		0.00	0.24	0.25	0.20	0.33	0.25	0.53	0.50	0.93	0.57
2M22	/	/	/	/	/	/	/	/	/	/		0.12	1.33	1.93	11.18	/	/	/	/	/	/
6GBM	/	/	/	/	/	/	/	/	/	/		0.20	0.13	2.21	2.13	0.33	0.47	/	/	/	/
1TLR	/	/	/	/	/	/	/	/	/	/		0.00	0.20	0.47	0.47	0.73	0.29	6.27	12.40	2.00	6.38
1BVJ	/	/	/	/	/	/	/	/	/	/		0.00	0.43	0.73	0.64	5.42	1.71	2.86	0.93	/	/
2PJP	/	/	/	/	/	/	/	/	/	/		0.21	0.11	1.06	0.89	1.53	5.50	1.44	1.50	1.75	1.82
1MFK	/	/	/	/	/	/	/	/	/	/		0.00	0.11	0.63	0.59	1.63	5.38	1.53	1.00	1.27	1.38
2UWM	/	/	/	/	/	/	/	/	/	/		0.28	0.21	1.31	1.47	1.13	13.73	12.15	11.86	1.06	9.94
2N3O	/	/	/	/	/	/	/	/	/	/		0.00	0.22	0.29	0.41	0.29	0.22	0.35	0.18	0.50	0.29
2RO2	/	/	/	/	/	/	/	/	/	/		0.00	0.11	0.29	0.29	0.65	0.29	0.29	14.14	0.18	0.29
1BGZ	/	/	/	/	/	/	/	/	/	/		0.38	0.00	1.53	0.25	1.76	0.86	0.60	1.93	2.80	10.55
1S2F	/	/	/	/	/	/	/	/	/	/		0.00	1.13	0.88	0.41	1.12	1.60	3.36	1.13	5.42	0.53
2M12	/	/	/	/	/	/	/	/	/	/		0.31	0.20	0.38	1.00	1.25	0.00	0.29	0.92	2.20	1.54
2N82	/	/	/	/	/	/	/	/	/	/		1.45	1.27	2.17	1.45	1.82	3.00	/	/	/	/

	DNA model											RNA model									
PDB	mfold MFE	subopt #2	subopt #3	subopt #4	subopt #5	subopt #6	subopt #7	subopt #8	subopt #9	subopt #10		mfold MFE	subopt #2	subopt #3	subopt #4	subopt #5	subopt #6	subopt #7	subopt #8	subopt #9	subopt #10
2N7X	/	/	/	/	/	/	/	/	/	/		0.00	0.29	0.13	1.14	1.14	2.38	/	/	/	/
3PHP	/	/	/	/	/	/	/	/	/	/		0.00	0.31	0.25	0.69	0.50	0.53	0.69	0.20	0.20	0.47
5NG6	/	/	/	/	/	/	/	/	/	/		0.00	2.00	0.67	2.29	3.42	7.00	0.36	0.20	0.45	0.73
3NVK	/	/	/	/	/	/	/	/	/	/		2.38	6.17	3.71	1.50	7.00	2.60	2.50	5.86	5.50	2.60
1A9N	/	/	/	/	/	/	/	/	/	/		0.00	2.46	1.50	1.50	10.82	3.17	0.64	2.08	1.92	2.23
5F9F	/	/	/	/	/	/	/	/	/	/		0.00	0.26	0.53	/	/	/	/	/	/	/
1KKS	/	/	/	/	/	/	/	/	/	/		0.13	0.20	0.27	0.64	0.77	1.38	0.62	1.08	0.57	0.77
2QH2	/	/	/	/	/	/	/	/	/	/		0.18	0.00	0.22	0.11	0.38	0.35	0.50	1.94	1.69	0.56
1MT4	/	/	/	/	/	/	/	/	/	/		0.00	0.24	0.44	0.47	0.71	1.60	2.40	0.87	1.73	1.43
2LK3	/	/	/	/	/	/	/	/	/	/		0.00	0.11	0.22	2.27	1.47	2.27	2.67	/	/	/
2HEM	/	/	/	/	/	/	/	/	/	/		0.00	0.33	0.33	2.77	1.54	2.00	1.54	1.69	2.38	2.15
1TFN	/	/	/	/	/	/	/	/	/	/		0.13	0.33	0.47	2.00	0.29	3.90	2.21	0.92	0.57	1.23
1RHT	/	/	/	/	/	/	/	/	/	/		0.13	0.40	0.33	2.00	0.57	4.10	2.21	0.92	0.57	1.23
2LV0	/	/	/	/	/	/	/	/	/	/		0.00	0.71	0.44	0.33	0.33	1.00	1.38	1.60	2.40	0.87
5UDZ	/	/	/	/	/	/	/	/	/	/		0.15	0.38	0.75	0.31	1.60	0.75	0.62	0.42	1.80	1.83
1NYB	/	/	/	/	/	/	/	/	/	/		0.00	0.11	0.33	0.29	0.22	0.44	0.67	0.18	0.47	0.82
1NC0	/	/	/	/	/	/	/	/	/	/		0.12	0.25	0.31	0.56	0.50	0.40	0.60	1.13	1.36	3.17
1SYZ	/	/	/	/	/	/	/	/	/	/		0.00	0.33	0.47	0.33	1.43	2.20	/	/	/	/
1E4P	/	/	/	/	/	/	/	/	/	/		0.00	1.08	1.29	2.17	2.83	0.79	1.00	1.15	/	/
6F4G	/	/	/	/	/	/	/	/	/	/		0.00	3.43	1.00	0.38	1.33	0.77	6.00	6.18	6.33	6.33
1M82	/	/	/	/	/	/	/	/	/	/		0.11	0.32	0.82	0.39	1.31	0.22	0.76	0.47	1.73	0.44
1QC8	/	/	/	/	/	/	/	/	/	/		0.22	0.39	0.50	2.50	2.67	0.93	/	/	/	/
6DU5	/	/	/	/	/	/	/	/	/	/		4.60	5.09	5.00	0.22	5.25	5.27	5.11	9.00	8.11	14.00
4QOZ	/	/	/	/	/	/	/	/	/	/		0.00	0.17	0.27	0.50	1.17	0.42	0.92	0.55	0.91	1.27
2L5Z	/	/	/	/	/	/	/	/	/	/		0.00	0.59	0.28	0.61	2.14	0.59	1.56	4.00	5.57	8.31
1QWB	/	/	/	/	/	/	/	/	/	/		0.00	1.00	0.62	1.23	0.92	0.27	13.00	0.45	12.00	/
4TV0	/	/	/	/	/	/	/	/	/	/		0.00	1.29	0.17	6.33	8.21	8.57	7.38	15.67	0.64	7.00
4BW0	/	/	/	/	/	/	/	/	/	/		0.00	0.50	1.00	0.45	1.36	1.36	12.50	12.20	13.00	/
6XH0	/	/	/	/	/	/	/	/	/	/		0.16	0.21	0.30	2.78	3.11	3.32	2.12	8.33	3.18	2.82
1FQZ	/	/	/	/	/	/	/	/	/	/		0.00	1.00	1.50	0.23	11.70	12.58	1.36	10.45	12.23	2.50
1XSG	/	/	/	/	/	/	/	/	/	/		0.00	0.14	0.30	0.33	0.24	0.29	1.21	0.48	0.09	0.14
1XSH	/	/	/	/	/	/	/	/	/	/		0.00	0.09	0.09	0.14	0.33	0.14	0.09	0.14	0.40	0.40
1F7F	/	/	/	/	/	/	/	/	/	/		0.00	0.18	0.14	0.33	0.24	0.09	0.14	1.11	0.40	0.60
2LQZ	/	/	/	/	/	/	/	/	/	/		0.11	0.11	6.20	0.22	0.39	2.18	0.28	0.39	0.22	0.35
2AHT	/	/	/	/	/	/	/	/	/	/		0.10	0.47	0.44	0.78	0.10	1.00	0.30	0.25	0.35	1.47
2LDL	/	/	/	/	/	/	/	/	/	/		0.11	0.29	1.33	0.28	1.67	0.39	1.00	1.44	1.33	1.38
2LJJ	/	/	/	/	/	/	/	/	/	/		0.11	1.13	1.13	1.81	0.75	1.28	5.13	7.93	1.59	10.86
2IXY	/	/	/	/	/	/	/	/	/	/		0.14	0.09	0.40	0.74	0.35	13.47	0.45	3.17	0.40	5.06
1FYO	/	/	/	/	/	/	/	/	/	/		0.24	0.11	1.50	0.22	0.44	0.56	1.05	1.20	0.47	1.31
1YSV	/	/	/	/	/	/	/	/	/	/		0.00	1.10	0.29	0.60	0.60	0.48	0.86	1.30	13.00	1.90
484D	/	/	/	/	/	/	/	/	/	/		0.00	1.18	1.26	0.33	0.44	0.44	0.35	1.24	0.71	0.59
5M0I	/	/	/	/	/	/	/	/	/	/		0.73	2.00	4.45	3.18	13.67	/	/	/	/	/
2NCI	/	/	/	/	/	/	/	/	/	/		0.22	0.28	0.44	0.41	0.53	0.35	0.94	0.50	1.06	0.94
2KMJ	/	/	/	/	/	/	/	/	/	/		0.00	1.62	2.84	1.77	0.90	0.70	0.90	2.59	3.16	8.00
2GIP	/	/	/	/	/	/	/	/	/	/		0.00	1.05	0.26	1.24	0.37	0.16	1.50	0.95	0.68	0.26
28SP	/	/	/	/	/	/	/	/	/	/		0.00	0.47	1.23	1.47	0.20	0.33	1.23	2.85	8.31	4.46
1ZBN	/	/	/	/	/	/	/	/	/	/		0.14	0.19	1.11	0.38	1.43	2.68	1.95	3.42	7.61	1.94
6SNJ	/	/	/	/	/	/	/	/	/	/		0.00	0.44	0.70	0.37	0.74	0.58	16.44	17.00	16.47	16.06
2LUN	/	/	/	/	/	/	/	/	/	/		0.24	0.53	0.37	0.74	0.95	14.13	14.00	0.56	0.56	0.47
2NC0	/	/	/	/	/	/	/	/	/	/		0.00	0.20	0.47	0.45	0.30	0.26	0.95	1.89	1.00	1.35
6VZC	/	/	/	/	/	/	/	/	/	/		0.00	0.24	0.33	0.60	0.26	0.80	0.26	0.42	1.00	0.74
6AAS	/	/	/	/	/	/	/	/	/	/		0.00	0.13	0.22	0.67	0.50	0.95	0.57	0.67	1.90	0.55
1OOA	/	/	/	/	/	/	/	/	/	/		0.00	0.21	0.35	0.50	0.71	7.47	0.72	0.42	0.55	0.22
5LSN	/	/	/	/	/	/	/	/	/	/		0.00	0.17	0.45	0.43	0.64	4.06	0.82	0.86	1.24	1.20
1JBT	/	/	/	/	/	/	/	/	/	/		1.00	1.13	1.07	3.40	1.80	5.64	1.07	2.47	0.86	1.43
5LM7	/	/	/	/	/	/	/	/	/	/		0.00	5.77	4.33	15.00	6.67	2.18	18.00	7.85	1.25	17.42
2JWV	/	/	/	/	/	/	/	/	/	/		0.00	0.21	0.35	0.50	0.71	0.72	0.42	0.55	0.22	9.08
3SN2	/	/	/	/	/	/	/	/	/	/		0.13	0.08	0.39	0.65	4.06	0.41	5.00	1.60	1.53	1.53
2M24	/	/	/	/	/	/	/	/	/	/		0.00	0.40	0.71	0.21	0.21	1.00	0.42	0.42	1.83	0.89

	DNA model											RNA model									
**PDB**	**mfold MFE**	**subopt #2**	**subopt #3**	**subopt #4**	**subopt #5**	**subopt #6**	**subopt #7**	**subopt #8**	**subopt #9**	**subopt #10**		**mfold MFE**	**subopt #2**	**subopt #3**	**subopt #4**	**subopt #5**	**subopt #6**	**subopt #7**	**subopt #8**	**subopt #9**	**subopt #10**
1NBR	/	/	/	/	/	/	/	/	/	/		0.13	1.45	1.23	0.27	1.05	8.41	8.37	1.43	1.16	0.59
1L1C	/	/	/	/	/	/	/	/	/	/		0.67	0.00	0.61	0.89	0.89	1.00	2.53	2.50	4.00	6.13
1ANR	/	/	/	/	/	/	/	/	/	/		0.11	0.21	0.35	2.72	3.00	3.26	1.88	2.94	2.47	11.33
2GIO	/	/	/	/	/	/	/	/	/	/		0.16	0.47	0.68	0.74	0.29	0.22	1.05	0.41	1.25	1.78
1F84	/	/	/	/	/	/	/	/	/	/		0.15	1.08	1.54	0.36	10.42	1.42	2.46	14.30	11.36	1.29
1SCL	/	/	/	/	/	/	/	/	/	/		1.00	1.07	1.13	1.80	1.40	1.07	1.93	2.47	5.69	0.86
2K5Z	/	/	/	/	/	/	/	/	/	/		0.00	0.86	0.18	1.52	1.62	0.95	0.29	0.36	0.80	2.05
1L1W	/	/	/	/	/	/	/	/	/	/		0.00	0.20	1.11	0.50	1.22	0.37	0.47	0.53	1.22	4.67
1EBS	/	/	/	/	/	/	/	/	/	/		0.00	0.10	0.24	0.84	1.00	1.33	5.41	1.29	0.53	0.74
6DU4	/	/	/	/	/	/	/	/	/	/		1.25	1.76	6.31	6.00	5.50	1.50	4.92	1.75	11.67	5.71
1HVU	/	/	/	/	/	/	/	/	/	/		6.53	4.00	8.71	6.38	5.76	7.67	12.86	5.58	/	/
1LDZ	/	/	/	/	/	/	/	/	/	/		0.00	1.89	0.50	2.11	1.24	1.10	3.50	4.59	11.88	8.38
1EKZ	/	/	/	/	/	/	/	/	/	/		0.07	1.55	0.88	5.62	18.00	13.71	13.45	/	/	/
5Y58	/	/	/	/	/	/	/	/	/	/		0.00	2.10	1.00	1.33	10.00	6.17	/	/	/	/
1RFR	/	/	/	/	/	/	/	/	/	/		0.00	8.00	11.40	12.18	16.00	16.00	/	/	/	/
1AUD	/	/	/	/	/	/	/	/	/	/		3.18	0.00	7.33	0.89	3.74	4.07	5.20	12.13	4.53	1.56
1KP7	/	/	/	/	/	/	/	/	/	/		0.00	5.53	6.75	11.69	4.38	8.50	4.11	13.63	2.69	2.65
6MCE	/	/	/	/	/	/	/	/	/	/		0.13	2.19	1.81	14.70	2.45	13.62	8.47	2.90	7.81	2.26
1NA2	/	/	/	/	/	/	/	/	/	/		0.00	1.38	6.20	13.40	2.27	14.00	11.18	4.15	/	/
1HWQ	/	/	/	/	/	/	/	/	/	/		0.11	8.56	9.19	7.00	2.25	9.25	15.00	1.88	/	/
1EBR	/	/	/	/	/	/	/	/	/	/		0.00	1.00	7.00	3.13	5.93	5.11	9.67	9.57	2.41	3.47
5KMZ	/	/	/	/	/	/	/	/	/	/		4.26	8.76	9.67	5.39	7.22	5.53	5.89	/	/	/
1MFY	/	/	/	/	/	/	/	/	/	/		0.18	0.30	6.07	1.73	6.20	3.19	1.61	10.07	11.94	16.08
1JO7	/	/	/	/	/	/	/	/	/	/		0.33	7.56	1.63	9.06	3.47	3.69	1.26	1.94	10.47	11.47
1YNC	/	/	/	/	/	/	/	/	/	/		0.10	1.15	1.35	2.39	2.32	7.67	3.00	/	/	/
1YNG	/	/	/	/	/	/	/	/	/	/		0.20	1.32	1.53	7.33	7.50	1.53	/	/	/	/
6HYK	/	/	/	/	/	/	/	/	/	/		0.00	0.61	2.65	4.85	3.29	2.27	9.13	15.20	16.13	13.88
2LDT	/	/	/	/	/	/	/	/	/	/		0.00	1.00	1.78	5.55	15.80	/	/	/	/	/
5UZT	/	/	/	/	/	/	/	/	/	/		0.62	1.33	0.80	1.33	0.80	2.00	6.93	19.18	19.43	13.86
5A18	/	/	/	/	/	/	/	/	/	/		0.52	1.64	1.10	0.76	6.71	5.16	20.08	16.53	/	/
1XHP	/	/	/	/	/	/	/	/	/	/		0.00	14.94	15.95	1.00	14.95	16.28	1.57	5.95	/	/
1Z31	/	/	/	/	/	/	/	/	/	/		0.00	1.09	0.82	2.37	13.38	14.71	2.38	9.81	2.05	5.68
2LI4	/	/	/	/	/	/	/	/	/	/		0.00	1.04	1.57	1.91	3.22	17.30	3.95	11.42	11.42	/
1KAJ	/	/	/	/	/	/	/	/	/	/		5.50	8.41	4.22	8.65	7.29	6.25	5.13	4.63	6.71	12.67
1KPD	/	/	/	/	/	/	/	/	/	/		6.35	7.74	8.11	6.67	5.53	5.88	6.38	4.24	6.94	/
2LBS	/	/	/	/	/	/	/	/	/	/		0.00	7.90	0.76	0.76	17.11	17.22	/	/	/	/
2LUP	/	/	/	/	/	/	/	/	/	/		0.00	7.90	0.76	0.76	16.95	17.11	/	/	/	/
1G70	/	/	/	/	/	/	/	/	/	/		0.00	2.05	2.48	6.65	2.84	11.37	1.50	7.94	12.42	8.67
2JXV	/	/	/	/	/	/	/	/	/	/		0.00	1.78	1.86	2.36	3.60	4.14	7.63	5.11	7.13	10.59
1EXY	/	/	/	/	/	/	/	/	/	/		0.00	1.09	3.00	13.68	8.11	7.78	1.71	2.74	6.09	12.33
3ID5	/	/	/	/	/	/	/	/	/	/		10.00	17.00	8.92	11.25	3.80	9.42	15.64	11.91	4.73	14.08
4OOG	/	/	/	/	/	/	/	/	/	/		0.00	5.00	5.23	5.00	3.52	18.00	10.50	14.78	18.68	14.32
1R2P	/	/	/	/	/	/	/	/	/	/		0.15	1.17	0.58	1.17	2.65	2.55	5.24	10.00	/	/
2F88	/	/	/	/	/	/	/	/	/	/		0.07	1.00	0.96	2.71	6.48	7.30	3.73	5.44	/	/
1R7W	/	/	/	/	/	/	/	/	/	/		0.00	4.09	2.18	3.27	7.38	3.83	1.62	12.95	11.48	7.45
1R7Z	/	/	/	/	/	/	/	/	/	/		0.00	2.18	8.86	3.18	3.57	12.95	1.62	11.48	7.45	12.10
2KPV	/	/	/	/	/	/	/	/	/	/		0.12	1.00	1.05	2.22	2.41	2.65	5.40	5.39	7.35	10.89
2JTP	/	/	/	/	/	/	/	/	/	/		0.08	0.88	8.05	13.00	9.80	13.29	/	/	/	/
1P5N	/	/	/	/	/	/	/	/	/	/		0.11	16.65	7.17	2.41	14.75	20.63	15.65	20.06	15.47	1.65
6SDY	/	/	/	/	/	/	/	/	/	/		0.00	0.70	0.70	7.91	2.70	15.54	9.20	2.75	14.71	16.44
4X4O	/	/	/	/	/	/	/	/	/	/		0.00	1.70	4.95	2.50	3.14	8.29	4.17	9.00	11.70	7.72
2EUY	/	/	/	/	/	/	/	/	/	/		0.13	9.67	7.55	11.00	17.06	14.59	4.50	10.12	13.68	9.23
1RNK	/	/	/	/	/	/	/	/	/	/		7.44	5.20	5.94	7.70	4.90	8.20	6.89	6.71	4.11	7.00
2RVO	/	/	/	/	/	/	/	/	/	/		0.00	2.29	2.96	1.61	2.55	0.91	5.14	/	/	/
2L3C	/	/	/	/	/	/	/	/	/	/		0.00	1.04	1.36	1.76	1.21	3.96	14.60	/	/	/
1T28	/	/	/	/	/	/	/	/	/	/		0.11	3.53	1.05	7.12	5.50	1.63	9.69	4.82	13.25	15.15
1ETF	/	/	/	/	/	/	/	/	/	/		0.00	2.57	10.76	4.48	2.80	1.10	1.82	2.24	5.17	13.52
4C4W	/	/	/	/	/	/	/	/	/	/		16.69	14.94	6.60	14.93	0.50	20.50	7.33	15.64	15.07	2.79
5FJ4	/	/	/	/	/	/	/	/	/	/		9.50	0.00	6.53	6.53	10.94	1.83	11.56	12.81	5.88	11.50

	DNA model											RNA model									
PDB	mfold MFE	subopt #2	subopt #3	subopt #4	subopt #5	subopt #6	subopt #7	subopt #8	subopt #9	subopt #10		mfold MFE	subopt #2	subopt #3	subopt #4	subopt #5	subopt #6	subopt #7	subopt #8	subopt #9	subopt #10
2PCV	/	/	/	/	/	/	/	/	/	/		0.60	5.50	3.89	6.44	5.89	2.11	3.78	2.70	9.67	3.22
2M57	/	/	/	/	/	/	/	/	/	/		0.26	1.23	15.56	13.41	3.29	1.76	15.78	14.42	11.78	13.50
2DRB	/	/	/	/	/	/	/	/	/	/		0.00	4.19	1.62	2.05	10.00	6.05	8.05	10.19	11.43	7.14
1ULL	/	/	/	/	/	/	/	/	/	/		0.00	3.82	2.80	8.83	13.24	1.33	3.09	4.30	4.80	8.83
2L3E	/	/	/	/	/	/	/	/	/	/		0.00	0.50	2.82	7.55	2.00	11.00	3.61	3.42	12.11	19.26
6BHJ	/	/	/	/	/	/	/	/	/	/		0.07	1.31	1.54	2.43	18.50	14.83	/	/	/	/
6SY6	/	/	/	/	/	/	/	/	/	/		1.38	11.09	3.45	3.08	3.69	15.40	8.40	13.33	9.80	15.67
2TPK	/	/	/	/	/	/	/	/	/	/		5.00	9.64	8.24	8.53	4.90	8.10	5.80	6.11	5.20	7.62
2N6S	/	/	/	/	/	/	/	/	/	/		0.10	2.22	1.74	1.84	19.22	3.85	1.15	1.15	3.41	/
1N8X	/	/	/	/	/	/	/	/	/	/		0.29	1.15	1.36	5.91	2.35	4.96	4.56	3.67	7.90	15.09
4X4P	/	/	/	/	/	/	/	/	/	/		0.00	1.70	4.95	2.50	8.36	6.00	11.38	3.72	12.89	13.43
2HW8	/	/	/	/	/	/	/	/	/	/		0.00	0.65	2.24	2.64	3.50	12.00	3.24	6.05	17.12	8.10
5KQE	/	/	/	/	/	/	/	/	/	/		0.19	1.16	7.77	6.43	2.92	2.29	4.68	2.12	1.04	3.00
2FDT	/	/	/	/	/	/	/	/	/	/		0.19	7.18	1.88	6.10	1.76	4.38	8.90	3.29	4.63	9.11
2LUB	/	/	/	/	/	/	/	/	/	/		0.00	0.82	0.74	2.13	2.52	3.07	3.73	4.14	1.76	9.21
2LHP	/	/	/	/	/	/	/	/	/	/		0.00	0.82	0.74	2.13	2.52	1.76	4.14	3.07	2.31	3.85
6U79	/	/	/	/	/	/	/	/	/	/		0.00	1.24	2.89	1.86	0.81	2.00	14.05	12.91	0.96	16.14
6DTD	/	/	/	/	/	/	/	/	/	/		0.35	1.22	2.74	0.61	5.52	4.71	5.00	4.21	/	/
1M5L	/	/	/	/	/	/	/	/	/	/		0.00	1.75	0.91	6.05	6.96	9.45	0.82	12.10	10.86	3.50
2KHY	/	/	/	/	/	/	/	/	/	/		0.11	0.95	11.71	4.20	9.87	9.87	3.87	3.41	5.35	4.45
4PDB	/	/	/	/	/	/	/	/	/	/		0.30	0.07	0.52	2.30	0.62	2.77	3.00	19.42	1.36	13.35
1TXS	/	/	/	/	/	/	/	/	/	/		0.00	2.00	0.73	2.92	9.04	7.25	1.00	18.18	3.91	9.44
6D12	/	/	/	/	/	/	/	/	/	/		19.00	0.23	18.73	17.50	1.17	18.19	1.96	1.00	17.42	2.00
2A9L	/	/	/	/	/	/	/	/	/	/		0.00	1.23	11.10	12.04	12.22	7.91	4.57	3.48	10.60	10.09
1B36	/	/	/	/	/	/	/	/	/	/		0.00	0.70	1.00	3.00	19.29	10.53	2.33	4.88	3.74	11.75
4KR7	/	/	/	/	/	/	/	/	/	/		0.17	3.08	2.56	6.50	1.46	4.10	8.90	8.84	11.67	7.68
4KR9	/	/	/	/	/	/	/	/	/	/		0.84	3.31	2.81	7.00	1.88	4.73	9.50	9.30	9.36	6.96
2MXL	/	/	/	/	/	/	/	/	/	/		0.00	1.40	5.52	1.64	5.43	5.91	11.33	6.76	12.80	11.95
2NBY	/	/	/	/	/	/	/	/	/	/		0.79	0.45	2.50	5.69	3.15	4.00	5.15	1.30	4.62	2.56
2HUA	/	/	/	/	/	/	/	/	/	/		0.17	0.12	2.27	14.82	1.09	1.32	15.18	0.87	2.77	14.36
4PMI	/	/	/	/	/	/	/	/	/	/		0.54	0.00	0.74	3.96	4.21	8.33	1.50	8.39	1.46	5.04
2NBZ	/	/	/	/	/	/	/	/	/	/		0.00	0.48	1.64	4.74	4.26	4.70	2.38	4.88	4.41	3.69
5W1H	/	/	/	/	/	/	/	/	/	/		10.08	8.79	28.67	8.86	2.83	8.18	6.73	18.14	8.57	24.31
4M6D	/	/	/	/	/	/	/	/	/	/		10.10	4.36	2.39	5.80	10.24	9.45	1.35	14.05	9.05	6.65
5V17	/	/	/	/	/	/	/	/	/	/		0.00	5.15	0.90	4.67	3.10	5.00	15.72	2.50	4.19	5.28
1A51	/	/	/	/	/	/	/	/	/	/		0.38	2.17	0.46	7.22	3.77	4.72	4.39	1.18	10.52	10.04
1ZC5	/	/	/	/	/	/	/	/	/	/		0.00	0.84	4.13	1.53	4.54	2.34	5.43	13.04	9.37	5.38
6W3M	/	/	/	/	/	/	/	/	/	/		0.14	16.00	0.54	2.68	2.16	2.16	16.92	16.46	11.33	7.23
5WLH	/	/	/	/	/	/	/	/	/	/		10.08	8.79	28.67	8.86	2.83	8.18	6.73	18.14	8.57	24.31
2N6T	/	/	/	/	/	/	/	/	/	/		0.24	1.12	2.33	3.28	7.15	3.25	2.46	1.15	3.96	7.46
1MNX	/	/	/	/	/	/	/	/	/	/		0.00	0.54	9.55	7.70	1.04	2.64	13.05	5.27	16.62	6.28
2L2J	/	/	/	/	/	/	/	/	/	/		0.00	0.56	1.53	3.17	3.67	0.59	5.19	3.03	5.00	2.81
2FEY	/	/	/	/	/	/	/	/	/	/		0.24	3.67	8.57	1.00	6.00	10.69	10.00	1.23	2.58	12.57
1CQ5	/	/	/	/	/	/	/	/	/	/		0.25	0.48	1.00	3.13	0.56	6.71	7.16	7.42	1.65	4.95
1CQL	/	/	/	/	/	/	/	/	/	/		0.08	0.58	0.79	2.83	0.38	6.28	7.20	7.50	1.42	5.00
2ADT	/	/	/	/	/	/	/	/	/	/		0.00	2.14	0.73	3.00	2.87	1.87	7.55	4.06	3.96	4.36
2N6X	/	/	/	/	/	/	/	/	/	/		0.22	0.45	2.07	2.07	1.07	3.54	7.84	3.29	0.90	7.60
1A60	/	/	/	/	/	/	/	/	/	/		1.80	4.08	3.61	3.08	4.15	6.48	4.60	16.50	4.04	4.68
1P6V	/	/	/	/	/	/	/	/	/	/		0.00	0.38	10.40	2.33	10.48	7.07	9.56	19.28	4.30	1.22
1Z2J	/	/	/	/	/	/	/	/	/	/		0.00	1.09	5.91	10.34	1.15	23.00	1.35	5.03	4.67	15.77
2PXL	/	/	/	/	/	/	/	/	/	/		0.00	6.48	0.67	14.04	8.92	0.63	9.56	0.28	10.04	8.21
2MTJ	/	/	/	/	/	/	/	/	/	/		0.13	0.44	0.39	1.36	10.03	1.17	1.26	1.77	1.85	10.79
1S03	/	/	/	/	/	/	/	/	/	/		0.19	0.33	2.45	1.24	2.58	0.76	5.29	2.35	1.03	3.00
5KH8	/	/	/	/	/	/	/	/	/	/		5.57	4.88	3.28	3.92	7.28	6.00	4.24	5.70	8.13	4.56
1YMO	/	/	/	/	/	/	/	/	/	/		5.67	13.09	7.84	12.57	14.41	6.67	13.15	6.07	13.00	6.33
2VPL	/	/	/	/	/	/	/	/	/	/		0.12	1.82	1.26	1.83	2.48	0.68	2.45	2.71	5.30	4.13
2KUV	/	/	/	/	/	/	/	/	/	/		0.11	0.31	1.16	0.44	1.12	2.36	1.19	2.85	1.22	3.44
2KUU	/	/	/	/	/	/	/	/	/	/		0.11	0.31	0.44	1.21	0.97	0.74	3.00	2.52	8.75	1.42
2KUR	/	/	/	/	/	/	/	/	/	/		0.11	0.31	0.44	1.72	2.59	1.06	1.06	2.14	2.21	2.06

	DNA model											RNA model									
PDB	mfold MFE	subopt #2	subopt #3	subopt #4	subopt #5	subopt #6	subopt #7	subopt #8	subopt #9	subopt #10		mfold MFE	subopt #2	subopt #3	subopt #4	subopt #5	subopt #6	subopt #7	subopt #8	subopt #9	subopt #10
2KE6	/	/	/	/	/	/	/	/	/	/		0.16	0.37	1.24	0.51	1.22	2.50	1.28	3.00	1.31	3.61
2K95	/	/	/	/	/	/	/	/	/	/		13.35	8.23	12.88	13.00	7.00	5.67	13.29	11.92	12.31	13.30
2KUW	/	/	/	/	/	/	/	/	/	/		0.11	0.32	1.19	0.37	1.16	2.44	1.22	2.94	1.26	3.33
2M8K	/	/	/	/	/	/	/	/	/	/		5.52	5.83	5.26	5.06	4.44	15.57	21.75	8.26	14.39	/
4C7O	/	/	/	/	/	/	/	/	/	/		0.25	1.57	17.00	5.55	1.50	1.38	16.03	2.24	19.56	18.00
2PXT	/	/	/	/	/	/	/	/	/	/		0.00	0.58	0.55	2.13	0.24	1.79	1.90	1.03	4.17	4.54
2LU0	/	/	/	/	/	/	/	/	/	/		0.00	3.27	1.38	3.00	7.30	1.41	2.24	5.48	8.56	11.48
6MXQ	/	/	/	/	/	/	/	/	/	/		0.00	2.38	3.00	4.26	3.39	5.03	7.59	0.65	6.22	5.77
1U63	/	/	/	/	/	/	/	/	/	/		0.11	1.71	1.18	2.00	0.64	2.64	2.91	2.33	5.47	4.22
2PXQ	/	/	/	/	/	/	/	/	/	/		0.00	0.58	0.55	2.13	0.24	20.19	1.03	4.17	20.82	25.07
2PXP	/	/	/	/	/	/	/	/	/	/		0.00	0.58	0.55	2.13	0.24	4.17	1.03	9.14	4.54	2.42
2PXD	/	/	/	/	/	/	/	/	/	/		0.00	0.58	0.55	2.13	0.24	9.14	9.71	10.14	11.00	1.03
2PXF	/	/	/	/	/	/	/	/	/	/		0.00	0.58	0.55	2.13	0.24	4.17	1.03	2.42	10.72	5.78
2PXE	/	/	/	/	/	/	/	/	/	/		0.13	0.76	0.72	2.41	0.39	19.40	1.24	4.61	20.12	25.08
2PXK	/	/	/	/	/	/	/	/	/	/		0.13	0.76	0.72	2.41	0.39	11.54	13.29	20.96	10.23	4.61
2PXU	/	/	/	/	/	/	/	/	/	/		0.00	0.58	0.55	2.13	0.24	4.17	1.03	4.54	2.42	6.61
2PXB	/	/	/	/	/	/	/	/	/	/		0.07	0.71	0.68	2.43	0.33	14.14	1.21	4.70	5.22	13.14
2PXV	/	/	/	/	/	/	/	/	/	/		0.13	0.76	0.72	2.41	0.39	14.20	14.31	13.80	1.24	4.61
6IV9	/	/	/	/	/	/	/	/	/	/		0.22	5.62	29.42	25.39	/	/	/	/	/	/
6IV8	/	/	/	/	/	/	/	/	/	/		0.22	5.62	29.42	25.39	34.56	/	/	/	/	/
2MHI	/	/	/	/	/	/	/	/	/	/		0.21	3.17	3.66	7.84	11.42	9.09	9.42	5.75	/	/
2N4L	/	/	/	/	/	/	/	/	/	/		0.29	2.64	3.31	3.37	1.89	7.24	7.21	19.38	16.76	32.32
1P5M	/	/	/	/	/	/	/	/	/	/		0.08	1.06	1.08	2.38	25.08	10.94	5.63	17.18	10.84	/
2KZL	/	/	/	/	/	/	/	/	/	/		0.00	0.61	4.93	9.07	9.33	2.35	7.26	8.17	11.54	10.27
2HGH	/	/	/	/	/	/	/	/	/	/		0.64	0.38	4.48	12.13	11.67	10.32	10.10	10.83	11.52	10.19
6NOA	/	/	/	/	/	/	/	/	/	/		0.00	0.82	7.67	35.50	2.28	3.86	8.89	44.45	20.35	/
2LC8	/	/	/	/	/	/	/	/	/	/		7.65	9.27	11.55	10.00	7.06	11.52	13.90	8.75	9.69	14.41
6MCF	/	/	/	/	/	/	/	/	/	/		0.63	0.98	3.40	4.38	11.68	17.14	5.00	8.24	21.71	16.59
5IEM	/	/	/	/	/	/	/	/	/	/		0.57	0.90	4.21	5.58	17.47	4.05	11.22	21.97	7.33	16.63
4M4O	/	/	/	/	/	/	/	/	/	/		2.25	1.03	4.82	2.89	5.05	5.71	4.69	9.47	8.65	13.27
6DB8	/	/	/	/	/	/	/	/	/	/		1.06	2.05	5.95	3.71	5.00	2.50	6.11	2.14	7.49	6.09
4U7U	/	/	/	/	/	/	/	/	/	/		29.42	30.73	27.96	31.78	30.91	26.64	22.73	30.00	29.36	18.00
1UN6	/	/	/	/	/	/	/	/	/	/		0.05	10.54	4.41	12.54	17.25	6.39	6.91	11.70	17.11	4.23
2N3Q	/	/	/	/	/	/	/	/	/	/		0.10	1.75	2.46	9.05	5.58	4.73	12.30	1.63	4.54	8.49
3EGZ	/	/	/	/	/	/	/	/	/	/		0.12	6.19	4.13	13.29	4.29	6.39	13.13	13.71	14.29	3.81
5WT1	/	/	/	/	/	/	/	/	/	/		13.05	0.89	12.62	5.41	7.74	1.71	7.36	1.81	5.29	11.26
2NC1	/	/	/	/	/	/	/	/	/	/		0.51	5.89	5.93	17.74	2.56	5.72	3.65	4.23	3.07	6.86
5HR6	/	/	/	/	/	/	/	/	/	/		10.08	1.82	5.87	0.55	9.82	8.21	9.24	8.93	9.60	7.00
2N6W	/	/	/	/	/	/	/	/	/	/		0.28	1.26	1.48	4.24	3.73	9.19	10.61	21.46	7.70	34.45
2MQT	/	/	/	/	/	/	/	/	/	/		0.12	2.17	13.55	3.20	5.33	2.94	6.72	5.50	7.04	19.48
6U8D	/	/	/	/	/	/	/	/	/	/		0.08	7.45	6.54	4.51	3.53	6.28	12.90	5.54	13.17	13.35
3EPJ	/	/	/	/	/	/	/	/	/	/		2.58	0.83	9.19	9.05	8.74	5.54	4.85	7.60	8.31	3.54
5HR7	/	/	/	/	/	/	/	/	/	/		10.08	1.82	0.55	10.00	5.87	9.24	11.05	11.10	8.93	7.00
5V6X	/	/	/	/	/	/	/	/	/	/		0.34	10.02	1.18	7.00	11.13	2.12	9.48	4.33	9.00	7.67
2N8V	/	/	/	/	/	/	/	/	/	/		3.04	4.72	13.77	4.61	11.30	6.35	11.33	13.87	5.10	6.63
2DET	/	/	/	/	/	/	/	/	/	/		10.03	2.17	5.61	0.78	7.46	9.09	10.13	9.82	9.81	10.72
1KXK	/	/	/	/	/	/	/	/	/	/		0.26	9.95	1.64	10.30	4.29	13.37	2.46	14.88	8.50	20.34
2DU6	/	/	/	/	/	/	/	/	/	/		0.75	9.50	4.47	1.21	8.44	6.29	6.29	6.85	7.65	7.84
2DU3	/	/	/	/	/	/	/	/	/	/		6.40	0.81	6.31	8.60	4.76	8.82	1.34	8.93	5.54	2.17
2DU5	/	/	/	/	/	/	/	/	/	/		0.75	9.70	6.29	4.47	8.44	1.21	6.85	6.38	7.80	7.65
2ZZN	/	/	/	/	/	/	/	/	/	/		0.60	5.77	1.50	9.67	10.72	1.54	6.09	2.23	8.11	7.91
2L3J	/	/	/	/	/	/	/	/	/	/		0.00	5.98	4.91	0.80	0.80	4.87	1.74	1.40	18.32	16.74
2MS0	/	/	/	/	/	/	/	/	/	/		3.54	8.30	0.61	9.41	8.85	12.28	7.32	13.70	4.64	7.65
5TF6	/	/	/	/	/	/	/	/	/	/		17.97	22.97	28.91	2.39	3.30	27.92	16.83	29.15	7.78	1.76
4YVK	/	/	/	/	/	/	/	/	/	/		13.23	9.45	3.80	10.73	10.02	9.11	5.43	13.02	15.55	7.65
4YVI	/	/	/	/	/	/	/	/	/	/		9.69	13.09	3.80	4.12	10.98	5.41	7.65	10.17	7.47	11.00
4YVJ	/	/	/	/	/	/	/	/	/	/		9.45	13.23	3.80	9.11	10.73	10.77	7.65	6.80	5.43	7.47
2AKE	/	/	/	/	/	/	/	/	/	/		0.00	1.10	1.91	9.86	8.66	11.45	7.69	12.32	8.51	11.83
4ZT0	/	/	/	/	/	/	/	/	/	/		6.52	8.89	4.97	8.62	14.28	7.22	12.55	13.66	7.13	11.97

	DNA model											RNA model									
PDB	mfold MFE	subopt #2	subopt #3	subopt #4	subopt #5	subopt #6	subopt #7	subopt #8	subopt #9	subopt #10		mfold MFE	subopt #2	subopt #3	subopt #4	subopt #5	subopt #6	subopt #7	subopt #8	subopt #9	subopt #10
1DRZ	/	/	/	/	/	/	/	/	/	/		10.11	12.93	9.45	15.16	16.57	16.42	9.80	13.20	14.51	15.33
2ZNI	/	/	/	/	/	/	/	/	/	/		0.10	10.20	5.90	6.74	14.60	7.17	5.97	2.08	11.74	10.36
1EUQ	/	/	/	/	/	/	/	/	/	/		0.59	9.95	2.41	7.65	2.56	3.38	4.46	3.69	3.90	8.29
2MF0	/	/	/	/	/	/	/	/	/	/		0.11	13.66	16.42	10.08	5.61	8.17	4.81	10.81	15.50	13.92
3WC1	/	/	/	/	/	/	/	/	/	/		6.93	7.34	9.20	8.48	0.62	7.93	6.53	8.77	19.35	8.27
4X0A	/	/	/	/	/	/	/	/	/	/		0.64	1.59	6.51	1.71	6.71	3.59	1.58	5.87	2.08	8.49
3WFQ	/	/	/	/	/	/	/	/	/	/		0.83	1.95	6.08	1.21	4.92	3.70	1.05	6.28	2.50	8.95
5WT3	/	/	/	/	/	/	/	/	/	/		0.77	7.49	6.68	1.39	14.86	1.56	3.38	8.05	9.76	1.93
5VW1	/	/	/	/	/	/	/	/	/	/		0.15	5.41	2.34	6.38	3.43	11.68	7.06	6.00	13.21	6.83
2ZM5	/	/	/	/	/	/	/	/	/	/		0.60	8.55	8.91	1.59	10.84	2.27	3.96	1.49	4.82	10.19
3FOZ	/	/	/	/	/	/	/	/	/	/		0.60	8.55	8.91	3.96	10.84	2.27	1.49	10.19	4.82	14.59
3TUP	/	/	/	/	/	/	/	/	/	/		8.84	7.63	8.91	10.93	7.20	0.61	9.50	9.30	10.26	6.28
5D6G	/	/	/	/	/	/	/	/	/	/		0.68	7.72	2.45	10.25	8.05	2.89	10.95	23.22	5.75	6.97
4YCO	/	/	/	/	/	/	/	/	/	/		0.60	8.55	8.91	1.59	10.84	2.27	3.96	1.49	4.82	8.47
3WC2	/	/	/	/	/	/	/	/	/	/		10.13	11.06	10.70	8.33	11.16	6.89	0.55	7.65	11.67	2.40
3WFS	/	/	/	/	/	/	/	/	/	/		0.00	1.00	6.00	1.18	6.07	2.71	0.85	5.51	1.60	8.32
1GTR	/	/	/	/	/	/	/	/	/	/		10.86	11.57	0.54	12.29	2.51	7.85	3.64	8.15	9.58	8.86
2DER	/	/	/	/	/	/	/	/	/	/		10.95	2.45	1.13	10.00	9.70	10.41	9.42	7.83	7.06	5.48
4YYE	/	/	/	/	/	/	/	/	/	/		3.73	7.39	0.80	1.86	8.06	7.68	10.91	5.84	4.71	10.39
3AKZ	/	/	/	/	/	/	/	/	/	/		9.22	12.74	3.85	9.18	12.95	3.83	10.70	5.30	9.52	6.88
2IHX	/	/	/	/	/	/	/	/	/	/		0.04	3.57	3.26	3.02	0.63	14.88	3.92	4.96	15.04	6.20
2DR2	/	/	/	/	/	/	/	/	/	/		0.15	1.31	2.15	8.68	9.26	10.49	6.73	11.00	9.14	12.59
4TZV	/	/	/	/	/	/	/	/	/	/		3.79	0.60	3.84	5.10	6.53	11.63	7.22	12.26	18.45	12.53
5X6B	/	/	/	/	/	/	/	/	/	/		0.77	4.93	1.33	10.26	10.16	1.39	6.49	2.49	8.38	8.26
2ZUE	/	/	/	/	/	/	/	/	/	/		5.09	2.76	2.17	10.79	0.79	10.87	7.74	8.49	7.02	3.11
3WQZ	/	/	/	/	/	/	/	/	/	/		9.82	0.45	11.19	2.02	6.52	11.92	9.17	3.80	6.51	9.13
4WC2	/	/	/	/	/	/	/	/	/	/		0.55	1.60	6.60	1.55	6.49	3.29	1.41	6.10	2.13	8.27
1N77	/	/	/	/	/	/	/	/	/	/		0.75	2.00	6.14	9.23	7.14	3.93	5.73	14.75	8.95	2.26
3WQY	/	/	/	/	/	/	/	/	/	/		9.90	0.50	11.11	2.11	11.59	6.70	9.26	12.77	3.93	6.27
1FFY	/	/	/	/	/	/	/	/	/	/		10.90	0.51	4.04	6.76	11.40	7.81	9.80	13.98	12.91	8.24
1EIY	/	/	/	/	/	/	/	/	/	/		0.49	9.72	1.81	11.38	10.00	10.28	10.15	10.44	2.24	8.84
4WC3	/	/	/	/	/	/	/	/	/	/		0.55	1.60	6.60	1.55	6.49	3.29	1.41	6.10	2.13	8.27
2K4C	/	/	/	/	/	/	/	/	/	/		9.21	8.67	9.16	1.57	3.70	8.91	6.90	2.42	2.73	16.30
4WJ3	/	/	/	/	/	/	/	/	/	/		6.91	0.55	2.27	6.28	9.89	9.45	3.60	9.18	7.32	6.55
3A2K	/	/	/	/	/	/	/	/	/	/		7.83	9.35	0.57	11.70	8.39	1.93	17.88	9.60	2.48	3.89
5CCB	/	/	/	/	/	/	/	/	/	/		2.35	0.18	9.78	6.84	11.85	1.62	4.49	10.47	11.62	1.53
1P5P	/	/	/	/	/	/	/	/	/	/		9.38	0.10	14.24	8.48	1.75	12.85	12.07	11.45	11.28	11.04
4X0B	/	/	/	/	/	/	/	/	/	/		0.31	0.78	4.73	1.83	6.16	2.92	1.24	3.92	1.19	8.11
3AMT	/	/	/	/	/	/	/	/	/	/		4.14	1.66	7.00	9.45	7.66	0.50	7.07	6.12	12.66	10.44
3U4M	/	/	/	/	/	/	/	/	/	/		13.13	3.02	13.74	1.93	3.13	8.83	24.91	11.73	5.02	13.07
6B14	/	/	/	/	/	/	/	/	/	/		10.31	8.87	10.04	8.25	4.75	5.56	2.52	12.39	8.52	12.62
6B3K	/	/	/	/	/	/	/	/	/	/		10.16	11.39	10.31	9.80	7.61	8.60	12.61	6.96	2.52	10.11
2ZZM	/	/	/	/	/	/	/	/	/	/		11.05	14.11	12.05	11.93	11.86	12.00	9.89	5.85	6.83	13.33
3A3A	/	/	/	/	/	/	/	/	/	/		0.48	8.00	1.51	5.14	8.60	4.40	6.45	1.93	2.95	12.15
3K0J	/	/	/	/	/	/	/	/	/	/		5.13	14.06	7.47	0.55	7.65	2.39	8.51	11.19	3.46	1.53
1WZ2	/	/	/	/	/	/	/	/	/	/		10.96	10.24	6.52	11.61	10.38	4.25	5.68	12.94	6.47	11.96
5XBL	/	/	/	/	/	/	/	/	/	/		4.68	3.43	6.95	6.83	6.74	10.67	5.98	7.45	17.00	4.82
3ADB	/	/	/	/	/	/	/	/	/	/		0.43	7.33	7.13	8.52	6.40	11.26	6.03	4.42	3.64	3.10
2N7M	/	/	/	/	/	/	/	/	/	/		0.00	9.73	3.00	1.00	3.24	2.20	5.12	3.95	9.66	2.28
3W1K	/	/	/	/	/	/	/	/	/	/		0.37	8.98	4.92	4.59	12.24	10.89	9.62	6.17	10.12	7.69
3KTW	/	/	/	/	/	/	/	/	/	/		0.13	5.59	7.08	2.77	1.24	7.08	10.26	6.06	6.95	6.92
2V3C	/	/	/	/	/	/	/	/	/	/		0.03	0.41	1.89	2.52	6.56	7.36	2.05	7.52	3.00	6.50
6JXM	/	/	/	/	/	/	/	/	/	/		0.43	1.64	1.84	6.43	9.04	7.42	10.33	6.76	11.04	9.39
1LNG	/	/	/	/	/	/	/	/	/	/		0.03	0.41	6.41	1.98	7.40	7.33	2.52	8.20	3.17	6.98
3W3S	/	/	/	/	/	/	/	/	/	/		8.97	0.41	4.25	6.00	14.46	8.75	8.83	8.32	10.72	11.27
6MJ0	/	/	/	/	/	/	/	/	/	/		14.32	14.53	10.63	15.28	14.67	14.07	5.64	13.57	14.78	4.25
1S9S	/	/	/	/	/	/	/	/	/	/		0.03	0.61	5.04	1.28	1.77	5.44	0.57	4.93	3.65	2.22
2XXA	/	/	/	/	/	/	/	/	/	/		6.64	0.03	2.82	5.82	3.66	8.66	5.78	7.94	7.11	13.57
2KRL	/	/	/	/	/	/	/	/	/	/		1.49	6.03	2.27	4.26	15.00	23.98	15.15	25.10	8.41	10.05

	DNA model											RNA model									
**PDB**	**mfold MFE**	**subopt #2**	**subopt #3**	**subopt #4**	**subopt #5**	**subopt #6**	**subopt #7**	**subopt #8**	**subopt #9**	**subopt #10**		**mfold MFE**	**subopt #2**	**subopt #3**	**subopt #4**	**subopt #5**	**subopt #6**	**subopt #7**	**subopt #8**	**subopt #9**	**subopt #10**
7K1Z	/	/	/	/	/	/	/	/	/	/		2.16	19.58	19.66	17.55	16.83	16.88	16.87	1.72	16.23	0.89
2NBX	/	/	/	/	/	/	/	/	/	/		0.87	2.85	1.49	5.40	2.51	14.46	5.87	3.16	6.28	10.44
2LKR	/	/	/	/	/	/	/	/	/	/		0.00	3.25	1.94	1.09	1.12	4.03	5.56	3.60	10.88	17.25
4P3E	/	/	/	/	/	/	/	/	/	/		1.28	6.48	3.70	3.45	8.87	15.18	5.33	14.38	17.53	11.06
3IVK	/	/	/	/	/	/	/	/	/	/		4.58	5.05	8.60	13.00	6.37	12.88	11.70	4.81	8.81	7.07
3NDB	/	/	/	/	/	/	/	/	/	/		0.06	1.65	3.40	17.86	6.21	10.88	14.01	5.13	10.83	4.62
2N1Q	/	/	/	/	/	/	/	/	/	/		8.30	9.87	11.93	9.80	0.41	15.20	5.06	5.71	15.92	8.55
2R8S	/	/	/	/	/	/	/	/	/	/		4.40	0.66	5.77	9.01	13.61	28.31	12.99	5.13	8.88	17.38
4P8Z	/	/	/	/	/	/	/	/	/	/		6.90	6.61	7.26	3.55	6.05	6.77	6.18	5.29	7.21	5.63
1GRZ	/	/	/	/	/	/	/	/	/	/		2.58	3.11	2.22	4.61	6.83	13.20	14.90	12.49	12.97	17.80
5IWA	/	/	/	/	/	/	/	/	/	/		42.31	49.39	44.85	20.76	42.97	54.17	58.31	42.48	40.58	78.59
