# Supplementary material for: Comparative Study of Single-stranded Oligonucleotides Secondary Structure Prediction Tools
Source: BMC Bioinformatics. 2023 Nov 8;24:422. doi: 10.1186/s12859-023-05532-5 (PMC10634105; doi:10.1186/s12859-023-05532-5)
Supplement: Supplementary file 6 — Additional file 6. Predicted secondary structure for RNAfold under RNA (Turner (2004)) and DNA (Mathews (2004)) model in the dot-bracket notation. The PDB code is reported in the first column. "/" characters indicate either structures predicted as unfolded or software failure during the computation or sequences for which the parameters were not applied. [file 12859_2023_5532_MOESM6_ESM.pdf]

Additional File 6. Predicted secondary structures for RNAfold under RNA (Turner (2004)) and DNA (Mathews (2004)) model in the dotbracket notation. The PDB code is reported in the first column. "/" characters indicate either structures predicted as unfolded or software failure during the computation or sequences for which the parameters were not applied.

DNA model

PDB	RNAfold MFE	subopt #2	subopt #3	subopt #4	subopt #5	subopt #6	subopt #7	subopt #8	subopt #9	subopt #10
1AC7	((((((....))))))	.(((((....))))).	(((((......)))))	..((((....))))..	((((.(....).))))	.((((......)))).	....	(.((((....)))).)	((((........))))	.(((.(....).))).
1B4Y	..........((((((....))))))....	..........(((((......)))))....	....((((((........))))))....	..........(((((....)))))....	..........(((.((....)).)))....	..........((.(((....))).))....	....((((..............))))....	..........((((........))))....	....(((((............)))))....	....((((.((........)).))))....
1BJH	((((...))))	....	.(((...))).	(((.....)))	..((...))..	.(....)....	.(.....)...	((.......))	.((.....)).	.(...).....
1ECU	((((((((...))))))))	(((((((.....)))))))	.(((((((...))))))).	((((((........))))))	((((((.(...).))))))	(((((.((...)).)))))	.((((((.....)))))).	(((((((....)).)))))	(((((((....).))))))	((((((.(....)))))))
1EN1	..(((.....)))....	(.(((.....))))....	...(((....)))....	....	((((......))))....	..((((....))).)...	.........((...)).	(.(((.....)))..)..	...((......))....	.(((......)))....
1EZN	((((((((((....)))((((....))..)))))))	((((((.(((.....)))((.....)).)))))))	((((((.((((...))))((.....)).)))))))	((((((((((....))).((((....)))))))))	(((((((((((....)))((.....))..)))))))	((((((((((....)))..((.....)).)))))))	((((((((((....)))..........)))))))	((((((.((....))((((....)))).)))))))	.((((((((....)))(((....))).)))))).	((((((........(((((....)))))))))))
1JVE	((((((((((((...))))))))))))	((((((((((((.....))))))))))	.((((((((((((...)))))))))))).	(((((((((.(((...))).))))))))	(((((((.((((...)))).)))))))	((((((((((.((...)).))))))))	((((((.(((((...))))).))))))	(((.((((((((...)))))))).)))	(((((.((((((...)))))).))))	((((.(((((((...))))))).))))
1LA8	(((((...)))))	((((.....))))	.((((...)))).	((((....).)))	(((.(....))))	.(((.....))).	..(((...)))..	(((.......)))	(((.(...).)))	....
1NGO	((((((((((((.....))))))))))))	(((((.(((((.....))))).)))))	((((.((((((.....)))))).))))	((((..(((((.....)))))))))..	((((((.((((.....)))).)))))	((((..(((((.....)))))..))))	.((((((((((.....)))))))))).	......(((((.....)))))......	.((((.(((((.....))))).)))).	(((((((((.......)))))))))
1NGU	((((..(((((.....)))))..))))	.(((..(((((.....)))))..))).	......(((((.....)))))).....	((((..((((.......))))..))))	............(((.....))))).	((((...((((.....))))...))))	..(((.(((((.....)))))...)))	..((..(((((.....)))))..))..	(((...(((((.....)))))...)))	.......(((.....)))).......
1OSB	.((((((....))))))........	(((((((....))))))).......	.((((((....))))))(...)...	.((((((....)))))).(...)..	.((((((....)))))).(.....)	(((((((....)))))))(...)..	(((((((....)))))))(.....)	((((((....))))))..).....	..(((((....))))).........	((((((....))))))...)....
1P0U	(((((...)))))	((((.....))))	.((((...)))).	....	(((.......)))	(((.(...).)))	((.((...)).))	.(((.....))).	..(((...)))..	....((....)).
1PQT	....	((...))	..(...)	.(...).	(.....)	/	/	/	/	/
1SNJ	((((((((((....)))(((....)))..)))))))	.((((..(((....))))))).(((((....)))).	.((((.((.....))..))))..((((....)))).	.((((((((....)))(((....)))..)))))).	((((((((((....)))((.....))..)))))))	((((((.(((....)))(((....)))...))))))	.((((..(((....)))))).(((((....)))))	.((((.(((...)))..)))).(((((....)))).	.((((.((......))..)))).((((((....)))))	((((((((.......))(((....)))..)))))))
1UUT	((((((...))))))	(((((.....)))))	.(((((...))))).	..((((...))))..	.((((.....)))).	((((.......))))	((((.(...).))))	(.((((...)))).)	..(((.....)))..	....
1XUE	....	.((.....)).......	((.....))........	...(....)........	........(....)...	.....((...)).....	......(.(...).)..	.(.....).........	(((...)))........	(.......).......
1YTB	(((((((((((((.....)))))))))))))	.((((((((((((.....)))))))))))).	..((((((((((.....))))))))))..	(((((.((((((.....)))))).)))))	((((.(((((((.....))))))).))))	(((.((((((((.....)))))))).)))	((.((((((((.....))))))))).))	((((((.(((((.....))))).))))))	((((((((((.(....)))))))))))	...(((((((((.....)))))))))...
1ZHU	....	..((...)).	((......))	.....(...)	..(.....).	...(.....)	....(...).	(...).....	...(...)..	.(.....).
1ZM5	.((((((....))))))........	(((((((....))))))).......	.((((((....))))))(...)...	.((((((....)))))).(...)..	.((((((....)))))).(.....)	(((((((....)))))))(...)..	(((((((....)))))))(.....)	((((((....))))))..).....	..(((((....))))).........	((((((....))))))...)....
2A0I	....	..(.....)..	.(.....)..	(.....)...	.(...)....	...(...)..	(...).....	((.....)).	.....(...)	(.......).
2A6O	((((((((....)))).)))).	((((.(((....)))..)))).	(((.((((....)))).)))..	(((.((((....))))..))).	.(((((((....)))).)))..	.((((((....))))..))).	.((((((....)))..)))).	(((((.((....)).).)))).	((((............)))).	(((((((......))).)))).
2CDM	((((((....)))))).........	((((((....))))))((.....))	.(((((....)))))..........	((((((....))))))...(....)	((((((....))))))..(.....)	((((((....))))))(...)....	((((((....)))))).(...)...	((((((....)))))).(.....).	(((((......))))).........	((((((....))))))(.......)
2EXF	..(((.....))).	...(((....))).	(.(((.....))))	....	...((......)).	.(((......))).	..((......))..	...((......))..	((((......))))	.....(....)...
2JZW	..(((.....))).	...(((....))).	(.(((.....))))	....	...((......)).	.(((......))).	..((......))..	...((......))..	((((......))))	.....(....)...
2K71	....	((....))	..(....)	.(....).	(......)	/	/	/	/	/
2L5K	......((((...))))......	(((...((((...))))...)))	..((..((((...))))))....	(((..(((((...)))).).)))	............((....))..	....	.....(((((...)))).)....	.......(((...))).......	......(((.....)))......	(((.(.((((...)))))..)))
2LO5	(((......)))	....	((((...).)))	....((...)).	(....)......	.((......)).	((.(.....)))	.((....))...	..(....)....	(((.(...))))
2LO8	....	...((...))	((......))	.(....)...	((....))..	..(.....).	(((...).))	(...).....	..((...)).	...(...)..
2M8Y	((((((...))))))	(((((.....)))))	.(((((...))))).	((((.......))))	(((((....).))))	((((.(...).))))	.((((.....)))).	((((.(....)))))	..((((...))))..	(((.((...)).)))
2N8A	((((((((....)))))))).(((((((((....)))))))))	((((((((....))))))))).((((((((......))))))))	((((((((....))))))))..((((((((....)))))))).	.((((((((....))))))))..((((((((....)))))))))	((((((((......)))))))).(((((((((....)))))))))	((((((.(....).)))))).((((((((....))))))))))	((((((.((....)).)))))).(((((((((....))))))))))	((((((((....)))))))).(((((((........)))))))	((((((((....)))))))).(((((.(((....))).)))))	((((((((....)))))))).((((((.(....).)))))))
2VHG	((((((((....)))).))))...	((((.(((....)))..))))...	(((.((((....)))).))).....	(((.((((....))))..)))...	.(((((((....)))).))).....	.((((((....))))..))).....	.((((((....)))..))))....	(((((.((....)).).))))...	((((............))))...	(((((((......))).))))...
2VIC	....((((((((...)))))).))))	....((((.((((...))))..))))	....(((.(((((...))))).))).	....(((((((.....)))).))))	....(((.(((((...))))).)))	....(((((((...))))).))).	....((((.(((.....)))..))))	....(((.((((.....)))).))).	....(((((((...)))))..)))	....(((((.(((...))).).))))
2VJU	...((((((((....)))).))))).........	.(.((((((((....)))).)))).).......	...(((((.(((....)))..))))).........	.(.(((((.(((....)))..)))))).).......	...(((((((((....)))).))))(.....)..	...(((((((((....)))).)))).........	..(((((((((....)))).)))).).......	...((((.((((....))))..)))).........	...((((((((....)))).)))...(....)	((.((((((((....)))).)))).)....)..
3C46	......((((((...))))))	.......(((((...))))).	......(((((.....)))))	(....)((((((...))))))	.....(.(((((...))))))	........((((...)))..	(...).((((((...))))))	......(((.((...)).)))	......((.(((...))).))	....(..(((((...))))))
3DSD	.(((((((....))))))))....	..((((((....)))))).....	((((((((....)))))))..).	.(((((((....)))))).)...	.((((.((....)).))))....	.(((.(((....))).)))....	.((((((......))))))....	.((.((((....)))).))....	.(.(((((....))))).)....	...(((((....)))))......
3HXO	((((.....))))......((((((.....))).)))).	((((.....))))....((.((((((.....))).)))))	((((.....))))....(.((((((.....))).)))))	(((.......)))......((((((.....))).)))).	..........(((...)))((((((.....))).)))).	(.((...........)).)((((((.....))).)))).	.(((((((..(((...)))..))(((.....))).)))))	(((.......)))....((.((((((.....))).)))))	(((.......)))....(.(((((((.....))).)))))	.(((((....(((...)))...((((.....)))))))))
3Q0A	.......(((((...)))))	........((((...)))).	.......((((.....))))	.(....)(((((...)))))	.(...).(((((...)))))	.........(((...)))..	....	.......((.((...)).))	.......(.(((...))).)	.((....))(((...)))..
3Q23	......((((((...))))))	.......(((((...))))).	......(((((.....)))))	(....)((((((...))))))	.....(.(((((...))))))	........((((...)))..	(...).((((((...))))))	......(((.((...)).)))	......((.(((...))).))	....(..(((((...))))))
3Q24	.......((((((...))))))	........(((((...))))).	.(....)((((((...))))))	.......(((((.....)))))	.(...).((((((...))))))	......(.(((((...))))))	.........((((...)))..	.......(((.((...)).)))	.......((.(((...))).))	.....(..(((((...))))))
3THW	(((((((((((((((((((((...)))))))))....))))))))))))))	((((((((((((((((((((.....))))))))....))))))))))))))	.((((((((((((((((((((...)))))))))....))))))))))))).	(((((.(((((((((((((((...)))))))))....))))))))).)))))	((((.((((((((((((((((((...)))))))))....)))))))).))))	..((((((((((((((((((((...)))))))))....)))))))))))..	((((((((((((((((((((.......)))))))....))))))))))))))	(((((((((((((((((.((...)).))))))....)))))))))))))	((((((((((((((((.(((((...))))).)))....))))))))))))))	((((((((((((((((.((((((...)))))).))....))))))))))))))
3WPD	....	((.....)).	(((....)))	((......))	..(....)..	..(.....).	.((.....))	.((....)).	.(.....)..	.(.......)
3WPG	....	(((....))).	((.....))..	((......)).	.((.....)).	..(....)...	..(.....)..	.((....))..	.(.....)...	.(.......).

RNA model

PDB	RNAfold MFE	subopt #2	subopt #3	subopt #4	subopt #5	subopt #6	subopt #7	subopt #8	subopt #9	subopt #10
1AC7	((((((....))))))	.(((((....))))).	..((((....))))..	(((((......)))))	.((((......)))).	((((........))))	..(((......)))..	.(((........))).	((((.(....).))))	(.((((....)))).)
1B4Y	....(((((((........)))))))....	..........((((((....))))))....	..........(((((......)))))....	....((((((........))))))....	..........(((((....)))))....	....((((.((........)).))))....	....((((((.(........)))))))..	....(((.(((........))).)))....	....(((((.(........).)))))....	....((.((((........)))).))....
1BJH	((((...))))	....	.(((...))).	(((.....)))	..((...))..	((.......))	.((.....)).	...(...)...	((.(...).))	(.((...)).)
1ECU	((((((((...))))))))	(((((((.....)))))))	((((((.......))))))	.(((((((...))))))).	.((((((.....)))))).	(((((.........)))))	((((((.(...).))))))	.(((((.......))))).	(((((((....)).)))))	(((((((....).))))))
1EN1	((((......))))....	(.(((.....))))....	(((((.....))).))..	..(((......)))....	((.(((.....))).))..	(.(((.....)))...).	(((........)))....	((.((......)).))..	(((.(.....))))....	((.((.....))))....
1EZN	((((((......(((((.....)))))).)))))))	((((((......((((((...)))))).)))))))	.((((((......(((((.....))))).)))))).	.((((((......((((((...)))))).)))))).	(((((((..(...(((((.....))))))))))))	((((((........(((((....)))))))))))	((((((..(...((((((...)))))))))))))	((((((.......((((.....)))..)))))))	(((((........((((((....)))))))))))	(((((....((..(((((.....)))).)))))))
1JVE	((((((((((((...))))))))))))	.(((((((((((...)))))))))))).	(((((((((((.....)))))))))))	(((((((((((.......)))))))))))	(((((.((((((...))))))).))))	(((.(((((((...))))))).))))	((((((((.((...)).))))))))	((((((.(((((...))))).))))))	(((.((((((((...)))))))).)))	.((((((((((.....)))))))))).
1LA8	(((((...)))))	.((((...)))).	((((.....))))	.(((.....))).	..(((...)))..	(((.......)))	/	/	/	/
1NGO	((((((((((((.....))))))))))))	((((((((((.......))))))))))	.((((((((((.....)))))))))).	(((((((((((.(....)))))))))))	(((((.(((((.....))))).)))))	..(((((((((.....)))))))))..	.((((((((((.......)))))))))).	(((((((((.........)))))))))	((((((.((((.....)))).)))))	((((.((((((.....)))))).))))
1NGU	((((..(((((.....)))))..))))	.(((..(((((.....)))))..))).	((((...((((.....))))...))))	((((..((((.......))))..))))	(((...(((((.....)))))...)))	..(((.(((((.....)))))...)))	..((..(((((.....)))))..))..	.(((...((((.....))))...))).	((((....(((.....)))....))))	(((....((((.....))))....)))
1OSB	(((((((....))))))).......	.((((((....))))))........	..(((((....))))).........	((((((......)))))).......	((((.((....)).)))).......	/	/	/	/	/
1P0U	(((((...)))))	.((((...)))).	((((.....))))	....	(((.......)))	((.((...)).))	(((.(...).)))	.(((.....))).	/	/
1PQT	....	((...))	.(...).	(.....)	/	/	/	/	/	/
1SNJ	((((((.(((...............)).)))))))	.((((..(((....))))))..((((....)))).	((((((.(((..((.........))))).)))))))	.((((((.(((...............)).)))))).	.((((.((......))..)))).((((....)))).	.((((..(((....))))))).((((....)))))	.((((.(((...)))..)))).((((....)))).	.((((.((......))..)))).((((....)))))	((((((.(((..(......)....))).)))))))	.((((((.(((..((........)))).)))))).
1UUT	((((((...))))))	.(((((...))))).	(((((.....)))))	..((((...))))..	((((.......))))	.((((.....)))).	(.((((...)))).)	((((.(...).))))	/	/
1XUE	....	.((.....)).......	((.....))........	(((...)))........	..(.....)........	((........)).....	...(....)........	........(....)...	((.............))	(.......).......
1YTB	(((((((((((((.....)))))))))))))	.((((((((((((.....)))))))))))).	..((((((((((.....)))))))))))..	(((((((((((.......)))))))))))	(((((((((((.(....)))))))))))	((((((((.(((.....))).))))))))	((((((.((((.....)))).)))))))	((((((.(((((.....))))).)))))))	((((((((((.((.....)).))))))))))	((((.((((((.....)))))).)))))
1ZHU	....	((......))	(........)	..((...)).	.(......).	(...).....	..(.....).	/	/	/
1ZM5	(((((((....))))))).......	.((((((....))))))........	..(((((....))))).........	((((((......)))))).......	((((.((....)).)))).......	/	/	/	/	/
2A0I	....	..(....)..	(.(....).)	.(.....)..	((.....)).	(........)	/	/	/	/
2A6O	((((((((....)))).)))).	((((.(((....)))..)))).	(((.((((....)))).)))..	.((((((....)))).)))..	((((((((......))).)))).	((((..((....))...)))).	.((((((....)))..)))).	.(((((((....))))..))).	(((((.((....)).).)))).	(((.((((....))))..))).
2CDM	((((((....)))))).........	.(((((....)))))..........	.(((((....)))))(......)...	((((((....))))))((.....))	(((((......))))).........	(((.((....)).))).........	((((((....))))))..(.....)	/	/	/
2EXF	((((......))))	..(((.....))).	(.(((.....))))	...(((....))).	.(((......))).	..((......))..	...((.....))..	....	...((......)).	(((........)))
2JZW	((((......))))	..(((.....))).	(.(((.....))))	...(((....))).	.(((......))).	..((......))..	...((.....))..	....	...((......)).	(((........)))
2K71	((....))	....	.(....).	..(....)	(......)	/	/	/	/	/
2L5K	(((...((((...))))...)))	......((((...))))......	(((..(((((...)))).).)))	.((...((((...))))...)).	(((....(((...)))....)))	(((...(((.....)))...)))	..((..((((...)))))).....	.....(((((...)))).).....	((....((((...))))....))	..(...((((...))))...)..
2LO5	(((......)))	((((...).)))	....	.((......)).	....((...)).	((........))	((.(.....)))	.(((...).)).	(((.(...))))	((.((...))))
2LO8	....	((......))	...((...))	((....))..	(((...).))	.(....)...	.(......).	..(.....).	(...).....	..((...)).
2M8Y	((((((...))))))	(((((.....)))))	.(((((...))))).	.((((.....)))).	((((.......))))	.(((.......))).	((((.(....)))))	(((((....).))))	((((.(...).))))	(((.........)))
2N8A	((((((((....)))))))).((((((((....)))))))))	((((((((....))))))))..((((((((....)))))))).	((((((((....)))))))).(((((((......))))))))	((((((((....))))))))..(((((((......))))))).	.((((((((....))))))))..((((((((....)))))))))	((((((((....)))))))).((((((.........)))))))	((((((.((....)).)))))).((((((((....)))))))))	((((((((......)))))))).((((((((....)))))))))	(((((((.(....).))))))).(((((((((....)))))))))	.((((((((....))))))))...((((((((....)))))))).
2VHG	((((((((....)))).))))...	((((.(((....)))..))))...	(((.((((....)))).))).....	.(((((((....)))).)))....	((((((((......))).))))....	((((..((....))...))))...	.(((((((....)))..))))...	.(((((((....))))..)))....	(((((.((....)).).))))...	(((.((((....))))..)))...
2VIC	....((((((((...)))))).))))	....(((((((.....)))).))))	....(((.(((((...))))).))).	....((((.((((...))))..))))	.....((((((((...))))).))).	....(((.((((.....)))).))).	....((((.(((.....)))..))))	.....((((((.....)))).))).	....(((..((((...))))..))).	.....(((.((((...))))..))).
2VJU	...((((((((....)))).)))))..........	....(((((((....)))).))))........	.(.((((((((....)))).)))).).......	...(((((.(((....)))..)))))..........	((.((((((((....)))).))))).....))..	..((((((((....)))).)))).).......	...((((((((....)))).)))))(.....)..	(..((((((((....)))).)))).).......	(..((((((((....)))).))))...).....	(..((((((((....)))).))))......)..
3C46	......((((((...))))))	.......(((((...))))).	......(((((.....)))))	.......((((.....)))).	.....(.(((((...))))))	........((((...)))..	(((.........)))......	......(((.((...)).)))	......((.(((...))).))	.........(((...)))...
3DSD	.((((((....))))))))....	..((((((....)))))).....	.(((((((....)))))).)...	.((((((......))))))....	.((((.((....)).))))....	.(((.(((....))).)))....	((((((((....)))))))..).	...(((((....)))))......	.(.(((((....))))).)....	/
3HXO	(((.((((..(((...)))..)))).)))..........	((((.(((..(((...)))..))).))))..........	((((.....))))......(((((((.....))).)))).	((((((((..(((...)))..)))).)))).........	((((((((..(((...)))..)).)))))..........	(((.(((((.(((...))))).)))..))).........	(((.......)))......((((((...))).)))).	(((..(((..(((...)))..)))...))).........	((((.((((.((((.......).)))).)))...))))....	(((.((((..(((...)))..))))..)))..((...)).
3Q0A	.......(((((...)))))	........((((...)))).	.......((((.....))))	.........(((...)))..	....	.......((.((...)).))	.......(.(((...))).)	.....(((........))).	..........((...))...	((........))........
3Q23	......((((((...))))))	.......(((((...))))).	......(((((.....)))))	.......((((.....)))).	.....(.(((((...))))))	........((((...)))..	(((.........)))......	......(((.((...)).)))	......((.(((...))).))	........(((...)))...
3Q24	.......((((((...))))))	........(((((...))))).	.......(((((.....)))))	........((((.....)))).	......(.(((((...))))))	.........((((...)))..	.(((.........)))......	.......(((.((...)).)))	.......((.(((...))).))	.........(((...)))...
3THW	((((((((((((((((((((((...)))))))))....))))))))))))))	.((((((((((((((((((((((...)))))))))....)))))))))))))).	(((((((((((((((((((((.....))))))))....)))))))))))))	((((((((((((.((((((((...)))))))).....)))))))))))))	((((((((((((((((((((.......)))))))....))))))))))))))	.((((((((((((((((((((.....))))))))....))))))))))))).	(((((((((((.((((((((...))))))))).....))))))))))))	((((((((((.((((((((((...))))))))....)).)))))))))))	(((((.(((((((((((((((...)))))))))....))))))))).)))))	(((((((((((((((((((.(...).))))))....))))))))))))))
3WPD	((.....)).	(((....)))	....	((......))	.((....)).	.(.....)..	.((.....))	.(......).	(......)..	(.......).
3WPG	(((....))).	((.....))..	((......)).	....	.((.....)).	.((....))..	.(.....)...	.(......)..	((.....).).	(......)...

PDB	DNA model: RNAfold MFE	subopt #2	subopt #3	subopt #4	subopt #5	subopt #6	subopt #7	subopt #8	subopt #9	subopt #10
3WPH	....	(((....)))..	((.....))...	((......))..	.((.....))..	..(....)....	..(.....)...	......(....)	.((....))...	.(.....)....
3ZH2	(((((((.((......)).)).)))))	((((((((...)))......).))))))	((((((.(........).)).)))))	(((((................)))))	((((((............)).)))))	.((((((.((......)).)).)))).	(((((((.(((...).)).)).)))))	((((((.............).))))))	(((((.(.((......)).)..))))))	(((((.(((...))).(....))))))
4ER8	......(((((..((((....)))).))))))	......((((...((((....))))...))))	......(((((...(((.......))))))))	......(((((..(((......))).)))))	.......((((..((((....))))..)))).	......(((.(..((((....)))..).)))	.(...)(((((..((((....)))..)))))	......(((((...(((....)))...)))))	......(((....((((....))))....)))	......(((((..((((....)))))..))))
4F41	((((((((((((((....))))))))))))))	.(((((((((((((....)))))))))))))).	(((((((((((((......)))))))))))))	((((((((((((........))))))))))))	..(((((((((((((....))))))))))))..	((((((((((((.(....).))))))))))))	((((((((((.((....)).))))))))))	(((((.((((((((....))))))).)))))	((.((((((((((....))))))))))).))	((((((.(((((((....))))))).))))))
4F43	((((((((((((((....))))))))))))))	.(((((((((((((....)))))))))))))).	(((((((((((((......)))))))))))))	((((((((((((........))))))))))))	..(((((((((((((....))))))))))))..	((((((((((((.(....).))))))))))))	((((((((((.((....)).))))))))))	(((((.((((((((....))))))).)))))	((.((((((((((....))))))))))).))	((((((.(((((((....))))))).))))))
4FF1	.......(((((...)))))	........((((...)))).	((...))(((((...)))))	.......((((.....))))	.(....)(((((...)))))	(((....)))..........	.........(((...)))..	(...)..(((((...)))))	(....).(((((...)))))	....
4HT4	.(((((((...)))))))..........	.(((((((...)))))))....(....)	.((((((.....)))))).........	.(((((((...))))))).(....)...	....((((...))))((((....)))).	.(((((((...)))))))(....).....	..((((((...))))))...........	((((((((...)))))))....).....	.(((.(((...))).)))..........	.((((.((...)).)))).........
4KB0	((((((....))))))..	.(((((....)))))...	(((((......)))))..	((((((....))))).).	((((((....)))).)).	((((.(....).))))..	((((........))))..	..((((....))))....	((((((....)))))..)	(((.((....)).)))..
4KB1	((((((....))))))..	(((((......)))))..	.(((((....)))))...	((((.(....).))))..	((((........))))..	(((.((....)).)))..	.(((((....)))).)..	..((((....))))....	((((((....)))))..)	((((((....))))).).
5F55	....	.......((...))	(.....).......	......(....)..	...(....).....	......(.....).	(.....)((...))	..(....)......	...((.......))	....((...))...
5GWL	....	.(.....)	..(....)	(......)	/	/	/	/	/	/
5GWQ	....	.(.....)	..(....)	(......)	/	/	/	/	/	/
5HRU	((((.........(((((.....)))))))))	............(((((.....)))))....	.........((.(((((.....))))).)).	..........(.(((((.....))))).)..	........(((.(((((.....)))))).)))	((((.........((((.......))))))))	............((((.......))))....	.(((.........(((((.....))))))).	..........((.((((.......)))).)).	((((.........(((........)))))))
5HTO	(((((.........(((((.....))))))))))	(((((.........(((.......)))))))))	.((((.........(((((.....))))))).	.............(((((.....)))))....	(((((.........(((.........))))))))	..........((.(((((.....))))).))..	............(.(((((.....))))).)...	..........(((.(((((.....)))))).))).	(((((..(.....)(((((.....))))))))))	(((((...(.....)(((((.....))))))))))
5N2Q	((((((((....)))))))........	.((((((....))))))........	((((((......))))))........	((((((((....)))))))(.....).	(((((.(....).))))........	((((.((....)).)))).......	(((((((....)))))))(...)...	((((((((....))))))).(...)..	(((((((....))))))).(.....)	(((((((....)))))))..(...).
5OND	....	.(....)..	((....)).	...(....)	..(.....)	...(...).	..(....).	..((...))	((....).)	(......).
6FK4	..(((((...))))).	.(((.....))).....	((((.....))))...	...((((...))))..	..((((.....)))).	....(((...)))...	....	..((.((...)).)).	..(.(((...))).).	.((.......))....
6FK5	(((((...))))).	.((((...))))..	((((.....)))).	..(((...)))...	....	((.((...)).)).	(.(((...))).).	...((...))....	((.....)).....	.(((.....)))..
6FKE	.((((...))))	..(((...))).	....	.(((.....)))	...((...))..	.(.((...)).)	..((.....)).	((......))..	(...).......	.(....).....
6IY5	....	((...))...	.....(...)	(...).....	(.....)...	.(...)....	(......)..	(........)	/	/
6J37	....	..(....)	.(.....)	(......)	/	/	/	/	/	/
6M0B	....	(....)..	..(....)	.(...)..	.(.....)	(......)	/	/	/	/
6M0C	....	..(....)	.(...)..	.(.....)	(......)	((...).)	/	/	/	/
6SEI	.(((.((((((((...)))))))..)).)))	..((.((((((((...)))))))..)).)).	.....((((((((...)))))))).)).....	.(((.((((((((.....)))))).)).)))	.......((((((...)))))))........	.(((.((.((((((...))))))...)).)))	...(.((((((((...)))))))..)).)..	.(((.(((.(((((...))))).).)).)))	.((..((((((((...)))))))..))..))	.(((..(((((((...)))))))..)..)))
6U82	((((((((((((((((...))))))))))...))))))	((((((((((((((.....))))))))))...))))))	.(((((((((((((((...))))))))))...))))).	((((((((.(((((((...))))))).))...))))))	(((.(((((((((((...))))))))))...)).)))	......((((((((((...)))))))))).........	((((((((.((((((...)))))).)))...))))))	(((((((.((((((((...))))))))).)...))))))	((((.((((((((((...))))))))))...).))))	((.(((((((((((...))))))))))...))).))
17RA	/	/	/	/	/	/	/	/	/	/
1A1T	/	/	/	/	/	/	/	/	/	/
1A4T	/	/	/	/	/	/	/	/	/	/
1A51	/	/	/	/	/	/	/	/	/	/
1A60	/	/	/	/	/	/	/	/	/	/
1A9N	/	/	/	/	/	/	/	/	/	/
1AFX	/	/	/	/	/	/	/	/	/	/
1ANR	/	/	/	/	/	/	/	/	/	/
1ATO	/	/	/	/	/	/	/	/	/	/
1ATV	/	/	/	/	/	/	/	/	/	/
1ATW	/	/	/	/	/	/	/	/	/	/
1AUD	/	/	/	/	/	/	/	/	/	/
1B36	/	/	/	/	/	/	/	/	/	/
1BGZ	/	/	/	/	/	/	/	/	/	/
1BVJ	/	/	/	/	/	/	/	/	/	/
1BZ2	/	/	/	/	/	/	/	/	/	/
1BZ3	/	/	/	/	/	/	/	/	/	/
1CQ5	/	/	/	/	/	/	/	/	/	/

PDB	RNA model: RNAfold MFE	subopt #2	subopt #3	subopt #4	subopt #5	subopt #6	subopt #7	subopt #8	subopt #9	subopt #10
3WPH	(((....)))..	((.....))...	((......))..	....	.((.....))..	.((....))...	.(.....)....	.(......)...	((.....).)..	(......)....
3ZH2	(((((((.((......)).)).)))))	.((((((.((......)).)).)))).	(((((((.(........).)).)))))	((((((..((......))..).)))))	..(((((.((......)).)).)))..	(((((((.(((...).)).)).)))))	((((((............)).)))))	((((((((...)))......).))))))	(((((((..(......)..)).)))))	.((((((.(........).)).)))).
4ER8	......((((..((((....))))..)))))	.......((((..((((....)))).)))).	......(((((..(((......)))..)))))	......((((...((((....))))...))))	.......((((..(((......)))..)))).	......(((...((((....))))...))).	((...))((((..((((....)))..)))).	......((((...(((.......)))...))))	......(((....((((....))))...)))	(.....)((((..((((....))))..)))).
4F41	(((((((((((((....))))))))))))))	(((((((((((((......)))))))))))))	.(((((((((((((....)))))))))))))).	(((((((((((........)))))))))))))	.((((((((((((......))))))))))))).	..(((((((((((....)))))))))))..	((((((((((.(((....))).))))))))))	((((.((((((((....))))))))).))))	(((((((((((((...).)))))))))))))	.(((((((((((........)))))))))))).
4F43	(((((((((((((....))))))))))))))	(((((((((((((......)))))))))))))	.(((((((((((((....)))))))))))))).	(((((((((((........)))))))))))))	.((((((((((((......))))))))))))).	..(((((((((((....)))))))))))..	((((((((((.(((....))).))))))))))	((((.((((((((....))))))))).))))	(((((((((((((...).)))))))))))))	.(((((((((((........)))))))))))).
4FF1	.......(((((...)))))	........((((...)))).	.......((((.....))))	.........(((...)))..	....	(((....)))..........	.......((.((...)).))	.((....))(((...))..	.......(.(((...))).)	.....(((........))).
4HT4	.(((((((...)))))))..........	.((((((.....))))))..........	..((((((...))))))...........	.(((((((...)))))))....(....)	.((((.((...)).))))..........	...(((((...)))))(((....)))..	((((((((...)))))))....).....	.(((.(((...))).)))..........	...(((((...)))))((......))..	.(((((((...))))))).(......).
4KB0	((((((....))))))..	(((((......)))))..	.(((((....)))))...	((((((....))))).).	((((((....)))).)).	.((((......))))...	((((........))))..	(((((......)))).).	(((((......))).)).	/
4KB1	((((((....))))))..	(((((......)))))..	.(((((....)))))...	((((........))))..	.((((......))))...	/	/	/	/	/
5F55	....	......(....)..	.......((...))	......(.....).	(.....).......	...((.......))	...((.(....)))	...(....).....	(.......).....	..(.((...)).).
5GWL	....	.(.....)	(......)	/	/	/	/	/	/	/
5GWQ	....	(......)	/	/	/	/	/	/	/	/
5HRU	.........(((.(((((.....))))).)))	..........((.(((((.....))))).)).	((.....))(((.(((((.....)))))).)))	...........(.(((((.....))))).)..	((((.........(((((.....)))))))))	((.....)).((.(((((.....))))).)).	............(((((.....))))).....	.(((.........(((((.....))))))).	((.....))..(.(((((.....))))).)..	.(.....).(((.(((((.....))))).)))
5HTO	.........(((.(((((.....))))).))).	.((.....))(((.(((((.....))))).))).	.((((.........(((((.....))))))))).	(((((.........(((((.....)))))))))	..........((.(((((.....))))).))..	(((....)))(((.(((((.....))))).))).	...........(.(((((.....))))).)...	.((.....)).((.(((((.....))))).))..	..(.....).(((.(((((.....))))).))).	............(((((.....))))).....
5N2Q	((((((((....)))))))........	.((((((....))))))........	((((((......))))))........	(((((........)))))........	.(((((......))))).........	(((.(((....))).))).......	..(((((....)))))..........	((((.((....)).)))))........	(((((((....)))))))((...)).	((.((((....)))).))........
5OND	....	((....)).	.(....)..	((....).)	..(.....)	...(....)	..((...))	..(....).	/	/
6FK4	..(((((...))))).	((((.....))))....	..((((.....)))).	...((((...))))..	.(((.....))).....	(((.......)))...	....(((...)))...	..((.((...)).)).	..(.(((...))).).	....
6FK5	(((((...))))).	((((.....)))).	.((((...))))..	..(((...)))...	((.((...)).)).	(.(((...))).).	....	/	/	/
6FKE	.((((...))))	..(((...))).	....	...((...))..	((.......))..	.(((.....)))	..((.....)).	(........)..	.(.((...)).)	...(.....)..
6IY5	....	(......)..	/	/	/	/	/	/	/	/
6J37	....	(......)	/	/	/	/	/	/	/	/
6M0B	....	(......)	(....)..	/	/	/	/	/	/	/
6M0C	....	(......)	/	/	/	/	/	/	/	/
6SEI	.(((.((((((((...))))))))..)).)))	.(((.((.((((((...))))))...)).)))	..((.((((((((...)))))))..)).)).	..((.((.((((((...))))))...)).)).	.....((((((((...)))))))..)).....	...(.((((((((...)))))))..)).)..	.....((.((((((...))))))...))....	...(.((.((((((...))))))...)).)..	.((..((((((((...))))))))..))..))	.(((.(((((((.....))))))).)).)))
6U82	((((((((((((((((...))))))))))...))))))	(((((.((((((((((...))))))))))....)))))	.((((((((((((((...))))))))))...))))).	((((((((((((((.....))))))))))...))))))	((((..((((((((((...))))))))))....))))	((((((.((((((((((...))))))))....))))))	(((((((((.((((((...)))))).)))...))))))	((((.((((((((((...))))))))))...).))))	((((((((.((((((...))))))).))...))))))	(((.((((((((((...))))))))))...)).)))
17RA	((((((.((....))))))))	(((((.(((....))))))))	((((((.(.....).))))))	.(((((.((....)))))))).	.((((.(((....)))))))).	(((((..((....)).)))))	((((..(((....))).))))	((((((.(......)))))))	(((((.((......)))))))	((((((.........))))))
1A1T	((((((((....))))))))	.(((((((....)))))))).	(((((((......)))))))	(((((.((....)).)))))	((((.(((....))).))))	/	/	/	/	/
1A4T	(((((.....)))))	.((((.....)))).	((((.......))))	(((((....).))))	..(((.....)))..	/	/	/	/	/
1A51	((((.((....((((((((...))))))))....)).))))	((((.......((((((((...))))))))......))))	((((.((..(.((((((((...))))))).)..)).))))	((((..(....((((((((...))))))))....)..))))	((((......(.((((((((...))))))).)......))))	((((..(....((((((((...))))))))...)...))))	((((.((....(((((((.....))))))....)).))))	((((.......(((((((.....)))))).......))))	.(((.((....((((((((...))))))))....)).))).	.(((.......((((((((...))))))))......))).
1A60	(((((.......)))))......((((((......))))))...	(((((.......)))))......(((((......)))))....	(((((.......))))).(((((....)))))...........	(((((.......)))))........((((......)))).....	(((((.....)))))........((((((......))))))...	(((((.......))))).......(((((........)))))...	((((.........))))......(((((......))))))...	(((((.....))))).........(((((......)))))....	(((((.......))))).......((((........))))....	(((((.....))).)).......((((((......))))))...
1A9N	(((((............))))).	((((((((....)))))...))).	((((((((....)))...))))).	(((((((....))))...)))).	(((((...((...))...))))).	(((((....(......)..))))).	...(((((....))))).......	(((((..(......)...))))).	(((((...(......)...))))).	(((((((......))))...))).
1AFX	((((....))))	.(((....))).	(((......)))	....	.((....))...	((........))	(((....)).).	((.(....).))	(.......)...	.((......)).
1ANR	((((((...((((......))))))))))	((((((...(((((...).))))))))))	.(((((...((((......)))))))))).	((((((....((((......)))).)))))	((((((...((((.(...)))))))))))	((((((...(((.((...))))))))))	((((((...(((........)))))))))	.(((((...(((((...).)))))))))).	((((((....(((......))).))))))	(((((....(((((...).)))).)))))
1ATO	((((((.......))))))	((((((.(...).))))))	.(((((.......))))).	(((((((....).))))))	.(((((.(...).))))).	(((((.........)))))	(((.((.......)).)))	.((((((....).))))).	/	/
1ATV	((((((....)))))).	(((((......))))).	.(((((....)))))..	/	/	/	/	/	/	/
1ATW	(((((....))))).	((((......)))).	.((((....))))..	((.((....)).)).	/	/	/	/	/	/
1AUD	.((((.((((....)))).)))).......	(((((.((((....)))).))))).....	(((((.((((....)))).......)))))	.(((..((((....))))..))).......	(((((((((....))))).).....)))))	((((..((((....))))..)))).....	(((((.((((....)))).))))..)....	(((((.((((....)))).))).))....	(((((((((....))))..)....)))))	((((((((((....)))).)).....))))
1B36	(((((.......((((....)))).........)))))	(((((..((...((((....))))....))..)))))	(((((..((((.((((....))))))...))..)))))	(((((.....((.((((....))))))........)))))	(((((.((....((((....))))......))..)))))	(((((.((.((.((((....))))))...))..)))))	(((((.......((((....))))(((...))))))))	(((((.......))))..((.(((((...))))).))	(((((..(....((((....))))......)..)))))	(((((.......((((......))))........)))))
1BGZ	(((((((((...)))))..)))))	(((((((((...)))))..))))	(((((..(((....))).)))))	((((.((((...))))...))))	((((((((.....)))..)))))	(((((.(((...)))...)))))	((((((((.....))))..))))	(((((...((....))..)))))	(((.(((((...)))))...)))	((((...(((....)))..))))
1BVJ	(((((.(((......))))))))	.((((.(((......)))))))).	(((((.((........)))))))	((((..(((......))).))))	(((((..((......)).)))))	(((((............)))))	(((((.(..........))))))	(((((.(((.....).)))))))	(((((.(.(.......).))))))	(((((...(.....)...)))))
1BZ2	(((((.......)))))	.((((.......)))).	((((.........))))	.(((.........))).	(((((.(...).)))))	(((((.(....))))))	.((((.(...).)))).	.((((.(....))))).	....	..(((.......)))..
1BZ3	(((((.......)))))	.((((.......)))).	((((.........))))	.(((.........))).	(((((.(...).)))))	(((((.(....))))))	.((((.(...).)))).	.((((.(....))))).	....	..(((.......)))..
1CQ5	(((((((....(((....(((....)))....))).)))))))	((((((.....(((....(((....)))....)))..))))))	(((((...(..(((....(((....)))....)))..))))))	(((((......(((....(((....)))....)))...)))))	((((....((.(((....(((....)))....)))..))))))	((((((....(((..(.(((....)))..).))).)))))))	((((((....((.....(((....))).....)).)))))))	.((((((....(((....(((....)))....))).)))))).	((((((....(((..(.(((....)).).).))).)))))))	((((((....((.(...(((....)))....))).)))))))

	DNA model											RNA model									
PDB	RNAfold MFE	subopt #2	subopt #3	subopt #4	subopt #5	subopt #6	subopt #7	subopt #8	subopt #9	subopt #10		RNAfold MFE	subopt #2	subopt #3	subopt #4	subopt #5	subopt #6	subopt #7	subopt #8	subopt #9	subopt #10
1CQL	/	/	/	/	/	/	/	/	/	/		((((((....(((....(((....)))....))).)))))))	(((((.....(((....(((....)))....)))..))))))	(((((...(..(((....(((....)))....)))..))))))	(((((......(((....(((....)))....)))...)))))	((((....((.(((....(((....)))....)))..))))))	((((((....(((..(.(((....)))..).))).)))))))	((((((....((.....(((....))).....)).)))))))	.((((((....(((....(((....)))....))).)))))).	((((((....(((..(.(((....)).).).))).)))))))	((((((....((.(...(((....)))....))).)))))))
1D0U	/	/	/	/	/	/	/	/	/	/		((((((.((....))))))))	.(((((.((....))))))).	..((((.((....))))))..	((((((.(......)))))))	((((((.........))))))	(((((..((....)).)))))	((((((.(......).))))))	((((((..(....).))))))	(.((((.((....)))))).)	.(((((.((....)))))).)
1DRZ	/	/	/	/	/	/	/	/	/	/		(.(((((.((((.(((((......)))))).))))))))).)....(((((((((....))).).)))))...	(.(((((.((((.(((((......))))))).))))))))).)....(((((((((....)).)).)))))...	(.(((((.((((.(((((......))))).))))))))).)....((((((((....)).)))).)))...	(.(((((.((((.(((((......)))))).))))))))).)....(((((((((....)).)).))))...	(.(((((.((((.(((((......))))).))))))))).)....(((((((((....)).)))).)))...	(.(((((.((((.(((((......)))))).))))))))).)....((((((((((....)).))).))))...	(.(((((..(((.(((((......))))).))).))))).)....((((((((....))).).)))))...	(.(((((..(((.(((((......))))).))).))))).)....(((((((((....)).)).)))))....	(.(((((.((((.(((((......))))).))))))))).)....((((((.(((....)))...)))))...	(.(((((.((((.(((((......))))).))))))))).)..(.((((((((((....))).).)))))..
1E4P	/	/	/	/	/	/	/	/	/	/		((((...(((....)))...))))	.(((...(((....)))...))).	..((...(((....)))...))..	..(((..(((....)))....)))	((((...(((....)).)..))))	((((....((....))....))))	((.((..(((....))))).))..	((((...((......))...))))	(.((...(((....)))...)).)	(((((..(((....))))..))))
1EBR	/	/	/	/	/	/	/	/	/	/		((((..((((((....)))).))...))))	(((((.((((((....)))).)).).))))	((((.(((((((....)))).)).).))))	((((.(((((((....)))).).)).))))	((((.((.((.((((....))))...)).))))	((((..((((((....)))).).)..))))	.(((..((((((....)))).))...)).	.((((.((((((....)))).)).).))).	(((.(.((((((....)))).)).)..)))	((((..(.((((....))))...)..))))
1EBS	/	/	/	/	/	/	/	/	/	/		((((..((((((....))))))...)))))	(((((.((((((....)))))).).))))	((((.(((((((....)))))).).))))	((((.((((((....))))).)).))))	((((..((((((....))))).)..))))	.(((..((((((....)))))...))).	.((((.((((((....)))))).).))).	(((.(.((((((....)))))).)..)))	((((...(((((....)))))....))))	(((...((((((....))))))...)))
1EIY	/	/	/	/	/	/	/	/	/	/		((((((((.((((........)))).(((((.......)))))......((((.......)))))))))))).....	((((((..((((........)))).(((((.......)))))).....(((((.......))))))))))))....	((((((((.((((........)))).(((((.(....)))))).....((((.......)))))))))))))....	((((((((.((((........)))).((((.......))))......((((.......))))))))))))....	((((((..((((........)))).(((((.(....))))))....(((((.......)))))))))))))....	(((((((..((((........)))))..((((.......))))......(((((.......)))))))))))))....	((((((((.((((........)))).((((.........)))).......((((.......))))))))))))....	(((((((..((((........)))).((((.........)))).....(((((.......))))))))))))....	(((((((..((((........)))).(((((.......)))))......((((.......)))).)))))))....	((((((((.((((........))))..((((.(....))))).......((((.......))))))))))))....
1EKZ	/	/	/	/	/	/	/	/	/	/		((((((((((((....)))))))))))))	.((((((((((((....))))))))))))).	((((((((((((......))))))))))))	((((((.(((((....))))).)))))))	((((.((((((((....)))))))).))))	/	/	/	/	/
1ESH	/	/	/	/	/	/	/	/	/	/		(((((...)))))	.((((...)))).	((((.....))))	((.((...)).))	.(((.....))).	/	/	/	/	/
1ESY	/	/	/	/	/	/	/	/	/	/		((((((....))).))))	.((((((....))).))).	((((((.......)).))))	(((.(((....)))..)))	((((.((....))..))))	((((...........))))	.(((((......)).))).	/	/	/
1ETF	/	/	/	/	/	/	/	/	/	/		((((((..((((((....)))).))...))))))	(((((((.((((((....)))).)).).))))))	(((((((.(((((((....)))).)).).))))))	((((((.((((((((....)))).).)).))))))	(((((((.((.((((....))))...)).))))))	((((((..((((((....)))).).)..))))))	.(((((..((((((....)))).))...))))).	.((((((.((((((....)))))).).).))))).	(((((.(.((((((....)))).)).)..)))))	(((((((..(.((((....))))...)..))))))
1EUQ	/	/	/	/	/	/	/	/	/	/		((((((((((...)))))......(((((.......)))))..((((((.......))))))))))......	((((((((((...))))))......(((((.......)))))..((((((.......)))))).))))......	(((((...(((.........))).(((((.......)))))..((((((.......))))))))))))).....	((((((..(((.........))).(((((.......))))))...((((((.......))))))))))......	(((((((.(((....)))...((.(((((.......)))))..))((((.......))))))))))))).....	((((.(((((...)))))......(((((.......)))))..((((((.......)))))).)))).....	((((((((((...)))......((.((((.......)))))..))(((((.......))))))))))......	((((((.((((...)))).......(((((.......)))))..((((((.......))))))))))).....	(((((...(((....)))......(((((.......)))))..((((((.......)))))))))))......	((((((((.(((.........))).(((((.......)))))....((((.......)))))))))))......
1EXY	/	/	/	/	/	/	/	/	/	/		((((((((((((....)))))..))).)))))).	((((((((((((....)))))..))))..))))).	((((((((((((....)))))..)))..))).))	(((((((.((((....))))...))..))))).	((((.(((((((....)))))..)))...))))).	((((((((((((....)))))..))))..)).))	.((((((((((((....)))))..)))..))))..	(((((((.((((....))))...)))..))))).	((((((((.(((....)))...)))..))))).	.((((((((((((....)))))..))))..)))..
1F7F	/	/	/	/	/	/	/	/	/	/		(((((.((((((....)))))))))))	.((((.((((((....)))))))))).	(((((((.((((....))))))))))))	((((((.((((((....))))))))))))	(((((.(((((......))))))))))	(((.(.((((((....)))))))).)))	((.((.((((((....)))))))).))	((((..((((((....))))))).))))	.((((((.((((....)))))))))).	.(((((.(((((....)))))))))).
1F84	/	/	/	/	/	/	/	/	/	/		((((.....(((......)))....))))	((((...(.(((......)))...))))	.(((.....(((......)))....))).	((((...(.(((......))).)..)))	((((.((.....)).))))((.....)).	((((.....((........))....))))	((((.(........).))))((.....)).	((((...........))))((.....)).	((((.....((((...).)))....))))	((((......(((......)))...)))
1F85	/	/	/	/	/	/	/	/	/	/		(((((....)))))	.((((....)))).	((((......))))	..(((....)))..	.(((......))).	..(((....)))...	(.(((...))).).	.((((....))).)	(.(((....))).)	..((.....))...
1F9L	/	/	/	/	/	/	/	/	/	/		((((..(((....)))..))))	.(((..(((....)))..))).	((((...((....))...))))	((((..((......))..))))	(((...(((....)))...)))	((((..(.(....).)..))))	/	/	/	/
1FFY	/	/	/	/	/	/	/	/	/	/		(((((((.......(((((((.((((.(((((((.((....)).)))..))))))))).)).)))))))))))))..	(((((((.......(((((...((((.(((((((.((....)).)))..)))))))....))))))))))))..	(((((((.......((((((..((((.(((((((.((....)).)))..)))))))..).))))))))))))..	(((((((.......(((((.(.((((.(((((((.((....)).)))..))))))).)..)))))))))))))..	(((((((.......(((((((.((((.((((((((.(......).)))..))))))))).)).)))))))))))))..	(((((((.......(((((...(((.((((((((.(......).)))))..))))))))....))))))))))))..	(((((((.......(((((((.((((.((((((((..........)))..)))))))).)).)))))))))))))..	(((((((.......(((((((.((((.((((((((.((....)).)))..))))))))))..)))))))))))))..	((((((((((((.....)))))((.(.(((((.......))))).).))(((((.......))))))))))))..	(((((((.......(((((...((((.((((((((..........)))..))))))))....))))))))))))..
1FHK	/	/	/	/	/	/	/	/	/	/		((((......))))	(((........)))	.(((......))).	...(((.....)))	.((........)).	....	...((((...))))	((((.(...)))))	(((.((...)))))	(((.(.....))))
1FJE	/	/	/	/	/	/	/	/	/	/		((((..............))))	((((...((......)).))))	((((....(......)...))))	.(((..............))).	((((.....(......)..))))	((((.(...........).))))	((((....(.....)....))))	((((..(..........).))))	((((.....(.......)..))))	(((((.......)......))))
1FQZ	/	/	/	/	/	/	/	/	/	/		(((.....(((.......)))....)))	.((((......))))(((.......)))	(((...(.(((......))))...)))	.((((......))))...((.....))	.((.....(((.......)))....)).	(((...(.(((......))).)..)))	.((((......))))...........	(((.....((.........))....)))	(((.....((((...).)))....)))	(((.((......)).))).((.....))
1FYO	/	/	/	/	/	/	/	/	/	/		((((.(.((((....))))..).))))	((((...((((....))))....))))	(((((..((((....))))..).))))	.(((.(.((((....))))..).))).	.(((...((((....))))....))).	((((.(..(((....)))...).)))))	((((((((.((...))))))...))))	.((((..((((....))))..).))).	(((....((((....))))......)))	(((.((((.((...)))))).)))...
1G70	/	/	/	/	/	/	/	/	/	/		((((((..(((((....))).))...))))))	(((((((.(((((....))).))).).))))))	((((((.(((((((....))).)).).))))))	((((((.(((((....))).).)).))))))	((((((.((.(((....)))...)).))))))	((((((..(((((....))).).)..))))))	.(((((..(((((....))).))...))))).	.((((((.(((((....))).)).).))))).	(((((.(.(((((....))).)).)..)))))	((((((..(.(((....)))...)..))))))
1GRZ	/	/	/	/	/	/	/	/	/	/		(((((.((...((((((...(((((((....((((.((((((((.(((((((((....)))))))))).(((((....)))))).))))......))))))))...)))))))..)).))))((...((((...((((((((....)))))))).))))))((((....))))...)).)))))).(((((((.....))))))))....((..((.((.(((...((((....)))))))).)).)).)).	[illegible](((....((((.((((((((((.((((((((((....))))))))).(((((....))))).))))......))))))))...))))))..)).))))((...((((...((((((((....))))))))..))))))((((....))))...)).))))).((((((((.....))))))).........((.((.((((...((((....))))))).)).))..	[illegible](((((....(((.((((((((((.((((((((((....)))))))))).(((((....))))).)))).....))))))))...))))))))..)).))))((...((((...((((((((....))))))))..))))))((((....))))...)).))))).((((((((.....)))))))....((..((.((.(((.[illegible]	(((((.((...((((((...(((((((....(((.((((((((.((((((((((....)))))))))).(((((....))))).))))......))))))))...))))))..)).))))((...((((...((((((((....))))))))...)))))((((....))))...)).))))).((((((((.....))))))))...(.((.((.(((...((((....)))))).)).)))....	[illegible]((((....((((.(((((((((.((((((((((....)))))))))).(((((....))))).))))......)))))))...))))))..)).))))((...((((...(((((((((....))))))))..)))))((((....))))...)).))))).(((((((((.....)))))))....((..((.((.((...(((((....))[illegible]	[illegible](((....((((.((((((((.((((((((((....))))))))).((((((....))))).))))......))))))))...))))))..)).))))((...((((...(((((((((....)))))))))..))))))((((....))))...)).))))).(((((((((.....))))))).........((.((.((...((((((....))))))).))[illegible]	(((((.((...((((((...(((((((....(((.(((((((((.((((((((((....))))))))).(((((....))))).))))......))))))))...))))))..)).))))((...((((...((((((((....))))))))..))))))(((((....))))...)).))))).((((((((.....))))))))....((..((.((.((...((((((....)))))))).)).)).)).	[illegible]((((....(((.(((((((((.((((((((((....))))))))).((((((....))))).)))).....))))))))...))))))..)).))))((...((((...(((((((((....))))))))..))))))((((....))))...)).))))).(((((((((.....)))))))........((.((.((...((((((....))[illegible]	[illegible](((....((((.(((((((((.((((((((((....))))))))).(((((....))))).))))......)))))))...))))))..)).))))((...((((.(.((((((((....))))))).))))))((((....))))...)).))))).((((((((.....)))))))....((..((.((.((((...((((....))))))).)).)).)[illegible]	[illegible](((....((((.(((((((((.((((((((((....))))))))).(((((....))))).))))......))))))))...))))))..)).))))((...((((.(.((((((((....))))))).))))))((((....))))...)).))))).((((((((.....))))))).........((.((.((((...((((....))))))).)).))..
1GTR	/	/	/	/	/	/	/	/	/	/		((((((..(((.........))).(((((.......)))))......(((((.......))))))))))).....	(((((((.(((.........))).(((((.......)))))......((((.......))))))))))))).....	((((((((((...)))))......(((((.......)))))......(((((.......))))).)))))).....	((((((((((...))))))......(((((.......)))))......(((((.......))))).)))).....	(((((((.(((.........)))...((((((.((((.(((....)).)))).)))))).))))))).....	(((((((.(((.........)))...((((((.((.(((((....)))))).)).))))))))..)))))))......	(((((...(((.........))).(((((.......)))))......(((((.......))))).)))).....	((((((((((...))))......(((((.......)))))......(((((.......)))))))))))....	((((((..(((....)))......(((((.......)))))......(((((.......))))))))))))).....	((((((((.(((....)))..((..(((((.......)))))....))(((((.......))))))))))))).....
1HLX	/	/	/	/	/	/	/	/	/	/		((((((((....))))))))	.((((((((....)))))))).	(((((((......)))))))	(((((.((....)).)))))	((((((.(....).))))))	((((.(((....))).))))	((((((.........))))))	(((.((((....)))).)))	/	/
1HS1	/	/	/	/	/	/	/	/	/	/		(((......))).	((........)).	....	.((......))..	..((......)).	/	/	/	/	/
1HS2	/	/	/	/	/	/	/	/	/	/		(((......))).	((........)).	((((...).))).	....	.((......))..	((.....))....	..((......)).	/	/	/
1HS3	/	/	/	/	/	/	/	/	/	/		(((......))).	((........)).	....	.((......))..	..((......)).	/	/	/	/	/
1HS4	/	/	/	/	/	/	/	/	/	/		(((......))).	((........)).	((((...).))).	....	.((......))..	..((......)).	/	/	/	/
1HS8	/	/	/	/	/	/	/	/	/	/		(((......))).	((........)).	....	.((......))..	..((......)).	/	/	/	/	/
1HVU	/	/	/	/	/	/	/	/	/	/		...(((((........))))).........	.........((((((........))))))	..........(((((........))))).	...((((........))))...........	.....(((........)))..........	..............((((......)))).	.((........))..((((......)))).	...((((..........))))........	............((((........))))..	((.(((((........)))))....))....

	DNA model											RNA model									
PDB	RNAfold MFE	subopt #2	subopt #3	subopt #4	subopt #5	subopt #6	subopt #7	subopt #8	subopt #9	subopt #10		RNAfold MFE	subopt #2	subopt #3	subopt #4	subopt #5	subopt #6	subopt #7	subopt #8	subopt #9	subopt #10
1HWQ	/	/	/	/	/	/	/	/	/	/		(((((...(((((....)))))...))))))	(((((...((((......))))...)))))	.((((...(((((....)))))...)))).	(((((...(((((....))))).)..)))))	(((((...(((((....))).))..)))))	(((((...(((((....)).))).)))))	(((((....((((....))))....)))))	((.((...(((((....))))).))..)).))	((((((..(((((....))))))..)))))	.((((...((((......))))...)))).
1I3X	/	/	/	/	/	/	/	/	/	/		(((((((.....)))))))	.((((((.....)))))).	((((((.......))))))	(((.(((.....))).)))	/	/	/	/	/	/
1I46	/	/	/	/	/	/	/	/	/	/		(((((...)))))	.((((...)))).	((((.....))))	((.((...)).))	.(((.....))).	/	/	/	/	/
1I4B	/	/	/	/	/	/	/	/	/	/		(((((...)))))	.((((...)))).	((((.....))))	((.((...)).))	.(((.....))).	/	/	/	/	/
1IDV	/	/	/	/	/	/	/	/	/	/		(((....)))	((.....)).	....	.((....)).	((......))	.((.....))	(......)..	.(.....)..	.(......).	/
1IK1	/	/	/	/	/	/	/	/	/	/		(((((...))))).	((((.....)))).	(((.......))).	.((((...))))..	(((.(...).))).	((.((...)).)).	/	/	/	/
1IKD	/	/	/	/	/	/	/	/	/	/		((((((((....))))))))....	((((((......))))))....	.((((((....)))))).....	((.((((....)))).))....	.((((((....)))).))....	(((((.(....).)))))....	/	/	/	/
1JBT	/	/	/	/	/	/	/	/	/	/		((((((...((.(.....).)).))))))	((((((...((.(.....)..)).))))))	((((((......(....).....))))))	.(((((...((.(.....).)).))))).	((((((.................))))))	((((((...((.........)).))))))	.(((((...((.(....)..)).)))))).	((((((((..........))...))))))	.(((((......(....).....))))).	.(((((.................))))).
1JO7	/	/	/	/	/	/	/	/	/	/		(((((((.((.(((....))))).)))))))	(((((((.(.((((....)))))).)))))))	(((((((...((((....))))..)))))))	.((((((.((.(((....))))).)))))).	.((((((.(.((((....))))).)))))).	.((((((...((((....))))...)))))).	((((((....((((....))))...))))))	((((((..((.(((....))))))..))))))	((((((..(.((((....)))))..))))))	.(((((....((((....))))...))))).
1JOX	/	/	/	/	/	/	/	/	/	/		(((((.((......)))))))	.((((.((......)))))).	..(((.((......)))))..	(((((.(((...).)))))))	.((((.((......))))).)	(((((.(........))))))	(.(((.((......))))).)	...((.((......))))...	(((((.((.(...))))))))	/
1JTJ	/	/	/	/	/	/	/	/	/	/		(((((((.........)))))))	.((((((.........)))))).	(((((((..(....).)))))))	((((((...........))))))	..(((((.........)))))..	.((((((..(....).)))))).	...((((..........))))...	((((((...(....)..))))))	(((((.(.........).)))))	(((((.............)))))
1JTW	/	/	/	/	/	/	/	/	/	/		.(((((....))))).	.((.((....)).)).	.(.(((....))).).	..((((....))).).	...(((....)))...	....((....))....	((..((....)).)).	....(((....)))..	..((((....))))..	(..(((....))).).
1JUR	/	/	/	/	/	/	/	/	/	/		((((((((....)))))..)))	(((.((((....))))...)))	.(((((((....)))))..)).	...(((((....)))))....	((.(((((....)))))...))	(((((((......))))..)))	(((..(((....)))....)))	.((.((((....))))...)).	((((.(((....))).)..)))	((((((.(....).)))..)))
1JWC	/	/	/	/	/	/	/	/	/	/		((((((....))))))	(((((......)))))	.(((((....))))).	.((((......)))).	(((((.(...))))))	((((........))))	/	/	/	/
1JZC	/	/	/	/	/	/	/	/	/	/		(((((...)))))	.((((...)))).	((((.....))))	((.((...)).))	.(((.....))).	/	/	/	/	/
1K2G	/	/	/	/	/	/	/	/	/	/		..(((....)))..........	.......(.(((......))).)	..(((....))).((....)).	.........(((......)))..	((..(((.(....).))).)).	....	(.((((....))))).........	..(((....))).(......).	....(((.(....).)))....	.....(((.......)))....
1K4A	/	/	/	/	/	/	/	/	/	/		(((((....)))))	.((((....)))).	((((......))))	((.((....)).))	(((.(....).)))	(((........)))	....	/	/	/
1K4B	/	/	/	/	/	/	/	/	/	/		(((((....)))))	.((((....)))).	((((......))))	((.((....)).))	(((.(....).)))	(((........)))	....	/	/	/
1K5I	/	/	/	/	/	/	/	/	/	/		((((((((((.....))))))))))	.((((((((.....)))))))).	(((((((((......)).))))))	(((((((((.......)))))))))	((((((.((.....)).))))))	/	/	/	/	/
1K6G	/	/	/	/	/	/	/	/	/	/		((((((((((....))))))))))	.((((((((....)))))))).	((((((((.......))))))))	(((((((.(....).)))))))	((((((.((....)).))))))	((((((((........))))))))	((((.((((....)))).))))	/	/	/
1K6H	/	/	/	/	/	/	/	/	/	/		((((((((((....))))))))))	.((((((((....)))))))).	((((((((.......))))))))	((((((.((....)).))))))	((((.((((....)))).))))	((((((((.(....).))))))))	((((((((........))))))))	/	/	/
1KAJ	/	/	/	/	/	/	/	/	/	/		(((((((....)).))))).............	(((..(((((.......))))).....))))..	(((((..........)))))..............	((.((((((((.......))))).....)))).	(((..(((((.(....)))))).....)))..	(((..((((.((....))))))......)))..	(((..(((.(((....))))))).....)))..	(((..((((((....).))))).....)))..	((.((((((((.(....))))))....)))).	((.((((((.((....))))))....)))).
1KKA	/	/	/	/	/	/	/	/	/	/		((((((((...))))))))	((((((.....))))))	(((((.......)))))	.((((((...)))))).	.(((((.....))))).	(((((.(....))))))	((((.........))))	(((((.(...).)))))	((((((....).)))))	.((((.......)))).
1KKS	/	/	/	/	/	/	/	/	/	/		((..((((((....))))))..))	((..((((((....)))))).)).	....((((((....)))))).....	((..(((((......)))))..))	((..(((((......))))).)).	....(((((......)))))....	.(..((((((....))))))..).	.(..(((((......)))))..).	(...((((((....)))))).)..	.(..((((((....)))))).)..
1KP7	/	/	/	/	/	/	/	/	/	/		((((.((....(((....)))..))).))))	((((.((...((((....))).))).))))	((((.......(((....))).....))))	((((.((..(.(((....)))).))).))))	((((..(....(((....)))..)..))))	.(((.((....(((....)))..)).))).	((((.....(((((....))).))..))))	(((((((..........))).)).))...	((((.(...(((((....))).))).))))	((((.((.....((....))....)).))))
1KPD	/	/	/	/	/	/	/	/	/	/		(((((((....)).))))).............	(((..(((((.......))))).....))))..	(((((..........)))))..............	((.((((((((.......))))).....)))).	(((..(((((.(....)))))).....)))..	(((..((((.((....))))))......)))..	(((..(((.(((....))))))).....)))..	(((.((((((.......)))))).....))))..	(((..((((((....).)))))).....)))..	((.((((((((.(....))))))....)))).
1KXK	/	/	/	/	/	/	/	/	/	/		.....(((.((((((...((((((((((((((((....)))))))..))))))))..)))))))))....	.....(((..(((((...(((((((((((((((....))))))..)))))))))..))))).))).....	.....(((.((((((...((((((((((((((....))))))..))))))))))..))))))))).....	.....(((..(((((...((((((((((((((....)))))..)))))))))..))))).))).....	.....(((.((((((.(.((((((((((((((....))))))..)))))))).)))))))))).....	.....(((..(((((.(.((((((((((((((....))))))..))))))))).))))).))).....	.....((..((((((...((((((((((((((....))))))..)))))))))..)))))).)).....	.....(((.((((((.(.(((((((((((((((....))))))..)))))))))).)))))))))).....	.....(((..(((((.(.((((((((((((((((....)))))..))))))))))).))))))).))).....	.....((..((((((...((((((((((((((....)))))..))))))))))..))))).)).....
1L1C	/	/	/	/	/	/	/	/	/	/		(..((((..((((....))))))))..).	((.((((..((((....))))))))))..))	...((((..((((....)))))))))....	....(((..((((....))))))).....	((.((....((((....))))....)).))	((.((....((((....))))....))))	.........((((....)))........	(..(((...((((....)))).))..).	((..(((..((((....))))))))...))	((.(((...((((....)))).)))..))
1L1W	/	/	/	/	/	/	/	/	/	/		((((..((((((....))))))..)))).	((((.((.(.((((....))))))..)))).	((((..((((((......)))))..)))).	((((...(((((....)))))...)))).	(((...((((((....)))))).)..))).	.(((..((((((....))))))..)))..	((((..(.((((....)))).)..))))).	((((.((((((....).))))).)))).	((((.((.((((....)))))).).))).	((.(..((((((....))))))..).)).
1LDZ	/	/	/	/	/	/	/	/	/	/		(((((....(((((....)))))..))))))	((((((...(((((....)))))).)))))	.((((....(((((....)))))..)))).	((((.(...(((((....)))))..)))))	((((.....(((((....)))))...))))	.(((((...(((((....)))))).)))).	(((((....((.((....)).))..)))))	(((((....(((.(....).)))..)))))	..(((....(((((....))))).)))..	(((((....((((......)))).)))))
1LNG	/	/	/	/	/	/	/	/	/	/		..((((..(((((.(((((((((....)))))))))..)))))....((((((.....(((.....(((....)))......)))..)))))).))))	..((((..(((((.(((((((((....)))))))))..)))))....((((((..(((..((.....(((....))).))...))).)))))).))))	..((((..(((((.((((((((((....)))))))))..))))).....((((((..(((........(((....)))......))).)))))).))))	((((((((((((.(((((((((....)))))))))..)))))...))).)))))..((.(((..((((.....))).))...))).))..........	((((((((((((.(((((((((....)))))))))..)))))...))).)))))..((.(((..(((.(...........))))).))).)).....	((((((((((((.(((((((((....)))))))))..)))))...))).)))))..((.(((..((.((...........)))).))).)).....	((((((((((((.(((((((((....)))))))))..)))))...))).)))))..((.(((..(.(((..........)))).))).)).....	((((((((((((.(((((((((....)))))))))..)))))...))).)))))..((.((((....((...........)))..))).)).....	..((((..(((((.(((((((((....)))))))))..)))))....((((((..(((((....))(((....)))......))).)))))).))))	..((((..((((..(((((((((...)))))))))..))))......((((((.....(((.....(((....))).....)))..)))))).))))
1M5L	/	/	/	/	/	/	/	/	/	/		((((....((((.((((....)))))...))))..))))	((((....((((..(((((...))))).))))..))))	((((....(((..((((....))))....)))..))))	.(((....((((.((((....)))))...))))..))).	((((....((((.((((....))).)..))))..))))	((((....((((.(((........))).))))..))))	((((....((((..(((((....)))))))))..))))	((((....((((..((((.....)))).))))..))))	((((....((((..(((....)))....))))..))))	((((....((((.((((....)).))..))))..))))
1M82	/	/	/	/	/	/	/	/	/	/		((((((((((....)))).))))))	((((((((((....))).)))))))	.((((((((....)))).))))).	.((((((((....))).))))).	(((((((((....)).))))))))	(((((((((.....).)))))))))	(((((.(((....)))..))))))	(((.((((((....)))).)).)))	(((.((((((....))).))).)))	((((((((((.......)))))))))
1MFJ	/	/	/	/	/	/	/	/	/	/		..(((((((....)))))))	...((((((....)))))).	....(((((....)))))..	..(.(((((....))))).)	..((((((......))))))	..(((((.(....).)))))	/	/	/	/
1MFK	/	/	/	/	/	/	/	/	/	/		(((((.(((....)))))))))	(((((.((((....)))))))))	(((((.((......)))))))	(((((.(((......))))))))	.(((((.(((....)))))))).	.((((.((((....)))))))).	(((((..(((....))).)))))	.(((((.((......))))))).	.((((.(((......))))))).	(((((..((......)).)))))
1MFY	/	/	/	/	/	/	/	/	/	/		(((.((((..((((....))))..)))))))	....((((..((((....))))..))))...	.((.((((..((((....))))..)))))).	(((.((((((.(((....))))).))))))	(((.(((((.((((....))))).)))))))	(((.(((...((((....))))...))))))	((..((((..((((....))))..)))).))	....((((((.(((....))))).))))...	....(((((.((((....))))).))))...	....(((...((((....))))...)))...
1MNX	/	/	/	/	/	/	/	/	/	/		((((.......((((((((....))))))))......))))	((((.....(.((((((((....))))))).).....))))	.(((.......((((((((....))))))))).......))).	((((.....(.((((((((....))))))))......))))	((((.(.....((((((((....))))))))).....).))))	((((........((((((....))))))))........))))	(((........((((((((....))))))))........)))	((((.........((((((....))))))).........))))	((((.....(.((((((((....))))))))..)....))))	.(((.....(.((((((((....))))))).).....))).
1MT4	/	/	/	/	/	/	/	/	/	/		((((((((........))))))))	.(((((((........))))))).	((((((((.(....).))))))))	((((((((.((...))))))))))	((((((((.(......)))))))))	(((((.((........)).)))))	((((((((..(....)))))))))	((((((.(........).))))))	((((((((.(...)..))))))))	((((((((..(...).))))))))
1N66	/	/	/	/	/	/	/	/	/	/		((((..(((....)))..))))	.(((..(((....)))..))).	((((((((....))))..))))	(((((((......)))..))))	(((...(((....)))...)))	((((..((......))..))))	((((...((....))...))))	((((.(((....)))...))))	.(((((((....))))..))).	((((..(.(....).)..))))

PDB	DNA model: RNAfold MFE	subopt #2	subopt #3	subopt #4	subopt #5	subopt #6	subopt #7	subopt #8	subopt #9	subopt #10
1N77	/	/	/	/	/	/	/	/	/	/
1N8X	/	/	/	/	/	/	/	/	/	/
1NA2	/	/	/	/	/	/	/	/	/	/
1NBR	/	/	/	/	/	/	/	/	/	/
1NC0	/	/	/	/	/	/	/	/	/	/
1NYB	/	/	/	/	/	/	/	/	/	/
1OOA	/	/	/	/	/	/	/	/	/	/
1OQ0	/	/	/	/	/	/	/	/	/	/
1OSW	/	/	/	/	/	/	/	/	/	/
1OW9	/	/	/	/	/	/	/	/	/	/
1P5M	/	/	/	/	/	/	/	/	/	/
1P5N	/	/	/	/	/	/	/	/	/	/
1P5P	/	/	/	/	/	/	/	/	/	/
1P6V	/	/	/	/	/	/	/	/	/	/
1PJY	/	/	/	/	/	/	/	/	/	/
1Q75	/	/	/	/	/	/	/	/	/	/
1QC8	/	/	/	/	/	/	/	/	/	/
1QFQ	/	/	/	/	/	/	/	/	/	/
1QWA	/	/	/	/	/	/	/	/	/	/
1QWB	/	/	/	/	/	/	/	/	/	/
1R2P	/	/	/	/	/	/	/	/	/	/
1R4H	/	/	/	/	/	/	/	/	/	/
1R7W	/	/	/	/	/	/	/	/	/	/
1R7Z	/	/	/	/	/	/	/	/	/	/
1RFR	/	/	/	/	/	/	/	/	/	/
1RHT	/	/	/	/	/	/	/	/	/	/
1RKJ	/	/	/	/	/	/	/	/	/	/
1RNG	/	/	/	/	/	/	/	/	/	/
1RNK	/	/	/	/	/	/	/	/	/	/
1ROQ	/	/	/	/	/	/	/	/	/	/
1S03	/	/	/	/	/	/	/	/	/	/
1S2F	/	/	/	/	/	/	/	/	/	/
1S9S	/	/	/	/	/	/	/	/	/	/

PDB	RNA model: RNAfold MFE	subopt #2	subopt #3	subopt #4	subopt #5	subopt #6	subopt #7	subopt #8	subopt #9	subopt #10
1N77	(((((((.(((((........)))))(((((.......)))))....(((((.......)))))))))))))....	((((((..(((((........)))))(((((.......)))))....(((((.......))))).))))))....	(((((((..((((........)))).(((((.......)))))....(((((.......))))))))))))....	(((((((.(((((........)))))(((((.(...).)))))....(((((.......))))))))))))....	(((((((.(((((........))))(((((.(....))))))....(((((.......))))))))))))....	(((((((.(((((........))))(((((.......)))))....((((......))))).)))))))....	(((((((.(((((........)))))((((.((....))))))....(((((.......)))))))))))....	(((((((.(((((........)))))(((.(((....))))))....(((((.......)))))))))))))....	(((((((.(((((........)))).((((.......))))....(((((.......)))))))))))))....	(((((((.(((((.(....).)))))(((((.......)))))....(((((.......)))))))))))....
1N8X	(((((((.((((((....)))))))...)))))))	(((((((.((((((....)))))..)).)))))))	(((((((.((((((....))))..))).)))))))	(((((((.((((((....))))))..).)))))))	(((((((.((.((((....))))...)).)))))))	(((((((.(.(((((....)))))...).)))))))	.((((((.((((((....)))))))...)))))).	.((((((.((((((....)))))..)).)))))).	((((((..((((((....)))))))....))))))	(((((((..((((((....))))))....)))))))
1NA2	((((((....((.....))....)))))).	((((((.....(.....).....)))))).	((((((.................)))))).	.(((((....((.....))....)))))..	((((((......((...))....)))))).	((((((...((......))....)))))).	((((((..((.......))....))))))).	(((((.....((.....)).....))))).	((((((.((.........))....)))))).	((((((...(.(.....))....)))))).
1NBR	((((((.(((((......))))))))))	.(((((.(((((......)))))))))).	((((((..((((......)))).))))))	((((((.((((........)))))))))))	(((((..(((((......))))).)))))	((((((...(((......)))..))))))	((((((.(.(((......))).)))))))	((((((.((((((...).))))))))))))	((((((.(((.(......).)))))))))	(((((((..(((......)))).))))))
1NC0	(((((((((.....))).))))))	.((((((((.....))).))))).	((((((((.......)).))))))	((((((.((......)).))))))	((.((((((.....))).))).))	(((.(((((.....))).)).)))	(((((((((....).)).))))))	/	/	/
1NYB	((((((((((......))))))))))	((((((((.........))))))))	((((((((.(.....)))))))))	.((((((((......)))))))).	.(((((((........))))))).	.(((((((.(.....)))))))).	(((.(((((......))))).)))	((.((((((.......))))))).))	(((((((.((.....)))))))))	((((((.(((.....)))))))))
1OOA	(((((....((((....))))...)))))	(((((.(..((((....)))).).)))))	((..((((......))))..)).......	(((((.(.((.((....)))).).)))))	(((((.(.(.(((....)))).).)))))	(((((....(((......)))...)))))	.((((....((((....))))...)))).	(((((.(.(..((...))..).).)))))	(((((.....(((....))....)))))	(((((..(.((((....)))).))))))
1OQ0	((((((...))))))	(((((.....)))))	.(((((...))))).	.((((.....)))).	..((((...))))..	((((.......))))	(((.......)))..	..(((.....)))..	(((.((...)).)))	(.((((...)))).)
1OSW	((((.(((....)))...))))	((((.(((....))..).))))	((((.(.(.......).).))))	((((..((....))....))))	.(((.(((....)))...))).	((((.(...........).))))	(((..(((....)))....)))	.(((.(((....))..).))).	((((.(.(....)...).))))	((((.(((....))...)))))
1OW9	((((...(((....)))..))))	.(((...(((....)))..))).	..((...(((....)))..))..	((((...((......))..))))	((((....((....))...))))	(((....(((....)))...)))	(.((...(((....)))..)).)	/	/	/
1P5M	(((((.((((.....(((((..(((.((....)).)))..).)))))))))))))))	(((((.((((.....((((...(((.((....)).)))....))))))))))))))	(((((.((((.....((((.(.(((.((....)).)))..).))))))))))))	(((((.((((.....(((.(..(((.((....)).)))..)..))))))))))))	(((((.((((.....(((.(..(.(.((....)).)))....))))))))))))	(((((.((((.....(((....((.(.((....)).))).....))))))))))))	.((((.((((.....(((((..(((.((....)).)))..).)))))))))))).	.((((.((((.....((((...(.(.(.((....)).)))....)))))))))))).	(((((.((((...(((......(((.((....)).)))..)))...))))))))))	(((((.((((.....(((((..((..((....))..))..).))))))))))))))
1P5N	((((...((((((.......))))))....))))	((((((.....))).))).(((((....)))))	((((....(((((.......))))).....))))	((((...((((.(((....)))))))....))))	((((((.......)).))).)..(((((....)))))	((((...((((((.(....)))))))....))))	.(((...((((((.......)))))).....))).	((((.(.((((((........))))))..).))))	((((((.....))).))).)..((((......))))	((((.....(((.(((....)))))).....))))
1P5P	(((((.((((.....(((((..(((.((...((((((.......))))))....)).)))..).)))))))))))))	(((((.((((.....((((...(((.((...((((((.......))))))....)).)))....)))))))))))))	(((((.((((.....((((......)))).((((((.......)))))).((((((...)))))))..)))))))))	(((((.((((.....(((((..(((.((....((((((.......))))))....)).)))..).)))))))))))))	(((((.((((.....((((...(.(.((....((((((.......))))).......)).)))....))))))))))))))	(((((.((((.....((((......))))..(((((((.......)))))).(((((.....))))))..)))))))))	(((((.((((.....((((.(.(((.((...((((((.......))))))....)).)))..).)))))))))))))	(((((.((((.....((((.......))))...(((((.......))))))..((((((...)))))..))))))))))	(((((.((((.....(((.(..(((.((...((((((.......))))))....)).)))..)..)))))))))))))	(((((.((((.....(((((..(((.((...((((.(((....)))))))....)).)))..).)))))))))))))
1P6V	.......((((((....))))))...(((((.......)))))	.....(.((((((....))))))..)(((((.......)))))	...((..((((((....)))))))).(((((.......)))))	....((.((((((....))))))).))((((.......)))).	........(((((....))))))....(((((.......)))))	.......((((((....)))))))....((((.......)))).	....(..((((((....)))))))).(((((.......)))))	(.(((((...((((....))))))))))(((((.......)))))	(..((..((((((....)))))))))(((((.......)))))	(......((((((....)))))))..)(((((.......)))))
1PJY	(((((((((....)))))))))	.((((((((....)))))))).	((((((((......))))))))	((((.((((....)))).))))	(((((.(((....))).)))))	/	/	/	/	/
1Q75	(((((.....)))))	.((((.....)))).	((((.......))))	(((.(.....).)))	/	/	/	/	/	/
1QC8	(((.((((((((....))))))))))	(((.((((((......)))))))))	....(((((((....)))))))...	.((.(((((((....))))))))).	....((((((......))))))...	.((.((((((......)))))))).	(((.((((((........))))))))	(((.(((((((...)).))))))))	(((.(((((((...).))))))))	..(.(((((((....))))))))..
1QFQ	(((((.....)))))	.((((.....)))).	((((.......))))	(((((....).))))	/	/	/	/	/	/
1QWA	((((((((....)).))))))	((((((((......).))))))	((((((.........))))))	((((((..(....).))))))	.(((((((....)).))))).	((((((.(.....).))))))	(((((.(.........))))))	.((((((......).))))).	(((((((....)...))))))	((((((...(...).))))))
1QWB	((((((.............))))))	((((((...((......)).))))))	((((((....(.....)...))))))	.(((((..............))))).	((((((.....(......)..))))))	(((((((.(..........).)))))))	((((((....(....)....))))))	((((((..(.........).))))))	((((((....(.......)..))))))	(((((((.......)......)))))))
1R2P	(((((((((((((....))))))).)))))))))	(((((((((((((....))))))).)))))))))	(((((((.((((((....))))))...)))))))	((((((((.(((((....))))))...))))))))	((((((((((((((....))))).))))))))))	(((((((((((((......)))))..))))))))	(((((((((((((......)))))..))))))))	.((((((((((((((....))))))..)))))))).	.((((((((((((((....))))).)))))))).	..((((((((((((....))))))..)))))..
1R4H	(((....)))	((.....)).	....	.((....)).	((......))	.((.....))	(......)..	.(.....)..	.(......).	/
1R7W	(((((((......(((((....))))))))))))	((((((......((((((....)))))))))))	.((((((......(((((....))))))))))).	((((((.....(.(((((....))))))))))))	(((((((.......((((....)).)))))))	((((((.......(((((....))))).))))))	((((((...((...((((....))))))))))))	((((((....((..((((....)))))))))))	.(((((......((((((....))))))))))).	(((((((......((((......)))))))))))
1R7Z	(((((((......(((((....))))))))))))	((((((......((((((....)))))))))))	.((((((......(((((....))))))))))).	((((((.....(.(((((....))))))))))))	(((((((.......((((....)).)))))))	((((((.......(((((....))))).))))))	((((((...((...((((....))))))))))))	.(((((......((((((....)))))))))).	(((((((......((((......)))))))))))	(((((((.(......(((....))))))))))))
1RFR	(((((...(((((....))))))...))))))	(((((...((((.......))))...)))))	.((((...((((((....)))))...)))).	(((((...(((.........))...)))))	.((((...((((.......)))).)))).	..(((...(((((....))))))...)))..	(((((...((((.(...)))))...)))))	((((....(((((....))))))....))))	.((((...(((.........))...)))).	(((((....((((....)))).....)))))
1RHT	.((((((.((....))))))))..	..(((((.((....)))))))...	.((((((.(......)))))))..	.((((((..........))))))..	..(((((.(......)))))))...	((.((((.((....))))))))..	(.(((((.((....))))))))..	.((((((..(....).)))))))..	..(((((..........))))))...	((((((((.((....)))))))).)
1RKJ	(((((((((....)).))))))	((((((((......).))))))	((((((.........))))))	((((((..(....).))))))	.(((((((....)).))))).	((((((.(.....).))))))	(((((.(.........))))))	.((((((......).))))).	(((((((....)...))))))	((((((...(...).))))))
1RNG	((((....))))	.((....))...	.(((....))).	(((......)))	....	..((....))..	.((......)).	...((....)).	(.......)...	.(((....)).)
1RNK	(((((((....)).)))))...............	(((((.........)))))..............	(((..(((((.......))))).......)))...	......((((((((.............))))))	((.(((((((.......)))))).......))))...	(((((((....)).))))).((......))......	(((.((((((.......)))))....))))...	(((..(((((.(....))))))......)))...	(((..((((.((....)))))))......)))...	(((..(((.(((....))))))......)))...
1ROQ	(((((....)))))	((((......))))	.((((....)))).	(((........)))	....	((((.(...)))))	.(((......))).	(......)......	(((((...).))))	(((.(....).)))
1S03	((((((.((.((...((((((((....))))))))..))))))))))	((((((.((.((....(((((((....)))))))...))))))))))	((((((.((.((...((((((((......))))))))..))))))))))	((((((.((.((..(((((((((....))))))).)))))))))))	((((((.((.((..((((((((((....)))))))).)))))))))))	((((((.((....(((((((((((....)))))))).)))).))))))	((((((.((....(((((((((((....)))))).))))).))))))	((((((.((.(..((((((((((....)))))))).)))))))))))	((((((.((.(..((((((((((....))))))).)))))))))))	((((((.((.((....(((((((......))))))...))))))))))
1S2F	((((((.(((....)))))))))	((((((.((......))))))))	((((((.(........)))))))	.(((((.(((....)))))))).	.(((((.((.......)))))))	.(((((.(((....))).)))))	((((((...........))))))	.((((..((.......)).))))	((((((.(.......).))))))	.((((..(((....)))..))))
1S9S	.((((..((((((....))))))..))))......((((..(((.(((((((((....)))))....)))))))))))((((((((((....))))))))))	.((((..((((((....))))))..))))......((((..(((.(((((((((....)))))....))))))))))))).((((((((((....))))))))))).	.((((..((((((....)))))))..))))......((((.((.(.((((((((((....)))))....))))))))))((((((((((....))))))))))	.((((..((((((....))))))..)))......((((.(.((.(((((((((....)))))....)))))))))))(((((((((....))))))))))	.((((..((((((....))))))..))))......((((.((...(((((((((....))))).....))).)))))((((((((((....))))))))))	.((((..((((((....))))))..))))......((((..(((.((((((((....)))))....)))))))))((((((((((......))))))))))	.((((..((((((....))))))..))))......((((..(((.(((((((((....)))))....))))))))))..((((((((....))))))))..	.((((..((((((....))))))..))))......((((.((.(.((((((((((....)))))....))))))))))).((((((((....)))))))).	.((((..((((((....))))))..)))))......((((.(.((.(((((((((....)))))....)))))))))))).((((((((((....))))))))).	.((((..((((((....))))))..)))))......((((.((.(((.(.(((((....))))).)..)))..))))))((((((((((....))))))))))))

	DNA model											RNA model									
PDB	RNAfold MFE	subopt #2	subopt #3	subopt #4	subopt #5	subopt #6	subopt #7	subopt #8	subopt #9	subopt #10		RNAfold MFE	subopt #2	subopt #3	subopt #4	subopt #5	subopt #6	subopt #7	subopt #8	subopt #9	subopt #10
1SCL	/	/	/	/	/	/	/	/	/	/		((((((...((.(.....).)).))))))	((((((...((.(....)..)).))))))	((((((......(....).....))))))	((((((.................))))))	((((((...((..........)).))))))	((((((((..........))...))))))	((((((...(..(.....)..).))))))	.(((((...((.(.....).)).))))).	((((((((.....))........))))))	((((((((.......))......))))))
1SLP	/	/	/	/	/	/	/	/	/	/		.((((((....)).)))).	(((((((....)).)))))	.(((((......).)))).	((((((......).)))))	..(((((....)).)))...	.((((.........)))).	(((((.........)))))	..((((......).)))..	...((((....)).))...	.((((..(...)..)))).
1SYZ	/	/	/	/	/	/	/	/	/	/		(((((.(((.....)))..)))))	.((((.(((.....)))..)))).	(((((..((......))..)))))	((((((((........))..)))))	(((((.((.......))..)))))	((((..(((.....)))...))))	(((((..((.....))...)))))	(((((((.(.....)))..)))))	((((((.((.....)))..)))))	((((.((((.....)))..)))))
1SZY	/	/	/	/	/	/	/	/	/	/		(((((((.......)))))))	(((((((.(....))))))))	(((((((.(...).)))))))	.((((((.......)))))).	((((((.........))))))	.((((((.(....))))))).	.((((((.(...).)))))).	(((.(((.......))).)))	/	/
1T28	/	/	/	/	/	/	/	/	/	/		(((((((((.............))))))))))	(((((((((..(.........).))))))))))	((((((((((...........))).))))))))	((((((((((...........)).))))))))	((((((((((...........).))))))))))	((((((((((..(....)......)))))))))))	(((((((((................))))))))))	.((((((((..............)))))))).	((((((((((......)......))))))))))	((((((((.................))))))))
1TFN	/	/	/	/	/	/	/	/	/	/		.((((((.((....))))))))..	..(((((.((....)))))))...	.((((((.(......)))))))..	.((((((.........))))))..	..(((((.(......)))))...	((.((((.((....))))))))..	(.(((((.((....))))))))..	.((((((..(....).))))))..	..(((((.........)))))...	(((((((.((....)))))))).)
1TJZ	/	/	/	/	/	/	/	/	/	/		(((..((((....))))..)))	(.((.((((....)))))).).	((((.((((....)))))).))	((((.((((....))))).)))	.((..((((....))))..)).	..((.((((....))))))...	(((...(((....)))...)))	((...((((....)))...))	.(((.((((....)))))).).	...(.((((....))))))....
1TLR	/	/	/	/	/	/	/	/	/	/		((((...(((....)))..))))	((((.(.(((....))).)))))	(((((((........))).))))	((((....((....))...))))	((((...............))))	((((.(..((....))..)))))	.(((...(((....)))..))).	((((((..((....)))).))))	(((((..(((....)))).))))	((((.((........))..))))
1TXS	/	/	/	/	/	/	/	/	/	/		((((..((((...(((((....)))))...))))))))	((((..((((...((((......))))...))))))))	.(((..((((...(((((....)))))...))))))).	((((..((((...(((........)))...))))))))	((((...(((...(((((....)))))...))).))))	(((...((((...(((((....)))))...)))).)))	.(((..((((...((((......)))))...))))))).	((((..(((....(((((....)))))....)))))))	((((..(((....(((((....)))))..)))..))))	((((..((((....((((....)))))....))))))))
1U2A	/	/	/	/	/	/	/	/	/	/		(((((((......)))))))	.((((((......)))))).	((((((........))))))	((((((((...)).))))))	((((((((...).)))))))	((((.((......)).))))	(((((((.(...))))))))	.(((((........))))).	/	/
1U63	/	/	/	/	/	/	/	/	/	/		((((((((((..((((((((....)))))).)))......))))))))))	((((((((((..((((((((....))))).))))......))))))))))	((((((((((..((((((((......))))).)))......))))))))))	((((((((((..((((((((......)).))))......))))))))))	.(((((((((..((((((((....)))))).)))......))))))))).	.((((((((..((((((((....))))).))))......))))))))).	((((((((((..(((.((((....)))))..)))......))))))))))	((((((((((..((((.(((....)))..))))......))))))))))	(((((((((...((((((((....)))))).)))........)))))))))	(((((((((...((((((((....)))).))))........)))))))))
1ULL	/	/	/	/	/	/	/	/	/	/		(((((..(((((((....))))..)))...)))))	(((((..((.((((....)))).)).....)))))	.((((..((((((((....))))..)))...)))).	(((((..(((((((....)))).).))...)))))	(((((..(((.(((....)))....)))...)))))	(((((..((.((((....))))...))...)))))	(((((...((((((....))))..))....)))))	(((((..((((((((....)))).)).)...)))))	(((((...((((((....)))).)).....)))))	(((((..(((..((....))....))))...)))))
1UN6	/	/	/	/	/	/	/	/	/	/		((((((....((....))(((((((..((.((((((...)))))).)))))))))))))))	((((((....((....))(((((((.....((((((...))))))...))))))))))))))	((((((....((....))((((((((.....((.((....)).))....)))))))))))))	((((((....((....)).((((((....))).)))..((((((.....)))))))))))))	((((((....((....)).((.(((....))).))...((((((.....))))))))))))	((((((....((....))(((((((.(...((((((...)))))))..))))))))))))))	((((((....((....))((((((.((...((((((...))))))..))))))))))))))	((((((....((....))((((((((...(.((((((...)))))))).).)))))))))))))	((((((....((....))((((((...((.((((((...)))))).)).))))))))))))	((((((....((....))((((((((...(.((((((...)))))))..)))))))))))))
1UUU	/	/	/	/	/	/	/	/	/	/		((((((((...))))))))	(((((((.....)))))))	.(((((((...))))))).	.((((((.....)))))).	(((((.((...)).)))))	((((.(((...))).))))	/	/	/	/
1VOP	/	/	/	/	/	/	/	/	/	/		(((((...)))))	((((.....))))	.((((...)))).	....	(((.......)))	..(((...)))..	.(((.....))).	(((.(...).)))	..((.....))..	/
1WKS	/	/	/	/	/	/	/	/	/	/		((((((.....))))))	.(((((.....))))).	(((((.......)))))	((((.(.....).))))	((((.........))))	(((.((.....)).)))	((((.(......)))))	(((.((......)))))	.((((.......)))).	((((((....))).)))
1WZ2	/	/	/	/	/	/	/	/	/	/		((((((((......((((......)))).(((((.((..((((((..(((....)))....)))))).)).))))))))))))))....	((((((((......((((......)))).(((((.((..((.((((.(((....)))....)))).)))).))))))))))))))....	((((((((......((((......)))).(((((.....((.((((.(((....)))....)))).))...))))))))))))))....	((((((((......((((......))))((((((((..((.(((((.....))))).))..)))))......))))))))))))....	((((((((......((((......)))).(((((.((..((.((((((((...))))....)))).)))).)))))))))))))....	(((((((.......((((......))))((((((.......))))))(((....)))...(((((.......))))))))))))))....	((((((((......((((......))))((((((.......))))))(((....)))....((((.......)))))))))))))))....	(((((((.......((((......)))).(((((.((..((((((..(((....)))....)))))).)).))))).)))))))....	((((((((......((((......))))((((((((..((.(((((.....))))).))..))))......))))))))))))....	((((((((......((((......)))).(((((.((...(((((..(((....)))....)))))..)).))))))))))))....
1XHP	/	/	/	/	/	/	/	/	/	/		((((.((((.(((.....)))..)))).))))	.(((.((((.(((.....)))..)))).))).	((((.((((..((.......))..))))).))))	..((.((((.(((.....)))..)))).))..	((((.((((((........))..)))).))))	((((.((((.((.......))..)))).))))	((((.(((..(((.....)))...))).))))	((((.((((..((.....))...)))).))))	((((..(((.(((.....)))..)))..))))	.....((((.(((.....)))..))))......
1XSG	/	/	/	/	/	/	/	/	/	/		(((((.(((((((....))))))))))))	.((((.((((((....)))))))))).	(((((.(((((......))))))))))	(((.(.((((((....))))))).)))	((.((.((((((....)))))))).))	((((..((((((....)))))).))))	/	/	/	/
1XSH	/	/	/	/	/	/	/	/	/	/		((((((.(((((....)))))))))))	(((((((.((((....))))))))))))	.(((((.(((((....))))))))).	(((((.((((((....))))))))))	((((((.((((......))))))))))	((((((..((((....)))).))))))	(((.((.(((((....))))))).)))	((.(((.(((((....))))))).))	.((((((.((((....)))))))))).	.((((.((((((....)))))))))).
1XWP	/	/	/	/	/	/	/	/	/	/		((((......)))).	(((........))).	((((.(...))))).	.(((......)))..	((.(......).)).	/	/	/	/	/
1XWU	/	/	/	/	/	/	/	/	/	/		((((.......)))).	((((.(...).)))).	(((.........))).	....	.(((.......)))..	(((..(...)..))).	((...........)).	.(((.(...).)))..	.((.........))..	(........)......
1YMO	/	/	/	/	/	/	/	/	/	/		((((((....((.....))....))))))..................	(((.........)))((((((((((..............)))))))))).	.............((((((((((..............)))))))))).	(((.........)))((((((((..............)))))))).	((((((....((.....))....))))))...((......)).....	..............((((((((...............)))))))).	(((.........)))((((((((..(.......)...)))))))))).	((((((.....(.....).....))))))..................	((((((.................)))))).................	..............((((((((..(.......)...)))))))))).
1YN1	/	/	/	/	/	/	/	/	/	/		((((((.....))))))	(((((.......)))))	.(((((.....))))).	(((((.(....))))))	..((((.....))))..	.((((.......)))).	(((.((.....)).)))	/	/	/
1YNC	/	/	/	/	/	/	/	/	/	/		((((((((((((.........)))).)))))))	((((((((((((.((...)))))).)))))))	(((((((((((.........))).))))))))	(((((((((((.((...))))).))))))))	(((((((((((.(.....))))).)))))))	((((((((((((.(.....)))).))))))))	((((((.((((.........))))..))))))	((((((.((((.((...))))))..))))))	((((((.((((.(.....))))).))))))	((((((((....((...))....))))))))
1YNG	/	/	/	/	/	/	/	/	/	/		((((((((((((.........)))).)))))))	((((((((((((.........))).))))))))))	((((((.((((.........))))..))))))	((((((((((((.....)).))).))))))))	((((((((((((.....).)))).)))))))	.((((((((((.........))))).)))))).	((((((((((((.....)).)).)))))))))	((((((((((((.....).))))..)))))))))	(((((((((((.((...))))))).)))))))	(((((((.(((.........)))..))))))))
1YSV	/	/	/	/	/	/	/	/	/	/		(((((((((((.....)))))))))))	.((((((((((.....)))))))))).	((((((((((........))))))))))	(((((((((((....).))))))))))	(((((((((.(.....).)))))))))	((((((((((.........)))))))))	(((.(((((((.....))))))).)))	((((((((.(((.....))).)))))))	(((((((.((((.....)))).))))))	((((((((.((.....)).))))))))
1Z2J	/	/	/	/	/	/	/	/	/	/		(((((((((((((((((((....)))))))))))...))))))))	(((((((((((((((((((....))))))))))))...))))))))	((((((((.(((((((((((....)))))))))))....)))))))	.((((((((((((((((((....))))))))))...)))))))).	.((((((((((((((((((....)))))))))))...))))))).	((((((.((((((((((((....))))))))))))....)))))))	((((((((((((((((((....))))))))))..)).))))))))	(((((((((((((((((((....))))))))))..).))))))))))	(((((((((((((((((....))))))))..))).))))))))	((((((((((((((((((....))))))))))..)).))))))))
1Z30	/	/	/	/	/	/	/	/	/	/		((((((((....))))))))	.((((((....)))))).	..(((((....)))))..	((((((......))))))	((((.((....)).))))	.((((((....))))).)	(((((.(....).)))))	(.(((((....))))).)	...((((....))))...	/
1Z31	/	/	/	/	/	/	/	/	/	/		(((((.(((..(((....)))...))))))))	(((((.((((((....)))......))))))))	.((((.(((..(((....)))...))))))).	((((..(((..(((....)))...))).))))	(((((.(((((......)).....))))))))	.((((.((((((....)))......))))))).	(((((((...)))))))(((....)))....	(((((.(((...((....))....))))))))	(((((.((.(.(((....)))...))))))))	(((((.((...(((....)))....)))))))
1ZBN	/	/	/	/	/	/	/	/	/	/		((((((.(.((((....))))))))))))))	((((((.(.(((......)))))))))))	((((((...((((....))))))))))))	((((((...(((......))).))))))	((((((.(..(((....))).))))))))	.((((((.(.((((....))))))))))).	((((((.(..((......)).)))))))	.(((((.(.(((......))))))))).	((((((....(((....))..))))))	.((((((...((((....)))).))))).
1ZC5	/	/	/	/	/	/	/	/	/	/		((((((((((((((((....)))))))))))...))))))	(((((((((((((((((....))))))))))))...)))))	(((((((((((((((......))))))))))...))))))	(((((((((((((((......))))))))))))...)))))	(((((.((((((((((....))))))))))....)))))	(((..(((((((((((....))))))))))).....)))	.((((((((((((((((....))))))))))...))))).	((((.((((((((((((....))))))))))))....))))	.((((((((((((((((....))))))))))))...))))).	((((((((((((((((....))))))))))..)).)))))
1ZIF	/	/	/	/	/	/	/	/	/	/		((((....))))	.(((....))).	..((....))..	((.(....))).	(.((....))).	((.......)).	(((......)))	.((......)).	....	/
1ZIG	/	/	/	/	/	/	/	/	/	/		((((....))))	.(((....))).	..((....))..	((.(....))).	(.((....))).	((.......)).	(((......)))	.((......)).	....	/
1ZIH	/	/	/	/	/	/	/	/	/	/		((((....))))	.(((....))).	..((....))..	((.(....))).	(.((....))).	((.......)).	(((......)))	.((......)).	....	/
28SP	/	/	/	/	/	/	/	/	/	/		(((((....(((....)))....)))))	(((((..(.(((....)))..).))))))	((((.....(((....))).....))))	.((((....(((....)))....)))).	(((((..(.(.(((....)).).).)))))	((((.(...(((....)))....)))))	(((((.....((....)).....)))))	((((.(.(.(((....)))..).)))))	(((((...((((....))..)).)))))	((((.(.(.(((....)).).).)))))

	DNA model										RNA model									
PDB	RNAfold MFE	subopt #2	subopt #3	subopt #4	subopt #5	subopt #6	subopt #7	subopt #8	subopt #9	subopt #10	RNAfold MFE	subopt #2	subopt #3	subopt #4	subopt #5	subopt #6	subopt #7	subopt #8	subopt #9	subopt #10
2A9L	/	/	/	/	/	/	/	/	/	/	(((((((((...((((....))))))))...))))))	.((((((((...((((....))))))))...))))).	(((((.((((...((((....)))))))....)))))	((((((.......((((....)))).......))))))	((((((((((...((((....)))))))).)..)))))	((((((.(((...((((....)))))))....))))))	((((((((((....((((....))).))))...))))))	(((((((((....((((....)))).)))...))))))	((((((.((....((((....))))...))..))))))	((((((((((.(..((((....))))))))...))))))
2ADT	/	/	/	/	/	/	/	/	/	/	(((((..((((((((((((....))))))))))))...)))))	(((((.((((((((((((((....)))))))))))).).)))))	.((((..((((((((((((....))))))))))))...))))).	((((...((((((((((((....))))))))))....))))	.((((.((((((((((((((....)))))))))))).).)))).	(((....((((((((((((....))))))))))))....))).	.(((...((((((((((((....))))))))))))....))).	((((((.((((((((((((....))))))))))))..)))))	((((((.((((((((((((....)))))))))))))..))))	((((..((((((((((((((....)))))))))))).)..))))
2AHT	/	/	/	/	/	/	/	/	/	/	((((((((((....)))).)))))))	((((((.((((....))))..))))))	((((((((.(((....)))..)))))))	.((((((((((....)))).)))))).	((((((((((......))).)))))))	.(((((.((((....))))..))))).	((((((.(((......)))..))))))	((((((..(((....)))...))))))	.(((((((.(((....)))..)))))).	((((((((((......)))..))))))
2AKE	/	/	/	/	/	/	/	/	/	/	(((((((..((((.......))))(((((....)))))..........(((((.......))))))))))))).	(((((((..((((.......)))).(((((.......)))))......(((((.......))))))))))))).	(((((((..((((.......)))).((((((....).)))))......(((((.......))))))))))).	(((((((..((((.......))))(((((....)))))...........((((.......)))).))))))).	(((((((..((((.......))).((((....)))).........(((((.......)))))))))))).	((((((((((((.......))))(((((....)))))......((((.((....)))))).)).))))))).	(((((((..((((.......))))(((((....))).)..........(((((.......))))))))))).	((((((...((((.......))))(((((....))))).........(((((.......))))).)))))).	(((((((..((((.......))))(((((....)))))(....)...(((((.......))))))))))))).	(((((((..((((.......)))).((((.......))))......(((((.......))))))))))).
2ANN	/	/	/	/	/	/	/	/	/	/	((((..............))))	.(((..............))).	(((((........))...)))..	(((((.........))..)))..	((((.....(....)....))))	(((..............)))..	((((..(.........)..))))	((((.....(...).....))))	((((......(.....)...))))	(((((........)).....)))
2B6G	/	/	/	/	/	/	/	/	/	/	(((((((.....)))))))	.((((((.....)))))).	((((((.......))))))	..(((((.....)))))..	.(((((.......))))).	...((((.....))))...	((.((((.....)))).))	(((.(((.....))).)))	(.(((((.....))))).)	..((((.......))))..
2B7G	/	/	/	/	/	/	/	/	/	/	(((((((.....)))))))	.((((((.....)))))).	((((((.......))))))	..(((((.....)))))..	.(((((.......))))).	...((((.....))))...	((.((((.....)))).))	(((.(((.....))).)))	(.(((((.....))))).)	..((((.......))))..
2DER	/	/	/	/	/	/	/	/	/	/	(((((((..((((.........))))((((((.......))))))...(((((.......)))))))))))))..	(((((((.......(((.(((.(...((((((.......))))))..).))).)))..........))))))..	(((((((.......(((.(((..(..((((((.......))))))..).))).)))..........))))))..	(((((((..((((.(.....).))))((((((.......))))))...(((((.......))))))))))))..	(((((((..(....(((.(((.(...((((((.......)))))))..).))).)))....)....)))))))..	(((((((.......(((.(((.....((((((.......)))))).....))).)))..........)))))))..	(((((((..(....(((.(((.(...((((((.......))))))..).))).)))......)..))))))..	(((((((..(....(((.(((..(..((((((.......)))))))..).))).)))....)....)))))))..	(((((((..(....(((.(((..(..((((((.......))))))..).))).)))......)..))))))..	((((((...((((.........))))((((((.......))))))...(((((.......))))).))))))..
2DET	/	/	/	/	/	/	/	/	/	/	..(((((..((((.........))))((((((.......))))))...(((((.......))))))))))	..(((((.......(((.(((.(...((((((.......))))))..).))).)))..........)))))	..(((((.......(((.(((..(..((((((.......))))))..).))).)))..........)))))	..(((((..((((.(.....).))))((((((.......))))))...(((((.......))))))))))	..(((((..(....(((.(((.(...((((((.......)))))))..).))).)))....)....)))))	..(((((.......(((.(((.....((((((.......)))))).....))).)))..........)))))	..(((((..(....(((.(((.(...((((((.......))))))..).))).)))......)..)))))	..(((((..(....(((.(((..(..((((((.......)))))..).))).)))....)....)))))	..(((((..(....(((.(((..(..((((((.......))))))..).))).)))......)..)))))	..((((...((((.........))))((((((.......))))))...(((((.......))))).))))
2DR2	/	/	/	/	/	/	/	/	/	/	(((((((..((((.......))))(((((....)))))..........(((((.......)))))))))))).....	(((((((..((((.......)))).(((((.......)))))......(((((.......)))))))))))))....	(((((((..((((.......)))).((((((....).)))))......(((((.......)))))))))))....	(((((((..((((.......))))(((((....)))))...........((((.......)))).)))))))....	(((((((..((((.......))).((((....)))).........(((((.......)))))))))))....	((((((((((((.......))))(((((....)))))......((((.((....)))))).)).)))))))...	(((((((..((((.......))))(((((....))).)..........(((((.......))))))))))))....	((((((...((((.......))))(((((....))))).........(((((.......))))).)))))).....	(((((((..((((.......))))(((((....)))))(....)...(((((.......))))))))))))))....	(((((((..((((.......)))).((((.......))))......(((((.......))))))))))))....
2DRB	/	/	/	/	/	/	/	/	/	/	((((((((((((.......)))))))))))))....	((((((((((((.........)))))))))))))....	(((((.((((((.......)))))).))))))....	.(((((((((((((.......))))))))))).....	((((((((((((((...)..))))))))))))))....	/	/	/	/	/
2DU3	/	/	/	/	/	/	/	/	/	/	((((((((((.((((.((.(((((((....))))))).)).)))).))((((.......))))))))))))	((((((((((..(((.((.((((((((....))))))).)).)))..))((((.......))))))))))))	(((((((.((.((((.((.((((((((....))))))).)).)))).))((((.......)))).))))))	(((((((....((((.((.(((((((....))))))).)).))))..(((((.......))))))))))))	((((((((((.((((.((.(((((((....))))))).)).)))).))((((.(...).))))))))))))	((((((((((.((((.((..((((((....))))))..)).)))).))((((.......)))))))))))))	(((((((..(.((((.((.((((((...)))))).)).)))).)(((((.......))))))))))))	.(((((((((.((((.((.(((((((....))))))).)).)))).))((((.......)))))))))))).	((((((((((.((((((.(((((((....)))))))))).)))).))((((.......))))))))))))	(((((((..((((((...))))))((((((.......))))))....(((((.......)))))))))))
2DU5	/	/	/	/	/	/	/	/	/	/	(((((((..((((((...))))))((((((.......))))))....(((((.......))))))))))))	(((((((..((((((...))))))((((((.....).))))))....(((((.......)))))))))))	((((((((.((((((...)))))((((((.......))))))......((((.......))))))))))))	((((((((.((((((...))))))(((((((....).))))))....((((.......))))))))))))	(((((((..((((((...)))))).((((((.......)))))).....((((((.......))))))))))))	(((((((..((((((...))))))((((((.........))))))....((((.......)))))))))))))	(((((((..((((((...))))))).(((((((.....).)))))......(((((.......))))))))))))	(((((((..((((((...))))))((((((.(...).)))))....(((((.......))))))))))))	(((((((..(((((.....)))))((((((.......))))))....(((((.......))))))))))))	((((((((.((((((...)))))).((((((.......))))))......((((.......))))))))))))
2DU6	/	/	/	/	/	/	/	/	/	/	(((((((..((((((...))))))((((((.......))))))....(((((.......))))))))))))	(((((((..((((((...))))))((((((.....).))))))....(((((.......)))))))))))	((((((((.((((((...)))))((((((.......))))))......((((.......))))))))))))	((((((((.((((((...))))))(((((((....).))))))....((((.......))))))))))))	(((((((..((((((...)))))).((((((.......)))))).....((((((.......))))))))))))	(((((((..((((((...))))))((((((.........))))))....((((.......)))))))))))))	(((((((..((((((...))))))).(((((((.....).)))))......(((((.......))))))))))))	(((((((..((((((...))))))((((((.(...).)))))....(((((.......))))))))))))	(((((((..(((((.....)))))((((((.......))))))....(((((.......))))))))))))	((((((((.((((((...)))))).((((((.......))))))......((((.......))))))))))))
2ES5	/	/	/	/	/	/	/	/	/	/	(((((((((.....)))))))))	((((((((.......))))))))	.((((((((.....)))))))).	((((.((((.....)))).))))	(((.((((((.....)))))).)))	.((((((((.......)))))))).	(((((.(((.....))).)))))	((.((((((.....)))))).))	/	/
2EUY	/	/	/	/	/	/	/	/	/	/	((((((.((..((((....)))).))..))))))	((((((..(..((((....)))).)..))))))	(((((..((..((((....)))).))...)))))	.(((((.((..((((....)))).))..))))).	.(((((..(..((((....))))..)..))))).	((((((.....((((....)))).....))))))	((((((.((..(((......))).)).))))))	.((((..((..((((....)))).))...)))).	((((...((..((((....)))).))....))))	((((((.((...((((....)))..))..))))))
2EVY	/	/	/	/	/	/	/	/	/	/	(((((....)))))	((((......))))	.((((....)))).	(((........)))	....	.(((......))).	(((.(....).)))	((.((....)).))	/	/
2F87	/	/	/	/	/	/	/	/	/	/	((((....))))	(((......)))	.(((....))).	.((......)).	....	(.((...)).).	/	/	/	/
2F88	/	/	/	/	/	/	/	/	/	/	(((((((((((((....)))))..))))))))	((((((((((((((....))))))..))))))))	((((((((.(((((....)))))...))))))))	((((((((((((((......))))..))))))))	.(((((((((((((....)))))..))))))).	(((((((((((((......)))))..))))))))	((((((((((((((....))))).).))))))))	.(((((((((((((....))))))..))))))).	..(((((((((((....))))))..)))))))..	((((((((.((((((....))))))...)))))))
2FDT	/	/	/	/	/	/	/	/	/	/	(((((((.(.((((((.....)))))).))))))))	(((((((...((((((.....))))))..)))))))	((((((((.(.((((((.....)))))).)))))))	((((((..(.((((((.....)))))).).))))))	.((((((.(.((((((.....))))).)))))))).	(((((((.(.(((((.......)))))).))))))))	((((((....((((((.....))))))...))))))	((((((((.(.((((.(.....).))))).))))))))	((((((((.(..(((((.....))))))..))))))))	(((((((.(.((((.........)))).))))))))
2FEY	/	/	/	/	/	/	/	/	/	/	(((((.(((.(...((((((.......))))))).)))))))))	(((((.(((.....((((((.......))))))..))))))))	(((((.(((..((((.((((.......))))))))))))))))	(((((.(((..(((.((((((.......)))))))))))))))	(((((.(((..((.((((((.......))))))))))))))))	(((((.(((.(...(((((........))))))).))))))))	(((((..((.(...((((((.......)))))).)).)))))	(((((.(((.....(((((.........)))))..))))))))	(((((..((.....((((((.......)))))).)).)))))	(((((.(((.(....(((((.......))))).).))))))))
2FY1	/	/	/	/	/	/	/	/	/	/	((((((((.....))))))))	.(((((((.....))))))).	(((((((.(....))))))))	(((((((........)))))))	((((.(((.....))).))))	/	/	/	/	/
2G1W	/	/	/	/	/	/	/	/	/	/	((((.....))))..........	.....((((.........)))).	((((......))))........	....(((((.........)))))	.((.((...........)).)).	((((....))))..........	(((......)))...........	.(((.....)))...........	((((.....))))...(....)	(((.......))).........
2GIO	/	/	/	/	/	/	/	/	/	/	(((((...(.(((....))).)..)))))	(((((.....(((....)))....)))))	((((((((((.(......).))))..)))))	(((((..((.(((....)))))..)))))	((((((((((.(.....).)))))..))))	(((((..(..(((....))).)..)))))	((((....(.(((....))).)...))))	((((.((((.(.....).)))))..))))	(((((.(((.(.....).)))...)))))	((((((((((.((....))))))..)))))
2GIP	/	/	/	/	/	/	/	/	/	/	(((((..(((((....)))))..)))))	((((((((((((....))))))..)))))	((((((((((((....)))))))..))))	.((((..(((((....)))))..)))).	(((((...((((.....))))...)))))	((((((((((......)))))..)))))	((((.((((((....))))))...))))	(((((((((((......)))))))..))))	((((...(((((....)))))...))))	(((((.(((((....)))))...)))))
2GRW	/	/	/	/	/	/	/	/	/	/	((((.((((....)))).))))	((((..(((....)))..))))	.(((.((((....)))).))).	(((..((((....))))..)))	(((((((((....))))..))))	((((.(((......))).))))	(((.(((((....)))).))))	.(((..(((....)))..))).	((((.((.(.....).)).))))	/
2GV3	/	/	/	/	/	/	/	/	/	/	(((((((........)))))))	(((((((.(.....).)))))))	((((((..........))))))	((((((((.....)..)))))))	.((((((........)))))).	(((((((.(......))))))))	((((((.(.....)...))))))	((((((..(.....)..))))))	(((((((.(....)..)))))))	.((((((.(.....).)))))).
2GV4	/	/	/	/	/	/	/	/	/	/	(((((.(((....))).)))))	.((((.(((....))).)))).	(((((..((....))..)))))	((((..(((....)))..))))	(((((.((......)).)))))	(((((.(.(....).).)))))	/	/	/	/
2GVO	/	/	/	/	/	/	/	/	/	/	((((((......))))))	.(((((......))))).	..((((......))))..	(((.((......)).)))	(((((........)))))	((((((.....).)))))	((((.(......).))))	(.((((......)))).)	/	/
2HEM	/	/	/	/	/	/	/	/	/	/	(((..(((((....)))))..)))	.((..(((((....)))))..)).	((...(((((....)))))..)).	(((...((((....))))...)))	(((..((((......))))..)))	(((..(.(((....))).)..)))	((...(((((....)))))...))	/	/	/
2HGH	/	/	/	/	/	/	/	/	/	/	((((....((....))(((((((...((((.....))))......))))))))))	((((....((....))((((((((.....((........))....))))))))))))	((((....((....))(((((((.....((.(.....).))....))))))))))	((((............((((((((...((((......))))......)))))))))))	((((....((....)).((((((...((((.....))))......))))).))))	((......))....(.((((((((...((((.....))))......))))))).)..	((((....((....))(((((((.(...((.........))...)))))))))))))	((((....((....))(((((((....(((.....)))......)))))))))))	((((....((....))((((((.((...((.........))...)))))))))))))	((((....((....))((((((....((((.....))))......))))))))))

PDB	DNA model RNAfold MFE	subopt #2	subopt #3	subopt #4	subopt #5	subopt #6	subopt #7	subopt #8	subopt #9	subopt #10
2HNS	/	/	/	/	/	/	/	/	/	/
2HUA	/	/	/	/	/	/	/	/	/	/
2HW8	/	/	/	/	/	/	/	/	/	/
2IHX	/	/	/	/	/	/	/	/	/	/
2IXY	/	/	/	/	/	/	/	/	/	/
2IXZ	/	/	/	/	/	/	/	/	/	/
2JPP	/	/	/	/	/	/	/	/	/	/
2JR4	/	/	/	/	/	/	/	/	/	/
2JSE	/	/	/	/	/	/	/	/	/	/
2JTP	/	/	/	/	/	/	/	/	/	/
2JWV	/	/	/	/	/	/	/	/	/	/
2JXV	/	/	/	/	/	/	/	/	/	/
2JYM	/	/	/	/	/	/	/	/	/	/
2K4C	/	/	/	/	/	/	/	/	/	/
2K5Z	/	/	/	/	/	/	/	/	/	/
2K66	/	/	/	/	/	/	/	/	/	/
2K95	/	/	/	/	/	/	/	/	/	/
2KD8	/	/	/	/	/	/	/	/	/	/
2KE6	/	/	/	/	/	/	/	/	/	/
2KHY	/	/	/	/	/	/	/	/	/	/
2KMJ	/	/	/	/	/	/	/	/	/	/
2KOC	/	/	/	/	/	/	/	/	/	/
2KPC	/	/	/	/	/	/	/	/	/	/
2KPD	/	/	/	/	/	/	/	/	/	/
2KPV	/	/	/	/	/	/	/	/	/	/
2KRL	/	/	/	/	/	/	/	/	/	/
2KRP	/	/	/	/	/	/	/	/	/	/
2KUR	/	/	/	/	/	/	/	/	/	/
2KUU	/	/	/	/	/	/	/	/	/	/
2KUV	/	/	/	/	/	/	/	/	/	/
2KUW	/	/	/	/	/	/	/	/	/	/
2KVN	/	/	/	/	/	/	/	/	/	/
2KZL	/	/	/	/	/	/	/	/	/	/

PDB	RNA model RNAfold MFE	subopt #2	subopt #3	subopt #4	subopt #5	subopt #6	subopt #7	subopt #8	subopt #9	subopt #10
2HNS	((((((((....))))))))	.(((((((....))))))).	((((((((......))))))))	((((.((((....)))).))))	(((((((.(....).)))))))	((((((.((....)).))))))	(((((((........)))))))	/	/	/
2HUA	(((((((.((.....((....))....))....)))))))	(((((((.((...((((....)).)).))....)))))))	((((((...(..((((....)).))..)....)))))))	(((((((.((...(.((....)).).))....)))))))	((((((......((((....))).))......)))))))	(((((((.((.....((...))...))....)))))))	(((((((.(.(..((((....)).)).)...))))))))	(((((((.((....(((....)).)..))....)))))))	(((((((.....(((((....)).)).).....)))))))	(((((((........((....)).........)))))))
2HW8	(((((((((..(((....))).....))))))))))	((((((((((.(((....))).)....))))))))))	(((((((((...((.......))....)))))))))))	.((((((((..(((....))).....)))))))))).	(((((((((.....((((....)))).)))))))))))	.(((((((((.(((....))).)....)))))))).	(((((((((......(((....))).)))))))))	(((((((((..((.......)).....)))))))))))	((((((((...(((....))).....))))))))))	((((((((((..((....))..)....)))))))))))
2IHX	(((((((...((((.(((((.......)))))))))....(((......))))(((....)))...)))))))))	((((((((.((((.(((.((.....)).)))))))......((((.....))))(((....)))...))))))))	(((((((...((((.(((((.......)))))))))....((((......))))((....))....)))))))	(((((((...((((.(((((.......))))))))).....((((.....)))).((....))....))))))))	((((((((((.(((.(((((.......))))))))))..((((......))))(((....)))...))))))	((((((((((.((((.(((((.......))))))))))..((((.....))))(((....)))...)))))))	.(((((((...((((.(((((......))))))))....((((.....))))(((....)))...))))))).	((((((((((.(((.(((((.......))))))))))).((((......))))(((....)))....))))))))	((((((((((.((((.(((((.......))))))))))).((((.....))))(((....)))....)))))))	((((((((.((((((.(((((.......))))))))..))((((.....))))(((....)))...))))))))
2IXY	((((((((((......))))))).))))	((((((((((((...)).)))))).)))	((((((((((((...).)))))).))))	((((.(((((......)))))..))))	.((((((((((......)))))).))).	((((((((((........))))).))))	((((.((((((...)).))))..))))	((((.(((((...).)))))..)))))	((((((((.((...))..)))).))))	.((((((((((...)).))))).)).
2IXZ	....	((...)).	(.....).	.(...)..	(......)	/	/	/	/	/
2JPP	((((((((....))))))))	((((((......)))))))	.(((((((....))))))).	.((((((......)))))).	((((.(((....))).))))	((((((........))))))	(((((.((....)).)))))	((((.((......)).))))	(((((.(.......).)))))	/
2JR4	(((((.......)))))	.((((.......)))).	(((((.(...).)))))	(((((.(....))))))	((((.((....))))))	(((.(((....))))))	((((.(......)))))	(((.((......)))))	((((.........))))	.((((.(...).)))).
2JSE	((((..(((....)))..))))	.(((..(((....)))..))).	((((((.((....)))).))))	(((((.(((....)))).))))	((((.((((....))).)))))	(((...(((....)))...)))	((((...((.....))...))))	((((((.(.....).)).))))	((((.(.((....)))..))))	((((..((......))..))))
2JTP	(((((((((((...((...))..)))))))))))	((((((((((((............)))))))))))	((((((((((....((...))...))))))))))	.((((((((((...((...))..))))))))).	((((((((((((...(...)....)))))))))))	((((((((((((...(......)..))))))))))	((((((((((((.............))))))))))	((((((((((((........)..))))))))))	(((((((((.....((...))....)))))))))	((((((((((((....(...)...))))))))))
2JWV	(((((....((((....))))...)))))	(((((.(..((((....)))).).)))))	(((((.(.((.((....)))).).)))))	(((((.(.(.(((....)))).).)))))	(((((....(((......))).....)))))	(((((.(.(..((...))..).).)))))	(((((.....(((....)))....)))))	.((((....(((....)))...)))).	(((((..(.((((....)))..))))))	(((((.(.((.((...)).)).).)))))
2JXV	((((((((..((((....))))...))))))))	((((((((.(((((....)))).).))))))))	.(((((((..((((....))))...))))))).	((((((((.(((((....))))..))))))))	(((((((((.((((....)))))..))))))))	.(((((((.(((((....)))).).))))))).	((((((((...(((....)))....))))))))	((((((((...((((....))))....)))))))	((((((((..(((((....)))).)..)))))))	((((((((((.((((....))))))..)))))))
2JYM	(((((.(((.....))))))))	.((((.(((.....))))))).	(((((.((.......)))))))	(((((............)))))	..(((.(((.....))))))..	(((((..((.....)).)))))	.((((.((........)))))).	((((..(((.....))).))))	(((((.(.........))))))	(((((((.....))...)))))
2K4C	(((((((.(((...)))(((((.....)))))........((.((((((((....)))))))).))))))))))....	(((((((.(((...)))(((((.....)))))........((.(((((((......))))))).))))))))))....	((((((..(((...)))((((((.....)))))........((.(((((((((....)))))))).)).)))))))....	((((((..(((...)))(((((.....)))))........((.((((((((......)))))))).)).))))))....	(((((((...........(((((.....)))))........((.((((((((.....))))))).)))))))))))....	(((((((...........(((((.....)))))........((.(((((((......)))))).)))))))))).....	(((((((..((...)).(((((.....)))))........((.((((((((....)))))))).))))))))))....	(((((((..((...)).(((((.....)))))........((.(((((((......))))))).)))))))))))....	(((((((...((.....(((((.....)))))....))((.((((((((....))))))).))))))))))....	(((((((...((.....(((((.....)))))....))((.((((((((.......)))))).))))))))))....
2K5Z	(((((((((.((....)).))))))).))))	((((.((((.((....)).)))))..))))	(((((((((..((....))..)))).))))	.((((((((.((....)).))))).)).	(((((((((..(....)..)))).))))	((((.((((.((....))...))))))))	((((((((((.((....)).))))))).))))	((((((((.(((....).)))))).))))	(((((((((..........))))).))))	((((.(((..((....))..)))...))))
2K66	(((((((((....)))))))))	((((((((......))))))))	.((((((((....)))))))).	(((((.(((....))).)))))	((((((.((....)).))))))	.(((((((......))))))).	((((.((((....)))).))))	((.((((((....)))))).))	/	/
2K95	((((((....((.....))....)))))).................	((((((....((.....))....))))))...(((.....))).....	((((((....((.....))....))))))(..(((.....)))..)...	((((((.....(.....).....)))))).................	((((((.................)))))).................	((((((....((.....))....))))))...((.......)).....	((((((....((.....))....))))))....((.....))......	((((((....((.....))....))))))...........((....))	(((........)))((((((.....................)))))).	((((((.....(.....).....))))))...(((.....)))....
2KD8	(((((((((....)))))))))	((((((((......))))))))	(((((((........)))))))	.((((((((....)))))))).	.(((((((......))))))).	(((((((.(....).)))))))	.((((((........)))))).	((((((.((....)).))))))	((((((..........))))))	(((.(((((....))))).)))
2KE6	((((((((((((((((((((........))))))).))))).)))))))	((((((((((((((((((((........))))))).))))).))))))))	(((((((((((((((((((........))))))).))).)))))))))	(((((((((((((((((((..........)))))).))))).)))))))	((((((((((((((((((((..........)))))).))))).))))))))	(((((((((((((((((((..........)))))).))).)))))))))	((((((((((((.((((((.........)))))..))))).)))))))	((((((((((((.((((((........))))))..))))).))))))))	((((((((((((.((((((........))))))..))).)))))))))	(((((((.(((((((((((........))))))).))))..)))))))
2KHY	(((((.......((((....)))).......))))))	.(((((.......((((....)))).......))))).	((((((...(...((((....))))...)...))))))	((((((......(.((((....)))).)......))))))	((((((....((.((((....))))..))...))))))	((((((....(((((((....))))...))).))))))	..((((.......((((....)))).......))))..	.(((((...(...((((....))))...)...))))).	.(((((.....(.((((....)))).)......))))).	(((((.........((((....)))).........)))))
2KMJ	(((((((..((((....))))))))))))	.((((((..((((....))))))))))).	((((((...((((....)))))..))))))	.(((((...((((....)))).))))).	((((((...(((....))).))))))	..((((..((((....)))))))))..	((((((..((((......)))))))))))	.((((((...(((....))).)))))).	..((((...((((....)))).))))..	.((((((..((((....)))))))))).)
2KOC	(((((....)))))	.((((....)))).	((((......))))	(((........)))	(((.(....).)))	/	/	/	/	/
2KPC	((((((.....))))))	.(((((.....))))).	(((((.......)))))	.((((.......)))).	(((((....).)))))	.((((....).)))).	..((((.....))))..	..(((.......)))..	((.(((.....))).))	/
2KPD	(((((.......)))))	.((((.......)))).	..(((.......)))..	((((.........))))	.(((.........))).	((.((......)).))	/	/	/	/
2KPV	((((((((..((((....))))...))).))))	((((((((.(((((....)))).).)))).))))	.(((((((..((((....))))...)))).))).	(((((((...((((....))))....))).))))	((((((((.(((((....))))).)))).))))	(((.((((..((((....))))...))))..)))	((((.(((..((((....))))...)))..))))	.((((((((.(((((....))))).).)))).))).	((((((((...(((....)))....)))).))))	((((((((..(((......)))...)))).))))
2KRL	((((((((....))))))))......(((((((((.........)))))))))...((((.(((((......(((....)))......))))).))))....	(((.....(((((........)))))(((((((((.........)))))))))...((((.(((((......(((....)))......))))).))))))).	((((((((....)))))))))......((((((((((.........))))))))))...(((..(((((......(((....)))......))))))..)))....	((((((((....))))))))......(((((((((.........)))))))))...((((..((((......(((....)))......))))..))))....	.(((((((....)))))))......((((((((.........)))))))))...((((.(((((......(((....)))......))))).))))....	...(((..(((((........)))))(((((((((.........)))))))))...((((.(((((......(((....)))......))))).)))).)))	((((((((....))))))))......((((((((((..(....).))))))))))...((((.(((((......(((....)))......))))).))))....	(((.....(((((........))))(((((((((.........)))))))))...(((..(((((......(((....)))......)))))..)))))).	((......(((((........))))(((((((((.........)))))))))...((((.(((((......(((....)))......))))).)))).)).	(((.....(((((........)))))(((((((((.........)))))))))...((((..((((......(((....)))......))))..)))))))).
2KRP	(((((.......)))))	.((((.......)))).	(((((.(....))))))	(((((.(...).)))))	..(((.......)))..	.(((....)))......	(((((...)..)))))	((((.........))))	.((((.(....))))).	.((((.(...).)))).
2KUR	((((((((((((((((((((........))))))).))))).)))))))	((((((((((((((((((((........))))))).))))).))))))))	(((((((((((((((((((........))))))).))).)))))))))	(((((((((((((((((((..........)))))).))))).)))))))	((((((((((((((((((((..........)))))).))))).))))))))	(((((((((((((((((((..........)))))).))).)))))))))	((((((((((((.((((((.........)))))..))))).)))))))	((((((((((((.((((((........))))))..))))).))))))))	((((((((((((.((((((........))))))..))).)))))))))	(((((((.(((((((((((........))))))).))))..)))))))
2KUU	((((((((((((((((((((........))))))).))))).)))))))	((((((((((((((((((((........))))))).))))).))))))))	(((((((((((((((((((........))))))).))).)))))))))	(((((((((((((((((((..........)))))).))))).)))))))	((((((((((((((((((((..........)))))).))))).))))))))	(((((((((((((((((((..........)))))).))).)))))))))	(((((((.(((((((((((........))))))).))))..)))))))	((((((((.(((((((((((........)))))))).)))..))))))))	((((((((((((.((((((........))))))..))).)))))))))	((((((((((((.((((((........))))))..))).))))))))
2KUV	((((((((((((((((((((........))))))).))))).)))))))	((((((((((((((((((((........))))))).))))).))))))))	(((((((((((((((((((........))))))).))).)))))))))	(((((((((((((((((((..........)))))).))))).)))))))	((((((((((((((((((((..........)))))).))))).))))))))	(((((((((((((((((((..........)))))).))).)))))))))	((((((((((((.((((((.........)))))..))))).)))))))	((((((((((((.((((((........))))))..))))).))))))))	((((((((((((.((((((........))))))..))).)))))))))	(((((((.(((((((((((........))))))).))))..)))))))
2KUW	((((((((((((((((((((........))))))).)))).))))))))	((((((((((((((((((((........))))))).))).)))))))))	(((((((((((((((((((.........))))))).))))).))))))))	(((((((((((((((((((..........)))))).))).))))))))	((((((((((((.((((((........))))))..)))).))))))))	((((((((((((.((((((........)))))))..))).)))))))))	(((((((.(((((((((((........)))))))).))))..)))))))	((((((((((((.((((((........)))))))..))).))))))))	((((((((((((.((((((........)))))))..)).)))))))))	.((((((((((((((((((........))))))).))))).))))))).
2KVN	(((((......))))).	((((........)))).	.((((......))))..	((((((...)).)))).	((((((...).))))).	((.((......)).)).	/	/	/	/
2KZL	((((((......((((.......((((....)))).......))))...))))))	((((((..(...((((.......((((....))))........)))))..))))))	((((((....((.(((.......((((....)))).......)))))..))))))	((((((....(.((((.......((((....)))).......)))))..))))))	((((((......((((...(...((((....))))...)...))))...))))))	((((((...(..((((.......((((....))))........)))))..))))))	((((((......((((.....(.((((....)))).)......))))...))))))	(((((.......((((.......((((....))))........)))).....)))))	((((((......((((....((.((((....))))..))...))))...))))))	((((((......((((.....(((((((....))))...))).))))...))))))

	DNA model											RNA model									
PDB	RNAfold MFE	subopt #2	subopt #3	subopt #4	subopt #5	subopt #6	subopt #7	subopt #8	subopt #9	subopt #10		RNAfold MFE	subopt #2	subopt #3	subopt #4	subopt #5	subopt #6	subopt #7	subopt #8	subopt #9	subopt #10
2L2J	/	/	/	/	/	/	/	/	/	/		((((.(((((((((.((((....))))).))))))))).))))	((((.(((((((((..(((....)))..))))))))).))))	(((..(((((((((.((((....)))).)))))))))..)))	.(((.(((((((((.((((....)))).))))))))).)))).	(((..((((((((..(((....)))..)))))))))..)))	((((.((((((((..((((....))))..))))))))).))))	.(((.(((((((((..(((....)))..))))))))).))).	((((..((((((((.((((....)))).))))))))..))))	((((.((((((((((.(((....))))))).)))))))).))))	((((.(((((((((((.(((....)))))).)))))))).))))
2L3C	/	/	/	/	/	/	/	/	/	/		((((.((((((((((....)))))))))).))))	((((.(((((((((......)))))))))).))))	((((.((((((((........))))))))).))))	.(((.((((((((((....)))))))))).))).	.(((.((((((((......)))))))).))).	((((..(((((((((....)))))))))..))))	(((..((((((((((....))))))))))..)))	((((..((((((((......)))))))..))))	(((..(((((((((......)))))))))..)))	((((.(((((((..........))))))).))))
2L3E	/	/	/	/	/	/	/	/	/	/		((((((.....((((((....)))))))))))))	((((((......((((((....))))).))))))	.((((((.....((((((....)))))))))))).	(((((((....((.((((....)))))))))))))	(((((((....(.(((((....)))))))))))))	((((((...(((..((((....))))))))))))))	(((((.......((((((....))))))..)))))	(((((.(.....((((((....)))))).)))))	((((.((.....((((((....))))))).))))	((((((((.(.....((((((....)))))))))))))
2L3J	/	/	/	/	/	/	/	/	/	/		(((((((.(((((((((.(((.(((((((((((.....))))))))))))).))).))))))))).)))))))	(((((((.(((((((((..((.(((((((((((.....)))))))))))).))..))))))))).)))))))	((((((..(((((((((.(((.((((((((((((.....)))))))))))).))).)))))))))).))))))	.((((((.(((((((((.(((.(((((((((((.....))))))))))))).))).))))))))).))))))).	(((((((.(((((((((.(((.(((((((((((.......)))))))))))).))).)))))))))).)))))))	(((((((.(((((((((.(((..((((((((((.....))))))))))..))).))))))))).))))))))	(((((((.(((((((((.((..(((((((((((.....))))))))))))..)).)))))))).)))))))	((((((..(((((((((..((.((((((((((((.....)))))))))))).))..)))))))))..))))))	(((((((.((((((((..(((.((((((((((((.....)))))))))))).)))..)))))))).)))))))	(((((((.(((((((((.(((.((((((((((((....).))))))))))).))).))))))))).)))))))
2L5Z	/	/	/	/	/	/	/	/	/	/		((((((...(((....))).))))))	((((((..((......))..))))))	(((((....(((....)))..)))))	.(((((...(((....))).))))).	((((((((.........)).))))))	((((((((.(......))).))))))	.(((((..((......))..))))).	(((((((.((......))).))))))	(((((...((((....))).))))))	..((((...(((....))).))))..
2L6I	/	/	/	/	/	/	/	/	/	/		(((((.....))))).	((((.......)))).	.((((.....))))..	....	((.((.....)).)).	(.(((.....))).).	(((.........))).	/	/	/
2LAC	/	/	/	/	/	/	/	/	/	/		(((((.......)))))	(((((.(....)))))	((((.........))))	(((((.(...).)))))	.((((.......)))).	((((.........)))).	.((((.(....))))).	((((..(....).))))	.(((.........))).	(((..........))).
2LBJ	/	/	/	/	/	/	/	/	/	/		((((((...)))))))	((((((.....))))))	(((((.......)))))	.((((((...)))))).	.(((((.....))))).	(((((.(....))))))	(((((.(...).)))))	((((((....).)))))	.((((((...)))).))	.((((.......)))).
2LBK	/	/	/	/	/	/	/	/	/	/		((((((.....))))))	.(((((.....))))).	((((...))))......	..((((.....))))..	(((.....)))......	(((((........)))))	(.((((.....)))).)	.(((((.....)))).)	/	/
2LBL	/	/	/	/	/	/	/	/	/	/		(((((.......)))))	.((((.......)))).	(((....)))).......	.(((...))).......	(((((.(...).)))))	(((((.(....))))))	((((.((....))))))	((......)).......	..(((.......)))..	.((.....)).......
2LBS	/	/	/	/	/	/	/	/	/	/		((((((((((((((....))))))))))))))	.(((((((((((((....))))))))))))).	..(((((((((((((....)))))))))))..	((((((((((((((......)))))))))))))	(((((((((.((((....))))).)))))))))	(.(((((((((((((....))))))))))).)	.(((((((((((((((....))))))))))).)	(((((((((((.((....)).))))))))))))	((((.((((((((((....)))))))).))))	((((((((((((((.(....).)))))))))))
2LC8	/	/	/	/	/	/	/	/	/	/		.(((.((((((((........))))).)))..)))..((((((......)))))...	.(((.((((((((........)))).))))..)))..((((((......)))))....	.....((((.....))))((((((((....(((.....))))......)))))))...	.(((.((((((((.(....).))))).)))..)))..((((((......)))))...	.(((.((((((((.(....).)))).))))..)))..(((((((......)))))...	.(((.((((((((........))))).)))..)))..((((((.......)))))...	.(((.((((((((........)))).))))..)))..((((((.......)))))..	.(((.((((((((.(.....))))).)))..)))..(((((......)))))...	.(((.((((((((.(.....))))).))))..)))..(((((.......))))))...	((((((((...((.....)).))))))).))........((((((......)))))...
2LDL	/	/	/	/	/	/	/	/	/	/		((((((.((((.....)))).))))))	((((((.(((........))).))))))	((((((..(((.....)))..))))))	.((((((.((((.....)))).)))))).	((((((..((........))..))))))	.((((((.(((........))).))))).	((((((.(((.(....)))).))))))	((((((...............))))))	((((((..((((.....))))..)))))	((((((...((.....))...))))))
2LDT	/	/	/	/	/	/	/	/	/	/		(((((((..((((....))))...)))))))	(((((((...(((....)))....)))))))	((((((...((((....)))))....))))))	.((((((..((((....))))...)))))).	(((((((....(((....)))....)))))))	(((((((..((((....))))..).))))))	(((((((....((....)).....)))))))	(((((((...(((....)).)....)))))))	.((((((...(((....)))....)))))).	(((((((...(((....)))...).))))))
2LHP	/	/	/	/	/	/	/	/	/	/		(((((((((.((((((....))))))..)))))))))	(((((((..((((((....))))))...))))))))	.((((((((.((((((....))))))..))))))))).	(((((((...((((((....))))))....)))))))	(((((((((.((((((....)))))).).))))))))	(((((((((..(((((....))))))...)))))))))	((((((.((.((((((....))))))).)).))))))	(((((((((.(((((......)))))..)))))))))	(((((((.(.((((((....))))))..).)))))))	((((((....((((((....))))))......))))))
2LI4	/	/	/	/	/	/	/	/	/	/		(((((((((((((....))))))))))))))	(((((((((((((......)))))))))))))	.(((((((((((((....))))))))))))).	((((((((((((........))))))))))))	.((((((((((((......)))))))))))).	((((((((.((((((....)))))).))))))))	(((((((((.((((....)))).)))))))))	(((((((.((((((....)))))).)))))))	((((((((((((.(....).))))))))))))	(((((((.((((((....))))))).))))))
2LJJ	/	/	/	/	/	/	/	/	/	/		(((((..(((((.....)))))))))))	((((...(((((.....))))).))))	.((((..(((((.....))))))))).	(((((..((((.......)))))))))	(((((...((((.....)))).)))))	.(((...(((((.....))))).))).	(((((.(.((((.....))))))))))	..(((..(((((.....)))))))))..	((((...((((.......)))).))))	.((((..((((.......)))))))))).
2LK3	/	/	/	/	/	/	/	/	/	/		((((..(((((....)))))))))	(((...(((((....))))).)))	((((..((((......))))))))	(((.(.(((((....)))))))))	.(((..(((((....)))))))).	(((...((((......)))).)))	((((...((((....)))).))))	.((...(((((....))))).)).	(((.(.((((......))))))))	.(((..((((......))))))).
2LKR	/	/	/	/	/	/	/	/	/	/		((((((((.(..((((((((.((((.(((.....)))..)))).)))....((((((((((....)))))))))))(((....)))...)))))..).).)))).))))	((((((((.(..(((((.((.((((.(((.....)))..)))).))......(((((((((((....))))))))))))(((....)))...)))))....).).)))).))))	((((((((.(..((((((((.((((.(((.....)))..)))).)))...((((((((((((....)))))))))))))((....))....)))))..).).)))).))))	((((((((.(..((((((((.((((.(((.....)))..)))).))).....((((((((((....)))))))))).(((....)))...)))))..).).)))).))))	((((((((.(..(((((.((.((((.(((.....)))..)))).)))....(((((((((((....))))))))))))((....))....))))))..).).)))).))))	.(((((((.(..((((((((.((((.(((.....)))..)))).)))....((((((((((....))))))))))(((....)))...))))))..).).)))).))).	((((((((.(..(((((.((((.....))))...((((((...))))))..((((((((((....))))))))))))(((....)))...)))))..).).)))).))))	((((((((.(..(((((.((.((((.(((.....)))..)))).)).......(((((((((....)))))))))).(((....)))...)))))..).).)))).))))	((((((((..(..((((((((.(((.(((.....)))..)))).))).)...(((((((((((....))))))))))))(((....)))...))))))...)...)))).))))	((((((((.(..(((((.((((.....))))....((.....))......)..(((((((((((....))))))))))))(((....)))...))))))..).).)))).))))
2LP9	/	/	/	/	/	/	/	/	/	/		(((((......)))))	((((........))))	((((((...).)))))	.((((.......)))).	....	..(((.......)))..	.(((.........))).	((.........))...	((((.......)))).	((.((......)).))
2LPA	/	/	/	/	/	/	/	/	/	/		((((((...))))))	.(((((...))))).	(((((.....)))))	..((((...))))..	((((.......))))	((.(((...))).))	...(((...)))...	(.((((...)))).)	....	.((((.....)))).
2LQZ	/	/	/	/	/	/	/	/	/	/		((((((...((((....)))))))))))	((((((....(((....))).))))))	(((((....((((....))))).)))))	.((((((...((((....))))))))))).	((((((((.........))...))))))	(((((.....(((....)))..)))))	((((((.....((....))..))))))	((((.....((((....))))..))))	((((((...(((.......))))))))	((((((...(.((....)).))))))
2LU0	/	/	/	/	/	/	/	/	/	/		(((((..((((.((....))...((((((....)))))))))...)))))	(((((..((((............((((((....)))))))))...)))))	(((((.(((((.((....))...(((((....)))))))))).).)))))	(((((.(((((............((((((....)))))))))).).)))))	(((((..((((.((....))....((((....)))).))))...)))))	((((...((((.((....))...(((((....)))))))))....))))	((((...((((............(((((....))))))))....))))	(((((..((((..(....)....(((((....)))))))))...)))))	(((((.(((((.((....))....((((....)))).)))).).)))))	(((((..((((..((((.((......)))))).....))))...)))))
2LUB	/	/	/	/	/	/	/	/	/	/		(((((((((.((((((....))))))..)))))))))	.((((((((.((((((....))))))..)))))))).	(((((((((..(((((....)))))...)))))))))	((((((.((.((((((....))))))..)).))))))	(((((((((.(((((......)))))..)))))))))	((((((((..((((((....))))))...))))))))	(((((((.(.((((((....))))))).).)))))))	(((((.(((.((((((....)))))..))).)))))	(((((((...((((((....))))))....)))))))	(((.(((((.((((((....))))))..))))).)))
2LUN	/	/	/	/	/	/	/	/	/	/		((((.(..((((....))))..).))))	((((....((((....))))....))))	.(((.(..((((....))))...).))).	((((..((((((....)))..)))))))	((((.(((.(((....)))))).))))	((((.((.((((....))))))...))))	((((.(...(((....)))...).))))	((((..((((((....))..))))))))	((((.....(((....)))....))))	((((..((((((....))))..))))))
2LUP	/	/	/	/	/	/	/	/	/	/		((((((((((((((....))))))))))))))	.(((((((((((((....))))))))))))).	..(((((((((((((....)))))))))))..	((((((((((((((......)))))))))))))	(((((((((.((((....))))).)))))))))	(.(((((((((((((....))))))))))).)	.(((((((((((((((....))))))))))).)	(((((((((((.((....)).))))))))))))	((((.((((((((((....)))))))).))))	((((((((((((((.(....).)))))))))))
2LV0	/	/	/	/	/	/	/	/	/	/		((((((((........))))))))	.(((((((........))))))).	((((((((.(....).))))))))	(((((((..........)))))))	((((((..(((....)))))))))))	((((((((.((...))))))))))))	((((((((..(....)))))))))	((((((.(........).))))))	(((((((..((....)))))))))	(((((.((........)).)))))
2M12	/	/	/	/	/	/	/	/	/	/		((((((...........))))))	((((((.(((....)))))))))	((((((.((......))))))))	((((((.(........).))))))	(((((((((...))..)))))))	((((((..(....)...))))))	(((((..(((....))).)))))	((((((((.....))..))))))	(((((..((......)).)))))	(((((.............)))))
2M21	/	/	/	/	/	/	/	/	/	/		(((((((.......)))))))	((((((.........))))))	(((((((.(...).)))))))	.((((((.......)))))).	(((((((.(....)))))))	(((((...........)))))	(((((((.......).))))))	.(((((.........))))).	((((((..(...)..))))))	(((((.(........).)))))
2M22	/	/	/	/	/	/	/	/	/	/		(((((.((((...)))).)))))	(((((.(((.....))).)))))	(((((.((.......)).)))))	.((((.((((...)))).)))).	(((((..(((...)))..)))))	.((((.(((.....))).)))).	(((((..((......))..)))))	((((..((((...))))..))))	((((..(((.....)))..))))	(((((.............)))))
2M24	/	/	/	/	/	/	/	/	/	/		(((((((((...........)))))))))	(((((((((.((.......)))))))))))	(((((((((.(........).)))))))))	(((((((((.(((....))))))))))))	(((((((((...(....)...)))))))))	((((((((..((......)).))))))))	((((((((.............))))))))	(((((((((..(.......))))))))))	(((((((((.(.(...).).)))))))))	.((((((((...........)))))))).
2M4W	/	/	/	/	/	/	/	/	/	/		((((((....))).)))	((.(((....)))..))	(((.((....))..)))	...(((....)))....	.(((((....))).)).	((.(((....))).)).	(..(((....)))..).	....	(((...........)))	.(.(((....)))..).
2M57	/	/	/	/	/	/	/	/	/	/		(((.((((((((((.....))))))..))))).)))	(((.((((((((((.....))))))..))))))).)))	.((.((((((((((.....)))))..))))).)).	.((.((((((((((.....))))..))))))).)).	((..((((((((((.....)))))..)))))..))	(((.((((.(((((.....)))))...)))).)))	((..((((((((((.....))))..))))))..))	(((.(((((.((((.....))))...))))).)))	....((((((((((.....))))))..)))))....	.(..((((((((((.....))))).))))).)..).
2M5U	/	/	/	/	/	/	/	/	/	/		(((((((((....)))))))))	.((((((((....)))))))).	((((((.((....)).))))))	((((((((......))))))))	(((((((.(....).)))))))	(((((.(((....))).)))))	(((.(((((....))))).)))	/	/	/
2M8K	/	/	/	/	/	/	/	/	/	/		..........(((((((((((((((....)))).).)))))))))))	..........(((((((((((((((......))).).)))))))))))	...........((((((((((((((....)))).).))))))))))).	..........((((((((((.((((....))))...))))))))))	...........((((((((((((((......))).).))))))))))).	..........((((((((((((((........)).).))))))))))	............((((((((((((((....)))).).)))))))))..	..........((((((((((.(((......)))...)))))))))))	...........(((((((((.((((....))))...)))))))))).	............(((((((((((((......))).).))))))))))..
2MEQ	/	/	/	/	/	/	/	/	/	/		((((((.......))))))	.(((((.......))))).	(((((.........)))))	((((((....)).)))))	((((((....).))))))	((((((.(...).))))))	..((((.......))))..	.((((.........)))).	((((((......).)))))	.((((((....)).)))).

PDB	DNA model: RNAfold MFE	subopt #2	subopt #3	subopt #4	subopt #5	subopt #6	subopt #7	subopt #8	subopt #9	subopt #10
2MF0	/	/	/	/	/	/	/	/	/	/
2MFC	/	/	/	/	/	/	/	/	/	/
2MFD	/	/	/	/	/	/	/	/	/	/
2MFE	/	/	/	/	/	/	/	/	/	/
2MFF	/	/	/	/	/	/	/	/	/	/
2MFG	/	/	/	/	/	/	/	/	/	/
2MHI	/	/	/	/	/	/	/	/	/	/
2MNC	/	/	/	/	/	/	/	/	/	/
2MQT	/	/	/	/	/	/	/	/	/	/
2MS0	/	/	/	/	/	/	/	/	/	/
2MTJ	/	/	/	/	/	/	/	/	/	/
2MXJ	/	/	/	/	/	/	/	/	/	/
2MXL	/	/	/	/	/	/	/	/	/	/
2N0R	/	/	/	/	/	/	/	/	/	/
2N1Q	/	/	/	/	/	/	/	/	/	/
2N2O	/	/	/	/	/	/	/	/	/	/
2N2P	/	/	/	/	/	/	/	/	/	/
2N3O	/	/	/	/	/	/	/	/	/	/
2N3Q	/	/	/	/	/	/	/	/	/	/
2N4L	/	/	/	/	/	/	/	/	/	/
2N6S	/	/	/	/	/	/	/	/	/	/
2N6T	/	/	/	/	/	/	/	/	/	/
2N6W	/	/	/	/	/	/	/	/	/	/
2N6X	/	/	/	/	/	/	/	/	/	/
2N7M	/	/	/	/	/	/	/	/	/	/

PDB	RNA model: RNAfold MFE	subopt #2	subopt #3	subopt #4	subopt #5	subopt #6	subopt #7	subopt #8	subopt #9	subopt #10
2MF0	((((........))))..((((((......))))))......(((((.....)))))(((((.....)))))	((((........))))..((((((......))))))......(((((.....))))).((((.....)))).	.(((........)))...(((((......))))))......(((((.....)))))(((((.....)))))	((((........))))..((((((.....))))))......((((.....)))).(((((.....)))))	.(((........)))...((((((......))))))......(((((.....))))).((((.....)))).	((((....))))......((((((......))))))......(((((.....)))))(((((.....)))))	((((........))))(.((((((......))))))..)...(((((.....)))))(((((.....)))))	((((........))))..((((((......))))).)......(((((.....)))))(((((.....)))))	((((........))))...(((((......)))))......(((((.....)))))(((((.....)))))	((((........))))..((((((......))))))......((((.....)))).((((.....)))).
2MFC	(((((((........)))))))	.((((((........)))))).	(((((((.(....).)))))))	(((.(((........))).)))	/	/	/	/	/	/
2MFD	(((((((.....)))))))	.((((((.....)))))).	..(((((.....)))))..	((((.((.....)).))))	.((((((.....))))).)	(((((.(.....).)))))	(.(((((.....))))).)	((((((.......))))))	...((((.....))))...	/
2MFE	((((((((......))))))))	.(((((((......))))))).	(((((((........)))))))	(((((.((......)).)))))	.((((((......))))).))	((.(((((......))))).))	..((((((......))))))..	((((((.(......).))))))	/	/
2MFF	((((((((.....))))))))	.(((((((.....))))))).	(((((((.......)))))))	((((.(((.....))).))))	(((.((((.....)))).)))	/	/	/	/	/
2MFG	((((((((.....))))))))	.(((((((.....))))))).	(((((((.......)))))))	(((((.((.....)).)))))	((((((.(.....).))))))	/	/	/	/	/
2MHI	(((((((.(((((.((((...)))))))))).((((.....))))..)))))))	((((((..(((((.((((...))))))))).((((.....))))...))))))	((((((((((((.((((...))))))))).((((.....)))))).))))))	((((((((((((.((((...))))))))).((((.....))))).)))))))	((((((((.((((((.....)))))).))..((((.....))))...))))))	(((((((.(((((.((((...)))))))))..(((.....)))...)))))))	((((((..(((((.((((...)))))))))..(((.....)))....))))))	(((((((.((((((.(((...)))))))))).((((.....))))...)))))))	((((((..(((((.(((...)))))))).((((.....))))...))))))	(((((((.(((((.((((...))))))))..((((.....))))).)))))))
2MNC	((((.((...))))))	(((.(((...))))))	((((........))))	((((.(.....)))))	(((.((.....)))))	((((.(....).))))	.(((.((...))))).	.((.(((...))))).	.(((........))).	((((.(...)..))))
2MQT	(((((.((((((((...((((((..((((.((((....))))))))..)))))))))))))).)))))	(((((.((((((((...((((((..(((..((((....)))).))).))))))))))))))).)))))	(((((.(((...((((((((((((..((((.((((....))))))))..)))))))))))))).))))))	(((((.((((((((...((((((..((((.(((......)))))))..)))))))))))))).)))))	(((((.((((((((....((((((..((((.((((....))))))))..))))))).)))))))).)))))	(((((.(((((.(((..((((((..((((.((((....))))))))..)))))))))))))))).)))))	(((((.((((((((.....((((..(((.((((....))))))))..))))..)))))))).)))))	((((..((((((((...((((((..((((.((((....))))))))..))))))))))))..))))	.((((.((((((((...((((((..((((.((((....))))))))..))))))))))))))).)))).	(((((.(((.(((((..((((((..((((.((((....))))))))..)))))))))))))).)))))
2MS0	((((((((.(.....(((.....(((((((.......)))))))..)))((((....))))).))))))))	((((((((.......(((.....(((((((.......)))))))..)))(((.......))).))))))))	(((((((..............(((((((((.......))))))).))(((((.......))))))))))))	(((((((...............(((((((((.......)))))).)))(((((......)))))))))))	(((((((..(((.........)))(((((((.......)))))).....(((((.......))))))))))))	((((((((.(.....(((.((..((((((((.......))))))))))((((....))))).))))))))	((((((((.......(((.((..(((((((.......))))))))))))(((.......))).))))))))	(((((((.................((((((((.......)))))))...((((((.......)))))))))))	((((((((.(.....(((.((...(((((((.......)))))).)))))((((....))))).))))))))	((((((((.(.....(((.....(((((((.......)))))))..)))(((......)))).))))))))
2MTJ	(((((..(((((....))))).(((((....)))))......))))).	((((((((((((....))))).(((((....))))).))..))))).	(((((..(((((....)))))..((((....))))......))))).	(((((.((((((....))))).((((((....))))).)...))))).	(((((..(((((....)))))((((((....))))).)...))))).	(((((...((((....)))..(((((....)))))......))))).	(((((..(((((....)))))(((((((....))))..))..))))).	(((((..((((......)))).(((((....)))))......))))).	((((((((((((....)))))..((((....))))..))..))))).	(((((..(((((....))))))...(((....)))........))))).
2MXJ	((......)).	....	((.(...))).	.(.......)..	.(........).	(........).	(.......)..	...(...)...	.(.(...).).	/
2MXL	..(((((((..((((((.......)).))))..)))))))	...((((((..((((((.......)).))))..)))))).	....(((((..((((((......)).))))..)))))...	..(((((((..(((.((.......))..)))..)))))))	.....((((..((((((.......)).))))..))))...	..(((((((..((((((.(...))).))))..)))))))	..((((((...((((((.......)).))))...))))))	...((((((..(((.((.......))..)))..)))))).	.(.((((((..((((((.......)).))))..)))))))	......(((..((((((.......)).))))..)))....
2N0R	....((((....))))).......	(...((((....))))...)...	.(..((((....)))).).....	..(.((((....)))..))....	.((.((((....)))..))...	.(.(((((....)))..).)..	.....(((....)))........	((..((((....)))).).)...	.(..((((....)))....)..	...(((((....)))..)....
2N1Q	(((((((((((.((......(((((((....)))))))(((....)))...((....((((.(((((((....)))))))...))))..))((((((....))).))))......(((..(((((....)))))..))))).))))))))))))).	((((((((((((..........((((((....)))))))......(((((..((....((((.(((((((....))))))...))))..))((((((....))).))))............(((((....)))))...)))))))))))))))).	(((((((((((.((......((((((((....)))))))(((....)))...((....((((.(((((((....)))))..)).)))))..))((((((....))).))))......(((..(((((....)))))..))))).)))))))))))).	((((((((((((......(..(((((((....)))))))..)..(((((...((....((((.(((((((....)))))))...))))..))((((((....))).))))............(((((....)))))...))))))))))))))))).	((((((((((((.........(((((((....)))))))......(((((...((.....((((.(((((((....)))))..)).))))..))(((((((....))).))))...........((((.....)))))...))))))))))))))).	((((((((((((.......(..(((((((....)))))))..)..(((((...((.....((((.(((((((....)))))..)).))))..))((((((((....))).))))............(((((....)))))...)))))))))))))))).	((((((((((((.((.......(((((((....)))))))(((....)))...((....((((.(((((((....)))))))...))))..))((((((((....))).)))))............(((((....)))))......)).)))))))))))).	(((((((((((.((......(((((((....)))))))(((....)))...((.....((((.((((((....))))..))).))))..))(((((((....))).))))......(((..(((((....)))))..))))).))))))))))).	((((((((((((.........(((((((....)))))))......(((((..((.....((((.(((((((....))))..))).))))..))((((((....))).))))............(((((....)))))...))))))))))))))))).	((((((((((((((((.((((.(((((((....)))))))(((....))).)))))).)))....((((((....)))))))..((.....))((((((....))).))))......(((..(((((....)))))..)))...)))))))))))).
2N2O	((.(((((((...)))))))).	...((((((...)))))))...	....((((((...))))))....	((..((((((...)))))).)).	.(.(((((((...)))))))..	.........((((....)))..	.(..((((((...)))))).)..	((.((((((.....)))))))).	..((((((((...))))))).).	..((((((((...)))))).)).
2N2P	((.(((((((...)))))))).	...((((((...)))))))...	....((((((...))))))....	((..((((((...)))))).)).	.(.(((((((...)))))))..	.(..((((((...)))))).)..	((.((((((.....)))))))).	...((((((.....))))))...	....(((((.....)))))....	((..(((((.....))))).)).
2N3O	(((((((((.....)))))))))	.((((((((.....)))))))).	((((((((.......)))))))))	((((((.((.....)).))))))	/	/	/	/	/	/
2N3Q	(((((.(((.......((((((...(((((.....))))).))).)))....)))))))).	(((((.(((..((...((((((...(((((.....))))).))).)))....)))))))))).	(((((.(((........(((.(.(.(((((.....))))).))).))))).....)))))))).	(((((.(((........((.((.((.(((((.....))))).)).)))).....)))))))).	(((((.(((..((...(((.(.(.(((((.....))))).)).))))...)))))))))).	(((((.(((..((...((.((.((.(((((.....))))).)).))))...)))))))))).	(((((.(((..((((((....))).)))(((..((((....)))))))......))))))))).	(((((.(((........(((.(.(..((((((.....)))))..).))))).....)))))))).	(((((.(((........((.((.(..(((((.....)))))..).))))....)))))))).	(((((.(((........((((((.(.(((((.....))))))))).)))......)))))))).
2N4L	(((((((((((((..((..(((((.....))))))..)))))))))))))))	((((((((((((((..((((((......))...))))..))))))))))))))))	(((((((((((((..(((..((((.....))))))..))))))))))))))	((((((((((((((..((((((.....)))...)))..)))))))))))))	((((((((((((((..(((((...........)))))..)))))))))))))	((((((((((((((.....((((((.....))))))...)))))))))))))	((((((((((((((..((((((......)...)))))..)))))))))))))	((((((((((((((..((...((((.....)))).)).))))))))))))	((((((((((((((..((((..(((.....)))))))..))))))))))))))	((((((((((((((..((((.............)))).)))))))))))))))
2N6S	((((((((((((((((...)).)))))))))))))	(((((((((((((((.(.....).))))))))))))))	((((((((((((((((........)))))))))))))))	.((((((((((((((((...)).))))))))))))).	((((((((((((((((.....).))))))))))))))	((((((((((((((.((...))..))))))))))))	((((((((((((((..(.....)..))))))))))))))	.((((((((((((((.(.....).)))))))))))))).	(((((((((((((((..........))))))))))))))	(((((((.((((((((((...)).))))))).))))))
2N6T	((((.((((((((..((((....))))...))))))))))))	(((((.(((((((..((((....)))...))))))))))))	((((((.((((((..((((....))))...))))))))))))	((((.((((((((...((((....))))....)))))))))))	(((((.((((((...((((....)))....)))))))))))	((((((.(((((...((((....))))....)))))))))))	.((..((((((((..((((....))))...))))))))..))	((((.((((((((...(((....)))....)))))))))))	(((((.((((((...(((....))))....)))))))))))	((((((.((((((...(((....))))....)))))))))))
2N6W	((((((((((((((.((.((((((((...((((....))))...)))))))))))).)))))))))))))	((((((((((((((.(((.((((((((..((((....))))...))))))))))).)))))))))))))	((((((((((((((.((((.((((((..((((....))))...))))))))).))))))))))))))))	((((((((((((((.((.((((((((...((((....))))....)))))))))).)))))))))))))	((((((((((((((.(((.((((((...((((....))))....)))))).)))))))))))))	((((((((((((((....((((((((..((((....))))...))))))))...)))))))))))))	((((((((((((((.((((.((((((..((((....))))....)))))))))).)))))))))))))	((((((((((((((....(((((((...((((....))))....))))))...))))))))))))))	((((((((((((((.((.(((((((((...((((....))))....)))))))))).)))))))))))))	(((((((((((((((..(.((((((((..((((....))))...))))))))..)))))))))))))))
2N6X	((((((((.((.((((((((....))))))))).))))))))	((((((((.(((.((((((((....))))))))))).))))))))	((((((((.((((.((((((....)))))))))).))))))))	((((((((....((((((((....))))))))...))))))))	((((((((..(.((((((((....))))))))..))))))))	((((((((..((.((((((((....))))))))..))))))))	((((((((.((..((((((((....))))))).)).))))))))	((((((((..(((.((((((((....))))))))..))))))))	(((((((..((.((((((((....))))))))))..))))))))	((((((((.(((..((((((....))))).))).))))))))
2N7M	(((((((((((.(((((..(((((.(((.....(((....)))..))))))))..((((((((....)))))))))))))).)))))))))))	(((((((((((.(((((..(((((.(((.....(((....)))..))))))))...(((((((....)))))))).)))))).)))))))))))	(((((((((((.(((((..(((((.(((.(.((.(...).)))..))))))))..((((((((....)))))))))))))).)))))))))))	(((((((((((.(((((....(((((((((((((((.((........)).)))))))))))))......))..)))))).)))))))))))	.(((((((((((.(((((..(((((.(((.....(((....)))..)))))))..((((((((....))))))))))))).))))))))))).	(((((((((((.(((((..(((((.(((.(.((.(...).))).))))))))...(((((((....)))))))).))))).)))))))))))	(((((((((((.((((...(((((.((.....(((....)))..)))))))..((((((((....))))))).)))).))))))))))))	(((((((((((.(((((..(((((.(((.....((......))..))))))))..((((((((....))))))))))))).)))))))))))	.((((((((((.(((((..(((((.(((.....(((....)))..))))))))...(((((((....)))))))).))))).))))))))))).	(((((((((((.(((((..(((((.(((...((.(...).))...))))))))..((((((((....))))))))))))).)))))))))))

	DNA model											RNA model									
PDB	RNAfold MFE	subopt #2	subopt #3	subopt #4	subopt #5	subopt #6	subopt #7	subopt #8	subopt #9	subopt #10		RNAfold MFE	subopt #2	subopt #3	subopt #4	subopt #5	subopt #6	subopt #7	subopt #8	subopt #9	subopt #10
2N7X	/	/	/	/	/	/	/	/	/	/		(((((..((.....))..)))))	(((((............)))))	(((((.((......))..))))))	(((((..(((...)))..)))))	(((((....((.....)))))))	(((((((.......)))..)))	(((((..(........)..)))))	(((((....(......).)))))	(((((...(.......)..)))))	(((((((.......)))..))))
2N82	/	/	/	/	/	/	/	/	/	/		(((((..((.....))..)))))	(((((............)))))	(((((.((......))..))))))	(((((..(((...)))..)))))	(((((....((.....)))))))	(((((((.......)))..)))	(((((..(........)..)))))	(((((....(......).)))))	(((((...(.......)..)))))	(((((((.......)))..))))
2N8V	/	/	/	/	/	/	/	/	/	/		(((((.((((((((....))).))))).((((((((......))))))))))))...............	(((((.((((((((....)).)))))).((((((((......))))))))))))...............	(((((.(((((((......)).)))))).((((((((......)))))))))))))..............	(((((.((((((((......).)))))).(((((((((......)))))))))))))...............	(((((.((((((..........))))))).((((((((......)))))))))))))...............	(((((.((((((.(.....).)))))).((((((((......)))))))))))))...............	(((((.((((.(((....)))..)))).(((((((......)))))))))))))..............	(((((.((((((((....))).)))))).((((((((......))))))))))))(.......).......	(((((.((((((((....)).)))))).((((((((......))))))))))))(.......).......	(((((.(((((.((....))..))))).((((((((......)))))))))))))...............
2NBX	/	/	/	/	/	/	/	/	/	/		((((((...(((((((((((((((..(((((...)))))))))..))))))((((((.(((((((.....)))))))....)))))))........))))).))))))	((((((...((((((((((((((((.(((.........)))))))..))))))(((((((.(((((((.....)))))))....)))))))........))))).))))))	((((((...(((((((((((((((..(((((...)))))))))..))))))(((((((((((((((......)))))))...))))))))........))))).))))))	((((((...(((((((((((((((..(((((...)))))))))..))))))((((((..(((((((.....)))))))....)))))))........))))).))))))	((((((...((((((((((((((.(((.........)))))))..))))))(((((((((((((((......)))))))...)))))))........))))).))))))	((((((...((((((((((((((.(((.........)))))))..)))))(((((((..(((((((.....)))))))......))))))......))))).))))))	((((((...((((((((((.((...(((((...)))))..))..))))))((((((.(((((((.....)))))))....)))))))........))))).))))))	((((((...((((((((((((((..((((.....))))))))...)))))(((((((.((((((....)))))))....))))))).......))))).))))))	((((((...((((((((((((((((..(((((...)))))))))..))))))(((((((((((((((.....))))))..).)))))))........)))))).))))))	((((((...(((((((((((.((.(.(((((...))))).))).))))))(((((((.(((((((.....))))))....)))))))........)))))).))))))
2NBY	/	/	/	/	/	/	/	/	/	/		((((((((((((..(((((...))))))))))..)))))))	(((((((((((.(((.........))))))))..)))))))	(((((((.((...(((((...)))))..))..)))))))	((((((((((((..((((.....))))))))..)))))))	(((((((.((.(.(((((...))))).))).)))))))	((((((((((...(((((...))))).))).)))))))	(((((((.(((..(((((...))))))))...)))))))	(((((((......(((((...)))))......)))))))	(((((((.(((.(((.........))))))...)))))))	((((((((.((..(((.........)))..))..)))))))
2NBZ	/	/	/	/	/	/	/	/	/	/		((((((((.(((((((.....)))))))....))))))))	(((((((((((((((((.....)))))))...)))))))))	(((((((..(((((((.....))))))).....)))))))	((((((((((((((((.....)))))))..).))))))))	((((((((.(((((((.....))))))).)...)))))))	(((((((.((((((((.....)))))))...).)))))))	((((((((..((((((.....)))))).....))))))))	((((((((((((((((.....))))))..)).))))))))	((((((((.((((((((.....)))))))..)..)))))))	((((((((.(((((((.....)))))).)...))))))))
2NC0	/	/	/	/	/	/	/	/	/	/		(((((...(((((....))))).)))))	(((((....((((....)))..))))))	((((....(((((....)))))..))))	.((((...(((((....))))).)))).	(((((..((.(((....))))).)))))	(((((..(.((((....))))).))))	(((((...((((......)))).)))))	(((((.(..((((....))))).)))))	.((((....((((....)))..)))).	(((((...(((.(....).))).))))
2NC1	/	/	/	/	/	/	/	/	/	/		((((((...((((((((((....)))))((((((((....)))))))........))))).))))))	((((((...((((((((((.....))))))((((((......)))))).......))))).))))))	((((((....(((((((((((....))))))((((((....))))))))........))))..)))))))	((((((...((((((((((....))))).((((((....))))).......))))).))))))	((((((...((((((((((......))))))((((((....)))))))........))))).))))))	((((((....((((((((((....))))))((((((......)))))).......))))..))))))	((((((...((((.((((((....))))))((((((....))))))........))))).))))))	((((((...(((((.(((((....)))))(((((((....))))))))........))))).))))))	(((((....((((((((((....))))))((((((....))))))).......)))))..)))))	.(((((...((((((((((....))))))((((((....))))))).......))))).))))).
2NCI	/	/	/	/	/	/	/	/	/	/		((((((....((((....)))))))))))	.(((((....((((....))))))))))).	((((((.....((((....))).))))))	((((((.....((((....)))).)))))	((((((...(.(((....))))))))))	(((((.(...((((....))))))))))	((((((.(...((((....))))))))))	(((((.((...(((....))))))))))	((((((....(((......)))))))))	(((((......(((....)))..)))))
2O33	/	/	/	/	/	/	/	/	/	/		(((..(((....)))..)))	.((..(((....)))..)).	((...(((....)))...))	.....(((....)))....	(((..((......))..)))	/	/	/	/	/
2OJ7	/	/	/	/	/	/	/	/	/	/		....	((....))	.(....).	/	/	/	/	/	/	/
2PCV	/	/	/	/	/	/	/	/	/	/		((((((.....((((....)))).....).)))))	((((((...(.((((....)))).)...).)))))	(((((.((...((((....))))).....)))))))	((((((......((((....))))......)))))	((((.(((...((((....)))).....)))))))	(((.((((...((((....)))))......)))))))	((((((......(((......)))......).)))))	((((((......(((....)))......).)))))	(((((.((.(.(((((....)))).)...)))))))	((((.(((.(.(((((....)))).)...)))))))
2PJP	/	/	/	/	/	/	/	/	/	/		((((((.(((....)))))))))	(((((.((((....)))))))))	((((((.((......))))))))	(((((.(((......))))))))	.(((((.(((....)))))))).	.((((.((((....)))))))).	(((((..(((....))).)))))	.(((((.((......))))))).	.((((.(((......))))))).	(((((..((......)).)))))
2PXB	/	/	/	/	/	/	/	/	/	/		((((((((((....(((....(((....)))....))).)))))))))))	(((((((((.....(((....(((....)))....)))..)))))))))	((((((((...(..(((....(((....)))....)))..)))))))))	((((((((......(((....(((....)))....)))...))))))))	(((((((....((.(((....(((....)))....)))..)))))))))	((((((((((....(((..(.(((....)))..).))).))))))))))	((((((((((....((.....(((....)))....)).))))))))))	.((((((((....(((....(((....)))....))).))))))))).	((((((((((....(((..(.(((....)).).).))).))))))))))	((((((((((....((.(....(((....)))....))).))))))))))
2PXD	/	/	/	/	/	/	/	/	/	/		((((((((((....(((....(((....)))....))).)))))))))))	(((((((((.....(((....(((....)))....)))..)))))))))	((((((((...(..(((....(((....)))....)))..)))))))))	((((((((......(((....(((....)))....)))...))))))))	(((((((....((.(((....(((....)))....)))..)))))))))	((((((((((....(((..(.(((....)))..).))).))))))))))	((((((((((....((.....(((....)))....)).))))))))))	.((((((((....(((....(((....)))....))).))))))))).	((((((((((....(((..(.(((....)).).).))).))))))))))	..((((((((.((.((......))....))))))))))...........
2PXE	/	/	/	/	/	/	/	/	/	/		((((((((((....(((....(((....)))....))).)))))))))))	(((((((((.....(((....(((....)))....)))..)))))))))	((((((((...(..(((....(((....)))....)))..)))))))))	(((.((((((....(((....(((....)))....))).)))))).)))	((.(((((((....(((....(((....)))....))).)))))).))	((((((((......(((....(((....)))....)))...))))))))	.((((((((....(((....(((....)))....))).)))))).))	(((((((....((.(((....(((....)))....)))..)))))))))	((((((((((....(((..(.(((....)))..).))).))))))))))	((((((((((....((......(((....))).....)).))))))))))
2PXF	/	/	/	/	/	/	/	/	/	/		((((((((((....(((....(((....)))....))).)))))))))))	(((((((((.....(((....(((....)))....)))..)))))))))	((((((((...(..(((....(((....)))....)))..)))))))))	((((((((......(((....(((....)))....)))...))))))))	(((((((....((.(((....(((....)))....)))..)))))))))	((((((((((....(((..(.(((....)))..).))).))))))))))	((((((((((....((.....(((....)))....)).))))))))))	.((((((((....(((....(((....)))....))).))))))))).	((((((((((....(((..(.(((....)).).).))).))))))))))	((((((((((....((.(....(((....)))....))).))))))))))
2PXK	/	/	/	/	/	/	/	/	/	/		((((((((((....(((....(((....)))....))).)))))))))))	(((((((((.....(((....(((....)))....)))..)))))))))	(((.((((((....(((....(((....)))....))).)))))).)))	((((((((...(..(((....(((....)))....)))..)))))))))	((.(((((((....(((....(((....)))....))).)))))).))	((((((((......(((....(((....)))....)))...))))))))	.((((((((....(((....(((....)))....))).)))))).))	(((((((....((.(((....(((....)))....)))..)))))))))	((((((((((....(((..(.(((....)))..).))).))))))))))	((((((((((....((......(((....))).....)).))))))))))
2PXL	/	/	/	/	/	/	/	/	/	/		..((((((((....(((....(((....)))....))).)))))))))	...(((((((....(((....(((....)))....))).))))))).	..(((((((.....(((....(((....)))....)))..)))))))	((((.(((((.((.((......))...)))))))).))...))....	...((((((.....(((....(((....)))....)))..)))))).	..((.(((((.((.((......))....))))))).))..........	((((.((((((((.......)))((....)))))).))...))....	((((.(((((.((...((.......)).))))))).))...))....	..((((((...(..(((....(((....)))....)))..)))))))	....((((((....(((....(((....)))....))).))))))...
2PXP	/	/	/	/	/	/	/	/	/	/		((((((((((....(((....(((....)))....))).)))))))))))	(((((((((.....(((....(((....)))....)))..)))))))))	((((((((...(..(((....(((....)))....)))..)))))))))	((((((((......(((....(((....)))....)))...))))))))	(((((((....((.(((....(((....)))....)))..)))))))))	((((((((((....(((..(.(((....)))..).))).))))))))))	((((((((((....((.....(((....)))....)).))))))))))	.((((((((....(((....(((....)))....))).))))))))).	((((((((((....(((..(.(((....)).).).))).))))))))))	((((((((((....((.(....(((....)))....))).))))))))))
2PXQ	/	/	/	/	/	/	/	/	/	/		((((((((((....(((....(((....)))....))).)))))))))))	(((((((((.....(((....(((....)))....)))..)))))))))	((((((((...(..(((....(((....)))....)))..)))))))))	((((((((......(((....(((....)))....)))...))))))))	(((((((....((.(((....(((....)))....)))..)))))))))	((((((((((....(((..(.(((....)))..).))).))))))))))	((((((((((....((.....(((....)))....)).))))))))))	.((((((((....(((....(((....)))....))).))))))))).	((((((((((....(((..(.(((....)).).).))).))))))))))	((((((((((....((.(....(((....)))....))).))))))))))
2PXT	/	/	/	/	/	/	/	/	/	/		((((((((((....(((....(((....)))....))).)))))))))))	(((((((((.....(((....(((....)))....)))..)))))))))	((((((((...(..(((....(((....)))....)))..)))))))))	((((((((......(((....(((....)))....)))...))))))))	(((((((....((.(((....(((....)))....)))..)))))))))	((((((((((....(((..(.(((....)))..).))).))))))))))	((((((((((....((.....(((....)))....)).))))))))))	.((((((((....(((....(((....)))....))).))))))))).	((((((((((....(((..(.(((....)).).).))).))))))))))	((((((((((....((.(....(((....)))....))).))))))))))
2PXU	/	/	/	/	/	/	/	/	/	/		((((((((((....(((....(((....)))....))).)))))))))))	(((((((((.....(((....(((....)))....)))..)))))))))	((((((((...(..(((....(((....)))....)))..)))))))))	((((((((......(((....(((....)))....)))...))))))))	(((((((....((.(((....(((....)))....)))..)))))))))	((((((((((....(((..(.(((....)))..).))).))))))))))	((((((((((....((.....(((....)))....)).))))))))))	.((((((((....(((....(((....)))....))).))))))))).	((((((((((....(((..(.(((....)).).).))).))))))))))	((((((((((....((.(....(((....)))....))).))))))))))
2PXV	/	/	/	/	/	/	/	/	/	/		((((((((((....(((....(((....)))....))).)))))))))))	(((((((((.....(((....(((....)))....)))..)))))))))	((((((((...(..(((....(((....)))....)))..)))))))))	((((((((......(((....(((....)))....)))...))))))))	(((((((....((.(((....(((....)))....)))..)))))))))	((((((((((....(((..(.(((....)))..).))).))))))))))	((((((((((....((.....(((....)))....)).))))))))))	.((((((((....(((....(((....)))....))).))))))))).	((((((((((....(((..(.(((....)).).).))).))))))))))	((((((((((....((.(....(((....)))....))).))))))))))
2QH2	/	/	/	/	/	/	/	/	/	/		((((((((........))))))))	.(((((((........))))))).	(((((((((.....).)))))))))	((((((((.(.....)))))))))	(((((((((..(....)))))))))	((((((((((...)).))))))))	(((((((((.(...)..))))))))	((((.(((.........))).))))	((.(((((........))))).))	/
2QH3	/	/	/	/	/	/	/	/	/	/		((((((((.......))))))))	.(((((((.......))))))).	(((((((((....).))))))))	((((((((.(...).))))))))	(((((((.........)))))))	((((.(((.......))).))))	.((((((((....).))))))).	((.(((((.......))))).))	/	/
2QH4	/	/	/	/	/	/	/	/	/	/		((((((......))))))	(((((........)))))	.(((((......))))).	.((((........)))).	(((.((......)).)))	(((((((...).))))))	/	/	/	/

	DNA model											RNA model									
PDB	RNAfold MFE	subopt #2	subopt #3	subopt #4	subopt #5	subopt #6	subopt #7	subopt #8	subopt #9	subopt #10		RNAfold MFE	subopt #2	subopt #3	subopt #4	subopt #5	subopt #6	subopt #7	subopt #8	subopt #9	subopt #10
2R8S	/	/	/	/	/	/	/	/	/	/		.((((((......((((((....((((.((((((((.((((((((....)))))))))).(((((....))))).)))).....))))))))...)))))))............((((...(((((((((...))))))))..))))))))))).	.((((((......((((((....(((.((((((((.((((((((....))))))))).(((((....))))).))))......))))))))...))))))).............((((...(((((((((...)))))))))..)))))))))).	((..(((((.....((((((....((((.((((((((.(((((((((....))))))))).((((.(....))))).)))).....))))))))...))))))))....)))))))..((((...((((((((((...)))))))))..))))........	((..(((((.....(((((((....(((.(((((((((.((((((((((....)))))))).(((((....))))).))))......))))))))...)))))))....)))))))..((((...(((((((((...)))))))))..))))..........	.((((((((.(...((((((....((((.((((((((.(((((((((....)))))))))).(((((....))))).))))......))))))))...))))))....).)......((((...(((((((((...))))))))))..)))))))))))).	.((((((((.(...((((((((....(((.(((((((((.((((((((((....)))))))))).(((((....))))).))))......))))))))...)))))))....).)......((((...((((((((((...)))))))))..)))))))))))).	((...((((.....(((((((....((((.((((((((.((((((((((....))))))))).(((((....))))).))))......))))))))...)))))))....)))).))..((((...(((((((((...)))))))))..))))........	((...((((.....(((((((....(((.(((((((((.(((((((((....)))))))).(((((....))))).))))......))))))))...)))))))....)))).))..((((...((((((((((...))))))))..))))........	.((((((((.(....((((((....((((.((((((((.(((((((((....)))))))))).(((((....))))).))))......))))))))...)).)))....).)......((((...(((((((((...))))))))))..)))))))))).	.((((((((.(....((((((....(((.(((((((((.(((((((((....))))))))).(((((....))))).))))......))))))))...)).)))....).)......((((...(((((((((...)))))))))..)))))))))).
2RLU	/	/	/	/	/	/	/	/	/	/		(((((((.....)))))))	((((((((...))))))))	.((((((.....)))))).	.(((((((...))))))).	((((((.......))))))	((((((.(....)))))))	(((.(((.....))).)))	((.((((.....)))).))	(((.((((...)))).)))	((.(((((...))))).))
2RO2	/	/	/	/	/	/	/	/	/	/		(((((((((.....)))))))))	((((((((((...))))))))))	((((((((.......))))))))	.((((((((.....)))))))).	.(((((((((...))))))))).	((((((((.(...).))))))))	((((((((......)).))))))	((((((((......).)))))))	((((.((((.....)))).))))	.(((((((.......))))))).
2RPK	/	/	/	/	/	/	/	/	/	/		(((((((......)))))))	.((((((......)))))).	(((((((.(...))))))))	((((((........))))))	((((.((......)).))))	(((.(((......))).)))	/	/	/	/
2RPT	/	/	/	/	/	/	/	/	/	/		((((.(((....))).))))	.(((.(((....))).))).	((((..((....))..))))	(((..(((....)))..)))	((((.((......)).))))	((((.(((....))).)))))	(((.((((....))).))))	(((.((((....)).)))))	((.(((((....))).))))	((.(((((....)).)))))
2RVO	/	/	/	/	/	/	/	/	/	/		((((((((...((((....))))).)))))))	((((((((...((((......)))).)))))))	((((((((.(((..........))).))))))))	((((((((....((((....))))..)))))))	(((((((..(((..........)))..)))))))	((((((((...(((.........))).))))))))	((((((((....((((....))))..)))))))	((((((((.((............)).))))))))	((((((((..((..........))..)))))))	((((((((....(((......)))..)))))))
2TPK	/	/	/	/	/	/	/	/	/	/		........((((((.(.........).))))))..	........(((((((((.....))).))))))..	........((((((.............))))))..	(.....).((((((.(.........).))))))..	........((((((.((......).).))))))..	........(((((..(.........)..)))))..	........(((((((((........)).)))))..	(....)..((((((.(........).)))))))..	........(((((.(((.....)))..)))))..	........(((((.(.........).))))))...
2UWM	/	/	/	/	/	/	/	/	/	/		((((((((((.....))))))))))	((((((((((...))))))))))	.((((((((.....)))))))).	.((((((((...))))))))).	((((((((.......))))))))	(((((.(((.....))).)))))	((((.((((.....)))).))))	(((((.((((...)))).)))))	((((.(((((...))))).))))	/
2V3C	/	/	/	/	/	/	/	/	/	/		((((..(((((.(((((((((....))))))))..)))))....((((((.....(((.....(((....))).....)))..)))))).)))).	((((..(((((.((((((((((....)))))))))..)))))....((((((..(((..((.....(((....))).))...))).)))))).)))).	((((..(((((.((((((((((....)))))))))..)))))....((((((..(((.........(((....)))......))).)))))).)))).	((((..(((((.(((((((((....)))))))))..)))))....((((((..(((((....))(((....)))......))).)))))).)))).	((((..((((..(((((((((....)))))))))..))))......((((((.....(((......(((....)))......)))..)))))).)))).	((((..(((((.(((((((((....)))))))))..)))))....((((((.....(((....((((.....)).))..)))..))))).)))).	(((((.((((..((((((((....))))))))..)))))....((((((....(((......(((....)))......)))..)))))).)))).	((((..((((..((((((((((....)))))))))..)))).....(((((..(((..((.....(((....))).))...))).))))).)))).	(((((.((((..((((((((((....)))))))))..)))))....((((((..(((..((.....(((....))).))...))).))))).)))).	((((..((((..((((((((((....)))))))))..)))).....((((((..(((.........(((....))).....))).))))).)))).
2VPL	/	/	/	/	/	/	/	/	/	/		((((((((..((((((((....)))))).))).......))))))))).	((((((((..((((((((....))))).))))......))))))))).	((((((((..((((((((......))))).)))......)))))))))).	(((((((((..(((((((......))).))))......))))))))).	((((((((..(((.((((....)))).)))......))))))))).	.(((((((..((((((((....)))))).)))......)))))))...	.(((((((..((((((((....)))).))))......)))))))..	((((((((..((((.(((....)))..))))......)))))))).	((((((((...((((((((....)))))).)))......))))))).	((((((((...((((((((....)))).))))......)))))))).
2W2H	/	/	/	/	/	/	/	/	/	/		((((((((((....))))))))))	((((((((......))))))))	(((((((.........)))))))	.((((((((....)))))))).	(((((((..(....))))))))	.(((((((......))))))).	(((((((((.(...)))))))))	(((((((.((...)))))))))	((((((((.(....).)))))))	((((((..........))))))
2XXA	/	/	/	/	/	/	/	/	/	/		((((((((((...((.(((..((((.((((((...(((((....)))))((((((....((....))..)))))).))))))))))))))).))))))))))	((((((((((.((((((((.(((((((((((.....((((.....(((....)))....))))...))).)).))))))..))).)))))...))))))))))	((((((((((.((((((((.(((((((((((......((((....(((....)))....))))...))).)).))))))..)).)))))))...))))))))))	((((((((((...((.(((((..((.((((((...(((((....)))))((((((....((....))..)))))).)))))))))))))).))))))))))	((((((((((...((.(((..(((.((((((...(((((....)))))..(((....))).(((((....)))))))))))))))))))).))))))))))	((((((((((.((((((((.((((((((((.....((((....(((....)))....))))...))).)).))))))).).))).)))))...))))))))))	((((((((((.((((((((.(((((((((((.....((((....(((....))).....))))...))).)).))))).).)).))))))...))))))))))	((((((((((.((((((((.((((((((((....(((((....)))))..(((....)))....))).)).))))))..))).))))))...))))))))))	((((((((((.((((((((.(((((((((((....(((((....)))))..(((....)))....))).)).))))))..)).))))))...))))))))))	((((((((((...((.((...(((((.((((((...(((((....)))))((((((....((....))..)))))).)))))))))).)))).))))))))))
2Y8W	/	/	/	/	/	/	/	/	/	/		(((((((....)))))))..	.((((((....))))))...	((((((......))))))..	(((((........)))))..	..(((((....)))))....	.(((((......)))))...	(((((.(....).)))))..	/	/	/
2Y8Y	/	/	/	/	/	/	/	/	/	/		(((((((....))))))).	.((((((....))))))..	((((((......)))))).	(((((........))))).	..(((((....)))))...	.(((((......)))))..	(((((.(....).))))).	/	/	/
2Y95	/	/	/	/	/	/	/	/	/	/		(((((....)))))	.((((....)))).	((((......))))	(((((...).))))	/	/	/	/	/	/
2Y9H	/	/	/	/	/	/	/	/	/	/		(((((((....)))))))	.((((((....)))))).	((((((......))))))	..(((((....)))))..	.(((((......))))).	(((((........)))))	.((((........)))).	(((((.(....).)))))	/	/
2ZM5	/	/	/	/	/	/	/	/	/	/		(((((((..((((........)))))).(((((((...)))))))......(((((.......))))))))))))))..	(((((((..((((........)))).((((((.....))))))......(((((.......))))))))))))))..	((((((((.((((........)))).(((((((...))))))))......((((.......)))))))))))..	((((((....((((........))))).(((((((...)))))))......(((((.......))))).)))))))..	((((((((.((((........)))).((((((.....))))))......((((........))))))))))))..	((((((...((((((((((.((.((((((((((...))))))).)).).)).....)))))..))))))))))..	((((((...((((((((.((.(((((((((((...))))))).))).))....))))))..)))))))))..	((((((...((((........)))).((((((.....)))))).....(((((.......))))).))))))..	((((((...((((((((((..(((((((((((...))))))).))..)))...))))))..)))))))))..	((((((((.((((........)))).(((((((...))))))).......(((....))))))))))..
2ZNI	/	/	/	/	/	/	/	/	/	/		(((((((.((((.....)))).((((((.......))))))...(((((.......)))))))))))))....	(((((((.((((.....)))).((((((.......))))))...(((((.(...).))))))))))))))....	(((((((((((((.....)))).((((((.......))))))....((((.......))))))))))))....	(((((((.((((.....)))).(((((.........)))))...(((((.......))))))))))))))....	((((((..((((.....)))).((((((.......))))))...(((((.......))))).))))))).....	((((((.(((((.....)))).((((((.......))))))...(((((.......))))).).)))).)).	((((((((((((.....)))).((((((.......))))))....((((.(...).))))))))))))))....	(((((((.((((.....)))).((((((.(...).))))))...(((((.......)))))))))))))).....	(((((((.((((.....)))).((((((.......))))))....((((.......)))).))))))))....	(((((((.((((.....)))).((((..........))))...(((((.......)))))))))))))....
2ZUE	/	/	/	/	/	/	/	/	/	/		((((((((..(((....)))...((((.(((((........)))))..))))((((........)))))))))))).	((((((((.((((....))).).((((.(((((........)))))..))))((((........))))))))))))).	((((((((..((......))...((((.(((((........)))))..))))((((........)))))))))))).	((((((((.(((......)).).((((.(((((........)))))..))))((((........)))))))))))).	((((((((...........((((...(((((...........)))))..))))((((........)))))))))))).	((((((((.((...))..((((...(((((...........)))))..))))((((........))))))))))))).	((((((((...(((....)))...((((.(((((........)))))..))))((((........)))).))))))).	((((((((..(((....)))...((((.(((((........))))).))))((((....))))).))))))).	((((((((..((((....))).).(((.(((((........)))))..)))((((........)))).))))))).	((((((((.((((....))).).((((.(((((........)))))..))))((((....))))).))))))).
2ZZM	/	/	/	/	/	/	/	/	/	/		(((((((........((((......))))(((((((....).))))))(((....)))...((((((........))))))))))))	(((((((........((((......))))((((((........))))))(((....)))...((((((........))))))))))))	((((((((.......((((......))))((((((....).))))))(((....)))....((((.......))))))))))))	(((((((..((((....))))...((((.....)))).((((((..........))))))((((((........)))))))))))))	(((((((........((((......)))((((((....).)))))(((...)))....(((((.......)))))))))))))	(((((((..((((....))).......((((((....).))))))(((....)))...((((((.......))))))))))))	(((((((........((((......)))).((((((....).))))).(((....)))...((((((........))))))))))	....((((......((((((((....).)))))))....)))))......((((((...(((((.......))))))))))))	(((((((..(((..((((......))))....)))...((((((..........))))))(((((........))))))))))))	(((((((........((((......))))((((((.(...).))))))(((....)))...((((((........))))))))))))
2ZZN	/	/	/	/	/	/	/	/	/	/		(((((((..((((((...))))))).(((((........)))))......(((((........))))))))))))	(((((((..((((((...)))))).((((((....).)))))......(((((........))))))))))))	(((((((..((((((...)))))).(((((.(....))))))......(((((........))))))))))))	(((((((..((((((...))))))).((((..........))))......((((((.......))))))))))))	(((((((..((((((...)))))).(((((.(...).)))))......(((((........)))))))))))))	(((((((..(((((.....))))).(((((........))))).......(((((........))))))))))))	(((((((..(((((.....))))).((((((....).)))))......(((((.......))))))))))))	(((((((..((((((...)))))).(((((........))))).....(((((.(...).)))))))))))))	.((((((..((((((...)))))).(((((........)))))......(((((........)))))))))))).	(((((((((((((....))))).....(((((........)))))......((((........))))))))))))
3A2K	/	/	/	/	/	/	/	/	/	/		(((((((..(((....((((((((((.....)))).))))))...))).(((((........))))))))))))....	((((((...(((....((((((((((.....)))).))))))...))).)(((((........))))).)))))))....	(((((((..(((..(.((((((((((.....)))).))))))).).))).(((((........)))))))))))).....	(((((((..((.(.(.((((((((((.....)))).))))))).).))).(((((........))))))))))))....	(((((((..((.(...(((((((((((.....)))).))))))...))).(((((........))))))))))))....	(((((((..(((..(..((((((((((.....)))).))))))..).))).(((((........)))))))))))))....	(((((((..(((.....(((((((((.....)))).)))))....))).((((((.......)))))))))))))....	(((((((..((.(.(..(((((((((.....)))).))))))..).))).(((((........))))))))))))....	(((((((..((......((((((((((.....)))).))))))....)).(((((........)))))))))))....	((((((...(((..(.(((((((((((.....)))).)))))).).)))).(((((........))))).)))))))....
3A3A	/	/	/	/	/	/	/	/	/	/		(((((((.(..((((((....)))))))((((((........)))))).(((((....))))).((((........))))).)))))))	(((((((....((((((....))))))((((((........)))))).(((((....))))).((((........))))..)))))))	(((((((.(..((((((....)))))))).(((((........))))))..(((((....))))).((((.......))))).)))))))	(((((((....((((((....)))))))).(((((........)))))..(((((....))))).((((........)))))..)))))))	((((((.....((((((....))))))((((((........)))))).(((((....))))).((((.......))))...)))))))	((((((..(..((((((....))))))((((((........)))))).(((((....))))).((((........)))))..)))))))	(((((......((((((....))))))((((((........)))))).(((((....))))).((((........))))....))))	(((((((.(..((((((....))))))(((((((.(....))))))).(((((....))))).((((.......))))).)))))))	(((((((.(((((.(((....)))))))((((((........))))).(((((....))))).((((........))))).)))))))	(((((((.(..((((((....))))))(((((((.(...).)))))).(((((....))))).((((........))))).)))))))

	DNA model											RNA model									
PDB	RNAfold MFE	subopt #2	subopt #3	subopt #4	subopt #5	subopt #6	subopt #7	subopt #8	subopt #9	subopt #10		RNAfold MFE	subopt #2	subopt #3	subopt #4	subopt #5	subopt #6	subopt #7	subopt #8	subopt #9	subopt #10
3ADB	/	/	/	/	/	/	/	/	/	/		((((((((..((((((....))))))((((((.......))))))((((((....)))))))((((.......)))))))))))))....	((((((((..((((((....))))))((((((....).))))))((((((....))))))((((.......)))))))))))))....	((((((((..((((((....))))))(((((.........)))))(((((((....)))))))(((.......))))))))))))....	((((((((..((((((....))))))((((((.(...).))))))((((((....))))))((((.......)))))))))))....	((((((((..((((((....))))))((((((.(....)))))))((((((....)))))))((((.......)))))))))))))....	((((((((..((((((....))))))((((((.......))))))((((((....))))))((((......)))).))))))))....	((((((((..((((((....))))))((((((....).))))))((((((....))))))((((......)))).))))))))....	(((.((((((.((((((....))))))((((((.......))))))((((((....))))))((((.......)))..))))).))).	(((.((((((.((((((....))))))((((((.......))))))..(((((....))))((((((.......))))))))))).))).	(((.((((((.((((((....))))))((((((((....).))))))((((((....)))))))((((......)))).))))).))).
3AKZ	/	/	/	/	/	/	/	/	/	/		((((((((((((.......)))))))((((((.(((((..(....)..))))))))))....))))))....	(((((((..((((.......))))((.((((((.(((((..(....)..)))))))))).)))))))))).....	.(((((((((((.......)))))))((((((.(((((..(....)..))))))))))....))))).....	((((((((((((.......)))))))((((((.((((((.......).)))))))))....))))))....	((((((((((((.......))))))).((((((.(((((..(....)..))))))))))...))))))))....	(((((((.(((((.......)))))(((((.......)))))....(((((.......)))))))))))).....	((((((.(((((.......)))))..(((((.(((((..(....)..)))))))))))...))))))))....	((((((((((((.......))))))((((((.((((((.(....)).))))))))))...))))))))....	(((((((.(((((.......)))))(((((((....).)))))....(((((.......)))))))))))....	.((((((..((((.......))))((.((((((.(((((..(....)..)))))))))).))))))))......
3AMT	/	/	/	/	/	/	/	/	/	/		(((((((..((((....)))).((.(((((((.......))))))).))(((((.......)))))))))))....	(((((((..(((......)))..((.(((((((.......))))))).))(((((.......))))))))))))....	(((((((...............((.(((((((.......)))))).)).))(((((.......))))))))))))....	(((((((..((...)).......((.(((((((.......))))))).))(((((.......)))))))))))....	((((((..((((....))).).((.((((((.......)))))).)).))(((((.......)))))))))))))....	((((((...((((....)))...((.(((((((.......))))))).))(((((.......))))))))))))....	((((((((.((((....)))).)((.(((((((.......))))))).))(((((.......))))))))))))....	(((((((..((........))..((.(((((((.......))))))).))(((((.......))))))))))))....	((((((...((((....))))..((.(((((((.......))))))).))(((((.......))))).))))))))....	(((((((..((((....))))..((.(((((((.(....)))))))).))(((((.......)))))))))))))....
3EGZ	/	/	/	/	/	/	/	/	/	/		(((((..(((((..........)))))(((((((..........)))))))).......)))))	(((((...((((..........))))((((((((..........))))))))........)))))	(((((..(((((..(....)...)))))((((((((..........)))))))........)))))	(((((...((((..(....)...))))((((((((..........))))))))........)))))	(((((..(((((..........)))))).((((((..........))))))..........)))))	(((((...((((...........)))).(((((((..........)))))))..........)))))	(((((...(((..........)))...((((((((..........))))))))........)))))	((((((.(((((..........)))))((((((((..........))))))))......).)))))	.((((..(((((..........)))))(((((((..........))))))........)))).	(((((.((((((..........)))))(((((((..........))))))))....)...)))))
3EPJ	/	/	/	/	/	/	/	/	/	/		(((((((.((((.......))))((((((((...))))))))((((........))))....)))))))	(((((((.((((.......))))(((((((.....)))))))((((........))))....)))))))	((((((..((((.......))))((((((((...))))))))..(..(((((.......))))))))))))	(((((((.((((.......)))).(((((((...)))))))).((((........))))....)))))))	(((((((.((((.......))))((((((((...))))))))((((........)).))...)))))))	((((((..((((.......))))(((((((.....)))))))....(((((.......))))))))))	((((((...(((.......)))((((((((...)))))))))...(((((.......))))))))))	((((((((((.(.(((..((.(((((((((...))))))))))..))).).)).)).))...))))))))	(((((((.((((.......)))).((((((.....)))))).((((........))))....)))))))	((((((..((((.......)))).(((((((...)))))))......(((((.......))))))))))
3FOZ	/	/	/	/	/	/	/	/	/	/		(((((((..((((........))))).(((((((...)))))))......(((((.......))))))))))))..	(((((((..((((........)))).((((((.....))))))......(((((.......)))))))))))))..	((((((((.((((........))))).((((((...))))))))......((((.......)))))))))))))..	((((((...((((........)))).(((((((...)))))))......(((((.......))))).))))))..	((((((((.((((........)))).((((((.....))))))......((((.......)))))))))))))))..	((((((...((((((((((((.((.((((((((((...)))))))).))).)).....))))))..)))))))))..	(((((((...(((((((((((.((.((((((((((...)))))))).))).))....))))))..)))))))))))..	((((((...((((........)))).((((((.....))))))....(((((.......))))).))))))..	((((((...((((((((((..((((((((((((...)))))))).))..)))...))))))..))))))))))..	((((((((.((((........)))).((((((...)))))))..........(((....))))))))))))..
3ID5	/	/	/	/	/	/	/	/	/	/		(((...)))((..((((....))))...))....	.((((((.......))))))..............	((((...)))..((((....)))))........	.((((((....).))))).............	((((((.......)))))).).............	(((...)))....((((....))))........	(.(((((...(.....)...))))).)......	(.(((((..............)))))).)......	(((...))).(..((((....))))..).....	(((((((....).)))))).)...........
3IVK	/	/	/	/	/	/	/	/	/	/		((((((((..........))))))))................((((...))))((((((((((((.....)))))..(((((....)))))((.(((((....)))).)))))))))).........	((((((((..........)))))))).......(((....)))((((...)))(((((((((((((.....)))))..(((((....)))))((.(((((....))))).)))))))))).........	((((((((..........))))))))................((((...)))((((((.(((((.....)))))..(((((....)))))((.(((((....))))).)).)))))).........	((((((((..........))))))))................((((...))))(((((((((((.....)))))..(((((....)))))((..((((....))))..)))))))))).........	((((((((..........))))))))................((((...))).((((((((((.....)))))..(((((....)))))((.(((((....))))).))))))))).........	((((((((..........)))))))).......(((....)))((((...)))((((((.((((((.....)))))..(((((....)))))((.(((((....))))).)).)))))).........	((((((((..........)))))))).......(((....)))((((...))))(((((((((((.....)))))..(((((....)))))((..((((....)))))..))))))))).........	((((((((..........)))))))).......(((....)))((((...)))).(((((((((((.....)))))..(((((....)))))((.(((((....))))).))))))))).........	.((((((((..........))))))))................((((...))))(((((((((((((.....)))))..(((((....)))))((.(((((....))))).)))))))))).........	((((((((..........))))))))................((((...))))(((((((((((((.....)))))..(((((....)))))((.((((......)))).)))))))))).........
3K0J	/	/	/	/	/	/	/	/	/	/		(((((((((((((........))))))))((.((.(........).)).)).((((..((((......))))..))))..))))))	(((((((((((((........)))))))).....................((.((((..((((......))))..)))))))))))))	(((((((((((((........)))))))((.((.........)))...((.((((..((((.......))))..))))))))))))))	(((((((((((((........))))))))).(.((((.(...((.(((.(((....))).))).)).)...)))))).....))))))	(((((((((((((........))))))).(.((((.(......(((.(((....))).)))......)))).).....))))))	(((((((((((((........))))))))..((..........))....((.((((..((((......))))..))))))))))))	(((((((((((((........))))))))..((..........))...((.((((..((((......))))..))))))))))))))	(((((((((((((........))))))))..((.((.....)).))...((.((((..((((......))))..))))))))))))	(((((((((((((........))))))))...((((.(...((.(((.(((....))).))).))...))))).......))))))	(((((((((((((........))))))))...((.(........).)).((.((((..((((......))))..)))))))))))))
3KTW	/	/	/	/	/	/	/	/	/	/		.((((((((((((((((((((((....)))))))))))).))))..((.((((.....)))).))(((.(((..(....)..))).))).)))))))	.((((((((((((((((((((((....)))))))))).)))))..((.((((.....)))).))(((.(((..(....)..))).))).)))))))	.(((((((((((((((((((((((....))))))))))).))))..((.((((.....)))).))((.(((..........))).))).)))))))	.((((((((((((((((((((((....)))))))))).))))))..((.((((.....)))).))(((.(((..........))).))).)))))))	.(((((((((((((((((((((((....))))))))))).))))....(((((....((.....)))))))..(((.((....)).))))))))))))	.(((((((((((((((((((((((....))))))))))).)))))....(((((....((.....)))))))..(((.((....)).)))))))))))	.(((((.((((((((((((((((....))))))))))).)))))....((((.....))))((.(((.(((..(....)..)))).))).)))))))	.(((((.(((((((((((((((....))))))))))).))))))....((((.....))))((.(((.(((..(....)..))).))).)))))))	.((((((((((((((((((((((....))))))))))).))))).(((.((((.....)))).))).(((....)).(((....)))..)))))))	.((((((((((((((((((((((....))))))))))).))))).....((((.....))))...(((.(((..(....)..))).))).)))))))
3NDB	/	/	/	/	/	/	/	/	/	/		((((((((((((((((.(((((..(((((.((((((((((....)))))))))..)))))....((((((.....(((.....(((....)))......)))...)))))).))))))).))))))))....)))))))	((((((((((((((((.(((((..(((((.(((((((((....))))))))))..)))))....((((((..(((..((....(((....))).))....)))).)))))).))))))).))))))))...)))))))	((((((((((((((((.(((((..(((((.((((((((((....)))))))))..)))))....((((((..(((.........(((....)))......))).))))))).))))))))).))))))))...)))))))	((((((((((((((((.(((((..(((((.(((((((((....))))))))..)))))....((((((..(((((....))((((....)))......))).)))))).))))))).))))))))....)))))))	((((((((((((((((.((((((..(((((.(((((((((....)))))))))..)))))....((((((.....(((.....(((....))).....)))..)))))).))))))).))))))))...))))))	((((((((((((((((.((((((..((((..(((((((((....)))))))))..))))......((((((.....(((.....(((....)))....)))..)))))).))))))).))))))))...)))))))	((((((((((((((((.(((((..(((((.((((((((((....))))))))))..)))))....((((((.....(((....((((.....)).))..))).)))))).))))))).))))))))...)))))))	((((((((((((((((.(((((((.((((..(((((((((....))))))))))..)))))....((((((.....(((.....(((....))).....)))..)))))).))))))).))))))))...)))))))	(((((((((((((((.((.((((((.(((((.(((((((((....)))))))))..)))))....((((((.....(((.....(((....)))......))..)))))).))))))).).))))))...)))))))	((((((((((((((((.((((((.(((((.(((((((((....))))))))))..)))))....((((((..(((..((....(((....))).))....)))).)))))).))))))).)))))))))...)))))))
3NVK	/	/	/	/	/	/	/	/	/	/		...((((.((....)))).))...	.((.((.......))))).......	..(((((.((....)))).)).).	...((...((....))...))...	.....((.((....))))......	.(((.(......).))).......	......(.((....))).......	........((....))........	...((.(.((....)))..))...	....(((.((....)))).)....
3PHP	/	/	/	/	/	/	/	/	/	/		((((((((......)))))))).	(((((((........))))))).	((((((((((...).))))))))).	.(((((((......)))))))...	((.(((((......))))).)).	(((.((((......)))).))).	/	/	/	/
3SN2	/	/	/	/	/	/	/	/	/	/		((((((.(((((......)))))))))))))	((((((.((((((...)).)))))))))))	((((((.((((((...).)))))))))))	.(((((.(((((......)))))))))).	((((((.((((........))))))))))	(((((..(((((......)))))).)))))	((((((.(((.((...))..)))))))))	.(((((.((((((...)).))))))))).	.(((((.((((((...).))))))))))).	..((((.((((((......)))))))))..
3TUP	/	/	/	/	/	/	/	/	/	/		(((((((....((((((((.........)))))))......((((.(((((.....))))).)))).))))))))..	(((((((....(((((((..........)))))))......((((.((((......)))).)))).)))))))..	(((((((....(((((((.((.....)))))))))......((((.(((((....))))).)))).))))))..	(((((((....(((((((.((.....)))))))))......((((.(((((......)))).)))).))))))))..	(((((((....((((((.(((.....)))))))))......((((.(((((....))))).)))).))))))))..	(((((((....((((((.(((.....)))))))))......((((.((((......)))).)))).))))))))..	(((((((.(((..((((((....((.((..((((........))))..))))...))))))..)))))))))..	(((((((.((((((((((..........))))))).((((((.......))))))........)))))))))..	(((((((.(((..((((((....((..((.((((.......))))))...))...))))))..))))))))))..	(((((((.(((..((((((....((..(((.(((........))))).)..))...))))))..)))))))))))..
3U4M	/	/	/	/	/	/	/	/	/	/		..........(((..(((((..(((.(((..((((((((....)))))))))....))).)))......)))))..))).	(((.((.((.(((.....(.(((..((((.....((((((((....))))))))))..)))).))).)....)))))))).....	((((.((.((...)).)).((.((((.....((((((((....))))))))..)))).))((((......))))..))))	..........(((..(((((..((((.....((((((((....))))))))...)))).............)))))..))).	(((.((.(((.....(.(((..((((.(...((((((((....))))))))).)))).))).)....))))))).....	((((.((.((...)).)).((.((((.(...((((((((....)))))))))).)))).))((((......))))..))))	(((((((.((...)).)))((.((((.....((((((((....))))))))..)))).))((((......))))..))))	((((...............((.((((.....((((((((....)))))))..)))).))((((......)))))..))))	(.....)...(((..(((((..(((.(((..((((((((....))))))))......))).)))......)))))..))).	.(.....)...(((..(((((..(((.(((..((((((((....))))))))......))).)))......)))))..))).
3W1K	/	/	/	/	/	/	/	/	/	/		(((((((...((((((....))))))((((((.......)))).))(((((((.....))))))).((((........)))))).))))))).	(((((((....(((((....))))).((((((.......)))).))((((((((.....))))))).(((((.......))))).))))))).	(((((((...((((((....))))))((((((........))))).)).((((((.....))))))..((((((.......))))).)))))))).	((((((....((((((....))))))((((((.......)))).))((((((((.....))))))).(((((.......)))))..)))))))).	((((((...((((((....))))))(((((.........))).))((((((((.....)))))))).(((((.......))))).))))))).	(((((((...((((((....))))))((((((.(...).)))).))(((((((.....))))))).(((((.......))))).))))))).	(((((((...((((((....))))))((((((.......)))).))((((((.......)))))).(((((.......))))).)))))))).	(((((((...((((((....))))))(.((((.......)))).).((((((((.....)))))))).(((((.......))))).))))))).	(((((((...((((((....))))))..((((.......))))...(((((((.....))))))).(((((.......))))).))))))).	(((((((...((((((....))))))((((((.(....))))).))(((((((.....)))))))).(((((......))))).))))))).

	DNA model											RNA model									
PDB	RNAfold MFE	subopt #2	subopt #3	subopt #4	subopt #5	subopt #6	subopt #7	subopt #8	subopt #9	subopt #10		RNAfold MFE	subopt #2	subopt #3	subopt #4	subopt #5	subopt #6	subopt #7	subopt #8	subopt #9	subopt #10
3W3S	/	/	/	/	/	/	/	/	/	/		((((((...(((.(((....))))))(((((.......))))).)((((.((((....)))).))))(((((.......))))))))))))...	((((((...(((.(((....)))))).(((((.......)))))..((((.((((....)))).))))(((((.......))))))))))))...	((((((...((.((((....))))))(((((.......))))).)((((.((((....)))).))))(((((.......))))))))))))...	((((((...((.((((....)))))).(((((.......)))))..((((.((((....)))).))))(((((.......))))))))))))...	((((((...((.((((....)))).))(((((.......))))).((((.((((....)))).))))(((((.......))))))))))))...	((((((((..(((.(((....))))))(((((.......))))).)(((.((((....)))).)))).(((((.......))))))))))))...	(((((((..(((.(((....)))))).(((((.......)))))..((((.((((....)))).)))).(((((......))))))))))))...	((((((((..((.((((....))))))(((((.......))))).)((((.((((....)))).)))).(((((.......))))))))))))...	((((((((..((.((((....)))))).(((((.......)))))..((((.((((....)))).)))).(((((.......))))))))))))...	((((((((..((.((((....))).))(((((.......)))))..((((.((((....)))).)))).(((((.......))))))))))))...
3WC1	/	/	/	/	/	/	/	/	/	/		(((((((..((.((((((((((...........)))))).)))).))((((((.......)))))))))))))..	(((((((..((.((((((((((.((.....)).)))))).)))).))(((((.......)))))))))))))..	((((((...((.((((((((((...........)))))).)))).))(((((.......))))).)))))))..	((((((((.((.((((((((((...........)))))).)))).)).((((.......)))))))))))))..	(((((((..(..((((((((((...........)))))).))))..)(((((.......)))))))))))..	((((((...((.((((((((((.((.....)).)))))).)))).))(((((.......))))).))))))..	(((((((.....(((((((((((...........)))))).))))...(((((.......)))))))))))))..	((((((((.((.(((((((((((.((.....)).)))))).)))).)).((((.......))))))))))))..	(((((((..((....((((((...........))))))......))((((((.......)))))))))))))..	(((((((..(..((((((((((.((.....)).)))))).))))..))(((((.......)))))))))))))..
3WC2	/	/	/	/	/	/	/	/	/	/		(((((((..((((........)))).....(((((((((.(((.(.....).))))))))))))))))))))..	(((((((..((((........))))......(((((((((.(((.........))))))))))))))))))))..	(((((((..((((........))))......(((((((((..(((........)))))))))))))))))))..	((((((...((((........)))).....(((((((((.(((.(.....).))))))))))))).))))))..	((((((((.((((........))))......((((((((.(((.(.....).))))))))))))))))))))..	((((((...((((........))))......(((((((((.(((........)))))))))))).)))))))..	((((((((.((((........))))......((((((((.(((.........))))))))))))))))))))..	(((((((..((((........))))......((((((((.(((.((...)).))))))))))))))))))))..	((((((...((((........))))......(((((((((..(((........))))))))))).))))))..	((((((((.((((........))))......((((((((..(((........))))))))))))))))))..
3WFQ	/	/	/	/	/	/	/	/	/	/		(((((((..((((........)))).(((((.......)))))......(((((.......))))))))))))).	(((((((..((((........)))).(((((.(....)))))).....(((((.......))))))))))))).	(((((((..((((........)))).((((.........))))....(((((.......)))))))))))).	(((((((..((((........)))).(((((.(...).)))))......(((((.......))))))))))))).	(((((((..(((((.....).)))).(((((.......))))).....(((((.......))))))))))).	((((((...((((........)))).(((((.......))))).....(((((.......))))).)))))).	(((((((..((((........)))(((((.......))))).)...(((((.......)))))))))))).	(((((((..((((........))))((((((.......)))).))..(((((.......)))))))))))).	(((((((..((((........)))).(((((.......)))))(...)(((((.......))))))))))))).	((((((((.((((........))))(((((.......))))).....(((((.......))))))))))))).
3WFS	/	/	/	/	/	/	/	/	/	/		(((((((..((((........)))).(((((.......)))))......(((((.......))))))))))))..	(((((((..((((........)))).(((((.(....)))))).....(((((.......))))))))))))..	(((((((..((((........)))).((((.........))))....(((((.......)))))))))))..	(((((((..((((........)))).(((((.(...).)))))......(((((.......))))))))))))..	(((((((..(((((.....).)))).(((((.......))))).....(((((.......)))))))))))..	((((((...((((........)))).(((((.......))))).....(((((.......))))).))))))).	(((((((..((((........)))(((((.......))))).)...(((((.......)))))))))))..	(((((((..((((........))))((((((.......)))).))..(((((.......)))))))))))..	(((((((..((((........)))).(((((.......)))))(...)(((((.......))))))))))))..	((((((((.((((........))))(((((.......))))).....(((((.......))))))))))))..
3WQY	/	/	/	/	/	/	/	/	/	/		(((((((.......((((((((((.((((((((((...)))))))).).).))))...))))))))))))))....	(((((((.......((((((((((.((((((((((...))))))).)).).))))...))))))))))))))....	(((((((.......((((((((((((.((((((((...))))))).))..))))...)))))))))))))))....	(((((((.......((((((((((.(.((((((((...)))))))...).))))...))))))))))))))....	(((((((.......((((((((((.((((((((((...))))))))...))))))...)))))))))))))....	(((((((.......((((((((((((.((((((((...)))))))).)))..)))...))))))))))))))....	(((((((.......((((((((.(((.((((((((...)))))))).)))...))...))))))))))))))....	(((((((.......((((((...(((.((((((((...))))))).).)))........))))))))))))))....	(((((((.......((((((((((.(((((((((((...))))))...))))))))...))))))))))))))....	(((((((.......((((((((((.(((((((((((...)))))))...)))))))...))))))))))))))....
3WQZ	/	/	/	/	/	/	/	/	/	/		(((((((.......((((((((((.((((((((((...)))))))).).).))))...))))))))))))))....	(((((((.......((((((((((.((((((((((...))))))).)).).))))...))))))))))))))....	(((((((.......((((((((((((.((((((((...))))))).))..))))...)))))))))))))))....	(((((((.......((((((((((.(.((((((((...)))))))...).))))...))))))))))))))....	(((((((.......((((((((((.((((((((((...))))))))...))))))...)))))))))))))....	(((((((.......((((((((((((.((((((((...)))))))).)))..)))...))))))))))))))....	(((((((.......((((((((.(((.((((((((...)))))))).)))...))...))))))))))))))....	(((((((.......((((((...(((.((((((((...))))))).).)))........))))))))))))))....	(((((((.......((((((((((.(((((((((((...))))))...))))))))...))))))))))))))....	(((((((.......((((((((((.(((((((((((...)))))))...)))))))...))))))))))))))....
484D	/	/	/	/	/	/	/	/	/	/		((((((..((.......)))))))))	((((((.((........))))))))	((((((.(((........))))))))	((((((((((....)))...)))))))	((((((............)))))))	((((((...((.......)).))))))	(((((((((((....))))...))))))	((((((.(.........).)))))))	((((((.((....))....)))))))	((((((.(..........))))))))
4A4S	/	/	/	/	/	/	/	/	/	/		(((((((((....)))))))))	.((((((((....)))))))).	((((((((......))))))))	((((((.((....)).))))))	/	/	/	/	/	/
4AL5	/	/	/	/	/	/	/	/	/	/		.(((((.....)))))	..((((.....)))).	...(((.....)))..	.(.(((.....))).)	.((((.......))))	/	/	/	/	/
4AL7	/	/	/	/	/	/	/	/	/	/		(((((.....)))))	.((((.....)))).	..(((.....)))..	(.(((.....))).)	((((.......))))	/	/	/	/	/
4BW0	/	/	/	/	/	/	/	/	/	/		(((...(((....)))......))).	.(((..(((....)))......))).	..(((.(((....)))......))).	((....(((....)))......))..	.((...(((....)))......))..	((.(..(((....)))......))).	(.((..(((....)))......))).	(((...((......))......))).	((....(((....)))........)).	..((..(((....)))......))..
4C4W	/	/	/	/	/	/	/	/	/	/		..((((......)))).....((((....))))..	(.((((......)))).)...((((....))))..	((((((......)))).)).((((....))))..	..(((........))).....((((....))))..	..((((......)))).....((((......)))..	((((((......)))..))).((((....))))..	......(((........))).(((())))..	((((..(((........)))..))))..........	(.(((........))).)...((((....))))..	..(((((...).)))).....((((....))))..
4C7O	/	/	/	/	/	/	/	/	/	/		((((((((((((..(((((....)))))..))))).).))))))))	.((((((((((((..(((((....)))))..))))).).))))))).	((((((((((((((((((....)))).))).))))).).))))))))	(((((((((((((((((((....)))).)).))))).).))))))))	.((((((((((((((((((....)))).))).))))).).))))))).	.((((((((((((((((((....)))).)).))))).).))))))).	((((((((.(((((..(((((....)))))..)))))...))))))))	((((((((((((((((.((((....))))))).))))).).))))))))	((((((((((((((((.((((....))))).))))).).))))))))	(((((((((((((((.(((((....)))))).))))).).))))))))
4ILM	/	/	/	/	/	/	/	/	/	/		....	.....((((...))))	.(((........))).	......(((...))).	.....(((.....)))	......((.....)).	.((..........)).	.....((.......))	(........)......	..((......))....
4KR7	/	/	/	/	/	/	/	/	/	/		(((((((.......(((((....)))))))))))))....	((((((((.((.(((...))).))...))))))))....	((((((((.......((((....))))))))))))....	((((((((.((..((...))..)).. .))))))))....	((((((........(((((....))))).))))))....	((((((((.(..(((...)))..)...))))))))....	((((((((....(((...)))......)))))))))....	((((((((........(((....))).))))))))....	(((((((..((.(((...))).))....)))))))....	(((((((......(.((((....))))))))))))....
4KR9	/	/	/	/	/	/	/	/	/	/		(((((((.......(((((....)))))))))))))....	((((((((.((.(((...))).))...))))))))....	((((((((.......((((....))))))))))))....	((((((((.((..((...))..)).. .))))))))....	((((((........(((((....))))).))))))....	((((((((.(..(((...)))..)...))))))))....	((((((((....(((...)))......)))))))))....	((((((((........(((....))).))))))))....	(((((((..((.(((...))).))....)))))))....	(((((((......(.((((....))))))))))))....
4L8H	/	/	/	/	/	/	/	/	/	/		((((.((((...))))))))	.(((.((((...)))))))).	..((.((((...))))))..	((((.(((.....)))))))	((((..(((...))).))))	.(((.(((.....)))))).	.(((..(((...))).))).	(((..((((...)))).)))	..((.(((.....)))))..	..((..(((...))).))..
4M4O	/	/	/	/	/	/	/	/	/	/		((((((.(((((.........(((((.(..........).)))))..))))).))))))	((((((.(((((.........(((((..............)))))..))))).))))))	((((((.(((((.........(((((....((...))...)))))..))))).))))))	((((((.(((((.........(((((.(..((...)).).)))))..))))).))))))	((((((.(((((.((....((((..((.(.....).))))))..)).))))).))))))	((((((.(((((.((.......((((.(...........).)))))).)))))).))))))	((((((.(((((.((.......((((.............)))))).))))).))))))	((((((.(((((.(.......(((((.(...........).)))))).)))))).))))))	((((((.(((((.(.......(((((..............)))))).))))).))))))	((((((.((((((((....)))((((.(...........).))))...))))).))))))
4M6D	/	/	/	/	/	/	/	/	/	/		((((.....(((((......))))))......))))......	((((..((.(((((......)))))..))..))))......	(((.........(((((.(..........).)))))..)))	(((.........(((((.............)))))..)))	(((.........(((((....((...))...)))))..)))	((((..(..(((((......)))))..)...))))......	((((.....((((........))).....))))......	(((.........(((((.(..((...)).).)))))..)))	.........(..(((((.(..........).)))))..)..	.........(..(((((..............)))))..)..
4OOG	/	/	/	/	/	/	/	/	/	/		(((((((((((((....))))))))))))))..	((((((((((((((......))))))))))))))..	.((((((((((((((....)))))))))))))))...	(((((((((((((.(....).)))))))))))..	(((((((((((.((....)).))))))))))))..	((((((((.((((((....)))))).))))))))..	((((((.((((((((....))))))).))))))..	((.(((((((((((....))))))))))).))..	(.((((((((((((....)))))))))))).)..	..((((((((((((....))))))))))))....
4P3E	/	/	/	/	/	/	/	/	/	/		(((((...(((((((((((.((((((..((((((....))))))..))))).))))......((((...(((.((.((((.(.........))).)).))))...)))).)))))))....)))))	(((((...(((((((((((.(((((..((((((....))))))..)))))).))))......((((...((.(((.((((.(.........))).)).)))))...)))).)))))))....)))))	(((((...(((((((((((.(((((..((((((....))))))..))))).))))((((....))).((...((((((....)))))..))((((...........)))).)))))))....)))))	(((((...(((((((((((.((((((..((((((....))))))..))))).))))(((.....)))((...(((((....)))))..))((((..........)))).)))))))....)))))	.((((...(((((((((((.((((((..((((((....))))))..)))))).))))......((((...(((.((.((((.(.........))).)).)))))...)))).)))))))....)))).	.((((...(((((((((((.((((((..((((((....))))))..)))))).))))......((((...((.(((.((((.(.........))).)).)))))...)))).)))))))....)))).	(((((...(((((((((((.((((((.((((((....))))))..))))).)))......((((...(((.((.((((.........)).)).))))...)))).)))))))....)))))	(((((...(((((((((((.((((((..((((((....)))))))..)))))).))))......((((...((.(((.((((............)).)).)))))...)))).)))))))....)))))	(((((...((((((((((..(((((..((((((....))))))..))))))..)))......((((...(((.((.((((.(.........))).)).)))))...)))).)))))))....)))))	(((((...((((((((((..(((((..((((((....))))))..))))))..)))......((((...((.(((.((((.(.........))).)).)))))...)))).)))))))....)))))

	DNA model											RNA model									
PDB	RNAfold MFE	subopt #2	subopt #3	subopt #4	subopt #5	subopt #6	subopt #7	subopt #8	subopt #9	subopt #10		RNAfold MFE	subopt #2	subopt #3	subopt #4	subopt #5	subopt #6	subopt #7	subopt #8	subopt #9	subopt #10
4P8Z	/	/	/	/	/	/	/	/	/	/		(((((..((((((.(((((((((.......)))))))))....(((((..((.((..(((.((((..((((((.((((...)))).))....))))..))))..)))..)).))..))))))((((.....(((((.(((...((((....))))......))).))))).)))))))))..)))))	(((((..((((((.(((((((((.......))))))))).((((((.(....(((..((((((((..(((((((.(((...)))).))....))))..))))...((((.(((....(((...)))......))).)))).(((((....)))))))))))....)))))))).))))))..)))))	(((((..((((((.((((((((.......))))))))).((((((.((....(((..((((((((..((((((.((((...))))).))....))))..))))...((((.(((....(((...)))....))).)))).(((((....)))))))))))....))))))))).))))))..)))))	(((((..((((((.(((((((((.......))))))))).((((((.(....(((..((((((((..((((((.((((...)))).))....))))..))))).(.((((.(((....(((...))).....))).)))))(((((....))))))))))))....))))))).))))))..)))))	(((((..((((((.(((((((((.......)))))))))).((((((.((....(((..((((((((..((((((.((((...)))).))....))))..)))).(.((((.(((....(((...)))......))).)))(((((....))))))))))))....))))))))).))))))..)))))	(((((..((((((.(((((((((.......)))))))))..(((((.((((.......((((((((..((((((.((((...)))).))....))))..))))....(((.(((....(((...))......))).)))))))......))).))))).((((....)))....))))))..)))))	(((((..((((((.(((((((((........))))))))..(((((.((((........((((.((((..((((((.((((...)))).))....))))..))))...((((.(((....(((...))......))).))))))).......))).)))))..(((....)))....))))))..)))))	(((((..((((((.((((((((.......))))))))....((((((..((.((..(((.((((..((((((.(((......))).))....))))..))))..))..)).)).))))))((((......(((((.(((...((((....))))......))).))))).))))))))))..))))))	(((((..((((((.(((((((((.......)))))))))....((((((..((.((..(((.((((..((((((.(((...)))).))....)))))..))))..)))..)).)).)))))))((((.....(((((.(((...((((....))))......))).))).)))))))))))..)))))	(((((..((((((.(((((((((.......)))))))))((((.((((((....))))).))))...(((((.(.((((...)))).))....))))(((..(((.(((.(((((((.((.(.(((((...))))).).)).)).))..)))))))))))..)))............))))))..)))))
4PDB	/	/	/	/	/	/	/	/	/	/		(((((((((.(..((((....)))).).)))))))))	(((((((((....((((....)))))....)))))))))	.((((((((.(..((((....)))))..).)))))))).	(((((((((..((((((....))))..))))))))))))	((((((((..(..((((....))))..)..))))))))	(((((((((.(((.(((....))))))..)))))))))	(((((((((.((.((((....))))))..)))))))))	(((((((((.(...(((....)))...).)))))))))	(((((((((.....(((....)))).....)))))))))	(((((((((..((((((....))))..))))))))))))
4PMI	/	/	/	/	/	/	/	/	/	/		((((((.....(.(((((....))))).)....))))))	.(((((.....(.(((((....))).)).)....))))).	((((((...((..(((((....))).))...)).))))))	((((((.......(((((....))))).......))))))	((((((...(((.(((((....))).)).).)).))))))	((((((.(((.(.(((......))).).)))...))))))	.(((((...((..(((((....))).))...)).))))).	.(((((.......(((((....))).))......))))).	.(((((...(((.(((((....))).)).).)).))))).	.(((((.(((.(.(((......))).).)))...))))).
4QI2	/	/	/	/	/	/	/	/	/	/		.((((((...))))))).	..((((((...))))))..	.......((((....))))	.((((((.....)))))).	..(((((.....)))))..	........(((....))).	....	...(((((...)))))...	.(((((.......))))).	.((((.((...)).)))).
4QIL	/	/	/	/	/	/	/	/	/	/		.((((((...))))))	..(((((...))))).	.(((((.....)))))	.((((.......))))	.(((.((...)).)))	..((((.....)))).	.((((.(...).))))	/	/	/
4QOZ	/	/	/	/	/	/	/	/	/	/		.....((((((....))))))....	.....(((((......)))))....	......(((((....))))).........	.....(((((.(...))))))....	.....((((.........)))).... .	.....(((......)))......	.....(((.((....)).)))....	/	/	/
4TV0	/	/	/	/	/	/	/	/	/	/		.......((((((....))))))...	.......(((((......)))))...	((.....((((((....)))))).))	((.....(((((......))))).))	........(((((....)))))......	.......(((((.(...))))))... .	.......((((........))))...	.........((((......))))... .	.(.....((((((....)))))).) .	/
4TZV	/	/	/	/	/	/	/	/	/	/		.....((((.......)))).(((((((...)))))))(((..(((((.......)))))))((((....))))	.....((((.......)))).(((((((...))))))(((.((.((((....)))))).)))((((....))))	.....((((.......)))).((((((.....))))))(((..(((((.......)))))))((((....))))	(((..((((.......)))).(((((((...)))))))......(((((.......))))))))((((....))))	.....((((.......)))).(((((((.....))))))(((.((.((((.....)))))).)))((((....))))	(((..((((.......)))).(((((((.....))))).....((((((.......))))))))((((....))))	.....((((.......)))).((((((...)))))))..(..(((((.......)))))..)((((....))))	.....((((.......)))).(((((((...))))))(((...((((.......)))).)))((((....))))	.....(((((....).)))).(((((((...)))))))(((..(((((.......))))))))((((....))))	.....((((.......)))).(((((((...)))))))(((.((.(((......))))).)))((((....))))
4U7U	/	/	/	/	/	/	/	/	/	/		...((((((........))))))......................((((((....))))))	.......((((((.((((((((....)))))))......).))))))(((((....))))).	...((((((.(.....))))))).......................((((((....))))))	...(((((.((......))))))).......................((((((.....))))))	...((((.(((......))))))).........................((((((....))))))	.........((((.((((((((....)))))))......).)))))(((((((....))))))	.......(((((((.((((((((....))))))).....).))))))(((((.....))))).	...(((((........)))))........................((((((....))))))	.........((((.((((((((....)))))))......).)))).((((((....))))))	...((((((........)))))).((((.......))))......((((((....))))))
4WC2	/	/	/	/	/	/	/	/	/	/		(((((((..((((.........)))))).(((((........)))))......(((((.......))))))))))))...	(((((((..((((.........)))).(((((.(....))))))......(((((.......))))))))))))...	(((((((..((((.........))))).((((..........))))......(((((.......)))))))))))))...	(((((((..((((.........)))))).(((((.(...).)))))......(((((.......))))))))))))...	(((((((..(((((......).)))).(((((.......)))))......(((((.......))))))))))))...	((((((...((((.........)))).(((((.......)))))......((((((.......))))).))))))....	(((((((..((((.........))))((((((.......))))).)...(((((.......)))))))))))...	(((((((..((((.........))))(((((((.......)))).)))...(((((.......))))))))))))...	(((((((..((((.........)))).(((((.......)))))(...)(((((.......))))))))))))...	((((((((.((((.........)))))(((((.......)))))......(((((.......))))))))))))...
4WC3	/	/	/	/	/	/	/	/	/	/		(((((((..((((.........)))))).(((((........)))))......(((((.......)))))))))))))....	(((((((..((((.........)))).(((((.(....))))))......(((((.......))))))))))))... .	(((((((..((((.........))))).((((..........))))......(((((.......)))))))))))))....	(((((((..((((.........)))))).(((((.(...).)))))......(((((.......)))))))))))))....	(((((((..(((((......).)))).(((((.......)))))......(((((.......)))))))))))))....	((((((...((((.........)))).(((((.......)))))......((((((.......))))).))))))....	(((((((..((((.........))))((((((.......))))).)...(((((.......))))))))))))....	(((((((..((((.........))))(((((((.......)))).)))...(((((.......)))))))))))))....	(((((((..((((.........)))).(((((.......)))))(...)(((((.......))))))))))))... .	((((((((.((((.........)))))(((((.......)))))......(((((.......))))))))))))... .
4WJ3	/	/	/	/	/	/	/	/	/	/		(((((((..(((((((.(((..((((......)))).)))))))))))).((((((.......)))))))))))))....	((((((((.(((((((.(((..((((......)))).))))))))))..((((.......)))))))))))))....	(((((((..((((.........))))).....((((...........))))((((((.......))))))))))))....	((((((...(((((((.(((..((((......)))).))))))))))).((((((.......))))).)))))))....	(((((((..(((((((.(((..(((........))).)))))))))))).(((((.......)))))))))))))....	.((((((..(((((((.(((..((((......))).)))))))))))).(((((.......))))))))))))....	((((((...(((((((.(((..((((......)))).))))))))..(((((.......))))))))))))....	(((((((..((((.........)))).(((((.......))))....(((((.......)))))))))))))....	((((((((.((((.........))))....(((............))).((((.......))))))))))))....	((((((((..(((((((.(((...(((......)))..))))))))).(((((.......)))))))))))))....
4X0A	/	/	/	/	/	/	/	/	/	/		((((((..((((.........)))).(((((.......)))))......((((((......)))))))))))...	((((((..((((.........)))))).(((((.(....))))))......(((((.......)))))))))))...	((((((..((((.........)))).((((..........))))....(((((.......)))))))))))...	((((((..((((.........)))).(((((.(...).)))))......((((((......)))))))))))...	((((((..(((((......).))))).(((((.......))))).....(((((.......)))))))))))...	(((((...((((.........)))))).(((((.......))))).....((((((.......))))).))))))...	((((((..((((.........))))((((((.......))))).)...((((((.......)))))))))))...	((((((..((((.........))))((((((.......)))).))...(((((.......))))))))))...	((((((..((((.........)))))).(((((.......)))))(...)((((((.......))))))))))...	(((((((.((((.........)))))(((((.......)))))......((((((.......))))))))))...
4X0B	/	/	/	/	/	/	/	/	/	/		((((((((..((((.........)))).((((((.......)))))......(((((.......)))))))))))))...	((((((((..((((.........)))).(((((.(....))))))......(((((.......)))))))))))))....	((((((((..((((..........))))).((((..........)))))......(((((.......))))))))))))))...	((((((((..((((.........)))).(((((.(...).)))))......(((((.......)))))))))))))...	((((((((..(((((......).)))).(((((.......)))))......(((((.......))))))))))))))...	(((((((...((((.........)))).(((((.......))))).....(((((.......))))).)))))))...	((((((((..((((.........))))((((((.......))))).)...((((((.......)))))))))))))...	((((((((..((((.........)))))((((((.......)))).))...(((((.......))))))))))))))...	((((((((..((((.........)))).(((((.......)))))(...)(((((.......))))))))))))))...	(((((((((.((((.........)))))(((((.......))))))......(((((.......))))))))))))))...
4X4O	/	/	/	/	/	/	/	/	/	/		((((((((((((.......))))))))...)))	((.(((((((((.......)))))))))).))...	...(((((((((.......))))))))))......	((((((((((((.(...).))))))))))...)))	.((((((((((((.......))))))))...)).	(((((((((((((....).))))))))...)))	(..(((((((((.......))))))))..)...	(((.((((((((.......))))))))....)))	(((((((((((((.........))))))))...)))	((.((((((((((.(...).)))))))))).))...
4X4P	/	/	/	/	/	/	/	/	/	/		((((((((((((.......)))))))))...)))..	(((((((((((((....).))))))))...)))..	((((((((((((.(...).))))))))...)))..	(((.((((((((.......))))))))....)))..	((.((((((((((.......))))))))).)).....	...((((((((((.......))))))))))........	((((((((((((.........)))))))...)))..	((.((((((((((.......))))))))).....))..	.(((((((((((.......)))))))))...))...	(..((((((((((.......)))))))))..).....
4YCO	/	/	/	/	/	/	/	/	/	/		((((((..((((.........)))))).(((((((...)))))))......(((((.......)))))))))))..	(((((((..((((.........)))).(((((((.....))))))......(((((.......)))))))))))..	((((((((.((((.........))))).(((((((...)))))))).....((((.......)))))))))))))..	((((((...((((.........)))))).(((((((...)))))))......(((((.......))))).))))))..	((((((((.((((.........)))).((((((.....))))))......((((.......))))))))))))..	((((((...((((((((((.((.((((((((((...))))))).))).))....))))))..)))))))))..	((((((...((((((((((.((.((((((((((...)))))))).))).))....))))))..)))))))))..	((((((...((((.........)))).((((((.....))))))......(((((.......))))).)))))))..	((((((...(((((((((((..(((((((((((...))))))).))..)))...))))))..)))))))))..	((((((((.((((.........))))).(((((((...)))))))...........(((....))))))))))))..
4YVI	/	/	/	/	/	/	/	/	/	/		((((((((((((((.......))))))))((((((.(((((..(.....)..))))))))))))....))))))).	(((((((..((((.......))))(((.((((((.(((((..(.....)..)))))))))).))))))))))).	.((((((((((((.......)))))))((((((.(((((..(....)..)))))))))))....))))))..	((((((((((((((.......)))))))((((((.((((((.......).)))))))))).....))))))).	((((((((((((((.......))))))).((((((.(((((..(....)..)))))))))))...)))))))).	(((((((.(((((.......))))))(((((.......)))))....(((((.......))))))))))))))).	(((((((.(((((.......)))))..((((((.(((((..(....)..))))))))))))...))))))).	((((((((((((((.......)))))))((((((.((((((.(....)).)))))))))))....))))))).	(((((((.(((((.......)))))((((((....).)))))....((((((.......)))))))))))).	.((((((..((((.......))))((.((((((.(((((..(.....)..)))))))))))).)))))))))..
4YVJ	/	/	/	/	/	/	/	/	/	/		(((((((.(((((.......)))))((((((....).)))))....((((((......))))))))))))).	(((((((..........((.(((((..(((((.(((.(((....))).))))))))).))))))))))))))).	((((((((((((...)))).....(((((((((....).)))))))..(((((.......))))))))))))))).	(((((((.(((((.......)))))(((((.......)))))....((((((......)))))))))))))).	(((((((.(((...)))......((((((((....).)))))))..(((((.......)))))))))))).	.((((((.(((((.......)))))((((((....).)))))....(((((.......)))))))))))..	(((((((..((((.......))))((((((((....).))))))...((((((.......)))))))))))))).	(((((((...((((....)))))).((((((((....).)))))))..(((((.......)))))))))))).	.((((((..........((.(((((..(((((.(((.(((....))).)))))))))).))))))))))))..	(((((((.....(((....)))..((((((((....).)))))))..((((.......))))))))))).
4YVK	/	/	/	/	/	/	/	/	/	/		(((((((..........((.(((((..(((((.(((.(((....))).)))))))))).))))))))))))))))).	.((((((..........((.(((((..(((((.(((.(((....))).))))))))).))))))))))))))..	(((((((..........((.(((((..(((((.(((.(.((...))).)))))))))).))))))))))))))).	(((((((..((...))((.(((((..(((((.(((.(((....))).)))))))))).)))))))))))))))).	(((((((..........((.(((((..(((((.(((.((......))).))))))).))))))))))))))).	(((((((..........((..((((..(((((.(((.(((....))).))))))).))))).))))))))).	(((((((.(((((.......)))))((((((....).)))))....(((((.......))))))))))))).	(((((((((((...)))).(((((..(((((.(((.(((....))).))))))).))))..))))))).	(((((((.(((((.......)))))((((((.......)))))....(((((.......))))))))))))).	(((((((.(((...)))..(((((..(((((.(((.(((....))).)))))))).))))..))))))).
4YYE	/	/	/	/	/	/	/	/	/	/		((((((((((((.........)))))..(.(((((....))))).)...((((((....)))))))))))))))..	((((((((((((.........)))))(..(.(((((....))))).)..)(((((....)))))))))))))))..	((((((((((.....((((.....((..(.(((((....))))).)..))....))))....)))))))))))..	((((((((((((.........))))...(.(((((....))))).)...((((......)))))))))))))..	((((((((((((((.........)))).((((..........))))......(((((....)))))))))))))))...	((((((((((((.........))))...(.(((((....))))).)...(((.........))))))))))))..	(((((((.((((.........)))))...(.(((((....))))).)...((((....))))).)))))))))..	((((((((((((((.........))))(..(.(((((....))))).)..)((((......))))))))))))))...	((((((((((((.........)))).....((((....))))).....(((((....)))))))))))))))..	((((((((((((.........))))(..(.(((((....))))).)..)(((.........))))))))))))..
4Z0C	/	/	/	/	/	/	/	/	/	/		..((......)).	....	..((.....))..	..((.......))	..(......)...	.(.....).....	..(........).	...(......)..	..(.......)..	...(.......).
4Z7L	/	/	/	/	/	/	/	/	/	/		....	((..........))	(........)....	.(..........).	......(....)..	/	/	/	/	/
4ZLD	/	/	/	/	/	/	/	/	/	/		(((((((...)))))))	.((((((...))))))).	..(((((...)))))..	((((((.....))))))	...((((...))))...	.(((((.....))))).	....	(((((.......)))))	..((((.....))))..	(.(((((...))))).)

	DNA model											RNA model									
PDB	RNAfold MFE	subopt #2	subopt #3	subopt #4	subopt #5	subopt #6	subopt #7	subopt #8	subopt #9	subopt #10		RNAfold MFE	subopt #2	subopt #3	subopt #4	subopt #5	subopt #6	subopt #7	subopt #8	subopt #9	subopt #10
4ZT0	/	/	/	/	/	/	/	/	/	/		((((((((((((((((.((((....))))...)))))))...))..)))..))))).((((....))))..	((((((((..((((((..((((....))))..........))))))..)))..))))).((((....))))..	((((.((((((((((((.((((....))))...)))))))...))..)))))))....((((....))))..	((((.(((..((((((..((((....))))..........))))))..))))))....((((....))))..	((((.((((((((((((.((((....))))...)))))))...)).)))...)))).((((....))))..	((((.(((..((((((..((((....))))..........))))))..)))...)))).((((....))))..	((((((((((.((((((.((((....))))...))))))...).))..)))..)))))).((((....))))..	((((((((((.((((((.((((....))))...))))...)).)).)))..))))).((((....))))..	((((((((((((((((..((((....))))....))))))...))..)))..))))).((((....))))..	(((((((((((((((((.((((....))))...)))))))...))..)))..)))))..((((....)))...
5A18	/	/	/	/	/	/	/	/	/	/		((((((((((..(.....)..))).))))))))	((((((((((..(.....)..)).))))))))	((((((((...((.......)).))))))))	((((((((..(..........)..))))))))	((((((((......(((....))))))))))))	(((((((....((.......))...)))))))	((((((((((...........))).)))))))	((((((((((...........)).))))))))	((((((((....(((.....))).))))))))	((((((((..((.......))))).)))))))
5CCB	/	/	/	/	/	/	/	/	/	/		.((((((((.((((........)))))))).((((((((...))))))))(((((.......)))))..)))))....	.(((((((..((((........))))....((((((((...))))))))(((((.......))))))))))))....	.((((((((.((((........))))))).(((((((.....))))))((((((.......))))).)))))....	.(((((((..((((........))))....(((((((.....)))))))(((((.......))))))))))))....	.((((((...((((........))))....((((((((...))))))))((((((.......))))).))))))....	(((((((((.((((........)))))).((((((((...))))))))(((((.......)))))..))))).)..	.((((((((.((((........)))))....((((((((...)))))))).((((.......))))))))))))....	((((((((..((((........))))....((((((((...)))))))(((((.......))))))))))).)..	.((((((((((((........))))....((((((((...)))))))).(((.......))))))))))))....	.((((((...((((........))))....(((((((.....)))))))(((((.......))))).)))))....
5D6G	/	/	/	/	/	/	/	/	/	/		((((......(((((((((.((((.......))))).))))....((((........)))).))))))..))))	((((..(((...(((((((.(((((.......))))).))))....((((........)))).))))))).))))	((((....(.((((((((.(((((.......))))).))))....((((........)))).)))))).)))))	((((..(...((((((((.(((((.......))))).))))....((((........)))).)))))).))))	((((..((...((((((((.((((((.......))))).))))....((((........)))).)))))).))))	((((..(((.(.(((((((.((((((.......))))).)))))...((((........)))).)))))).))))	((((......(((((.(((.(((((.......))))).)))......((((........)))).)))))..))))	((((..(((...(((.(((.((((((.......))))).)))....((((........)))).)))))).))))	((((....(.(((((.(((.(((((.......))))).)))....((((........)))).))))).)))))	((((..(((...(((((((.(((((.......))))).)))))...((((........)))).)).))).))))
5F5F	/	/	/	/	/	/	/	/	/	/		(((((.(......).)))))	.((((.(......).)))).	..(((.(......).)))..	...((.(......).))...	((((..(......)..))))	(((((.(.......))))))	(((((..........)))))	.(((..(......)..))).	.((((.(.......))))).	.((((..........)))).
5F5H	/	/	/	/	/	/	/	/	/	/		((((((......)))).)))	.((((((......)))).)).	...((((......))))....	((.((((......))))..))	(..((((......))))..).	(((.(((......)))..)))	(((((((.(...))))).)))	.(.((((......))))..).	((((((........))).)))	((((.((......)).).)))
5F9F	/	/	/	/	/	/	/	/	/	/		((((((((((....))))))))))	(((((((((......)))))))))	.(((((((((....)))))))))).	..((((((((....))))))))..	((((((((........))))))))	(((((((.((....)).)))))))	((.((((((((....))))))).))	((((((.(((....))).))))))	.((((((((......)))))))).	((((((((.(....).))))))))
5FJ4	/	/	/	/	/	/	/	/	/	/		..((.....)).((((((....))))))).......	....(((..(..((((((....))))))).)..)))	....(((.....((((((....))))))....)))	...((((.((....)).))))...............	...((....)).((((((....)))))).......	....(((...(.((((((....))))))).)..)))	...((((.((....)).))))(((.......))).	..((.....))..(((((....)))))........	..........(.((((((....)))))).).....	............((((((....)))))).......
5FMZ	/	/	/	/	/	/	/	/	/	/		....	.((....))...	.(......)...	....(...)...	(....)......	..(......)..	.....(....).	..(....)....	/	/
5HR6	/	/	/	/	/	/	/	/	/	/		(((((..((((.........))))((((((.......))))))...(((((.......))))))))))	(((((.......(((.(((.(...(((((((.......))))))..).))).)))..........)))))	(((((.......(((.(((..(..((((((.......))))))..).))).)))..........)))))	(((((..((((.(......).))))((((((.......))))))...(((((.......))))))))))	(((((..(....(((.(((.(...(((((((.......))))))..).))).)))....)....)))))	(((((.......(((.(((.....(((((((.......))))))....)).)))..........)))))	(((((..(....(((.(((.(...((((((.......))))))..).))).)))......)..)))))	(((((..(....(((.(((..(..((((((.......))))))..).))).)))....)....)))))	(((((..(....(((.(((..(..((((((.......))))))..).))).)))......)..)))))	((((...((((.........))))((((((.......))))))...(((((.......))))).))))
5HR7	/	/	/	/	/	/	/	/	/	/		(((((..((((.........))))((((((.......))))))...(((((.......))))))))))..	(((((.......(((.(((.(...(((((((.......))))))..).))).)))..........)))))..	(((((.......(((.(((..(..((((((.......))))))..).))).)))..........)))))..	(((((..((((.(......).))))((((((.......))))))...(((((.......))))))))))..	(((((..(....(((.(((.(...(((((((.......))))))..).))).)))....)....)))))..	(((((.......(((.(((.....(((((((.......))))))....)).)))..........)))))..	(((((..(....(((.(((.(...((((((.......))))))..).))).)))......)..)))))..	(((((..(....(((.(((..(..((((((.......))))))..).))).)))....)....)))))..	(((((..(....(((.(((..(..((((((.......))))))..).))).)))......)..)))))..	((((...((((.........))))((((((.......))))))...(((((.......))))).))))..
5ID6	/	/	/	/	/	/	/	/	/	/		....(((((.....)))))..	.....((((.....))))...	...(.((((.....)))))..	.(..(((((.....)))))).	......(((.....)))....	....((((.......))))..	....((.((.....)).))..	.((..((((.....)))))).	....(((.(.....).)))..	....
5IEM	/	/	/	/	/	/	/	/	/	/		((((((((((.(((((..(((((((....))).)))))))..))...))))))))))	((((((((((...(((..(((((((....))).))))....)))...))))))))))	(((((((((((((((((..(((((((....))).)))))))..)).).))))))))))	((((((((((.((((...((((((((....))).)))).))..))...))))))))))	((((((((((.((.....(((((((....))).))))......))...))))))))))	((((((((((...((((((((((((....))).)))).)).)))...))))))))))	((((((((((...((((.(((((((....))).)))))..).)))...))))))))))	((((((((((...((((.(((((((....))).)))).)..)))...))))))))))	((((((((((.(((((..(((((((....)).))))))))..))...))))))))))	((((((((((...((...(((((((....))).)))).....))...))))))))))
5IWA	/	/	/	/	/	/	/	/	/	/		/	/	/	/	/	/	/	/	/	/
5KH8	/	/	/	/	/	/	/	/	/	/		(((((......)))))......((((....)))).............	((((........))))......((((....)))).............	(((((......)))))....(.((((....)))))............	(((((......)))))......((((....))))....(....)...	(((((......))))).((...((((....))))...))........	(((((......))))).((...((((....))))))...........	(((((......)))))......((((....))))((........)).	(((((......)))))......(((....))).............	(((((......)))))..(...((((....))))...).........	(((((......)))))....(.((((....))))...).........
5KMZ	/	/	/	/	/	/	/	/	/	/		.......((((.(((........))).))))	(((((.........)))))..........	.......(((.(((........))).))).	.((((.........))))............	.......((((..((........))..))))	.......(((..(((........)))..)))	.......(((.....)))...........	.......(((..((........))..))).	.......((..(((........)))..)).	(((((.........)))))((......))..
5KQE	/	/	/	/	/	/	/	/	/	/		(((((((......(((((((....))))))))))))))	.((((((......((((((....)))))))))))).	((((((.......((((((....))))))).))))))	((((((.....(.((((((....))))))))))))))	(((((((......((.(((....))).)))))))))	(((((((......(((((......)))))))))))))	(((((((.......(((((....))))).)))))))	((((((........((((((....))))))..)))))	((.((((......((((((....))))))))))).))	(((((((......(((.((....)).))))))))))
5L1Z	/	/	/	/	/	/	/	/	/	/		.....((((......))))..	.....(((((...).))))..	((...((((......))))))	.....((((.(...)))))..	.....(((.((...)))))..	.....(((........)))..	......(((......)))...	((...(((((...).))))))	.....(((.(.....))))..	.(...((((......))))).
5LM7	/	/	/	/	/	/	/	/	/	/		.............(((((.....)))))	....(((....)))(((((.....)))))	..............((((.....))))).	.............((((.......))))	.....((....)).(((((.....)))))	...((.......))(((((.....)))))	....((......))(((((.....)))))	.............(((((....).))))	..(((.......)))((((.....)))).	...(........).(((((.....)))))
5LSN	/	/	/	/	/	/	/	/	/	/		((.((((((((((...)))))))))))).))	((.(((((((((.....))))))))))).))	.(.((((((((((...))))))))))).).	((.((((((((.......)))))))).))	.(.((((((((((.....)))))))))).).	((..(((((((((...)))))))))))..))	...((((((((((...))))))))))...	((.((((((((.(....)))))))))).))	((..((((((((.....)))))))))..))	...((((((((((.....))))))))))...
5M0I	/	/	/	/	/	/	/	/	/	/		(((((.((..((.....)))))))))...	(((((((.........))...)))))....	(((((.((............)))))))...	(((((.((...(.....).)))))))....	(((((.....((.....))..)))))...	((((..((..((.....)))).))))...	(((((...............)))))...	(((((.((..(.....)..)))))))...	(((......)))................	(((((..(..((.....))).)))))....
5N5C	/	/	/	/	/	/	/	/	/	/		(((((((((...)))))))))	(((((((.....)))))))	.(((((((...))))))).	..((((((...))))))..	.((((((.....)))))).	..(((((.....)))))..	((((((.......))))))	...(((((...)))))...	.(((((.......))))).	..((((.......))))..
5NG6	/	/	/	/	/	/	/	/	/	/		....(((((....)))))......	.....((((....)))).......	(((((((((....)))))..))))	.(((((((((....)))))..))).	..(.(((((....))))).)....	(((.(((((....)))))...)))	(((((((((....))))..)))))	.((.(((((....)))))...)).	..((((((....)))))..))..	...(((((((....))))).)....
5TF6	/	/	/	/	/	/	/	/	/	/		(((((.((((........))))..))))))(((.((((.(((.....)))..))))).))).............	(((((.((((........))))..))))).((.((((.(((.....))))..)))).))............	(((((.((((........))))..)))))....((((.(((.....)))..)))).(((((.....))))))	(((((.((((........))))..))))))....((((.(((.....)))..)))))...............	((((..((((........))))...))))(((.((((.(((.....)))..)))).)))............	(((((.((((.(....).)))).)))))(((.((((.(((.....)))..)))).)))............	(((((.((((........))))..))))).((((.....))))....((.....))(((((.....)))))	(((((.((((........))))...)))))..(.((((.(((.....)))..)))).).............	(((((.((((........))))..))))).((((.....))))...............(((((.....)))))	((((..((((........))))...))))).((.((((.(((.....))).)))).)).............
5UDZ	/	/	/	/	/	/	/	/	/	/		(((((((.....)))))))....	.((((((.....))))))......	(((((((.......))))))).....	.(((((.......)))))......	(((((.........)))))....	((((((((....)).)))))....	(((((((....).))))))....	(((((.(.....).)))))....	(((..((....))..)))......	(((((.(......))))))....
5UF3	/	/	/	/	/	/	/	/	/	/		(((((((.((....)))))))))	((((((.(((....)))))))))	(((((((.(.....).))))))))	(((((..(((....))).)))))	.((((((.((....)))))))).	.(((((.(((....)))))))).	((((((..((....)).))))))	(((((((.(......))))))))	((((((.((......))))))))	(((((((.........)))))))
5UZT	/	/	/	/	/	/	/	/	/	/		(((((((.((((.((...)))))))))))))	(((((((.(((.(((...))))))))))))))	(((((((.((((........)))))))))))	(((((((.((..(((...))).)))))))))	(((((((.((((.(.....))))))))))))	(((((((.(((.((.....))))))))))))	(((((((.((..((.....)).)))))))))	(((((((.(((........)).).)))))))	(((((((.((((.(....).))))))))))	(((((((.....(((...)))...))))))))
5V17	/	/	/	/	/	/	/	/	/	/		((((((.....(((((((((......)))))))))))))))	(((((((.....((((((((......)))))))))))))))	.(((((.....((((((((......)))))))))))))).	((((((......((((((((......))))))).))))))	(((((......((((((((((......)))))))))).)))))	.((((((.....((((((((......)))))))))))))).	(((((.(....((((((((......)))))))))))))))	((((((.....((((((((........)))))))))))))	((((((.....((((.((((......)))).))))))))))	((((((.....(((.(((((......))))).)))))))))
5V6X	/	/	/	/	/	/	/	/	/	/		(((((((.((((.....))))((((((((.......)))))))..(((((.......)))))))))))))..	(((((((.((((.....))))((((((.........))))))..(((((.......)))))))))))))..	(((((((((((((.....))))(((((((.......)))))))....((((.......))))))))))))..	(((((((.((((.....))))(((((((.(...).)))))))..(((((.......)))))))))))))..	(((((((.((((.....))))(((((((.(....))))))))..(((((.......)))))))))))))..	(((((((((((((.....))))((((((.........))))))...((((.......)))))))))))))..	(((((((.((((.....))))(((((((.......)))))))...((((.......)))).)))))))..	((((((((((((.....)))))((((((.......))))))...(((((.......))))))))))))..	((((((..((((.....))))(((((((.......)))))))..((((((.......))))).))))))..	((((((((((((.....))))((((((((.(...).)))))))...((((.......))))))))))))..

	DNA model											RNA model									
PDB	RNAfold MFE	subopt #2	subopt #3	subopt #4	subopt #5	subopt #6	subopt #7	subopt #8	subopt #9	subopt #10		RNAfold MFE	subopt #2	subopt #3	subopt #4	subopt #5	subopt #6	subopt #7	subopt #8	subopt #9	subopt #10
5VW1	/	/	/	/	/	/	/	/	/	/		..........((((((.((((....))))...)))))).................((((....))))...	.........((((((..((((....))))..........))))))..........((((....))))...	..........(((((((.((((....))))...)))))))..((((......)))....((((....)))))...	..........((((((.((((....))))...)))))))..((.....))......((((....))))...	...................((((....))))(((((...((((.(......).)))).)))))..........	..........((((((.((((....))))...)))))))...((.....))......((((....))))...	.................((((....))))(((((...((((((.....)).))))).)))))...........	.........((((....))))......((((((...((((.(.......).)))).))))))...........	..........((((((..((((....))))....))))))...........((((....))))...	..((......(((((((.((((....))))...)))))))...))......((((....))))...
5W1H	/	/	/	/	/	/	/	/	/	/		......((((........))))......(((.......)))	......((((........))))..............	...((.((((........)))).))...(((.......)))	...((.((((........)))).))..............	......((((........))))......((.......)).	......((((........)))).(((......)))......	...(..((((........))))..)...(((.......)))	...(..((((........))))..)...............	....(.((((........)))).)....(((.......)))	....(.((((........)))).)...............
5WLH	/	/	/	/	/	/	/	/	/	/		.......((((........)))).....(((.......)))	.......((((........))))..............	....((.((((........)))).))...(((.......)))	....((.((((........)))).))..............	(...((.((((........)))).))...)...........	.......((((........))))......((.......)).	.......((((........)))).(((.......)))......	(......((((........)))).......)...........	....(..((((........))))..)...(((.......)))	....(..((((........))))..)..............
5WQ1	/	/	/	/	/	/	/	/	/	/		(((((((((.....)))))))))	.((((((((.....)))))))).	((((((((.......))))))))	((((((((....).)))))))	(((((.(((.....))).)))))	(((((((.(.....).)))))))	((((.((((.....)))).))))	(((((((.........)))))))	/	/
5WT1	/	/	/	/	/	/	/	/	/	/		((((..((((.........)))).(((((.......)))))......(((((......)))))))))	((((..((((..(....)..)))).(((((.......))))).....(((((.......)))))))))	((((..((((.........)))).((((((.....).))))).....(((((.......)))))))))	((((..((((..(....)..)))).(((((.....).))))).....(((((......)))))))))	((((..((((.........)))).(((((.(.....)))))).....((((((.......)))))))))	((((..((((..(....)..)))).(((((.(.....))))))......((((.......))))))))))	.(((....((((.((((.......)))).))))....)))......(((((((.....)))))).	((....))((((.((((.......)))).))))......((((....(((((.....)))))))))).	((((..((((.........)))).(((((.......))))).....(((((....))))))).))))	((((..((((.........)))).((((.........))))......((((((......)))))))))
5WT3	/	/	/	/	/	/	/	/	/	/		(((((((..((((.........)))).(((((.......))))).....((((((.......)))))))))))))	(((((((..((((..(....)..)))).(((((.......))))).....(((((.......))))))))))))	(((((((..((((.........))).(((((((.....).))))).....(((((.......))))))))))))	(((((((..((((..(....)..)))).(((((.....).))))).....((((.......)))))))))))	(((((((..((((.........)))).(((((.(.....))))))....(((((.......))))))))))))	(((((((..((((..(....)..)))).(((((.(.....)))))).....(((((.......)))))))))))	(((((((..((((.........)))).((((((.......))))).....((((((.....))))))).)))))))	(((((((..((((.........)))).((((.........)))).....(((((.......))))))))))))	(((((((..((((..(....)..)))).(((((.......))))).....((((((....)))))).)))))))	(((((((..((((..(....)..)))).((((.........)))).....(((((.......))))))))))))
5X6B	/	/	/	/	/	/	/	/	/	/		((((((..((((((....)))))).(((((.......))))).....(((((.......)))))))))))))....	(((((((..((((((....)))))).((((((.....).))))).....((((((.......))))))))))))....	(((((((..((((((....))))).(((((.(.....))))))......(((((.......))))))))))))....	((((((..((((((....)))))).((((.........)))).....(((((.......)))))))))))))....	(((((((..((((((....)))))).(((((.(....).))))).....(((((.......)))))))))))).....	(((((((..(((((.....))))).(((((.......))))).....((((.......)))))))))))))....	(((((((..((((((.....)))))).((((((.....).))))).....(((((.....)))))))))))))....	(((((((..((((((....))))).(((((.......))))).....(((((.(....).)))))))))))).....	(((((((((((((....))))).....(((((.......))))).....((((.......)))))))))))))....	(((((((..((..(((((.(..((((....))))..).)).))).))((((.......))))))))))))).....
5XBL	/	/	/	/	/	/	/	/	/	/		.(((.((((((((((((.((((....))))...)))))))...)))))..)))).....((((....))))(((((((..))))))).	.(((..(((((((((((.((((...))))...)))))))...))))..))).......((((....))))((((((...))))))).	.(((.(((((((((((.(((....))))...)))))))...)))))..))).....((((....)))).((((((...))))))..	.(((.(((((((((((.((((...))))...)))))))...)))))..)).....((((....))))((((((....)))))).	((.(((.(((((((((.((((.....))))...)))))))...)))))).)).......((((....))))(((((((...))))))).	((.((.((((((((((.((((....))))...)))))))...)))))).)).......((((....))))(((((((...))))))).	.(((.(((((.((((((.((((....))))...)))))....).))))..))))......((((....))))(((((((...)))))).	.(((.(((((.((((((.((((....))))...)))))...)).)))))..)))......((((....))))(((((((...))))))).	.(.(((.(((((((((((.((((....))))...)))))))...)))))).........((((....))))((((((((...))))))).	.(.((.(((((((((((.((((....))))...)))))))...)))))).........((((....))))(((((((...))))))).
5Y58	/	/	/	/	/	/	/	/	/	/		(((((..(((((.......))))))))))).	(((((..(((((.......))))))))))).	((((((..(((.......))))))))))).	(((((...((((.......)))).)))))).	((((((...(((.......))).)))))).	((((...(((((.......))))).)))).	((((((((((....)))......))))))).	((((.(.(((((.......)))))))))).	(((((((..............)))))))).	((((((..(((.........))))))))).
6AAS	/	/	/	/	/	/	/	/	/	/		((((((((((((....)))))))))))))	.((((((((((((....))))))))))).	(((((((((((......))))))))))))	(((.((((((((....)))))))))).)))	((((.((((((((....))))))))).))))	(((((((((.((....)).)))))))))	(((((((.(((....))).))))))))	((.(((((((((....))))))))).))	.(((((((((((......))))))))))).	((((((.(((((....))))).))))))
6B14	/	/	/	/	/	/	/	/	/	/		((((((((((..((((.(..(.((...((((((((.....)))))))..)).....)).)...).))))...))))))))))	((((((((((..((((....(.((...((((((((.....)))))))...)).....)).)......))))...))))))))))	((((((((((..((((.(..(.((.(.((((((((.....))))))))..)).)...)).)...).)))...))))))))))	((((((((((..((((....(.((.(.((((((((.....)))))))..)).)...)).)......))))...))))))))))	((((((((((..((((.(....((((.((((((((.....))))))..)).)....))...)).))))...))))))))))	((((((((((..((((.(....((...((((((((.....))))))))..)).....))......).))))...))))))))))	((((((((((..((((.(....((.(.(((((((((.....))))))..)).)...)).....).))))...))))))))))	((((((((((..((((.(....((..((((((((((.....)))))))..)).....)))...).))))...))))))))))	((((((((((..........(((....))((((((.....))))))(((.(..((((....))))).)))))))))))))	((((((((((...((((.(..(.((..(((((((((.....))))))))..)).....)).)...).))))....))))))))
6B3K	/	/	/	/	/	/	/	/	/	/		((((((((((.........((..(((((((.........))))))..))((((..(..((((....))))).))))))))))))))	((((((((((.((((((((.....((((((.........))))))))))))).....((((....))))......))))))))))	((((((((((.........((..(((((((.....).))))))..))((((.(..((((....)))).).))))))))))))))	((((((((((.((((((((.....((((((.....).)))))))))))))).....((((....)))).......))))))))))	((((((((((..........((..(((((((.........))))))..))((((.(..((((....))))).))).)))))))))	((((((((((.........((..(((((((.........))))))..))(((....((((....))))..)))))))))))))	((((((((((..(((((((.....(((((.........))))))))))))).....((((....))))........))))))))))	((((((((((.........((..(((((((.....).))))))..))((((.(..((((....))))).)))).))))))))))	((((((((((.........((..((((((.....).))))))..))((((....((((....)))).).))))))))))))	((((((((((....((((.....(((((((.........))))))))))((.(..((((....)))))).))))))))))
6BHJ	/	/	/	/	/	/	/	/	/	/		..((((((((((((((...)))))))))))))))	...((((((((((((((...)))))))))))))).	..(((((((((((((((.....)))))))))))))	..((((((((.(((((((...))))))).)))))))	..((((((((.((((((...)))))).)))))))))	..(((((((((((((.((...)).))))))))))))	...(((((((((((((.....)))))))))))).	/	/	/
6CYT	/	/	/	/	/	/	/	/	/	/		.....(((......)))	......((......)).	....	.(((.......)))...	.(((.(...).)))...	.....((((...).)))	..((.......))....	..((......)).....	......(((...).)).	..((.(...).))....
6D12	/	/	/	/	/	/	/	/	/	/		(((((((.....)))))))(((((((.....)))))))	(((((((.....))))))).(((((.....)))))).	(((((((((((.((((.....)))))))))))).))))))	((((((((((((.((((....))))))))).)))))))	(((((((((((.(((((....)))))))))).))))))	(((((((((((.(((((....)))))))))).)))))))	(((((((.....(((((....))))).....)))))))	(((((((.....)))))))..(((((.....)))))..	.((((((.....)))))).(((((((.....)))))))	((((((((.....).))))))(((((((.....)))))))
6DB8	/	/	/	/	/	/	/	/	/	/		(((((((((...........(((.....)))(((((.........)))))..)))))))))	(((((((((...........((((.....))))((((.........))))...)))))))))	(((((((((.((.......(((.....))))((((.........))))).)))))))))	(((((((((..........(((((..)))))(((.........)))....))))))))	(((((((((...........(((.....))).((((.........))))...)))))))))	(((((((((.......((.((.....)).))(((((.........)))))..)))))))))	(((((((((.(..........(((.....)))(((((.........)))))).))))))))	(((((((((......(((.((((.....)))))))(((.........)))..)))))))))	(((((((((.......((.(((((...))))))(((.........))).)))))))))	(((((((((.((......(((((...)))))(((.........))).)).)))))))))
6DTD	/	/	/	/	/	/	/	/	/	/		.((((....(((((((.....))).))))....))))	.((((....(((((((.....)).)))))....))))	.((((....(((((((.....).)))))....))))	.((((....((((((.........)))))))....))))	.((((..(.(((((((.....)).)))).)..))))	.((((..(.(((((((.....)).))))).)..))))	.((((..(.(((((((.....).))))).)..))))	((((.....((((((.....)).)))).))))..	((((.....(((((((.....)).))))))..))))..	((((.....(((((((.....).)))))..))))..
6DU4	/	/	/	/	/	/	/	/	/	/		(((((((...............)))))))	(((((((.....((....))..)))))))	(((((((.....(....)....))))))	(((((((...(....)......))))))	(((((((.....(......)..)))))))	((((((((((...))).......)))))))	.(((((((...............))))))).	(((((((.(...........).)))))))	(((((((....(...)......)))))))	.(((((((.....((....))..)))))).
6DU5	/	/	/	/	/	/	/	/	/	/		((((..(.((...)).)..))))..	((((..(((...)))....))))..	((((....((...))....))))..	((((..((......))....))))..	((((...............))))..	((((..(.(.....).)..))))..	((((..(.........)..))))..	..(((((.............)))))	(((...(.((...)).)...)))..	((((....(......)...))))..
6F4G	/	/	/	/	/	/	/	/	/	/		..((((((...........))))))	((.((.((((....)))).)).)).	..((((((..((...))..)))))))	..((((((...(......).))))))	..((((((..(......)..))))))	..((((((((....))...))))))	..((((((.(.......)..))))))	..(((((.............)))))	..((((((...(......)))))))	((.((.(((......))).)).)).
6F4H	/	/	/	/	/	/	/	/	/	/		((((((..........))))))	.(((((...........))))).	(((((((........).))))))	((((((...(...)..))))))	((((((...(.....).))))))	((((((...(.....)))))))	(((((............)))))	.((((((.......).))))).	.(((((...(...)..))))).	/
6FQ3	/	/	/	/	/	/	/	/	/	/		(((((...)))))	((((.....))))	.((((...)))).	.(((.....))).	(((.......)))	....	((...))......	((.((...)).))	/	/
6FQL	/	/	/	/	/	/	/	/	/	/		(((((...)))))	.((((...)))).	((((.....))))	.(((.....))).	(((.......)))	.((.......)).	....	..(((...)))..	(((.(...).)))	.((.(...).)).
6GBM	/	/	/	/	/	/	/	/	/	/		(((((((.........)))))))	((((((...........))))))	(((((((.(......))))))))	((((((.((.......)))))))	.((((((.........)))))).	(((((((.(....)..)))))))	(((((.............)))))	((((((.(.........)))))))	((((((((...(....))))))))	(((((..((.......)).)))))
6HYK	/	/	/	/	/	/	/	/	/	/		(((((......((((....))))...)))))	(((((....(.((((....))))).)))))	(((((..(.(.((((....))))).))))))	(((((..(...((((....)))).).)))))	.((((......((((....)))).)..)))).	(((((..((..((((....)))))).)))))	(((((...(..((((....)))))..)))))	(((((..(...((((....))))..))))))	.((((....(.((((....))))).)))).	(((((.(....((((....))))).)))))
6IV8	/	/	/	/	/	/	/	/	/	/		.(((((((((....)))))))))..............................	.((((((((......))))))))..............................	..((((((((....))))))))...............................	..(((((((......)))))))..............................	.(((((((((....))))))))).......((...))............	.(((((((((....)))))))))............(........)......	.(((((((((....)))))))))........(....)..........	.(((((((((....))))))))).......(.....)...............	.((((((((......)))))))).......((...))..............	.(((((((((....)))))))))(...........)...............
6IV9	/	/	/	/	/	/	/	/	/	/		.(((((((((....))))))))).............................	.((((((((......)))))))).............................	..((((((((....))))))))...............................	..(((((((......))))))).............................	.(((((((((....))))))))).......((...))...........	.(((((((((....)))))))))............(........).....	.(((((((((....)))))))))........(....).........	.(((((((((....))))))))).......(.....).............	.((((((((......)))))))).......((...))..............	.(((((((((....)))))))))(...........)...............

	DNA model											RNA model									
PDB	RNAfold MFE	subopt #2	subopt #3	subopt #4	subopt #5	subopt #6	subopt #7	subopt #8	subopt #9	subopt #10		RNAfold MFE	subopt #2	subopt #3	subopt #4	subopt #5	subopt #6	subopt #7	subopt #8	subopt #9	subopt #10
6JXM	/	/	/	/	/	/	/	/	/	/		((((((..((((.......)))).((((((((....(((......))).....))))))))(((.(.((....)).).)))..)))))))....	((((((..((((.......)))).(((((((....(((......))).....))))))))).....(((((.......)))))))))))....	((((((..((((.......)))).((((((((.....(((......)))....))))))))(((.(.((....)).).)))..))))))....	((((((..((((.......)))).((((((((.....(((......)))....))))))))).....(((((.......)))))))))))))....	((((((..((((.......))).((((((((....(((......)))......))))))))(((...((....))...)))..)))))))....	((((((..((((.......)))).((((((((.....(((......)))....)))))))))(((...((....))...)))..))))))))....	((((((..((((.......)))).(((((((((...(((......)))..)..)))))))(((.(.((....)).).)))..)))))))....	((((((..((((.......))).((((((((...(((......)))..)..))))))))....(((((.......)))))))))))....	((((((..((((.......)))).(((((((....(((......))).....))))))))......((((.......)))).)))))))....	((((((..((((.......)))).((((((((.....(((......)))....))))))))......((((.......)))).)))))))....
6KYV	/	/	/	/	/	/	/	/	/	/		(((((((((....)))))))))	.((((((((....)))))))).	(((.(((((....))))).)))	((((.((((....)))).))))	((((((((......))))))))	(((((.(((....))).)))))	((.((((((....)))))).))	/	/	/
6MCE	/	/	/	/	/	/	/	/	/	/		((((((..((((......)))))))))))))	(((((...((((......)))).))))))	((((((..(((((...).))))))))))	.((((((..((((......)))))))))).	((((((...(((......))).)))))))	(((((...(((((...).)))).))))))	((((((..((((.(...))))))))))))	(((((((..(((.((...)))))))))))	((((((..(((........))))))))))	.((((...((((......)))).))))).
6MCF	/	/	/	/	/	/	/	/	/	/		((((((((((.(((((..((((((....))).)))))))..))...))))))))))	((((((((((...(((..(((((((....))).))))....)))...)))))))))	(((((((((((((((((..(((((((....))).)))))))..)).).))))))))))	((((((((((.((((...((((((....))).)))).)).)..))...))))))))))	((((((((((.((.....((((((....))).))))).....))...))))))))))	((((((((((...((((((((((((....))).)))).)).)))...))))))))))	((((((((((...((((.((((((....))).))))..).)))...))))))))))	((((((((((...((((.(((((((....))).))))..)..))).)))))))))))	((((((((((.(((((..(((((((....)).)))))))..))...))))))))))	((((((((((...((...(((((((....))).))))......))...))))))))))
6MJ0	/	/	/	/	/	/	/	/	/	/		..((((.((((........(((((((((....)))))))))).((((((....))))))....(((.......)))((((((.....)))))).))))))))	..((((.((((........(((((((((....)))))))))).((((((....))))))...((((.......))))(((((.....)))))..))))))))	..((((.((((........(((((((((....)))))))))).((((((....))))))....(((.(...).)))((((((.....)))))).))))))))	..((((.((((........(((((((((....)))))))))).((((((....))))))...((((.(...).))))(((((.....)))))..))))))))	..((((.((((........((((((((......)))))))).((((((....))))))....(((.......)))((((((.....)))))).)))))))))	..((((.((((........((((((((......)))))))).((((((....))))))...((((.......))))(((((.....)))))..))))))))	..((((.(((........((((((((....)))))))))).((((((....))))))....(((.......)))((((((.....))))))..)))))))	..((((.((((........((((((((....)))))))..((((((....))))))....(((.......)))((((((.....)))))).)))))))))	..((((.(((........(((((((((....))))))))).((((((....))))))...((((.......))))(((((.....)))))...)))))))	..((((.((((........((((((((....))))))))..((((((....))))))...((((.......))))(((((.....)))))..))))))))
6MXQ	/	/	/	/	/	/	/	/	/	/		((((((.(((...((((((......)))))))..........)))))))))	((((((.(((...(((.((.((.(((....)))))..)))...))))))))))	((((((.(((...(((....(((....)))....)))...)))))))))	((((((..((...((((((......)))))))......)).....))))))	((((((.(((.(((((.......)))))............)))))))))	((((((..((...((((((......))))))........))...))))))	((((((.(((...((((((........))))).........)))))))))	((((((.(((...((..((.(((....)))))...))...)))))))))	(((((((.((...((((((......))))))........)).)).)))))	(((((((.((...((((((......))))))........)).).))))))
6NOA	/	/	/	/	/	/	/	/	/	/		((((((.(((...(((.((.((.(((...........)))))..)))...)))))))))	((((((.(((...(((.((.((.(((..........))).)).)))...))))))))))	((((((.(((...(((....(((...........)))....)))....)))))))))	((((((.(((...(((........................)))...)))))))))	((((((.(((...((..((.(((..........)))))...))...))))))))	((((((.(((...((............................))...))))))))))	((((((.(((...(((.((.((............)).)).)))...))))))))))	((((((.(((...(((.((..................))..)))...))))))))	((((((.(((...)))))))))	((((((.(((...(((..(.(((...........))))...)))...)))))))))
6PK9	/	/	/	/	/	/	/	/	/	/		(((((((......)))))))	((((((.........))))))	.((((((......)))))).	(((((((.(...))))))))	((((((((...).)))))))	((((((..(...).))))))	((((.((.......)).))))	.(((((........))))).	((((.(.......).))))..	.(((.(.......).)))...
6SDY	/	/	/	/	/	/	/	/	/	/		(((((((((((((((....))))))))))))))))	.((((((((((((((....))))))))))))).	((((((.((((((((....))))))).))))))	((((((((((((((......))))))))))))))	(((((((.(((((((....)))))).)))))))	(((((.(((((((((....))))))))).)))))	(((((((((.(((((....))))).))))))))	(((.(((((((((((....)))))))))).)))	(((((((((((((.(....).)))))))))))))	/
6SNJ	/	/	/	/	/	/	/	/	/	/		((((((((((((....).)))))).)))))	(((((((((((.......)))))).)))))	((((.((((((....).)))))..))))	((((.((.((((....)))).)).))))	.((((((((((....).)))))).))).	((((((((((.(....))))))))).))))	(((((((((.((....))))))).))))	((((((((.(((....)))))))).))))	(((((((.((((....)))))))).))))	((((.(((((.......)))))..))))
6SY6	/	/	/	/	/	/	/	/	/	/		.(((...........(((....))).........)))	.(((....(.((...(((....))).)).)...)))	...............(((....)))...........	.(((.......((...(((....))).)).....)))	.(((..........((.((...))).))........)))	.(((.......((...(((....)))...))...)))	.(((((...........))))).............	.(((....(.((.((.((...)).))).)...)))	.(((.......((...(((....))).))....)))	..((...........(((....)))........)).
6TQB	/	/	/	/	/	/	/	/	/	/		((((((((...))))))))	(((((((.....)))))))	((((((........))))))	.(((((((...))))))).	.((((((.....)))))).	(((((.........)))))	(((.((((...)))).)))	(((((.((...)).)))))	((((.(((...))).))))	((((((.(...).))))))
6U79	/	/	/	/	/	/	/	/	/	/		(((((((((((((((.....))).)).))))))))))	(((((((((((((((........)).)).))))))))))	.((((((((((((((.....)).)).)))))))))).	(((((((((((((((.....))).))).))))))))))	(((((((((((.(((.....)))..).))))))))))	(((((((((((.(((..(....)..)))))))))))))	((((((((((..(((.....)))....))))))))))	(((((((((((((((.(....))).)).))))))))))	(((.(((((((((((.....))).)).)))))).)))	(((((((((((((.((.....))..)).))))))))))
6U8D	/	/	/	/	/	/	/	/	/	/		.((((((((((..((((((....))))))((((((((....)))))))).(((....)))))))))))))	.((((((((((.....((...((((.((.((((((((....))))))))).)).))))))))))))))))	.((((((((((..((((((.....)))))).((((((....))))))..(((....)))))))))))))))	.((((((((((...(((((....)))))((((((((....))))))))((((....)))))))))))))	.((((((((((.......(.((((.((.((((((((....))))))))).)).)))).)))))))))))	.((((((((((......(.(.((((.((.((((((((....))))))))).)).))))))))))))))))	.((((((((((......((.(.((((.((.((((((((....)))))))).)).))))))))))))))))	.((((((((((..((((((....))))))..(((((....)))))...(((....))))))))))))))	.((((((((((..((((((....))))))((((((....))))))).((.....)).))))))))))	.((((((((((.........((((.((.((((((((....)))))))).)).))))..))))))))))
6VZC	/	/	/	/	/	/	/	/	/	/		((((((..((((....))))..))))))	.(((((..((((....))))..))))).	((((((...(((....)))...))))))	((((((((.(((....))))).))))))	(((((((.((((....))))).))))))	((((((..(((......)))..))))))	((((((..(.((....)).)..))))))	((((((...((((....))))..)))))	((((((.(((((....)))).)))))))	((((((.....((....))....))))))
6W3M	/	/	/	/	/	/	/	/	/	/		(((((((((...((((((.....))))))...)))))))))	((((((((....((((((.....))))))....))))))))	(((((((((....((((((.....)))))....)))))))))	.((((((((...((((((.....))))))...)))))))).	((((((((...((((((......)))))...))))))))	((((.((((...((((((.....))))))...)))).))))	(((((.(((...((((((.....))))))...))).)))))	(((((((.....((((((.....))))))......)))))))	(((((((.(...((((((.....))))))...).)))))))	((((((((((.....((((.....))))......))))))))))
6XH0	/	/	/	/	/	/	/	/	/	/		(((((...((((......)))))))))	(((((...(((((...).))))))))))	.((((...((((......)))))))).	((((....((((......)))).))))	(((((...((((.(...))))))))))	(((((...(((.((...))))))))))	(((((...(((........))))))))	.((((...(((((...).)))))))).	(((((....(((......))).)))))	((((....(((((...).)))).))))
6XWJ	/	/	/	/	/	/	/	/	/	/		(((((((((...)))))))))	((((((((.....))))))))	(((((((.......)))))))	.((((((((...)))))))).	.(((((((.....))))))).	((((((((.(....))))))))	((((((.........))))))	((((((((....).)))))))	((((((((.(...).)))))))	.((((((.......)))))).
7K1Z	/	/	/	/	/	/	/	/	/	/		(((((((((..((((((((.((.(((((.......)))..)).)).)...))))))))........(((.....)))((((........))))...)))))))))	(((((((((..(((((((((((.(.(((.......))).).).)))...))))))).........(((.....)))((((........))))...)))))))))	(((((((((..(((((((((((...(((.......)))...).)))...)))))))........(((.....)))((((........))))...)))))))))	(((((((((..(((((((((((.(.(((.......))).).).))...))))))))........(((.....)))(((........))))...)))))))))	(((((((((..((((((((.((.(.(((.......)))...).)))...)))))))........(((.....)))((((........))))...)))))))))	(((((((((...(((((((.((.(((((.......)))..)).))...)))))))((....)).(((.....)))((((........))))...))))))))))	(((((((((..((((((((...(((.((...........)).)))...))))))))........(((.....)))((((........))))...)))))))))	(((((((((...((((((((((.(.(((.......))).).).)))...))))))((....)).(((......)))((((........))))...)))))))))	(((((((((..((((((((((((...(((.......)))...).))...))))))))........(((.....)))((((........))))...)))))))))	(((((((((..((((((((((..(((((.......)))..)).)))...)))))))........(((.....)))((((........))))...)))))))))
