# Supplementary material for: Comparative Study of Single-stranded Oligonucleotides Secondary Structure Prediction Tools
Source: BMC Bioinformatics. 2023 Nov 8;24:422. doi: 10.1186/s12859-023-05532-5 (PMC10634105; doi:10.1186/s12859-023-05532-5)
Supplement: Supplementary file 7 — Additional file 7. Predicted secondary structure for mfold under RNA (Mathews (1999)) and DNA (SantaLucia (1998)) model in the dot-bracket notation. The PDB code is reported in the first column. "/" characters indicate either structures predicted as unfolded or software failure during the computation or sequences for which the parameters were not applied. [file 12859_2023_5532_MOESM7_ESM.pdf]

Additional File 7. Predicted secondary structures for mfold under RNA (Mathews (1999)) and DNA (SantaLucia (1998)) model in the dotbracket notation. The PDB code is reported in the first column. "/" characters indicate either structures predicted as unfolded or software failure during the computation or sequences for which the parameters were not applied.

DNA model

PDB	mfold MFE	subopt #2	subopt #3	subopt #4	subopt #5	subopt #6	subopt #7	subopt #8	subopt #9	subopt #10
1AC7	((((((....))))))	/	/	/	/	/	/	/	/	/
1B4Y	..........((((((....))))))....	....(((((((........)))))))....	.((((................)))).....	................((.....))....	/	/	/	/	/	/
1BJH	((((...))))	/	/	/	/	/	/	/	/	/
1ECU	(((((((.....)))))))	((((((((...))))))))	(((((((....)).)))))	(((((.((....)))))))	((((((.....)..)))))	..(((...........)))	(((((((....))).))))	(((...........)))..	((((((.....))..))))	/
1EN1	..(((.....))).....	...(((....))).....	/	/	/	/	/	/	/	/
1EZN	((((((((((....)))(((....)))..)))))))	(((((((((....)))..((.....)).))))))	((((((.((...............))..))))))	((((((.(((.....)))((.....)).)))))))	(((((((.......((((.....))))..)))))))	(((((((.((...(((......))))..)))))))	........(((.....)))......(((.....)))	...((....))..(((((((....))))..)))...	(((((..(((....)))..((........)))))))	...(((......)))..(((....))).........
1JVE	(((((((((((...))))))))))))	.((((((((((...)))))))))).)	.((.((((((((...))))))))..))	((((((..((((...)))..)))))))	(((((((..(((...))))..))))))	(((((((((......)...))))))))	/	/	/	/
1LA8	(((((...)))))	/	/	/	/	/	/	/	/	/
1NGO	((((((((((.....)))))))))))	((((..(((((.....)))))))))..	(((((..((((.....)))))))))..	..(((.....))).............	.((((.....))))............	((((((..(((.....)))))))))..	..(((.(((((.....))))))...)))	..((...((((.....))))))....	/	/
1NGU	((((..(((((.....)))))..))))	..(((.(((((.....)))))...)))	..............(((.....)))..	.............((((.....)))).	...........(((((.....)))))	.....((((((.....))))...))..	/	/	/	/
1OSB	.((((((....)))))).........	(((((((....))))))).......	.((((((....))))))(...)...	(((((((....))))))......)..	.((((((....)))))......)...	..(((((....))))))(.....)..	..(((((....)))))(...)....	...((((....))))(.....)...	.((((((....)))).....))..	((..(((....)))))..........
1P0U	(((((...)))))	/	/	/	/	/	/	/	/	/
1PQT	((...))	/	/	/	/	/	/	/	/	/
1SNJ	((((((((((....)))(((....)))..)))))))	.((((..(((....)))))))..((((....)))).	.((((.((.....))..)))).((((....)))).	(((((((.(((..............))).)))))))	(((((((.(((.((....)).....))).)))))))	(((((((.(((....((.....).))).)))))))	(((((((......((..........))..)))))))	...(((......)))........((((....)))).	(((((((((((((....))).....)))...))))))	.....(((((....)))...)).((((....)))).
1UUT	((((((...))))))	/	/	/	/	/	/	/	/	/
1XUE	.....((...)).....	.((.....)).......	((.....))........	/	/	/	/	/	/	/
1YTB	(((((((((((.....)))))))))))	(..(((((((((.....))))))))))..	..((..((((((.....))))))))....	....((((((((.....))))))..))..	...(((((((((.....)))))))..))).	....((((((((.....))))).)))..	..(((..(((((.....))))))))....	((((..((((((.....)))))))))))..	(....(((((((.....))))))))....	.....(((((((.....)))))))....).
1ZHU	..((...)).	/	/	/	/	/	/	/	/	/
1ZM5	.((((((....)))))).........	(((((((....))))))).......	.((((((....))))))(...)...	(((((((....))))))......)..	.((((((....)))))......)...	..(((((....))))))(.....)..	..(((((....)))))(...)....	...((((....))))(.....)...	.((((((....)))).....))..	((..(((....)))))..........
2A0I	.(.....)..	(.....)...	((.....)).	.(...)....	/	/	/	/	/	/
2A6O	((((((((....)))).)))).	(((.((((....)))).)))..	.(((((((....)))..))).	.(((((((....)))..)))).	((..((((....)))).))...	.(((((((....))..))))).	..((((((....))))...)).	..((((((....)))...))).	(((((((....))......)))).	((((((....)))......))).
2CDM	((((((....))))))).........	((((((....)))))).(......).	((((((....))))))((.....))	((((((....))))))(...)....	.(((((....)))))(.....)...	((((((....)))))......).....	.(((((....)))))(...).....	..((((....))))(.....).....	((((((....))))......))....	...(((....)))((.....))...
2EXF	..(((.....))).	...(((....))).	/	/	/	/	/	/	/	/
2JZW	..(((.....))).	...(((....))).	/	/	/	/	/	/	/	/
2K71	((....))	/	/	/	/	/	/	/	/	/
2L5K	......((((...))))......	.............((....))..	(((...((((...))))...)))	/	/	/	/	/	/	/
2LO5	(((......)))	/	/	/	/	/	/	/	/	/
2LO8	...((...))	((......))	..(.....).	.(....)...	/	/	/	/	/	/
2M8Y	((((((...))))))	.((...)).......	(((((....).))))	......((....)).	/	/	/	/	/	/
2N8A	((((((((((....)))))))))).((((((((....)))))))))	(((((((((....))))))))).(((((((...))...))))))	....(((((....))....))).((((((((((....)))))))))	(((..((.....)).)))......((((((((((....)))))))))	(((((((((....)))))))))..((.....))..((.....)).	...((((((....)))))).((....((((((....)))))))).	((((((((((....)))))))))..((.........))........	.((.........))..........(((((((((....))))))))	((((((((((....)))))))))).........((.........)).	..((....))............(((((((((....))))))))
2VHG	((((((((....)))).))))...	(((.((((....)))).)))....	.(((((((....))))..)))...	.(((((((....)))..))))...	((..((((....)))).)).....	.((((((((....))..)))))..	..((((((....))))...))...	..((((((....)))...)))...	((((((....))......))))..	((((((....)))......)))...
2VIC	....(((((((((...)))))).))))	....(((.(((((...))))).))).	(((((.....)))))...........	.....((((((((...)))))..)))	....((..(((((...))))).))..	.....(((((((((...))))..))))	(((((.....)))))...(.....).	(((((.....)))))...((....))	(((((.....))))).(....)....	(((((.....)))))...((...)).
2VJU	...((((((((....)))).))))).........	....(((.((((....)))).)))((.....))..	...((((((....)).....))))).........	..........(((....))).((..........)).	/	/	/	/	/	/
3C46	......((((((...))))))	.(.....).(((...)))...	((.....))(((...)))...	/	/	/	/	/	/	/
3DSD	.((((((((....)))))))....	...(((((....)))..))....	(..((((((....)))))).....	.((..(((....)))))......	...(((....)))..........	((....))..............	(((........)))........	/	/	/
3HXO	((((.....))))......((((((((.....)).)))).	..........(((...)))(((((((.....))).)))).	.(((((((.(((..........))).))).......))))	..((..........))..((((((.....))).)))).	(((.((((..(((...)))..))))..))).........	.....((...)).......((((((.....))).)))).	(((.((((..(((...)))..))))..)))..((...)).	.....((..........))(((((.....))).)))).	.......((...)).....(((((.....))).)))).	.(((((..........(((........)))......)))))
3Q0A	.......(((((...)))))	..(....).(((...)))..	.((....))(((...)))..	/	/	/	/	/	/	/
3Q23	......((((((...))))))	.(.....).(((...)))...	((.....))(((...)))...	/	/	/	/	/	/	/
3Q24	.......((((((...))))))	.((.....))(((...)))...	.(((.........)))......	/	/	/	/	/	/	/
3THW	((((((((((((((((((((((...))))))))).....))))))))))))))	((((((((((((((.......((..((.......)).))))))))))))))))	((((((((((((.............(((.........)))))))))))))))	(((((((((((......))).((((((............)))))))))))))	(((((...........(((..((((((............)))))))))))))	/	/	/	/	/
3WPD	((.....)).	/	/	/	/	/	/	/	/	/
3WPG	(((....))).	/	/	/	/	/	/	/	/	/
3WPH	(((....)))..	/	/	/	/	/	/	/	/	/
3ZH2	((((((((.((......)).)).)))))	..((.((.((......)).)).))...	((((((((...)))......).)))))	(((((..(............)..)))))	(((((.......((....))..)))))	..((.........))............	.(((.........)))..........	/	/	/
4ER8	......(((((..((((....))))..)))))	......(((((...(((.......))))))))	...(((.......((((....))))...))).	......(((.(((.....)))..)))..........	...............(((....)))((....))	.....(((.......)))..............	((...........((((....))))))).....	.....(((.......)))...((.......))	/	/
4F41	((((((((((((((....))))))))))))))	....((((((((((....))))))))...)).	.((...((((((((....))))))))).....	...((((.(((....))).)))).........	.(((((((...(((....)))))))))))....	/	/	/	/	/
4F43	((((((((((((((....))))))))))))))	....((((((((((....)))))))))...)).	.........((((.(((....))).))))..	.((...((((((((....)))))))))))....	........(((((.(((....))).)))..))	....((((.((...(((....)))))).)))).	....(((....)))(((....)))........	.((.......))..(((....))))........	/	/
4FF1	.......(((((...)))))	(.....)(((((...)))))	((...))(((((...)))))	.(....)(((((...)))))	..(....).(((...)))..	.((....))(((...)))..	.(.....)((((...)))).	(((....)))((...))...	/	/

RNA model

PDB	mfold MFE	subopt #2	subopt #3	subopt #4	subopt #5	subopt #6	subopt #7	subopt #8	subopt #9	subopt #10
1AC7	((((((....))))))	/	/	/	/	/	/	/	/	/
1B4Y	..........((((((....))))))....	....(((((((........))))))).....	....((((...(((.....))))))))....	(((((................)))))).....	................((.....))....	/	/	/	/	/
1BJH	((((...))))	/	/	/	/	/	/	/	/	/
1ECU	((((((((...))))))))	(((((((....)).)))))	(((((.((....)))))))	(((((((....))).))))	((((((.....)..)))))	(((.((.....)).)))..	..(((...........)))	(((((....))...)))..	(((((....)))...))..	/
1EN1	((((......))))....	(.(((......)))....	(((((.....))).))..	((.(((....))).))..	/	/	/	/	/	/
1EZN	((((((((((....)))(((....)))..)))))))	((((((......((((.....))))).)))))))	((((((((((....))).(((.....))))))))))	((((((.(((.....))).........)))))))	(((((((.((...((((....))))))..)))))))	(((((..(((....)))..((........)))))))	...((....))..((((((((....))))))).....	........((..((((((((....)))))))..))	(((((((.((((((...))))...).)).)))))))	((((((((((....))).........))...)))))
1JVE	((((((((((((...))))))))))))	.((((((((((...)))))))))).)	.(((((((((((...))))))))))).))	((((((((((......))...)))))))	((((((((.........))..))))))	((((((..((.........)))))))))	/	/	/	/
1LA8	(((((...)))))	/	/	/	/	/	/	/	/	/
1NGO	((((((((((.....)))))))))))	((((..(((((.....)))))))))..	((((((.(((.....))).))))))..	(((((((.((((.....)))))).))))	(((.(((((((.....))))).)))))	..((((((((.....))))..)))))	((((((((......))...))))))..	(((.((((((.....))).)))))))	((((((.(((.....))))))).)))	((((((((....))))......))))..
1NGU	((((..(((((.....)))))..))))	..(((.(((((.....)))))...)))	((((..(((((.....)).)).))))	.............((((.....)))).	((....(((((.....)))))..))..	..(((.(((((.....)))..).)))	............((((((.....)))))	.....(((((.....)))...))..	..(((.(((((.....))..)).)))	/
1OSB	(((((((....))))))).......	(((((((....)))))......))..	.((((((....))))))(...)...	((..(((....))))).........	((((((....))))......)))..	(((..((....))))).........	((((..(....)))))........	..(((((....)))))(.....)..	..(((((....)))))(...)....	..(((((....)))..)).......
1P0U	(((((...)))))	/	/	/	/	/	/	/	/	/
1PQT	((...))	/	/	/	/	/	/	/	/	/
1SNJ	((((((((((....)))(((....)))..)))))))	.((((..(((....)))))))..((((....)))).	(((((((.(((.............))).)))))))	(((((((.(((..((........)))))).)))))))	(((((((.(((.((....))....))).)))))))	.((((.((.....))..)))).((((....)))).	(((((((.(((....(((...))).))).)))))))	((.((((....))))).......((((....)))).	(((((((..(((....)))..........)))))))	(((((((((((((....))))....))))...)))))
1UUT	((((((...))))))	/	/	/	/	/	/	/	/	/
1XUE	((.....))........	/	/	/	/	/	/	/	/	/
1YTB	((((((((((((.....)))))))))))))	(((((..(((((.....))))))))))..	(((((((((.((.....))))).))))))	(((((((.(((((.....)).))))))))))	(((((((((....(.....))))))))))	...((((((((((.....)))))..)))).	(((((((((.....(......)))))))))	((((((((....((.....))))))))))	((((((((....(((.....))))))))))	(((((((.....((......)))))))))
1ZHU	((......))	..((...)).	...(.....)	....(...).	/	/	/	/	/	/
1ZM5	(((((((....))))))).......	(((((((....)))))......))..	.((((((....))))))(...)...	((..(((....))))).........	((((((....))))......)))..	(((..((....))))).........	((((..(....)))))........	..(((((....)))))(.....)..	..(((((....)))))(...)....	..(((((....)))..)).......
2A0I	(.....)...	(.......).	.(...)....	/	/	/	/	/	/	/
2A6O	((((((((....)))).)))).	(((.((((....)))).)))..	.(((((((....)))..)))).	.(((((((....))..))))).	((..((((....)))).))...	..((((((....)))...))).	((((((....))......)))).	((((((....)))....)))).	((((.......(....))))).	.......(((((....))))).
2CDM	((((((....))))))).........	((((((....))))))((.....))	((((((....)))))......)....	((((((....))))))(...)....	.(((((....)))))(.....)...	.(((((....)))))(...).....	.(((((....)))..))........	((((((....))))......))....	..((((....))))(.....)....	...(((....)))((.....))...
2EXF	..(((.....))).	((((......))))	...(((....))).	/	/	/	/	/	/	/
2JZW	..(((.....))).	((((......))))	...(((....))).	/	/	/	/	/	/	/
2K71	((....))	/	/	/	/	/	/	/	/	/
2L5K	(((...((((...))))...)))	..((..((((...))))))....	.............((....))..	/	/	/	/	/	/	/
2LO5	(((......)))	/	/	/	/	/	/	/	/	/
2LO8	((......))	((....))..	...((...))	/	/	/	/	/	/	/
2M8Y	((((((...))))))	(((((....).))))	......((....)).	/	/	/	/	/	/	/
2N8A	((((((((((....)))))))))).(((((((((....))))))))))	..((((((((....)))))))((....((((((....))))))))).	(((((((((....)))))))....))((((((....))))))...	((((((((((....)))))))))).((.....))..((.....)).	((((((((((....))))))))))...((((......))))......	((((((((((....)))))))))..((((.....)))))........	((((((((((....))))))))))....(((((....)))...)).	...((((((....))....))))(((((((((....)))))))))	(((....((....))))).....((((((((....))))))))))	((((((((((....))))))))))..........((.........)).
2VHG	((((((((....)))).))))....	(((.((((....)))).)))....	.(((((((....)))..))))...	.(((((((....))..)))))...	((..((((....)))).)).....	..((((((....)))...)))...	((((((....))......))))...	((((((....)))....))))....	((((.......(....)))))....	......(((((........)))))
2VIC	....((((((((.....)))).))))	....(((((((((...))))).))))	....(((.((((.....)))).))).	.....((((((.....)))..)))))	.....((((((.....))..)))))	....((..((((.....)))).)).	......((((((.....))).....)))	(((((.....)))))...........	....((((((...))))....))).	....((((........(....)))))
2VJU	...((((((((....)))).))))).........	((.((((((((....)))).))))).....))..	....(((.((((....)))).)))((.....))..	...(((((((....)).....))))).........	..........(((((........)))))).......	.......((((....)))....))..........	((.....((..((........)).)).....))..	...............(((......))).......	/	/
3C46	......((((((...))))))	(((.........)))......	/	/	/	/	/	/	/	/
3DSD	.((((((((....)))))))....	(((..(((....)))))).....	.(((.(((....)).))))....	...(((((....)))..))....	(((........)))........	((....))..............	/	/	/	/
3HXO	((((.....))))......((((((.....))).)))).	.(((((....(((...)))...((((.....)))))))))	((((.(((.(((..........))).)))...))))....	((((((((..(((...)))..))).)))).........	.((((((.(..((..........))..)....).)))))	.((((((((.((..(((......))).))))..).)))))	.((((((((.((.....(((....)))))))..).)))))	(((.((.((...)).)))))((((((.....))).)))..	(((..((...))......)))((((((.....))).)))..	.....((...(((...))).((((((.....))).)))))
3Q0A	.......(((((...)))))	/	/	/	/	/	/	/	/	/
3Q23	......((((((...))))))	(((.........)))......	/	/	/	/	/	/	/	/
3Q24	.......((((((...))))))	.(((.........)))......	/	/	/	/	/	/	/	/
3THW	((((((((((((((((((((((...))))))))).....))))))))))))))	((((((((((((((...((...)).((.......))...))))))))))))))))	((((((((((((((.......(((........)))....))))))))))))))	((((((((.((......))..((((((............)))))))))))))))	............((.((((((((...))))))))))....((((.....))))	/	/	/	/	/
3WPD	((.....)).	(((....)))	/	/	/	/	/	/	/	/
3WPG	(((....))).	((.....))..	/	/	/	/	/	/	/	/
3WPH	(((....)))..	((.....))...	/	/	/	/	/	/	/	/
3ZH2	(((((((.((......)).)).)))))	((((((((.....))......).)))))	((((((((...)))......).)))))	((((((((......))..)).))))))	(((((..(((......)).)..)))))	((((((((.....))...)...)))))	(((((.......((....))..)))))	(((((((.....))...))...))))	..((.((.((......)).)).))...	(((((..((.........))..)))))
4ER8	......(((((..((((....))))..)))))	...(((.......((((....))))...))).	......(((((...(((.......))))))))	...((((((.((((....))))..)))..))).	.((.......)).((((....)))).......	((...........((((....)))))).....	(((.((((.......)))))))........	...((.((..............)))).......	...((.((((........)).)))).......	/
4F41	((((((((((((((....))))))))))))))	....((((((((((....)))))))..))).	.(((...((((((((....))))))))))....	.((((((.(((....))).)))))).......	...............(((((....))))))..	.(((((((((....)))...))))))....	/	/	/	/
4F43	((((((((((((((....))))))))))))))	....((((((((((....)))))))..))).	.(((...((((((((....))))))))))....	..........((((.(((....))).))))...	.((((((...((((....)))))))))))....	/	/	/	/	/
4FF1	.......(((((...)))))	/	/	/	/	/	/	/	/	/

	DNA model									
PDB	mfold MFE	subopt #2	subopt #3	subopt #4	subopt #5	subopt #6	subopt #7	subopt #8	subopt #9	subopt #10
4HT4	.(((((((...))))))).........	....((((...))))((((....)))).	((((((((...)))))))......)...	((((((((...))))).........))).	..((((((...))))))(.....).....	((((((((...)))).........)))).	..((((((...))))))(..........).	.(((((((...)))).........)))	((....)).......((((....)))).	((((((((...)))))).......))...
4KB0	((((((....))))))..	((((((....)))).)).	((((((....)))))..)	((((((....))))..))	((((..(....)))))..	/	/	/	/	/
4KB1	((((((....))))))..	((((((....))))).)	.(((((....)))).)..	((((..(....)))))..	(.((((....))))..).	/	/	/	/	/
5F55	.......((...))	/	/	/	/	/	/	/	/	/
5GWL	/	/	/	/	/	/	/	/	/	/
5GWQ	/	/	/	/	/	/	/	/	/	/
5HRU	.............(((((.....))))).....	.(((.........(((((.....)))))))).	..........((.(((((.....))))).)).	.......((....(((((.....))))))))..	/	/	/	/	/	/
5HTO	(((((.........(((((.....))))))))))	...........((.(((((.....))))).))..	.....((.((....(((((.....)))))).))	(((((((((......)))).........)))))	(((((.........((.......))....)))))	/	/	/	/	/
5N2Q	((((((((....))))))))........	((((((((....))))))))..(...).	.(((((((....))))))).(.....).	((((((((....)))))))).(.....)	.(((((((....))))))).(...)...	.(((((((....))))))).((...)).	.(((((((....))))))(.....)...	.(((((((....))))))((.....))	.(((((((....))))))(...).....	(((((((....)))).....)))...
5OND	.(....)..	((....)).	..(.....)	/	/	/	/	/	/	/
6FK4	..(((((...))))).	.(((.....))).....	((((.....))))...	/	/	/	/	/	/	/
6FK5	(((((...))))).	/	/	/	/	/	/	/	/	/
6FKE	..(((...))).	.((((...))))	/	/	/	/	/	/	/	/
6IY5	(.....)...	((...))...	/	/	/	/	/	/	/	/
6J37	/	/	/	/	/	/	/	/	/	/
6M0B	/	/	/	/	/	/	/	/	/	/
6M0C	/	/	/	/	/	/	/	/	/	/
6SEI	.(((.((((((((...))))))))..)).)))	..(((....)))...................	.((.....))((((...))))...........	.(((.((..(((...........)))).)))	......(((......))).............	/	/	/	/	/
6U82	((((((((((((((((...)))))))))))...))))))	(((.....(((((((((...))))))))))))........	........((((((((...))))))))).((......))	((((((.....(((((...)))))))))))........	......((((((((((...))))))))........)).	......((((((((((...))))).........))))).	(((((((.((((.......)))))))))))........	..........(((((...)))))((......))....	/	/
17RA	/	/	/	/	/	/	/	/	/	/
1A1T	/	/	/	/	/	/	/	/	/	/
1A4T	/	/	/	/	/	/	/	/	/	/
1A51	/	/	/	/	/	/	/	/	/	/
1A60	/	/	/	/	/	/	/	/	/	/
1A9N	/	/	/	/	/	/	/	/	/	/
1AFX	/	/	/	/	/	/	/	/	/	/
1ANR	/	/	/	/	/	/	/	/	/	/
1ATO	/	/	/	/	/	/	/	/	/	/
1ATV	/	/	/	/	/	/	/	/	/	/
1ATW	/	/	/	/	/	/	/	/	/	/
1AUD	/	/	/	/	/	/	/	/	/	/
1B36	/	/	/	/	/	/	/	/	/	/
1BGZ	/	/	/	/	/	/	/	/	/	/
1BVJ	/	/	/	/	/	/	/	/	/	/
1BZ2	/	/	/	/	/	/	/	/	/	/
1BZ3	/	/	/	/	/	/	/	/	/	/
1CQ5	/	/	/	/	/	/	/	/	/	/
1CQL	/	/	/	/	/	/	/	/	/	/
1D0U	/	/	/	/	/	/	/	/	/	/
1DRZ	/	/	/	/	/	/	/	/	/	/
1E4P	/	/	/	/	/	/	/	/	/	/
1EBR	/	/	/	/	/	/	/	/	/	/
1EBS	/	/	/	/	/	/	/	/	/	/

	RNA model									
PDB	mfold MFE	subopt #2	subopt #3	subopt #4	subopt #5	subopt #6	subopt #7	subopt #8	subopt #9	subopt #10
4HT4	.(((((((...))))))).........	...(((((...)))))(((....)))..	((((((((...)))))))......)...	((((((((...))))).........))).	..............((((....)))).	.(((.......))).((((....)))).	((((..((((...))))......)))).	((....)).......((((....)))).	.(((.((...............).)))	((((((((...))))).......))...
4KB0	((((((....))))))..	((((((....)))).)).	((((..(....)))))..	((.(((.....)))))..	((((((....)))))..)	((((((....)))..))	((((........)).))..	((..(((....)))..))	((((...(...)))))..	(((...(.....))))..
4KB1	((((((....))))))..	((((..(....)))))..	((((((....)))).)).	((.(((.....)))))..	((((((....)))))..)	((((........)).))..	(((..((....)))))..	((..(((....)))))..	((((((....))))..))	((((...(...)))))..
5F55	.......((...))	...((........))	..((.....))...	/	/	/	/	/	/	/
5GWL	/	/	/	/	/	/	/	/	/	/
5GWQ	/	/	/	/	/	/	/	/	/	/
5HRU	.........(((.(((((.....))))).)))	((((.........(((((.....)))))))))	((.....))..(.(((((.....)))))).)..	.......((....(((((.....)))))))..	....((((.....(((((.....))))))))))	.((((...)))).(((((.....))))).....	(((((..((((.........)))..).))))	...((......))(((((.....)))))....	((((...(...(..(.....)..)...)))))	((((((((......))))).......))))
5HTO	..........(((.(((((.....)))))))).	.((((.........(((((.....)))))))))).	.((.....))(((.(((((.....))))).))).	.....((((.....(((((.....))))))))))).	..((((...)))).(((((....))))).....	.(((((..((((.........))).)..).)))).	.((((...(...(..(.....)..)...))))).	.((((((((......))))).........)))).	.....(((..(((..(.....)..).))..))).	.(((.((((......)))).((((....)))))).
5N2Q	((((((((....))))))))........	(((((((....)))))))((...)).	(((((((....))))))).(.....)	(((((((....))))))).((...))	(((.(((.........))).)))..	/	/	/	/	/
5OND	((....)).	/	/	/	/	/	/	/	/	/
6FK4	..(((((...))))).	((((.....))))...	/	/	/	/	/	/	/	/
6FK5	(((((...))))).	/	/	/	/	/	/	/	/	/
6FKE	.((((...))))	/	/	/	/	/	/	/	/	/
6IY5	((...))...	/	/	/	/	/	/	/	/	/
6J37	/	/	/	/	/	/	/	/	/	/
6M0B	/	/	/	/	/	/	/	/	/	/
6M0C	/	/	/	/	/	/	/	/	/	/
6SEI	.(((.((((((((...))))))))..)).)))	....((..((((((...)))))))).......	........((((((...))))))((....)).	.(((.((..(((...........)))).)))	....(((((......))).)).........	.............((((((....)))).))..	....((......))..........	....((......))....(((.......)))	/	/
6U82	((((((((((((((((...))))))))))...))))))	(((((((.((((.......))))))))))))........	.......(((((((((...)))))))))((......))	......(((((((((((...)))))))........))).	((((((.............((((...)))).))))))	((((((((..........)))).......))).........	((.............)).((((.....)))))......	.....................((((((......))))))	((((((..........((......))..))))))	...............((.((((....)))).....))
17RA	((((((.((....))))))))	(((((.(((....))))))))	(((((..((.....)))))))	...((((((....))).))).	(((((((...))))....)))	((.((((...))))))....	/	/	/	/
1A1T	((((((((....))))))))	(.((((((....))))))).	((.(((((....))))))).	(((((...((...)))))))	........(((......)))	/	/	/	/	/
1A4T	(((((.....)))))	((.........))..	/	/	/	/	/	/	/	/
1A51	((((.((....((((((((...))))))))....)).))))	((((.........((((((...))))))((....)).))))	((((....((...((((((...))))))))).......))))	.(((...))).((((((((...))))))))...........	((((.((....))((((((...))))))((....)).))))	((((.((((......(((...))))))((....)).))))	.......(((...((((((...))))))((....))..)))	.((.........((((((((...)))))))))....))....	.(((...)))...((((((...)))))((........)).	.(((...))).((..((((...))))).(((....))).)).
1A60	(((((.......)))))......((((((......))))))...	(((((.......))))).(((((....)))))...........	(((...............)))..(((((((......))))))...	(((((.......))))).(((((....)))))((....))....	(((((.....))))).(((((((....)))))......)).....	(((((.......)))))........((.((....)).)).	(((((.....)))).........((((((......)))))))).	.((..((..((((((........))))))....))..)).	(((((.......))))).((....((....))......)).....	.((...))..............((((((......)))))...
1A9N	(((((.............))))).	((((((((....)))))...))).	(((((...((...))...))))).	(((((.......((...))))))).	.(((......))).(((....)))	.((((((((....)))))....)).	((((((......).....))))).	((((((((....)))))..).)).	((((((((....))).)..)).)).	((((((((....))).)).)..))).
1AFX	((((....))))	/	/	/	/	/	/	/	/	/
1ANR	((((((...((((......))))))))))	((((((...(((.(.....))))))))))	((((((...(((.((...)))))))))))	(((((((((.......)))....)))))	(((((((((......)))......))))))	((((((((((....))))......)))))	((((((.((..........))...))))))	((((((((........)).......))))))	((((((..........((...))))))))	(((........))).((....))).....
1ATO	((((((.......))))))	((((...((....))))))	((((.((......))))))	(((((..(.....))))))	((((..((.....))))))	.((.........))......	((((....(.....)))))	(((((....(...))))))	/	/
1ATV	((((((....)))))).	.(((((....))).)).	((.(((....)))))..	(((.((....)))))..	/	/	/	/	/	/
1ATW	(((((....))))).	((((.....).))).	((((.....)).)).	/	/	/	/	/	/	/
1AUD	.((((.((((....)))).)))).......	(((((.((((....)))).......))))	......((((....))))...((....)).	(((((.((((....))......)).)))))	(((((.((....(((....))).)))))))	((....((((....)))).)).........	((((.........((.........))))))	((......))..((((.......))))..	(((.(((...))).((........)).)))	(((((.((..((...))......))))))
1B36	(((((.......((((....))))..........)))))	(((((..((...((((....))))......))..)))))	(((((..((((.((((....))))))...))..)))))	(((((.......((((....))))(((...))))))))	(((((.......))))).((.((((...))))).))	.((......)).((((....))))..............	.(((.....((.((((....)))))))....))).....	.((((.......((((....))))))))).........	((((.....((.((((....))))))).)))).......	.((((.(((.......))).)))))..............
1BGZ	(((((..(((....))).)))))	((((..((((....)).))))))	(((((((((...))))..)))))	(((..(((((....)).))))))	(((((((((...)))))..)))))	(((....(((....)))..))).	.((..(((((....)).))).))	(((.....((((....))))))))	((..(((((...)))))..))..	(((.......))).........
1BVJ	(((((.(((......))))))))	(((((..(.........))))))	(((((....((....)).)))))	((((..((.........))))))	......((((.........))))	((((((...)........)))))	((((((...))........))))	((((...(.((....)).)))))	/	/
1BZ2	(((((.......)))))	/	/	/	/	/	/	/	/	/
1BZ3	(((((.......)))))	/	/	/	/	/	/	/	/	/
1CQ5	(((((((....(((....(((....)))....))).)))))))	((((....((.(((....(((....)))....)))..))))))	(((((((.....((....(((....))).)).....)))))))	(((((((.((......))(((....))).........)))))))	(((((((....(((...((((....))..)).))).)))))))	(((((((.((.((......)))).((......)).)))))))	(((.....((......))(((....)))....)))........	((......)).(((....(((....)))....)))........	(((((((.........((((((....)).....))).)))))))	(((...............(((....)))....((....)))))
1CQL	(((((((....(((....(((....)))....))).)))))))	((((....((.(((....(((....)))....)))..))))))	(((((((.....((....(((....))).)).....)))))))	(((((((.((......))(((....))).........)))))))	(((((((....(((...((((....))..)).))).)))))))	(((((((.((.((......)))).((......)).)))))))	(((.....((......))(((....)))....)))........	((......)).(((....(((....)))....)))........	(((((((.........((((((....)).....))).)))))))	(((...............(((....)))....((....)))))
1D0U	((((((.((....))))))))	.(((((.((....)))))).)	(((((..((.....)))))))	((.(((.((....))))))).	(((.((.((....))))))).	((.....))...........	((((((........).)))))	.(((((.((....))))).))	((((((........)).))))	.((....))...........
1DRZ	..(((((.((((.(((((......))))).)))))))))......(((((((((....)).)).))))))...	..(((((.((((.(((((......)))).)))))))))(((((.....))))..(((.((.....)).))).	..(((((.((((.(((((......)))).)))))))))......((((((.....)))))).........	.....((((((((((((((.((...))....))))))))....))))).)).(((.((.....)).))).	.(((((((((((((((((.((.....))....))))))).....)))).))).....))))......... ..	((((.....))))....(((..(((...((((((....((((.....))))...)))))))))..)))..	.........((((((((((.((.((((....))))...((((...)))).....)).)))...)))))	((((((......(((((...))))).)))))))((....)).......(((.((.....)).))).	....(((.((....)))))(((....((.(((((....(((.....))))...)))))).))...)))).	(((.((.......)).)))((((...((.((((((....((((.....)))...)))))).))...)))).
1E4P	((((...(((....)))...))))	..(((..(((....)))....)))	((.((..(((....))))).))..	.((....(((....)))))....	..(((....((.......))..)))	((((((...(....)))...))))	((((......(.....)...)))))	((((......((...))...))))	/	/
1EBR	((((..((((((....)))).))...))))	...((.((((((....)))).)).....))	.........((((....))))..((....))	.((.....((((....))))))..........	.((.(((.........))).))..........	((((.((...)).(((.....)))..))))	(((.((.......)).)))..........	.((....)).((....))............	((((..(.......((....)).).)..))))	((((......(((((....)).)).))))
1EBS	((((..((((((....))))))...))))	((((..((((((....))))).)..)))))	((((.((((((((....))))).)).))))	.(((..((((((....))))))).)))...	...((.((((((....))))))......))	....((((((((....))))))......))	........(((((....)))))((......))	.(....((((((....))))).).)....	((((.((..(((....)))))....))))	((((.(((..((....)))))....))))

PDB	DNA model: mfold MFE	subopt #2	subopt #3	subopt #4	subopt #5	subopt #6	subopt #7	subopt #8	subopt #9	subopt #10
1EIY	/	/	/	/	/	/	/	/	/	/
1EKZ	/	/	/	/	/	/	/	/	/	/
1ESH	/	/	/	/	/	/	/	/	/	/
1ESY	/	/	/	/	/	/	/	/	/	/
1ETF	/	/	/	/	/	/	/	/	/	/
1EUQ	/	/	/	/	/	/	/	/	/	/
1EXY	/	/	/	/	/	/	/	/	/	/
1F7F	/	/	/	/	/	/	/	/	/	/
1F84	/	/	/	/	/	/	/	/	/	/
1F85	/	/	/	/	/	/	/	/	/	/
1F9L	/	/	/	/	/	/	/	/	/	/
1FFY	/	/	/	/	/	/	/	/	/	/
1FHK	/	/	/	/	/	/	/	/	/	/
1FJE	/	/	/	/	/	/	/	/	/	/
1FQZ	/	/	/	/	/	/	/	/	/	/
1FYO	/	/	/	/	/	/	/	/	/	/
1G70	/	/	/	/	/	/	/	/	/	/
1GRZ	/	/	/	/	/	/	/	/	/	/
1GTR	/	/	/	/	/	/	/	/	/	/
1HLX	/	/	/	/	/	/	/	/	/	/
1HS1	/	/	/	/	/	/	/	/	/	/
1HS2	/	/	/	/	/	/	/	/	/	/
1HS3	/	/	/	/	/	/	/	/	/	/
1HS4	/	/	/	/	/	/	/	/	/	/
1HS8	/	/	/	/	/	/	/	/	/	/
1HVU	/	/	/	/	/	/	/	/	/	/
1HWQ	/	/	/	/	/	/	/	/	/	/
1I3X	/	/	/	/	/	/	/	/	/	/
1I46	/	/	/	/	/	/	/	/	/	/
1I4B	/	/	/	/	/	/	/	/	/	/
1IDV	/	/	/	/	/	/	/	/	/	/
1IK1	/	/	/	/	/	/	/	/	/	/
1IKD	/	/	/	/	/	/	/	/	/	/
1JBT	/	/	/	/	/	/	/	/	/	/

PDB	RNA model: mfold MFE	subopt #2	subopt #3	subopt #4	subopt #5	subopt #6	subopt #7	subopt #8	subopt #9	subopt #10
1EIY	((((((..((((........)))).((((.......)))))......(((((.......))))))))))))....	((((((((.((((........))))..(((......(((((.......))))))......)))))))))))....	((((((..((((........)))).....((((.......))))...(((((........)))))))))))....	((((((((.((((((((.((.....)))))))).(((((.......))))))......))))))))))....	((((((((.((((........))))..(((...((((((((.......))))))...))))))))))))))....	((((((((.((......((..((((.(((((((.......))))...))))))))....))))))))))))....	((((((((.((..((((((....((..((.((((.......))))))..))...))))))..)))))))))))....	((((((((.(((((((((.((.....)))))))))).(((((...((....))...)))))..))))))))))....	((((((((.((((.........))))).(((((.......)))))..(((........)))......))))))))....	((((((.....(((((((.((.....))))))))....(((...((........)))...))))))))))....
1EKZ	(((((((((((((....))))))))))))))	.....((((((((....))))))...))..	(((((...(((((....)))...))))))))	((....))(((((....))))).........	((((....))))......((((....))))	((((....))))...(((.......))))...	.((.((......)))..((((....))))	/	/	/
1ESH	(((((...)))))	/	/	/	/	/	/	/	/	/
1ESY	((((((((....))).))))	.......((((....))))	((((..((.....))))))	/	/	/	/	/	/	/
1ETF	((((((..((((((....)))).))...))))))	.(((((....((((....)))).))).))......	.((....)).((((....))))..(((....)))	((((((.(((...)))..((.....)).))))))	......((((((((....))))).)......)))	((((((..(((.......))......)..))))))	((((((..(....(((.....).)))..))))))	(((((((((.........))).......))))))	.(((.......(((....))))))..........	((..((.((...)).))..))...(((....)))
1EUQ	(((((...(((..........))).(((((.......)))))..((((((.......))))))))))).....	(((((((.(((..........)))...((((((.(((..((....))..))).)))))..)))))))).....	((((((((((...))))))......(((((.......)))))..((((((.......)))))).)))).....	....((((((...)))))).(((.(((((.......)))))..((((((.......))))))......)))).	(((((...(((..((......))...(((.......)))))).((((((.......))))))))))))).....	(((((...(((....(((.....))))(((.......)))))).((((((.......)))))))))))).....	((..((((((...)))))).((..(((((.......)))))..((((((.......))))))...))..)).	(((((...(((..((.......))..(((...)))......))).((((((.......)))))))))))))).....	((((((((((...((......)).(((((.......))))).)))))....(((....)))))))))).....	..((.....))....(((..((..(((((.......)))))..((((((.......))))))..))..)))).
1EXY	((((((((((((....))))..)))..)))))).	((((((((...((.......)).)))....))))).	...((.((.(((....))))).)).........	..(((.......)))..((((....)))).....	........((((....))))...((......))	((...)).((((....)))).............	.((.(.((((((....))))........))).))	(((((.......((...........))...))))).	((((..((...))...((((....)))))))).	............((..((((....))))))...
1F7F	(((((.((((((....)))))))))))	(((((((((.((((....))))))))))))))	(.(((.((((((....)))))))))))).	(((.(.((((((....)))))))))))).	((.((.((((((....))))))))))))	(((((.((((((...).))))))))))	(((((.(((((.....).)))))))))	((....((((((....))))))))))...	(..((.((((((....))))))))))..	((..(.((((((....))))))))))..
1F84	((((.....(((.......)))....))))	((((......(((......)))...))))	((((..((..(......)..))....))))	((((......((((...).)))......))))	((((.((......)).))))...........	((((..(..(.......)..)....))))	((((.........(((.....)))..))))	...............((((.......))))	((((.((......)).))))((......)).	((((.......((.((...))))...))))
1F85	(((((....)))))	..((.....))...	..(((...)))...	/	/	/	/	/	/	/
1F9L	((((..(((....)))..))))	.((...(((....))).))...	(((...((((.....)))))))	..(...(((....))))).....	((((...((....)))...)))	..((....))............	(((...))).............	/	/	/
1FFY	(((((((........(((((((.((((.(((((((.((....)).)))..)))))))))).)).))))))))))))..	(((((((..((((.........))))).(((((.......))))).....((((........)))))))))))))..	(((((((...(((.((((((....)).))))(((....)))..)))...(((((.......))))))))))))..	(((((((....(((((.(((.......))))))))..............(((((.......))))))))))))..	(((((((.......(((((((.((((.((((...(((.........))))))))))).)).))))))))))))))..	((((((((((((.....)))))((((.((((((((.((....)).)))..))))))).........)))))))..	((((((((.((.(((((.(((........))))))))...((((((.........))))..))).)))))))))..	((((.....)))).(((((((.((((.(((((((.((....)).))))..))))))).)).))))).........	((((.....)))).((((((....)).))))((((....)))((.(((((((((.........)))))..)))))))	(((((((..((((.....)....((((.(((((((.((....)).)))..))))))).))))......)))))))..
1FHK	((((......))))	...(((.....)))	/	/	/	/	/	/	/	/
1FJE	((((..............))))	((((...((......)).))))	(((((......)......))))	..........((......))..	(((((......))......)))	((.........))...........	/	/	/	/
1FQZ	(((.....(((......)))....)))	(((......(((......)))...)))	(((..((..(......)..))....)))	(((.....((((...).)))....)))	.((((......))))..........	.((((......))))...((.....))	(((..(..(........)..)....)))	(((.((.....)).)))..........	.((((......))))(((......)))	(((.........(((.....)))....)))
1FYO	((((...((((....))))....))))	(((((..((((....)))..).))))	(((....((((....))))..)))...	(((((..((((....))))..)).))))	((((..(((((....)))...))))))	((((((..(((....)))))...))))	((((((((.((...))))))...))))	...((..((((....)))).....)).	(((...(((((....))))....))))	...(.((((((....))))....))).
1G70	((((((..(((((....))).))...))))))	((((((..(......((....)))..))))))	((((((.....((..((....)))).))))))	......(((......(((....)))......)))	.(((((....(((....))).))).)).....	.((....)).(((....)))..((((....)))	((((((..(.....((...)).)..))))))	.........((.(((((....))))...)))).	...((((((.....))))).....(((....)))	.(((((((.....)))).))).........
1GRZ	(((((.((..(((((((...(((..((....(((.((((((((((.((((((((((....)))))))))).((((((....))))).)))).....)))))))))...))..)))..)).)))))....((((...(((((((((....))))))))..))))..((((....))))...)).))))).(((((((......)))))))................((.((((((....)))))..)).........	(((((.((..(((((((...(((..((....((((.((((((((.((((((((((....)))))))))).(((((....)))).))))......))))))))...))..)))..)).)))))...((((....((((((((.....)))))))).(((((...)))))..))))...)).))))).(((((((......))))))))................((.((((((....))))))..))........	(((.(((...(((((((...(((..((....((((.((((((((.(((((((((((....))))))))).(((((....))))).))))......))))))))...))..)))..)).)))))....((((...(((((((((....))))))))..))))...........)))...(((((......(((((((.....)))))))....))))).)))...((.(((((....)))))..)).......	(((((.((..(((((((...(((..((....((((.((((((((.((((((((((....))))))))).(((((....))))).))))......)))))))))...))..)))..)).))))))....((((...(((((((((....))))))))..))))..((((.....))))...)).))))).(((..(((....((((.....))))....)))...((.((((((....)))))..))....))).	(((((.((..(((((((...(((..((....((((.((((((((.((((((((((....)))))))))).(((((....))))).)))).....))))))))...))..)))..)).)))))....((((...(((((((((....))))))))..))))..((((....))))...)).))))).....((.(((((((.........(((...)))......(((((....)))))..))))))))).	..((((.......))))......(((((..((((.........(((((.((((((((((....))))))))).(((((....))))).)))))....))))..((((.((((....)))....(((...((((...((((((((....))))))))..))))))).))))...(((((..((((.....(((((((.....)))))))....)))))))).))))))((((((....))))).............	..((((.......))))......((((..((..((((.((((((((.(((((((((....))))))))).(((((....))))).))))......))))))))....(((((..........((...((((...((((((((....))))))))..)))))))........(((((..((((.....(((((((.....))))))....)))))))))))))).((((((....))))).....)))))).	...........((((((...(((..((....((((.((((((((.(((((((((....))))))))).(((((....))))).)))).....))))))))...)).)))..)).)))).(((.((((...((((((((....)))))))))).).(((((((.(((...((((........))))...)))..))))))))................))))..(((((....))))).))).........	..((((((.....((..(((..((.........)).)))..(((((.((((((((((....)))))))))).(((((....))))).))))....)))))))).....(((((...........((...((((((...((((((((....))))))))..)))))))........(((((..((((.....((((((((.....)))))))....))))))))))))).(((((....))))))...........	..((((.......))))......((((((....((.(((((.(((((.((((((((((....))))))))).(((((....))))).)))))..........((((.((((............)))).))))...((((((((....))))))))).)))))))........(((((..((((......(((((((.....)))))))....))))))))).)))))(((((....)))))............
1GTR	(((((((.(((.........)))...((((((.(((..(((....)))..))).))))))..)))))))).....	(((((((.(((....))).........((((((.(((..(((....)))..))).))))))..))))))).....	((((((..(((.........))).(((((.......)))))......(((((.......)))))))))))).....	(((((((.........(((.....)))((((((.(((..(((....)))..))).))))))..))))))))....	((((((..(((..((......))...(((.......))))))....(((((.......)))))))))))).....	....((((((...)))))).(((.(((((.......))))).....((((.......)))))........))).	((((((..(((..((......))..(((...)))......)))....(((((.......)))))))))))).....	(((((((.(((....)))..((..((((..(((....)))...)))..)))..........)))))))....	....((((((...)))))).((.(((((.......))))).........(((.(((....))).)))...))).	(((((((.......(((...((..(((((.......))))).....))....)))........))))))).....
1HLX	((((((((....))))))))	((.(((((....))))))).	.((((((....)))))).))	(((...((.....))..))).	(..(((((....))))))..	((..((((....))))))..	((((...(.......)))))	(((...((.......)))))	(((.....))).........	.((......)).........
1HS1	(((......))).	/	/	/	/	/	/	/	/	/
1HS2	(((......))).	((((...).))).	/	/	/	/	/	/	/	/
1HS3	(((......))).	/	/	/	/	/	/	/	/	/
1HS4	(((......))).	/	/	/	/	/	/	/	/	/
1HS8	(((......))).	/	/	/	/	/	/	/	/	/
1HVU	...(((((.........))))).........	..........((((((.........))))))	...............((((......)))).	.((.........))..((((......)))).	((.(((((.........)))))....))...	...(((..(((((......))))).....)))	.((((.........)))).............	((((...))))(((((........))))).	/	/
1HWQ	(((((...(((((.....)))))...))))))	.........(((((((......))).))))	(((((((......))).))))..........	((.((((.(.....).)))).))........	..((....(((((....)))))))......	.........(((((..((.....)))))))	(..((.....))..)..(((.....)))..	(((((......((.........)).)))))	/	/
1I3X	(((((((.....)))))))	....(((((.....)))))	(((..........)))....	(((.((.....)))))....	(((..(...)..)))....	(((.(......).)))....	((.(((.....)))))....	/	/	/
1I46	(((((...)))))	/	/	/	/	/	/	/	/	/
1I4B	(((((...)))))	/	/	/	/	/	/	/	/	/
1IDV	(((....)))	/	/	/	/	/	/	/	/	/
1IK1	(((((...))))).	/	/	/	/	/	/	/	/	/
1IKD	(((((((....)))))))....	.((((((....)))).))....	((((((....)))))...)).	.((((((....))).)))....	((((((....))))..).)).	((((((....)))..)).)).	.((((((....))))....)).	(((.(((....)))).))....	(((.(((....)))))).....	((((((...).))))))....
1JBT	((((((.................))))))	((((((((...........))...))))))	((((((...((..........)).))))))	((((((((........)))))......)))	((((((((.....))........)))))))	((.(((((.......)))))..)).....	((((((((..........))....))))))	((((((((.....))).........)))))	((((((.......(........)))))))	(((((........((........)))))))

	DNA model											RNA model									
PDB	mfold MFE	subopt #2	subopt #3	subopt #4	subopt #5	subopt #6	subopt #7	subopt #8	subopt #9	subopt #10		mfold MFE	subopt #2	subopt #3	subopt #4	subopt #5	subopt #6	subopt #7	subopt #8	subopt #9	subopt #10
1JO7	/	/	/	/	/	/	/	/	/	/		((((((...((((....))))..)))))))	..........((((....))))((....)).	...(((((..((((....))))...))).))	.((....)).((((....))))((....)).	(((.......((((....)))))))......	........((((((....))))....))..	(((((.....((((....)))).))...)))	((.((.....((((....))).)).))...	..........(((...((....))...)))	.((.(((.......))).))..((....)).
1JOX	/	/	/	/	/	/	/	/	/	/		(((((.((......)))))))	(((((.((.(...))))))))	((((((........))).)))	((((.........))))....	(((((.(.(.....)))))))	(((((((......))))).)))	(((.((......)))))....	((....((......)))...	(((...)))............	/
1JTJ	/	/	/	/	/	/	/	/	/	/		(((((((.........)))))))	(((((((........))..)))))	(((((((.....)....))))))	..(((....)))...........	..(((.........)))).......	(((((((.....))....)))))	..(((....))).((...))...	.........((.......))...	(((((........))).))....	..(((((...)).))).......
1JTW	/	/	/	/	/	/	/	/	/	/		.(..((....))..).	.(((((....))))).	((.(((....))))).	....(((....)))..	/	/	/	/	/	/
1JUR	/	/	/	/	/	/	/	/	/	/		((((((((....)))))..)))	((.(((((....)))...))))	(((((((.(...)))))..)))	((((((.((...)))))..)))	(((.....(.....)....)))	(((.....((...))....)))	(((....((....))....)))	/	/	/
1JWC	/	/	/	/	/	/	/	/	/	/		((((((....))))))	(((((.(...))))))	((.(((.....)))))	((((..(....)))))	((((.......)).))	((((...(...)))))	/	/	/	/
1JZC	/	/	/	/	/	/	/	/	/	/		(((((...)))))	/	/	/	/	/	/	/	/	/
1K2G	/	/	/	/	/	/	/	/	/	/		..(((....)))..........	..(((....))).((....)).	(((((....))).......)).	.........(((.....)))..	/	/	/	/	/	/
1K4A	/	/	/	/	/	/	/	/	/	/		(((((....)))))	/	/	/	/	/	/	/	/	/
1K4B	/	/	/	/	/	/	/	/	/	/		(((((....)))))	/	/	/	/	/	/	/	/	/
1K5I	/	/	/	/	/	/	/	/	/	/		(((((((((.....)))))))))	((((((((......)).))))))	(.(((((((.....)))))))).	((.((((((.....)))))))).	((((((.((......))))))))	......((((((.....))))))	(((((.((......)).)).)))	......((((((...)))))))	(((.(((((.....))).)))))	......((((.(......)))))
1K6G	/	/	/	/	/	/	/	/	/	/		((((((((((....))))))))))	((((((..(......)))))))	.((..((((....))))))...	(((((..(........))))))	...((((((....))))..)).	(((.......)))........	(((.......).)).......	(((((.......(...))))))	(((.......((.....)))))	/
1K6H	/	/	/	/	/	/	/	/	/	/		(((((((((....)))))))))	((((((((.....)).))))))	(((((((((....)..)))))))	((((((((....))..))))))	((((((((...)...)))))))	.((((..........))))...	((((((((...))...))))))	...((((((....))..)))).	/	/
1KAJ	/	/	/	/	/	/	/	/	/	/		(((((((....)).))))).............	(((..(((((.......))))).....)))..	(((((((....)).))))).((....))....	(((....(((((....)))...))...)))..	........(((((............))))).	........(((((((.....)).....))))).	(((((..((...))))))).............	.((...))(((((............))))).	(((((.....))....)))............	(((.......)))..................
1KKA	/	/	/	/	/	/	/	/	/	/		(((((((...)))))))	((((........)))).	(((.........)))..	.(((..........)))	/	/	/	/	/	/
1KKS	/	/	/	/	/	/	/	/	/	/		((..((((((....))))))..))	((..((((((....)))))).)).	((..(((((.(...))))))..))	((..((.(((.....)))))..))	(....(((((....)))))))....	((....((((....)))))))....	....((((((....)))))...).	....((((((....))))...)).	((..((((..(....)))))).))	((..((((.......)).))..))
1KP7	/	/	/	/	/	/	/	/	/	/		((((.((....(((....)))..)).))))	(((((.....))...(((....)))..)))	((.((.....))...(((....)))))....	((.((.....)).))(((....))).....	(((((((...........))).)).))....	..........((...(((....))).)).	(((((((..((.....))))).)).))....	((.((.....)).)).(((.....)))...	((((........((...(....))).))))	((((........((.((...)).)).))))
1KPD	/	/	/	/	/	/	/	/	/	/		(((((((....)).))))).............	((.(((((((.......))))).....)))).	((.((..(((((....)))...))...)))).	........(((((............))))).	........(((((((.....))......))))).	(((((..((...))))))).............	(((.......)))...(((.......)))...	.((...))(((((............))))).	((....((.(((....))).......)))).	/
1KXK	/	/	/	/	/	/	/	/	/	/		.....(((.((((((...((((((((((((((....))))))..))))))))))..))))))))).....	((((.......))))...((((((((((((((((....))))))..))))))))))...((....)).....	.((..(((((........(((((((((((((((....))))))..))))))))))))...))..)).....	.((..((.....((((....)))...((((((....))))))......(((.....))).)).)..))....	.....(((.((((((............((((((....)))))).((((......))))))))))))))).....	....(((.....((((....)))...((((((....))))))......)))......((....)).....	.....(((.((((((..........(((((((((....))))))..........)))))))))))......	.(((((......((((....))))...((((((....)))))).))).)).......((....))....	.....(((.((((((......((.....))...(((..(((......))))))...))))))))))......	...(((......((((....))))))).((((((....)))))).....(((.........)))..........
1L1C	/	/	/	/	/	/	/	/	/	/		...((((..((((....)))))))).....	((.((((..((((....)))))))))..))	((.((....((((....))))...)).))	((.((....((((....))))....))))	...(((((..(((....)))))))).....	((....((.((((....))))..)..))	.....(((.((((....))))....))).	......((.((((....))))....))...	.........((((....))).).......	.((...)).((((....))))........
1L1W	/	/	/	/	/	/	/	/	/	/		((((..((((((....))))))..)))).	((((.((.((((....))))))..)))).	(((...((((((....)))))))))).....	((((.(((((.(....))))))...)))).	((((...((((........)))))))))).	((((..(.((((....).))))...)))).	((((...(((((....).))))).)))).	((((.((..(((....))))))...)))).	((((.((((.........))))...)))).	(((...(((....))).((....)))))).
1LDZ	/	/	/	/	/	/	/	/	/	/		(((((....(((((....)))))..)))))	.....(((.(((((....)))))...))).	((((((....((((....))))))..))))	((((((.(((.........))).))..))))	((.......(((((....))))).).))...	(((((....((..(((...))))).)))))	........((((((....)))).......))	(((((((......)).......)...)))))	((......))..((((..........))))	.((((((......))...))))........
1LNG	/	/	/	/	/	/	/	/	/	/		..((((..(((((.((((((((((....))))))))..)))))....((((((.....(((.....(((....)))......)))..)))))).))))	..((((..(((((.(((((((((....)))))))))..)))))....(((((((..(((..((....(((....))).))...))).))))))).))))	((((((((((((((.(((((((((((....)))))))))..)))))...))).)))))..((.(((...(((....))).((....)).))).)).).....	..((((..(((((.(((((((((....)))))))))..)))))).((.(((((((....((....))(((....)))......))))))).)).))))	(((((((((((((.(((((((((....)))))))))..))))).....))).)))))....((((...(((....))).((........)).)).))..	(((((((((((((.(((((((((....)))))))))..)))))...))).)))))...(((.....(((....)))......)))..(((.....)))	..((((..(((((.((((((((((....)))))))))..)))))))....((((((...(((..((......))..))).((....)).))))))).))))	((((((((((((((.((((((((((....)))))))))..)))))...))).)))))......((....(((....))).)).(((.........))).	..((((..(((((.((((((((....))))))))..))))).((((((((......((....))(((....))).)))..))))))......))))	..((((..(((((.((((((((....)))))))))..)))))...((((.((.....)).))))(((....))).((........))....))))
1M5L	/	/	/	/	/	/	/	/	/	/		((((....((((.((((....)))...))))..))))	((((....(((...(((.((....))))))))..))))	((((.....((..((((....)))).)).......))))	((((...........((((((.......))))))))))	((((.((......)).(((((.......))))).))))	............(((.(((((.......))))))))..	((((....((((.((.....)).....))))..))))	..((........)).((((((.......)))))))....	.((.......))(((.((((((.......)))))))..	((((.........(((.(((...)))..)))..))))
1M82	/	/	/	/	/	/	/	/	/	/		((((((((((....)))).))))))	((((((((((....).)))))))))	((((..((((....))))..)))).	((.(((((((....))).)).))))	(((...((((....))))..)))..	((((((.(((....))..)))))))	((((..((((....)))))...)))	(((((..(((....))))...))))	((....((((....))))..))...	(((((((.((....)..))))))))
1MFJ	/	/	/	/	/	/	/	/	/	/		..(((((((....)))))))	..(((((((...).))))))	..((((((.....).)))))	..(((((..(....))))))	..(((..(((....))))))	..((((((....)..)))))	..((((((....)))..)))	/	/	/
1MFK	/	/	/	/	/	/	/	/	/	/		((((((.(((....)))))))))	(((((.((((....)))))))))	(((((((........).))))))	((((((((......)).))))))	...((((((....))))..)))	.((((........))))......	((....((((....))))))...	((((((.........)..)))))	((((((.........))..))))	((((((...).......))))))
1MFY	/	/	/	/	/	/	/	/	/	/		....((((..((((....))))..))))....	(((.((((..((((....))))..)))))))	..........((((....))))((....)).	...((.....((((....))))........))	.((....)).((((....))))..........	(((.......((((....))))))))......	(((((.....((((....))))).))...)))	..........(((...((....))...)))	.((.(((.......))).))...((....)).	...............(((........)))
1MNX	/	/	/	/	/	/	/	/	/	/		((((.......((((((((....))))))))........))))	((((.....((.((((((((....)))))))))....)).))))	.((((....))))((((((....))))))...........	............((((((....))))))((........)).	(((........((((((((....))))))........)))))	((((....((...))((((....))))...........))))	.((((....)))).((((....))))((..........))..	((((.(((....)))..(((.....)))..........))))	.((((....))))...(((.....((...)).......))).	..(((...........(((....)))))))............
1MT4	/	/	/	/	/	/	/	/	/	/		((((((((.........))))))))	((((((((..(.....)))))))))	((((((((.((...))))))))))	((((((((..((....)))))))))	(((((((..(((....)))))))))	(((((......((....)))))))	((((......(((....))))))))	((((((.......(...)))))))	(((((.......((...))))))))	((((((....).........)))))
1N66	/	/	/	/	/	/	/	/	/	/		((((..(((....)))..))))	((((((((....))))..))))	(.((..(((....)))..))).	((.(..(((....)))..))).	.(((...((....)))))....	((((((......))....))))	((....(((....)))))....	/	/	/
1N77	/	/	/	/	/	/	/	/	/	/		(((((((.(((((........)))))(((((.......)))))....(((((.......)))))).)))))))....	((((((((.......(((........)))((((.......))))......(((((......))))).))))))).....	(((((((.........((((((((........)))......)))))....((((.......))))).))))))).....	((((((..((((((........)))))((((((........))))).......)))))).(((((((....)))))))....	((((((((..(((((((.((((.......)))))))....))))).....(((((((......))))).)))))))....	(((((((..........(((......(((((.......))))).)))(((((.......))))).)))))))....	((((((((..(((..(((.......))))))................(((((.......)))))).)))))))....	((((....(((((........))))))).))))).......((((.....(((((......))))).....)))).....	((((((((.((.....))......(((..((((((((((......))...)))))).)).)))....))))))).....	((((((((.((((((........)))))((((((.......))))))...(((....)))........)))))))....
1N8X	/	/	/	/	/	/	/	/	/	/		(((((((.((((((((....))))..))).)))))))	((((((((.(((..((........)).))).)))))))	((((((((((....(....)))))......)))))))	........((((((((....)))))))).((....)).	......(((((((((....))))))).........))	......((((((((....).......))))))).	((((((..(((....)))...((.....))))))))	...((.....((((((....)))))))............	((.......))((((((....)))))..............	....((((....)))).(((.((.....))..))).
1NA2	/	/	/	/	/	/	/	/	/	/		((((((....((.....))....)))))).	((((((((..........)))....))))).	(((...........((((.....)))))))	(((.......)))..((((.....))))...	(((((((........))........))))).	(((...)))......((((.....))))...	.(((...........))).............	.((..(((..........)))...))......	/	/
1NBR	/	/	/	/	/	/	/	/	/	/		((((((.(((((......)))))))))))	((((((((...........)).))))))	((((((((.((....))...)).))))))	((((((.(.(((......)))).)))))	((((((..((...........))))))))	.(((..((.....))..)))..........	.(((((((......))))))).........	((((((......(((...)))..))))))	((((((..(...........)..))))))	(((..((((((((......))))).)))))
1NC0	/	/	/	/	/	/	/	/	/	/		(((((((((.....))).))))))	((((((.((......)).))))))	(((((.(((......)).).))))	(((((.(((......))).))).))	((((.((((......)).))))))	(((((((.........))..)))))	((((((..(.........)))))))	((((..(((.........)))))))	((((((.........)))...)))	..((((.........))))....
1NYB	/	/	/	/	/	/	/	/	/	/		((((((((((......))))))))))	((((((((.(.....)))))))))	((((((.(((.....)))))))))	((((((((........)).))))))	((((((((..(....)))))))))	(((((((..((.....)))))))))	((((((..(((....)))))))))	(((.(((((......))))))).))	(((((((...(.....))))))))	((((((...((.....))))))))

	DNA model											RNA model									
PDB	mfold MFE	subopt #2	subopt #3	subopt #4	subopt #5	subopt #6	subopt #7	subopt #8	subopt #9	subopt #10		mfold MFE	subopt #2	subopt #3	subopt #4	subopt #5	subopt #6	subopt #7	subopt #8	subopt #9	subopt #10
1OOA	/	/	/	/	/	/	/	/	/	/		(((((....((((....))))...)))))	(((((..(.((((....))))..))))))	(((((((..((((....)))))).)))))	((((((..(..((....))..).)))))	((((((..(..(.....).)..).)))))	((..((((......))))..)).......	((((((..(..((...)).)..).)))))	((((..((.((((....))))..))))))	((((((((..(((....)))))).)))))	(((((...((.((....)))).. .)))))
1OQ0	/	/	/	/	/	/	/	/	/	/		(((((.....)))))	((((((...))))))	(((.......)))..	..(((.......)))	/	/	/	/	/	/
1OSW	/	/	/	/	/	/	/	/	/	/		((((.(((....)))...))))	((...(((....)))...))..	(((.((((....))...)))))	((.(((((....))...)))))	((...(((....)))...).).	((...(((....)))....)).	(((.((.((......)))))))	...(((((....)))....))	((((((......))....))))	...((.(((.......))).))
1OW9	/	/	/	/	/	/	/	/	/	/		((((...(((....)))..))))	((((((...(....)))..))))	....((.(((....)))....))	((((.....((.....)).))))	((((...(((....))))..)))	((((.....(((...))).))))	......((((....)))....).	/	/	/
1P5M	/	/	/	/	/	/	/	/	/	/		(((((.((((.....((((...(((.((....)).)))....)))))))))))))	(((((.((((...(((......(((.((....)).)))..)))...)))))))))	(((((.((((.....(((....((((((....)).....))))))))))))))))))	(((((.((((...(((...((((.((....))))))....)))...))))))))))	(((.(((((((.(....)))))))))))....((((.((......)))))))....	((((((((((.(....)))))).. ((....)).......)))))).......	(((((.((((.((....))......((...(((.....))).))...))))))))	(((.(((((((.(....)))))))))).((....))...)))...((........)).	(((((((((((...............)))).((....))..))))))).......	/
1P5N	/	/	/	/	/	/	/	/	/	/		((((...((((((.......))))))....))))	((((((.....))).)))..(((((....)))))	........((((((((......))))).)))).	((((....((.....((....))....)).))))	........((.....))...(((((....)))))	.((.....))..........(((((....)))))	((((((.....))).)))...((....)).....	(((.......)))........(((((....)))))	(((....((...)).)))..(((((....)))))	.((....((((((.......)))))).)).....
1P5P	/	/	/	/	/	/	/	/	/	/		(((((.((((.....((((.......)))).((((((........)))))).(((((.....)))))..)))))))))	(((((.((((.....((((...(((.((...((((((........)))))).....)).)))....)))))))))))))	(((((.((((..(((.((.((..((((.....))))..)).)))))......(((((.....)))))..)))))))))	(((((.((((.............((((.....((.....))....)))).(((((.....)))))..))))))))))	(((((.((((...(((.(..((((..(....((((((........))))))).)..)))..).)))...))))))))))	(((((.((((.............((((.....))))...((.((((((((.......))))))))))..)))))))))	(((((.((((........(((...((((.....))))........)))....(((((.....)))))..))))))))))	(((((.((((........(..(((.....)))..)......((....))....(((((.....))))))..)))))))))	(((((.((((........(((.....(((((.....))).)).....)))....(((((.....))))))..)))))))))	(((((.((((......((((......))))...(((.....((((((.(....))))).))....)))..))))))))))
1P6V	/	/	/	/	/	/	/	/	/	/		.......(((((((....)))))))...(((((........)))))	...((..(((((((....)))))))).(((((........)))))	((.(((((((((((....))))))).........)))).))....	....((.(((((((....))))))).....(((.......))))).	((((((.((((((....)))))))....))))))..........	((.((((((((((....))))))).((....)))))).))....	.......((((((....))).(((((((....))))))..)))).	.......((((((..(((..((.((....)))..))))))))))).	(((((((.((((((....))))))))....))))))((.....)).	((.........((((....))))).....(((((........)))))
1PJY	/	/	/	/	/	/	/	/	/	/		((((((((((....))))))))))	((((((.((.....))))))))	((((((((.....)).))))))	((((((((.....)))).))))	((((.((((....))).)))))	((.((((((....))))).)))	((.((((((....))).)))))	(((((((......))..))))))	(((((((......)))..))))))	(((..((((....)))..))))
1Q75	/	/	/	/	/	/	/	/	/	/		(((((.....)))))	(((..((...)))))	/	/	/	/	/	/	/	/
1QC8	/	/	/	/	/	/	/	/	/	/		(((.((((((((....)))))))))))	(((.((((((((...)).))))))))	(((.(((((((...))).))))))))	....(((.(((...)))))).....	.....((((..........))))..	....((((((((....)))))))..)..	/	/	/	/
1QFQ	/	/	/	/	/	/	/	/	/	/		(((((.....)))))	..(((.......))).	.((........))..	/	/	/	/	/	/	/
1QWA	/	/	/	/	/	/	/	/	/	/		((((((((....)).))))))	(.((((((....)).))))).	((.(((((....)).))))).	((((((........)..)))))	(((....)))..........	((((((........))..))))	((........))..........	/	/	/
1QWB	/	/	/	/	/	/	/	/	/	/		((((((...............))))))	((((((...((.......)).))))))	(((((((.......)......))))))	(((((((......)).......)))))	((((((...........))...))))	(.((((...............)))))).	(((.......))).............	((.(((...............)))))).	((..........))..........	/
1R2P	/	/	/	/	/	/	/	/	/	/		(((((((((((((....))))))..))))))))	((((((((((...((......))..))))))))))	(((((((..(((((....))))))).....)))))	(((((((((((........))....)))))))))	..((....((((((....)))))).)).......	(((((((.....(((.........)))))))))))	..((.......(((....)))))...........	..((.....))(((....)))...............	/	/
1R4H	/	/	/	/	/	/	/	/	/	/		(((....)))	/	/	/	/	/	/	/	/	/
1R7W	/	/	/	/	/	/	/	/	/	/		(((((((.......((((((....))))))))))))	(((((((..(((....)))........)))))))	(((((((......(((....)))....))))))))	(((((.....))..(((((....))))).)))...	(((.....))).((((((....))))))......	(((((((.((((.....)).))....)))))))	...(((.......((((((....)))))...)))	.((((.....))))..(((.(((....)))))).	.((((.....)))).(((....))).((....))	(((((.(((((.....))))).)).....)))...
1R7Z	/	/	/	/	/	/	/	/	/	/		(((((((.......((((((....))))))))))))	(((((((......(((....))).....)))))))	((((....)))).(((((....)))))).......	(((((.....)).(((((....)))).)))...	(((((((..((((....)).))).....)))))))	.((((.....))))..(((.(((....)))))).	...(((.......((((((....)))))...)))	.((((.....)))).(((....))).((....))	(((((.(((((.....)))))).)).....)))...	.((((.....))))..((((.......))))...
1RFR	/	/	/	/	/	/	/	/	/	/		(((((...(((((....)))))...))))))	((.(((.((....)).))).))..........	.............(((((.....)))))..	.((.......))..(((((.....)))))...	(..((....))..)................	(..((....))..)..(..((....))..)	/	/	/	/
1RHT	/	/	/	/	/	/	/	/	/	/		.((((((.((....)))))))))..	((.((((.((....))))))))..	(((.(((.((....)))))))))..	.((((((.....))....))))..	(..((((.((....)))))))).. .	......(((..........)))...	.(((((((...)))....))))..	.(((((((.......)..))))))..	((..(((.((....)))))))....	.((((((.......))..))))..
1RKJ	/	/	/	/	/	/	/	/	/	/		((((((((....)).))))))	(.((((((....)).))))).	((.(((((....)).))))).	((((((........)..)))))	(((....)))..........	((((((........))..))))	((........))..........	/	/	/
1RNG	/	/	/	/	/	/	/	/	/	/		((((....))))	.((....))...	/	/	/	/	/	/	/	/
1RNK	/	/	/	/	/	/	/	/	/	/		(((((((....)).))))).............	(((((((....)).))))).((....))......	......((((((..............)))))))	((.(((((((........)))))).......)))))..	......((((((((((......))....)))))))	((.((..(((((....)))...)).....))))..	(((((..((...)))))))..............	(((.......)))...(((........))).....	.((...))(((((...............))))))..	((....((.(((....))).((....)))))))..
1ROQ	/	/	/	/	/	/	/	/	/	/		(((((....)))))	/	/	/	/	/	/	/	/	/
1S03	/	/	/	/	/	/	/	/	/	/		((((((.((.((...((((((((....))))))))..))))))))))	((((((.((....((((((((((.....)))))))).))))).))))))	.......(((((.((((((((((...)))))))).)))...))))).	....(((((.((...((((((((....)))))))..)).).)))).	((((((.((.....))((((((((....))))))))......)))))))	((((((.((.((...((((((...))......))))..))))))))))	((((...((.....))((((((((....)))))))..)))).......	((((((.((.((...........(((.....)))...))))))))))))	.((....((.((...((((((((....))))))))..)))))))....	((((((.((.((..((.....))(((.....)))...))))))))))
1S2F	/	/	/	/	/	/	/	/	/	/		((((((.(((....)))))))))	.(((((.((........)))))))	((((((((.....))..))))))	(((.((.((.(((....))))))))).	((((((((.....)))..)))))	.((((..((.........))))))	(((((..........))))))...	((((((((.....)....))))))	(((............)))....	((((.(.(((....)))))))).
1S9S	/	/	/	/	/	/	/	/	/	/		.((((..((((((....))))))..))))......((((..(((.(((((((((....))))))....)))))))))))).(((((((((....)))))))))).	.((((..((((((....))))))..))))......((((..(((.((((...(((.......)))..)))))))))))).((((((((....))))))))))).	.((((..((((((....)))))).(((((.....))))).(((.(((((((((....)))))....))))))))))).((((((((((....)))))))))).	...(((((.(((((.......)))))..)))))..((((..(((.(((((((((....)))))....)))))))))).(((((((((....)))))))))).	...(((((.....(((.......))).)))))..((((..(((.((((((((((....)))))....))))))))))).((((((((....)))))))))).	.(((((.((((((....))))))...........(((....)))((((((((((....)))))....))))..)))))).((((((((....))))))))).	(((.....((((((....))))))))..........((((..(((.((((((((....)))))....))))))))))).((((((((....)))))))))).	.((((..((((..((((.......)))......))))......(((.((((((((....))))).....))))))))))).((((((((....)))))))))).	.((((..((((((....))))))..))))......((((...((..(......)..))......((((......))).)))).((((((((....)))))))))).	.((((..((((((....))))))..))))....(((((((...))).(((((....)))))......))).))..(((((((((....))))))))
1SCL	/	/	/	/	/	/	/	/	/	/		((((((.................))))))	((((((...((.........)).))))))	((((((((..........))...))))))	((((((((.....))........))))))	((((((((.......)).......)))))	((((((((..........))....))))))	((((((((.......))).......)))))	((((((((.....)))........)))))	.(((.(((.......)))..)))......	((((((........(.........)))))))
1SLP	/	/	/	/	/	/	/	/	/	/		.((((((....)).)))).	(((((((....)).)))))	....((........))...	/	/	/	/	/	/	/
1SYZ	/	/	/	/	/	/	/	/	/	/		(((((.(((.....)))..)))))	(((((..((.......))..)))))	((((((((.........))..)))))	((((..(((.....)))..)).))	((((((..........)))...)))	(((((.......((....)))))))	/	/	/	/
1SZY	/	/	/	/	/	/	/	/	/	/		(((((((.......)))))))	((((((........)).))))	((((.((.........))))))	(((.(((........))))))	.....(((..........)))	/	/	/	/	/
1T28	/	/	/	/	/	/	/	/	/	/		((((((((((..............))))))))))	((((((((((.....))))).........)))))	((((((((((..........)))......)))))))	.(((.(((((.....))))))....))).......	((((.(((((.....))))).((.....))))))	((((((((((..........))).))...)))))	.((..(((((.....))))).))...........	((((.((.....))...((.......))..))))	.....(((((.....)))))........((...))	.((..((.....))..))..............
1TFN	/	/	/	/	/	/	/	/	/	/		.((((((.((....)))))))))..	((.((((.((....))))))))..	(((.(((.((....)))))))))..	.((((((.....))....))))..	(..((((.((....)))))))).. .	......(((..........)))...	.(((((((...)))....))))..	.(((((((.......)..))))))..	((..(((.((....)))))))....	.((((((.......))..))))..
1TJZ	/	/	/	/	/	/	/	/	/	/		(((..((((....))))..)))	((((.((((....)))))).))	.(...((((....))))).....	.((...(((....))))).....	.(((...((....))))).....	((.....))....(((...)))	(((...)))....(((...)))	(((...(((....)..)).))))	(((.....((.....))..)))	......((.((.....)).)).
1TLR	/	/	/	/	/	/	/	/	/	/		((((...(((....)))..))))	((((.(.(((....))).)))))	((((((..((....)))).))))	((.(((.(((....))).)))))	(((((((..(....)))).))))	((((..((.(....)))..))))	((((.........))))......	............(((....)))	(((((........).....))))	((((.((...)).))))......
1TXS	/	/	/	/	/	/	/	/	/	/		((((..((((...(((((....)))))...))))))))	((((..((((.(((......))).......)))))))))	((((((.((....(((((....))))..)))).))))	((((.((....))(((((....))))))......))))	((((..((......)).......(((....))))))))	((((..((.......))..(((((.....)))))).))))	((((..((((..((.........))......))))))))	..((((((....)))))......((((....)))).....	((((.((....(((......)))...)).......))))	((((.((.((....)).))...((((....))))))))
1U2A	/	/	/	/	/	/	/	/	/	/		(((((((......)))))))	(((((((...)).)))))	(((((..((....)))))))	((((((.....)...)))))	(((((((...))...)))))	/	/	/	/	/

	DNA model											RNA model									
PDB	mfold MFE	subopt #2	subopt #3	subopt #4	subopt #5	subopt #6	subopt #7	subopt #8	subopt #9	subopt #10		mfold MFE	subopt #2	subopt #3	subopt #4	subopt #5	subopt #6	subopt #7	subopt #8	subopt #9	subopt #10
1U63	/	/	/	/	/	/	/	/	/	/		((((((((((..((((((((....))))).)))......))))))))))	((((((((....((((((((....)))).)))).((...))))))))))	((((((((((....((((((....)))......)))...)))))))))	((((((((((....(((((...........)).)))...)))))))))	((((((((((....(((((....)))))..)).......)))))))))	((((((((((....((((....((.....)).))))...)))))))))	((((((((((....((((.....((...))))))...))))))))))	.(((((......((((((((....)))).))))......)))))......	((((((((((((.....)......((((......))))...)))))))))	(((((((((((((.........))).((....)).....))))))))))
1ULL	/	/	/	/	/	/	/	/	/	/		(((((..(((((((....))))..)))...)))))	(((((.........(((((....)))))...)))))	..((...((.((((....)))).))))........	..........((((....)))).((........))	.((((((.......)))))).((((......)))..	....((.(((((((....)))).....)))......))	((((((((.((.....)))).)........)))))	((((......((.(((((....)))))..))))))	(((((.........(((.........))).)))))	((.....)).((((....))))..............
1UN6	/	/	/	/	/	/	/	/	/	/		((((((....((....))((((((((.....((.((....)).))....)))))))))))))	((((((....((....)).((.(((....))).))...((((((.....)))))))))))))	...((.....))..(((.(((((((.....((.((....)).))....)))))))).)))...	((((((....((((.((..(.......)..))))))..((((((.....)))))))))))))	((..((((..((....))...))))..))..((((...((((((.....)))))).)))).	((((((...((((..((..(((((((....))).((....)).)))....)))))))))))	(((.((((..((....))...))))..((((((((.............))))))))).)))	((((((..((..(((..((.....)).)))..))....((((((.....))))))))))))	(((((((....(....))))).)))......((((...((((((.....)))))).)))).	((((((...((((.(((...))).......((.((....)).)).......)))))))))
1UUU	/	/	/	/	/	/	/	/	/	/		((((((((...))))))))	((((((..(...)))))))	(((((..(.....))))))	.(((.......)))).....	......(((.....)))..	(((((.......)..))))	/	/	/	/
1VOP	/	/	/	/	/	/	/	/	/	/		(((((...)))))	/	/	/	/	/	/	/	/	/
1WKS	/	/	/	/	/	/	/	/	/	/		((((((.....))))))	(((.((......)))))	((((((....))).)))	(((((.....)..))))	/	/	/	/	/	/
1WZ2	/	/	/	/	/	/	/	/	/	/		(((((((........((((......)))).(((((.((..((((((..(((....)))....)))))).)).))))).)))))))....	((((((((..((..((((......)))).))((((((..((.(((((.....)))))).))..))))))........)))))))....	((((((((....((((((.......((.((((((.......)))))).))....))))))).((((......)))).)))))))))....	(((((..((((((....))).))).((((((((((((..((.(((((.....))))).))..)))))......)))))))))))....	(((((((..(((..((...))....)).(((((.((..((((((..(((....)))....)))))).)).))))).)))))))....	(((((((..(((..((((......))))).(((((.......)))))........)))..(((((........)))))))))))....	((((((((...((.((((......)))).(((((.......)))))...))..((((((((........)))))).)))))))))....	((((((((((....))))........((((((((((((..((.(((((.....))))).))..))))).....))))))))))))....	((((((((....((((((.((......)).)))..)))..((.(((((.....))))).)).((((......))))).)))))))))....	(((((((((.((..((((......)))).))((((((..((.(((((.....))))).))..)))))).))))))))).........
1XHP	/	/	/	/	/	/	/	/	/	/		((((.((((.(((.....)))..)))).))))	..((((.....))))....((.....))....	..((((.....))))....(((((...)))))	((((.((((((..........)))...)).))))	..((((.....))))..((((.....)).)).	..((((.....))))........(((...)))	((((.((((......((....)))))).))))	((((.((.......))....((.....))))))	/	/
1XSG	/	/	/	/	/	/	/	/	/	/		(((((.((((((....)))))))))))	(.(((.((((((....))))))))))).	((.(..((((((....)))))))..)))	(((.(.((((((....)))))))))).	((.((.((((((....)))))))).))))	(((..((((((....)))))).))))	....((((((((....)))))).)).	((..((((((((....))))))).))))	(((((.((((((...).))))))))))	(((((.(((((.....).)))))))))
1XSH	/	/	/	/	/	/	/	/	/	/		((((((.(((((....))))))))))))	(((((((.(((((....)))))))))))))	(((((.((((((....))))))))))))	(.((((.(((((....))))))))))).	(((.((.((((((....)))))))))).	((.(((.(((((....))))))))))))	((((((.(((((...).))))))))))))	((((((.((((.....).))))))))))	((.((..(((((....)))))..))))	(..(((.(((((....))))))))))..
1XWP	/	/	/	/	/	/	/	/	/	/		((((......)))).	/	/	/	/	/	/	/	/	/
1XWU	/	/	/	/	/	/	/	/	/	/		((((.......)))).	/	/	/	/	/	/	/	/	/
1YMO	/	/	/	/	/	/	/	/	/	/		(((......)))..((((((((((..............))))))))).	((((((....((.....))....))))))).................	((((((....((.....))....))))))...((......)).....	..(((((((((...((((.....))))..........))))).)))).	.((((.((((....((((.....))))......)))).))))......	(((...)))......((((((((................)))))))).	.((((((((.....((((.....))))...)))))......))).....	(((......)))..(((((((((...........))...)))))))).	((((((((..........)))....)))))...................	(((......)))..((((((((........))......))))))).
1YN1	/	/	/	/	/	/	/	/	/	/		((((((.....))))))	(((((....)...))))	(((((....))...)))	/	/	/	/	/	/	/
1YNC	/	/	/	/	/	/	/	/	/	/		(((((((((((.........)))).)))))))	((((((((((......))......))))))))	((((((((......((...))..)))))))	(((((.....(((...........))))))))	((((((((((...))...........)))))))	.(((..((......))...))).........	((((((........(((......))))))))	/	/	/
1YNG	/	/	/	/	/	/	/	/	/	/		(((((((((((.........)))).)))))))	((((((((((......))......))))))))	((((((((......((...))..)))))))	........((((((..........)))))).	.(((..((......))...))).........	((((((...((............))))))))	/	/	/	/
1YSV	/	/	/	/	/	/	/	/	/	/		((((((((((((.....))))))))))))	(((((((....((.....))))))))))	(((((((((..(.....))))))))))	(((((((..((.......)))))))))	(((((((((........))..)))))))	(((((((((((...)....))))))))))	(((((((((((...))....))))))))	((((((...(.((.....)))))))))	((((......)))).............	((((...(((.((.....)))))))))
1Z2J	/	/	/	/	/	/	/	/	/	/		(((((((((((((((((((....))))))))))))...))))))))	((((...(((((((((((((....)))))))))))))...))))...	........((((((((((((....))))))))))))(((....))).	(((....)))((((((((((....)))))))))).((((....))))	.....((((((((((((((.....))))))))))))......))...	((((((.........)))))).....(((((((.........)))))))	(((((((((....(((((((....))))......)))..))))))))	((((((((........((((....((.....)).)))))))))))))	((((((((((((.....)).....((.....))..))).))))))))	(((.........)))((((....))))......((((....))))
1Z30	/	/	/	/	/	/	/	/	/	/		(((((((....)))))))	((((((.....)).))))	......(((....)))..	/	/	/	/	/	/	/
1Z31	/	/	/	/	/	/	/	/	/	/		(((((.(((..(((....)))...))))))))	(((((.((((((....)))......)))))))	((((((((...(((....))).)))..)))))	..((.(((...(((....))).))))).....	(((((((...)))))))(((....))).....	(((((((...)))))))((((........)))...	(((((.((((.(....))))))......)))))	..((....)).(((....)))............	(((((.(((.........((...))))))))))	..(((.((((.(....))))))))........
1ZBN	/	/	/	/	/	/	/	/	/	/		((((((...((((....)))).))))))	(((((..(.((((....)))).))))))	((((((.((..........))..))))))	((((..((.((((....)))).))))))	((((((.((((....))).)..))))))	((((((((......))........))))))	((((((((.........))....))))))	((((((((......))).......)))))	((((.(((......))).))))).......	(((((((...........))...))))))
1ZC5	/	/	/	/	/	/	/	/	/	/		((((((((((((((((....))))))))))))...))))))	((...((((((((((((....)))))))))))...))...	......((((((((((....))))))))))((.....))	((((((....((((((....))))......)))..))))))	(((.........((((((....)))))))))...........	((((((((..((((.......))))..))........))))))	((((((......(((((....((.....))))))))))))	(((......))).((((....)))).(((..........)))	........(((.(((((....((.....)).))))))..)))	((((((((((....))......((.....))..))).)))))
1ZIF	/	/	/	/	/	/	/	/	/	/		((((....))))	((.(....))).	.(((....).))	/	/	/	/	/	/	/
1ZIG	/	/	/	/	/	/	/	/	/	/		((((....))))	((.(....))).	.(((....).))	/	/	/	/	/	/	/
1ZIH	/	/	/	/	/	/	/	/	/	/		((((....))))	((.(....))).	.(((....).))	/	/	/	/	/	/	/
28SP	/	/	/	/	/	/	/	/	/	/		(((((....(((....)))....)))))	(((((...((((....))..)).)))))	.((.......(((....)))....))..	.((((....(((....))).)).))...	(((((....(.(....))......)))))	(((((....((.....)).....)))))	...((....(((....))).....)).	.((......(((....))).))......	.........(((....))).((...)).	.......(((((....)).......)))
2A9L	/	/	/	/	/	/	/	/	/	/		((((((((((...((((....))))))))...))))))	.((((.((((...((((....)))))))).)))))....	(((....)))...((((....))))............	(((....)))...((((....)))).((....))....	(((....)))...((((....))))((........)).	(((....))).((((((....))))........))....	(((((((((........))).........))))))	((((((.........(((...........))).))))))	.(((..(((.........)))))).............	(((.((((.....)))..........((....)).)))
2ADT	/	/	/	/	/	/	/	/	/	/		(((((..((((((((((((....))))))))))))...)))))	........((((((((((((....)))))))))........))).	(((((.(((..((((((((....))))))...))))).)))))	((((........((((((((....))))))))))).........	((((...(((...((((((....))))))))))))).........	(((((..((((((..........((...))))))))...)))))	(((((..((.....)).(((((.........)))))..)))))	(((((.(((........(((((......))))))))).))))))	(((((............(((....)))((.....))...)))))	...........((((((((....)))))...........))..
2AHT	/	/	/	/	/	/	/	/	/	/		(((((((((((....)))).)))))))	(((((((((((.......)))..))))))	((((((..((.......))..))))))	((((((((.........))...))))))	(((((.(((((....)))..))))))	((((((((.........)))...)))))	(((((.(((((....)))..)))))))	(.(((((((((....)))).))))))).	((.(((((((((....)))).))))))).	((((((...(((.......)))))))))
2AKE	/	/	/	/	/	/	/	/	/	/		(((((((..((((.......)))).((((((.......)))))......(((((.......)))))))))))).	(((((((..((((.......)))).((((....))))..........(((((.......))))))))))))).	(((((((..((((.......)))).(((((.......))))).((((.((....))))))....))))))).	(((((((..(((..((....(((((((((((.......)))))....)))))......))..)))))))))).	(((((((..((((.......))))).((.(((....((((....))))....))).)).......)))))))).	(((((((((((.(((((((((...(((((.......)))))..))))).))))))..)))))...)))))).	(((((((..(((((((........(((((....)))))......)))))))...((.....)).))))))).	(((((((..(((..((...(((((((..((((...........))))))))))...))..))))))))))).	(((((((..((((.......))))...........(((..(((.(.....).)))..)))....)))))))).	.........((((.......))))...(((((((.((((....))))(((((.......))))).)))))))
2ANN	/	/	/	/	/	/	/	/	/	/		((((...............))))	(((((..........))..)))..	(((((.........))...)))..	((((((....)).......))))	/	/	/	/	/	/
2B6G	/	/	/	/	/	/	/	/	/	/		(((((((.....)))))))	((..((....))..))...	.((((((.....))).)))	(((((....))..)))).	/	/	/	/	/	/
2B7G	/	/	/	/	/	/	/	/	/	/		(((((((.....)))))))	((..((....))..))...	.((((((.....))).)))	(((((....))..)))).	/	/	/	/	/	/
2DER	/	/	/	/	/	/	/	/	/	/		(((((((........(((.(((......((((((........))))))....))).))).......))))))))..	((((((...(((....)))........((((((.......))))))...(((((.......))))).))))))..	((((((...((((..........))))((((((.......))))))...(((((........))))).))))))..	((((((.(((((.(((((....((...))..)))))...))))).....(((((........))))).)))))))..	(((((((.(((((..(((....(((.......))))))..)))))....(((((.......))))).))))))..	(((((((......((.((....(((((((((........))))))......))))....)).)))))))))..	..(((((..(((....)))......((((((.......)))))))...)))))).......((((....))))...	((((((...........(((.(((.......)))......))))......(((((........)))))).))))))..	.........((((......(((.....((((((.......))))))...(((((........)))))).))))))).	((((((((..(((....)))...((.((((((.......))))))(((....)))....)))...))))))))..
2DET	/	/	/	/	/	/	/	/	/	/		..(((((........(((.(((......((((((........))))))....))).)))..........)))))	..((((...(((....)))........((((((.......))))))...(((((.......))))).))))	.....((((.....))))(((.....((((((.......))))))...((((.......)))))).))).	..((((...((((..........))))((((((.......))))))...(((((.......))))).))))	((((.((((.....))))....)))))((((((.......))))))).(((((........)))))......	..(((((..(((....)))........((((((.......))))))....)))))........(((....)))	..((((.(((((.(((((....((...))..)))))...))))).....(((((........))))).))))	..((((.(((((..(((....(((........))))))..)))))....(((((........))))).))))	..(((((.......((.((....((((((((((........))))))........))))....)).))))))	((((.((((.....))))....))))....(((.....((.....))....(((((........))))).))).

DNA model

PDB	mfold MFE	subopt #2	subopt #3	subopt #4	subopt #5	subopt #6	subopt #7	subopt #8	subopt #9	subopt #10
2DR2	/	/	/	/	/	/	/	/	/	/
2DRB	/	/	/	/	/	/	/	/	/	/
2DU3	/	/	/	/	/	/	/	/	/	/
2DU5	/	/	/	/	/	/	/	/	/	/
2DU6	/	/	/	/	/	/	/	/	/	/
2ES5	/	/	/	/	/	/	/	/	/	/
2EUY	/	/	/	/	/	/	/	/	/	/
2EVY	/	/	/	/	/	/	/	/	/	/
2F87	/	/	/	/	/	/	/	/	/	/
2F88	/	/	/	/	/	/	/	/	/	/
2FDT	/	/	/	/	/	/	/	/	/	/
2FEY	/	/	/	/	/	/	/	/	/	/
2FY1	/	/	/	/	/	/	/	/	/	/
2G1W	/	/	/	/	/	/	/	/	/	/
2GIO	/	/	/	/	/	/	/	/	/	/
2GIP	/	/	/	/	/	/	/	/	/	/
2GRW	/	/	/	/	/	/	/	/	/	/
2GV3	/	/	/	/	/	/	/	/	/	/
2GV4	/	/	/	/	/	/	/	/	/	/
2GVO	/	/	/	/	/	/	/	/	/	/
2HEM	/	/	/	/	/	/	/	/	/	/
2HGH	/	/	/	/	/	/	/	/	/	/
2HNS	/	/	/	/	/	/	/	/	/	/
2HUA	/	/	/	/	/	/	/	/	/	/
2HW8	/	/	/	/	/	/	/	/	/	/
2IHX	/	/	/	/	/	/	/	/	/	/
2IXY	/	/	/	/	/	/	/	/	/	/
2IXZ	/	/	/	/	/	/	/	/	/	/
2JPP	/	/	/	/	/	/	/	/	/	/
2JR4	/	/	/	/	/	/	/	/	/	/
2JSE	/	/	/	/	/	/	/	/	/	/
2JTP	/	/	/	/	/	/	/	/	/	/
2JWV	/	/	/	/	/	/	/	/	/	/
2JXV	/	/	/	/	/	/	/	/	/	/
2JYM	/	/	/	/	/	/	/	/	/	/

RNA model

PDB	mfold MFE	subopt #2	subopt #3	subopt #4	subopt #5	subopt #6	subopt #7	subopt #8	subopt #9	subopt #10
2DR2	(((((((..((((.......)))).(((((.......)))))....(((((.......))))))))))))....	(((((((..((((.......)))).((((....))))..........(((((.......)))))))))))))....	(((((((..((((.......)))).(((((.......))))).((((.((....))))))....))))))).....	(((((((..(((..((....((((((((((.......)))))....)))))......))..)))))))))....	(((((((..((((.......))).((.(((....((((....))))....))).)).......)))))))....	(((((((((((.((((((((...(((((.......)))))..))))).))))))..)))))...)))))....	(((((((..((((((........(((((....)))))......)))))))...((.....)).)))))))....	(((((((..(((..((...(((((((..((((...........))))))))))...))..)))))))))....	(((((((..((((.......))))...........(((..(((.(.....).)))..)))....)))))))....	.........((((.......))))...(((((((.((((....))))(((((.......))))).))))))...
2DRB	(((((((((((((.......))))))))))))....	.....((((((((.......))))))))((....)).	(((((((((.........))....))))))....	((...(((((((.......))))).....)).)).	.......((.((((((.........)))))).)).	.....(((((((..((....))..))..))).)).	(((..((((.........))))))..((....)).	((..((((....)))).....(((...)))..)).	.(((...)))((((((.........))))))....	(((.((((....))))...((.....))))))....
2DU3	(((((((....((((.((.(((((((....)))))))).)).))))..(((((.......))))))))))))	(((((((..((((((...)))))).(((((.......)))))......(((((.......))))))))))))	(((((((.(((....(((.(((((((....)))))))))))))....(((((.......))))))))))))	((((((((.((.((((.((.(((((((....))))))).)).)))).))))(((((((....))))))))))	((((((((......((((...((.((((((.......)))))).))))))....((....)).))))))))	((((((((((..((((((...((.((((((.......)))))).))))))...))....))..))))))))	(((((((.(((......)))....((((((.......)))))))....(((((.......))))))))))))	((((((((.((((((...))))))..((((......((.(((........))).))...)))).)))))))	(((((((..((((....(((....)))...))))..............(((((.......))))))))))))	((((((((.((((((...)))))).))......((.......))....(((((.......))))).))))))
2DU5	(((((((..((((((...)))))).(((((.......)))))......(((((.......))))))))))))	((((((((.((((((...))))))..((((.(((.((.((.(((.........))).)))))))))).))))))))	(((((((....((((.((.((((.((....)).)))).)).))))..(((((.......)))))))))))))	((((((((......((((...((.((((((.......))))))).))))))....((....)).))))))))	((((((((((..((((((...((.((((((.......)))))).))))))...))....))..)))))))))	(((((((.(((.......)))....((((((.......))))))....(((((.......))))))))))))	(((.....)))..((((.......((((((.......)))))))....(((((.......))))))))))...	(((((((.((..((((.(((....)))...))))....))......(((((.......))))))))))))	((((((((.((((((...))))))..((((((...................))))))......))))))))	(((((((.(((.((((((.((((...))))))))...)).)))....(((((.......))))))))))))
2DU6	(((((((..((((((...)))))).(((((.......)))))......(((((.......))))))))))))	((((((((.((((((...))))))..((((.((..((.(((.........))).))))).)))).))))))))	((((((((......((((...((.((((((.......)))))).))))))....((....)).))))))))	(((((((.(((.......)))....((((((.......))))))....(((((.......))))))))))))	((((((((((..((((((...((.((((((.......)))))).))))))...))....))..)))))))))	(((((((....((((.((.((((.((....)).)))).)).))))..(((((.......)))))))))))))	(((((((.((.((((..(((....)))...))))....)).......(((((.......))))))))))))	(((.....)))..((((.......((((((.......))))))....(((((.......)))))))))....	(((((((.(((.((((((.((((...))))))))...)).)))....(((((.......))))))))))))	((((((((.((((((...))))))..((((((.((((.....))))....))))))......)))))))))
2ES5	(((((((((.....)))))))))	((((((((....))..)))))).	((((((((..(...)))))))))	((((..((....))..))))....	((((((((..(.....))))))))	(((.(((((.....))).)))))	...((((((.....)))))..)).	((.((((((.....))).)))))	((((...))))............	...((((((.....)))..))).
2EUY	((((((.((..((((....)))).))..)))))	...........((((....))))(((....))).	(((..((((((....))).))).))........	..((...))..((((....))))(((....))).	(..((........))..)......(((....))).	(((...............)))..(((....))).	(((((..........((((......)))))))))	...........((..((((......))))..)).	(((...((.......)).))))..(((....))).	(((..((((((....))).)))).)))((....))
2EVY	(((((....)))))	/	/	/	/	/	/	/	/	/
2F87	((((....))))	/	/	/	/	/	/	/	/	/
2F88	((((((((((((((....)))))..))))))))))	(((((((((....((.....))...)))))))))	(((((((((((.......))......)))))))))	..((....((((((....)))))).))).......	........((((((....)))))).((...))..	..((...))(((((....)))))..........	(((((((...........((.....)))))))))	..((.......(((....)))))...........	/	/
2FDT	(((((((...((((((.....))))))..)))))))	........(((((...((((......))))))))))).	....((.((((((((((.....))))))...))))).	........(((((..((..........))...))))).	.((........((((((.....))))))...)).....	((((((.((.......))((((......))).))))))	...........(((((.....)))))(((...))).	((........((((((.....)))))))).......	((((((..(((......)))...((...))))))))	.(((....(((......)))...)))........
2FEY	(((((.(((..((((.((((.......)))))))))))))))))	.........((((((((((((.......)))))...)))))))..	(((((.(((((...)))))...(((((....)))))..)))))	(((((.(((..((((((((........)))..)))))))))))))	((.....))((((((((((((.......)))))...))))))..	(((((.(((((...))))).........(((....))))))))	(((((.(((((...)))))......(((((....))))))))))	((((((((.......(((((.......))))))))...)))))	(((((.((....))((((((.......))))))).....)))))	...............((.((((((((....)))))).))).))
2FY1	(((((((((.....)))))))))	(.((((((.....))))))).	((.((((((.....))))))).	((((((....))......))))	((.....))..........	(((...)))..........	/	/	/	/
2G1W	((((.....)))).........	((((....)))).........	.....((((........)))).	((((......))))........	....(((((........)))))	.((.((..........)).)).	(((.....)))..........	((((...))))...........	((.((((....))..))..)).	((((....))))(.....)...
2GIO	(((((..((.(((....))))..)))))	(((((((...(((....)))))..)))))	(((((((...(((....))))).))))	((((((((...((....))))))..)))))	(((((.....(((....)).)...)))))	(((((..(..(((....)))...)))))	((((((((...(....)))))..)))))	(((((.....(((....).))...)))))	(((((((((.((....))))))..)))))	(((((((((........)))...)))))
2GIP	(((((..((((((....)))))..)))))	((((((((((((((....))))))..)))))	(((((...((((....)).)).)..))))))	((((((((((((....)))))))))..))))	(((((...((((....).)))...)))))	(((((..(.(((....))))...))))))	(((((((((((.....)))))....)))))	(((((((((((.(...))))))..)))))	(((((...((.((....))))...)))))	(((((..((.((....)))).....)))))
2GRW	((((.((((....)))).))))	(.((.((((....)))).))).	((.(.((((....)))).))).	((((((((....))))..))))	((((...((......)).))))	((((((......))....))))	/	/	/	/
2GV3	(((((((........)))))))	..........(((....))..	((........)).........	/	/	/	/	/	/	/
2GV4	(((((.(((....))).)))))	(.(((.(((....))).)))).	((.((.(((....))).)))).	(((.(((((....))).)))))	((((((((....)))..)))))	((((((((....))))..))))	((((..(((....))..)))))	(((..((((....)))..))))	(((((....(......))))))	((((((......)....)))))
2GVO	((((((......))))))	((((((.....).)))))	(((((((...)).)))))	......((((....))))	/	/	/	/	/	/
2HEM	(((..((((((....)))))..)))	((...(((((....)))))..)).	.((..(((((....)))))...))	.....(((((....))....))).	..(...((((....)))))....	..((...(((....)))))....	.(....((((....)))))....	(.....((((....))))).....	.....(((((....))).....))	.((....(((....)))))....
2HGH	((((....((....))(((((((...((((.....))))......)))))))))))	((((....((....))((((((((.......(((......)))...)))))))))))	((......))....((((((((((...((((.....))))......))))))))).)).	(((((..........)))))......(((((((....((((....))))))))))...	.(((((..((.....))..)))).((((((....((((....)))))))))))..	((((...(((....((..((....)..)).....((((....)))).)))))))	((((...(((...........(((....))).....((((....)))).)))))))	((.(((.......))).)).....((((((.(((......)))....))))))..	((((...(((..((((((...(((....)))..))))).((.....)).)))))))	((((...(((.......(((.(((....))).....)))((.....)).)))))))
2HNS	(((((((((....)))))))))	((((((((...)...)))))))	((((((((...))...))))))	.((..((((....))))))...	.(((..(((....))))))...	...((((((....))))..)).	((((((......)....)))))	.((((.((...)).).))).....	...((((((....)))..)))).	...........(((....))).
2HUA	((((((((.((.....((....))....))....)))))))	((((((((.((...((((....)).)).))....)))))))	((((((((.((....))(.....)...........)))))))	.((.....))...((((((((............))))))))	((((((((.((..((.....))......))....)))))))	(((((((........(((....).....))....)))))))	((...))......((((((((............))))))))	(((((((((.......((....)).))........)))))))	((((((((..(((......)))............)))))))	....((....)).((((((((...........))))))))
2HW8	((((((((((..(((....)))......)))))))))	((((((((((...((........)).....))))))))))	(((((((((.....((((....)))).))))))))))	(((((((...((..((((....)))).)))))))))	(((((......(((....)))......)))))....	.((.(((((....))))).))..............	(((((((...........((.......)))))))))	........((.(((....))).((....))..)).	(((((.......)))))..................	........((.((.((((....))))......))))
2IHX	((((((((...((((.((((((.......)))))))))....(((((.....)))).((....))....))))))))	((((((((.((((.(((.((......)).))))))((.....((((......))))....)).......))))))))	((((((((...((((.((((((.......))))))))).((((((.((((.......)))).)).))).))))))))	((((((((..((((.((((((........))))))........((((......))))...))))......)))))))))	((((((((...((((.((((((........)))))))))....((((.....))))..(((......)))))))))))	((((((((..(((((((.((((((((.(((.............))).))).))).)))..))))......))))))))	((((((.((((.(((.((((((.......)))))))))))..((((((((......))))....))).))))))	((((((((......(((((......))))).((((........((((......))))..))))........))))))))	((((((((..((((...(((..(((....((.((........))))....)))..))).)))))......)))))))	((((((((.((((...........))))..((((........((((.....))))..))))........))))))))
2IXY	((((((((((......)))))).))))	(((((((((((...)).))))).))))	((((.(((((......))).)).))))	((((.((.((......)).)).))))	((((.(((((......))))))..)))	(((......)))..(((......))).	((.(((((((......))))))..))))	((((.....(((........)))))))	((((((.(((......))))))..))))	((((..........(((...)))))))
2IXZ	((...)).	/	/	/	/	/	/	/	/	/
2JPP	((((((((....))))))))	((.((((((....))))))).	.(((((((....))))).))	(((((((...))...)))))	((((((........)).))))	((((((....)....)))))	((((.(((....)).)))))	((((((....))....))))	(((.((((....)).)))))	((((((...).....)))))
2JR4	(((((.......)))))	(((.(((....))))))	....((((.....))))	.(..(((....)))..)	(((((..(...))))))	.((.(((....))).))	(((........)))...	...((((....))).).	..(((((....))).))	...((((.....)).))
2JSE	((((..(((....)))..))))	((((((.((....)))).))))	((((((.((....))))).)))	(..(((.((....)))))..).	(((...(((....))).)))..	(..((.((....)))..)).	(((..((((....)))..))))	((....(((....))).))....	((((..(((....))..)))))	.....((((....)))....).
2JTP	(((((((((((...(......)..)))))))))))	((((((((((...((.........))))))))))))	(((((((((.......))))).....)))......	....((((........))))...((.....))..	((.((((((.......)))))...))..........	((...((........))))..............	/	/	/	/
2JWV	(((((....((((....))))...)))))	(((((..(.((((....)))..))))))	(((((((..((((....))))))).)))))	((((((..(..((....)))..).)))))	((((((..(..(.....).)..).)))))	((((((..(..((....)).)..).)))))	((((..((.((((....)))))..))))))	((((((((..(((....))))).)))))	(((((...((.((....))))...)))))	....((((......))))...........
2JXV	((((((((..((((....))))...))))))))	((.((.(((.((((....)))))))).))...	...((..(((((((....))))....)))..))	((((((((........((.....))))))))))	((((......((((....))))).))))......	((((((...........(((....))))))))	........((((.(((......))).))))..	(((((.......((....)))).))).......	((..........((....))))...........	..........((.....(((....)))....))
2JYM	(((((.(((.....))))))))	(((((((.....))...)))))	(((((....((....)))))))	((((.......)))).......	(((.(......))))).......	.((.....))..........	.(((...)))..........	/	/	/

	DNA model									
PDB	mfold MFE	subopt #2	subopt #3	subopt #4	subopt #5	subopt #6	subopt #7	subopt #8	subopt #9	subopt #10
2K4C	/	/	/	/	/	/	/	/	/	/
2K5Z	/	/	/	/	/	/	/	/	/	/
2K66	/	/	/	/	/	/	/	/	/	/
2K95	/	/	/	/	/	/	/	/	/	/
2KD8	/	/	/	/	/	/	/	/	/	/
2KE6	/	/	/	/	/	/	/	/	/	/
2KHY	/	/	/	/	/	/	/	/	/	/
2KMJ	/	/	/	/	/	/	/	/	/	/
2KOC	/	/	/	/	/	/	/	/	/	/
2KPC	/	/	/	/	/	/	/	/	/	/
2KPD	/	/	/	/	/	/	/	/	/	/
2KPV	/	/	/	/	/	/	/	/	/	/
2KRL	/	/	/	/	/	/	/	/	/	/
2KRP	/	/	/	/	/	/	/	/	/	/
2KUR	/	/	/	/	/	/	/	/	/	/
2KUU	/	/	/	/	/	/	/	/	/	/
2KUV	/	/	/	/	/	/	/	/	/	/
2KUW	/	/	/	/	/	/	/	/	/	/
2KVN	/	/	/	/	/	/	/	/	/	/
2KZL	/	/	/	/	/	/	/	/	/	/
2L2J	/	/	/	/	/	/	/	/	/	/
2L3C	/	/	/	/	/	/	/	/	/	/
2L3E	/	/	/	/	/	/	/	/	/	/
2L3J	/	/	/	/	/	/	/	/	/	/
2L5Z	/	/	/	/	/	/	/	/	/	/
2L6I	/	/	/	/	/	/	/	/	/	/
2LAC	/	/	/	/	/	/	/	/	/	/
2LBJ	/	/	/	/	/	/	/	/	/	/
2LBK	/	/	/	/	/	/	/	/	/	/
2LBL	/	/	/	/	/	/	/	/	/	/
2LBS	/	/	/	/	/	/	/	/	/	/
2LC8	/	/	/	/	/	/	/	/	/	/
2LDL	/	/	/	/	/	/	/	/	/	/
2LDT	/	/	/	/	/	/	/	/	/	/
2LHP	/	/	/	/	/	/	/	/	/	/
2LI4	/	/	/	/	/	/	/	/	/	/

	RNA model									
PDB	mfold MFE	subopt #2	subopt #3	subopt #4	subopt #5	subopt #6	subopt #7	subopt #8	subopt #9	subopt #10
2K4C	(((((...........(((((.....))))).......((.((((((((....)))))).)).)).)))))))....	((((((..(((...)))(((((.....)))))......((.((((((((....)))))))).)).))))))....	(((((((...(((....(((((.....)))))......)))(((((((....))))))))...))))))).....	((((((...((((........))))..(((((((.....)))))))...(((((.......))))).))))))....	(((((((..((((........)))).(((........)))..((((((((....))))))))...))))))))....	((((((.(((........(((((.....)))))))....((.(((((((....)))))))).)).)))))).....	(((((((.(((...)))((((((((((((.(((((.....))))))))......)))).)))..)))...))))))))....	((((((........(((.....)))..((((((.....)))))))..(((((.......))))).)))))))....	((((((...((((.(((.....))).(((((.......)))))))).(((((.......))))).)))))))....	((((.....))))....(((((.....)))))......((.((((((.(((((.......)))))))).))).)).
2K5Z	((((((((((.((....)).))))).))))	((((.((((.((....))...)))))))))	((((((((.(((....).)))))).))))	((((.(((.(((........))))))))))	((((.((((...((....)).))))))))	(((.(((((.((....))...))))))))	((((((((((......)..))))).))))	((((((((((((....))..)))).))))	((((((...((....)).)))...))))	((((.((...((.........)))))))))
2K66	(((((((((....)))))))))	(.(((((((....))))))))).	((.((((((....))))))))).	(((((..(((....))))))))	((((..((((....))))))))	(..((((((....)))))))..	((..((((((....))))))))..	(((((..((....))..)))))	/	/
2K95	((((((....((......))....)))))).................	((((((....((......))....))))))...(((......)))....	((((((....((......))....))))))...........((....))	..((((.........((((.....))))......(((......))))))))).	(((......)))..((((((.......................))))))).	(((......)))..(((((((((..............))).)))))).	..((((((((...((((.....))))........)))))....)))).	..((((........((((.....))))..((..........)).)))).	..((((..(((...((((.....))))..........)))...)))).	.((((((((.....((((.....))))...)))))......))).....
2KD8	(((((((((....)))))))))	(.(((((((....))))))))).	((.((((((....))))))))).	....((.(((......))).))	(((((((....)....))))))	........(((........)))	....((....((....))..))	....((...((.....))..))	..........(((.......)))	....(((.((......))))).
2KE6	(((((((((((((((((((.........)))))))).))))).)))))))	((((((((((((((((((((....))..))))))).))))).)))))))	(((((((((((((.(((....((((...))))))).))))).)))))))	(((((((((((..((((((........))))))))....)))))))))	((((((((((((((...............))....))))).)))))))	((((((((((((((.........))...........))))).)))))))	(((((((((((((((((((...)))........))).))))).)))))))	((((((((((((((.....))...............))))).)))))))	((((((((((((.(((........((...))))).))))).))))))))	(((((((((.((....))..((((...))))........)))))))))
2KHY	((((((.......((((....))))........))))))	((((((....(((((((....)))))...))).))))))	.(((((..(((.....))).)))))............	....((........((((....))))))...........	...((......)).((((....))))............	............((((....))))((.......))..	.........((..((((....))))..........)).	((((((....(((....((((...))))))).))))))	((((((..(((......))))..((......)).))))))	((((((((((((.....))..)).........))))))
2KMJ	((((((((..((((....)))))))))))))	(((((((((((.....)))...)))))))	.....((((((((....))))...))))	(((((((((((...))))...)))))))	(((((((....((....))))).))))	(((((....((((....))..)))))))	(((((....((((....)..))))))))	((.......((((....)))))).....	.....((((((((....))))...)))))	((((.((.........))))).........
2KOC	(((((....)))))	/	/	/	/	/	/	/	/	/
2KPC	.(((((.....))))).	((((((.....))))))	.((((..(...))))).	/	/	/	/	/	/	/
2KPD	.((((........)))).	(((((........)))))	.((((..(...))))).	/	/	/	/	/	/	/
2KPV	((((((((..((((....))))...)))).))))	((((...(((((((....))))....))).))))	...(((((..((((....))))...)))....))	((((...((((..((.......))...)))).))))	...((.(((.((((....))))).......)))))	(((((((.......(((......))))))).))))	(((((.......((....))))).)))........	...((.(((..((.(((......))).)))))))	((...........((....))))...........	..........((.....(((....)))....))
2KRL	((((((((....))))))))......(((((((((..........)))))))))...((((.(((((......(((....)))......))))).)))).....	...(((..(((((.........)))))(((((((((.........)))))))))...((((.(((((......(((....)))......))))).)))).)))	((((((((....))))))))......((((.....(((((....)))))))))...((((.(((((.......(((....)))......))))).))))....	((.((((((((....))))..)))).(((((((((.........))))))))))...((((.(((((......(((....)))......))))).))))).)).	...(((.((.((((((((((((((.......))).)))))....)))))))))..((((.(((((......(((....)))......))))).)))).)))	(((.(((.((..(((.(((((.....((((((((.........))))))))))..(((....)))......(((....)))))))).))))).))).))).	...(((.((.((((((((((((.((....))))))))..........))))))))..((((.(((((......(((....)))......))))).)))).)))	(((.(((.((..(((.(((((.((..(((((((((.........)))))))))).(((((.............)))))..)).))))).))))).))).))).	((..(((((((((........)))))...))))..(((((....)))))......((((.((((((......(((....)))......))))).)))).)).	((((((((....))))))))......((((((((.(((((....)))))((((..(((....)))......(((....))).)))))..))))).))))....
2KRP	(((((.......)))))	.(((....))).......	/	/	/	/	/	/	/	/
2KUR	(((((((((((((((((((.........)))))))).))))).)))))))	((((((((((((((((((((....))..))))))).))))).)))))))	(((((((((((..((((((........))))))))....)))))))))	(((((((((((((.......((((((...)))))...))))).)))))))	((((((((((((((......)))...........))))).)))))))	((((((((((((((..............)))....))))).)))))))	((((((((((((...((((.............))).))))).)))))))	((((((((((((..((((...))))..........))))).)))))))	((((((((((((........(((........))).))))).))))))	(((((((((....((....((((((...)))))...))..)))))))))
2KUU	(((((((((((((((((((.........)))))))).))))).)))))))	((((((((((((((((((((....))..))))))).))))).)))))))	(((((((((((..((((((........))))))))....)))))))))	((((((((((((.((((....(((...))).)))))))))).)))))))	(((((((((...((((((((........))))......))))))))))))	((((((...((.((((((((........)))))))......))))))))	(((((((((((((...((......))...))))........))))))))	(((((((((((((((........))............))))).)))))))	.((......)).(((((((..........))))))).............	((.....(((((((((((((.......))))))).))))).)).....
2KUV	(((((((((((((((((((.........)))))))).))))).)))))))	((((((((((((((((((((....))..))))))).))))).)))))))	(((((((((((((.(((....((((...))))))).))))).)))))))	(((((((((((..((((((........))))))))....)))))))))	((((((((((((((...............))....))))).)))))))	((((((((((((((.........))...........))))).)))))))	(((((((((((((((((((...)))........))).))))).)))))))	((((((((((((((.....))...............))))).)))))))	((((((((((((.(((........((...))))).))))).))))))))	(((((((((.((....))..((((...))))........)))))))))
2KUW	(((((((((((((((((((.........)))))))).)))).))))))))	((((((((((((((((((((....))..))))))).)))).))))))))	(((((((((((((.(((....((((...))))))).)))).))))))))	(((((((((((..((((((........))))))))....)))))))))	((((((((((((((...............))....)))).))))))))	((((((((((((((.........))...........)))).))))))))	(((((((((((((((((((...)))........))).)))).))))))))	((((((((((((((.....))...............)))).))))))))	((((((((((((.(((........((...))))).)))).)))))))))	(((((((((.((....))..((((...))))........)))))))))
2KVN	(((((......))))).	((((((...)).)))).	(((((...))).))).	/	/	/	/	/	/	/
2KZL	((((((......((((.......((((....))))........))))...))))))	((((((......((((....(((((((....))))...))).))))...))))))	((((((.....(((......))).((((....))))..............))))))	((((((...(((((((..........))))))).((......)).....))))))	(((.(((...(((......))).((((....))))...))).))).........	((((((......((((....(((....((((...))))))).))))...))))))	((((((.....(((.((((((.....)))..))))))...((....)).))))))	..............(((.......((((....)))).....((....))..)))..	((((((.(((.(((.......))).)))......((......))....))))))	((((((....(((.......)))......(((..............))))))))))
2L2J	((((.(((((((((.((((.....)))).)))))))))).))))	((...((((((((((.((((.....)))).)))))))))))...	.....((((((((((.((((.....)))).))))))......)))	.....((((((((((.((((.....))))......)))))).)))	(((.((((((((.....((....))).)))))))))......	((((.((((((((((.....((...)).)))))))))).))))	(((.(((((((((((.....))))....)))))))))......	((((.((((((.((...))....((....)).)))))).))))	((((.((((((.....))))...((....))....))).))))	........((((((.((((.....)))).)))).........))
2L3C	((((.(((((((((((....)))))))))))).))))	((((.(((((((........)))...)))).))))	....((((((((((((....)))))))...))))).	((((.(((((((........))))))...).))))	..((...((((((((....))))))))))....	.((((..((((((........)))))))))......	((..(((((....)))))))................	/	/	/
2L3E	((((((.....((((((....))))))))))))))	((((((...(((..((((....))))))))))))))	.......(((..((((((....))))))...))).	(((....)))..((((((....)))))).......	(((((((.((..(((.....)))...))))))))	(((....)))....((((....))))((.....))	((((((((((..........))))....))))))	(((((((((.......))..((...)).)))))))	.(((...((.......))...)))...........	(((....)))..........(((........)))
2L3J	(((((((.(((((((((.(((.((((((((((((......))))))))))).))).))))))))).)))))))	(((((((.(((((((((..(((((((.....)))))))..(((....)))......))))))))).)))))))	(((((((.(((((((((.....(.....)...((((((...........)))))).))))))))).)))))))	(((((((.(((((((((.(((.....((((((((......)))))))))......)))))))).)))))))	(((((((.(((((((((......((((((((((......))))))))......))).))))))))).)))))))	(((((((.((((((((((((((((((((....))))))....)))...........))))))))).)))))))	........((((((((((.(((.(((((((((((.....)))))))))))).))).))))))........)))	(((((((.((((((((((.(((.((((..............))).......).))).))))))))).)))))))	..((((......))))......((((((((((.....))))))))))(((((...........))))).	(((.................(((((((....)))))))......((((..(((.......)))..)))))))
2L5Z	((((((...(((....))).))))))	((((((..((......))..))))))	(((((((...((....)).))))))	((((((((...(....))).))))))	.....((..(((....)))...))..	((((((.....((....)).))))))	(((((((.........))...)))))	.......(((((....))....)))).	.......(((.((.......)))))).	..((....))((....))........
2L6I	(((((.....))))).	/	/	/	/	/	/	/	/	/
2LAC	(((((.......)))))	((((........)))).	(((.........)))..	.(((..........)))	/	/	/	/	/	/
2LBJ	(((((((...)))))))	(((((.(....))))))	.((((((...)))).))	.(((...))).......	(((.(((....))))))	((.((((...)))))).	((......))......	/	/	/
2LBK	((((((.....))))))	(((.....))).....	((((...))))......	.(((((.....)))).)	(((....))).......	((.(((.....))))).	.((......)).....	((((.((....))))))	(((.((......))))).	.(((((.....))).))
2LBL	(((....))).......	(((((.......)))))	.((.....)).......	.(((...))).......	((((.((....))))))	/	/	/	/	/
2LBS	((((((((((((((....))))))))))))))))	((....))..((((....)))).........	(((((((...((((....))))))...)))))	(((((...((((((....)))))...)))))))	.((((.....))))................	((....))..........((........))..	/	/	/	/
2LC8	.(((.((((((((........))))).)))..)))..(((((......)))))...	.....((((.....))))((((((((....(((.....))).....)))))))))..	((((((((...((....)).)))))).)).......(((((......)))))...	..............((((((((((....))))..............))))))).	.(((.((((((((........)))).))))......(((.......))).))).	((((........)).)).((((((((....(((.....)))......))))))))..	..............(((((((..(((.....((.....)))))......))))))).	..((((((...((....)).))))).(((..(((..........)))..)))...	.(((.((((((((........)))).)))..)))..........(((....))).	....((((..((....))..((((....))))...)))))....(((....))).
2LDL	((((((.((((.....)))).))))))	((((((.((.........))..))))))	(((((((((.....)))....))))))	(.((((.((((.....)))).))))).	(((((((((.....)))))....)))))	((.(((.((((.....)))).))))).	((((((....(.........)))))))	((((((....(.((....)))))))))	((((((....(.((...)).)))))))	(((((....((.........)))))))
2LDT	(((((((..((((....))))...)))))))	(((((((......((...))....)))))))	(((......((((....))))....)))...	((((.((......))..(((....)))))))	.((........))....((........))..	/	/	/	/	/
2LHP	((((((((.((((((....))))))..))))))))	(((((((((((...(....))))....)))))))))	(((((((((....(((....)...))..)))))))))	(((.......((((((....)))))))....))).....	(((((((((((((....))).........))))))))))	((((((......((((....))))))........))))	........((((((((....))))).........)).	((((((((((.(((...))).)))........)))))))	((((((((((.......((.......)))))))))))))	(((((((..(((....))).((......)))))))))
2LI4	(((((((((((((((....))))))))))))))))	(((((((((....((......))))))))))))	.((....((((((((....))))))))))).....	.....(((((((((....))))))....))).	(((((((((...))...............)))))))	..(((......)))......(((.....)))..	(((((.........((..........)))))))	(((((.....))).......))..........	..........((.......(((.....)))))	/

	DNA model											RNA model									
PDB	mfold MFE	subopt #2	subopt #3	subopt #4	subopt #5	subopt #6	subopt #7	subopt #8	subopt #9	subopt #10		mfold MFE	subopt #2	subopt #3	subopt #4	subopt #5	subopt #6	subopt #7	subopt #8	subopt #9	subopt #10
2LJJ	/	/	/	/	/	/	/	/	/	/		(((((..(((((.....))))))))))	(((((..((.........))..)))))	....((.(((((.....))))).))..	(((((((............)).)))))	...((..(((((.....))))).))..	(((((....((.((....)))))))))	.......((.((((......))))).	.((....)).((((......))))...	(((((......(((....))).)))))	.((....))...(((........))).
2LK3	/	/	/	/	/	/	/	/	/	/		((((..(((((....)))))))))	(((.(.(((((....)))))))))	((.((.(((((....)))))))))	((((.......((.....))))))	((((...((.....))....))))	((((((.........))...))))	((((((.........)))...)))	/	/	/
2LKR	/	/	/	/	/	/	/	/	/	/		((((((((.....((((((((.((((.(((.....)))..)))).......(((((((((((....)))))))))))).......)))..)))))......)))).))))	((((((((.....(((((.((((.....)))).........(((.....((((((((((((....))))))))))))).....)))....)))))......))))).))))	((((((((.....(((((.((.(((.(((.....)))..)))).))......((((((((((....))))))))))))(((....)))...))))).....)))).))))	......(((((..((((((((.((((.(((.....)))..))))......((((((((((((....))))))))))))).......)))..))))).........))))).	.((.((((.....((((((((.((((.(((.....)))..)))).......((((((((((((....))))))))))))).......)))..)))).)))).)).......	((((((((.....(((((.((((.....)))).((((......(((.....(((((((((((....))))))))))).....))))))))))).......)))).))))	((((((((.....(((((.((((.....))))...((((((...)))))..((((((((((((....))))))))))(((....)))...)))))......)))).))))	((((((((.....(((((.((((.....))))..((((.............((((((((((((...))))))))))))))).........)))))......))))).))))	......((((...((........))..((((((...(((.(((((.....(((((((((((...))))))))))))......))).))..)))..))))))..)))).	..................((((.....(((((...(((.(((((.....(((((((((((....))))))))).....))).)).)))..))))))...)))).
2LP9	/	/	/	/	/	/	/	/	/	/		(((((......)))))	/	/	/	/	/	/	/	/	/
2LPA	/	/	/	/	/	/	/	/	/	/		((((((...))))))	/	/	/	/	/	/	/	/	/
2LQZ	/	/	/	/	/	/	/	/	/	/		((((((...((((....))))))))))	((((.(...((((....)))).)))))	((....)).((((....)))).......	(((((....((((....)).)))))))	((((((....(((....))))).))))	((((((((........))...))))))	(.((((...((((....))))))))).	((.(((...((((....)))))))).	(((.((...((((....)))).)))))	((((.....((((....)))..)))))
2LU0	/	/	/	/	/	/	/	/	/	/		(((((..((((.((....))...(((((....)))))))))...)))))	(((((..(.....)...(((...(((((....)))))..)))..)))))	(((((..((((...((.......)).((....))...))))...)))))	(((((....((...)).(((...(((((....)))))..)))..)))))	...........(((...(((...(((((....)))))..)))..)))..	(((....(((..((....))...(((((....)))))......))))))	(((((..(((.......)))...(((((....)))))........)))))	(((((..((((..((((.((......))))))......)))).....)))))	..............((((((...(((((....)))))..))))...))).	........(((.(....))).(((((....)))))((((...)))).
2LUB	/	/	/	/	/	/	/	/	/	/		((((((((.((((((....))))))..))))))))	(((((((((((...(....))))....)))))))))	(((((((((....(((.....)...)))..)))))))))	(((........((((((....)))))).....))).....	(((((((((((((....))).........))))))))))	(((((((((.(((...))).)).........)))))))	((((((.......(((((........))))))))))	........((((((....)))))..........)).	((((((.......((((....))))))........))))	(((.((..((((....)))).)))))...........
2LUN	/	/	/	/	/	/	/	/	/	/		((((....((((....))))....))))	((((..((((((....)))..)))))))	((((.(((.(((....))))))..))))	((((..((((((....))..))))))))	((((..((((((....)..))))))))	.((((....))))..(((......)).	(((((....))))).(((......))).	((.(....((((....))))......))).	.(((....((((....))))......).))	((((.(...((((....))))....))))
2LUP	/	/	/	/	/	/	/	/	/	/		(((((((((((((((....))))))))))))))))	((....))..((((....))))..........	(((((((...((((....)))))))...)))))	(((((...((((((....))))....)))))))	((....))..........(((........))).	.((((.....))))..................	/	/	/	/
2LV0	/	/	/	/	/	/	/	/	/	/		(((((((((........)))))))))	((((((..(((....)))))))))	((((((((.((...)))))))))))	((.(((((........)))))))).	.(((((((........))))).))	(((((...(((....)).))))))	((((...((((....)).))))))	(((((......((....))))))))	((((......(((....)))))))	((((((.......(...))))))))
2M12	/	/	/	/	/	/	/	/	/	/		((((((...........))))))	((((((.((......))))))))	((((((.(((....))))))))))	(((((((((...))))..))))))	(((((((((...))))..)))))	((((((..(........)))))))	(((((..((........)))))))	((((((.......)....)))))	((((.((((...)))).)).)).	((((((.......))....))))
2M21	/	/	/	/	/	/	/	/	/	/		(((((((.......)))))))	(((((((...))....)))))	(((((((...)))....))))	(((((........)...))))	(((((((...))))....)))	/	/	/	/	/
2M22	/	/	/	/	/	/	/	/	/	/		(((((.((((...)))).)))))	(((((((.....))....)))))	((((.....((......))))))	.(((....)))............	/	/	/	/	/	/
2M24	/	/	/	/	/	/	/	/	/	/		((((((((((...........))))))))))	((((((((((.((......)))))))))))))	(((((((((.(((....))))))))))))	(((((((((..(.......))))))))))	((((((((((.......)..)))))))))	(((((((......((.....))))))))))	((((((((..((........))))))))))	((((((((((.......))..))))))))	(((((((((....))).......))))))	(((((((((......)).....))))))))
2M4W	/	/	/	/	/	/	/	/	/	/		((((((....))).)))	(..(((....)))..).	((.(((....))).)).	((.(((....))..)))	/	/	/	/	/	/
2M57	/	/	/	/	/	/	/	/	/	/		(((.(((((((((((.....)))))..))))).)))	(((.(((((((..((.......)).)))))))).)))	...((((....))))...((((......))))...	(((((((....))))...)))..............	.....(((..((((......)))))))).........	(((.((((((((....))........))))))).)))	...((.....))...((.((((......)))).))	(((((((....))))...)))....((....))..	(((((..(((...)))))))).............	...((((....)))).((.........))......
2M5U	/	/	/	/	/	/	/	/	/	/		(((((((((....)))))))))	((((((((.....)).)))))))	((((((....)....))))))	(((((((....))....)))))	..........(((......)))	(((((......(....))))))	..........((((....))))	((((......((....))))))	..(((...)))...........	/
2M8K	/	/	/	/	/	/	/	/	/	/		..........(((((((((((.((..........)).)))))))))))	..........((((((((((((....((......))..)))))))))))	..........((((((((((((.(((.....)).)).)))))))))))	((...))...((((((((((((.((..........)).)))))))))))	.....((....)).(((((((.((..........)).)))))))... .	(((((.................)))))...................	((..((........)).....))........................	..........(((((((((............(((....))))))))))))	(((((......((....))....)))))...................	/
2MEQ	/	/	/	/	/	/	/	/	/	/		(((((((.......))))))	((((((((....)).)))))	((((((....)...)))))	/	/	/	/	/	/	/
2MF0	/	/	/	/	/	/	/	/	/	/		((((........))))..((((((......))))))......(((((.....)))))(((((.....)))))	.((((.............((((((........))))))...((.(((((.....))))))))........))))..	.((((...(((.((.((....((((((.......))))))......(((((.....))))))))))....))))..	((((........))))....((((...(((....)))..((.(((((.....)))))).........)))).	((((........))))..(((((((......))))))......(((((...(((....))).......))))).	((((........))))...((.....))...((.(((..((.(((((.....)))))...))..))).))..	.(((..............((((((......))))))...))).((((.....)))).(((((.....)))))	((((........)))..((..((.....((((((........))))))...)).))...(((((.....)))))	((((........))))....((((......((((((.......(((((.....))))))))))......)))).	.(((((.((..........)).))...))).((.((..((.(((((.....)))))...))..))).))..
2MFC	/	/	/	/	/	/	/	/	/	/		(((((((.........)))))))	((.((((.........)))))).	((((((((.....)).....)))))	.((((((.........)))).))	(((((((....))).....))))	(((((((....))))....)))	(((((((...)......))))))	(((((((.....).....)))))	(((((((....)))..)).)).	/
2MFD	/	/	/	/	/	/	/	/	/	/		(((((((.....)))))))	(((....)))..........	((((((......)).))))	/	/	/	/	/	/	/
2MFE	/	/	/	/	/	/	/	/	/	/		((((((((......))))))))	.(((((((......))))).))	((.(((((......))))))).	((((((((...))...))))))	..........((((....))))	.((...))..((((....))))	((....))..((((....))))	(((((...(.......))))))	((((...((.......))))))	...((.....))..........
2MFF	/	/	/	/	/	/	/	/	/	/		((((((((.....))))))))	((.(((((.....))))))).	(((.((((.....))))))).	.(((((((.....))))).))	(..(((((.....))))))..	((..((((.....))))))..	((((((....)).....))))	.........(((.....))).	/	/
2MFG	/	/	/	/	/	/	/	/	/	/		((((((((.....))))))))	((.(((((.....))))))).	.(((((((.....))))).))	(((((...(.......))))))	(((...(((......))))))	..(((.....)))........	(((((........))...)))	(((((........)))..)).	/	/
2MHI	/	/	/	/	/	/	/	/	/	/		((((((..(((((.((((...))))))))).((((.....))))...))))))	((((((...((((((.....)))))).(((((...)))))........))))))	((((((((((.((.....)))))).........((((.....))))...))))))	(((((((..((((..(((((.((....))...)))))......)))))))))))	(((..(((.((((((.....))))))).)))......)))...............	.....(((.((((((.....)))))).)))..(((..((........)).))).	(((((((..((((...((..(((.((.....)))))).))..))))))))))	(((((((.((((((((((......)))......))..))))....).)))))))	/	/
2MNC	/	/	/	/	/	/	/	/	/	/		((((.(......)))))	(((.((......)))))	(((.(((...))))))	/	/	/	/	/	/	/
2MQT	/	/	/	/	/	/	/	/	/	/		(((((.((((((((...((((((..((((.((((....))))))))..)))))))))))))).)))))	......((((..((((..(((((..((((.((((....)))))))))..)))))..))))......))))	....((((....)))).((((((..((((.((((....))))))))..))))))..((((....))))	(((((.((((((((....(((.....))).((((....)))).............))))))))).)))))	((((.(((....)))(..((((((..((((.((((....))))))))..)))))..)..))))......	(((((.((((((((..((((.((((((((.....))))......))).))))...)))))))).)))))	((((((((....)))).(((((((..((((.((((....)))))))))..))........))))..))))	(((((.((((((((.(((.....(((((.....)))))))))).((....))....))))))))).)))))	(((((.((((((((.((((.((..........)))))).(..(...)..)....)))))))).)))))	....(((((.(((((((((((((......)))...)))).))))).))))).......((((....))))
2MS0	/	/	/	/	/	/	/	/	/	/		((((((((.......(((.....((((((.......)))))))..)))(((.......))).))))))))	(((((....((((.(((((.(((((((((((........)))))))..........))).)))))))))))))))	(((((((..(((..........)))((((((........))))))....((((((.......))))))))))))	(((((((((..((..(((.....((((((((........)))))))..)).))..))........)))))))	((((((((((.....(..(..(((((((((........)))))).)))....)..).....))).)))))))	(((((....((((.(((((.(((.....(((..(((.........)))..)))))).)))))))))))))))	(((((((.(.((((((((........)).)))))).).........((((((........))))))))))))	..(((((...((((((((..........)).))))))..)))))..((((.(((.......))).))))).....	(((((...........(((.....(((((((........)))))))..)))..((((........)))))))))	((((....))))....(((.....((((((........)))))).((((.(((......))).))))..))))
2MTJ	/	/	/	/	/	/	/	/	/	/		(((((..(((((....))))).(((((....)))))......))))).	((((((((((((....))))).(((((....))))).))..))))).	(((((..(((((....)))))((((((....))))..))..))))).	(((((..(((....)))......(((((....)))))......))))).	((((.....))))..((((((((((((....))))..)))).)))).	(((((..(((((....)))))....((((....))))....))))).	(((((......((......)).(((((....)))))......))))).	(((((...((.((......)).)).((....))........))))).	(((((..(((((....)))))((....))............)))).	((.....))......((((((((((((....))))..)))).)))).
2MXJ	/	/	/	/	/	/	/	/	/	/		((......)).	/	/	/	/	/	/	/	/	/
2MXL	/	/	/	/	/	/	/	/	/	/		..(((((((..((((((......)).))))..)))))))	..(((((((...((.(((........))).)))))))))	..((((((.((....))....((........))))))))	..(((((((..((..........)).......)))))))	..((((.((..((..........)).)).))))......	..((((((.((....))..........((....))))))))	((.........))..(((........)))..........	...........((((((((......)).)))).((....))	((.((....)))..(((..........))).........	..(((((..((....))......)))))..........
2N0R	/	/	/	/	/	/	/	/	/	/		....((((....)))).......	.(..((((....))))...)...	..(.((((....))))..)....	.(..((((....)))))......	.((..(((....)))))......	..(.((((....))))......).	....((((....))))(....).	.((.((((....))))......))	.....(((....)))((....))	.((..((....))..))......

	DNA model											RNA model									
PDB	mfold MFE	subopt #2	subopt #3	subopt #4	subopt #5	subopt #6	subopt #7	subopt #8	subopt #9	subopt #10		mfold MFE	subopt #2	subopt #3	subopt #4	subopt #5	subopt #6	subopt #7	subopt #8	subopt #9	subopt #10
2N1Q	/	/	/	/	/	/	/	/	/	/		((..((((((..........(((((((....)))))))....((((((..(((....((((.(((((((....)))))..)).)))).)))......(((((....)))))..........(((((....)))))...)))))))))))..)).	((..((((((..........(((((((....)))))))....((((((...((....((((.((((((((....)))))..)).)))).)))((((((((....))).))))..........(((((....))))))...))))))))))))..)).	((..((((((..........(((((((....)))))))....((((((....((((((((.(((((((....)))))..)).)))))....(((((((....))).)))).....))))).(((((....)))))...))))))))))))..)).	((..(((((((.((((((..(((((((....)))))))(((....))).))))))..((((.(((((((....)))))..)).))))....(((((((....))).)))).....((((..(((((....)))))..)))).)))))))..)).	((..(((((((.(((((((.(((((((....)))))))...(((..(((.(((....((((.((((((....)))))..)).))))..)).)))..)))...)))))).....((((((....)))))......)).)))))))..)).	((..(((((((.((((((..(((((((....)))))))(((....))).))))))..(((..(((((((....))))))...(((((...(((((((....))).)))).....)))).(((((....)))))...))).....)))))))..)).	((..(((((((((((((....(((((((....)))))))(((....)))..(((....((((.(((((((....)))))..)).))))...))))))).((((....)))).....((((..(((((....))))))..))))..)))))))..)).	((..(((((((.((......(((((((....)))))))(((((...(((.(((....((((.((((((....)))))..)).))))..))).))).(((((....)))))...)))))..(((((....)))))......)).))))))..)).	((..(((((((.((......(((((((....)))))))....(((.((..(((((..((((.(((((((....)))))..)).))))....(((((((....))).))))............)).)))..))..)))....)).)))))))..)).	((..(((((((.((......(((((((....)))))))....((((((..............(((((((....)))))))...)))))))).(((((((....))).)))).............(((((....))))).....)).)))))))..)).
2N2O	/	/	/	/	/	/	/	/	/	/		.........((((....))))..	...(((((((...)))))))...	((.(((((((...))))))))).	..((((((((...)))))).)).	/	/	/	/	/	/
2N2P	/	/	/	/	/	/	/	/	/	/		...(((((((...)))))))...	((.(((((((...))))))))).	((((.....))))..........	..((((((((...)))))).)).	.........((.......))....	/	/	/	/	/
2N3O	/	/	/	/	/	/	/	/	/	/		(((((((((.....)))))))))	((((((((.....)).)))))))	((.((((((.....))))))))).	(((.(((((.....))))))))).	.((((((((.....)))))).))	((((((((...)..))))))))	((((((((.....)..))))))))	((((.((((.....))).)))))	(..((((((.....)))))))...	((((.((((.....)).))))))
2N3Q	/	/	/	/	/	/	/	/	/	/		(((((.(((..((((((....))).)))(((..((((....)))))))).....)))))))).	(((((.(((..((((((....))).)))......((((........)))))...)))))))).	(((((.(((........((((......))))...((((....)))).........)))))))).	(((((.(((........(((.(.((.(((((......))))).)).)))).....)))))))).	(((((.(....)....(((((.((....))...((((....))))....))))).))))).	(((((.(((........((((((......)).))))((....))..........)))))))).	(((((..((...))..)).)))((((.((((..((((....))))......))))..)))).	(((((.(((..((((((....))).)))((.........)))...........)))))))).	(((((.(((........))).........((((..((((....))))......)))).))))).	(((((.(((........(((......(((((......))))))).........)))))))).
2N4L	/	/	/	/	/	/	/	/	/	/		((((((((((((((......((((((.....))))))...)))))))))))))))	(((((((((((((((.((((.......))))...........))))))))))))))))	((((((((((((((((.(((((.......))))(((...))).)))))))))))))))	((((((((((((((((((((......)))..............)))))))))))))))	((((((((((((((.........(((..............)))))))))))))))))))	((((((((((..(((((......))))..))....(......)...))))))))))	..........((((......((((((......))))))...))))(((....)))	...........((((((......))))))(((((...............))))).	....(((.........)))..((((((......)))))))........((((....)))	((((((((.........))).)))))...................(((....)))
2N6S	/	/	/	/	/	/	/	/	/	/		((((((((((((((((((...)).))))))))))))))))	(((((((((((((....)))........))))))))))	((((((((((((.........((...))))))))))))))	(((((((....(((.............))))))))))	((.(((....))).)).)...(((...........)))	(((((((..(((....)))..((....)))))))))	(((....((((((((((...)).)))))....)))))	(((((....((((((((...)).)))))))....)))	((((((((((((((....))))..)))......))))))	/
2N6T	/	/	/	/	/	/	/	/	/	/		((((.((((((((..((((....))))...)))))))))))))	.....(((((((((..((((....))))...))))))...)).	((((.((((((((((.........((....))))))))))))))	((((((((........((((....)))..))).))))))....	(((..............((((....))))))............	((((((..((....))(((....)))..........))))))	(((((((.........((((....)))))).........)))))	((((.(((((((((((........)).....)))))))))))	(((((((......)).(((....)))............)))))	(((((((.((....)).))...((....)).......)))))
2N6W	/	/	/	/	/	/	/	/	/	/		(((((((((((((..(.((((((((..((((....))))...)))))))))..)))))))))))))	((((((((((((((..(((((.......((((....))))..)))......))..)))))))))))))))	((((((((((((((.........((((((..((((....)))).........)))))))))))))))))))	..........((((..((((((((((((..((((....))))...))))))))........)))..)))).	((((((((((((((.....(((.......((((....))))..))).))))))).........))))))	(((((((((((((.((.(((.((....)).))).)).)).................))))))))))	((((((((((((((((.....))).....((((....))))..))))))..((((....)))))))))))	(((.........((((.....))))....((((....)))))))......(((((....))))).....	(((((((((((............))))...((((....))))........................))))))	(((..(((...((((.....))))...)))..)))............(((((....)))))....
2N6X	/	/	/	/	/	/	/	/	/	/		((((((((..(.((((((((....)))))))))..))))))))	(((((((..((((((((((((....))))))))).))))))))))	.....(((.((((((((((((....))))))))...))).))).	((((.(((......(((((((....)))))))))).))))....	(((((((.(((......(((....))))))......))))))))	..........((((((((((((....)))))))).......))).	.((....))...((((((((....)))))))............	((((((((..(((((....)))..........))..))))))))	((((((((.......((((((....)))....))).))))))))	............((((((((....))))))).((....))..
2N7M	/	/	/	/	/	/	/	/	/	/		(((((((((((.(((((..(((((.(((.....(((....)))..))))))))...(((((((....))))))).))))).)))))))))))	(((((((((((.(((((....(((((((((..(((((.((.........)).))))))..)))))))......))..))))).))))))))))))	(((((((((((.(((((...(((.......))).(((....)))..............((((((((....))))))))..)))))).))))))))))))	(((((((((((.(((((........((((((..(((....)))...))).))...((((((((....))))))).))))).)))))))))))))	(((((((((((.(((((..(((...(((.......))).)))...............((((((((....)))))))).))))).)))))))))))	(((((((((((.(((((((.(((......))).(((....)))...)))......((((((((....))))))))).)))).)))))))))))	(((((((((((.((((....(((......))).(((....)))....(((((...(((((....))))))...))))))))).)))))))))))	(((((((((((.(((((...(((......))).(((....))).(((......)))(((((((....))))))).))))).)))))))))))	(((((((((((.(((((.(((((....(((..(((((.((.......)).)))))..)))))))..........))))).))))))))))	(((((((((((.((((.(((....)))((((..(((....)))....))))......((((((((....))))))).)))).))))))))))
2N7X	/	/	/	/	/	/	/	/	/	/		(((((..((.....))..)))))	(((((.((......))..)))))	(((((..(((...)))..)))))	(((((....((.....)))))))	(((((((.......)))))..)))	((.((((.......))))))...	/	/	/	/
2N82	/	/	/	/	/	/	/	/	/	/		(((((..((.....))..)))))	(((((.((......))..)))))	(((((..(((...)))..)))))	(((((....((.....)))))))	(((((((.......)))))..)))	((.((((.......))))))...	/	/	/	/
2N8V	/	/	/	/	/	/	/	/	/	/		(((((.((((((((....))).))))).(((((((((......))))))))))))))))..................	(((((.(((((.....))))).......((((((((......)))))))))))))..............	......((((((((....)).))))))....((((((((.......(((....)))...))))))))...	(((((.......(((........)))..((((((((......))))))))))))...............	....(((..(((.(((....((..(((.((((((((......)))))))))))..))....)))))))))	..............(((((((....)).((((((((......))))))))).............))))).	......((((((((....)).))))))....(((((......)))))(((((...........))))))..	(((((.((((((.((....(((((..((......)))))))).))))))))))))))...............	(((((.((((((((....)).))))))..........(((.....)))...)))))...............	(((.(((.....))).((.......)).((((((((......))))))))..)))..............
2NBX	/	/	/	/	/	/	/	/	/	/		((((((...(((((((((((((((.(((........))))))).)))))((((((.((((((((.....)))))))).....)))))))........))))).))))))	((((((.....((.((((((((((((.(((.........)))))))..))).)))).))...((((((((.....)))))))......((.(((....))).))..))))))	((((((...(((((((((((((((((.......)))).....))..))))))((((((((.(((((((.....))))))))....)))))))........))))).))))))	((((((...((((.((((...((((.....)))...)))))))........((((((((.(((((((.....)))))))....))))))))........ .....))))))	((((((...(((((((((((((((.(((.........))))))))..))))))((((((.........(((..((....))..)))))))))).......))))).))))))	.((((.........))))...(((((((((.........))))))))......(((((((....((((((((.....))))))))......((.(((....))).)).)))))))	((((((...((((.((((...(((.....)))...))))))))(((....((((((((.(((((((......)))))))....))))))))........)))..))))))	((((((.....(((.(((((((((((.(((.........))))))))..))))))..........(((((((.....))))))))...)))((.(((....))).)).)..))))))	(((((.....((((.((((...(((.....)))...))))))))...(((((((((((.(((((((.....)))))))))....)))))))..........)))))))))	((((((...((.((((.....)))).))..........((((.........((((((((.((((((((.....)))))))).....)))))))))..........)))).))))))
2NBY	/	/	/	/	/	/	/	/	/	/		((((((((((((.(((.........))))))..)))))))	((((((((...((.(((((...)))))...)).)))))))	(((((((((((((((......))))).....))..)))))))	(((((((.((....)).......((...))..)))))))	(((((((.((....)).((......)).....)))))))	(((((((.((....))...((.....))....)))))))	(((((((.((....))....((........)).)))))))	(((((((......((((((...)))))).))).....))))	(((((((.((....)).((............)))))))))	(((((((......(((((...)))).)))).)))....
2NBZ	/	/	/	/	/	/	/	/	/	/		((((((((.(((((((.....))))))))....))))))))	((((((((..(((((((.....))))).....)))))))))	((((((((((.....((((.......))))...)))))))))	(((((((.........(((..((....))..)))))))))))	((((((((.((....))...((....))....))))))))	((((((((.((....)).......((....)).))))))))	.((((....((((((((.....))))))..)))))......	((((((((..((....))...((.........)))))))))	((((((((.((....))..((..........)).))))))))	(((((((((((((....))...)).............)))))))
2NC0	/	/	/	/	/	/	/	/	/	/		(((((...(((((....))))).)))))	(((((..((.(((....))))).)))))	((.((...(((((....))))))).)).	((((((...((((....))))))).)))	(((((..(((.((....))))).)))))	.((((...(((((....))))).)).))	(((((((....((....))))..)))))	(((((......((((....)))))))))	(((((...)((((....))))...))))	(((((.........((.....))..))))
2NC1	/	/	/	/	/	/	/	/	/	/		((((((...(((((((((((....))))))(((((((....)))))))........))))).))))))	((((((.....((.(((((((.....))).)))).))........((.(((.....)))).))..))))))	((((((...((....))....((((....((((((((....)))))))).......)))).))))))	.((((.........)))...........((((((((....)...((.(((....))).)).)))))))	((((((...((((((((((((....))))))..........(((....))))......))))).))))))	((((((.....((.(((((((......))).)))).))...((((....(((....))))))).))))))	((((((...((((..........))))..((((((((....))))))))).............))))))	((((((.....(((.((((((....)))))).((...))..)))((.(((....))).))..))))))	.((((..........((((((....))))))((((((((....)))))))).........)))))......	((((.....((....)).........((((((((((((....)))))))..........)))))))))))
2NCI	/	/	/	/	/	/	/	/	/	/		((((((.....((((.....))))))))))	(((((.(...((((....))))))))))))	(((((.((...(((....))))))))))	(.((((.....((((.....))))))))).	((.(((.....((((.....)))))))))).	((((((......(((.....).))))))))	(((((((...)(((.....))).))))))	(((..(.....((((.....)))))..))))	(((((((.....)((.....))..))))))	((((((......)(((.....))...))))))
2O33	/	/	/	/	/	/	/	/	/	/		(((..(((....)))..)))	((....))..........	(((..(.(.....))..)))	(((..(.((...)))..)))	((...(((....)))))...	....((((....))..)..	/	/	/	/
2OJ7	/	/	/	/	/	/	/	/	/	/		((....))	/	/	/	/	/	/	/	/	/
2PCV	/	/	/	/	/	/	/	/	/	/		(((.((((...((((....)))).....)))))))	......(((((((..((.......)))))))))..	(((((.((....)).((.......)....)))))	(((((.((....)).......((......)))))))	(((((.((....)).....((.....))..)))))	(((((.......(((....))).((...)))))))	(((((.....((....))..((......)))))))	(((.((((((((....)).))......)))))))	((.....))..((((....))))...........	(((((.......((.((.......))..)))))))
2PJP	/	/	/	/	/	/	/	/	/	/		((((((.(((....)))))))))	(((((.((((....)))))))))	(((((((.........).))))))	((((((((......)).))))))	...((((((....))))..)))	.((((.........)))).......	((....((((....))))))...	((((((.........)..))))))	((((((.........))..))))	(((((((...)......))))))
2PXB	/	/	/	/	/	/	/	/	/	/		((..((((((....(((....(((....)))....))).))))))..))	((..(((....((.(((....(((....)))....)))..)))))..))	((..((((((.....((....(((....))).)).....))))))..))	((..((((((.((......))(((....)))........))))))..))	((..((((((....(((...((((....))..)).))).))))))).))	((((.....)))).(((....(((....)))....)))..(((...)))	((..((((((.........((((....)).....))).)))))).))	((..((((((....((......))....((.......)).)))))))..))	((..(.................(((....)))....((....)).)..))	((((((....(((......)))((....)).)))).))..(((...)))
2PXD	/	/	/	/	/	/	/	/	/	/		(((((((((((....(((....(((....)))....))).)))))))))))	(((((((.....((.(((....(((....)))....)))..)))))))))	((((((((((.....((....(((....))).)).....))))))))))	((((((((((.((......))(((....)))........)))))))))	((((((((((....(((...((((....))..)).))).))))))))))))	..(((((((.(((......)))((....)).)))))))..........	..((((((((.((...((......))..)))))))))............	..((((((((.((.((......))....)))))))))))...........	..(((((((.(((......)))((....)).)))))))..((....))	((((((((((..........(((((....)).....))).))))))))))

	DNA model											RNA model									
PDB	mfold MFE	subopt #2	subopt #3	subopt #4	subopt #5	subopt #6	subopt #7	subopt #8	subopt #9	subopt #10		mfold MFE	subopt #2	subopt #3	subopt #4	subopt #5	subopt #6	subopt #7	subopt #8	subopt #9	subopt #10
2PXE	/	/	/	/	/	/	/	/	/	/		((..((((((....(((....(((....)))....))).))))))..))	((..(((....((.(((....(((....)))....)))..)))))..))	((..((((((.....((....(((....))).)).....))))))..))	((..((((((.((......))(((....)))........))))))..))	((..((((((....(((...((((....))..)).))).))))))..))	.(..(((.....)))..)...(((....)))....((....))......	((..((((((.........(((((....)).....))).))))))..))	((..((((((....((......))....((......)).))))))..))	.(..(((.....)))..)...(((....)))....(((......)))...	((..(((..........)))..))....(((....((....))...)))
2PXF	/	/	/	/	/	/	/	/	/	/		((((((((((....(((....(((....)))....))).))))))))))	(((((((....((.(((....(((....)))....)))..)))))))))	((((((((((.....((....(((....))).)).....))))))))))	((((((((((.((......))(((....)))........))))))))))	((((((((((....(((...((((....))..)).))).))))))))))	((((((((((....((......))....((......)).))))))))))	((((((((((.........(((((....)).....))).))))))))))	((((((((((((...))....(((....)))........))))))))))	((..(...)..)).(((....(((....)))....)))...........	((.(((....))).(((....(((....)))....))).......))
2PXK	/	/	/	/	/	/	/	/	/	/		((..((((((....(((....(((....)))....))).))))))..))	((..(((....((.(((....(((....)))....)))..)))))..))	((..((((((.....((....(((....))).)).....))))))..))	((..((((((.((......))(((....)))........))))))..))	((..((((((....(((...((((....))..)).))).))))))..))	.((((....)))).(((....(((....)))....)))..........	.((((....)))).(((....(((....)))....)))..((....)).	.((((....))))........(((....)))....((....))..........	((.(((....(((......)))((....))..))).))..............	((..((((((....((......))....((......)).))))))..))
2PXL	/	/	/	/	/	/	/	/	/	/		..((((((((....(((....(((....)))....))).))))))))	((.(((....))).(((....(((....)))....)))...))....	..(((((....((.(((....(((....)))....)))..)))))))	((.(((....)))........(((....))).)).((....))....	..((.((((.(((......)))((....)).)))).)).........	..((((((((.....((....(((....))).)).....))))))))	..((.(((((.((...((......))..))))))).)).........	..((((((((....(((...((((....))..)).))).))))))))	..((.(((((.((.((......))....))))))).)).........	((.(((....)))......(((((....)).....)))...))....
2PXP	/	/	/	/	/	/	/	/	/	/		((((((((((....(((....(((....)))....))).))))))))))	(((((((....((.(((....(((....)))....)))..)))))))))	((((((((((.....((....(((....))).)).....))))))))))	((((((((((.((......))(((....)))........))))))))))	((((((((((....(((...((((....))..)).))).))))))))))	((((((((((....((......))....((......)).))))))))))	((((((((((.........(((((....)).....))).))))))))))	..(((((((.(((......)))((....)).)))))))............	(((((...............(((....)))....((....)).)))))	((((((((((((...))....(((....)))........)))))))))
2PXQ	/	/	/	/	/	/	/	/	/	/		((((((((((....(((....(((....)))....))).))))))))))	(((((((....((.(((....(((....)))....)))..)))))))))	((((((((((.....((....(((....))).)).....))))))))))	((((((((((.((......))(((....)))........))))))))))	((((((((((....(((...((((....))..)).))).))))))))))	.((((((.....))))))...(((....)))....((....))......	((((((((((.........(((((....)).....))).))))))))))	((((((((((....((......))....((......)).))))))))))	.((((((.....))))))...(((....)))....(((......)))...	(((((((..........))))))....(((....((....))...)))
2PXT	/	/	/	/	/	/	/	/	/	/		((((((((((....(((....(((....)))....))).))))))))))	(((((((....((.(((....(((....)))....)))..)))))))))	((((((((((.....((....(((....))).)).....))))))))))	((((((((((.((......))(((....)))........))))))))))	((((((((((....(((...((((....))..)).))).))))))))))	((((((........(((....(((....)))....)))..))))))....	...((((((..((.(((....(((....)))....)))..)).)))))))	((((((((((.........(((((....)).....))).))))))))))	((((((((((....((......))....((......)).))))))))))	(((((...............(((....)))....((....)).)))))
2PXU	/	/	/	/	/	/	/	/	/	/		((((((((((....(((....(((....)))....))).))))))))))	(((((((....((.(((....(((....)))....)))..)))))))))	((((((((((.....((....(((....))).)).....))))))))))	((((((((((.((......))(((....)))........))))))))))	((((((((((....(((...((((....))..)).))).))))))))))	((((((((((....((......))....((......)).))))))))))	((((((((((.........(((((....)).....))).))))))))))	(((((...............(((....)))....((....)).)))))	((((((((((((...))....(((....)))........)))))))))	(((((.(((.(((......)))((....)).))).((....)).)))))
2PXV	/	/	/	/	/	/	/	/	/	/		((..((((((....(((....(((....)))....))).))))))..))	((..(((....((.(((....(((....)))....)))..)))))..))	((..((((((.....((....(((....))).)).....))))))..))	((..((((((.((......))(((....)))........))))))..))	((..((((((....(((...((((....))..)).))).))))))..))	((((.....)))).((.(((.(((....)))....((....)).)))))	((((.....)))).(((....(((....)))....)))..((....)))	..((((....))))((.(((.(((....)))....((....)).)))))	((..((((((.........(((((....)).....))).))))))..))	((..((((((....((......))....((......)).))))))..))
2QH2	/	/	/	/	/	/	/	/	/	/		((((((((........))))))))	(((((((((.....).))))))))	((((((((.(.....)))))))))	((((((((((...)).))))))))	(.((((((........))))))).	((((((.((.......))))))))	((.(((((........))))))).	(((((......((....))))))))	((((((((....))......)))))	(((((((.......)...))))))
2QH3	/	/	/	/	/	/	/	/	/	/		((((((((.......))))))))	((((((((..(...)))))))))	((((((.((......))))))))	(.((((((.......))))))).	((.(((((.......))))))).	((((((((..(.....))))))))	(((((((......)...))))))	((((((..((.....))))))))	(((((((......))...)))))	(..(((((.......)))))..
2QH4	/	/	/	/	/	/	/	/	/	/		((((((......))))))	/	/	/	/	/	/	/	/	/
2R8S	/	/	/	/	/	/	/	/	/	/		.(((((((......(((..((....(((.(((((((((.((((((((((....))))))))).(((((....))))).)))).....))))))))...))..)))............((((...((((((((.....))))))))..))))))))))).	((..(((((.....(((..((....((((.((((((((.((((((((((....))))))))).(((((....))))).))))......))))))))...))..))).....)))))))..((((...((((((((.....))))))))..)))))........	.(((((((.(((.....(((..((((.......(((((.((((((((((....))))))))).(((((....))))).)))))....))))...)))....)))..............((((...((((((((.....))))))))..))))))))))).	.(((((((........(((...((((.......(((((.((((((((((....))))))))).(((((....))))).)))))....))))..((((...))))..........)))..((((...((((((((.....))))))))..))))))))))).	.((((((((............((((.......(((((.(((((((((....)))))))))).(((((....))))).)))))....))))..((((.((((..........)))).))))...((((((((.....))))))))......)))))))).	.((((((((........(((((((((....((..(((((.(((((((((....))))))))).(((((....))))).)))))......((((((((.((((...........)))).))))...))))...))....)))))))).....)))))))).	.(((((((.((.....(((.....)))......(((((.(((((((((....)))))))))).(((((....))))).)))))....((((..((((...))))...)).))..))..((((...((((((((.....))))))))..))))))))))).	.(((((((.((......((.....(((((((((((((.(((((((((....))))))))).(((((....))))).))))...)))))......)))))....)).......))..((((...((((((((.....))))))))..))))))))))).	.(((((((.........(((..((((.......(((((.(((((((((....))))))))).(((((....))))).)))))....))))...)))...((((....((........))......((((((((.....))))))))..)))))))))))).	.(((((((.......(((((..((((.......(((((.(((((((((....)))))))))).(((((....))))).)))))....))))..((((.((((...........)))).))))...........)))))(((((....))))).))))))).
2RLU	/	/	/	/	/	/	/	/	/	/		(((((((.....)))))))	((((((((...))))))))	.......(((......)))	/	/	/	/	/	/	/
2RO2	/	/	/	/	/	/	/	/	/	/		(((((((((.....)))))))))	((((((((((...))))))))))	((((((((......)).))))))	((.((((((.....)))))))).	(((((.(((.....)))))))).	.((((((((.....)))))).))	((((((.((......))))))))	((....))......(((...)))	((((.((((.....))).)))))	(((.(((((.....))).)))))
2RPK	/	/	/	/	/	/	/	/	/	/		(((((((......)))))))	((.((((......)))))).	(((.(((......)))))).	.((((((......)))).))	((((((.......).)))))	((((((.......)).))))	((((((...)).....))))	(..((((......))))..	((..(((......)))))..	/
2RPT	/	/	/	/	/	/	/	/	/	/		((((.(((....))).))))	((.(((((....)).)))))	((((........))))....	.....((((......)))).	((.((.......)))))....	((....))....((....))	/	/	/	/
2RVO	/	/	/	/	/	/	/	/	/	/		((((((((...(((((....))))).))))))))	((((((((.........(((...)))))))))))	((((((((((.....))..........))))))))	((((((((((..........))....))))))))	((((((((((..........))......)))))))	((((((((......((.......)).))))))))	((((((((((...))).............))))))	/	/	/
2TPK	/	/	/	/	/	/	/	/	/	/		........((((((((((.....))).)))))))..	((((((....((((....))))....))))..))..	..(((((........))))................	..(((((........)))))......((....))..	((((((((........))))..((...))...)))..	((((....((.(((.........))).))..))))..	((.((((........))))....((....)).))..	(((((((........))))).......))........	((.((((........)))).((........))))..	((((((..(((....)))........))))..))..
2UWM	/	/	/	/	/	/	/	/	/	/		(((((((((.....)))))))))	((((((((((...))))))))))	((((((....(.....)))))))	((((((....((...))))))))	...((((((.....))))..)).	(((...)))......(((...)))	(((...))).....(....)...	(((...)))....((....))..	((((((((......)...))))))	(((...)))(((..(....))))
2V3C	/	/	/	/	/	/	/	/	/	/		((((..(((((.((((((((((....))))))))))..)))))....((((((.....(((.....(((....)))......)))..))))).)))).	((((..(((((.((((((((((....))))))))))..)))))....((((((..(((..((....(((....))).))...))).)))))).)))).	((((..(((((.((((((((((....))))))))))..))))).((.(((((((....((....))(((....))).....))))))).))..)))).	((((..(((((.((((((((((....))))))))))..)))))....((((((...(((..((......))..))).((....)).)))))).)))).	((((((((((.((((((((((....)))))))))..)))))...))).)))....((.(((...((((...))).((....)).))).))......	(((((((((((.((((((((((....))))))))))..)))))...))).)))......(((.....(((....)))......)))..((((.....))).	((((..(((((.((((((((((....))))))))))..)))))....((((((.....((....)).(((...........)))..)))))).)))).	(((((((((((.((((((((((....))))))))))..)))))...))).)))......((((...(((....))).((.........)).)).))...	((((..(((((.(((((((((((....))))))))))..))))).((((((((......((....))(((....))).)))..))))))......)))).	((((..(((((.((((((((((....))))))))))..)))))....((((.((.....)).))))(((....))).((........))....)))).
2VPL	/	/	/	/	/	/	/	/	/	/		(((((((((..((((((((....))))).)))......))))))))).	((((((((....((((((((....)))).)))).((...))))))))).	(((((((((....((((((....)))......)))...))))))))).	(((((((((....(((((............)).)))...))))))))).	(((((......(((((((((....))))).)))))......)))))....	((((((((((....(((((....))))))..)).......))))))))).	(((((((((....((((....((.....)).))))...))))))))))).	(((((((((....((((.......((...))))))...))))))))))).	((((((((((.....).....((((......))))...)))))))))).	(((((((((((.........))).((....)).....)))))))))).
2W2H	/	/	/	/	/	/	/	/	/	/		((((((((......))))))))	(((((((((....)))))))))	((((((.(.....)))))))	((((((.((...))))))))	((.........))..........	((((((...))).))))......	/	/	/	/
2XXA	/	/	/	/	/	/	/	/	/	/		((((((((((...((.(((..((((.((((((............(((....))).(((.((.....)).)))....)))))))))))))).)))))))))))	((((((((((.((((((((.(((((((((((.....((((....(((....)))....)))...))).)).))))))..))).)))))...)))))))))))	((((((((((.((((((((.((((((((((....(((((....)))))..(((....)))....))).)))))))).)))).)))))...))))))))))	((((((((((...((.(((..((((.((((((....((((....(((....)))....)))).(((....))).))))))))))))))).)))))))))))	((((((((((...((.(((..((((.((((((..((((((....(((....)))....)))...)))........)))))))))))))).)))))))))))	((((((((((...((.(((..((((.((((((...((((((....)))))(((((((....((....))..))))))).))))))))))))))))).))))))))))	((((((((((...((.(((..((((.((((((.....(((....(((....)))......((....))...)))...))))))))))))))).))))))))))	((((((((((.((((((..((.(((....))).(((((......(((....))).(((.((.....)).)))....))))))))))))...))))))))))	((((((((((...((.(((..((((.((((((.........(((.(((....))).(((.((.....)).))).)))))))))))))))))).))))))))))	(((((((((.(((((.(((..(((....)))...)))......(((....))).(((.((.(((((....))))).)).))).))))))...))))))))))
2Y8W	/	/	/	/	/	/	/	/	/	/		(((((((....)))))))..	((((((....)).))))....	(((((..(....))))))..	..(((.((....)))))...	(((.((....)).)))....	...((.((....)).))...	/	/	/	/
2Y8Y	/	/	/	/	/	/	/	/	/	/		(((((((....))))))).	((((((....)).))))..	(((((..(....))))))).	..(((.((....)))))..	(((.((....)).)))...	...((.((....)).))..	/	/	/	/
2Y95	/	/	/	/	/	/	/	/	/	/		(((((....)))))	.((......))...	...((......)).	/	/	/	/	/	/	/
2Y9H	/	/	/	/	/	/	/	/	/	/		(((((((....)))))))	((((((....)).)))).	..(((.((....))))).	(((.((....)).)))..	...((.((....)).)).	/	/	/	/	/
2ZM5	/	/	/	/	/	/	/	/	/	/		(((((((..((((........)))).(((((((...))))))).....((((((......).)))))))))))..	((((((...(((((((((.((..(((((((((...))))))).))..))..)))))))..))))))))))..	((((((((.((((........))))..(((((((((.....(((.....))))))))))))..))))))))).	((((((((.((((........)))).((((((((...)))))))............(((....)))))))))))..	((((((((.......((((..((((..((((((((........)).)))))..))))....)))))))))))))..	((((((((.((((........)))).((((((((...)))))))..(((........)))......)))))))).	((((((((((((((.........)))).((((((((...))))))))).)))))..(..(((....)))..).))))..	(((((((..(((.....)))...(((((((((((...))))))).))).(((((.......)))))))))))))..	(((((((((((((........))))).((((((((...)))))).))))).......(((.....))).))))..	..........((((........))).((((((((...)))))))((((((..(..(((....)))..).)))))))

	DNA model										RNA model									
PDB	mfold MFE	subopt #2	subopt #3	subopt #4	subopt #5	subopt #6	subopt #7	subopt #8	subopt #9	subopt #10	mfold MFE	subopt #2	subopt #3	subopt #4	subopt #5	subopt #6	subopt #7	subopt #8	subopt #9	subopt #10
2ZNI	/	/	/	/	/	/	/	/	/	/	(((((((.((((.....)))).((((((.......))))))...(((((.......)))))))))))))....	(((((((.((((.....))))..((((.......((((((....)))))).......))))))))))))....	((((.(((((((.....)))).)))............((((.((.(((.....)))))))).))))....	(((((((....((((((((((.....)).)))).))))....((((((.......))))))))))))))....	(((..(((((((.....)))).)))...)))...........((((((.((.((....))..)))))))).	((((((((((....((((((.....)).)))).))))......(((((.......)))))))))))))....	....(((((............((((((.......))))))...(((((.......)))))....)))))).	(((((((.((((.....)))).......((.((......)).))(((((.......)))))))))))....	((((((((((.....((((((.....)).)))).((((((....))))))......)))...)))))))....	((((((((((((.........((((((.......))))))........)))...)))...))))))....
2ZUE	/	/	/	/	/	/	/	/	/	/	((((((((..........((((...((((((..........)))))..))))((((......)))).))))))))).	((((((((.(((..(((.......))).(((((.......))))).)))..((((......)))).)))))))).	((((((((..(((....)))...((((.(((((.......)))))..))))((((......)))).))))))))).	((((((((.....((((.((((...(((((..........)))))..))))..))))..........)))))))).	((((((((.((((..........))))).(((((.......))))).....((((......)))).)))))))).	((((((((((((...((.((((...(((((..........)))))..))))))))))..........))))))).	((((((((.((((.((..((..((((.........))))...))))))))..((((.......)))).))))))))).	((((((((.((((.((.((((...((((....))))))).....))))))..((((......)).)))))))))).	((((((((.((...))..((((.((((.(((((.......)))))..))))...))))........)))))))).	((((((((.(((..(((.......))))))(((((..........))))).((((......)))).))))))))).
2ZZM	/	/	/	/	/	/	/	/	/	/	((((((((.(((..((((......))))....)))...((((((...((((....)))).....))))))........))))))))	....((((((((..((((......))))....)))....)))))......(((((((...(((((.......))))))))))))	((((((((......(((..((......))..)))...((((((...((((....)))......))))))........))))))))	((((((((......((((......))))..(((((((.((((((..........)))).)).))))))).......))))))))	((((((((......((((......))))...(((....((((((...(((....))).....))))))....))).))))))))	((((((((.(((..((((......))))....)))...((((((...(((....))).....))))))..)))))))))......	((((((((.((((....))))...((((.....)))).((((((...(((....))).....))))))........))))))))	((((((((.((((....))))....((.((((((.......)))))).))..(((((((..........)))))))))))))))	(((((((...(((.((((......))))..))).((((((((...))))..))))......(((((.......)))))))))))))	((((((((....((.(((((((...((.((((((.......)))))).))....))...))))).))...))))))))......
2ZZN	/	/	/	/	/	/	/	/	/	/	(((((((..((((((...)))))).(((((.......)))))......(((((.......)))))))))))	(((((((.......(((((...((((....))))......)))))..(((((.......)))))))))))))	((((((((((((.....)))))....(((((.......)))))......(((((.......))))))))))))	(((((((..((((((...))))))...(((......(((((((.....)))))))....)))...)))))))	(((((((..((...((((....((((....)))).(((((((.....)))))))....))))))))))))))	(((((((.((((....))))(((..(((((.......))))))))..(((((.......)))))))))))))	(((((((((((((....(((....)))))))))))).............(((((.......))))).))))))	(((((((..((((((...))))))).(((....((......))....)))(((.......)))..))))))))	(((((((((((((....)))))).((((....)))).(((..((((.....))))....)))....)))))))	(((((((..((((((...))))))...((((.......((((.....)))))))).........)))))))))
3A2K	/	/	/	/	/	/	/	/	/	/	((((((...(((....((((((((((.....)))).))))))...))).(((((.......))))).))))))....	((((((((((((((....(((((((((.....)))).)))))....((((....)))).))).))).))).))))))))....	(((((((...((((.........))).(((((.......)))))......(((((.......))))).)))))))....	(((((((.......((.((...((((..((((((..........))))))..))))....)).)))))))))....	((((((((((.......((((((((((.....)))).)))))....(((((......)).)))))).)))))))....	((((((((((((((.....))))))).((.(((((.......)))))..)).(((((.......))))).)))))))....	((((((...(((....((((((((((.....)))).))))))....)))...)))))).........((....)).	((((((((..((((..........)))))....(((((..((((...........)))).)))))....))))))))....	((((((...(((..(((.......))).(((((.......))))).))).(((((.......)))))).))))))....	((..((((((((.....))))))))..(((((.......))))).((((((((.......)))))..))))..)).
3A3A	/	/	/	/	/	/	/	/	/	/	(((((((....((((((....))))))((((((.......)))))).(((((....))))).((((.......))))..)))))))	(((((((....((((((....)))))))....((.....((((.....((((((....)))))...))))......))....))))))))	(((((((....((((((....)))))).(((((.......))))).(((....)))...((.((((.......)))))))))))))	((((((((((..((((((....))))))..))...........((((.(((....))).)))).((((.......))))..)))))))	(((((((.........((.(.(((((.((((((.......))))).)...))))).))...((((.......))))..)))))))	(((((((.(((((((((....))))))((((((.......)))))).)))........((.((((.......)))))))))))))	(((((((...........(((.((((((....)))))).))).....(((((....))))).((((.......))))..)))))))	(((((((.((((......)))).....((((((.......)))))).(((((....))))).((((.......))))..))))))))	((((((((((.((((((....))))))((((((.......)))))).(((((....))))).......)))..........)))))))	.(((((((.((.....)).)))))).((((((.......)))))).(((((....))))).((((.......)))).........
3ADB	/	/	/	/	/	/	/	/	/	/	(((((((((..((((((....))))))((((((.......))))))((((((((....))))))))((((......)))).)))))))))....	(((.((((((..(((((((..((((((.((((((.......)))))).((((((....))))))))))))..))...))))).)))))).))).	(((((((((..((((((....))))))...(((.....(((((..((((((((....))))))))))))))....)))..))))))))))....	(((((((.(((((((((((..(....)((((((.......))))))).))))))))).))))...((((((.......)))))))))))))))....	(((((((((..((((((....))))))...(((((((.........((((((((....))))))))))))))........))))))))))....	(((((((.(((((((((((....(((((............)))))..)))))))).))))...((((((.......))))))))))))))....	(((((((((..(((((..(((((.......)))))......))))).(((((((....)))))))((((......)))).)))))))))....	((((((((((((....))))..(((((.((((((.......)))))).((((((....))))))))))))...........)))))))))))....	(((((((((..((((((....)))))))((((((.......))))))..((((((.(.....).))))))..........)))))))))....	(((((((((..((((((....))))))((((((.......)))..(((((((....)))))))).)).........)))))))))....
3AKZ	/	/	/	/	/	/	/	/	/	/	(((((((.((((((.......)))))..((((((..(.((((....))))..)..))))))...)))))))....	(((((((.........((.(((((..((((((.....((((....))))))))))).))))))))))))))))....	(((((((................(((((((.......)))))))..(((((.......)))))))))))))....	((((......)))).((((.((..((((((.......)))))))((.(((((.......))))))))..)).))).	..((((((((((((.......))))))))...))))......(((((.(((((.......)))))....))))))..	(((((((..((((..((......))..)))).(..(.....)..).((((((.......))))))))))))....	.((((((((((((.......)))))).(((((..(.((((....))))..)..))))))))))..........	(((((((........((((.((((((......)))..))))))..(((((.......)))))))))))))....	(((((((..((((..((.......))..))))...(((..((((......))..))..)))...))))))))....	((((.((((((((..((......))..))))....))))....((.(((((.......))))))).)))))....
3AMT	/	/	/	/	/	/	/	/	/	/	(((((((................((.(((((((.......))))))).))(((((.......))))))))))))))....	(((((((..((((....))))..((.((((((((.......))))))).))(((((.......))))))))))))))....	(((((((.......(((.(((..((((.....))))...))).)))....(((((.......)))))))))))))....	(((((((.((...((((......((.(((((((.......)))))))).))...))))........)).)))))))....	(((((((..(((..(((.(((..((((.....))))...))).))))).(((((.......))))))))))))....	(((((((..((((..........))))((((((.......))))))....(((((.......)))))))))))))....	(((((((..((((((((.(((......))).))))........))))....(((((.......))))))))))))....	(((((((.((...............(((((((.......)))))))).(((......)))....)).)))))))....	.((((.((......))..))))).((.(((((((.......)))))).))..(((((((........)))))))....	.(((..........))).((...((.((((((((.......))))))))).))..(((((((.........))))))).)).
3EGZ	/	/	/	/	/	/	/	/	/	/	(((((..((((((...........))))))((((((((..........)))))))........)))))	....(((.((((..............(((((((((..........))))))))......)))))))	(((((..((((((...........)))))..(((....))).....................)))))	(((((...((((.........(((.....)))........))))...((.......))...)))))	(((((...(((.........)))(((..(((.........)))..)))............)))))	(((((...(((..........(((.....))))))..(((......)))............)))))	(((((..(((((..((.....(((.....)))...........))...))))).......)))))	(((((...((((...(((...(((.....)))...))).))))...((........))...)))))	(((((..(((((..((...(((..((....))....)))...))...)))))........)))))	(((((..(((((..........)))))........(((.((....))...)))......)))))
3EPJ	/	/	/	/	/	/	/	/	/	/	(((((((.((((.......)))).(((((((...)))))))).((((.........))))....)))))))	((((((..((((.......)))).((((((((...))))))).....((((((.......)))))))))))	(((((((((((....((..((.(((((((((...))))))))))))))).......))..))...)))))))	(((((((.((((.......))))..((((((((.....(((.........)))..)))))).)).)))))))	(((((...((.(((.(((.((.(((((((((...))))))))))))............)))))).)))))))	(((((((.........((..((.(((((((((...)))))))))))))))).....(((....)))))))))))	(((((((.((((.......))))..(((...(((....))).((((........)))))))).)))))))	((((((.(((.(((..(....((((....))))....)..))))))(((((.......))))))))))))	(((((((.((((.......))))..(((((((...(((....))).........))))).)).)))))))	(((((((......(((....))).((((((((...))))))).((((.........))))....)))))))
3FOZ	/	/	/	/	/	/	/	/	/	/	(((((((..((((.........)))).(((((((...)))))))......(((((.......)))))))))))..	((((((...((((((((((.((..((((((((((...))))))).))..))...))))))..))))))))))))..	((((((((.((((.........))))..((((((((((.....(((......))))))))))))..))))))))))..	((((((((((((((........)))).(((((((((...))))))).)))))..(..(((....)))..).)))))..	(((((((.......((((..((((..(((((((((.........)).)))))..))))....)))))))))))))..	(((((((.((((.........))).(((((((...)))))))..(((........)))......)))))))).	(((((((..(((.....)))...((((((((((...))))))).))).(((((.......)))))))))))))..	.........((((........)))).((((((...)))))))(((((..(..(((....)))..).))))))	(((((((((((((........)))))).(((((((...))))))).)))))........(((.....))).))))..	.........(((((((((..((((..(((((..........))))))..))))....))))..)))))))..
3ID5	/	/	/	/	/	/	/	/	/	/	((((...))))..((((....))))..........	.((((((.......)))))).............	(((...)))((..((((....))))..))....	..(((((............)))))........	....(((......((((....)))))))).....	(((...)))((..((((....))))).....)).	.((((((.......)))))).((......))..	...((((.......))))((((...))))....	....((...((..((((....))))..))))..	((((...))))....(((.(((...))))))..
3IVK	/	/	/	/	/	/	/	/	/	/	((((((((..........))))))))...(((..........((((...)))).(((((((.(((......)))....(((((....)))))...(((((....)))))..))))))).......)))	(((....))).((((((..........................((((...)))).(((((((.(((......)))....((((((....)))))...(((((....))))))..)))))))...)))))).	((((((((..........))))))))...(((..........((((...)))).(((((((.(((.....))).......(((...(((((.....)))))...)))....))))))))...)))...)))	((((((((..........))))))))........((....((.((((...))))..)).....))....(((......(((((....)))))((.(((((....))))).))............))).	(((....))).((((((......((((...........))))((((...)))).(((((((.(((.....)))....(((((....)))))...(((((....)))))..)))))))...)))))).	((((((((..........))))))))...((((..........((((...))))))))...(((((.....)))))..(((((....)))))((.(((((....))))).))................	((((((((..........))))))))................((((...))))((((.....))))..(((......(((((....)))))((.(((((....))))).))............))).	....((((.....))))..((((((......................((((...)))).(((((((.((((.....)))....(((((....)))))...(((((....)))))..)))))))....))))).	((((((((..........))))))))...(((....((..(((((((...))))........(((((.....))))).)))..))........((.(((((....)))))).)).............)))	((((((((..........))))))))....................((.((((.......(((((.....))))).(((((....)))))...(((((....)))))..))))))).........
3K0J	/	/	/	/	/	/	/	/	/	/	(((((((((((((........))))))))......................((((..((((......))))..))))..))))))	(((((((((((((........))))))))...((((.....((.(((.(((....))).))).))....))))......)))))))	(((((((((((((........))))))))..............(((..(((....)))((((......))))......))).))))))	((((((..(((((.(((((..........)))))........)))))......((((..((((......))))..))))..))))))	((((((((((((((........))))))))........((.((....)).)).((((..((((......)))))..))))..))))))	((((((..(((((..(((....((.((....)))).)))...))))).......((((..((((......))))..))))..))))))	((((((((((((((........))))))))..............(((......((......))...(((....))).))).))))))	((((((((((((((........))))))))...((((............((((..((......))...))))))))........))))))	((((((((((((((........))))..(((...))).......))))......((((..((((.......))))..))))..))))))	((((((...((((((((((...........)))))............))))).((((..((((......))))..))))..))))))
3KTW	/	/	/	/	/	/	/	/	/	/	.(((((.((((((((((((((((.....))))))))))).)))))....(((((.....(((.....(((....)))......)))..)))))))))	.((((((((.((((((((((((((.....))))))))))))((((((((.((((.....))))......(((....)))...))))).)))..))))))))	.((((((((((((((((((((((((....))))))))))).))))).(((.((((.....)))).))).(((....))).(((....)))..))))))))	.(((((.((((((((((((((((((....))))))))))).)))))).....(((((....((((......))))..((......))...))))))))))))	.(((((.(((((((((((((((((....))))))))))).))))))).....(((((((..((.....))(((....))).....))...)))))))))))	.((((((((((((((((((((((((....))))))))))).)))))).....(((((....((.....))))))..(((.((....)).))).)))))))	.......(((((((((((((((((....))))))))))).))))))..(.((((.....)))).).((((.((..((......))...)).)))))...	.((((((((((((((((((((((((....))))))))))).))))).((.((((.....))))).))(((.(((..........))).))).))))))))	.(((((.(((((((((((((((((....))))))))))).)))))).((.((((.....))))).))..(((....)))......(((....))))))))	.(((((((..((((((((((((((....))))))))))).......((((.((((.....)))).....(((....)))..))))...))))))))))
3NDB	/	/	/	/	/	/	/	/	/	/	((((((((((((((((.(((((..(((((.((((((((((....))))))))))..))))))....((((((......(((......(((....)))......)))..))))))).))))))).)))))))))...)))))))	(((((((((((((((((.((((((..(((((.(((((((((....)))))))))..)))))....((((((...(((..((......))..))).((....)).))))))).))))))).))))))))...))))))))	((((((((((((((.....((..(.(((((.((((((((((....))))))))))..))))).)..)).(((((..((.(((...(((....))).((....)).))).))...))))).))))))))...)))))))	(((..((((((((((.(((((((((((((((.(((((((((....)))))))))))..))))))...))).)))).)))).......(((....))).....)))..)))..))).((.((((((....)))))).))..	((((((((((((((((.((((((((((((((.(((((((((....)))))))))..)))))...))).)))))).)))((((...(((....))).((........))........)))..))))))...)))))))	((((((((((((((((.(((((((((((((.((((((((((....)))))))))..)))))...))).))))).))))))....(((....))).....((..((((.(.....).)).)))..)).))).)))))))	((((((..((((((((.(((((((((((((.(((((((((....)))))))))..))))))...))).)))))).))))))....((((...(((.((.(((..........)))..)).))).))))))..))))))	(((((((((((((((((.(((((..(((((....))))))...(((((.........)))))...((((((.....(((.....(((....)))......)))..))))))).))))))).))))))))...)))))))	(((((((((((((((.(((((((((((((.(((((((((....))))))))))..)))))...))).)))))).))))))..(((((((......((........))......)))).)))......))).)))))))	((((((((((((((((.(((((..(((((.((((((((....)))))))))..))))).....(((.(((..((......))..))).)))..((........))....))))))).))))))))...)))))))

	DNA model									
PDB	mfold MFE	subopt #2	subopt #3	subopt #4	subopt #5	subopt #6	subopt #7	subopt #8	subopt #9	subopt #10
3NVK	/	/	/	/	/	/	/	/	/	/
3PHP	/	/	/	/	/	/	/	/	/	/
3SN2	/	/	/	/	/	/	/	/	/	/
3TUP	/	/	/	/	/	/	/	/	/	/
3U4M	/	/	/	/	/	/	/	/	/	/
3W1K	/	/	/	/	/	/	/	/	/	/
3W3S	/	/	/	/	/	/	/	/	/	/
3WC1	/	/	/	/	/	/	/	/	/	/
3WC2	/	/	/	/	/	/	/	/	/	/
3WFQ	/	/	/	/	/	/	/	/	/	/
3WFS	/	/	/	/	/	/	/	/	/	/
3WQY	/	/	/	/	/	/	/	/	/	/
3WQZ	/	/	/	/	/	/	/	/	/	/
484D	/	/	/	/	/	/	/	/	/	/
4A4S	/	/	/	/	/	/	/	/	/	/
4AL5	/	/	/	/	/	/	/	/	/	/
4AL7	/	/	/	/	/	/	/	/	/	/
4BW0	/	/	/	/	/	/	/	/	/	/
4C4W	/	/	/	/	/	/	/	/	/	/
4C7O	/	/	/	/	/	/	/	/	/	/
4ILM	/	/	/	/	/	/	/	/	/	/
4KR7	/	/	/	/	/	/	/	/	/	/
4KR9	/	/	/	/	/	/	/	/	/	/
4L8H	/	/	/	/	/	/	/	/	/	/
4M4O	/	/	/	/	/	/	/	/	/	/
4M6D	/	/	/	/	/	/	/	/	/	/
4OOG	/	/	/	/	/	/	/	/	/	/

	RNA model									
PDB	mfold MFE	subopt #2	subopt #3	subopt #4	subopt #5	subopt #6	subopt #7	subopt #8	subopt #9	subopt #10
3NVK	...((((.((....)))).))...	.((.((.......)))).......	.((...(.((....))))).....	...((((.((....))))....))	.(((..........))).......	......(.((....))..).....	.....((.((....))..))....	.(((((.......))).)).... ..	.((.((........)).))... ..	.......(((....))..).....
3PHP	((((((((......)))))))).	((((((((...(...))))))))).	((((((..(....))))))))).	((((((...((...))))))))).	((((((..((....)))))))).	(((((((.....)...)))))).	((((((((...))...)))))) .	(((.((((......))))).)).	(((((.((......))).)))).	(((((.((......))))).)).
3SN2	((((((.(((((......))))))))))))	((((((.((((((...)).))))))))))	((((((.(((.((...)).))).))))))	((((((.(((...((...)))))))))))	(.....)(((((......))))).	((((((.((.((........))))))))))	((...))(((((......)))))	((((((.((........))... .))))))	.((....(((((......))))))))....	....((.(((((......))))). ..)).
3TUP	(((((((....(((((((.((.....)))))))))).....((((.(((((....))))).)))).))))))..	(((((((..((((.........)))).. .............((((.(((((....))))).)))).)))))))..	(((((((.(((..((((((....((..((.((((........))))))..))...))))))..)))))))))). .	(((((((.((((((((((.((.. ...))))))))).((((((..... ..))))))........))))))))))..	(((((((.(((..((((((.... ((((((((........)))))).)))........))))))..))))))))))..	(((((((..((((.........)))).(((((.......)))))......(((((.......)))))))))))). .	(((((((.(((..((((....((((.((((((............))).))))))))))..))))))))))..	(((((((..((((........))))..(((......((((((.......))))))......))) .)))))))..	(((((((.((((((((((.((.....)))))))).(((((..((....))....)))))..)))))))))..	(((((((..........((((((...(((((.......)))))..(((((....)))))))))))))))))..
3U4M	((((.((((...((((((....)))))). ..((((((((....))))))))....)))).((((......))))..))))	..........(((..(((((..(((.(((..((((((((....)))))))).. ...))).)))......)))))..))).	(((..((((...((((((....))))))...((((((((....))))))))....)))).((((...)))).))).....	..........(((..(((((..((((.....((((((((....))))))))..)))).........))))) ..))).	(((.((.(((........(.((.((((......((((((((....))))))))..)))).)).)......)))))))).....	...................((((((((.....((((((((....))))))))..))))...((((......)))). .))))	............((((((....))))))...((((((((....))))))))(((((....(((((..... .))))))))).	((((.(((((((.......... .)))......((((((((....))))))))....)))).((((......))))..))))	..........(((..(((((.. (((.(((..((.((.(((((. ...))))))).))))).))). .....))))..))).	(((.((.(((..((((((....))))))..(((..(((((((.... .....))))))..)))....)))))))).....
3W1K	(((((((...((((((....))))))((((((.......)))).))(((((((.....))))))).(((((........))))).))))))).	(((((((...((((((....)))))) ....((..((.(((((((..(((((((.....))))))).))))))).)).)).....))))))).	((((((((((((((((....))))))............))))))..(((((((.....))))))).(((((.. .....)))))....)))).	((((((((..((((((....)))))).)))..................(((((((.....))))))).(((((. ......)))))...))))).	.(((((....((((((....)))))).....)))))........((((((((((.....))))))).(((((........)))))......)))	((((.(((..((((((....)))))).)))....((.(((((((..(((((((.....))))))).))))))).)).))))...........	(((((((....((..((((.... ....))))...((.(((((((.. (((((((.....))))))).)))))))).))..))....))))))).	(((((((..((((....(((. ((.....)).)))......)))) ...(((((((.....))))))).(((((........))))).))))))).	(((((((......((((...(((...((((((........)))).))...)))......)))).. ..(((((........))))).))))))).	(((((((...((((((....))))))................(((((((((.....))))))))...... ..)))..........))))))).
3W3S	((((((((..(((.(((....)))))).. ((((..((.((.(((...((((.((((.. ..)))).))))...))))).)).))))..))))))))...	((((((((..(((.(((....)))))).(((((........)))))..((((.((((....)))).)))).(((((..... ..)))))))))))))...	((((((((..((.((((....))))((((((((........)))))..((((.((((....)))).)))))...)))........))..)))))))).. .	((((((((..((..((((...(((.(((((((((........))))).))).)))(((....))))))))).(((((.......)))))))))))) ...	((((((((((((.((((....)))).))...))))))...))))(((((((.((((....)))).... .((((((........))))))..))))))).	((((((((..((((((..((.... .))))))....((.((.(((...((((.((((....)))).))))...)))))).))...))..))))))))). ..	((((((((..((((..((((.((..((((((((........))))) .)))...((((....)))))).))))..))........))..))))))))..	((((((((..(((.(((....))))))...((((((....(((....((((.((((....)))).))))))))))))))...... ...))))))))...	((((((((...(((....))) ........((((..((.((.(((...((((.((((....)))) .))))...))))).)).)))) ..)))))))))...	((((((((..((...((.((..((.((((((((........))))).))).))..))))....((((((. ...))))))......))..))))))))...
3WC1	((((((......((((((((((.((.....)).)))))).))))...(((((.........))))).))))))..	((((((.((((....((((((((.((. ....)).)))))))))))...(((((.......))))).))))))..	((((((((........((((((.((.....)).)))))).((..(((.. ..)))..)).....))))))))..	((((((((..((((((....))))))((((((...(((..........)))....))))).....)))))))). .	((((((....((((((....))))))(((((........)))))... .(((((........))))).))))))..	((((((((.........(((((((.((.....)).)))))))...(((... ....)))..))))))))..	((((((....(((((((((((... .....)))))))))).......... .(((((........))))).))))))..	(((((((..((.(((((((((((.((.....)).)))))).)))).)).......(((...))) .)))))))..	..............((.(((((((..(((((...(((....... ...)))....)))))..))))))).))..	((((((....((((((....))))))..........((((((..(((....)))..)))...)))))))))..
3WC2	((((((...((((.........))))...... .((((((((((.(((.(......).))))))))))))).))))))..	((((((((......((((.((......)).))))(((((((.(((.(......).)))))))))).))))))))..	((((((((..(((....)))..)) .......((((((((.(((.(... ..).))))))))))))).)))))). .	((((((((.((((.........))))....(((...((((((....)))))...)))..........)))))))). .	((((((.........(((.....)))......(((((((((.(((.(. ....).))))))))))))).))))))..	((((((((.....(((((.((.... ..)).))))).((((((....))))).(((........))).)))))))) ..	((((((...((((.........)))).((((((.......)))))... ..((((((.......))))).))))))..	((((((((..(((....))). ((((((..((((.......... .)))))..))))))........)))))))..	((((((((((((...))))))).. ..........((((((((((.(((.(......).)))))))))))).))))))..	((((((((.((((.........)))).((((((.......)))))).((((........))))....))))))))..
3WFQ	(((((((..((((.........)))).(((((.......)))))......(((((.......)))))))))))).	(((((((..((((.........)))).. ....(((......)))......(((((.. .....))))))))))))).	(((((((....(((((..((.....))..).))))............(((((.......))))))))))))).	(((((((..(((.....)))...((((((((.......)))).)))). ((((((.......))))))))))))) .	(((((((......((.((((.... .((......)).....)))).)). .(((((.......))))))))))))).	(((((((..((((.........))))...(((.......))).((.(((((....))))).))...))))))).	(((((((.......(((.....))).(((((........)))))... ..(((((........))))))))))))).	((((((((((..((((.(... ...)))))..))).......... .....(((((.......)))))))))))).	(((((((..((((.........))))....(((.......... ..)))(((((.......)))))))))))).	(((((((..((((.........))))..((((((...((....)). ....)))))))........))))))).
3WFS	(((((((..((((.........)))).(((((.......)))))......(((((.......))))))))))))..	(((((((..((((.........)))).. ....(((......)))......(((((.. .....)))))))))))))..	(((((((....(((((..((.....))..).))))............(((((.......)))))))))))))..	(((((((..(((.....)))...((((((((.......)))).)))). ((((((.......))))))))))))) ..	(((((((......((.((((.... .((......)).....)))).)). .(((((.......)))))))))))))..	(((((((..((((.........))))...(((.......))).((.(((((....))))).))...))))))). .	(((((((.......(((.....))).(((((........)))))... ..(((((........)))))))))))))..	((((((((((..((((.(... ...)))))..))).......... .....(((((.......))))))))))))..	(((((((..((((.........))))....(((.......... ..)))(((((.......))))))))))))..	(((((((..((((.........))))..((((((...((....)). ....)))))))........)))))))..
3WQY	(((((((.......((((((((((((.(((((((((...))))))))).))..))))...))))))))))))))....	(((((((..((((.......)))).((((((((...)))))))).....(((((.......))))))))))))))....	(((((((.......((((((((((.((((.........(((....)))))))))))...))))))))))))).. ..	(((((((..(((..((......)) .(((((((...)))))))).))). ((((((.......))))))))))))	((......((((.(((((((((((((.((((((((...))))))))).))..))))...)))))).))))...)).	(((((((.......((((((((..... ((((((((...)))))))).((((.. .))))....))))))))))))).. ..	(((((((.......((((((((((..(((....)))..((((...))))..))))...)))))))))))))))....	((((.....)))).(((((((((((((.((((((((...))))))))).))..))))...))))))............	(((((((.......((((.... ...((((((...))))))). ..))))(((.......)))..)))))))....	(((((((..((...((((..... ..)))).....))..(((....))) (((((.......))))))))))))....
3WQZ	(((((((.......((((((((((((.(((((((((...))))))))).))..))))...))))))))))))))....	(((((((..((((.......)))).((((((((...)))))))).....(((((.......))))))))))))))....	(((((((.......((((((((((.((((.........(((....)))))))))))...))))))))))))).. ..	(((((((..(((..((......)) .(((((((...)))))))).))). ((((((.......))))))))))))	((((((((.......(((((((... ..(((((((...))))))).((((...)))).....)))))))))))))....	......((.((((.((((((((((((.((((((((...)))))))).))..))))...)))))).))))..))..	(((((((.......((((((((((..(((....)))..((((...))))..))))...)))))))))))))))....	(((((((.......((((.... ...(((((((...))))))). ..))))(((.......)))..)))))))....	(((((((..((...((((... ....))))....))..(((... .)))(((((.......)))))))))))).....	(((((((.(((((.((((..... ..(((((((...)))))))...)))))))))..........))))))))....
484D	(((((((..((.......)))))))))	(((((((..(....)..)...))))))	(((((((((((....)))...)))))))	((((((.(((.........)))))))))	(((((((....((....)).)))))))	(((((.((((.........)))))))))	(((((..(.((.......)).))))))	((((((((..(....)..)..).)))))	(((((((......(.....))))))))	((((..((.((.......)).))))))
4A4S	(((((((((....)))))))))	(.((((((((....)))))))).	((.((((((....))))))))).	((((((((.....)...))))))	(((((((.....))...)))))	(((((.(((....))))).)))	(((.(((((....))).)))))	......(((((.....))))).	......((((((...)))))) .	......(((((.....))).))
4AL5	.(((((.....)))))	/	/	/	/	/	/	/	/	/
4AL7	(((((.....)))))	/	/	/	/	/	/	/	/	/
4BW0	(((...(((....)))......))).	.(((..(((....)))......))).	..(((.(((....)))......))) .	((....(((....)))......)). .	...((.(((....)))........)).	(((.....((.....)).....))) .	.((....))....((........)).	.((....))....((.......))..	((.....))....((........)).	/
4C4W	..((((......))))......((((....)))))..	((((((......))))..)).((((.. ..))))..	((((..(((.........)))..)))).........	........((....))......((((....))))..	(((((.(((((..........)))))..))..)))	..((....))..........((((. ...))))..	......(((.(((((.((....)).)))))..)))	..((((......))))....((......)).....	..((((......))))..((.. ....)).......	...(((..(((..........))))))........
4C7O	.((..(((.((((((((((((....)))).))).))))...)))..)).	.((((....((((((((((((....)))).)).)))))..)))).....	.((.((....)).)).((((....))))..((((((.......))))).	........((((((((((((....)))).)).))))).((.....)).	.....(((.((((((((((((.... .)))).)).)))))).......))).	.((..(((.(((((...((((... ...)))).)))))...)))..)).	.....((......)).((((....))))..((((((.......))))) .	((.......(((((((((((. ...)))).)).))))))..)).	.((((.........))))((.. ..))...((((((.......))))))	........((.((((...)))).))....((((((.......))))))
4ILM	.(((.........))).	.....((((...))))	/	/	/	/	/	/	/	/
4KR7	(((((((.......(((((....)))))))))))))....	((((((((.((.(((...))).)).. .)))))))))....	((((((((..(((((...)))...)).))))))))....	.........(((((((((((....))))....)))))))..	((((((((..((...((....)) .)).))))))))))....	....((....((..(((((....)))))..))....)).	........((((((..(((.... ....))))))))))..	.............(((((....)))))...((...)).	....((((...)))).(((... .))).((....))....	((.......))((((((((....))))....))))....
4KR9	(((((((.......(((((....)))))))))))))....	((((((((.((.(((...))).)).. .)))))))))....	((((((((..(((((...)))...)).))))))))....	.........(((((((((((....))))....)))))))..	((((((((..((...((....)) .)).))))))))))....	....((....((..(((((....)))))..))....)).	........((((((..(((.... ....))))))))))..	.............(((((....)))))...((...)).	....((((...)))).(((... .))).((....))....	((.......))((((((((....))))....))))....
4L8H	((((.((((...))))))))	....((((........))))	/	/	/	/	/	/	/	/
4M4O	((((((.(((((.((..((...((.(((. ..))).))...))..)).))))).))))))	((((((.(((((.........(((((. ....)))))..))))).))))))	((((((.(((((......(((((. .....))))).((.....))...))))).))))))	((((((.((((((((....)))((((..............))))...))))).))))))	((((((.((((((((...((((......))))))).......... .))))).))))))	((((((.((...((.........)). (((((...(((....)))..))))))).))))))	((((((.((((((((........ ...)))..(((((....))).)) .))))).))))))	((((((......((........))...((((...((((...... .))))))))))))))	((((((...........(((((......)))))...(((.... .....))).))))))	.(((((....))))).(((..(((((..............)))))..))).........
4M6D	((((..((.((((((......)))))..)). .))))......	(((.((..((...((.(((...))).))...))..)).)))	(((.........(((((......... .....)))))..)))	((((((....)))((((....... ....))))...)))	(((((((...((((......)))))))....))))....	(((......(((((......))))) .((.....))...)))	(((.........(((((....((...))...)))))..)))	(((.((....)))))...(((...(((....)))..)))..	((((((.........))....... (((((....))).)).)))	((.((.........)).(((..... ...)))...))......
4OOG	((((((((((((((....))))))))))))))..	...((((((((((.........)))))))))))	...((((((((((..((.....)))))))))))	(((((((((.........))))))))).......	.....((((((((....)))))))))((...))	((((.......))))...(((((... ...))))).	........((((((...((((.. .))))))))))	....((((...))))..(((((......))))).	(((...)))........(((((......))))).	.((.((((...))))))(((((. .....))))).

PDB	DNA model: mfold MFE	subopt #2	subopt #3	subopt #4	subopt #5	subopt #6	subopt #7	subopt #8	subopt #9	subopt #10
4P3E	/	/	/	/	/	/	/	/	/	/
4P8Z	/	/	/	/	/	/	/	/	/	/
4PDB	/	/	/	/	/	/	/	/	/	/
4PMI	/	/	/	/	/	/	/	/	/	/
4QI2	/	/	/	/	/	/	/	/	/	/
4QIL	/	/	/	/	/	/	/	/	/	/
4QOZ	/	/	/	/	/	/	/	/	/	/
4TV0	/	/	/	/	/	/	/	/	/	/
4TZV	/	/	/	/	/	/	/	/	/	/
4U7U	/	/	/	/	/	/	/	/	/	/
4WC2	/	/	/	/	/	/	/	/	/	/
4WC3	/	/	/	/	/	/	/	/	/	/
4WJ3	/	/	/	/	/	/	/	/	/	/
4X0A	/	/	/	/	/	/	/	/	/	/
4X0B	/	/	/	/	/	/	/	/	/	/
4X4O	/	/	/	/	/	/	/	/	/	/
4X4P	/	/	/	/	/	/	/	/	/	/
4YCO	/	/	/	/	/	/	/	/	/	/
4YVI	/	/	/	/	/	/	/	/	/	/
4YVJ	/	/	/	/	/	/	/	/	/	/
4YVK	/	/	/	/	/	/	/	/	/	/

PDB	RNA model: mfold MFE	subopt #2	subopt #3	subopt #4	subopt #5	subopt #6	subopt #7	subopt #8	subopt #9	subopt #10
4P3E	(((((...((((((((((.(((((..((((((....))))))..))))).))))...…((((.((.....))((((....(((....)))....))))....)))).)))))))....)))))	(((((...(((((((.....(((((..((((((....))))))..)))))((((((((......)))).))))((((....(((....)))....)))).........)))))))....)))))	(((((...((((((((((.((((((..((((((....))))))..))))).))))....((((..((...(((((....)))))..)).((.....)).…..)))).)))))))....)))))	(((((...(((((((......((..(((.((((....))))((....)).)))..))...((((.((....)))((((....(((....)))....))))....)))).)))))))....)))))	(((((...(((((((.....(((((..((((((....))))))..))))))..(..(((......)))..).(((((....)))))....((((..........)))).)))))))....)))))	(((((.....(((.......((((((..((((((....))))))..))))))((((((((......)))).)))).)))..(((((....((((..........))))....))))))..)))))	(((((...(((((((.....(((((..((((((....)))))).....)))))..((((((......((((......))))...(((....)))...))).)))........))))))....)))))	..........((((......(((((..((((((....))))))..)))))...))))...((((.((....))((((....(((....)))....))))....)))).((((....))))...	(((((...(((((((........((((((((.(((...((....))....(((((((((......)))).))))..))).)).((....))....)))))).......)))))))....)))))	(((((.((((((((......((((((..((((((....))))))..)))))))...))))........((...(((((....)))))..)).))))).(((((..((....))..)))))..)))))
4P8Z	(((((..((((((.(((((((((.......))))))))).((((((...(((((((..(((((....((((((((.....))))).......((.((....))))....))))...)))))......))))))))...............((((....))))..))))))))).))))))..)))))	(((((..((((((.(((((((((.......))))))))).((((((.((.......((((.((((..(((((((((.....)))))......))))..)))).....((((.(((....(((...))).....))).))))))))......)).((((....))))..)))))))).))))))..))))))	(((((..((((((.(((((((((.......))))))))).((((((.........((((.((((..(((((((((.....)))))......)))).))))...((((.(((....(((...)))......))).))))......))))..((((....))))..))))))).))))))..)))))	(((((..((((((.((((((((........)))))))).((((((((.((((......(((((....((((((((((.....))))........((.((....))))....))))...))))).(((.....))))))).))..........((((....))))..)))))).))))))..)))))	(((((..((((((.(((((((((........)))))))).((((((...((((((((..(((((....((((((((.....))))........((.((....))))....))))...)))))......)))))))........((((....))))(((....))))...)))))))).))))))..)))))	(((((..((((((.(((((((((.......))))))))).((((((...............((((..(((((((((.....)))))......))))..)))).((....(((((...(((...)))......((.....)).......)))))..(((....))).)).)))))).))))))..)))))	(((((..((((((.(((((((((........)))))))).((((((((...((((((((..((((((..((((((((.....))))))......))))..))))......(((.((....))..)))))..))))))))).................((((....))))..))))))).))))))..)))))	(((((..((((((.(((((((((.......)))))))).(((((((...((((((((...((((.........((((.....))))).......((.((....))))...))))....(((...))))..))))))))...............((((....))))..)))))))).))))))..)))))	(((((..((((((.(((((((((.......)))))))).(((((((...((((((((..(((((......((..((((((...........)))))).)).(((......)))..)))))......))))))))..............((((....))))..)))))))).))))))..)))))	(((((..((((((.(((((((((.......)))))))).((((((((.((.......((((((((..((((((((.....)))))......))))..))))..........(((((..((((.....))))))))))))))......)).((((....))))..))))))).))))))...)))))
4PDB	((((((((....((((....))))....))))))))))	(((((((((..((((((....))))..))))))))))))	(((((((((.(((.(((....))))))..)))))))))	(((((((((...((....)).((...)).)))))))))	(((((((((..((((..((...)).)))))))))))))	(((((((((..(((....))).)).......)))))))	(((((((.........(((...((...)))))))))))	(((((.........)))))..(((((.........)))))	(((((.....((.((((....))))......)))))))	.((....))....((((....))))....(((...)))
4PMI	((((((........(((((....))).))......))))))	((((((...((..(((((....))))).))...)).))))))	((((((.(((...(((((....))).)))))...))))))	((((((....((.....))(((....))))......))))))	((((((...((....))..(((....)))......))))))	..........(((.((...(((....))).....)).)))	((((((...((..(((......))).)).......))))))	..........(((.((.(((((....)))...)))).)))	((((((((......(((((....))).))))))....))))	((((((....(((.....))).(((....)))..))))))
4QI2	.(((((((...))))))).	.......((((....))))	/	/	/	/	/	/	/	/
4QIL	.((((((...))))))	..((((......))))	.(((((....)).)))	.((.(((...))))).	(((.(((...))))))	/	/	/	/	/
4QOZ	.....((((((....))))))....	.....(((((.(...))))))....	.....((((.(.....))))).....	.....((((((....)))))....).	.....((((((....))))....)).	.....((((((....)))))...)..	.....((((((....))))...))..	.....((((..(....))))).....	.....(((..((....)))))....	.....((..(((....))))).....
4TV0	.......((((((....))))))...	((.....((((((....)))))).))	.......(((((.(...))))))....	((((...((......))..))))...	(((((((((....))))...)))))...	((((((((...))))....)))))...	((((..(((....)))...))))...	((((...))))........((....))	.......((.(((.....)))))....	((((...((.....))...)))))...
4TZV	.....((((........)))).(((((((...)))))))......(((((........))))))...((((....))))	(((..((((........)))).(((((((...)))))))......(((((.......))))))))((((....))))	.....((((.......)))).(((((((...)))))))(((.((.(((......))))).)))((((....))))	.....((((.......))))....(((..(.....)..)))...(((((.......)))))...((((....))))	..........(((......)))..(((..(.....)..)))...(((((.......)))))...((((....))))	.....((...(((((.....)))))...))..((..(((..(((......)))..)))..)).((((....))))	(((...))).(((......)))..(((..(.....)..)))...(((((.......)))))...((((....))))	...........((((((.........)))))).(..(((..(((......)))..)))..)..((((....))))	..........(((((.....))))).(((((.((..(((..(((......)))..)))..))..))))))......	............((..((((.((.(((..(.....)..))).....))))))....))......((((....))))
4U7U	...((((((.(.....)))))))...........................((((((....))))))	...((((((.(.....)))))))....(..((...))..)......((((((....))))))	........(((((..((((((((....))))))).......)))))((((((....))))))	...((((((.(.....))))))))).((((.......))))......((((((....))))))	...(((.((....)).((((((....))))))...))).......((((((....))))))	........(((....(((((((....)))))))..)))........((((((....))))))	..............((.((((((((.....)))))..))).))..((((((....))))))	...(((.((....)).((((((....))))))..........)))((((((....))))))	...((((((.(.....))))))).........(((.......)))((((((....))))))	........((..((....))..))......................((((((....))))))
4WC2	(((((((..((((........)))).(((((.......)))))......(((((.......))))))))))))...	(((((((..((((........)))))......(((......))).....(((((.......))))))))))))...	(((((((....(((((..((.....))..).))))............((((.......)))))))))))...	(((((((..(((.....)))...((((((((.......)))).))))).(((((........))))))))))))...	(((((((.....((.((((.....((......)).....)))).))..(((((.......)))))))))))...	(((((((..((((........))))...(((.......))).((.(((((....))))).))...)))))))....	(((((((.......(((.....))).(((((.......)))))....(((((.......)))))))))))...	((((((((((..((((.(.....)))))..))).............(((((.......))))))))))))...	(((((((..((((........))))....(((............)))(((((.......))))))))))))...	(((((((..((((........))))..((((((...((....))......))))))........))))))))...
4WC3	(((((((..((((........)))).(((((.......)))))......(((((.......))))))))))))....	(((((((..((((........)))))......(((......))).....(((((......))))))))))))....	(((((((....(((((..((.....))..).))))............((((.......)))))))))))))....	(((((((..(((.....)))...((((((((.......)))).))))).(((((.......))))))))))))....	(((((((.....((.((((.....((......)).....)))).))..(((((.......))))))))))))....	(((((((..((((........))))...(((.......))).((.(((((....))))).))...)))))))....	(((((((.......(((.....))).(((((.......)))))....(((((.......)))))))))))....	((((((((((..((((.(.....)))))..))).............(((((.......))))))))))))....	(((((((..((((........))))....(((............)))(((((.......))))))))))))....	(((((((..((((........))))..((((((...((....))......))))))........))))))))....
4WJ3	(((((((..(((((((.(((..((((.....)))).))))))))))).(((((........))))))))))))....	(((((((..((((........)))).(((((.......)))))......(((((.......))))))))))))....	(((((((..((((........))))....((((...........))))(((((.......)))))))))))))....	(((((((.....((((((.........))))))..............(((((.......))))))))))))....	((((((((.((((........))))....((((...(((((..((...))))))..))))...)))))))))....	(((((((..((((........))))......(((....((((..(....)..))..))...))).)))))))...	(((((((..((((........))))...........(((....))).(((((.......))))))))))))....	((((((((.(((((..(((..((...((((((.......)))))...)).)))..).)))).....))))))))....	((((((((.((((........))))................((((............)))).))))))))....	((((((((((..(((((..........))))))))...........(((((.......)))))))))))....
4X0A	((((((..((((........)))).(((((.......)))))......(((((.......))))))))))))...	((((((..((((........)))).....(((......))).....(((((.......))))))))))))...	((((((....(((((..((.....))..).))))............(((((.......)))))))))))...	((((((..(((.....)))...((((((((.......)))).)))).(((((.......))))))))))))....	((((((.....((.((((.....((......)).....)))).))..(((((.......))))))))))))...	((((((..((((........))))...(((.......))).((.(((((....))))).))...))))))...	((((((.......(((.....))).(((((.......)))))....(((((.......)))))))))))...	(((((((((..((((.(......)))))..)))............(((((.......))))))))))))...	((((((..((((........))))....((((............)))(((((.......))))))))))...	(((.....)))....((((((.((((((((.......)))).)))).(((((.......))))).))))))..
4X0B	((((((((..((((........)))).(((((.......)))))......(((((.......))))))))))))))...	((((((((..((((........))))......(((......))).....(((((.......)))))))))))))...	((((((((....(((((..((.....))..).))))............(((((.......)))))))))))))...	((((((((..(((.....)))...(((((((((.......)))).)))).(((((.......))))))))))))))...	((((((((.....((.((((.....((......)).....)))).))..(((((.......)))))))))))))))...	((((((((..((((........))))...(((.......))).((.(((((....))))).))...)))))))))...	((((((((.......(((.....))).(((((.......)))))......(((((.......)))))))))))))))...	(((((((((((..((((.(......)))))..)))..............(((((.......)))))))))))))...	((((((((..((((........))))....(((.............)))(((((.......))))))))))))))...	((((((((..((((........)))))..((((((...((....)).....))))))........))))))))...
4X4O	((((((((((((.......))))))))))...)))	.((...((((((.......))))))))).......	......((((((.......))))))((....)).	(((....((.(((.........)))))....)))	.((.((((..(((....))))))).)).......	......(((.((((((.........)).)))))))	(((......(((.......)))))).........	.((....)).(((.........)))........	.((....)).((((((..........)))..)))	(((..(((.....)))......)))........
4X4P	((((((((((((.......))))))))))...)))..	.((...((((((.......)))))))).........	......((((((.......))))))((....))...	(((....((.(((.........)))))....)))..	......(((.((((((.(.....))).))))))..	(((...(((.........)))..)))...........	.((....)).((((((.(.....)..)))..)))..	.((.((((...........)))).))..........	.((....)).(((....((......))....)))..	.((....)).((..(((.((.......))))))..
4YCO	(((((((..((((.........)))).(((((((...)))))))......(((((.......))))))))))))..	((((((...((((((((((.((..(((((((((...))))))).))..))...))))))..))))))))))..	((((((((.((((........))))..((((((((.....(((......)))))))))))..))))))))))..	((((((((.((((........)))).(((((((...)))))))).............(((....))))))))))))..	((((((((.......((((..((((..((((((((........)).)))))..))))....))))))))))))...	((((((((.((((........)))).(((((((...))))))))..(((........)))......))))))))))..	((((((((((((((........)))).(((((((...)))))))).)))))..(..(((....)))..).))))...	(((((((..(((.....)))...((((((((((...))))))).))).(((((.......))))))))))))..	(((((((((((((........)))).(((((((...))))))).)))))........(((.....))).)))))..	((((((((.((((........))))...((((......(((.(((......)))...)))))))..)))))))))..
4YVI	(((((((.(((((.......)))))..((((((..(.((((....))))..)..))))))...))))))).	(((((((.........((.(((((..((((((.....((((....))))))))))).))))))))))))).	(((((((................((((((((.......)))))))..(((((.......)))))))))))).	(((((((..((((..((......))..)))).(..(.....)..).((((((.......)))))))))))).	.((((((((((((.......))))))).(((((..(.((((....))))..)..))))))))))........	(((((((.........((((.((((((......)))..)))))))..(((((.......))))))))))))).	((((......))))..((.(((.(((((((.......)))))))...(((((.......)))))))).))..	(((((((..((((..((.......))..))))...(((..((((......))..))..)))...))))))).	((((.((((((((..((.......))..))))....))))....((.(((((.......)))))).)))).	.((((((...((.(((........(((((((.......)))))))......))).))..)))))).......
4YVJ	(((((((.(((((.......)))))..((((((....((((....))))......))))))...))))))).	(((((((.........((.(((((..(((((.(((.(((....))).))))))))).)))))))))))))).	(((((((................((((((((.......)))))))..(((((.......)))))))))))).	(((((((.(((((.......)))))..((((((..((...(((......))))))))))...))))))).	(((((((..((((..((......))..)))).(((((((((.....))))...)))))......))))))).	.(((((((((((((.......)))))))).(((((....((((....))))......))))))))))).......	((((......))))..((.(((.(((((((.......)))))))...(((((.......)))))))).))..	(((((((....(((.((((((((.........)))))..))).))).(((((.......))))))))))))).	(((((((........((((.(((((((.......))).)))))))..(((((.......))))))))))))).	((((.((((((((..((......))..))))....))))....((.(((((.......))))))).))))).
4YVK	(((((((.........((.(((((..(((((.(((.(((....))).)))))))).))))))))))))))).	(((((((.(((((.......))))).((((((....((((....))))......))))))...))))))).	(((((((................((((((((.......)))))))..(((((.......)))))))))))).	(((((((..((((..((......))..)))).((((((((((....))))...)))))......))))))).	.((((((.(((((.......))))).(((((.(((.(((....))).)))))))...)))))).......	(((((((.(((((.......)))))..((((((..((...(((......))))))))))))...))))))).	(((((((.........((((.((((((.......))).)))))))...(((((.......)))))))))))).	(((((((((((((.......))))))))...))))))).....((((.(((((.......)))))....))))	((((......))))..((.(((((..(((((.(((.(((....))).)))))))).)))))).)).......	((((......))))..((.(((.(((((((.......))))))..(((((.......)))))))).))..

DNA model

PDB	mfold MFE	subopt #2	subopt #3	subopt #4	subopt #5	subopt #6	subopt #7	subopt #8	subopt #9	subopt #10
4YYE	/	/	/	/	/	/	/	/	/	/
4Z0C	/	/	/	/	/	/	/	/	/	/
4Z7L	/	/	/	/	/	/	/	/	/	/
4ZLD	/	/	/	/	/	/	/	/	/	/
4ZT0	/	/	/	/	/	/	/	/	/	/
5A18	/	/	/	/	/	/	/	/	/	/
5CCB	/	/	/	/	/	/	/	/	/	/
5D6G	/	/	/	/	/	/	/	/	/	/
5F5F	/	/	/	/	/	/	/	/	/	/
5F5H	/	/	/	/	/	/	/	/	/	/
5F9F	/	/	/	/	/	/	/	/	/	/
5FJ4	/	/	/	/	/	/	/	/	/	/
5FMZ	/	/	/	/	/	/	/	/	/	/
5HR6	/	/	/	/	/	/	/	/	/	/
5HR7	/	/	/	/	/	/	/	/	/	/
5ID6	/	/	/	/	/	/	/	/	/	/
5IEM	/	/	/	/	/	/	/	/	/	/
5IWA	/	/	/	/	/	/	/	/	/	/
5KH8	/	/	/	/	/	/	/	/	/	/
5KMZ	/	/	/	/	/	/	/	/	/	/
5KQE	/	/	/	/	/	/	/	/	/	/
5L1Z	/	/	/	/	/	/	/	/	/	/
5LM7	/	/	/	/	/	/	/	/	/	/
5LSN	/	/	/	/	/	/	/	/	/	/

RNA model

PDB	mfold MFE	subopt #2	subopt #3	subopt #4	subopt #5	subopt #6	subopt #7	subopt #8	subopt #9	subopt #10
4YYE	((((((((.((((........)))).........((((....)))).((((......))))).))))))))..	(((((((((..........((((((....)))))).((((....)))).((((......)))))))))))))..	((((((((.((((........)))).((((.........))))......((((......)))).))))))))..	((((((((.((((........))))......(((((....)))))......((((......)))).))))))))..	((((((((((.....(((((((((....)))))...)))))..((((((.....)))))).))))))))))..	((((((((.((((..((((((((((....)))))...))))).))))..((((......)))).)))))))))..	((((((((((........((..((((.((((......((((....))))))))))))....))))))))))))..	((((((((.(((.....)))(((((....)))))..((((....)))).((((......)))).))))))))..	(((((((((............((((.((((.........))))).))))((((......))))))))))))..	((((((((((.....(((.((((((....)))))).((((....))))).......)))....))))))))))..
4Z0C	..((......)).	/	/	/	/	/	/	/	/	/
4Z7L	((..........))	/	/	/	/	/	/	/	/	/
4ZLD	(((((((...)))))))	/	/	/	/	/	/	/	/	/
4ZT0	((((((((((((((((.((((....))))...)))))))...))..)))..))))).((((....))))..	((((((((...........((((....)))).((((.......)))).)))..)))).((((....))))..	(((.((....(((((((.((((....))))...)))))))....)).)))........((((....))))..	..(((...(((..((((.((((....))))..((....))....))))..)))..)))((((....))))..	......((((.((((...((((....)))).((((...((((((.....)).)))).)))))..)))).))))	..(((((....)))))..((((....))))............((.....))........((((....))))..	((((((((...(((((....))))).((((.............)))))))..))))).((((....)))))..	......((((.........((((....))))..........((((................))))......))))	((((.....))))......((((....))))............((.....)).......((((....))))..	..(((...(((..((((..((.....((((....)))......)).))))..))).)))((((....))))).
5A18	((((((((...((........))..))))))))	(((((((((......(((....)))))))))))	((((((((((..........))......)))))))	((((((((((......)......)).)))))))	........(((...(((....)))...)))...	...((...(((...(((....)))...)))))	..((....))......................	..((....))........((.......))...	/	/
5CCB	.(((((((..((((.........))))....((((((.....)))))))(((((.......))))))))))))....	.(((((((..((((.........))))).(((((.......)))))......((((((.......))))))))))))....	.(((((((((((((.........))))....(((((....((((......))))....)))))..))))))))))....	.(((((((...(((((.....((((.......))))....)))))....((((((........))))))))))))....	.((((((((((.......((..((((....(((((((.....)))))))....))))....))))))))))))....	.((((((((.((((.........)))).(((((.......)))))..)))(((((.......))))..))))))....	.((((((((((.......((....((.((((((.......)))))..)))))).(((......))))))))))))....	(((((((((.((((.......)))).(((((........)))))..)))).)))))...((((......))))....	.((((((((((.......((((.....))).(((((....((((......))))....))))).)))))))))))....	.((((((((.((((.........))))).(((((.......)))))(((..............))).))))))))....
5D6G	((((.......((((((((.(((((.......))))).))))....((((.........)))).)))))..))))	((((.......(((((..((((((((....))).((((..((....))..)).))..)))))..)))))..))))	((((..((((((..(((((.(((((.......))))).)))))......((.....)).)))......))).))))	((((.......(((((..(((((........(((.......(((....))))))....)))))..)))))..))))	((((.......(((((..(((((((((.((((.........))))...))))......)))))..)))))...))))	((((.......((((((((..(((((.......)))))....(((.........)))...))).)))))..))))	((((.......(((((..(((((..(((...(((.......((((....))))).))))))))..))))))..))))	(((((((.((((.......)))).)))))..)).(((.((.......((((.........))))........)))))	((((...((.((..(((((.(((((.......))))).)))))......))..))....((......))..))))	((((..(((.((.....(((....)))....))...............((((.........))))....))).))))
5F5F	(((((..........)))))	/	/	/	/	/	/	/	/	/
5F5H	(((((((......)))).)))	...((((.......)))...).	...((((.......))...)).	/	/	/	/	/	/	/
5F9F	((((((((((....))))))))))	((.((((((((....)))))))))).	(((((((((....))..)))))))	/	/	/	/	/	/	/
5FJ4	...((((.((....)).))))..............	(((...(((((..........))))).......)))	(((((....)).((((((....))))))....)))	....(((.....((((((....))))))....)))	...((((.((....)).)))).((.......))..	(((....((.(((((.....))))))).......)))	...((((.((....)).))))......((.....))	((.((....)).))((((.....))))..........	(((.((.......))((((....))))......)))	(((...(((....))).))).............
5FMZ	.((....))...	/	/	/	/	/	/	/	/	/
5HR6	(((((.......(((.(((.....((((((.......))))))....))).)))...........)))))	((((...(((....)))........(((((((........))))))...(((((.......))))).))))	...((((.....))))(((.....(((((((........))))))...((((((.......))))).))).	((((...((((..........))))(((((((........))))))...(((((........))))).))))	(((((..(((....)))........(((((((........))))))...))))).......(((....)))	..(((..(((....)))..)))..((((((((........))))))...((((((........)))))).....	((((.(((((.(((((....((...))..)))))...))))).....(((((........))))).))))	((((.(((((..(((....(((.......))))))..)))))....(((((........))))).))))	(((((.......((.((....(((((((((........)))))).......))))....)).))))))))	((((...........(((.(((.......))).....)))........(((((........))))).))))
5HR7	(((((.......(((.(((.....((((((.......))))))....))).)))...........))))))..	((((...(((....)))........(((((((........))))))...(((((.......))))).))))..	((((...((((..........))))((((((........))))))...(((((.......))))).))))..	(((((..(((....)))........(((((((........))))))...)))))......((((....)))).	...((((.....))))(((.....((((((........))))))...(((((........))))).)))...	((((.(((((.(((((....((...))..)))))...)))))....(((((........))))).))))..	.....((.(((((.(((((.....((((((.......))))))......)))........)).)))))).)).	.....((.(((((.((....((((((((((........)))))).......))))....)).))))).)).	((((.(((((..(((....(((.......))))))..)))))....(((((........))))).))))..	((((............(((.(((.......))).....)))........(((((........))))).))))..
5ID6	....(((((.....)))))..	...(.((((.....)))))..	.(..(((((.....)))))).	/	/	/	/	/	/	/
5IEM	((((((((((...(((..((((((((....))).))))....)))...))))))))))	(((((((((((.((....((((((....))).)))).)).))))......)))))))	((((((............((((((....))).))))..(((......))).))))))	((((((((((............(((.(((((....)))))..)))..)))))))))	(((..........)))...((((((((....))).))))).((((.((...)).))))..	((((((((((...((...((......)).(((((......))))))...))))))))))	((((((.((((......))))(((((...((((......)))....)))))))))))	(((..........)))..........(((....))).((((((.((....)).))))))..	(((((((((((.(((...((......))))).))).((((....)))).))))))))	.(((((.((((......)))).(((....))).)))))((((.((...)).))))..
5IWA	...(((..((.((((.(((..((((((((((.(((..(((((.(((..((((...(((..((((((.((((((((((.....))))))))))...(((.((.((.(((........((((((...((((.(((((((.((((((...((((((....))))))....))))))...(((((.(((((....))))).)))))..((((...)))).))))))).))))))))))))))).)).)))..((((((((.......))))))).)).)))))..((.(((((((.........))))))).)).(((((.(((.....))))))))).((((....))))...))).((((((.((((((.....((((....))))....(((((....)))))...))))).((((....))))......))))).((((((((....((((.....))))........(((((.((((.((((((((((..(((((((((.((....)))))...)))))))))).)).))))))((((((((.(..((((...((((((((..(((.((((((((..((((((((((......((((((.....))))))).....)))))))))..((((.(((...((((..(((..((((((((....((((((.((.(((.........)))..)).)))))))...((((...))))......)))))))..)))..)[illegible]	...(((.((.((((.((((((...)))))))))).)).(((((.(((((..(((......)))..((.(((((((((((.....)))))))))).))(((((((((((((.((...(((((((...((((.(((((((.((((((...(((((((....))))))....))))))...(((((.(((((....))))).)))))..((((...)))).))))))).)))))))))))((((((((....))))))))(((..(((((.....((.((((.(((((((((...((((....))))((((((((....((((((.(((.....))))))))).((((....))))...((..((((((.((((((.....((((....))))....((((((....)))))...)))))).((((....))))......)))))).(((((..((....((((.....))))......))...)))))......))))))))..((((((((((.((....)))))...)))))).((....))))))))).(((((((((((....(((((((..(((.(((((((..((((((((.......(((((.....)))))).....)))))))))..((((.(((...((((..(((..(((((([illegible]	...(((.((.((((.((((((...))))))))))).)).(((((.(((((..(((......)))..((.((((((((((.....)))))))))))).))((((((((((((.((...(((((((...((((.(((((((.((((((...((((((....))))))....))))))...(((((.(((((....))))).)))))..((((...)))).))))))).)))))))))))(((((((....))))))))(((..((((((.....((((((.(((((((((..((((((....))))((((((((....(((((((.(((.....))))))))).((((....))))...((..((((((.((((((((.....((((.....))))....(((((....)))))...))))))).((((....))))........)))))).(((((..((....((((.....))))......))...)))))......))))))))..((((((((.((....)))))...)))))).((....))))))))))(((((.(..((((...(((((((..(((.((((((..(((((((((......((((((.....)[illegible]	...(..(.((((((((((((((...(((((.....((((((((((.(((((....(((..(((((((.(((((((((((.....))))))))))))....(((.((.((.(((........(((((((((...((((.(((((((.((((((((...((((((....))))))....))))))...(((((.(((((....)))))).)))))..((((...))))).)))))))).)))))))))))))))).....)).)))..((((((((.......))))))))).)).)))))..((.(((((((((.........)))))))).)).(((((.(((.....))))))))).((((....)))))))....)))))....(((((...(((((....))))).)))))..))))))))....)).....))))))))))))).))))))))..).((((((((....((((((.....)))).........(((((.((((.(((((((((((..((((((((((.((....)))))...))))))))))).)).))))))(((((((((.(..((((...(((((((((..(((.(((((((..((((((((((......([illegible]	...(((.((.((((.((((((...))))))))))).)).(((((.(((((..(((......)))..((.(((((((((((.....)))))))))).))(((((((((((.((...((((((.(((((...(((((((.((((((...((((((....))))).....))))))....(((((.(((((((....)))))).)))))..((((...)))).)))))))..((((((.((((.......(((..((((((((........))))))))))))).....((((((((..((((....))))((((((((....(((((.(((.....))))))))).((((....))))...((..((((((.(((((((.....((((....)))....(((((....)))))...)))))).((((....))))......))))))).(((((..((....((((.....)))......))...))))).....)))))))))..((((((((((.((....)))))...))))))).((....))))))))))))).((((.(..((((((...((((((((..(((.((([illegible]	...(((.((.((((.((((((...)))))))))))).)).(((((.(((((..(((......)))..((.(((((((((((.....)))))))))))).))(((((((((((.((...(((((((...((((.(((((((.(((((((...((((((....)))))) ....)))))...(((((.(((((....))))).)))))..((((...))))).))))))))).)))))))))))))(((((((....))))))))(((..(((((((.....(((...(((..((.(((((((.........))))))).)).(((((.(((.....))))))))).((((....))))......))).((.....((((...((((....)))).((..(((((((((((((((..((.....((((....)))).....((((((..(((((..((....((((.....))))......))...)))))....(((((((((((..((((((((.((....)))))...))))))))).)).))))))....(((((.(..((((...(((((((..(((.((((((..((((((((......(((((([illegible]	...(((.((.((((.((((((...))))))))))).)).(((((.(((((..(((......)))..((.((((((((((.....))))))))))).))(((((((((((.((...(((((((...((((.((((((((.((((((...((((((....))))))...(((((.(((((....))))).)))))..((((...)))).))))))).))))))))))))(((((((((....))))))))(((..((((((.....(((((....((((.((((((((.........)))))))(((..(((((.((((...(((((((((.((((....))))...((.(((((...((((((.....((((....))))....(((((....))))).....))))))..(((((((((.((....(((((....)))))....))))))))..))))..))))).)).(((((....((((((((((..((((((((.((....)))))...))))))))).)).)))))))))).(((.(..((((...[illegible]	...(((..((.((((.(((..(((((.((((..((((.(((((((((.((..((..((..(((.....((((((((.((((.....(((((((....(((((.....((((.((...(((((((...((((.(((((((.((((((...((((((....))))))....))))))...(((((.(((((....)))))..)))).))))))).))))))))))(((((((((....))))))))))......)).))))..((((..(((.((...)).))).(.(((..((((((((....(((((.(((.....))))))))).((((....))))...((..((((((.(((((......((((....))))....(((((....))))))...)))))).((((....))))......)))))).(((((..((....((((.....)))).....))...)))))......))))))))))).)))).))))).....)))))......))))))))).[illegible]	...(((..((.((((.(((..(((((.(((......((((((((((.............(((((...((((((((((.....)))))))))).)))))).((((.((.(((...(((((((...((((.((((((.((((((...((((((((....))))))....))))))...(((((.(((((....)))))).)))))..((((...))))).)))))))).)))))))))))(((((((....))))))))))....((((((.(((.((.(((..(((((.((.(((.((.(((..((((((((....((((((.(((.....))))))))).((((....))))...((..((((((.((((((.....((((....))))....(((((....))))))...)))))).(((((..((....((((.....))))......))...))))).....)))))))))))).))))))..)))..)).))).))......(((((([illegible]	...(((..((.((((.(((..(.(((((((..((.(((((.((.(((((..(((......)))..((.((((((((((.....)))))))))).((((((.(((((.........((....))...((((((.((((((...((((((....))))))....))))))...(((((.(((((....))))).))))))..((((...)))).)))))))..((((((((.(((((........((((((((........))))))))(((..(((((((.((((((((.........)))))))....((((((.(((.....))))))).((((....))))...(((.((((((.((((((.....((((....))))....((((((....)))))...)))))).((((((....))))......)))))).(((((((((....((((.....))))........(((((.((((.((((((((((..((((((.((....)))))...))))))))).)).))))))((((((((((.(..((((...(((((((..(((.(([illegible]
5KH8	(((((......)))))......((((....))))..............	((..(((((....)))))....((((....))))........))...	(((((......))))).((...((((....))))...))........	(((((......))))).((...((((....))))))..........	(((((......)))))......((((....))))...((.....)).	......(((((....(((....((((....))))..)))))))...	(((((......))))).((...((((....))))..........)).	((.....(((.....))....((((....))))........))...	(((.(((((....)))))......((....)))))............	((..(((((....)))......((((....)))..))....))...
5KMZ	.......((((.(((........))).))))	(((((.........)))))..........	.......(((.....))).............	.......((((.....((....))...))))	.(((...(((.....)))..........)))	.....((((..(((.......)))..)))).	(((((.........)))))((......))..	/	/	/
5KQE	(((((((......(((((((....))))))))))))))	(((((((.((......(((....)))).)))))))))	(((....)))...((((((....))))))........	..((.......))((((((....)))))).......	(((((((.((..((.......))...)).)))))))	(((((((...((...))((....))....)))))))	............((((((....))))...))....	(((((((.((...(((......))).)).)))))))	((((((.........((((....))))))...))))	.(((...........((((....))))))......
5L1Z	.....((((......))))..	((...((((......))))))	.....(((.(.....))))..	.....(((.((...)))))..	...........(((....)))	/	/	/	/	/
5LM7	.............(((((.....)))))	....(((....)))(((((.....)))))	...((.......))(((((.....)))))	((((((..((.........))..)))))).	..(((.......)))((((.....)))).	.....(.......)(((((.....)))))	.((((((...........)))))).....	...((((....))))((((.....)))).	................(((......))).	(((((((...........)))))).))..
5LSN	((.((((((((((...))))))))))).))	.((((((((((((...))))))))))).))	...((((((((((...))))))))))..).	..((((((((((((...))))))))))..))	...((((((((((...))))))))))..)).	....(((((........)))).......	...((((((((((...)))))))))..))).	((.((((((....(.....))))))).))	((.(((((....((.....))))))).))	.....((((((((...))))))))....).

DNA model

PDB	mfold MFE	subopt #2	subopt #3	subopt #4	subopt #5	subopt #6	subopt #7	subopt #8	subopt #9	subopt #10
5M0I	/	/	/	/	/	/	/	/	/	/
5N5C	/	/	/	/	/	/	/	/	/	/
5NG6	/	/	/	/	/	/	/	/	/	/
5TF6	/	/	/	/	/	/	/	/	/	/
5UDZ	/	/	/	/	/	/	/	/	/	/
5UF3	/	/	/	/	/	/	/	/	/	/
5UZT	/	/	/	/	/	/	/	/	/	/
5V17	/	/	/	/	/	/	/	/	/	/
5V6X	/	/	/	/	/	/	/	/	/	/
5VW1	/	/	/	/	/	/	/	/	/	/
5W1H	/	/	/	/	/	/	/	/	/	/
5WLH	/	/	/	/	/	/	/	/	/	/
5WQ1	/	/	/	/	/	/	/	/	/	/
5WT1	/	/	/	/	/	/	/	/	/	/
5WT3	/	/	/	/	/	/	/	/	/	/
5X6B	/	/	/	/	/	/	/	/	/	/
5XBL	/	/	/	/	/	/	/	/	/	/
5Y58	/	/	/	/	/	/	/	/	/	/
6AAS	/	/	/	/	/	/	/	/	/	/
6B14	/	/	/	/	/	/	/	/	/	/
6B3K	/	/	/	/	/	/	/	/	/	/
6BHJ	/	/	/	/	/	/	/	/	/	/
6CYT	/	/	/	/	/	/	/	/	/	/
6D12	/	/	/	/	/	/	/	/	/	/
6DB8	/	/	/	/	/	/	/	/	/	/
6DTD	/	/	/	/	/	/	/	/	/	/
6DU4	/	/	/	/	/	/	/	/	/	/

RNA model

PDB	mfold MFE	subopt #2	subopt #3	subopt #4	subopt #5	subopt #6	subopt #7	subopt #8	subopt #9	subopt #10
5M0I	(((((.((..((....)))))))))...	(((((((........))...)))))...	......(((.((....)).....)))..	......(((.((....))..))).. ...	(((......)))...............	/	/	/	/	/
5N5C	((((((((...))))))))	(((((.(......))))))	.(.((((((...)))))))..	/	/	/	/	/	/	/
5NG6	....(((((....)))))......	((((((((....)))))..))))	..(((((((....))))).))...	((((((((((....)))))..)))))	....((((((((...))).)))))..	........(((((...)))))...	.(..(((((....)))))).....	...(.((((....))))).... ..	...((((((....)))))...)..	.((..((((....))))))....
5TF6	(((((.((((........))))..))))).((.((((.(((.....)))..)))).))..........	(((((.((((........))))..))))))....((((.(((.....)))..))))).(((((.....)))))	(((((.((((........))))..)))))).((((.....))))....((.....))..............	.(((..((((.........((....))...(((.((((.(((.....)))..)))).)))...))))..)))	.(((..((((((((......((.....))....((((.(((.....)))..)))).)))..)))..)))))	(((((.((((........))))..))))).((((.....))))...(((((((...))))))..........	(((..((((...........((.....)).((((.....))))....))))..)))...............	(((((.((((........))))..))))).((((.....))))).......((((....)))).........	.(((..((((((((....((....)).((((((.....))))).......)))...))))..)))))..)))	.(((..((((..........((((((.......((((.(((.....)))..)))))))))).))))..))))
5UDZ	(((((((.....))))))).....	((((((((....)).)))))....	(((.(((.....))))))......	(((((((...)..)))))).....	((((.........)..)))......	.((((((.....))).))).....	(((((((...))..))))).... .	(((((...(....))))))).....	((((........)...))).... ..	.(((..(((....))))))....
5UF3	(((((((.((....)))))))))	((((((.(((....)))))))))	((((((..((.....))))))))	(.(((((.((....)))))))))).	((((..((((....))).)))))	((.((((.((....)))))))).	(((..(((((....))).)))))	((..((((((....))).)))))	/	/
5UZT	(((((((.((((.(.....))))))))))))	(((((((.........((....)))))))))	(((((((.((((...)).....))))))))	(((((....((((.........)))))))))	((((((((.((.....((...)))))))))))	((((((((..........))).....)))))	........(((((..........)))))..	...................((....))..	(((....)))...........((....))..	(((...........)))...((....))..
5V17	(((((.....((((((((((......)))))))))))))))	((......)).(((((((((......))))))))......	((((((.....((((((((.....))))....)))))))))))	(((((((.....((((.....)))).(......).)))))))	(((((((((...)))(((((......))))).....))))))	((((((((.....((((.....)))).)).......))))))	((.......)).((((.....)))).((((......))))..	((((((((.....(((.(((....))))))......)))))))	(((((((..(((....))).((....))......)))))))	(((((.(((...)))..((.............))..)))))
5V6X	(((((((.((((......))))(((((((.......))))))))..(((((.......))))))))))))..	((((((((((((......))))....(((.(((((.((((......))))))))).))).))))))))..	(((((((((.......)))...(((((((.......))))))))..(((((.......))))).))))))..	(((((((.((......((((((.....)).))))........))..(((((.......)))))))))))))..	((((((((((((...((((((.....)).))))(((((((......))))).))))....))))))))..	(((((((.((((.....))))...(((...)))...........(((((......))))))))))))))..	((((((((.....((.(((..((..((((((.........))))))..)).)))..)).....)))))))..	((((.....((((.........(((((((.......))))))).(((((.......)))))))))))))..	(((((((((((((......))))((((.....((((.((((......))))))))))))...)))))))))..	((((((((......((..(((((((((((.......))))))).........)))..))))))))))..
5VW1	..........(((((((.((((.....))))...)))))))..((.....)).......((((....))))...	.........(((((((..((((.....))))..........)))))))..........((((.....))))...	..((((....(((((((.((((.....))))...))))))))..((.....)).))))..((((.....))))...	...................((((....))))).((((........))))............((((.....))))...	..........(((((((.((((....))))...)))))))..(((..............((((.....))))))))	........(((((......((((....))))............((.....))......)))))..........	...(((((...)))))..((((....))))............((.....)).......((((.....))))....	..........(((((((.((((....))))...)))))))..(((........(((......))))......)))	...........((((((.((((....))))............((.....))..........)))))).......	..........(((((((.((((....))))...)))))))...(((..((.(((...)))..))...)))...
5W1H	......((((.........))))......(((.......)))	...((.((((.........)))).))....(((.......)))	.................((((.(((......))).)))).	.((...((((.........)))).(((......)))...)).	...(((((((.........)))).........)))......	......((((.........))))..........((......))	.......((((.........))))...((..............))	.................((.......))..........	....((((((.........)))).(((......)))....))	..........((.........(((.(((......))).)))))
5WLH	.......((((.........))))......(((.......)))	....((.((((.........)))).))...(((.......)))	.................((((.(((......))).)))).	..((...((((.........)))).(((......)))...)).	....(((((((.........))))..........)))......	.......((((.........)))).........((......))	.......((((.........)))).((..............))	.................((.......))..........	.....((((((.........)))).(((......)))....))	..........((.........(((.(((......))).)))))
5WQ1	(((((((((.....)))))))))	(.((((((((.....)))))))).	((.((((((.....)))))))).	((((((.....(....)))))))	(((((.....((....)))))))	(((.........))).......	((((.....(((....)))))))	(((.((...)).)))........	..((..(((.....))))).....	........(((.........))).
5WT1	.(((...(((((.((((.......)))).))).))..))).(((..((((((......)))))..)))	((((..((((.........)))).(((((.......)))))......(((((......))))).))))	.((((.((((..........))))))))...........((((....(((((......))))))))).	..(((......))).(((...((((((((.......))).))))).(((((......)))))))))..	((((.......((((((.....((......))....)))))))...(((((......))))).))))	((((...((((...))))...((((((((.......))).))))).(((((......))))).))))	.((((.((((.........))))))))..(((......)))(((..(((((......)))))..)))	((((.......(((.....).)).(((((.......)))))....(((((......))))).))))	(((...((((.........)))).(((((........))))).)))((((((.........)))))).	((((..((((..........)))))...(((.((((..((((.....))))..))))..)))...))))
5WT3	(((((((..((((..........)))).((((.......))))).....(((((.......))))).)))))))	(((((((.....((((.((((.......)))).)))).............(((((......))))).)))))))	(((((((.......((((((((....((......))....)))))))...(((((......))))).)))))))	(((((((...(((.....)))...((((((((.......))).))))).(((((......))))).))))))))	((..((((.((((.........)))))))..)).........(((((..(((((......)))))..))))).	(((((((.......(((.....).)).(((((.......)))))......(((((......)))))).)))))))	((((((((......))).(((...(((((((.......))).)))).(((((......))))))))))))))	((((((.....((((.((((.......)))).))))......(((....((((.......))))))))))))	(((((((..((((.........))))...(((.((((..((((.....)))..))))..)))).....)))))))	(((((((..((((.........))))......(((......)))....(((((......))))).))))))))
5X6B	(((((((..((((((...)))))).(((((........)))))......(((((........)))))))))))))).....	(((((((.......(((((...((((....))))......)))))..(((((.......)))))))))))))).....	((((((((((((....))))).....(((((.......)))))......(((((.......))))))))))))))...	(((((((..(((((((...)))))))...(((.....(((((((.....))))))))....)))...)))))))).....	(((((((..((...((((....((((....)))).((((((((.....))))))).....)))))))))))))))....	(((((((.((((....))))(((((..(((((.......))))))))..((((........))))))))))))))...	(((((((((((((.....(((....))))))))))).............(((((........))))).)))))))....	(((((((..((((((...))))))).(((....((......))....)))((((.......)))..)))))))....	...(((....))).(((((..((..(((((........)))))).....(((((........))))).))..))))).	((((((((((((....)))))).((((....))))).(((..((((.....))))....)))....))))))))....
5XBL	.(((.((((((((((((.((((....))))...)))))))...)))))..)))...(((((....)))).((((((...)))))))..	...((((...((((((((.((((....))))...))))))).))))..............((((....)))).((((((...))))))..	......(((((..((((.((((.....))))..((....))....))))..)))))...((((....)))).((((((...)))))..	.((((...))))......((((....))))............((.....)).......((((....)))).((((((...)))))..	.((((...))))......((((....))))........((((((......)).))))..((((....))))..((((((...))))))..	.((((.....(((((((.((((....))))...)))))))..((((.((((.......((((....)))))).))..)))).))))..	.(((.(((((..((((..((((....))))..........)))))))))).)))......((((....)))).((((((...))))))..	..(((((((((((((((.((((....))))...)))))))...)))..........((.....))...)))))((((((...))))))..	.((((.((((.........((((....))))............((...(((.........((((....))))))).)))..)))))))..	.((.((((....(((...((((...)))))))......))))).............((((....)))).((((((...))))))..
5Y58	(((((((..((((.......))))))))))).	((((((((((....)))....))))))).	(((((((.....((......))))))))).	((((((((............))..)))))).	............((((.........))))..	(((.((.((.(((....))).))))).......	/	/	/	/
6AAS	((((((((((((....))))))))))))	(((.((((((((....))))))).))))	(((.((((((((....))))))).)))))	(...((((((((....)))))))))....	((((((((((...(.....))))))))))	((...((((((((....)))))))))....	((((((((((.........)..)))))))))	((((((((...(.......)))))))))	(((....((((((....)))))))).....	((((((..(((....)))))...)))))
6B14	(((((((((............(((....))).(((((.....))))).((((....((((....))))..)))).)))))))))	((((((((((..((((....(((....)))...((...((((..(((....)))...))))..)).))))...))))))))))	((((((((((..(((((((..(((....))).(((.....))).)))))))).....((((....))))........)))))))))	((((((((((..................((((((((.....)))))))))...(((.((((....))))....)))))))))))))	((((((((((............((((...((((((((.....)))))))........(((....))))))))...))))))))))	((((((((((....................((((((((.....)))))))))..((((.........)))).........)))))))))	((((((((((.....(((...(((..(.((((((((((.....)))))))..............)).))))...))))))))))))	((((((((((..........(((....)))..((((...(((((.(((.....))).))))))......))))..))))))))))	((((((((((..((((........((.(.....).))...(((((.(((.....))).)))))....))))...))))))))))	((((((((((.....(((..(((....)))..)))....(((((.(((.....))).)))))...........)))))))))))
6B3K	(((((((((..(((((((.....((((((.........)))))))))))))......((((....))))........)))))))))	((((((((((..........((..((((((.........))))))..))(((((....((((....))))..)))).))))))))	((((((((((............(((....))).(((((.....))))).((((....((((....))))..)))).)))))))))	((((((((((..(((((((.....((((((.........))))))))))))((((.........)))).......))))))))))	((((((((((..(((....((..((((((.........))))))..))........((((....)))))))...))))))))))	((((((((((.....(((..((..((((((.........))))))..)).......((((....))))....))))))))))))	((((((((((..........(((....)))..((((((...((((((.........))))))..))).)))..)))))))))))	((((((((((.....(((...(((.(((((.........)))))(((((.........)))))....)))...))))))))))))	((((((((((.....(((...(((..(.(((((((((......))))))))..............)))).))))...))))))))))))	(((((((((..(((((((.....((((((.........)))))))))))))........(((............)))))))))))
6BHJ	..((((((((((((((((...)))))))))))))))))	..((((...((((((((...))))))))))))...	.....(((((((((((((...)))))))...)))))	........((((((((((...))))))))......))	((((.........))))........((....))....	((((.........))))(((.....((....))))).	/	/	/	/
6CYT	.....(((......)))	/	/	/	/	/	/	/	/	/
6D12	(((((((.....(((((.....))))).....)))))))	(((((((......)))))))((((((......)))))))	(((((((.(..((((((.....))))).)..))))))))	..((..((((..((.......))...))))..))....	(((((((......)))))))..((((.....))))....	(((((((...((((((((.....))))))...)))))))))	..((((.....))))......(((((((......)))))))	(((((((......)))))))......((.........))..	(((((((......(((((.......)).))).)))))))	(((((((......)))))))..((......))........
6DB8	((((((((((..........((((......))))((((.........))))...)))))))))))	((((((((((.......((((((((.....))))))...((......)).)))..))))))))))	((((((((((((...(((.((((......))))))))..))).............)))))))))	(((((((((((.......(((.((((.....)))))))).......(((......))))))))))))	((((((((((((.....(((((((......))))...))).......)))......)))))))))	(((((((((((((.........))).........(((((.........)))))..)))))))))	((((((((((.......((.(((((...(((.........)))..)).)))))..)))))))))	((((((((...........(((.(((.....))).((((..........))))...)))))))))	(((((((((.......(((.((((.....))))))))))))).......((.....)).)))))	(((...((........))..(((.....((.(((((.........))))).)).))).)))
6DTD	.((((.....(((((((......))).))))....))))	((((......(((((((......).))))))..))))..	((((..((((...(((.....))))))...))))..	.((((.....(((((..((....)))))))....))))	((...))..(((((((......).))))))........	((((((.....)).(((.....))).........))))..	((((((....))..(((((...)))))....))))..	..((((........(((......))..)))).......	/	/
6DU4	(((((((.....((.....))..)))))))	(((((((((((...)))).......)))))))	........((.((((.........))))))).	(((((((.....))))).........)))....	(((((((.....))).........))))....	((((((..(...((.....))..))))))))	....((.....((((.........))))..))	((((((..((...((.....))...))))))))	.((.(((.....))))).............	(((((((.....))..)).....)))....

PDB	DNA model: mfold MFE	subopt #2	subopt #3	subopt #4	subopt #5	subopt #6	subopt #7	subopt #8	subopt #9	subopt #10
6DU5	/	/	/	/	/	/	/	/	/	/
6F4G	/	/	/	/	/	/	/	/	/	/
6F4H	/	/	/	/	/	/	/	/	/	/
6FQ3	/	/	/	/	/	/	/	/	/	/
6FQL	/	/	/	/	/	/	/	/	/	/
6GBM	/	/	/	/	/	/	/	/	/	/
6HYK	/	/	/	/	/	/	/	/	/	/
6IV8	/	/	/	/	/	/	/	/	/	/
6IV9	/	/	/	/	/	/	/	/	/	/
6JXM	/	/	/	/	/	/	/	/	/	/
6KYV	/	/	/	/	/	/	/	/	/	/
6MCE	/	/	/	/	/	/	/	/	/	/
6MCF	/	/	/	/	/	/	/	/	/	/
6MJ0	/	/	/	/	/	/	/	/	/	/
6MXQ	/	/	/	/	/	/	/	/	/	/
6NOA	/	/	/	/	/	/	/	/	/	/
6PK9	/	/	/	/	/	/	/	/	/	/
6SDY	/	/	/	/	/	/	/	/	/	/
6SNJ	/	/	/	/	/	/	/	/	/	/
6SY6	/	/	/	/	/	/	/	/	/	/
6TQB	/	/	/	/	/	/	/	/	/	/
6U79	/	/	/	/	/	/	/	/	/	/
6U8D	/	/	/	/	/	/	/	/	/	/
6VZC	/	/	/	/	/	/	/	/	/	/
6W3M	/	/	/	/	/	/	/	/	/	/
6XH0	/	/	/	/	/	/	/	/	/	/
6XWJ	/	/	/	/	/	/	/	/	/	/
7K1Z	/	/	/	/	/	/	/	/	/	/

PDB	RNA model: mfold MFE	subopt #2	subopt #3	subopt #4	subopt #5	subopt #6	subopt #7	subopt #8	subopt #9	subopt #10
6DU5	((((..((.....))....))))..	((((..(((...)))....))))..	((((....((...))....))))..	..(((((.............)))))	........((((........)))).	((((.(((...))).....))))..	(((((........)).....)))..	..(((........)))..........	.((....))(((........)))..	.(((.....)))..............
6F4G	..((((((...........))))))	((.((.((((....)))).)).)).	..((((((..((...))..))))))	..((((((...(......)))))))	..((((.....((......)))))))	..(((((...((......)))))))	((((............)))).....	((((..(.....)....)))).....	(((((.(.....).)..))))....	((((..((...))....)))).....
6F4H	((((((..........))))))	((((((...(.....)))))))	((((.....((.....))))))	(((((...((.....)))))))	((((((...)).......))))	(((.....(((.....))))))	....((..(((.....))))).	.......((((.....))))..	(((((..........)..))))	((..((...)).)).......
6FQ3	((((.....))))	(((((...)))))	/	/	/	/	/	/	/	/
6FQL	.((((...)))).	(((((...)))))	/	/	/	/	/	/	/	/
6GBM	(((((((.........)))))))	((((((.((......))))))))	((((((.......)))....)))	(((((((....))).....))))	((((.(.((......)).)))))	(((.((.((......)).)))))	/	/	/	/
6HYK	(((((......((((....))))...)))))	(((((...((...((....)))...))))))	.....((((..((((....))))....))))	((((((((....))).(((....)))))))	(((((..(((......))).......)))))	..((.......((((....)))))).......	(((.(((.........))).)))........	.....(((....)))....(((......)))	((((......)).)).....(((......)))	((...(((....))).)).(((......)))
6IV8	.(((((((((....)))))))))..............................	.(((((((((....))))))))).(((..........)))...........	.....(((......((...(((((..........))))))..)))....	.....(((.....(((....(((.....)))...)))......)))....	............((((...(((((...........)))))......)))).	/	/	/	/	/
6IV9	.(((((((((....)))))))))..............................	.(((((((((....))))))))).(((..........))).........	.....(((......((...(((((..........)))))))..)))...	.....(((.....(((....(((.....)))...)))......)))...	/	/	/	/	/	/
6JXM	((((((..((((.......)))).(((((((((....(((.......))).....)))))))))......(((((.......)))))))))))))....	((((((..((((.......)))).(((((((((....(((.......))).....))))))))(((...((....)).....)))..)))))))....	..(((....((((.......)))))).(((((((((....(((.......))).....)))))))))(((..(((((.......)))))..)))..))).	((....))......(((((.((....(((((((((....(((.......))).....))))))))).....(((((.......))))))).)))))...	((....)).((((.......)))).((((((((....(((......)))......)))))))))).((..(((((..((......))...))))).)).	((....)).......(((.((....(((((((((....(((......)))......)))))))))(((..(((((.......)))))..))).)))))	((((((........(((..((...(((((((((....(((.......)))......)))))))))...))......)))........)))))))....	...(((....)))..(((.((....(((((((((....(((......)))......)))))))))(((..(((((.......)))))..))).)))))	((((((...(((........((...(((((((((....(((.......)))......))))))))...))........)))......)))))))....	((....)).((((.......)))).(((((((((....(((......)))......))))))))).((..((((....(((.......)))))))).)).
6KYV	(((((((((....)))))))))	(((((.(((....))))).)))	((((.((((....))).)))))	(((.(((((....))).)))))	(...(((((....))))))...	((...((((....))))))...	............(((....)))	(....((((....)))))....	(((((..((......)))))))	((((((((...)...)))))))
6MCE	((((((..((((.......)))))))))))	((((((((.........((...)))))))))	(((((((((........))....)))))))	((((.......)))).((.((.....))))	((((((((((....))).......)))))))	((((.......))))(((((....)).)))	...........((..(((....))).))..	((((((.((.........)).)....))))	((((...............))))).......	(((......((((.......)))))))....
6MCF	((((((((((...(((..(((((((.....)))).))))....)))...))))))))))	((((((((((((.((....(((((((....))).)))).)).)))).....)))))))	((((((((((...(((......(((.(((.......)))))))))))...)))))))))	((((((............(((((((....))).)))..(((.....))).))))))	((((((.((((......))))((((.((.((((.....))))..)).)))))))))	(((.........)))...(((((((....))).)))).((((.((...)).))))..	((((((((((...((.(((.....)))..((((.....)))))...)))))))))	(((((((((((.((..(((.....)))..)).))).((((....)))).))))))))	(((..........)))........(((....))).((((((.((....)).))))))..	.(((((.((((......)))).(((....))).)))))((((.((...)).))))..
6MJ0	..((.((((((........((((((((((....)))))))))).((((((....)))))))))))))).))...(((.((((((.....))))))....)))..	..(((((.(((((((....(((((((((....)))))))))).((((((....)))))).....(((((.......))))).......)))))))...)))))	..((((.((((........((((((((((....)))))))))).((((((....))))))....((((......))).((((((.....)))))).))))))))	..((.((((((........((((((((((....)))))))))).(((((((....)))))).))))))).))..............(((((......)))))..	..((.((((((..............(((((.......))))).(((((....)))))..)))))).))....(((.((((((.....))))))....)))..	..(..((((..........((((((((((....)))))))))).((((((....))))))....(((((.......)))))......))))..).........	(((.........))).....(((((((((....)))))))))).((((((....))))))....(((((........)))))....(((((......)))))..	..((((.((((........((((((((((....))))))))).((((((....)))))).))))((((........(((((((.....))))))))))).))))	..((((.((((........((((((((((....))))))))).((((((....))))))..(((((...................)))))).....))))))))	.((((..((((........))))..))))(((.......)))..((((((....))))))....(((((.......)))))....(((((.......)))))..
6MXQ	((((((.(((...(((.((.((.(((....)))))).)))...)))))))))	((((((.(((...((((((.......))))))..........)))))))))	((((((..((...((((((......))))))......))......))))))	((((((.(((.((((((.......)))))............)))))))))	((((((..((...((((((......))))))..)).........))))))	((((((.((((....))).(((....)))............))))))	((((((.((((....)))).......((....)).........))))))	((((((.(((...(((..((.......)).....)))...))))))))	((((((...((.......))......((....)).........))))))	((((((..((.(((((.......)))))))............))))))
6NOA	((((((.(((...(((.((.((.(((...........)))))..)))...)))))))))	((((((.(((...(((.((.((.(((......)))......))..)))...)))))))))	((((((.((((....))))).(((.......)))...................))))))	.(((((..........))))).............................	((((((.(((...(((....(((.......)))...))).........))))))))))	((((((.(((...(((...............)))..............))))))))	((((((..(((..((........)).)))......................))))))	(((......)))..	(((......)))......(((...............................))).	/
6PK9	(((((((......)))))))	((((((.....)).))))..	.(((......))).......	((((((.....)).))).).	(((((..........).))))	((((((....))....))))	((((...((.....))))))	...(((....))).......	((.(((....))).))....	((.((((......)))))).
6SDY	(((((((((((((((....))))))))))))))))))	(((...((((((((((....)))))))))...)))))	((((((((((...(((....)))))))...)))))))	(((....)))(((((((....)))))..........	.......((((((((....))))))......)).	((((((...))))).(.....).((((((...)))))	(((....)))((((((....))))).((....)).	(((.......((((((....))))))))......	....((((......))))...(((((...)))))	(((((...))))).....(((((.....)))))..
6SNJ	(((((((((((.......)))))).))))	((..((((((.......))))))..)).	((((.((.((((....)))).)).))))	(((.((((((.......))))))..))).	(((.((((((.......)))))))))))..	(((((((((.........)))))..))))	(((.....)))......(((.....)))	(((....)))......(((.....)))	(((.....)))......((((...))))	(((.....)))......(((....))).
6SY6	.(((.......((...(((....)))..))).....)))	(((.....)))....(((....)))............	..........(((...(((....))).....)))..	.(((...((...)).(((....)))........)))	.(((.((...))...(((....)))........)))	.(((((...........)))))...............	.((..........))(((....)))............	((...(((.((....)).)))..))..........	..............((..(((.......)))..)).	.(((((...........)))))((..........)).
6TQB	((((((((...))))))))	/	/	/	/	/	/	/	/	/
6U79	(((((((((((((((((.....))).)).)))))))))))	((((((((((.((.....((.....)))))))))))))	((((((((........((((......))))))))))))	((((((((((.....((..((....)).))))))))))))	((((((((((....(((......))).))....)))))))	((((((((((..........))........))))))))	((..((((......))))..))...............	.(((.....)))....((((......))))..........	(((((.....((((((......))).)))..))...)))	((((((........)).)))).................
6U8D	.((((((((((..((((((.....)))))).((((((.....))))))..(((.....)))))))))))))	.(((((((((((.....((...((((.((.((((((((.....)))))))).)).).)))))))))))))))))	((((..(((....((((((.....)))))).((((((.....)))))).....))).))))...........	.((((((((((..((((((.....))))))........((...((.......))......)).))))))))))	.((((((((((..((((((.....))))))..((((.((.........)))))).........))))))))))	.((((((((((..((((((.......((.........))..)))))....(((.....))))))))))))))	((.((......)).)).(((.((((.((.((((((((.....))))))))).)).)))).....)))......	.((((((((((.............((((..((...))..))))....(((.....)))))))))))))))))	((((((((((.....).))))).)).)).((((((((.....))))))))(((.....)))..........	((.((......)).))(((..((((.((.((((((((.....))))))))).)).)))).........)))
6VZC	((((((..((((.....))))..))))))	((((((((.(((.....))))).))))))	((((((((.(((.....)))))).)))))	((((..((((((.....)))..)))))))	((.(((..((((.....))))..))))).	((((..((((((.....))..))))))))	.(((((..((((.....))))..)).))	(((((((...((.....)))....)))))))	((((..((((((.....)..))))))))	((((((((...(.....)))...)))))
6W3M	((((..(((...((((((.....))))))...)))..))))	((((((((((.......))))))))))..............	((((..(((...((((((.....))))))...)..))))	((((..(((..........(((....)))...)))..))))	.....((((...((((((.....))))))......)))).	.((((.......((((((.....))))))...)))).....	(((((((((........)))))))))...((.........))	((((((((((........)))))))))((.........))...	.((((....))))(((((.....)))))............	(((((((((........))))...((....)).....)))))
6XH0	(((((...((((.......)))))))))	(((((...(((.(.....)))))))))	(((((...(((.((...))))))))))	((((((((........)))....)))))	((((((((.......)))......)))))	(((((((((....)))).....)))))	(((((.((.........))...)))))	..........((..(((....))).))	(((((((.......)).......)))))	(((((...........((...)))))))
6XWJ	(((((((((...)))))))))	((((.((.........))))))	.(..(((((...))))))...	/	/	/	/	/	/	/
7K1Z	(((((((((..((((((((.((.(((((.......)))..)).))...))))))))).........(((.....)))........((....))...)))))))))	(((((((((......(((.(((((............((((.((((((.....)))).)).)))).........)))))))))....((....))...)))))))))	(((((((((......(((....)))...((((....((((.((((((.....)))).)).)))).........))))).........((....))...)))))))))	(((((((((..((.(((....))))))...((((((((((.((((((.....)))).)).)))).(((.....)))...))))))...........))))))))))	(((((((((..((.(((....)))))...(((((.((((.((((((.....)))).)).)))).(((.....)))..........)))))....))))))))))	(((((((((.(((.(((....))).......(((((((((.((((((.....)))).)).)))).((((.....)))...))))))....)))...))))))))))	(((((((((............((((.........((((.((((((.....)))).)).)))).(((.....)))..))))..((....))...)))))))))	(((((((((..((((((((.((.((((((.......)))..)).))...)))))))).........(((......)))((((........))))...)))))))))	(((((((((......(((.(((((.(((....))).((((.((((((......)))).)).))))........)))))))....((....))...)))))))))	(((((((((..((((((((.((.((((((.......)))..)).))...)))))))).....((.(((.....)))..(((....)))..)))....)))))))))
