# Supplementary material for: Comparative Study of Single-stranded Oligonucleotides Secondary Structure Prediction Tools
Source: BMC Bioinformatics. 2023 Nov 8;24:422. doi: 10.1186/s12859-023-05532-5 (PMC10634105; doi:10.1186/s12859-023-05532-5)
Supplement: Supplementary file 8 — Additional file 8. Predicted secondary structure for CONTRAfold, CentroidFold, Linearfold, MC-fold, MXfold2, UFold and SPOT-RNA in the dot-bracket notation. The PDB code is reported in the first column. "/" characters indicate either structures predicted as unfolded or software failure during the computation or sequences for which the parameters were not applied. [file 12859_2023_5532_MOESM8_ESM.pdf]

Additional File 8. Predicted secondary structures for CONTRAfold, CentroidFold, Linearfold, MC-fold, MXfold2, Ufold and SPOT-RNA in the dotbracket notation. The PDB code is reported in the first column. "/" characters indicate either structures predicted as unfolded or software failure during the computation.

PDB	MXfold2	CONTRAfold	Centroidfold	Mcfold Default	Mcfold Pseudoknotted	Linearfold C	Linearfold V	Ufold	SPOT-RNA
1AC7	((((((....))))))	((((((....))))))	.(((((....))))).	(((((((..)))))))	(((((((..)))))))	((((((....))))))	((((((....))))))	((((((....))))))	((((((....))))))
1B4Y	....(((((((........)))))))....	..........((((((....))))))....	....	......(((((((((((..))))))))))))	......(((((((((((..)))))))))))	..........((((((....))))))....	..........((((((....))))))....	..........(((((((..)))))))....	..........(((((((..)))))))....
1BJH	((((...))))	((((...))))	....	((((...))))	((((...))))	....	....	((((...))))	((((...))))
1ECU	((((((((...))))))))	(((((((((.)))))))))	((((((((...))))))))	((((((((...))))))))	((((((((...))))))))	(((((((.....)))))))	(((((((.....)))))))	((((((((...))))))))	(((((((((.)))))))))
1EN1	((((......))))....	(.(((.....))))....	....	(((((((..)))).))).	(((((((..)))).))).	((((......))))....	((((......))))....	((((......))))....	(.(((.....))))....
1EZN	((((((((((....)))((((....)))..))))))))	(((((((.((....))((((....)))).))))))))	.((((((.....................)))))).	(((((((((((..))))((((..))))..)))))))	(((((((((((..))))((((..))))..)))))))	(((((((........(((((....))))))))))))	(((((((........(((((....))))))))))))	(((((((..((....<(((...))).))>)))))))	(((((((.......((((.....))))..))))))))
1JVE	((((((((((((...))))))))))))	((((((((((((...))))))))))))	((((((((((((...))))))))))))	((((((((((((...))))))))))))	((((((((((((...))))))))))))	((((((((((((...))))))))))))	((((((((((((...))))))))))))	((((((((((((...))))))))))))	((((((((((((...))))))))))))
1LA8	(((((...)))))	(((((...)))))	.((((...)))).	(((((...)))))	(((((...)))))	(((((...)))))	(((((...)))))	(((((...)))))	(((((...)))))
1NGO	(((((((((((.....)))))))))))	(((((((((((.....)))))))))))	((((((((((.(....)))))))))))	((((((((((.((..))))))))))))	((((((((((.((..))))))))))))	(((((((((((.....)))))))))))	(((((((((((.....)))))))))))	(((((((((((.....)))))))))))	(((((((((((.....)))))))))))
1NGU	((((..(((((.....)))))..))))	((((..(((((.....)))))..))))	((((..(((((.....)))))..))))	((((((((((((...))))))))))))	((((((((((((...))))))))))))	((((..(((((.....)))))..))))	((((..(((((.....)))))..))))	((((..(((((.....)))))..))))	(((((((((((.....)))))))))))
1OSB	(((((((....)))))))).......	(((((((....)))))))).......	.((((((....))))))........	((((((((..))))))))).......	((((((((..))))))))).......	(((((((....)))))))).......	(((((((....)))))))).......	(((((((....)))))))).......	((((((((..))))))))).......
1P0U	(((((...)))))	((((((.))))))	(((((...)))))	(((((...)))))	(((((...)))))	....	....	(((((...)))))	(((((...)))))
1PQT	((...))	....	....			....	....	....	((...))
1SNJ	((((((((((....)))((((....)))..))))))))	(((((((.(((.(((()))).....))).)))))))	.((((((.((....))............))))))	(((((((((((..))))((((..))))..)))))))	(((((((((((..))))((((..)))))))))))	(((((((.(((.............))).)))))))	(((((((.(((.............))).)))))))	(((((((.(((....(.<.....)>))))))))))	(((((((.........((((..))))..))))))
1UUT	((((((...))))))	((((((...))))))	((((((...))))))	((((((...))))))	((((((...))))))	((((((...))))))	((((((...))))))	((((((...))))))	((((((...))))))
1XUE	((............))	....	....	(((((((...)))))))	(((((((...)))))))	....	....	...(..)..........	((..(((...)))..))
1YTB	((((((((((((.....))))))))))))	((((((((((((.....))))))))))))	((((((((((((.....))))))))))))	((((((((((((.....))))))))))))	((((((((((((.....))))))))))))	((((((((((((.....))))))))))))	((((((((((((.....))))))))))))	((((((((((((.....))))))))))))	((((((((((((.....))))))))))))
1ZHU	(........)	....	....	((((..))))	((((..))))	....	....	.(......).	....
1ZM5	(((((((....)))))))).......	(((((((....)))))))).......	.((((((....))))))........	((((((((..))))))))).......	((((((((..))))))))).......	(((((((....)))))))).......	(((((((....)))))))).......	(((((((....)))))))).......	((((((((..))))))))).......
2A0I	(.(....).)	....	....			....	....	(...(<.))>	....
2A6O	((((((((....)))).)))).	((((((((....)))).)))).	((((((((....)))).)))).	(((((((((..))))).)))).	(((((((((..))))).)))).	((((((((....)))).)))).	((((((((....)))).)))).	(((((((((..))))).)))).	(((((((((..))))).)))).
2CDM	((((((....)))))).........	((((((....)))))).........	.(((((....))))).........	(((((((..)))))))(((...)))	(((((((..)))))))(((...)))	((((((....)))))).........	((((((....)))))).........	((((((....)))))).........	(((((((..))))))).........
2EXF	((((......))))	((((......))))	...(.......)..	(.((((...)))))	(.((((...)))))	....	....	((((......))))	(.(((.....))))
2JZW	((((......))))	((((......))))	...(.......)..	(.((((...)))))	(.((((...)))))	....	....	((((......))))	(.(((.....))))
2K71	((....))	((....))	((....))	(((..)))	(((..)))	....	....	((....))	(((..)))

PDB	MXfold2	CONTRAfold	Centroidfold	Mcfold Default	Mcfold Pseudoknotted	Linearfold C	Linearfold V	Ufold	SPOT-RNA
2L5K	(((...((((...))))...)))	(((...((((...))))...)))	(((...((((...))))...)))	((((((((((...))))))))))	((((((((((...))))))))))	....	....	(((...((((...))))...)))	(((..(((((...)))))..)))
2LO5	(((......)))	(((......)))	(((......)))	((((...).)))	((((...).)))	....	....	(((......)))	((((...).)))
2LO8	((......))	....	....	(((...).))	(((...).))	....	....	((......))	.(......).
2M8Y	((((((...))))))	((((((...))))))	.(((((...))))).	((((((...))))))	((((((...))))))	((((((...))))))	((((((...))))))	((((((...))))))	((((((...))))))
2N8A	(((((((((....))))))))).(((((((((....)))))))))	(((((((((....))))))))).((((((((....)))))))))	(((((((((....)))))))))..((((((((....)))))))).	((((((((((..)))))))))).((((((((((..))))))))))	((((((((((..)))))))))).((((((((((..))))))))))	(((((((((....))))))))).(((((((((....)))))))))	(((((((((....))))))))).(((((((((....)))))))))	(((((((((....))))))))).((((((((((..))))))))))	((((((((((..)))))))))).((((((((((..))))))))))
2VHG	((((((((....)))).))))...	((((((((....)))).))))...	((((((((....)))).))))...	(((((((((..))))).))))...	(((((((((..))))).))))...	((((((((....)))).))))...	((((((((....)))).))))...	(((((((((..))))).))))...	(((((((((..))))).))))...
2VIC	....(((((((((...))))).))))	....(((((((((...))))).))))	.....(((((((.....)))).))).	....(((((((((...))))).))))	....(((((((((...))))).))))	....((((((((.....)))).))))	....((((((((.....)))).))))	....(((((((((...))))).))))	....(((((((((...))))).))))
2VJU	...(((((((((....)))).))))).........	...(((((((((....)))).)))))..........	....((((((((....)))).))))..........	((((((((((((((..))))).)))))))))......	((((((((((((..))))).))))))))......	...(((((((((....)))).))))).........	...(((((((((....)))).)))))..........	.(.((((((((((..))))).))))).).......	..((((((((((((..))))).))))))........
3C46	......((((((...))))))	......((((((...))))))	.......(((((...))))).	......((((((...))))))	......((((((...))))))	......((((((...))))))	......((((((...))))))	......((((((...))))))	......((((((...))))))
3DSD	.(((((((....))))))))....	.(((((((....))))))))....	.(((((((....))))))))....	((((((((....))))))))...	((((((((....))))))))....	.(((((((....))))))))....	.(((((((....))))))))....	.(((((((....))))))))....	.(((((((....))))))))....
3HXO	((((.....))))....(.((((((((.....))).)))))	.(((((..(((((..((())))...)))))......)))))	.(((((......(....)......((.....))..)))))	.((((((((((((...)))))))((((...)))).)))))	((((((((((..))))))[[[[[))))))..]].]]]].	...................(((((((.....))).)))).	...................(((((((.....))).)))).	.(((((......(...)((((..)).))......)))))	.(((((....(((...)))....(((.....))).)))))
3Q0A	.......(((((...)))))	.......(((((...)))))	.......(((((...)))))	.......(((((...)))))	.......(((((...)))))	.......(((((...)))))	.......(((((...)))))	.......(((((...)))))	.......(((((...)))))
3Q23	......((((((...))))))	......((((((...))))))	.......(((((...))))).	......((((((...))))))	......((((((...))))))	......((((((...))))))	......((((((...))))))	......((((((...))))))	......((((((...))))))
3Q24	.......((((((...))))))	.......((((((...))))))	........(((((...))))).	.......((((((...))))))	.......((((((...))))))	.......((((((...))))))	.......((((((...))))))	.......((((((...))))))	.......((((((...))))))
3THW	(((((((((((((((((((((((...))))))))).....))))))))))))))	(((((((((((((((((((((((...)))))))))....))))))))))))))	(((((((((((((((((((((((...)))))))))....))))))))))))))	(((((((((((((((((((((((...))))))))))....))))))))))))))	((((((((((((((((((((((...))))))))))....)))))))))))))	(((((((((((((((((((((((...)))))))))....)))))))))))))	(((((((((((((((((((((((...)))))))))....)))))))))))))	((((((((((((((((((((((..)))))))))....))))))))))))))	(((((((((((((((((((((((...)))))))))....)))))))))))))
3WPD	(((....)))	(((....)))	.(......).	((((..))))	((((..))))	....	....	(((....)))	((((..))))
3WPG	(((....))).	((......)).	....	((((..)))).	((((..)))).	....	....	(((....))).	(((....))).
3WPH	(((....)))..	((......))..	....	((((..))))..	((((..))))..	....	....	(((....)))..	((.....).)..
3ZH2	(((((((.((......)).)).))))))	(((((.................)))))	(((((.................)))))	(((((((((((....))))))).)))))	(((((((((((....))))))).)))))	(((((.................)))))	(((((.................)))))	(((((...(............))))))	(((((...(((....)))....)))))

PDB	MXfold2	CONTRAfold	Centroidfold	Mcfold Default	Mcfold Pseudoknotted	Linearfold C	Linearfold V	Ufold	SPOT-RNA
4ER8	......(((((..((((....))))..)))))	......(((((..((((....))))..)))))	.......((((..((((....))))..)))).	......(((((((((((..))))))))))))	......(((((((((((..))))))))))))	......(((((..((((....))))..)))))	......(((((..((((....))))..)))))	......(((((..(((((..)))))..)))))	......(((((.((((((..)))))).)))))
4F41	(((((((((((((....)))))))))))))	((((((((((((((....))))))))))))))	((((((((((((....)))))))))))))	(((((((((((((..)))))))))))))	((((((((((((((..))))))))))))))	((((((((((((....)))))))))))))	((((((((((((....)))))))))))))	((((((((((((((..))))))))))))))	(((((((((((((..)))))))))))))))
4F43	(((((((((((((....)))))))))))))	((((((((((((((..))))))))))))))	((((((((((((....)))))))))))))	(((((((((((((..)))))))))))))	((((((((((((((..))))))))))))))	((((((((((((....)))))))))))))	((((((((((((....)))))))))))))	((((((((((((((..))))))))))))))	(((((((((((((..)))))))))))))))
4FF1	.......(((((...)))))	.......(((((...)))))	.......(((((...)))))	(((....)))((((..))))	(((....)))((((..))))	.......(((((...)))))	.......(((((...)))))	.......(((((...)))))	...(..)(((((...)))))
4HT4	.(((((((...)))))))..........	.(((((((...)))))))..........	.(((((((...)))))))..........	.((((((((((.((..)))))))))))))	.((((((((((.((..))))))))))))	.(((((((...)))))))..........	.(((((((...)))))))..........	((((((((...)))))........))).	.(((((((...)))))))..........
4KB0	((((((....))))))..	((((((....))))))..	((((((....))))))..	((((((....))))))..	((((((....))))))..	((((((....))))))..	((((((....))))))..	((((((....))))))..	((((((....))))))..
4KB1	((((((....))))))..	((((((....))))))..	((((((....))))))..	((((((....))))))..	((((((....))))))..	((((((....))))))..	((((((....))))))..	((((((....))))))..	((((((....))))))..
5F55	(..(....)....)	....	....	(((((..))..)))	(((((..))..)))	....	....	....	....
5GWL	(......)	....	....			....	....	....	....
5GWQ	(......)	....	....			....	....	....	....
5HRU	((((.........(((((.....)))))))))	.........(((.(((((.....))))).)))	.............(((((.....)))))....	(((..))).((((((((((...))))))))))	((((.[[[[)))(((((((...)))))))]]]]	.........(((.(((((.....))))).)))	.........(((.(((((.....))))).)))	(((((.(.....<.{.((......>)))})))))	((((........<<((((...>>)))).))))
5HTO	(((((.........(((((.....))))))))))	((.........((.(((((.....))))).))))	.............(((((.....)))))....	((((..))))((((((((((...)))))))))).	((((..))))((((((((((...)))))))))))).	..............(((((.....)))).....	..............(((((.....)))).....	(((((.........(((((.....))))))))))	(((((........((<<.....))..>>.)))))
5N2Q	(((((((....))))))))........	(((((((....))))))))........	.((((((....)))))).........	((((((((..))))))))........	((((((((..))))))))........	(((((((....))))))))........	(((((((....))))))))........	(((((((....))))))))........	((((((((..))))))))........
5OND	((....)).	....	....	(((..))).	(((..))).	....	....	..(.....)	.(....)..
6FK4	..(((((...))))).	..(((((...))))).	..(((((...))))).	.((((((...))))))	.((((((...))))))	..(((((...))))).	..(((((...))))).	..(..((...))..).	.((((((...))))))
6FK5	(((((...))))).	(((((...))))).	(((((...))))).	(((((...))))).	(((((...))))).	(((((...))))).	(((((...))))).	(((((...))))).	(((((...))))).
6FKE	.((((...))))	.((((...))))	..(((...))).	.((((...))))	.((((...))))	....	....	.((((...))))	.((((...))))
6IY5	(........)	....	....			....	....	....	....
6J37	(......)	....	....			....	....	...(.)..	....
6M0B	(......)	....	....	(((..)))	(((..)))	....	....	(......)	....
6M0C	(......)	....	....	(((..)))	(((..)))	....	....	....	....
6SEI	.(((.(((((((((...)))))))))..)).)))	.(((.(((((((((...)))))))))..)).)))	.....((((((((...))))))))..)).....	.((((((((((((...)))))))))..))))))	.((((((((((((...)))))))..))))))	.......(((((((...)))))))........	.......(((((((...)))))))........	.(((.(((((((((...)))))))))..)).)))	.((((((((((((...))))))))..))))))
6U82	(((((((((((((((...))))))))))))...))))))	((((((((((((((((...)))))))))))...))))))	((((((((((((((((...))))))))))))...))))))	((((((((((((((((...))))))))))))...))))))	((((((((((((((((...))))))))))...))))))	((((((((((((((((...)))))))))))...))))))	((((((((((((((((...)))))))))))...))))))	((((((((((((((((...))))))))))...))))))	((((((((((((((((...))))))))))))...))))))

PDB	MXfold2	CONTRAfold	Centroidfold	Mcfold Default	Mcfold Pseudoknotted	Linearfold C	Linearfold V	Ufold	SPOT-RNA
17RA	(((((.(((....))))))))	((((((.((....))))))))	(((((..((....)).)))))	(((((.(((....))))))))	(((((.(((....))))))))	(((((.(((....))))))))	(((((.(((....))))))))	(((((.(((....))))))))	(((((.(((....))))))))
1A1T	((((((((....))))))))	((((((((....))))))))	((((((((....))))))))	((((((((....))))))))	((((((((....))))))))	((((((((....))))))))	((((((((....))))))))	((((((((....))))))))	((((((((....))))))))
1A4T	(((((.....)))))	(((((.....)))))	(((((.....)))))	((((((...))))))	((((((...))))))	(((((.....)))))	(((((.....)))))	((((.......))))	(((((.....)))))
1A51	((((.((....((((((((...))))))))....)).))))	((((.((....((((((((...))))))))....)).))))	((((.......((((((((...))))))))......))))	((((((((((((((((((...))))))))))))))))))))	((((((((((((((((((...))))))))))))))))))	((((.((....((((((((...))))))))....)).))))	((((.((....((((((((...))))))))....)).))))	((((.((..(.((((((((...))))))).)..)).))))	((((((((((.((((((((...)))))))).))))))))))
1A60	(((((.......))))).(((((....)))))............	(((((.......))))).......((((((......))))))...	(((((.......)))))..((((....))))............	(((((((...)))))))....((((((((((..)))))))))).)	(((((((...)))))))(((.[[[[[[[()))...]]]]]]].	(((((.......)))))......((((((......))))))...	(((((.......)))))......((((((......))))))...	(((((.......)))))<<<...((((((>>>...))))))...	(((((.......)))))......((((((......))))))...
1A9N	(((((............))))).	((((((((((())))))...))))).	(((((............))))).	(((((((((..))))))...))).	(((((((((..))))))...))).	(((((............))))).	(((((............))))).	(((((.((....)).))...))).	((((((((....)))...))))).
1AFX	((((....))))	((((....))))	((((....))))	(((((..)))))	(((((..)))))	((((....))))	((((....))))	((((....))))	(((((..)))))
1ANR	((((((...((((......))))))))))	((((((...((((......))))))))))	((((((...((((......))))))))))	((((((...(((((...).))))))))))	((((((...(((((...).)))))))))	((((((...((((......))))))))))	((((((...((((......))))))))))	((((((...((((......))))))))))	((((((...(((((...).))))))))))
1ATO	((((((.......))))))	((((((.......))))))	((((((.......))))))	((((((((...))))))))	((((((((...))))))))	((((((.......))))))	((((((.......))))))	((((((.......))))))	((((((.......))))))
1ATV	((((((....)))))).	((((((....)))))).	((((((....)))))).	((((((....)))))).	((((((....)))))).	((((((....)))))).	((((((....)))))).	((((((....)))))).	((((((....)))))).
1ATW	(((((....))))).	(((((....))))).	(((((....))))).	((((((..)))))).	((((((..)))))).	(((((....))))).	(((((....))))).	((((((..)))))).	((((((..)))))).
1AUD	(((((.((((....)))).......)))))	(((((.((((....)))).......)))))	.((((.((((....)))).))))).......	((((((((((..))))))......)))))	((((((((((..))))))......)))))	(((((.((((....)))).......)))))	(((((.((((....)))).......)))))	((((((((((..))))).)....)))))	(((((.(((((..))))).......)))))
1B36	(((((.......((((....)))).........)))))	(((((.(..((.((((....))))))......))))))	(((((.......((((....))))((.....)))))))	((((((((((.(((((..)))))))))))...)))))	((((((((((.(((((..)))))))))))...)))))	(((((.......((((....)))).........)))))	(((((.......((((....)))).........)))))	(((((.(..(.((((((..)))))).).)..)..)))))	(((((.......(((((..))))).........)))))
1BGZ	(((((..(((....))).)))))	((((((((.....)))..)))))	(((((...((....))..)))))	((((((.((((..))))))))))	((((((.((((..))))))))))	(((((............)))))	(((((............)))))	(((((..((((..)))).)))))	(((((..((((..)))).)))))
1BVJ	(((((.(((......))))))))	(((((.(((......))))))))	(((((.((........)))))))	(((((.(((((..))))))))))	(((((.(((((..))))))))))	(((((.(((......))))))))	(((((.(((......))))))))	(((((.(((......))))))))	(((((..((......)).)))))
1BZ2	(((((.......)))))	(((((.......)))))	.((((.......)))).	(((((.((..)))))))	(((((.((..)))))))	(((((.......)))))	(((((.......)))))	(((((.(...).)))))	((((((.....))))))
1BZ3	(((((.......)))))	(((((.......)))))	.((((.......)))).	(((((.((..)))))))	(((((.((..)))))))	(((((.......)))))	(((((.......)))))	(((((.(...).)))))	((((((.....))))))
1CQ5	(((((((....(((....(((....)))....))).)))))))	(((((((....(((....(((....)))....))).)))))))	((((((..(........).((....))..........))))))	((((((((((((((((..)))))))..)))))).(((..))))))	((((((((((((((((..))))))..)))))).(((..))))))	(((((((....(((....(((....))).....))).)))))))	(((((((....(((....(((....))).....))).)))))))	(((((((....(((....(((....)))....))).)))))))	(((((((...(((((..(((((..))))).).)))))))))))
1CQL	(((((((....(((....(((....)))....))).)))))))	(((((((....(((....(((....)))....))).)))))))	((((((..(........).((....))..........))))))	((((((((((((((((..)))))))..)))))).(((..))))))	((((((((((((((((..))))))..)))))).(((..))))))	(((((((....(((....(((....))).....))).)))))))	(((((((....(((....(((....))).....))).)))))))	(((((((....(((....(((....)))....))).)))))))	(((((((...(((((..(((((..))))).).)))))))))))
1D0U	((((((.((....))))))))	((((((.((....))))))))	((((((.((....))))))))	((((((.((....))))))))	((((((.((....))))))))	((((((.((....))))))))	((((((.((....))))))))	((((((.((....))))))))	((((((.(((..)))))))))

PDB	MXfold2	CONTRAfold	Centroidfold	Mcfold Default	Mcfold Pseudoknotted	Linearfold C	Linearfold V	Ufold	SPOT-RNA
1DRZ	(((((((.........(((........)))))))))).......((((..........))))..........	(((((((......(((((......))))).))))))..((((.....)))).(....).............	.((((((......(((((......))))).))))))..(....).((((((.((....))..).)))))...	(((((((.(((..)))((((((..))))))))))))((((((.....))))))((((((((...))))))).	.((((((...[[[[[[(((((((..))))))))))))((((....)))((((((((..)))).))).]]]]]]	(((((((......(((((......))))).)))))))................................	(((((((......(((((......))))).)))))))................................	..........(((((((((.........()..))))).((((.....))))................))))	(((((((..{<<<<<<((......))....)))))))).........}.(.(...........)..)>>>>>>
1E4P	((((...(((....)))...))))	((((...(((....)))...))))	((((...(((....)))...))))	(((((((((((..)))))))))))	(((((((((((..))))))))))))	((((...(((....)))...))))	((((...(((....)))...))))	((((...(((....)))...))))	((((...((((..))))...))))
1EBR	((((..((((((....)))).))...))))	((((.((((((....)))).)).).).))))	((((..((((((....)))).))...))))	(((((((((((((..))))).).))))))))	(((((((((((((..))))).).)))))))	((((..((((((....)))).))...))))	((((..((((((....)))).))...))))	((((.((((((((..))))).)).).))))	((((((((((((((..))))).)).))))))
1EBS	((((..((((((....))))))...))))	((((.(((((((....)))))).).))))	((((..((((((....))))))...))))	(((((((((((((..))))))).))))))	((((((((((((((..))))))).))))))	((((..((((((....))))))...))))	((((..((((((....))))))...))))	((((.((((((((..)))))))).).))))	((((..(((((((..)))))))...))))
1EIY	(((((((..((((........))))..((((.......)))).......(((((.......)))))))))))))....	((((((((.((((........))))((((((.......))))).)....((((.......)))))))))))....	(((((((..((((........)))).((((.(....))))))......((((.......)))).)))))))....	((((((((.((((((((((((..))))))))))))((((((((((((..)))))))))))).)))))))))....	((((((((((((((((((((..))))))))))))((((((((.[[[[.))))))))))))))))))..]]]]]..	(((((((..((((........)))).((((........)))))......(((((........)))))))))))....	(((((((..((((........)))).((((........)))))......(((((.......)))))))))))....	(((((((..((((........)))).(((((.......))))).....((((.......))))))))))))....	(((((((<.((((><..{[..))))((((((.....)))))))...>((((((}].)..))))))))))))....
1EKZ	(((((((((((((....)))))))))))))	(((((((((((((....)))))))))))))	((((((((((((....)))))))))))))	((((((((((((((..)))))))))))))	((((((((((((((..))))))))))))))	(((((((((((((....))))))))))))))	(((((((((((((....))))))))))))))	((((((((((((((..))))))))))))))	(((((((((((((((..))))))))))))))
1ESH	(((((...)))))	(((((...)))))	(((((...)))))	(((((...)))))	(((((...)))))	(((((...)))))	(((((...)))))	(((((...)))))	(((((...)))))
1ESY	((((((....))).))))	((((((....))).))))	((((((....))).))))	((((((....))).))))	((((((....))).))))	((((((....))).))))	((((((....))).))))	((((((....))).))))	((((((....))).))))
1ETF	((((((..((((((....)))).))...))))))	((((((.(((((((....)))).)).).)))))	((((((..((((((....)))).))...)))))	(((((((((((((((..))))).).)))))))))	(((((((((((((((..)))).).).)))))))))	((((((..((((((....)))).))...))))))	((((((..((((((....)))).))...))))))	((((((.(((((((....)))).)).).))))))	((((((.((((((((..))))).)))..)))))
1EUQ	((((((..(((.........))).((((((.......)))))...((((((.......)))))))))))........	((((((((((...))))).....(.((((((.......)))))..)((((((.......)))))))))))).....	(((((....................(((((.......)))))...((((((.......)))))).))))).....	(((((((((((....)))))))))))((((((..)))))((((((.(.((((((((.....)))))))))))))))	((((((((((...))))))[[[[(((((((((...))))))))).((((((...)..)))))))))..]]]]	(((((...(((.........))).((((((.......)))))..((((((.......)))))))))))).....	(((((...(((.........))).((((((.......)))))..((((((.......)))))))))))).....	(((((((.(((.........))).((((((.......)))))....((((.......)))))))))))).....	((((((<.((((>......))))((((((.......))))))..((((((...)..)))))))))))).....
1EXY	((((((((((((....))))..)))..))))).	((((((((((((....))))..)))..)))))).	((((((((((((....))))..)))..)))))).	(((((((((((((..)))))..)))..)))))).	(((((((((((((..)))))..)))..))))).	((((((((((((....))))..)))..))))).	((((((((((((....))))..)))..))))).	((((((((((((....))))..))))..))))).	(((((((((((((..)))))..))))..)))).
1F7F	(((((.((((((....)))))))))))	(((((.((((((....)))))))))))	(((((.((((((....)))))))))))	(((((.(((((((..))))))))))))	(((((.(((((((..))))))))))))	(((((.((((((....)))))))))))	(((((.((((((....)))))))))))	(((((.(((((((..)))))))))))))	(((((.(((((((..))))))))))))
1F84	((((.....(((......)))....))))	((((.....(((......)))....))))	(((................(....))))	((((((.(((((((..)))))))))))))	((((((.(((((((..)))))))))))))	((((.....(((......)))....))))	((((.....(((......)))....))))	((((..().(((......)))....))))	(((((....(((......)))...)))))
1F85	(((((....)))))	(((((....)))))	(((((....)))))	((((((..))))))	((((((..))))))	(((((....)))))	(((((....)))))	(((((....)))))	(((((....)))))
1F9L	((((..(((....)))..))))	((((..(((....)))..))))	((((..(((....)))..))))	((((((((((..))))))))))	((((((((((..)))))))))))	((((..(((....)))..))))	((((..(((....)))..))))	((((..(((....)))..))))	((((..((((..))))..))))

PDB	MXfold2	CONTRAfold	Centroidfold	Mcfold Default	Mcfold Pseudoknotted	Linearfold C	Linearfold V	Ufold	SPOT-RNA
1FFY	(((((((..((((.........)))).(((((.......))))).....(((((.......))))))))))))..	(((((((..((((.........)))).(((((.(....))))))......(((((.......))))))))))))..	(((((((.......................(....)..........(((((.......)))))))))))..	(((((((.((((((((((((..))).))))((((..)))).)))))).((((((...)..))))))))))))..	(((((...)))))((((((((((((.((((((((..))))[[[.[[[[)))))))))))))))))..]]]]]]	(((((((.......(((((...((((.(((((((....)))).........))))))).....))))))))))))..	(((((((.......(((((...((((.((((((....)))).........))))))).....))))))))))))..	(((((((..((((.........)))).(((((.......)))))......(((((.......))))))))))))..	(((((((..(((((<......)))))((((((.....)))))))...>((((((...)..)))))))))))..
1FHK	((((......))))	((((......))))	((((......))))	((((((..))))))	((((((..))))))	....	....	((((......))))	((((......))))
1FJE	((((.............))))	((((.............))))	((((.............))))	(((((((.((..))).))))))	(((((((.((..))).))))))	((((.............))))	((((.............))))	((((.(..........).))))	(((((...(....)...)))))
1FQZ	(((.....(((......)))....)))	(((.....(((......)))....)))	..................(....)..	(((((.(((((((..))))))))))))	(((((.(((((((..))))))))))))	(((.....(((......)))....)))	(((.....(((......)))....)))	(((.....((((...).)))....)))	((((....(((......)))...))))
1FYO	((((...((((....))))....))))	((((.(.((((....))))..).))))	((((.(.((((....))))..).))))	(((((((((((..))))).)))))))	(((((((((((..))))).))))))	((((.(.((((....))))..).))))	((((.(.((((....))))..).))))	((((.(.(((((..)))))..).))))	((((((.(((((..)))))..))))))
1G70	((((((..(((((....))).))...))))))	((((((.((((((....))).)).).)))))))	((((((..(((((....))).))...))))))	(((((((((((((((..)))).).))))))))))	((((((((((((((..)))).).)))))))))	((((((..(((((....))).))...))))))	((((((..(((((....))).))...))))))	((((((.(((((((..)))).)).).))))))	((((((((((((((..)))).)))).))))))
1GRZ	(((((.(...(((((((....(((.(.....((((.((((((((.((((((((....))))))))).(((((....))))).)))).....))))))))).....).)))..)).)))))....((((...((((((((....))))))))..)))).((((....))))....).))))).(((((((.....))))))).................((.(((((....)))))..))........	..........(((((((...(((((((....(((.(((((((((.((((((((....))))))))).(((((....))))).)))).....))))))))...)))))))..)).)))))(...((((.(.((((((((....)))))))).)))))).........(((((..((((.....(((((((.....)))))))....))))))))...((.(((((....)))))..))........	..............................(((..(((.......((((((((....)))))))...((((....))))...........))).)))........................(((((((((....))))))))...............................((((((.....)))))))...................(((((....)))))..........			(((((.((...((((.....(((((((....(((.((((((((((.(((((((((....)))))))))).(((((....))))).))))))......))))))))...)))))))......)))((...((((...(((((((((.....))))))))..))))...))...........)).))))).(((((((.....))))))).................((.(((((....)))))..))........	....(((((((((....)))))))).............	.<<........((((>(...(((((((....((((.((((((((.((((((((((....)))))))))...(((<<..)))>>.))))......)))))))...)))))))..)..))))((...((((.(.((((((((....))))))))).))))))...))...(.(.<((..(>....>..(((((((.....))))))).......)))..))......(((((....)))))...........	(((............)).................................((((((((((..))))))))))).............................(......)............((((...(((((((((..)))))))))..)))).........((((.....)))).))..(((((((.....))))))..................(((((((..))))))...........
1GTR	((((((..(((.........))).((((((.......))))))......(((((.......))))))))))))))........	((((((..(((.........))).(((((.......)))))......(((((.......)))))))))))).....	((((((...(...........)..(((((.......)))))......((((.......)))).)))))).....	((((((((((...)))((((((((.((((((..))))))))))))))((((((...)..)))))))))))....	((((((((((...))))))[[[[((((((((((((((((((.....))))))))))))))))).)))))..]]]]	((((((..(((.........))).(((((.......)))))......(((((.......)))))))))))).....	((((((..(((.........))).(((((.......)))))......(((((.......)))))))))))).....	((((((..(((.........))).((((((.......)))))......((((((.......))))))))))))).....	((((((<.((((><.....))))(((((((.......)))))))...>((((((...).))))))))))).....
1HLX	((((((((....))))))))	((((((((....))))))))	((((((((....))))))))	(((((((((..)))))))))	(((((((((..)))))))))	((((((((....))))))))	((((((((....))))))))	(((((((((..)))))))))	(((((((((..)))))))))
1HS1	(((......))).	(((......))).	(((......))).	((((....)))).	((((....)))).	....	....	(((......))).	((((....)))).
1HS2	(((......))).	(((......))).	(((......))).	(((((..))))).	(((((..))))).	....	....	(((......))).	(((......))).
1HS3	(((......))).	(((......))).	(((......))).	((((....)))).	((((....)))).	....	....	(((......))).	((((....)))).
1HS4	(((......))).	(((......))).	(((......))).	(((((..))))).	(((((..))))).	....	....	(((.(..).))).	(((......))).
1HS8	(((......))).	(((......))).	(((......))).	((((....)))).	((((....)))).	....	....	(((......))).	(((......))).
1HVU	...(((((........))))).........	..........((((((........))))))	.....(((........)))...........	(((....)))(((((((((..)))))))))	(((....)))(((((((((..)))))))))	..........((((((........))))))	..........((((((........))))))	....<<<...((((((.>>>....))))))	....<<<<..((((((>>>>....))))))

PDB	MXfold2	CONTRAfold	Centroidfold	Mcfold Default	Mcfold Pseudoknotted	Linearfold C	Linearfold V	Ufold	SPOT-RNA
1HWQ	(((((...(((((....)))))...)))))	(((((...(((((....)))))...)))))	(((((...(((((....)))))...))))))	(((((((((((((..))))))))))))))	(((((((((((((..)))))))))))))))	(((((...(((((....)))))...)))))	(((((...(((((....)))))...)))))	(((((...(((((....)))))...)))))	(((((...(((((....)))))...)))))
1I3X	(((((((.....)))))))	(((((((.....)))))))	(((((((.....)))))))	((((((((...))))))))	((((((((...))))))))	(((((((.....)))))))	(((((((.....)))))))	(((((((.....)))))))	(((((((.....)))))))
1I46	(((((...)))))	(((((...)))))	(((((...)))))	(((((...)))))	(((((...)))))	(((((...)))))	(((((...)))))	(((((...)))))	(((((...)))))
1I4B	(((((...)))))	(((((...)))))	(((((...)))))	(((((...)))))	(((((...)))))	(((((...)))))	(((((...)))))	((((((.))))))	((((((.))))))
1IDV	(((....)))	((((()))))	(((....)))	((((..))))	((((..))))	(((....)))	(((....)))	((((..))))	((((..))))
1IK1	(((((...))))).	(((((...))))).	(((((...))))).	(((((...))))).	(((((...))))).	(((((...))))).	(((((...))))).	(((((...))))).	(((((...))))).
1IKD	(((((((....)))))))....	(((((((....)))))))....	(((((((....)))))))....	((((((((..))))))))....	((((((((..))))))))....	(((((((....)))))))....	(((((((....)))))))....	((((((((..))))))))....	(((((((....)))))))....
1JBT	((((((...((.(....)..)).))))))	((((((((.......))......))))))	.(((((......(....).....))))).	(((((((((.((((..)))))))))))))	(((((((((.((((..)))))))))))))	((((((.................))))))	((((((.................))))))	((((((.................))))))	((((((((.......))......))))))
1JO7	((((((((...((((....))))..))))))))	((((((((.(.((((....))))).))))))))	.((((((...((((....))))..)))))).	(((((((((.(((((..))))))))))))))	(((((((((.(((((..))))))))))))))	((((((((.(.((((....))))).))))))))	((((((((.(.((((....))))).))))))))	((((((.((.(((((..)))))).)))))))	((((((((.(.(((((..)))))).))))))))
1JOX	(((((.((......)))))))	(((((.((......)))))))	(((((.((......)))))))	(((((.((((..)))))))))	(((((.((((..)))))))))	(((((.((......)))))))	(((((.((......)))))))	(((((.((......)))))))	(((((.((......)))))))
1JTJ	(((((((.........)))))))	(((((((..(....).)))))))	(((((((.........)))))))	((((((((.((..))))))))))	((((((((.((..))))))))))	(((((((.........)))))))	(((((((.........)))))))	(((((((..((..)).)))))))	(((((((..((..)).)))))))
1JTW	.(((((....))))).	.(((((....))))).	.(((((....))))).	(((((((..)))))))	(((((((..)))))))	.(((((....))))).	.(((((....))))).	.(((((....))))).	.(((((....))))).
1JUR	((((((((....)))))..)))	((((((((....)))))..)))	((((((((....)))))..)))	(((((((((..))))))..)))	(((((((((..))))))..)))	((((((((....)))))..)))	((((((((....)))))..)))	((((((((....)))))..)))	(((((((((..))))))..)))
1JWC	((((((....))))))	((((((....))))))	((((((....))))))	((((((....))))))	((((((....))))))	((((((....))))))	((((((....))))))	((((((....))))))	((((((....))))))
1JZC	(((((...)))))	(((((...)))))	(((((...)))))	(((((...)))))	(((((...)))))	(((((...)))))	(((((...)))))	(((((...)))))	(((((...)))))
1K2G	..(((....))).((....)).	..(((....)))..........	..(((....))).((....)).	.(((((..)))))(((..))).	.(((((..)))))(((..))).	....	....	(..(((..)))<)((....))>	.(((((..)))))..........
1K4A	(((((....)))))	(((((....)))))	(((((....)))))	(((((....)))))	(((((....)))))	(((((....)))))	(((((....)))))	(((((....)))))	((((((..))))))
1K4B	(((((....)))))	((((((())))))))	(((((....)))))	((((((..))))))	((((((..))))))	(((((....)))))	(((((....)))))	((((((())))))))	((((((..))))))
1K5I	(((((((((.....)))))))))	(((((((((.....)))))))))	(((((((((.....)))))))))	((((((((((...))))))))))	((((((((((...))))))))))	(((((((((.....)))))))))	(((((((((.....)))))))))	(((((((((.....)))))))))	(((((((((.....)))))))))
1K6G	(((((((((....)))))))))	(((((((((....)))))))))	(((((((((....)))))))))	(((((((((....)))))))))	(((((((((....)))))))))	(((((((((....)))))))))	(((((((((....)))))))))	(((((((((....)))))))))	(((((((((....)))))))))
1K6H	(((((((((....)))))))))	(((((((((....)))))))))	(((((((((....)))))))))	(((((((((....)))))))))	(((((((((....)))))))))	(((((((((....)))))))))	(((((((((....)))))))))	(((((((((....)))))))))	(((((((((....)))))))))
1KAJ	(((((.........)))))..............	(((..(((((((..)).)))))).....)))..	....	((((((((((((..)).))))))...))))..	(((((..[[[[[[[)))))......]]]]]]]	(((((.........))))).............	(((((.........))))).............	<<<<<..((((((.>>>>>.......))))))	.((((<<<<{{{{.))))>>>>....}}}}..
1KKA	(((((((...)))))))	(((((((...)))))))	(((((.(....))))))	(((((((...)))))))	(((((((...)))))))	(((((((...)))))))	(((((((...)))))))	(((((((..).))))))	(((((((...)))))))
1KKS	((..((((((....))))))..))	((..((((((....))))))..))	....((((((....))))))....	((((((((((....))))))))))	((((((((((....))))))))))	((..((((((....))))))..))	((..((((((....))))))..))	((..((((((....))))))..))	...(((((((....)))))))...

PDB	MXfold2	CONTRAfold	Centroidfold	Mcfold Default	Mcfold Pseudoknotted	Linearfold C	Linearfold V	Ufold	SPOT-RNA
1KP7	((((.((....(((....)))..)).))))	((((.((....(((....)))..)).))))	((((.((....(((....)))..)).))))	(((((((...(((((..)))).)))))))	(((((((...(((((..)))).))))))))	((((.((....(((....)))..)).))))	((((.((....(((....)))..)).))))	((((.((..(.((((..))))).)).))))	((((.(...((((((..)))).))).))))
1KPD	(((((.........)))))............	(((((((....)).)))))............	....	((.(((((((((((..))))))))))))).	((((((.[[[[[[))))))......]]]]]]	(((((.........))))).............	(((((.........))))).............	<<<<<..((((((.>>>>>.......))))))	.((((<<<<.{{..))))>>>>.....}}...
1KXK	((.(.((..((((((...((((((((((((((....)))))..)))))))))))..)))))).)).).))	((...(((.((((((...((((((((((((((....))))))..))))))))))..))))))))))...))	......(..((((((...(((((((((.(((((....)))))...)))))))))..)))))).)......	((((((((.((((((.(((((((((((((((((..)))))))..)))))))))))))))))))))))	((((((((.((((((.((((((((((((((((((..)))))))..)))))))))))))))))))))))))	.....(((.((((((...((((((((((((((....))))))..)))))))))..))))))))).....	.....(((.((((((...((((((((((((((....))))))..)))))))))..))))))))).....	.(.(.((..(((((((..((((((((((((((....))))))..))))))))))).)))))))).)..).	((((.((..(((((((..(((((((((((((((..))))))..)))))))))))).)))))))..))))
1L1C	((.((((..((((....))))))))..))	((.((((..((((....))))))))..))	....(((..((((....))))))).....	((.((((((((((..))))))))))))	((.((((((((((..)))))))))))))	.........((((....))))........	.........((((....))))........	((.((((..(((((..))))))))))..))	(((((((..(((((..))))))))).)))
1L1W	((((..((((((....))))))..)))).	((((..((((((....))))))..)))).	((((..((((((....))))))..)))).	((((((((((((....)))))))))))).	((((((((((((....)))))))))))).	((((..((((((....))))))..)))).	((((..((((((....))))))..)))).	((((..((((((....))))))..))).	((((.((.((((....))))))..)))).
1LDZ	(((((....(((((....)))))..)))))	(((((....(((((....)))))..)))))	(((((....(((((....)))))..)))))	((((((...((((((..))))))).)))))	((((((...((((((..))))))).)))))	(((((....(((((....)))))..)))))	(((((....(((((....)))))..)))))	(((((....(((((....)))))..)))))	(((((...(((((((..))))))).)))))
1LNG	...(((..(((((.(((((((((....))))))))))..)))))....((((((.....(((.....(((....))).....)))..)))))).))).	..((((..(((((.(((((((((....)))))))))..)))))....((((((..(..(((.....(((....))).....)))).)))))).)))	..((((..(((((.(((((((((....)))))))))..)))))....((((((...(.........(((....)))........)..)))))).))))	..((((((((((((((((((((....)))))))).))))))))(.((((((((...((((((((((((..))))))))))))))))))))))))))	..(((((((((((((((((((....))))))))).))))))(.((((((((...(((((((((((..)))))))))))))))))))))))))	........(((((.(((((((((....)))))))))..)))))....((((((.....(((.....(((....))).....)))..))))))....	(((((((.(((((.(((((((((....)))))))))..)))))......))))))).........(((....)))....	.......((((((.(((((((((...)))))))))..)))))...)((((((..(((..(.....((((....)))..)..)))).)))))).....	<<......(((((.(((((((((..)))))))))..)))))....((((((>>...((((((((((((..))))))))))))).)))))).....
1M5L	((((....((((.((((....))))...))))..))))	((((....((((.((((....))))...))))..))))	((((....((((.((((....))))...))))..))))	(((((..((((((((((..)))))..))))))))))	(((((..((((((((((..))))..))))))))))))	((((....((((.((((....))))...))))..))))	((((....((((.((((....))))...))))..))))	((((....((((.(((((..)))))...))))..))))	(((((...((((((((((..))))).).)))).)))))
1M82	(((((((((((....))).))))))))	(((((((((((....)))).)))))))	((((((.(((....)))..))))))	(((((((((((..))))).)))))))	(((((((((((..))))).))))))	(((((((((((....)))).)))))))	(((((((((((....)))).)))))))	(((((((((((..))))).))))))	(((((((((((..)))).)))))))
1MFJ	..(((((((....)))))))	..(((((((....)))))))	..(((((((....)))))))	..((((((((..))))))))	..((((((((..))))))))	..(((((((....)))))))	..(((((((....)))))))	..((((((((..))))))))	..(((((((....)))))))
1MFK	((((((.(((....)))))))))	((((((.(((((())))))))))))	(((((..(((....))).)))))	((((((.((((..))))))))))	((((((.((((..))))))))))	((((((.(((....)))))))))	((((((.(((....)))))))))	((((((.(((((())))))))))))	((((((.((((..))))))))))
1MFY	(((.((((..((((....))))..)))))))	(((.((((..((((....))))..)))))))	....((((..((((....))))..))))...	(((.(((((((((((..))))))))))))))	(((.(((((((((((..)))))))))))))))	(((.((((..((((....))))..)))))))	(((.((((..((((....))))..)))))))	(((.(((((.(((((..)))))).)))))))	(((.(((..((((((..))))))..))))))
1MNX	((((.....(.((((((((....)))))))).).....))))	((((.(...(.((((((((....)))))))).)...).))))	.(....)....((((((((....))))))))..........	(((((((((((((((((((..)))))))))))))))))))	(((((((((((((((((((..))))))))))))))))))))	((((.......((((((((....)))))))).......))))	((((.......((((((((....)))))))).......))))	((((.(...(.((((((((....)))))).)...).))))	(((((((((((((((((((..)))))))))))))))))))
1MT4	((((((((........))))))))	((((((((........))))))))	((((((((..(....)))))))))	(((((((((((..)))))))))))	(((((((((((..))))))))))))	((((((((........))))))))	((((((((........))))))))	((((((((.(....).))))))))	((((((((........))))))))
1N66	((((..(((....)))..))))	((((..(((....)))..))))	((((..(((....)))..))))	((((((((((..))))))))))	((((((((((..)))))))))))	((((..(((....)))..))))	((((..(((....)))..))))	((((..(((....)))..))))	((((.(((((..))))).))))

PDB	MXfold2	CONTRAfold	Centroidfold	Mcfold Default	Mcfold Pseudoknotted	Linearfold C	Linearfold V	Ufold	SPOT-RNA
1N77	(((((((.(((((........)))))(((((.......)))))....(((((.......)))))))))))....	(((((((.(((((........)))))(((((.......)))))....(((((.......)))))))))))....	((((((...(((..........))).((((((.......)))))....(((((.......)))))).))))))....	(((((((.(((((((...))).))))(((((((...)))))))....((((((...)..))))))))))))....	((((...((((((...[[[[[)))))))))..((((((((((((((((..)))))))))))))))))]]]]].	(((((((.(((((........)))))(((((.......)))))....(((((.......)))))))))))....	(((((((.(((((........)))))(((((.......)))))....(((((.......)))))))))))....	(((((((..((((........)))).(((((.......)))))....(((((.......))))))))))))....	(((((((<.((((>.......))))((((((.......))))))...((((((...).)))))))))))....
1N8X	(((((((.(((((((....)))))))...))))))))	(((((((.(((((((....)))))))...)))))))	(((((((.(((((((....)))))))...)))))))	(((((((((((((((..)))))))))..)))))))	(((((((((((((((..))))))))..)))))))	(((((((.(((((((....)))))))...)))))))	(((((((.(((((((....)))))))...)))))))	(((((((.(((((((....)))))))...)))))))	(((((((.(((((((....)))))))...)))))))
1NA2	((((((....((.....))....)))))).	((((((....((.....))....)))))).	((((((.................)))))).	(((((((((((((...))))))))))))).	(((((((((((((...)))))))))))))).	((((((.................)))))).	((((((.................)))))).	((((((....((.....))....)))))).	(((((((....(.....)....))))))).
1NBR	((((((.(((((......)))))))))))	((((((.(((((......)))))))))))	((((((.(((((......)))))))))))	((((((.((((((...).)))))))))))	((((((.((((((...).))))))))))))	((((((.(((((......)))))))))))	((((((.(((((......)))))))))))	((((((.((((((...).)))))))))))	((((((.(((((((.)).)))))))))))
1NC0	((((((((.....))).))))))	((((((((.....))).))))))	((((((((.....))).))))))	(((((((((...)))).))))))	(((((((((...)))).)))))))	((((((((.....))).))))))	((((((((.....))).))))))	((((((((.....))).))))))	((((((((.....))).))))))
1NYB	((((((((......))))))))	((((((((......))))))))	(((((((........)))))))	(((((((((....)))))))))	(((((((((....)))))))))))	((((((((......))))))))	((((((((......))))))))	((((((((......))))))))	((((((((......))))))))
1OOA	(((((....((((....))))...)))))	(((((....((((....))))...)))))	(((((....((((....))))...)))))	(((((((((((((...)))))))))))))	(((((((((((((...)))))))))))))	(((((....((((....))))...)))))	(((((....((((....))))...)))))	(((((.(..((((....)))).).)))))	(((((....(((((..)))))...)))))
1OQ0	((((((...))))))	((((((...))))))	(((((.....)))))	((((((...))))))	((((((...))))))	(((((.....)))))	(((((.....)))))	((((((...))))))	(((((.....)))))
1OSW	((((.(((....)))...))))	((((.(((....)))...))))	((((.(((....)))...))))	((((((((..)))))..))))	((((((((..)))))..))))	((((.(((....)))...))))	((((.(((....)))...))))	((((.((((..))))...))))	((((((((....)))..)))))
1OW9	((((...(((....)))..))))	((((...(((....)))..))))	((((...(((....)))..))))	(((((.(((((..))))))))))	(((((.(((((..))))))))))	((((...(((....)))..))))	((((...(((....)))..))))	((((...(((....)))..))))	((((...((((..))))..))))
1P5M	(((((.((((.....((((...(((.((....)).)))....)))))))))))))	(((((.((((.....(((((..(((.((....)).)))..).)))))))))))))	(((((.((((............(((.((....)).)))........)))))))))	(((((.((((.....((((((((((((((..))))))))).))))))))))))	(((((.((((.....((((((((((((((..)))))))))).)))))))))))))	(((((.((((.....((((...(((.((....)).)))....)))))))))))))	(((((.((((.....((((...(((.((....)).)))....)))))))))))))	(((((.((((.....(((((..(((.(((..))).)))..).)))))))))))))	(((((.((((......((....(((((((.)))))))).....)).)))))))))
1P5N	((((....(((((.......)))))......))))	((((...((((((.......))))))....))))	((((...((((((.......))))))....))))	((((((((((.((((..))))))))).))))))	((((((((((.((((..))))))))).))))))	((((...((((((.......))))))....))))	((((...((((((.......))))))....))))	((((.(.((((((.......))))))..).))))	(((((..((((((.......))))))...)))))
1P5P	(((((.((((.....((((......))))..((((((.......)))))).(((((.....)))))..)))))))))	(((((.((((.....(((((..(((.((...((((((.....())))))))....)).)))..).)))))))))))))	(((((.((((..)))))))))	(((((.((((.....(((((((((((((((((((.((((..))))))))).)))))))))))).)))))))))))))	(((((.((((.....((((((((((((((((((.((((..)))))))))).)))))))))))).)))))))))))))	(((((.((((.....((((...(((.((...((((((.......))))))....)).))))....)))))))))))))	(((((.((((.....((((...(((.((...((((((.......))))))....)).)))....)))))))))))))	(((((.((((.....(((((..(((.(..(.((((((.......))))))..)..).)))..).))))))))))))	(((((.((((.....<<......((((>>...))))...............................)))))))))
1P6V	.......(((((((....)))))))...(((((.......)))))	.......(((((((....)))))))...(((((.......)))))	.......(((((((....)))))))...(((((.......)))))	(.(((((...(((((..))))))))))((((((...)..)))))	(.(((((...(((((..)))))))))))((((((...)..)))))	.......(((((((....)))))))...(((((.......)))))	.......(((((((....)))))))...(((((.......)))))	.......((((((((..))))))))...(((((.......)))))	<..(..((((((((((..)))))))))).((((((..>)..)))))
1PJY	((((((((......))))))))	(((((((((....)))))))))	(((((((((....)))))))))	(((((((((....)))))))))	(((((((((....)))))))))	(((((((((....)))))))))	(((((((((....)))))))))	(((((((((....)))))))))	(((((((((....)))))))))

PDB	MXfold2	CONTRAfold	Centroidfold	Mcfold Default	Mcfold Pseudoknotted	Linearfold C	Linearfold V	Ufold	SPOT-RNA
1Q75	(((((.....)))))	(((((.....)))))	(((((.....)))))	((((((...))))))	((((((...))))))	(((((.....)))))	(((((.....)))))	((((((..).)))))	(((((.....)))))
1QC8	(((.(((((((....))))))))))	(((.(((((((....))))))))))	....(((((((....)))))))...	(((.(((((((....))))))))))	(((.(((((((....)))))))))))	(((.(((((((....))))))))))	(((.(((((((....))))))))))	(((.(((((((....)))))))))))	(((.(((((((....))))))))))
1QFQ	(((((.....)))))	(((((.....)))))	(((((....).))))	((((((...))))))	((((((...))))))	(((((.....)))))	(((((.....)))))	(((((....).))))	(((((.....)))))
1QWA	((((((((....)).))))))	((((((((....)).))))))	((((((.........))))))	(((((((((..))).))))))	(((((((((..))).))))))	((((((.........))))))	((((((.........))))))	(((((((((..))).))))))	((((((.(....)..))))))
1QWB	((((((.............))))))	((((((.............))))))	((((((.............))))))	(((((((((.((..))).))))))))	(((((((((.((..))).))))))))	((((((.............))))))	((((((.............))))))	((((((.(..........).))))))	(((((((...(....)...)))))))
1R2P	((((((((((((((....)))))..)))))))))	((((((((((((((....))))))..))))))))	((((((((.(((((....)))))...))))))))	((((((((((((((((..)))))))..))))))))	((((((((((((((((..)))))))..))))))))	((((((((((((((....))))))..))))))))	((((((((((((((....))))))..))))))))	((((((((((((((....))))))..))))))))	((((((((((((((((..))))))..)))))))))
1R4H	(((....)))	(((....)))	(((....)))	((((..))))	((((..))))	(((....)))	(((....)))	((((..))))	((((..))))
1R7W	(((((((......(((((....)))))))))))))	(((((((......(((((....))))))))))))))	(((((((......(((((....))))))))))))	((((((((((((((((..)))))))))))))))))	((((((((((((((((..))))))))))))))))	(((((((......(((((....))))))))))))	(((((((......(((((....))))))))))))	(((((((......(((((....))))))))))))	(((((((......(((((....))))))))))))
1R7Z	(((((((......(((((....)))))))))))))	(((((((......(((((....))))))))))))))	(((((((......(((((....))))))))))))	(((((((......((((((..))))))))))))))	(((((((......((((((..)))))))))))))	(((((((......(((((....))))))))))))	(((((((......(((((....))))))))))))	(((((((......((((((....))))))))))))	(((((((......(((((....))))))))))))
1RFR	(((((...(((((....)))))...)))))	(((((...(((((....)))))...)))))	(((((...(((((....)))))...)))))	(((((((((((((((..)))))))))))))))	((((((((((((((..))))))))))))))	(((((...(((((....)))))...)))))	(((((...(((((....)))))...)))))	(((((...((((((..))))))...)))))	((((((..((((((..))))))..))))))
1RHT	.((((((.(......)))))))..	.((((((.((....))))))))..	.((((((.........))))))..	(((((((.((....))))))))).	(((((((.((....))))))))).	.((((((.((....))))))))..	.((((((.((....))))))))..	.((((((.((....))))))))..	.((((((.((....))))))))..
1RKJ	((((((((....)).))))))	((((((((....)).))))))	((((((.........))))))	(((((((((..))).))))))	(((((((((..))).))))))	((((((.........))))))	((((((.........))))))	(((((((((..))).))))))	((((((.(....)..))))))
1RNG	((((....))))	((((....))))	.(((....))).	(((((..)))))	(((((..)))))	....	....	(((((..)))))	(((((..)))))
1RNK	(((((.........)))))...............	((((((((....)).)))))..............	....	((((.((((((((..)).))))))).....)))...	((((((.[[[[[.))))))).(((..)))]]]]].	(((((.........)))))...............	(((((.........)))))...............	<<<<<.((((((((.>>>>>........))))))))	.<<<<{{((((((((.>>>>..}}.....)))))).
1ROQ	(((((....)))))	(((((....)))))	(((((....)))))	((((((..))))))	((((((..))))))	(((((....)))))	(((((....)))))	((((((..))))))	((((((..))))))
1S03	((((((.((.(..((((((((((....)))))))).)))))))))))	((((((.((....((((((((((....)))))))).)))).))))))	((((((.((......((((((((....))))))))....))))))))	((((((.((.(((..(((((((((..)))))))))).))))))))))	((((((.((.(((..(((((((((..)))))))))).))))))))))	((((((.((......((((((((....))))))))...)).))))))	((((((.((......((((((((....))))))))...)).))))))	((((((.((.((..(((((((((....)))))))).)))))))))))	(((((((((.....(((((((((....))))))).).)))))))))
1S2F	((((((.(((....)))))))))	((((((.(((....)))))))))	((((((...........))))))	((((((.((((..))))))))))	((((((.((((..))))))))))	((((((...........))))))	((((((...........))))))	((((((.(((....)))))))))	((((((..((....)).))))))
1S9S	.((((..((((((....))))))..))))..........((((..(((.(((((((((....)))))....))))))))))((((((((((....))))))))))	.((((..((((((....))))))..)))).....((((..(((.(((((((((....))))))....)))))))))))((((((((((....))))))))))	.((((..((((((....)))))).................((.((((.((((....))).....)))))).)))).(((((((((....))))))))).			.((((..((((((....))))))..))))).....((((..(((.(((((((((....)))))....)))))))))))((((((((((....))))))))))	.((((..((((((....))))))..))))).....((((..(((.(((((((((....)))))....)))))))))))...	.((((..((((((....))))))..)))).....((((..(((.(((((((((....)))))....)))))))))))((((((((((....))))))))))	.......((((((....)))))))....................((((((((....)))))....))))).......(((((((((((..))))))))))

PDB	MXfold2	CONTRAfold	Centroidfold	Mcfold Default	Mcfold Pseudoknotted	Linearfold C	Linearfold V	Ufold	SPOT-RNA
1SCL	((((((......(....).....))))))	((((((...((.(....)..)).))))))	((((((......(....).....))))))	(((((((((.((((..)))))))))))))	(((((((((.((((..))))))))))))))	((((((.................))))))	((((((.................))))))	((((((....(.(....).)...))))))	((((((...(((((..))).)).))))))
1SLP	(((((((....)).)))))	(((((((....)).)))))	.((((.........)))).	((((((((..))).)))))	((((((((..))).)))))	(((((.........)))))	(((((.........)))))	(((((((....)).)))))	(((((((....)).)))))
1SYZ	(((((.(((.....)))..)))))	(((((.(((.....)))..)))))	(((((.(((.....)))..)))))	((((((((((...))))).)))))	((((((((((...))))).)))))	(((((.(((.....)))..)))))	(((((.(((.....)))..)))))	(((((.(((.....)))..)))))	(((((.(((.....)))..)))))
1SZY	(((((((.......)))))))	(((((((.......)))))))	(((((((.......)))))))	(((((((((...)))))))))	(((((((((...)))))))))	(((((((.......)))))))	(((((((.......)))))))	(((((((.......)))))))	(((((((.......)))))))
1T28	((((((((((.............))))))))))	(((((((((((...........).))))))))))	((((((((((.............))))))))))	(((((((((((..)))(((....))))))))))	(((((((((((..)))(((....))))))))))	((((((((((.............))))))))))	((((((((((.............))))))))))	((((((((((.............))))))))))	((((((((((.............))))))))))
1TFN	.((((((.(......)))))))..	.((((((.((....))))))))..	.((((((.........))))))..	(((((((.((....))))))))).	(((((((.((....))))))))).	.((((((.((....))))))))..	.((((((.((....))))))))..	.((((((.((....))))))))..	.((((((.((....))))))))..
1TJZ	(((..((((....))))..)))	(((..((((....))))..)))	(....)(((....)))......	((((((((((..))))))))))	((((((((((..))))))))))	(((..((((....))))..)))	(((..((((....))))..)))	(((..((((....))))..)))	((((.(((((..))))).))))
1TLR	((((...(((....)))..))))	((((...(((....)))..))))	((((...(((....)))..))))	((((.((((((..))))))))))	((((.((((((..))))))))))	((((...............))))	((((...............))))	((((...((((..))))..))))	((((...((((..))))..))))
1TXS	((((..((((...(((((....)))))...))))))))	((((..((((...(((((....)))))...))))))))	((((..((((...(((((....)))))...))))))))	((((..(((((((((((((..)))))))))))))))))	((((..(((((((((((((..))))))))))))))))))	((((..((((...(((((....)))))...))))))))	((((..((((...(((((....)))))...))))))))	((((..((((...((((((..))))))...))))))))	((((..((((...((((((..))))))...))))))))
1U2A	(((((((......)))))))	(((((((......)))))))	(((((((......)))))))	((((((((....))))))))	((((((((....))))))))	(((((((......)))))))	(((((((......)))))))	(((((((......)))))))	((((((((...).)))))))
1U63	((((((((((..((((((((....)))).))))......))))))))))	((((((((((..((((((((....))))).)))......))))))))))	((((((((((..(((.(((......))).)))......))))))))))	(((((((((((((((((((((..))))).))))....))))))))))	((((((((((((((((((((..))))))).))))....)))))))))))	((((((((((..((((((((....)))).)))......))))))))))	((((((((((..((((((((....)))).)))......))))))))))	((((((((((..(((((((((..))))).)))......))))))))))	(((((((((((((((((((((..))))).)))))....))))))))))
1ULL	(((((..(((((((....))))..)))...)))))	(((((..(((((((....))))..)))...)))))	(((((..(((((((....))))..)))...)))))	(((((((((((((((..)))))..)))).))))))	(((((((((((((((..)))))..)))).))))))	(((((..(((((((....))))..)))...)))))	(((((..(((((((....))))..)))...)))))	(((((..((((((((..)))))..)))...)))))	(((((((((((((((..)))))..)))).))))))
1UN6	((((((....((....))(((((((.....((.((....)).))....))))))))))))	((((((....((....))(((((((..((.(((((...)))))).))))))))))))))	((((((....((....))(((((((.....((.((....)).))....))))))))))))	((((((.(((((...))))((((((((.((((((((..)))))))))))))))))))))	((((((.(((((...))))((((((.((((((((..)))))))))))))))))))))	((((((....((....))(((((((.....((.((....)).))....))))))))))))	((((((....((....))(((((((.....((.((....)).))....))))))))))))	((((((...........(((((((.....((.((....)).))....)))))))))))))	((((((....(((..)))(((((((((.((((((((..)))))))))))))))))))))
1UUU	((((((((...))))))))	((((((((...))))))))	((((((((...))))))))	((((((((...))))))))	((((((((...))))))))	((((((((...))))))))	((((((((...))))))))	((((((((...))))))))	((((((((...))))))))
1VOP	(((((...)))))	(((((...)))))	((((.....))))	(((((...)))))	(((((...)))))	....	....	(((((...)))))	((((.....))))
1WKS	((((((.....))))))	((((((.....))))))	((((((.....))))))	(((((((...)))))))	(((((((...)))))))	((((((.....))))))	((((((.....))))))	((((((.....))))))	((((((.....))))))
1WZ2	(((((((.......(((........))).((((((.......))))).(......).....((((((.......)))))))))))))....	((((((((....((((((......))))((((((.......))))))(((....))).)).((((.......)))))))))))))....	(((((((.......((((......)))).................................((((.......)))).)))))))....			(((((((.....((((((.......((.((((((.......)))))).))....))))))((((((.......)))))))))))).....	(((((((((..(((((((((.....((.((((((.......)))))).)).........)).)))))))))))))))..	(((((((..(((.............)))((((((.......))))))(((....)))...((((((.......))))))))))))....	(((((((..(((((.....<...))))))((((((.......))))))(((....)))...((((((.>.)..))))))))))))....

PDB	MXfold2	CONTRAfold	Centroidfold	Mcfold Default	Mcfold Pseudoknotted	Linearfold C	Linearfold V	Ufold	SPOT-RNA
1XHP	((((.((((.(((.....)))..)))).))))	((((.((((.(((.....)))..)))).))))	...(.((((..............)))).)...	(((((((((((((...))))).))))))))	(((((((((((((...)))).)))))))))	((((.((((.(((.....)))..)))).))))	((((.((((.(((.....)))..)))).))))	((((.((((.(((.....)))..)))).))))	((((((((.(((.....)))..)))))))))
1XSG	(((((.((((((....)))))))))))	(((((.((((((....)))))))))))	(((((.((((((....)))))))))))	(((((.(((((((..))))))))))))	(((((.(((((((..))))))))))))	(((((.((((((....)))))))))))	(((((.((((((....)))))))))))	(((((.(((((((..))))))))))))	(((((.(((((((..))))))))))))
1XSH	((((((.(((((....)))))))))))	((((((.(((((....)))))))))))	((((((.(((((....)))))))))))	((((((.((((((..))))))))))))	((((((.((((((..))))))))))))	((((((.(((((....)))))))))))	((((((.(((((....)))))))))))	((((((.((((((..))))))))))))	((((((.((((((..))))))))))))
1XWP	((((......)))).	((((......)))).	((((......)))).	((((.(...))))).	((((.(...))))).	((((......)))).	((((......)))).	((((......)))).	((((......)))).
1XWU	((((.......)))).	((((.......)))).	.(((.......)))..	((((((...)))))).	((((((...)))))).	....	....	((((.(...).)))).	((((((...)))))).
1YMO	((((((....((.....))....))))))......................	..............((((((((..(.......)...))))))))).	..............((((((((...............)))))))).	((((((((((((((((((...))))))...)))))))))))))...	(((((((((((((((((...)))))))...))))))))))))...	..............((((((((...............)))))))).	..............((((((((...............)))))))).	<<<<<<{{......(((((((((>>>>>>......}}))))))))).	<<<<<<.......(((((((((>>>>>>........)))))))))
1YN1	((((((.....))))))	((((((.....))))))	((((((.....))))))	(((((((...)))))))	(((((((...)))))))	((((((.....))))))	((((((.....))))))	((((((.....))))))	((((((.....))))))
1YNC	((((((((((........)))).)))))))	((((((((((........))).)))))))	((((((((((........)))).)))))))	((((((((((.((...)))))).)))))))	((((((((((.((...)))))).)))))))	((((((((((........))).))))))))	((((((((((........))).))))))))	((((((((((.((...)))))).)))))))	((((((((((.((...)))))).)))))))
1YNG	((((((((((........)))).)))))))	((((((((((........))).)))))))	((((((((((........)))).)))))))	((((((((((((((..))))))).)))))))	((((((((((((((..))))))).)))))))	((((((((((........))).))))))))	((((((((((........))).))))))))	((((((((((........)))).)))))))	(((((((((((......))))).)))))))
1YSV	((((((((((.....))))))))))	((((((((((.....))))))))))	((((((((((.....))))))))))	(((((((((((...)))))))))))	(((((((((((...)))))))))))	((((((((((.....))))))))))	((((((((((.....))))))))))	((((((((((.....))))))))))	((((((((((.....))))))))))
1Z2J	((((((((((((((((((......)))))))))...))))))))	((((((((((((((((((....))))))))))))...)))))))	((((((((((((((((((....)))))))))))...))))))))	((((((((((((((((((....))))))))))...))))))))	((((((((((((((((((....))))))))))...))))))))	((((((((((((((((((....)))))))))))...)))))))	((((((((((((((((((....)))))))))))...)))))))	((((((((((((((((((....))))))))))))...))))))))	((((((((((((((((((..)))))))))))))...)))))))
1Z30	(((((((....)))))))	(((((((....)))))))	(((((((....)))))))	((((((((..))))))))	((((((((..))))))))	(((((((....)))))))	(((((((....)))))))	(((((((....)))))))	(((((((....)))))))
1Z31	(((((.(((..(((....)))...))))))))	(((((.(((..(((....)))...))))))))	(((((.(((..(((....)))...))))))))	(((((.((((((((..)))))).))))))))	(((((.((((((((..)))))).))))))))	(((((.(((..(((....)))...)))))))))	(((((.(((..(((....)))...)))))))))	(((((.(((..((((..))))...))))))))	(((((.(((.(((((..)))))..))))))))
1ZBN	((((((.(.((((....))))))))))))	((((((.(.((((....))))))))))))	((((((...((((....)))).))))))	((((((.(.((((....))))))))))))	((((((.(.((((....))))))))))))	((((((...((((....)))).))))))	((((((...((((....)))).))))))	((((((.(.((((....))))))))))))	((((((.(.((((....))))))))))))
1ZC5	(((((((((((((((......))))))))))...))))))	((((((((((((((((....)))))))))))...)))))	((((((((((((((((....))))))))))...))))))	((((((((((((((((....)))))))))))...))))))	((((((((((((((((....))))))))))...))))))	((((((((((((((((....))))))))))...)))))	((((((((((((((((....))))))))))...)))))	((((((((((((((((....)))))))))))...))))))	((((((((((((((((....))))))))))))...)))))
1ZIF	((((....))))	((((....))))	.(((....))).	(((((..)))))	(((((..)))))	((((....))))	((((....))))	((((....))))	((((....))))
1ZIG	((((....))))	((((....))))	.(((....))).	(((((..)))))	(((((..)))))	((((....))))	((((....))))	((((....))))	((((....))))
1ZIH	((((....))))	((((....))))	.(((....))).	(((((..)))))	(((((..)))))	((((....))))	((((....))))	((((....))))	((((....))))
28SP	(((((....(((....)))....)))))	(((((....(((....)))....)))))	(((((....(((....)))....)))))	((((.((((((((..))))).))))))))	((((.((((((((..))))).))))))))	(((((....(((....)))....)))))	(((((....(((....)))....)))))	(((((..(.(((....)))..).))))))	(((((...(((((..)))))...)))))
2A9L	((((((((((...((((....))))))))))...)))))	((((((((((...((((....))))))))))...))))))	((((((((((...((((....)))))))))...))))))	((((((((((...(((((..))))))))))...))))))	((((((((((...(((((..)))))))))...))))))	((((((((((...((((....)))))))))...))))))	((((((((((...((((....)))))))))...))))))	((((((((((...((((....))))))))...))))))	((((((((((...(((((..))))))))))...))))))

PDB	MXfold2	CONTRAfold	Centroidfold	Mcfold Default	Mcfold Pseudoknotted	Linearfold C	Linearfold V	Ufold	SPOT-RNA
2ADT	(((((..(((((((((((....)))))))))))))...)))))	(((((..(((((((((((....))))))))))))...)))))	.((((..(((((((((((....))))))))))))...)))).	(((((((((((((((((((..)))))))))))))))))))).	((((((((((((((((((((..))))))))))))))))))))).	(((((..(((((((((((....))))))))))))...)))))	(((((..(((((((((((....))))))))))))...)))))	(((((.((((((((((((....)))))))))))).).)))))	(((((.((((((((((((....)))))))))))))..)))))
2AHT	((((((((((....)))).)))))))	((((((((((....)))).)))))))	((((((((((....)))).)))))))	(((((((((((..))))).)))))))	(((((((((((..))))).)))))))	((((((((((....)))).)))))))	((((((((((....)))).)))))))	((((((((((....)))).)))))))	((((((.(((((..)))))..))))))
2AKE	(((((((..((((.......)))).((((((.......)))))....(((((.......)))))))))))).	(((((((..((((.......)))).(((((.......))))).....(((((.......))))))))))))).	((((((...((((.......))))..((((.......))))........((((.......))))..)))))).	((((((((((((((...)))))(((((((((..)).)))))))(((((((..))))))).)))))))))).	((((((((((((((...))))))((((.[[[[[))))))))))))))((((((...)..))))).]]]]]..	(((((((..((((.......)))).(((((.......))))).....(((((.......)))))))))))).	(((((((..((((.......)))).(((((.......))))).....(((((.......)))))))))))).	(((((((..((((.......)))).(((((.......))))).....(((((.......)))))))))))).	(((((((..(((((.....)))))((((((.......))))))....((((((...)..)))))))))))).
2ANN	((((..............))))	((((..............))))	.((................)).	((((((((((...))))))))))	((((((((((...))))))))))	....	....	((((..............))))	((((..............))))
2B6G	(((((((.....)))))))	(((((((.....)))))))	(((((((.....)))))))	((((((((...))))))))	((((((((...))))))))	(((((((.....)))))))	(((((((.....)))))))	(((((((.....)))))))	(((((((.....)))))))
2B7G	(((((((.....)))))))	(((((((.....)))))))	(((((((.....)))))))	((((((((...))))))))	((((((((...))))))))	(((((((.....)))))))	(((((((.....)))))))	(((((((.....)))))))	(((((((.....)))))))
2DER	(((((((..((((.........))))((((((.......))))))...(((((.......))))))))))))..	(((((((..((((.(.(()).).))))((((((.......))))))...((((((....).))))))))))))..	(((((((..(((..........)))((((((.......))))))...(((((.......))))))))))))..	((((((((((...)))((((..))))((((((((...))))))))...((((((...)..))))))))))))..	((((((((((...)))((((..))))((((((((...))))))))...((((((...)..)))))))))))..	(((((((..((((.........))))((((((.......))))))...(((((.......))))))))))))..	(((((((..((((.........))))((((((.......))))))...(((((.......))))))))))))..	(((((((..((((.(..<{)..))))((((((.......))))))...((((((.>})..)))))))))))).	(((((((..(((((.......)))))((((((.......))))))...((((((...)..))))))))))))..
2DET	..(((((..((((.........))))((((((.......))))))...(((((.......)))))))))	..(((((..((((.(.....).))))((((((.......))))))...((((((....).))))))))))	...(((...(((..........)))((((((.......))))))...(((((.......)))))).))).	.((((((((((((..))))))))))((((((((((((...))))))))))))((((((...)..))))).))))	((((.((((((...))))))[[[[))))((((((((...))))))))...((((((...)..))))).]]]]	..(((((..((((.........))))((((((.......))))))...(((((.......)))))))))))	..(((((..((((.........))))((((((.......))))))...(((((.......)))))))))))	.((((.(..((((.(..<.)..))))((((((.......))))))...((((((.>.)..))))))))))	..(..((..((((.........))))(((((((.....)))))))...((((((...)..)))))))..)
2DR2	(((((((..((((.......)))).((((((.......)))))....(((((.......))))))))))))....	(((((((..((((.......)))).(((((.......))))).....(((((.......))))))))))))....	((((((...((((.......))))...(((.......)))........((((.......))))..))))))....	((((((((((((((...))))))(((((....))))))))))))))((((((...)..))))).((((..))))	..((((((((((((.[[[[))))((((((((((..)).)))))))(((((((..))))))).))))))))..]]]]	(((((((..((((.......)))).(((((.......))))).....(((((.......))))))))))))....	(((((((..((((.......)))).(((((.......))))).....(((((.......))))))))))))....	(((((((..((((.......)))).(((((.......))))).....(((((.......))))))))))))....	(((((((..(((((.....)))))((((((.......))))))....((((((...)..))))))))))))....
2DRB	((((((((((((.......))))))))))))....	((((((((((((.......))))))))))))....	((((((((((((.......))))))))))))....	(((((((((((((...)..))))))))))))....	(((((((((((((...)..))))))))))))....	((((((((((((.......))))))))))))....	((((((((((((.......))))))))))))....	(((((((((((((...)..))))))))))))....	(((((((((((((...)..))))))))))))....
2DU3	(((((((..(((.........))).((((((.......)))))....(((((.......))))))))))))	((((((((((.((((.((.(((((((....))))))).)).)))).))((((.......))))))))))))	(((((((..((((((...))))))((((((.......))))))....(((((.......))))))))))))	((((((((((((((((((((((((((((..)))))))))))))))))))((((((...)..))))))))))))	((((((((((((((((((((((((((..)))))))))))))))))))))((((((...)..))))))))))))	((((((((((.((((.((.(((((((....))))))).)).)))).))((((.......))))))))))))	((((((((((.((((.((.(((((((....))))))).)).)))).))((((.......))))))))))))	(((((((..((((.......))))((((((.......))))))....(((((.......))))))))))))	(((((((..(((((<....)))))((((((.......))))))...>((((((...)..))))))))))))
2DU5	(((((((..(((.........))).((((((.......)))))....(((((.......))))))))))))	((((((((.((((((...))))))((((((.......))))))....((((.......)))))))))))	(((((((..((((((...))))))((((((.......))))))....(((((.......))))))))))))	((((((((((((((((((((((((((((..)))))))))))))))))))((((((...)..))))))))))))	((((((((((((((((((((((((((..)))))))))))))))))))))((((((...)..))))))))))))	(((((((..((((((...))))))((((((.......))))))....(((((.......))))))))))))	(((((((..((((((...))))))((((((.......))))))....(((((.......))))))))))))	(((((((..((((.......))))((((((.......))))))....(((((.......))))))))))))	(((((((..(((((<....)))))(((((((.....)))))))...>((((((...)..))))))))))))

PDB	MXfold2	CONTRAfold	Centroidfold	Mcfold Default	Mcfold Pseudoknotted	Linearfold C	Linearfold V	Ufold	SPOT-RNA
2DU6	(((((((..(((.........))).(((((.......)))))......(((((.......))))))))))))	((((((((.((((((...))))))((((((.((()))))))))).....((((.......))))))))))))	(((((((..((((((...))))))((((((.......))))))....(((((.......))))))))))))	((((((((((((((((((((((((((((..)))))))))))))))))))((((...)..)))))))))))	((((((((((((((((((((((((((..))))))))))))))))))))((((...)..))))))))))))	(((((((..((((((...))))))((((((.......))))))....(((((.......))))))))))))	(((((((..((((((...))))))((((((.......))))))....(((((.......))))))))))))	(((((((..((((.......))))((((((.......))))))....(((((.......))))))))))))	(((((((..(((((<....)))))(((((((.......))))))...>((((((...)..))))))))))))
2ES5	(((((((((.....)))))))))	(((((((((.....)))))))))	(((((((((.....)))))))))	((((((((((...))))))))))	((((((((((...))))))))))	(((((((((.....)))))))))	(((((((((.....)))))))))	((((((((((..).)))))))))	(((((((((.....)))))))))
2EUY	((((((.((..((((....)))).))..))))))	((((((.((..((((....)))).))..))))))	.......(....)...........((....))...	(((((((((((((((..))))))))))))))))	(((((((((((((((..))))))))))))))))	((((((.((..((((....)))).))..))))))	((((((.((..((((....)))).))..))))))	((((((.((..(((((..))))).))..))))))	((((((.((..(((((..))))).))..)))))
2EVY	(((((....)))))	(((((....)))))	(((((....)))))	((((((..))))))	((((((..))))))	(((((....)))))	(((((....)))))	(((((....)))))	(((((....)))))
2F87	((((....))))	((((....))))	((((....))))	((((....))))	((((....))))	((((....))))	((((....))))	((((....))))	((((....))))
2F88	(((((((((((((....)))))..)))))))))	(((((((((((((....)))))..)))))))))	(((((((((((((....)))))..)))))))))	((((((((((((((..))))))))..))))))))	((((((((((((((..))))))))..))))))))	(((((((((((((....)))))..)))))))))	(((((((((((((....)))))..)))))))))	(((((((((((((....)))))..)))))))))	((((((((((((((..))))))))..))))))))
2FDT	(((((((.(.((((((.....)))))).))))))))	(((((((.(.((((((.....)))))).))))))))	(((((((.(.((((((.....)))))).)))))))))	(((((((.((((((((...))))))))))))))))	(((((((.((((((((...))))))))))))))))	(((((((.(.((((((.....)))))).))))))))	(((((((.(.((((((.....)))))).))))))))	(((((((.(.((((((.....)))))).))))))))	(((((((...((((((.....))))))..))))))
2FEY	(((((.(((.(...((((((.......))))))).))))))))	(((((.(((..((.((((((.......)))))))))))))))))	(((((...(..((..(((((.......))))).)))..)))))	(((((.(((..((((.((((((...))))))))))))))))))))	(((((.(((..((((.((((((...)))))))))))))))))))))	(((((.(((..((.((((((.......)))))))))))))))))))	(((((.(((..((.((((((.......)))))))))))))))))))	(((((..((..(((.(((((....(.))))))))))).)))))	(((((..((..(((.(((((.......)))))))))).)))))
2FY1	((((((((.....))))))))	((((((((.....))))))))	((((((((.....))))))))	(((((((((...)))))))))	(((((((((...)))))))))	((((((((.....))))))))	((((((((.....))))))))	((((((((.....))))))))	((((((((.....))))))))
2G1W	((((.....))))..........	((((.....))))..........	.(((.....)))...........	(((((((.(.....))))))))	((((.[[[[)))).....]]]].	((((.....))))..........	((((.....))))..........	((((<<<<.))))......>>>>	((((.....))))..........
2GIO	(((((...(.(((....))).)..)))))	(((((((...(((....)))))..)))))	(((((.....(((....)))....)))))	((((((.(((((((..))))))))))))))	((((((.(((((((..))))))))))))))	(((((.....(((....)))....)))))	(((((.....(((....)))....)))))	(((((..((.((((..))))))..))))))	((((((((.(((((..))))))))))))))
2GIP	(((((..(((((....)))))..)))))	((((((((((....))))))..)))))	(((((..(((((....)))))..)))))	(((((((((((((..)))))))))))))	(((((((((((((..)))))))))))))	(((((..(((((....)))))..)))))	(((((..(((((....)))))..)))))	(((((..((((((..))))))..)))))	(((((((((((((..)))))))))))))
2GRW	((((.((((....)))).))))	((((.((((....)))).))))	((((.((((....)))).))))	((((((((((..))))))))))	((((((((((..))))))))))	((((.((((....)))).))))	((((.((((....)))).))))	((((.((((....)))).))))	((((((((((..))))))))))
2GV3	(((((((........)))))))	(((((((.(....).)))))))	(((((((........)))))))	((((((((((..))))))))))	((((((((((..))))))))))	(((((((........)))))))	(((((((........)))))))	(((((((.(....).)))))))	(((((((........)))))))
2GV4	(((((.(((....))).)))))	(((((.(((....))).)))))	(((((.(((....))).)))))	((((((((((..))))))))))	((((((((((..))))))))))	(((((.(((....))).)))))	(((((.(((....))).)))))	(((((.(((....))).)))))	((((((((((..))))))))))
2GVO	((((((......))))))	((((((......))))))	((((((......))))))	((((((((..))))))))	((((((((..))))))))	((((((......))))))	((((((......))))))	((((((......))))))	((((((......))))))
2HEM	(((..(((((....)))))..)))	(((..(((((....)))))..)))	(((..(((((....)))))..)))	(((((((((((..)))))))))))	(((((((((((..)))))))))))	(((..(((((....)))))..)))	(((..(((((....)))))..)))	(((..((((((..))))))..)))	(((((((((((..)))))))))))
2HGH	((((....((....))(((((((...((((.....)))).....)))))))))))	((((((.......)).(((((((...(.((.(....).))..).)))))))))))	((((............(((((((.....((.(....).))....)))))))))))	((((....(((..)))(((((((((.((((((..)))))))))))))))))))))	((((....(((..)))((((((((((.(((((((..))))))))))))))))))))	((((....((....))(((((((.....((.(....).))....)))))))))))	((((....((....))(((((((.....((.(....).))....)))))))))))	..............(((((((((...(.((.(....).)).)..)))))))).).)	((((....((....))(((((((.(.(((((((..)))))))).)))))))))))

PDB	MXfold2	CONTRAfold	Centroidfold	Mcfold Default	Mcfold Pseudoknotted	Linearfold C	Linearfold V	Ufold	SPOT-RNA
2HNS	((((((((....))))))))	((((((((....))))))))	((((((((....))))))))	(((((((((..)))))))))	(((((((((..)))))))))	((((((((....))))))))	((((((((....))))))))	(((((((((..)))))))))	(((((((((..)))))))))
2HUA	((((((.((.....((....))....))....))))))))	((((((.((...((((....)).)).)).)).....)))))))	((((((........((....))..........))))))	(((((((((((.(((((..))))))))))...)))))))	(((((((((((.(((((..)))))))))...)))))))	............(((((((...........)))))))	............(((((((...........)))))))	((((((.((.(.(.(((..))).)).)).)....)))))))	((((((.......((((..))))..........))))))
2HW8	((((((((..(((....)))......)))))))))	(((((((((.(((....))).)....)))))))))	((((((((..(((....)))......)))))))))	(((((((((((((((..))))))....)))))))))	(((((((((((((((..))))))....)))))))))	((((((((..(((....)))......)))))))))	((((((((..(((....)))......)))))))))	(((((((((.((((..)))).)....)))))))))	(((((((((((((((..)))))....))))))))))
2IHX	(((((((...((((.(((((.......)))))))))....((((.....))))(((....)))...))))))))	(((((((((.((((.(((((.......)))))))))))..((((.....))))(((())))))...))))))))	((((((((..((....))....))))))))	((((((((((((((((((((((((((((.((((...)))))))))))))))))).)))))))))))))))	(((((((((((((((((((((((((((((.((((...))))))))))))))))).)))))))))))))))	(((((((...((((.(((((.......)))))))))))....((((.......))))...........))))))))	(((((((...((((.(((((.............)))))))))....((((.......)))).........))))))))	(((((((((<.(((.(((((.......)))))))).))(.((((......)))).)....>.....)))))))	(((((((...((((.((((.........))))))))....((((.....))))..............))))))))
2IXY	((((((((((......))))))).))))	((((((((((......))))))).))))	((((((((((......))))))).))))	((((((((((((..)))))))).))))	((((((((((((..)))))))).))))	((((((((((......))))))).))))	((((((((((......))))))).))))	((((((((((...).))))))).))))	((((((((((...).)))))).)))))
2IXZ	((...)).	....	....			....	....	((...).)	.(...)..
2JPP	((((((((....))))))))	((((((((....))))))))	((((((((....))))))))	(((((((((..)))))))))	(((((((((..)))))))))	((((((((....))))))))	((((((((....))))))))	((((((((....))))))))	((((((((....))))))))
2JR4	(((((.......)))))	(((((.......)))))	(((((.......)))))	(((.((((..)))))))	(((.((((..)))))))	(((((.......)))))	(((((.......)))))	(((((.......)))))	((((((.....))))))
2JSE	((((..(((....)))..))))	((((..(((....)))..))))	((((..(((....)))..))))	(((((((((..)))))))))	(((((((((..)))))))))	((((..(((....)))..))))	((((..(((....)))..))))	(((((.(((....)))).))))	((((..((((..))))..))))
2JTP	((((((((((...((...))..))))))))))	((((((((((...((...))..))))))))))	((((((((((...........))))))))))	(((((((((((.(((...))))))))))))))	(((((((((((.(((...))))))))))))))	((((((((((...........))))))))))	((((((((((...........))))))))))	((((((((((...((...))..)))))))))))	(((((((((((..((...)).))))))))))))
2JWV	(((((....((((....))))...)))))	(((((....((((....))))...)))))	(((((....((((....))))...)))))	((((((((((((...))))))))))))	((((((((((((...)))))))))))))	(((((....((((....))))...)))))	(((((....((((....))))...)))))	(((((.(..((((....)))).).).))))	(((((....(((((..)))))...)))))
2JXV	((((((((..((((....))))...))))))))	((((((((.(((((....)))).).))))))))	((((((((..((((....))))...))))))))	((((((((((((((((..))))).))))))))))	((((((((((((((((..)))))).))))))))))	((((((((..((((....))))...))))))))	((((((((..((((....))))...))))))))	((((((((.(((((....)))).).))))))))	((((((((..(((((..)))))...))))))))
2JYM	(((((.(((.....))))))))	(((((.(((.....))))))))	(((((.(((.....))))))))	(((((.((((..).))))))))	(((((.((((..).))))))))	(((((.(((.....))))))))	(((((.(((.....))))))))	(((((.((((..).))))))))	(((((.((((..).))))))))
2K4C	(((((((..((((........)))).(((((.......)))))......(((((.......))))))))))))....	(((((((..((((........))))..((((.......))))((((((((....)))))))).. .)))))))....	(((((((..((((........))))..(((.........))).((((((......))))))....)))))))....	(((((..((((...))))((((((((((.((((..))))))))))((((((((..)))))))))))))))))))	(((((((.(((...)))[[[[[)))))))..]]]]].(((((((((((((((...)..))))))))))))))))	(((((((..((((........))))..((((.......))))((((((((....))))))))...)))))))....	(((((((..((((........))))..((((.......))))((((((((....))))))))...)))))))....	(((((((<.((((><..{[..)))).(((((.......)))))....>((((((}].)..))))))))))))....	(((((((<.((((><......))))(((((((.....)))))))...>((((((...)..))))))))))))....
2K5Z	(((((((((.((....)).))))).))))	(((((((((.((....)).))))).))))	((((..((...........))...))))	((((((((((((....)))))))).))))	((((((((((((....))))))).))))	(((((((((.((....)).))))).))))	(((((((((.((....)).))))).))))	(((((((((.((....)).))))).))))	((((.((((.((....)).)..)))))))
2K66	((((((((....))))))))	((((((((....))))))))	((((((((....))))))))	(((((((((..)))))))))	(((((((((..)))))))))	((((((((....))))))))	((((((((....))))))))	((((((((....))))))))	((((((((....))))))))

PDB	MXfold2	CONTRAfold	Centroidfold	Mcfold Default	Mcfold Pseudoknotted	Linearfold C	Linearfold V	Ufold	SPOT-RNA
2K95	((((((....((.....))....))))))......................	((((((....((.....))....)))))).......................	....	(((((((((((((((((...))))))....)))))))))))...	(((((((((((.[.[[[[])))))))))...((((...))))]]]]]	((((((.................))))))..................	((((((.................))))))..................	<<<<<<{{......(((((((((>>>>>>......}}))).))))).	((((((........<<<<<<...))))))...........>>>>>>.
2KD8	(((((((((....)))))))))	(((((((((....)))))))))	(((((((........)))))))	((((((((((..))))))))))	((((((((((..))))))))))	(((((((((....)))))))))	(((((((((....)))))))))	(((((((((....)))))))))	((((((((......))))))))
2KE6	((((((((((((((((((........)))))).))).)))))))))	((((((((((((((((((........)))))).))).)))))))))	((((((((((((((((((........))))))).)))).))))))))	((((((((((((((((((((....))))))))).))).)))))))))	((((((((((((((((((((....)))))))))).))).))))))))	((((((((((((((((((........)))))))).))).)))))))))	((((((((((((((((((........)))))))).))).)))))))))	((((((((((((((((((........)))))))).)))).))))))))	((((((((((((((((((((......))))))))).)))).))))))))
2KHY	((((((.......((((....)))).......))))))	((((((....(((((((....))))...))).))))))	((((((.......((((....)))).......))))))	((((((((((((((((((..)))))))))))))))))))	((((((((((((((((((..))))))))))))))))))	((((((.......((((....))))....)))))))	((((((.......((((....))))....)))))))	((((((..(....(((((..))))).....)...))))))	((((((..(....(((((..)))))...)...))))))
2KMJ	(((((((..((((....)))))))))))	(((((((..((((....)))))))))))	(((((((..((((....)))))))))))	(((((((..(((((..))))))))))))	(((((((..(((((..))))))))))))	(((((((..((((....)))))))))))	(((((((..((((....)))))))))))	(((((((..(((((..))))))))))))	(((((((..(((((..))))))))))))
2KOC	(((((....)))))	(((((....)))))	(((((....)))))	((((((..))))))	((((((..))))))	(((((....)))))	(((((....)))))	((((((..))))))	((((((..))))))
2KPC	((((((.....))))))	((((((.....))))))	.(((((.....))))).	((((((.....))))))	((((((.....))))))	((((((.....))))))	((((((.....))))))	((((((.....))))))	((((((.....))))))
2KPD	(((((.......)))))	(((((.......)))))	.((((.......)))).	((((((.....))))))	((((((.....))))))	(((((.......)))))	(((((.......)))))	(((((.......)))))	(((((.......)))))
2KPV	((((((((..((((....))))...)))).))))	((((((((.(((((....)))).).)))).))))	((((((((..((((....))))...)))).))))	((((((((((((((..))))).)))))).))))	((((((((((((((..)))))).)))))).))))	((((((((..((((....))))...)))).))))	((((((((..((((....))))...)))).))))	((((((((.(((((....)))).).)))).))))	((((((((..(((((..)))))...)))).))))
2KRL	((((((((....))))))))......(((((((((.........)))))))))...((((.(((((......(((....)))......))))).)))).....	((((((((....))))))))......((((((((.........)))))))))...((((.((((((......(((....)))......))))).)))))....	........(((((........)))))(((((((((..(....).)))))))))...((((.(((((......(((....)))......)))))).))))....			((((((((....))))))))......(((((((((.........)))))))))...((((.(((((......(((....)))......)))))).))))....	((((((((....))))))))......(((((((((.........)))))))))..............................	((((((((....))))))))......((((((((((..<<<<...))))))))...((((.(((((....(.(((....))))......))))).))))>>>>	(((((((......))))))).......((((((((((.......)))))))))).......(((((......((((..))))......)))))........).
2KRP	(((((.......)))))	(((((.......)))))	(((((.......)))))	((((((...)..)))))	((((((...)..)))))	(((((.......)))))	(((((.......)))))	(((((.......)))))	((((((.....))))))
2KUR	((((((((((((((((((........)))))).))).)))))))))	((((((((((((((((((........)))))).))).)))))))))	((((((((((((((((((........))))))).)))).))))))))	((((((((((((((((((((....))))))))).))).)))))))))	((((((((((((((((((((....)))))))))).))).))))))))	((((((((((((((((((........)))))))).))).)))))))))	((((((((((((((((((........)))))))).))).)))))))))	((((((((((((((((((........)))))))).)))).))))))))	((((((((((((((((((((......))))))))).)))).))))))))
2KUU	((((((((((((((((((........)))))).))).)))))))))	((((((((((((((((((........)))))).))).)))))))))	((((((((((((((((((........))))))).)))).))))))))	((((((((((((((((((((....))))))))).))).)))))))))	((((((((((((((((((((....)))))))))).))).))))))))	((((((((((((((((((........)))))))).))).)))))))))	((((((((((((((((((........)))))))).))).)))))))))	((((((((((((((((((........)))))))).)))).))))))))	((((((((((((((((((((......))))))))).)))).))))))))
2KUV	((((((((((((((((((........)))))).))).)))))))))	((((((((((((((((((........)))))).))).)))))))))	((((((((((((((((((........))))))).)))).))))))))	((((((((((((((((((((....))))))))).))).)))))))))	((((((((((((((((((((....)))))))))).))).))))))))	((((((((((((((((((........)))))))).))).)))))))))	((((((((((((((((((........)))))))).))).)))))))))	((((((((((((((((((........)))))))).))).)))))))))	((((((((((((((((((((......))))))))).)))).))))))))
2KUW	((((((((((((((((((........)))))).)))).))))))))	((((((((((((((((((........)))))).))).)))))))))	((((((((.((((((((((........))))))).)))..))))))))	((((((((((((((((((((....))))))))).))).)))))))))	((((((((((((((((((((....)))))))))).))).))))))))	((((((((((((((((((........)))))))).))).)))))))))	((((((((((((((((((........)))))))).))).)))))))))	(((((((((((((((((((.....).))))))).))).)))))))))	((((((((((((((((((((......))))))))).)))).))))))))
2KVN	(((((......))))).	(((((......))))).	(((((......))))).	(((((((..))))))).	(((((((..))))))).	(((((......))))).	(((((......))))).	(((((.(..).))))).	(((((......))))).

PDB	MXfold2	CONTRAfold	Centroidfold	Mcfold Default	Mcfold Pseudoknotted	Linearfold C	Linearfold V	Ufold	SPOT-RNA
2KZL	((((((......((((.......((((....))))........))))...))))))	((((((..(...((((....(((((((....))))...))).)))))..))))))	((((((.......(((.......((((....))))........)))....))))))	((((((((...(((((((((((((((..)))))))))))))))))))))))))	((((((((...(((((((((((((((..)))))))))))))))))))))))))	((((((......((((.......((((....))))........))))...))))))	((((((......((((.......((((....))))........))))...))))))	((((((....(.((((..(..(.(((((..))))).).)...)))))..))))))	(((((((....(((((..(...((((((..))))))..)...))))).)))))))
2L2J	((((.(((((((((.((((....)))).))))))))).))))	((((.(((((((((.((((....)))).)))))))))).))))	((((.(((((((((.((((....)))).))))))))).))))	((((((((((((((((((((..)))))))))))))))))))	((((((((((((((((((((..))))))))))))))))))))	((((.(((((((((.((((....)))).))))))))).))))	((((.(((((((((.((((....)))).))))))))).))))	((((.(((((((((.(((((..)))).))))))))).))))	(((((((((((((.(((((..))))).))))))))))))))
2L3C	((((.((((((((((....)))))))))).))))	((((.((((((((((....)))))))))).))))	((((.((((((((((....))))))))))).))))	((((((((((((((((..))))))))))))))))	(((((((((((((((..))))))))))))))))	((((.((((((((((....))))))))))).))))	((((.((((((((((....))))))))))).))))	((((.(((((((((((..))))))))))).))))	((((((((((((((....)))))))))))))))
2L3E	(((((((.....((((((....))))))))))))	(((((((.....((((((....))))))))))))	(((((((.....((((((....)))))))))))))	(((((((.....(((((((..))))))))))))))	(((((((.....(((((((..))))))))))))))	(((((((.....((((((....)))))))))))))	(((((((.....((((((....)))))))))))))	(((((((.....(((((((..))))))))))))))	(((((((.....(((((((..))))))))))))))
2L3J	(((((((.(((((((((.(((.(((((((((((.....)))))))))))).))).))))))))).)))))))	(((((((.(((((((((.(((.(((((((((((.....)))))))))))).))).))))))))).)))))))	(((((((.(((((((((.(((.(((((((((((.....)))))))))))).))).))))))))).)))))))	((((((((((((((((((((((((((((((((((...))))))))))))))))))))))))))))))))))	((((((((((((((((((((((((((((((((...))))))))))))))))))))))))))))))))))))	(((((((.(((((((((.(((.(((((((((((.....)))))))))))).))).))))))))).)))))))	(((((((.(((((((((.(((.(((((((((((.....)))))))))))).))).))))))))).)))))))	(((((((.(((((((((.(((.(((((((((((.....))))))))))).))).))))))))).)))))))	((((((((((((((((((((((((((((((((((...))))))))))))))))))))))))))))))))))
2L5Z	((((((...(((....))).))))))	((((((...(((....))).))))))	((((((...(((....))).))))))	((((((..(((((..)))))))))))	((((((..(((((..)))))))))))	((((((...(((....))).))))))	((((((...(((....))).))))))	((((((...(((....))).))))))	((((((..(((((..)))))))))))
2L6I	(((((.....))))).	(((((.....))))).	(((((.....))))).	((((((..).))))).	((((((..).))))).	(((((.....))))).	(((((.....))))).	((((((..).))))).	(((((.....))))).
2LAC	(((((.......)))))	(((((.......)))))	(((((.......)))))	(((((.((..)))))))	(((((.((..)))))))	(((((.......)))))	(((((.......)))))	(((((.(...).)))))	((((((.....))))))
2LBJ	(((((((...)))))))	(((((((...)))))))	((((((.....))))))	(((((((...)))))))	(((((((...)))))))	((((((.....))))))	((((((.....))))))	(((((((...)))))))	(((((((...)))))))
2LBK	((((((.....))))))	((((((.....))))))	((((((.....))))))	(((((((...)))))))	(((((((...)))))))	((((((.....))))))	((((((.....))))))	((((((.....))))))	((((((.....))))))
2LBL	(((((.......)))))	(((((.......)))))	(((((.......)))))	(((((.((..)))))))	(((((.((..)))))))	(((((.......)))))	(((((.......)))))	(((((.......)))))	((((((.....))))))
2LBS	((((((((((((((....))))))))))))))	((((((((((((((....))))))))))))))	((((((((((((((....))))))))))))))	((((((((((((((..))))))))))))))	((((((((((((((..))))))))))))))	((((((((((((((....))))))))))))))	((((((((((((((....))))))))))))))	((((((((((((((..))))))))))))))	((((((((((((((..))))))))))))))
2LC8	((((((((...((....)).))))))).)).....…(((((......)))))...	.............(((((((...((....(((.....)))).....)))))))))).	....................(((....)))......................	.((((((((((((((((..))))))).))))))))((((((((((..))))))))))	(((((((((((((.[[[[[[[))))((((..)))).)))))))))...]]]]]]].	.............((((((((((((....))))..............))))))).	.............((((((((((((....))))..............))))))).	..((.((((((((.....<<.)))).))))<)).......>........>>.....	((.((((((......<<<<<<)))))..)).(((.....)))........>>>>>>.
2LDL	((((((.(((.......))).))))))	((((((.((((.....)))).))))))	((((((.(((.......))).))))))	(((((((((((...)))))))))))	(((((((((((...))))))))))))	((((((.(((.......))).))))))	((((((.(((.......))).))))))	((((((.((((.....)))).))))))	((((((((((.....))))))))))
2LDT	(((((((...(((....)))....)))))))	(((((((..((((....))))...)))))))	(((((((..((((....))))...)))))))	(((((((((((((((..)))).))))))))))	(((((((((((((((..)))).))))))))))	(((((((..((((....))))...)))))))	(((((((..((((....))))...)))))))	(((((((..((((....))))...)))))))	(((((((..(((((..)))))...)))))))
2LHP	(((((((((.((((((....))))))..)))))))))	(((((((((.((((((....))))))..)))))))))	(((((((((.((((((....))))))..)))))))))	((((((((((((((((((..)))))))).))))))))))	((((((((((((((((((..))))))))).)))))))))	(((((((((.((((((....))))))..)))))))))	(((((((((.((((((....))))))..)))))))))	(((((((((.(((((((..)))))))..)))))))))	(((((((((.(((((((..)))))))..)))))))))
2LI4	((((((((((((((....))))))))))))))	((((((((((((((....))))))))))))))	((((((((((((((....))))))))))))))	((((((((((((((..))))))))))))))	((((((((((((((..))))))))))))))	((((((((((((((....))))))))))))))	((((((((((((((....))))))))))))))	((((((((((((((....))))))))))))))	((((((((((((((....))))))))))))))
2LJJ	(((((..(((((.....))))))))))	(((((..(((((.....))))))))))	(((((..(((((.....))))))))))	(((((..(((((.....))))))))))	(((((..(((((.....))))))))))	(((((..(((((.....))))))))))	(((((..(((((.....))))))))))	(((((..(((((.....))))))))))	(((((..(((((.....))))))))))

PDB	MXfold2	CONTRAfold	Centroidfold	Mcfold Default	Mcfold Pseudoknotted	Linearfold C	Linearfold V	Ufold	SPOT-RNA
2LK3	((((..(((((....)))))))))	((((..(((((....)))))))))	((((..(((((....)))))))))	((((..((((((..))))))))))	((((..((((((..))))))))))	((((..(((((....)))))))))	((((..(((((....)))))))))	((((..(((((....)))))))))	((((..((((((..))))))))))
2LKR	((((((((.(..((((((((.((((.(((.....))).)))).)))....((((((((((....))))))))))(((....)))...)))))..).).)))).))))	((((((((.....((((((((.((((.(((.....)))..)))).)))...((((((((((((....))))))))))((....))....)))))......)))).))))	..(..........((((.((((.....)))).................((((((((((((....)))))))))))............)))).............)..	((((((((((((((((((((.((((((((...))))).))))((((((((((((((((((..))))))))))))))))))))))..)))))).))))))).))))	(((((((((((((((((((.((((((((...))))).))))((((((((((((((((((..)))))))))))))))))))))..)))))).))))))).))))	.................((((.....))))..................((((((((((....))))))))))..	.................((((.....))))..................((((((((((....)))))))))))).	((((((((..(..(((((.((.(((.(.(((.....)))..))))).......((((((((((((..)))))))))))))))........))...)))))....).)))).))))	(((..(((..........((.(.....).)).................((((((((((..))))))))))((((..))))............)))...)))
2LP9	(((((......)))))	(((((......)))))	(((((......)))))	((((((...).)))))	((((((...).)))))	....	....	((((((...).)))))	((((((....))))))
2LPA	((((((...))))))	((((((...))))))	((((((...))))))	((((((...))))))	((((((...))))))	((((((...))))))	((((((...))))))	((((((...))))))	((((((...))))))
2LQZ	((((((...((((....))))))))))	((((((...((((....))))))))))	((((((...((((....))))))))))	((((((...(((((..)))))))))))	((((((...(((((..)))))))))))	((((((...((((....))))))))))	((((((...((((....))))))))))	((((((...(((((..)))))))))))	((((((...(((((..)))))))))))
2LU0	(((((..((((.((....))...(((((....)))))))))...)))))	(((((..((((.((....))...(((((....)))))))))...)))))	(((((..((((.((....))....((((....)))).))))...)))))	((((((((((((((..)))))..((((((..))))))))).)))))))	((((((((((((((..))))..((((((..))))))))))).))))))	(((((..((((...........(((((....)))))))))...)))))	(((((..((((...........(((((....)))))))))...)))))	(((((..((((.((....))...(((((((..)))))))))...)))))	(((((..((((...........((((((..)))))))))...)))))
2LUB	((((((((.((((((....))))))..)))))))))	((((((((.((((((....))))))..)))))))))	((((((((.((((((....))))))..)))))))))	((((((((((((((((..)))))))).))))))))	(((((((((((((((..)))))))).)))))))))	((((((((.((((((....))))))..)))))))))	((((((((.((((((....))))))..)))))))))	((((((((.(((((((..)))))))..)))))))))	((((((((.(((((((..)))))))..))))))))
2LUN	((((.(..((((....))))..).))))	((((.(..((((....))))..).))))	((((.....(((....))).....))))	((((((((((((..))))))))))))	(((((((((((((..)))))))))))))	((((.(..((((....))))..).))))	((((.(..((((....))))..).))))	((((.((.((((((..))))))).).)))	(((((..((((((..)))))..))))))
2LUP	(((((((((((((....)))))))))))))	((((((((((((((....))))))))))))))	(((((((((((((....)))))))))))))))	(((((((((((((....)))))))))))))))	(((((((((((((....))))))))))))))	(((((((((((((....))))))))))))))	(((((((((((((....))))))))))))))	(((((((((((((....))))))))))))))	(((((((((((((....))))))))))))))
2LV0	((((((((........))))))))	((((((((........))))))))	(((((((..((....)))))))))	(((((((((((..)))))))))))	(((((((((((..)))))))))))	((((((((........))))))))	((((((((........))))))))	((((((((.((..)).))))))))	((((((((..(...).))))))))
2M12	((((((..........))))))	((((((.(((....)))))))))	((((((..(....)...))))))	((((((.((((..))))))))))	((((((.((((..))))))))))	((((((..........))))))	((((((..........))))))	((((((.(((....)))))))))	((((((.(((....)))))))))
2M21	(((((((.......)))))))	(((((((.......)))))))	(((((((.......)))))))	(((((((((...)))))))))	(((((((((...)))))))))	(((((((.......)))))))	(((((((.......)))))))	(((((((.......)))))))	(((((((.......)))))))
2M22	(((((.((((...)))).)))))	(((((.(((.....))).)))))	(((((.(((.....))).)))))	((((((((((...))))))))))	((((((((((...))))))))))	(((((.(((.....))).)))))	(((((.(((.....))).)))))	(((((.((((...)))).)))))	((((((((((...))))))))))
2M24	(((((((((..........)))))))))	(((((((((.((......)))))))))))	(((((((((..........)))))))))	(((((((((.((((..)))))))))))))	(((((((((.((((..)))))))))))))	(((((((((..........)))))))))	(((((((((..........)))))))))	(((((((((.(.(...).).)))))))))	(((((((((.((......)))))))))))
2M4W	((((((....))).)))	((((((....))).)))	((((((....))).)))	(((((((..)))).)))	(((((((..)))).)))	....	....	((.(((....)))()))	((((((....))).)))
2M57	(((.((((((((((.....)))))..))))).)))	(((.((((((((((.....)))))..))))).)))	.((.(((((.((((.....))))...))))).)).	((((((((((((((.....)))))..))))))))	(((((((((((((((.....))))..)))))))))	(((.((((((((((.....)))))..)))).)))	(((.((((((((((.....)))))..)))).)))	(((.((((((((((.....))))).)))))).)))	((((((((((((((.....))))..))))))))))

PDB	MXfold2	CONTRAfold	Centroidfold	Mcfold Default	Mcfold Pseudoknotted	Linearfold C	Linearfold V	Ufold	SPOT-RNA
2M5U	((((((((....))))))))	((((((((....))))))))	((((((((....))))))))	(((((((((..)))))))))	(((((((((..)))))))))	((((((((....))))))))	((((((((....))))))))	((((((((....))))))))	((((((((....))))))))
2M8K	....	..........((((((((((.((((....)))...))))))))))	...........(((((((((.((((....))))...))))))))).	..........((((((((((((((((..))))).).))))))))))	(((((((..)))[[[[[[[..)))))........]]]]]]]...	..........((((((((((.((((....))))...))))))))))	..........((((((((((.((((....))))...))))))))))	<<<<<.....((((((((((((>>>>>.....).).)))))))))))	..........((((((((((.(............).))))))))))
2MEQ	((((((.......))))))	((((((.......))))))	((((((.......))))))	((((((((...))))))))	((((((((...))))))))	((((((.......))))))	((((((.......))))))	((((((.......))))))	(((((((....).))))))
2MF0	.(((........)))...((((((......)))))))......(((((.....)))))(((((.....))))))	((((........))))..((((((......))))))).......(((((.....)))))(((((...))))))	....................(((((......)))))............(....).................	(((((((..)))))))(((((((..(..))))))).))).((((((((((((..))))))...).))))))	(((((.[[[[[)))))((((((((..))))))))]]]]].((((((((((((..))))))...).))))))	((((........))))..((((((......))))))......(((((.....)))))((((.....)))))	((((........))))..((((((......))))))......(((((.....)))))((((.....)))))	((((........))))(.((((((......)))))..)...(((((.....)))))(((((.....)))))	((((........))))..((((((......)))))).......(((((.....)))))((((.....)))))
2MFC	(((((((........)))))))	(((((((........)))))))	(((((((........)))))))	((((((((((..))))))))))	((((((((((..))))))))))	(((((((........)))))))	(((((((........)))))))	(((((((........)))))))	(((((((........)))))))
2MFD	(((((((.....)))))))	(((((((.....)))))))	(((((((.....)))))))	((((((((...))))))))	((((((((...))))))))	(((((((.....)))))))	(((((((.....)))))))	(((((((.....)))))))	(((((((.....)))))))
2MFE	((((((((......))))))))	((((((((......))))))))	((((((((......))))))))	((((((((..(..)))))))))	((((((((..(..)))))))))	((((((((......))))))))	((((((((......))))))))	((((((((..(..)))))))))	((((((((......))))))))
2MFF	((((((((.....))))))))	((((((((.....))))))))	((((((((.....))))))))	(((((((((...)))))))))	(((((((((...)))))))))	((((((((.....))))))))	((((((((.....))))))))	((((((((.....))))))))	((((((((.....))))))))
2MFG	((((((((.....))))))))	((((((((.....))))))))	((((((((.....))))))))	((((((((.(..)))))))))	((((((((.(..)))))))))	((((((((.....))))))))	((((((((.....))))))))	((((((((.....))))))))	((((((((.....))))))))
2MHI	(((((((.(((((.((((...))))))))).((((.....))))..)))))))	((((((((.((((((.....)))))).))..((((.....))))...))))))	((((((..(((((.((((...)))))))).((((.....))))...))))))	(((((((((((((.((((...))))))))).(((((...)))))).)))))))	(((((((((((((.((((...))))))))).(((((...)))))).)))))))	((((((((.((((((.....)))))).)..((((.....))))...))))))	((((((((.((((((.....)))))).)..((((.....))))...))))))	((((((((((((((.(((...))))))))).(((((..).))))).))))))	((((((..(((((.((((...))))))))(((((.....)))))..))))))
2MNC	((((.((...))))))	((((........))))	((((........))))	((((.((...))))))	((((.((...))))))	((((........))))	((((........))))	((((.(...)..))))	((((........))))
2MQT	(((((.((((((((...((((((..(((..((((....)))).)))..))))))))))))).)))))	(((((.((((((((...((((((..((((.((((....))))))))..)))))))))))))).)))))	((((((.((((((.....((((((..((((.((((....))))))))..))))))..)))))).)))))	(((((((((((((...((((((((((((.(((((..)))))))))))))))))))))))))))))))	(((((((((((((...(((((((((((.(((((..)))))))))))))))))))))))))))))))	(((((.((((((((...((((((..(((.((((....))))))))..)))))))))))))).)))))	(((((.((((((((...((((((..(((.((((....))))))))..)))))))))))))).)))))	(((((.((((((((....(((((..((((.(((((..)))))))))..))))).))))))).)))))	(((((((((((((...(((((((((((.(((((..))))))))))))))))))))))))))))))
2MS0	(((((((..(((.........)))((((((.......))))))....(((((.......)))))))))))	((((((((.......((((....(((((((.......))))))))))((((.......))))))))))))	(((((((..................(((((.......)))))......(((((.......))))))))))))	((((((((((....(((((....((((((((...)))))))))))))((((((..)))))))))))))	((((((((((....(((((...(((((((((...)))))))))))))(((((..)))))))))))))))	((((((((......(((((....(((((((.......)))))))))))(((......))).))))))))	((((((((......(((((....(((((((.......)))))))))))(((......))).))))))))	(((((((..(((.........)))((((((.......))))))....((((.......)))))))))))	(((((((<.((((><..{..))))((((((.......))))))...>((((((.}.)..)))))))))))
2MTJ	(((((..(((((....))))).((((((....))))))).....))))).	(((((((((((((....))))).(((((....)))))).))..))))).	(((((..(((((....))))).(((((...)))))).....))))).	((((((((((((((..))))))((((((((.)))))))))..))))).	((((((((((((((..))))))((((((((..))))))))))..)))))).	(((((..(((((....))))).((((((...)))))).....))))).	(((((..(((((....))))).((((((...)))))).....))))).	(((((..((((((..)))))).(((((....)))))).....))))).	(((((..((((((..)))))).((((((.))))))).....))))).
2MXJ	((......)).	....	....	((((..)))).	((((..)))).	....	....	((......)).	(((....))).

PDB	MXfold2	CONTRAfold	Centroidfold	Mcfold Default	Mcfold Pseudoknotted	Linearfold C	Linearfold V	Ufold	SPOT-RNA
2MXL	..(((((((..(((.((......))..)))..))))))))	..(((((((..((((((......)).)))))..)))))))	...((((((..((((((......)).))))..)))))).	..(((((((((((((((((...)))))))))))))))))	..(((((((((((((((((...)))))))))))))))))	..(((((((..(((.((......))..)))..)))))))	..(((((((..(((.((......))..)))..)))))))	..(((((((..((((((......)).))))..)))))))	..(((((((((((.((((....))).))))).)))))))
2N0R	(...((((....))))......)	....((((....))))).......	....((((....))))).......	(((((((((..)))))))))...	(((((((((..)))))))))...	....((((....)))).......	....((((....)))).......	(...((((....))))...)...	...((((((..))))))......
2N1Q	((((((((((.........(((((((....)))))))).....(((((...((....((((.(((((((....)))))))...))))..))(((((((....))).)))).........(((((....)))))...)))))))))))))))).	((((((((((.........(((((((....))))))).....((((((.(((....((((.((((((....)))))))...))))..))).)...(((((....))))).........(((((....)))))...)))))))))))))))).	((((((((((.........(((((((...)))))))).....(((((.........(((.(((((((....)))))))...)))).....(.....).(((......)))...........(((((....)))))...))))))))))))))).	(((((((((((((((((((((((((((..))))))))))))))))((((((((((((((((((((((((..))))))))))..)))))).))))))(((((....)))))....((((..(((((....))))))))))))))))))))))).	(((((((((((((((((((((((((((((..)))))))))))))((((((((((((((((((((((((..)))))))))..)))))).))))))(((((....)))))).....(((..(((((....))))))))))))))))))))))))))).	((((((((((..........(((((((...)))))))....((((((....(((((((((.(((((((....)))))))...)))).((((((.........)).)))).....))))).(((((....)))))...)))))))))))))))).	......((((..........(((((((....)))))))..........))))..........(((((((....)))))))	((((((((((..(.((((.((((((((....)))))))...(((..(((.(((....((((.(((((((....)))))))...)))..))).)))..))...))))..).....(.((..(((((.....)))))..)))...))))))))))).	((((((((((..........((((((((..))))))))......((((.........((((.((((((((..))))))))...))))).....(...............)..........((((((..))))))......))))))))))))))).
2N2O	((.(((((((...))))))))))).	...(((((((...)))))))...	.........((((....))))..	(((.((((((...)))))))))).	(((.((((((...)))))))))).	....	....	((.(((((((...))))))))))).	...(((((((...)))))))...
2N2P	((.(((((((...))))))))))).	...(((((((...)))))))...	....((((((...))))))....	(((.((((((...)))))))))).	(((.((((((...)))))))))).	....((((((...))))))....	....((((((...))))))....	((.(((((((...))))))))))).	...(((((((...)))))))...
2N3O	(((((((((.....)))))))))	(((((((((.....)))))))))	(((((((((.....)))))))))	((((((((((...))))))))))	((((((((((...))))))))))	(((((((((.....)))))))))	(((((((((.....)))))))))	(((((((((.....)))))))))	(((((((((.....)))))))))
2N3Q	(((((.(((..((((((....))).)))(((..((((....)))))))).....)))))))).	(((((.(((..((((((....))).))).((..(((((.......))))).)))))))))).	(((((.(....)................((...((((....)))).)).........))))).	((((((((((..))))(((((((((((((((((..)))))))))))))))))).)))))).	((((((((((..))))((((((((((((((((..))))))))))))))))).)))))).	....................(((((.((((..(((((.......))))).))))..)))))	....................(((((.(((((..(((((.......))))).))))..)))))	(((((.(((..((((((..)))).)))(((..(((((..)))))))).(..))))))))).	(((((.(((..(((((((..)))).))).((..(((...........))).)))))))))).
2N4L	(((((((((((((..((((((......))...))))..))))))))))))))	(((((((((((((..(((..((((.....)))))))..))))))))))))))	(((((((((((((..(((..(((.......))))))..))))))))))))))	((((((((((((((((((..(((((...))))))))))))))))))))))))))	((((((((((((((((((..(((((...)))))))))))))))))))))))))))	(((((((((((((..(((((...........)))))..))))))))))))))	(((((((((((((..(((((...........)))))..))))))))))))))	((((((((((((((.(((..(((.....)))))))).)))))))))))))	(((((((((((((..((..(((((.....)))))))..))))))))))))))
2N6S	((((((((((((((((...)).))))))))))))))	((((((((((((((.(.....).)))))))))))))	((((((((((((((.(.....).)))))))))))))	(((((((((((((((((..))))))))))))))))	(((((((((((((((((..))))))))))))))))))	((((((((((((((........))))))))))))))	((((((((((((((........))))))))))))))	((((((((((((((.((..)).))))))))))))))	((((((((((((((((...)).)))))))))))))
2N6W	((((((((((((((....((((((((..((((....))))...))))))))...))))))))))))))	((((((((((((((.((.((((((((..(((.....))))...))))))))).))))))))))))))	((((((((((((((.((..(((((((..((((....))))...)))))).)).)))))))))))))))	((((((((((((((((((.((((((((((((((..)))))).)))))))))))))))))))))))))	(((((((((((((((((((.((((((((((((((..)))))).)))))))))))))))))))))))))))))))	((((((((((((((....((((((((..((((....))))...))))))))...)))))))))))))))	((((((((((((((....((((((((..((((....))))...))))))))...)))))))))))))))	((((((((((((((..((.((((((((..(((((..)))))...)))))))..)))))))))))))))	((((((((((((((((((.((((((((((((((..)))))..)))))))))))))))))))))))))
2N6T	((((.((((((((..((((....))))...))))))))))))	((((.((((((((..((((....))))...))))))))))))	(((((.(((((((..((((....))))...)))))))))))))	(((((.((((((((((((((..)))))).))))))))))))))	(((((.((((((((((((((..)))))).))))))))))))))	.((..((((((((..((((....))))...))))))))..))	.((..((((((((..((((....))))...))))))))..))	((((.((((((((..(((((..))))))...))))))))))))	(((((.(((((((..(((((..)))))...))))))))))))

PDB	MXfold2	CONTRAfold	Centroidfold	Mcfold Default	Mcfold Pseudoknotted	Linearfold C	Linearfold V	Ufold	SPOT-RNA
2N6X	((((((((.((.((((((((....)))))))))).))))))))	((((((((.((.((((((((....))))))))))).))))))))	((((((((.((..(((((((....)))))))).)).))))))))	((((((((((((.((((((((..))))))))))))))))))))	((((((((((((.((((((((..))))))))))))))))))))	((((((((....((((((((....))))))))...))))))))	((((((((....((((((((....))))))))...))))))))	((((((((.((.(((((((((..)))))))))).))))))))	((((((((....(((((((((..))))))))))...))))))))
2N7M	((((((((((.(((((..(((((.(((.....(((....)))..))))))))...(((((((....))))))).))))).)))))))))))	((((((((((.((((((.(((((.(((.....(((....)))..)))))))).)((((((((....))))))))))))).)))))))))))	.(((((((((.((((................(((....)))............(((((((....))))))..)))).)))))))))).	(((((((((((((((((((((((.(((...((((((..))))))))))))))))))((((((((..))))))))..)))))))))))))))))	((((((((((((((((((((((((.(((...((((((..))))))))))))))))(((((((..))))))))..)))))))))))))))))	((((((((((.(((((..(((((.(((.....(((....)))..)))))))))..((((((((....))))))))))))).)))))))))))	...........(((((..(((((.(((.....(((....)))..))))))))..((((((((....))))))))))))	((((((((((.(((((..(((((.(((..(..((((..)))).)))))))))..((((((((....))))))))))))).)))))))))))	((((((((((.(((((........(((....(((((..))))).)))........(((((((....))))))).))))).)))))))))))
2N7X	(((((............)))))	(((((((.......))..)))))	(((((............)))))	((((((((((...))))))))))	((((((((((...))))))))))	(((((............)))))	(((((............)))))	(((((..(((...)))..)))))	(((((............)))))
2N82	(((((............)))))	(((((((.......))..)))))	(((((............)))))	((((((((((...))))))))))	((((((((((...))))))))))	(((((............)))))	(((((............)))))	(((((..(((...)))..)))))	(((((............)))))
2N8V	(((((.((((((((....))).))))).((((((((......)))))))))))))..............	(((((.((((((((....)).)))))).((((((((......)))))))))))))...............	(((((.(((((((......).)))))).((((((((......)))))))))))))..............	.(.((((((((((((..))).))))))))))((((((((....((((((....))))))))))))))...	((((((((((((.[[[[[[[[)))))))((((((((....)))))))))))))).......]]]]]]]]	(((((.((((((.........)))))).((((((((......)))))))))))))..............	(((((.((((((.........)))))).((((((((......)))))))))))))..............	(((((.(((((((<<<<<<).)))))).((((((((......))))))))))))).........>>>>>>	(((((.(((((((((..))).)))))).(((((((<<....)))))))))))))....>>........
2NBX	((((((...((((((((((.((...(((((...)))))..))..))))))(((((((.(((((((.....)))))))....)))))))......))))).))))))	((((((...((((((((((((((..(((((...)))))))))..))))))((((((((((((((.....)))))))...)))))))).......))))).))))))	((((((....((((((((((((..((((.....))))))))..))))))((((((((((((((.....)))))))...))))))))......))))..))))))			((((((...((((((((((((((..((((.....))))))))..))))))((((((((((((((.....)))))))...))))))))......))))).))))))	(((((.........((((((((((..((((.....))))))))..)))))))))))...(((((((.....)))))))..	((((((...((((((((((.(((<.((((((...))))))>))..))))))((((((((((((((.....)))))))...))))))))(.....))))))).))))))	((((((........((((((......(((((...)))))......))))))((((((((((((((...))))))))...))))))))............))))))
2NBY	(((((((((((..(((((...)))))))))))..))))))	(((((((((((..(((((...)))))))))))..)))))))	(((((((((((..((((.....))))))))..)))))))	(((((((((((((((((((...))))))))))))))))))))	(((((((((((((((((((...)))))))))))))))))))	(((((((((((..((((.....))))))))..)))))))	(((((((((((..((((.....))))))))..)))))))	(((((((.((.(.(((((...)))))).)))..)))))))	(((((((......(((((...)))))......)))))))
2NBZ	((((((((.(((((((.....)))))))....)))))))))	((((((((((((((((.....))))))))...)))))))))	((((((((((((((((.....)))))))...)))))))))	((((((((((((((((((...))))))))))...)))))))))	(((((((((((((((((...))))))))...)))))))))	((((((((((((((((.....)))))))...)))))))))	((((((((((((((((.....)))))))...)))))))))	((((((((((((((((.....)))))))...)))))))))	((((((((((((((((.....))))))))...)))))))))
2NC0	(((((...(((((....))))).)))))	(((((...(((((....))))).)))))	(((((...(((((....))))).)))))	((((((..((((((..))))))))))))	((((((..((((((..))))))))))))	(((((...(((((....))))).)))))	(((((...(((((....))))).)))))	(((((...(((((....))))).)))))	(((((...((((((..)))))).)))))
2NC1	((((((...((((((((((....))))))((((((((....)))))))......))))).))))))	((((((...((((((((((....))))))(((((((....)))))))......))))).))))))	((((((....(((((((((....))))))(((((((....)))))))......)))..))))))	((((((((((((((((((((..)))))))(((((((..))))))))...))))))))..))))))	((((((((((((((((((((..)))))))((((((((..))))))))...))))))))..))))))	((((((...((((((((((....))))))((((((....)))))))......)))).))))))	((((((...((((((((((....))))))((((((....)))))))......))))).))))))	((((((...((((((((((....)))))))((((((....)))))))........))))).))))))	((((((........(((((((..)))))))((((((((..))))))))............))))))
2NCI	((((((....((((....))))))))))	((((((....((((....))))))))))	((((((....((((....))))))))))	((((((....(((((..)))))))))))	((((((....(((((..)))))))))))	((((((....((((....))))))))))	((((((....((((....))))))))))	((((((....(((((..)))))))))))	((((((....(((((..)))))))))))

PDB	MXfold2	CONTRAfold	Centroidfold	Mcfold Default	Mcfold Pseudoknotted	Linearfold C	Linearfold V	Ufold	SPOT-RNA
2O33	(((..(((....)))..)))	(((..(((....)))..)))	(((..(((....)))..)))	((((((((....))))))))	((((((((....))))))))	(((..(((....)))..)))	(((..(((....)))..)))	(((..(((....)))..)))	(((((((((..)))))))))
2OJ7	((....))	....	....	(((..)))	(((..)))	....	....	((....))	((....))
2PCV	(((.((((.(.((((....)))).)...)))))<br>))	(((((....((((((....)))).....)))))<br>)))	((((...........................)<br>)))	((((((((((((((((..)))))))))))<br>).)))))	((((((((((((((((..)))<br>))))))))).)))))	((((((.....((((....))))......)<br>.)))))	((((((.....((((....))))......)<br>.)))))	((((.(((...((((....))))..<br>...)))))))	(((((....(.(((((..)))))......)<br>)))))
2PJP	((((((.(((....)))))))))	((((((.(((((())))))))))))	(((((..(((....))).)))))	((((((.((((..))))))))))	((((((.((((..))))))))<br>))	((((((.(((....)))))))))	((((((.(((....)))))))))	((((((.(((((()))))))))))))	((((((.((((..))))))))))
2PXB	((((((((((....(((....(((....)))...<br>.))).))))))))))	((((((((((....(((....(((....))).<br>...))).))))))))))	(((((((((............((....))<br>..........)))))))))	((.(((((((((((..)))))(((..))<br>))))))))).(((...)))	((.(((((((((((..))))<br>)(((..))))))))))).((<br>(...)))	((((((((((....(((....(((....)<br>))....))).))))))))))	((((((((((....(((....(((....)<br>))....))).))))))))))	((((((((((....(((....(((.<br>...)))....))).))))))))))	((((((((((...(((((.((((((..))<br>)))).))))))))))))))
2PXD	((((((((((....(((....(((....)))...<br>.))).))))))))))	((((((((((....(((....(((....))).<br>...))).))))))))))	(((((((....((......)).((....))<br>............)))))))	(((((((((((((((..)))))(((..))<br>))))).(((..)))))))))	((((((((((((((..))))<br>).[[[[[[)))))))))..<br>]]]]]]]	((((((((((....(((....(((....)<br>))....))).))))))))))	((((((((((....(((....(((....)<br>))....))).))))))))))	((((((((((.<<.(((..>>(<br>((....)))....))).))))))))<br>))	((((((((((...(((((.((((((..))<br>)))).))))))))))))))
2PXE	((((((((((....(((....(((....)))...<br>.))).))))))))))	((((((((((....(((....(((....))).<br>...))).))))))))))	(((((((((............((....))<br>..........)))))))))	(((((((((((((((..)))))(((..))<br>))))).(((..)))))))))	((((((((((((((..))))<br>)(((..))))))).(((..)))<br>))))))	((((((((((....(((....(((....)<br>))....))).))))))))))	((((((((((....(((....(((....)<br>))....))).))))))))))	((((((((((....(((....(((.<br>...)))....))).))))))))))	((((((((((...(((((.((((((..))<br>)))).))))))))))))))
2PXF	((((((((((....(((....(((....)))...<br>.))).))))))))))	((((((((((....(((....(((....))).<br>...))).))))))))))	(((((((((...........(((....))<br>)........)))))))))	(((((((((((((((..)))))(((..))<br>))))).(((..)))))))))	((((((((((((((..))))<br>)(((..))))))).(((..)))<br>))))))	((((((((((....(((....(((....)<br>))....))).))))))))))	((((((((((....(((....(((....)<br>))....))).))))))))))	((((((((((.<<.(((..>>(<br>((....)))....))).))))))))<br>))	((((((((((....((((.((((((..))<br>))))).))).))))))))))
2PXK	((((((((((....(((....(((....)))...<br>.))).))))))))))	((((((((((....(((....(((....))).<br>...))).))))))))))	(((((((((...........(((....))<br>)........)))))))))	(((((((((((((((..)))))(((..))<br>))))).(((..)))))))))	((((((((((((((..))))<br>)(((..))))))).(((..)))<br>))))))	((((((((((....(((....(((....)<br>))....))).))))))))))	((((((((((....(((....(((....)<br>))....))).))))))))))	((((((((((....(((....(((.<br>...)))....))).))))))))))	((((((((((...(((((.((((((..))<br>))))).)))))))))))))))
2PXL	...(((((((....(((....(((....)))....<br>))).))))))).	..((((((((....(((....(((....)))..<br>..))).))))))))	...................(((....))).<br>...............	..(((((((((((((..)))))(((..))<br>)))))))))(((...)))	..(((((((((((((..)))))<br>(((..)))))))))))(((...<br>)))	..((((((((....(((....(((....)<br>))....))).))))))))	..((((((((....(((....(((....)<br>))....))).))))))))	..((((((((....(((....(((..<br>..)))....))).))))))))	..........((.((((..(((((..))))<br>).).)))..)).....
2PXP	((((((((((....(((....(((....)))...<br>.))).))))))))))	((((((((((....(((....(((....))).<br>...))).))))))))))	(((((((((..(........).((....))<br>..........)))))))))	(((((((((((((((..)))))(((..))<br>))))).(((..)))))))))	((((((((((((((..))))<br>)(((..))))))).(((..)))<br>))))))	((((((((((....(((....(((....)<br>))....))).))))))))))	((((((((((....(((....(((....)<br>))....))).))))))))))	((((((((((.<<.(((..>>(<br>((....)))....))).))))))))<br>))	((((((((((...(((((.((((((..))<br>)))).))))))))))))))
2PXQ	((((((((((....(((....(((....)))...<br>.))).))))))))))	((((((((((....(((....(((....))).<br>...))).))))))))))	(((((((((............((....))<br>..........)))))))))	(((((((((((((((..)))))(((..))<br>))))).(((..)))))))))	((((((((((((((..))))<br>)(((..))))))).(((..)))<br>))))))	((((((((((....(((....(((....)<br>))....))).))))))))))	((((((((((....(((....(((....)<br>))....))).))))))))))	((((((((((....(((....(((.<br>...)))....))).))))))))))	((((((((((...((((((((((((..))<br>))))))))))))))))))))
2PXT	((((((((((....(((....(((....)))...<br>.))).))))))))))	((((((((((....(((....(((....))).<br>...))).))))))))))	(((((((((...........(((....))<br>)........)))))))))	(((((((((((((((..)))))(((..))<br>))))).(((..)))))))))	((((((((((((((..))))<br>)(((..))))))).(((..)))<br>))))))	((((((((((....(((....(((....)<br>))....))).))))))))))	((((((((((....(((....(((....)<br>))....))).))))))))))	((((((((((.<<.(((..>>(<br>((....)))....))).))))))))<br>))	((((((((((...(((((.((((((..))<br>)))).))))))))))))))
2PXU	((((((((((....(((....(((....)))...<br>.))).))))))))))	((((((((((....(((....(((....))).<br>...))).))))))))))	(((((((((...........(((....))<br>)........)))))))))	(((((((((((((((..)))))(((..))<br>))))).(((..)))))))))	((((((((((((((..))))<br>)(((..))))))).(((..)))<br>))))))	((((((((((....(((....(((....)<br>))....))).))))))))))	((((((((((....(((....(((....)<br>))....))).))))))))))	((((((((((.<<.(((..>>(<br>((....)))....))).))))))))<br>))	((((((((((...(((((.((((((..))<br>)))).))))))))))))))

PDB	MXfold2	CONTRAfold	Centroidfold	Mcfold Default	Mcfold Pseudoknotted	Linearfold C	Linearfold V	Ufold	SPOT-RNA
2PXV	((((((((((....(((....(((....)))....))).))))))))))	((((((((((....(((....(((....))).…))).))))))))))	((((((((((............(((....)))..........)))))))))	(((((((((((((((..)))))(((..)))))).(((..)))))))))	(((((((((((((((..)))))(((..))))))).(((..)))))))))	((((((((((....(((....(((....)))....))).))))))))))	((((((((((....(((....(((....)))....))).))))))))))	((((((((((....(((....(((....)))....))).))))))))))	((((((((((…(((((.((((((..))))))).))))))))))))))
2QH2	((((((((........))))))))	((((((((........))))))))	((((((((........))))))))	((((((((((((..))))))))))))	((((((((((((..))))))))))))	((((((((........))))))))	((((((((........))))))))	(((((((((.(..)).))))))))	((((((((........))))))))
2QH3	((((((((.......))))))))	((((((((.......))))))))	((((((((.......))))))))	(((((((((((...)))))))))))	(((((((((((...)))))))))))	((((((((.......))))))))	((((((((.......))))))))	((((((((.(...).))))))))	((((((((.......))))))))
2QH4	((((((......))))))	((((((......))))))	((((((......))))))	((((((((..))))))))	((((((((..))))))))	((((((......))))))	((((((......))))))	((((((......))))))	((((((......))))))
2R8S	.((((((((.(....(((.(.....((((.(((((((..(((((((((....)))))))))).(((((....)))))..)))......))))))))......).)))....).)......((((...(((((((((...))))))))))..))))))))))).	.((((((((.....(((((((....(((.((((((((.(((((((((....))))))))).(((((....))))).))))......)))))))...))))))....))))......((((.(.((((((((...)))))))))).)))))...)))).	.(((((((....................(..(((..((...((((((((....)))))))))..(((((....)))))..))........))).)...............................((((((((...)))))))))......)))))).	((((((((..((((((.........))))))(.(((((((((((((((..)))))))))))((((((....))))).))))))...((((.(((((...))))......)))))...((((.(((((((((((...))))))))))))))))))))))	((..((((((((..(((((((..(((((..(.((((((((((((((((..))))))))))(((((....))))).))))))..[[[[)))).).)))))))).))))))))..((((.(((((((((((...)))))))))))))).]]]]]..	.....((((.....(((((((....(((.(((((((((.((((((((((....)))))))))).(((((....))))).))))....))))))))...)))))))....))))......((((...(((((((((...))))))))))..)))).........	....((((((((((....)))))))))).((((((....)))))....	.((((.(((.(...(((((((....((((..(((((((.((((((((((...))))))))))..((((....))))..)))))......)).))))...))))))..).)))......((((.(.((((((((((...))))))))))).))))...)))).	((((((((......................................((((((((((..)))))))))))...(((.............))).((((...(((((((((...))))))))))..))))))))))))
2RLU	((((((((...))))))))	(((((((((.)))))))))	(((((((.....)))))))	((((((((...))))))))	((((((((...))))))))	(((((((.....)))))))	(((((((.....)))))))	((((((((...))))))))	(((((((.....)))))))
2RO2	(((((((((.....)))))))))	((((((((((...))))))))))	((((((((.......))))))))	((((((((((...))))))))))	((((((((((...))))))))))	((((((((.......))))))))	((((((((.......))))))))	((((((((((...))))))))))	(((((((((.....)))))))))
2RPK	(((((((......)))))))	(((((((......)))))))	(((((((......)))))))	(((((((.(...))))))))	(((((((.(...))))))))	(((((((......)))))))	(((((((......)))))))	(((((((......)))))))	(((((((......)))))))
2RPT	((((.(((....))).))))	((((.(((....))).))))	((((.(((....))).))))	(((((((((..)))))))))	(((((((((..)))))))))	((((.(((....))).))))	((((.(((....))).))))	((((.(((....))).))))	(((((((((..)))))))))
2RVO	((((((((...(((((....))))).))))))))	((((((((...(((((....))))).))))))))	((((((((....(((......)))..))))))))	(((((((((((..((((..)))))))))))))))	(((((((((((..((((..)))))))))))))))	((((((((...(((((....))))).))))))))	((((((((...(((((....))))).))))))))	((((((((.(..((((....))))).))))))))	(((((((((..((((((..))))))))))))))
2TPK	........(((((((.(.........).))))))))..	........((((((((((.....))).)))))))..	........(((((((.(.........).)))))))..	((((((((((((((((..)))))))))))))..)))..	((((((((((((((((..))))))))))))..)))..	........((((((((((.....))).)))))))..	........((((((((((.....))).)))))))..	..<<<<<.(((((((>>>>>.......)))))))..	..<<<<<((((((((>>>>>.......)))))))).
2UWM	(((((((((((...)))))))))))	((((((((((((.))))))))))))	(((((((((.....)))))))))	(((((((((((...)))))))))))	(((((((((((...)))))))))))	(((((((((.....)))))))))	(((((((((.....)))))))))	((((((((((((.))))))))))))	(((((((((.....)))))))))
2V3C	((((..(((((.((((((((((....))))))))))..))))).....((((((.....(((.....((((....))).....)))..)))))).))))).	((((..(((((.((((((((((....))))))))..)))))....((((((..(..((((.....(((....))).....)))).)))))).))))).	((((..(((((.((((((((((....))))))))..)))))....((((((...(.........(((....)))........)..)))))).)))).			......(((((.((((((((((....)))))))))..)))))....((((((.....(((.....(((....))).....)))..)))))).......	(((((.(((((.((((((((((....))))))))..)))))......))))).......(((....)))......	...((<(((((.((((((((((....))))))))..))))).))>((((((...(..((.....(((....))).....)))..))))))......	.......((<(.((((((((((..))))))))..).)).....(((((((..>.(((((((((((..))))))))))))).))))))......

PDB	MXfold2	CONTRAfold	Centroidfold	Mcfold Default	Mcfold Pseudoknotted	Linearfold C	Linearfold V	Ufold	SPOT-RNA
2VPL	(((((((((..((((((((....)))).)))).....))))))))).	(((((((((..((((((((....))))).)).......))))))))).	(((((((((..(((.(((......)))..)))......))))))))).	(((((((((((((((((((..))))))).))))....))))))))).	(((((((((((((((((((..))))))).))))....)))))))))).	(((((((((..((((((((....))))).)))......))))))))).	(((((((((..((((((((....))))).)))......))))))))).	(((((((((..(((((((((..)))))).)))......))))))))).	(((((((((((((((((((..))))).)))))....))))))))).
2W2H	(((((((((....)))))))))	(((((((((....)))))))))	(((((((((....)))))))))	(((((((((....)))))))))	(((((((((....)))))))))	(((((((((....)))))))))	(((((((((....)))))))))	(((((((((....)))))))))	((((((((......))))))))
2XXA	((((((((((.((((((((.((((((((((((.....((((....((((....)))....))))...))).)).))))))..)).))))))...))))))))))	(((((.((((..(......((((....)))........).)))).((((((((((((.(((.(((((....)))))..)..)).)))))))))))))))))))	((((((((((.(((..........(.(((((............((((....))).(((...........)))......))))))...)))...)))))))))	(((((((((((..((.((((((.(..(((((((..(((((....)))))((((((((..((....)))))))))))))))))))))))))))))))))))))	(((((((((((..((.((((((.(..(((((((..(((((....)))))(((((((((..((....)))))))))))))))))))))))))))))))))))))	((((((((((.((((((...((((((..(((.....((((....((((....)))....))))...)))....))))))......))))))...))))))))))	..............(((((.(((....(((.....((((....((((....)))....))))...))).))).)))))..	((((((((((.((((((((.((((((((((((.....((((....(((....)))....))))...))).)).))))))..)).))))))...))))))))))	((((((((((...........((....((((((....(((((..(((((.<)))))<<)))))..)))))......)).......>>.....>))))))))))
2Y8W	((((((((....)))))))..	((((((((((()))))))))..	.((((((....))))))...	((((((((..))))))))..	((((((((..))))))))..	((((((((....)))))))..	((((((((....)))))))..	((((((((....)))))))..	((((((((....)))))))..
2Y8Y	((((((((....)))))))).	((((((((((())))))))))).	.((((((....))))))..	((((((((..)))))))).	((((((((..)))))))).	((((((((....)))))))).	((((((((....)))))))).	((((((((....)))))))).	((((((((....)))))))).
2Y95	((((((....))))))	((((((....))))))	((((((....))))))	((((((....))))))	((((((....))))))	((((((....))))))	((((((....))))))	((((((....))))))	((((((....))))))
2Y9H	((((((((....))))))))	((((((((((()))))))))))	.((((((....)))))).	((((((((..))))))))	((((((((..))))))))	((((((((....))))))))	((((((((....))))))))	((((((((....))))))))	((((((((....))))))))
2ZM5	(((((((..((((........)))).((((((.....)))))))......(((((.......)))))))))))))..	((((((((...((((((((.((.((((((((((...))))))).))).)).....))))))..))))))))))..	((((((...((((........)))).((((((.(.....))))))......(((((.......))))).))))))..	(((((((((((((((((..))))))))((((((((...)))))))))))))).((((((((....)))))))))))..	..((((((.[[[[))))))..]]]].((((((((...))))))))((((((.((((((....))))))))))))))	(((((((..((((........)))).((((((((...)))))))......((((((.......)))))))))))))..	(((((((..((((........)))).(((((((...)))))))......((((((.......)))))))))))))..	(((((((..((((........)))).((((((.......)))))......(((((.......)))))))))))))..	(((((((<.((((><..{[..)))))(((((((.....))))))))...>((((((}].)..)))))))))))))..
2ZNI	(((((((.((((.....)))).((((((.......))))))...(((((.......)))))))))))))....	(((((((.((((.....)))).((((((.......))))))...(((((.......))))))))))))....	((((((..((((.....)))).((((((.......))))))...(((((.......))))).)))))))....	((((((((((((...)))))((((((((((...))))))))).((((((((...))))))))))))).)).	(((((((((((((...))))))[[[[[))))))))..]]]]].((((((((((((((....)))))))))))))	(((((((.((((.....)))).((((((.......))))))...(((((.......))))))))))))....	(((((((.((((.....)))).((((((.......))))))...(((((.......))))))))))))....	(((((((.(((.......))).((((((.......))))))...(((((.......)))))))))))))....	(((((((.((((.....))))((((((((.....))))))))..((((((...)..)))))))))))....
2ZUE	(((((((..((((..........)))).((((((......)))))......(((((.......)))))))))))).	((((((((.((((.(((.......))).(((((.......)))))))))..((((.......)))))))))))).	((((((((.....................(((((.......)))))......((((.......)))))))))))).	(((((((..((((((((((...)))))))((((((...)))))))))))).((((((...)..))))))))))).	((((((((((((((((.(((((.[[[[[[[))))))))))))))))))))))))(((((...)..))))]]]]].]]].	(((((((..((((.(((.......))).((((.......)))))))))).(((((.......))))))))))).	(((((((..((((.(((.......))).(((((.......)))))))))).((((((......))))))))))).	(((((((..((((..........)))).(((((.......)))))......(((((.......))))))))))))).	(((((((..(((((........)))))((((((.......))))))....((((((...)..))))))))))))).
2ZZM	(((((((..(((.............)))(((((((.......))))))((.....))....(((((.......))))))))))))	(((((((((.....(((((((.......)))))))(..))))))..........(((...(((((.......))))))))))))	(((((((......................(((((.......)))))..............(((((.......))))))))))))			....(((((.....((((((((.......))))))))....))))).......((((((((..(((((.......)))))))))))))	((((((((.......(((((((...((.(((((((.......)))))).))...))....))))).......))))))))..	(((((((..(((..(..<<<.).<<)))((((((.(...).))))))(((....)))>>.(((((.>>>...))))))))))))	(((((((..((((((.........))))))((((((.......))))))............((((((...)..))))))))))))
2ZZN	(((((((..((((.......)))).((((((.......)))))......(((((.......))))))))))))))))	(((((((..((((((...)))))).((((((((())).)))))......(((((.......))))))))))))	(((((((..(((((.....))))).(((((.......))))).....(((((.......))))))))))))	((((((((((((((..)))))))((((((((((((..)).)))).)))))((((((...)..))))))))))))	((((((((((((((..))))))(((((((((((..)).)))).)))))((((((...)..))))))))))))	(((((((..((((((...)))))).(((((.......))))).....(((((.......))))))))))))	(((((((..((((((...)))))).(((((.......)))))......(((((.......))))))))))))	(((((((..((((.......)))).(((((.......))))).....(((((.......))))))))))))	(((((((..(((((<....)))))((((((.......))))))...>((((((...)..))))))))))))

PDB	MXfold2	CONTRAfold	Centroidfold	Mcfold Default	Mcfold Pseudoknotted	Linearfold C	Linearfold V	Ufold	SPOT-RNA
3A2K	(((((((..((((.........)))).(((((.......)))))......(((((.......)))))))))))....	(((((((..(((..(.((((((((((.....))))).))))))).).))).(((((.......))))))))))))....	(((((((.......................................(((((.......))))))))))))....	(((((((..((((((((((((((((((...))))).))))))))))))))(((((((...)..))))))))))))....	(((((((..((((((((((((((((((...))))).)))))))))))))(((((((...)..)))))))))))))....	(((((((..(((..(.((((((((((.....)))).))))))).).))).(((((.......))))))))))))....	(((((((..(((..(.((((((((((.....)))).))))))).).))).(((((.......))))))))))))....	(((((((..((((.........)))).(((((.......)))))......(((((.......))))))))))))....	(((((((<.((((><...{...))))((((((.......))))))...>((((((.}.)..)))))))))))....
3A3A	(((((((.(..((((((....)))))).((((((.......)))))..(((((....))))).((((.......))))).)))))))	(((((((....((((((....))))))((((((.......))))).(((((....))))).((((.......))))..)))))))	(((((((....((((((....)))))).(((((.......)))))..(((((....))))).((((.......))))..)))))))	(((((((((..(((((((..)))))))(((((((...))))))))(((((((....)))))))(((((...)..))))))))))))))	(((((((((..(((((((..)))))))((((((((...))))))))((((((((....))))))(((((...)..)))))))))))))	(((((((....((((((....))))))(((((.......)))))).(((((....)))))).((((.......))))..)))))))	...........((((((....)))))).(((((.......)))))..(((((....))))).((((.......))))...	(((((((.(..((((((<.<.)))))((((((.......))))))<(((((...>))))).(((((>.>)..))))).)))))))	((((((((...((((((....)))))))((((((.....))))))).(((((....))))))((((((...)..)))))))))))))
3ADB	(((((((((..((((((....))))))(((((((.......))))))((((((......))))))((((.......))))))))))))....	(((((((((..((((((....))))))((((((.......))))))(((((((....)))))))((((.......))))))))))))....	(((((((((..((((((....))))))((((((.......))))))(((((((....))))))((((.......)))))))))))))....			(((((((((..((((((....))))))((((((.......))))))(((((((....))))))((((.......)))))))))))))....	(((....))).((((((....))))))((((((.......))))))((((((((....)))))))((((.......)))).	((((((((...((((((....))))))(((((((....).)))))).((((((....))))))(((((...)..))))))))))))))....	(((((((((..((((((....))))))((((((.....)))))))((((((((....))))))(((((...)..))))))))))))))....
3AKZ	(((((((.(((((.......)))))(((((....)))))....(((((.......))))))))))))....	((((((((((((.......)))))).(((((.((((((((())))))..))))))))..)))))))....	.((((((....(....)......((((((.......))))))..(((((.......))))))))))).....	((((((((((((((...)))))))(((((((..((((((((...)))))))))))))))))))))))....	.((((.(((((((((...)))))))))((((((.(((((.[[[[[.[..))))))))))))))))..]]]]]]..	(((((((.(((((.......)))))((((.......)))))....(((((.......))))))))))))....	(((((((.(((((.......)))))((((.......)))))....(((((.......))))))))))))....	(((((((..((((.{<<<..))))((((((.......)))))))..}((((((>>>)..))))))))))))....	(((((((<.((((>...<..))))(((((.......))))))...((((((.>.)..)))))))))))....
3AMT	(((((((..((((..........))))((((((.......))))))....(((((.......)))))))))))....	(((((((..(((......)))..((.((((((.......))))))).))(((((.......))))))))))))....	(((((((..................(((((((.......)))))))...(((((.......))))))))))))....	(((((((..(.((((((((((((((((..).)))).))))))))))))(((((((...)..)))))))))))....	(((((((..(.((((((((((((((((..).)))).)))))))))))(((((((...)..))))))))))))....	(((((((................((.(((((((.......))))))).))(((((.......))))))))))))....	(((((((................((.((((((((.......))))))).))(((((.......))))))))))))....	(((((((..((((..........))))(((((((.......))))))....((((((.......)))))))))))....	(((((((<.((((><........))))((((((.......))))))...>((((((...)..))))))))))))....
3EGZ	(((((...((((...........))))((((((((..........))))))))........)))))	(((((...((((..(....)...))))((((((((..........))))))))........)))))	(((((...((((...........)))).((((((((..........)))))))........)))))	(((((((((((((((((((((((((...))))))))))((((((..))))))))))))))))))))	((((((((((((((((((((((((...))))))))))((((((..)))))))))))))))))))))))	(((((...((((...........))))((((((((..........)))))))).......)))))	(((((...((((...........))))((((((((..........)))))))).......)))))	(((((.(.((((..((.).)<..)))(((((((((..........))))))))...>.).)))))	(((((...(((............))))((((((..........)))))))......)))))
3EPJ	((((((..((((.......))))(((((((.....)))))))....(((((.......))))))))))))	(((((((..(((.......)))((((((((((..)))))))))....((((.......))))))))))	(((((....(((.......)))((((((((.....)))))))...(((((.......))))).)))))	((((((.(((((.(((..))))((((((((...)))))))).)))((((((...)..))))))))))	((((((.(((((.(((..))))((((((((((...)))))))).)))((((((...)..))))))))))))	(((((((((.............((((((((...)))))))))..............))...)))))))	(((((((((.............((((((((...)))))))))..............))...)))))))	((((((((<<<))(({....>>>((((((((...)))))))).)).((((((...)}.))))))))))))	((((((..(((((.....)))))((((((((.....)))))))....((((((...)..))))))))))
3FOZ	(((((((..((((........)))).(((((((...)))))))......(((((.......))))))))))))..	((((((((...((((((((.((.((((((((((...))))))).))).))....))))))..)))))))))..	((((((...((((........)))).((((((.(....))))))......((((......))))..)))))))..	((((((((((((((((..)))))))(((((((((...)))))))))))))).(((((((.....)))))))))))..	..((((((.[[[[))))))..]]]].(((((((...)))))))((((((.(((((((....)))))))))))))	.........((((........)))).(((((((...)))))))((((((..((((((....)))))).))))))	.........((((........)))).(((((((...)))))))((((((..((((((....)))))).))))))	(((((((<.((((>.......)))).(((((.......)))))......(((((((...)..)))))))))))))..	(((((((<.((((><..{...))))(((((((.....)))))))...>((((((}..)..)))))))))))..
3ID5	(((....)))(..((((....)))).....)..	(((((())))))...((((....))))........	.((((((.......))))))..........	(((...)))((((((((((..))))))))))....	(((...)))((((((((((..))))))))))....	.((((((.......))))))..........	.((((((.......))))))..........	.........((.<((((....)))).))>...	.............(((((..))))))........

PDB	MXfold2	CONTRAfold	Centroidfold	Mcfold Default	Mcfold Pseudoknotted	Linearfold C	Linearfold V	Ufold	SPOT-RNA
3IVK	((((((((..........))))))))..................((((...))))((((((((((((.....)))))..(((((....)))))((.(((((....)))))).))))))))).........	((((((((..........))))))))...................((((...))))((((((((((((.....)))))..(((((....)))))((.(((((....))))).))))))))).........	.(((((((..........)))))))...................((....))..(((((((((((.....))))...(((((....))))))...(((((....)))))..)))))))..........	((((((((((((..))))))))))))...((((((((..))))((((...))))(((((((((((((...))))))..((((((..)))))((((((((((..))))))))))))))....).)))	(((((((((.[[[[[[[)))))))).......((((..))))((((...))))(((((((((((((...))))))..((((((..))))))((((((((((..)))))))))))))))))..]]]]]]	((((((((..........))))))))..................((((...))))((((((((((((.....)))))..(((((....)))))((.(((((....))))).))))))))).........	((((((((..........))))))))..................((((...))))........(((((.....)))))….	((((((((.(......).))))))))................((((...))))((((((((((((.....))))).(((((....)))))((.((((((.)))))).))))))))).........	(((((((((....<<<.))))))))).................(((((.))))).((((((((((.....)))))..(((((....)))))..(((((((..)))))))..)))))....>>>...
3K0J	((((((((((....(((((..........))))).........))))).....((((..((((......))))..))))..))))))	(((((((((((((((........))))))))........................((((..((((......))))..))))..))))))	((((((((((((((........))))))))........(....)..........(((...((((......))))...)))..))))))	(((((((((((((((((..)))))))))))(((((((.(((((((((((((((..))))))))))))))))))))))))....))))))	.....(((((((((...[[[[.))))))))))((((((.((((((((((((((..)))))))))))))))))))))]]]]	......((((((((........))))))))........................((((..((((......))))..))))........	......((((((((........))))))))........................((((..((((......))))..)))).	(((((((((((((........))))))))..........................((((..((((......))))..))))..))))))	((((((((((..(((.(((..........))))))........)))).....((((((.((((((.)).))))).))))).))))))
3KTW	.(((((.((((((((((((((((....))))))))))).))))))....(((((.(...(((.....(((....))).....)))).))))))))))	.(((((((((((((((((((((((....)))))))))).))))).((.((((.....)))).))(((.(((..(....)..))).)))..))))))	.((((((((((((((((((((((....)))))))))).))))........((....))........(((....)))............)))))))	.(((((.((((((((((((((((....)))))))))).)))))((((((((((...)))))))).(((((((((.(..)))))))))))))))))		.(((((.((((((((((((((((....)))))))))).)))))....(((((.....(((.....(((....))).....)))..))))))))))	.......(((((((((((((((....)))))))))).)))))....((((.....)))).....(((....)))....	.((((((((((((((((((((((.....))))))))).))))))..(.((((.....))).).(((((<<..)))......>>...))..))))))	.(((((((((((((((((((((((..)))))))))).)))))......((((.....((((((((((((..))))))))))).))))))))))
3NDB	(((((((((((((((((.(((((..(((((.((((((((((....)))))))))..))))).....((((((.....(((......(((....))).....)))..)))))).))))))).))))))))...))))))))	((((((((((((((((.((((((..(((((.((((((((....)))))))))..)))))....((((((..(..(((......(((....)))......))))).)))))).))))))).))))))))...)))))))	((((((((((((((((.(((((..((((((.((((((((((....)))))))))..))))))....((((((...(.........((((....))).......)..)))))).))))))).))))))))...)))))))	(((((((((((((((((((((((((((((((((((((((....)))))))))))))))....))).)))))))))))))...((((((((((.(((((((((..))))))))))))))))))))))).)))))))	((((((((((((((((((((((((((((((((((((((..))))))))))))))))....)))).))))))))))))...(((((((((((.(((((((((..))))))))))))))))))))))))))).)))))))	((((((((((((((..((((((..((((((.(((((((((....)))))))))..))))).....((((((.....(((......(((....))).....)))..)))))).))))))))..))))))))...)))))))	..........((((((.(((((((.(((((.((((((((((....)))))))))..))))))).....))))))).)))))).	((((((((((((((((<((((((((<(((((.(((((((((....))))))))..))))))..>((((((..(((..(......(((....))).....))))).)))))).))))))))>))))))))...)))))))	((((((.........<.<<......(((((.(((((((((((..)))))))))))..))))....((((((>>.>((((((((((((..))))))))))))).))))))....................))))))
3NVK	...((...((....))...))...	........((....))........	........((....))........	(((((.(.(((..)))))))))..	(((((.(.(((..))))))))))..	....	....	...((...((....))...)...)	...((.(((((..))))).))...
3PHP	((((((((......)))))))).	((((((((......)))))))).	((((((((......)))))))).	((((((((((..)))))))))).	((((((((((..)))))))))).	((((((((......)))))))).	((((((((......)))))))).	((((((((.(()).)))))))).	(((((((((...).)))))))).
3SN2	((((((.(((((......)))))))))))	((((((.(((((......)))))))))))	((((((.(((((......))))))))))))	((((((((((((...))))))))))))))	((((((((((((...)))))))))))))	((((((.(((((......))))))))))))	((((((.(((((......))))))))))))	((((((.((((((...).)))))))))))	((((((.(((((((.)).))))))))))))
3TUP	(((((((..((((........))))..((((.......))))......(((((.......)))))))))))))..	(((((((.((.(((((((.(.......)))))))))......(((.(((((....))))).)))))))))))..	(((((((..((((........))))..((((.(....)))))........((((.......)))).)))))))..	(((((((.((((((((((((((..))))))))))))((((((((((((..))))))))))))).)))))))))..	(((((((.((((((((((((((..)))))))))))(((((((((((..)))))))))))).))))))))))..	(((((((....(((((((..........))))))).....((((.(((((....)))))).)))).)))))))..	(((((((....(((((((..........))))))).....((((.(((((....)))))).)))).)))))))..	(((((((..((((........)))).(((((.......))))).....((((........)))))))))))))..	(((((((<.((((><...{..))))((((((.....)))))))...>((((((.}.)..)))))))))))))..

PDB	MXfold2	CONTRAfold	Centroidfold	Mcfold Default	Mcfold Pseudoknotted	Linearfold C	Linearfold V	Ufold	SPOT-RNA
3U4M	((((.(.........(((((..(((.(((..((((((((....))))))))).....))).)))......))))).))))	(.........(((..((((((.((((.....(((((((....))))))))..)))).)..........))))..))))	(((..........(((((....)))))....(((((((((....))))))))).........((((......))))...)))	((((((((((((((((((((((((...((((((((((..))))))))))))))))))))).))))))))))))))		..........(((..(((((..(((.(((..((((((((....))))))))).....)))).)))......)))))..))).	..........(((..(((((..(((.(((..((((((((....))))))))).....))).)))......)))))..))).	((.........(.<((((((......>.....(.((((((<<)))))).)>>................))))).).)))	((((((...(.....)((((((((((...(((((((((((..))))))))))))))))...((...)))))))))))))
3W1K	(((((((...((((((....))))))((((((.......)))).))(((((((.....))))))).(((((.......))))).))))))).	(((((((...((((((....))))))((((((.......)))).))(((((((.....)))))))).(((((.......))))).))))))).	(((((((...((((((....))))))....(....).((((((.....))))))..(((((.......))))).)))))))).			(((((((...((((((....))))))((((((.......)))).))(((((((.....))))))).(((((.......))))).))))))).	.(((((....((((((....))))))....))))).(((((((..(((((((.....)))))))).))))))).......	(((((((...((((((....))))))(.((((.......)))).).((((((((.....))))))).(((((.......))))).))))))).	(((((((...((((((....)))))).((((((.....))))))..((((((((.....))))))).((((((...)..))))).)))))))).
3W3S	((((((((..(((.(((....)))))).((((((.......)))))..((((.(((......))).)))).((((((.......))))))))))))...	((((((((..((.((((....))))))(((((((.......)))))).)((((.((((....))))).)))).(((((.......)))))))))))))...	(((((((......((((....))))...(((((.......)))))..((((.((((....)))).))))((((((.......))))))))))))...			(((((((...((.((((....)))).))(((((.......)))))..((((.((((....)))).))))((((((.......))))))))))))...	((((((((((((.((((....)))).))...))))))...))))...((((.((((....)))).))))..........	((((((((..((.((((....))))))<)((((((.......))))).>(((.((((....)))).)))).(((((.......))))))))))))...	((((((((..((.((((....)))))).(((((((.....))))))..((((((((((.))))))))))).((((((...)..)))))))))))...
3WC1	(((((((..((....(((((((...........))))))).....))((((((.......)))))))))))..	(((((((..((.((((((((((.((.....)).))))))).))).))((((((.......)))))))))))..	(((((((.........((((((.........)))))).........(((((.......)))))))))))))..	(((((((..(((((((((((((((.....)))))))))).)))))))((((((...).))))))))))))..		(((((((..((.((((((((((...........)))))).)))).))(((((.......))))))))))))..	(((((((..((.((((((((((...........)))))).)))).))(((((.......))))))))))))..	(((((((..(((..........))).(((((.......)))))....(((((.......))))))))))))..	(((((((..((((........)))).(((((.......)))))....((((((...)..))))))))))))..
3WC2	(((((((..((((........)))).((((((.......)))))).....((((((.......))))))))))))..	(((((((..((((........))))......(((((((((.((...........)))))))))))))))))))..	(((((((..((((........))))......((((((((................)))))))).)))))))..	(((((((...(((((((((((((..)))))))))))))((((((..))))(((((((...)..))))))))))))..		(((((((..((((........))))......(((((((((................))))))))))))))))))..	(((((((..((((........))))......(((((((((................))))))))))))))))))..	(((((((..((((....<...))).(((((.......))))).....(((((.>.....)))))))))))))..	(((((((<.((((><......))))((((((.......))))))...>((((((...)..))))))))))))..
3WFQ	(((((((..((((........)))).((((((.....)))))).....((((((.......))))))))))))).	(((((((..((((........))))((((((.......))))).)...((((((.......)))))))))))).	(((((((..((((........)))).(((((.(....))))))....((((((........))))))))))))).	(((((((..(((((((..))))))))((((((.((..))))))))....((((((...).)))))))))))).		(((((((..((((........)))).((((((.......))))).....(((((......))))))))))))).	(((((((..((((........)))).(((((.......))))).....((((((....)))))))))))).	(((((((..((((........)))).(((((((.......))))).....((((((.......))))))))))))).	(((((((<.((((><..{[..))))((((((((.....)))))))...>((((((}].)..)))))))))))).
3WFS	(((((((..((((........)))).((((((.....)))))).....((((((.......)))))))))))))..	(((((((..((((........))))((((((.......))))).)...((((((.......))))))))))))..	(((((((..((((........)))).(((((.(....))))))....((((((........)))))))))))))..	(((((((..(((((((..))))))))((((((.((..))))))))....((((((...).))))))))))))..	(((((((..(((((((..)))))))((((((.((..)))))))))....((((((...)..)))))))))))))..	(((((((..((((........)))).((((((.......))))).....(((((......)))))))))))))..	(((((((..((((........)))).(((((.......))))).....((((((....)))))))))))))..	(((((((..((((........)))).(((((((.......))))).....((((((.......)))))))))))))..	(((((((<.((((><..{[..))))((((((((.....)))))))...>((((((}].)..))))))))))))..
3WQY	(((((((..((((.......)))).((((((....)))))).....((((((.......))))))))))))))....	(((((((.......(((((((((((.(((((((...)))))))).)))..)))...)))))))))))))....	(((((((..................((((((.....)))))).....((((((.......)))))))))))))....	(((((((((((...))).))))))))((((....)))(((((((((((((((((...)..))))))))))))))))		(((((((.......((((((((((.((((........(((....))))))))))))...))))))))))))))....	(((((((.......((((((((((.((((........(((....))))))))))))...))))))))))))))....	(((((((..((((.......)))).((((((.......))))).....(((((.......))))))))))))....	(((((((<.((((>......))))((((((((.....)))))))....((((((...).))))))))))))....
3WQZ	(((((((..((((.......)))).((((((....)))))).....((((((.......))))))))))))))....	(((((((.......(((((((((((.(((((((...)))))))).)))..)))...)))))))))))))....	(((((((..................((((((.....)))))).....((((((.......)))))))))))))....	(((((((.(((...)))((((..))))((((((((...))))))).((((((...)..))))))))))))....		(((((((.......((((((((((.((((........(((....))))))))))))...))))))))))))))....	(((((((.......((((((((((.((((........(((....))))))))))))...))))))))))))))....	(((((((..((((.......)))).((((((.......))))).....(((((.......))))))))))))....	(((((((<.((((>......))))((((((((.....)))))))....((((((...).))))))))))))....

PDB	MXfold2	CONTRAfold	Centroidfold	Mcfold Default	Mcfold Pseudoknotted	Linearfold C	Linearfold V	Ufold	SPOT-RNA
484D	((((((..((.......))))))))	((((((..((.......))))))))	((((((............))))))	((((((..((((...))))))))))	((((((..((((...))))))))))	((((((..((.......))))))))	((((((..((.......))))))))	(((((((.((........)))))))))	(((((.((..(....)..)))))))
4A4S	((((((((....))))))))	((((((((....))))))))	((((((((....))))))))	((((((((....))))))))	((((((((....))))))))	((((((((....))))))))	((((((((....))))))))	((((((((....))))))))	((((((((....))))))))
4AL5	.(((((.....)))))	.(((((.....)))))	.(((((.....)))))	.((((((...))))))	.((((((...))))))	.(((((.....)))))	.(((((.....)))))	.(((((.....)))))	.(((((.....)))))
4AL7	(((((.....)))))	(((((.....)))))	(((((.....)))))	((((((...))))))	((((((...))))))	(((((.....)))))	(((((.....)))))	((((((..).)))))	(((((.....)))))
4BW0	(((...(((....)))......))).	(((...(((....)))......))).	.(((..(((....)))......))).	((((((((((..)))))....)))))	((((((((((..)))))....)))))	(((...(((....)))......))).	(((...(((....)))......))).	.(((..(((....)))......))).	(((...((((..))))......))).
4C4W	(((((.(((((..........)))))..))..)))	..((((......)))).....((((....))))..	..((((......))))....((((....))))..	(((((((((....)))))).)((((..))))))	(((((((((....)))))).)((((..)))))))	....	....	(((...(((((.(.)......)))))......)))	(((<....(((...>......))).......)))
4C7O	((((((((((((((.(((((....)))))).))))).).))))))))	((((((((((((((((((((....)))).)).)))))).).))))))))	.(((((((.((((((((((....)))).)).)))))...))))))).	(((((((((((((((((((((..))))).))).)))).).))))))))	(((((((((((((((((((((..))))).))).)))).).)))))))))	(((((((..(((((..(((((....)))))..)))))....)))))))	(((((((..(((((..(((((....)))))..)))))....)))))))	((((((((.(((((((.(((<...)>.))))).))))...))))))))	(((((((((((((((((((((..))))).))).)))).).))))))))
4ILM	.(((........))).	....	....	((((((....))))))	((((((....))))))	....	....	....	.(((.<......)))>
4KR7	(((((((.......(((((....)))))))))))))....	(((((((.......(((((....)))))))))))))....	(((((((.......(((((....))))))))))))....	(((((((((((((((...)))))))))..))))))....	(((((((((((((((...))))))))..)))))))....	(((((((.......(((((....))))))))))))....	(((((((.......(((((....)))))))))))))....	((((((((..({((.<<<.)).)}>>>))))))))....	((((((((.......((((....)))))))))))))....
4KR9	(((((((.......(((((....)))))))))))))....	(((((((.......(((((....)))))))))))))....	(((((((.......(((((....))))))))))))....	(((((((((((((((...)))))))))..))))))....	(((((((((((((((...))))))))..)))))))....	(((((((.......(((((....))))))))))))....	(((((((.......(((((....)))))))))))))....	((((((((..({((.<<<.)).)}>>>))))))))....	((((((((.......((((....)))))))))))))....
4L8H	((((.((((...))))))))	((((.((((...))))))))	.(((.((((...))))))).	((((.((((...))))))))	((((.((((...))))))))	((((.((((...))))))))	((((.((((...))))))))	((((.((((...))))))))	((((.(((((.)))))))))
4M4O	((((((.(((((.........(((((.(..........).)))))..))))).))))))	((((((.(((((.((..((.(.((.(((...))).)).).))..)).))))).))))))	((((((.(((((.(......).((((.............))))...))))).))))))	(((((((((((((((((((((((((((...)))))))))))))))))))))))))))))	((((((((((((((((((((((((((((...)))))))))))))))))))))))))))))))	((((((.(((((.........(((((.(..........).)))))..))))).))))))	((((((.(((((.........(((((.(..........).)))))..))))).))))))	((((((.(((((.........((((((..<((..>)))..)))))..)))).))))))	((((((((((((((.......((((((...((.))..)))))))))))))))))))))
4M6D	(((.........(((((.(..........).))))))..)))	(((.(.......(((((.(..........).))))))).)))	(((..........((((.............))))...)))	((((((((((((((((((...))))))))))))))))))))	((((((((((((((((((...))))))))))))))))))))	............(((((.(..........).)))))....	............(((((.(..........).)))))....	(((.(....<<.(((((.(..((>>.)).).)))))).)))	((((((.......((((((...((.))..)))))))))))))
4OOG	((((((((((((((....))))))))))))))..	((((((((((((((....)))))))))))))))..	(((((((((((((....))))))))))))))..	(((((((((((((....))))))))))))))..	(((((((((((((....)))))))))))))))..	((((((((((((((....))))))))))))))..	((((((((((((((....))))))))))))))..	((((((((((((((....))))))))))))))..	((((((((((((((....)))))))))))))))..
4P3E	(((((...((((((((((.(((((..(((((((....))))))..))))).))))((...)).((((((.....((((....(((....)))....)))).)..))))).)))))))....)))))	(((((.(.((((((((((.(((((..((((((....))))))..))))).)))).....((((...(..(((.(((.....((....))).)).)))....))))).))))))).)..)))))	.((((.(.((((((.............(((((....))))).................((((.((....))((((....(((....)))....))))....))))..)))))).)..)))).	((((((((((((((((((((((((((((((((((....)))))))))))))))))))))..(((((((((.((((((((((((((..)))))))))))))))))))))))))))))))).)))))	((((((((((((((((((((((((((((((((((....))))))))))))))))))))))....(((((((((.(((((((((((((..))))))))))))))))))))))))))))).)))))	(((((.(.((((((((((.(((((..((((((....))))))..))))).)))).....((((......(((.((((.........)).)).)))......)))).))))))).)..)))))	..........((((......(((((..((((((....))))))..)))))...)))).......((((.......))))..	(((((.(.(((((((.(((.(((((.(.(((((....))))))..))))).)))..........(((((..(((<(......(((....)))..).)))>...).))))))))))).)..)))))	(((((...(((((.......(((((...(((((((..))))))...)))))..........................(((........)))..((...................)))))))....)))))

PDB	MXfold2	CONTRAfold	Centroidfold	Mcfold Default	Mcfold Pseudoknotted	Linearfold C	Linearfold V	Ufold	SPOT-RNA
4P8Z	(((((..((((((.(((((((((.......))))))))).(((((((....(.((...(((((.....((((...((((...))))........)))))))))).)).)...(((((...((.(.(((((...))))).).))......))))).((((....))))..))))))).))))))..)))))	(((((..((((((.(((((((((.......))))))))).(((((((...((((((((............((((((((.....)(((......((((....))))............))))))))))..))))))))..........(....)((((....))))..))))))).))))))..)))))	(((((..(((((..(((((((((.......))))))))).(((((..((....))..((((....)))......)))))..)))))..)))))	....((((....))))........................	....((((....))))..........................	(((((..((((((.(((((((((.......))))))))).(((((((...((.....(((((((......(((((((.......((((....((.(((.........))).))....)))))))))))..........)))))).....))..((((....))))..))))))).))))))..)))))	..............(((((((((.......)))))))))..	(((((..((((((.(((((((((....<..))))))))).({{{(((((((({...{{{..(((((...((((((.((((..>))))....)).))))((...<))...<.<<<<<...)))))((((..}}}))))))}})).)).}}.>>>>>>((((>...))))...)))..).))))))..))))))	(((((.(((((((.((((((((((.....))))))))))....................................((((..............))))..(((((..)))))..........))))))).)))))
4PDB	((((((((.(..((((....))))..).))))))))))	((((((((.(..((((....))))..).))))))))))	((((((((.....(((....)))......)))))))))	(((((((((((((((((..)))))))))))))))))	((((((((((((((((..)))))))))))))))))	((((((((.(..((((....))))..).))))))))	((((((((.(..((((....))))..).))))))))	((((((((.((.(((((..))))))).).))))))))	(((((((((..((((((..))))..)))))))))))
4PMI	((((((.....(.(((((....))).)).)....))))))	((((((...((..(((((....))).))...)).))))))	.(((((....(..(((((....))).))...)..))))).	((((((..((((((((((..))))).).)))))))))))	((((((..((((((((((..)))).).)))))))))))	((((((.......(((((....))).)).....))))))	((((((.......(((((....))).)).....))))))	((((((.(.(((.((((((..)))).)).).)))))))))	((((((..((((((((((..)))).))).))))))))))
4QI2	.(((((((...))))))).	.(((((((...))))))).	........(((....))).	((((((((...))))))))	((((((((...))))))))	....	....	.(((((((...))))))).	.(((((((...))))))).
4QIL	.((((((...))))))	.((((((...))))))	.((((((...))))))	.((((((...))))))	.((((((...))))))	.((((((...))))))	.((((((...))))))	.((((((...))))))	.((((((...))))))
4QOZ	.....((((((....))))))....	.....((((((....))))))....	.....((((((....))))))....	.((((((((((....)))))))))).	.((((((((((....)))))))))))).	.....((((((....))))))....	.....((((((....))))))....	.....((((((....))))))....	....(((((((....)))))))....
4TV0	.......((((((....))))))...	.......((((((....))))))...	.......((((((....))))))...	(((....((((((....)))))))))	(((....((((((....)))))))))	.......((((((....))))))...	.......((((((....))))))...	.......((((((....))))))...	.......((((((....))))))...
4TZV	.....((((.......)))).(((((((...))))))))(((..((((((........))))))))((((....))))	.....((((.......)))).(((((((...))))))))(((..((((((....).))))))))((((....))))	.....((((.......)))).(((((((.....))))))(((...((((........)))).)))((((....))))	((((((((((((...)))))))(((((((...)))))))(((((((((..))))))))).)))((((((..))))))	(((((((((..[[[[[)))))))))..]]]]](((((((((((((..)))))))))))))).(((((..)))))	.....((((.......)))).((((((.....))))))).....(((((.......))))))...((((....))))	.....((((.......)))).((((((.....))))))).....(((((.......))))))...((((....))))	(((..........(....<)((((((((...))))))).>...(((((.......))))))))((((....))))	(((..((((((.....))))))(((((((((..)))))))....((((((....)..)))))))(((((..))))))
4U7U	...((((((........))))))..........................((((((....))))))	...((((((.(.....)))))))..........................((((((....))))))	...(((((..........)))))..............(....)..((((((....))))))	((((((.(((..)))((((((((..))))))))..))))))....(((((((..))))))))	((((((.(((..)))((((((((..))))))))..))))))....(((((((..)))))))	...((((((........)))))).....................((((((....))))))	...((((((........)))))).....................((((((....))))))	((.((((((.(.....))))))))..................((((((....))))))	....(((((((..)))))))
4WC2	(((((((..((((........)))).(((((.......)))))).....(((((.......)))))))))))))...	(((((((..((((........))))((((((.......))))).)...(((((.......))))))))))))...	(((((((..((((........)))).((((((.(....))))))).....(((((.......))))))))))))...	(((((((..((((((..)))))))((((((.((..))))))))....((((((...)..))))))))))))...	(((((((..((((((..)))))))((((((.((..))))))))....((((((...)..))))))))))))...	(((((((..((((........)))).((((((.......))))).....(((((.......))))))))))))...	(((((((..((((........)))).(((((.......))))).....((((((....)))))))))))))...	(((((((..((((........)))).(((((.......))))).....(((((((.......)))))))))))))...	(((((((<.((((><..{...))))((((((.....)))))))...>((((((}..)..))))))))))))...

PDB	MXfold2	CONTRAfold	Centroidfold	Mcfold Default	Mcfold Pseudoknotted	Linearfold C	Linearfold V	Ufold	SPOT-RNA
4WC3	(((((((..((((........)))).(((((.......))))).....(((((.......))))))))))))....	(((((((..((((........))))((((((.......))))).)...(((((.......)))))))))))))....	(((((((..((((........)))).(((((.(....))))).....(((((.......))))))))))))....	(((((((..(((((((..)))))))((((((.((..))))))))....((((((...)..))))))))))))....	(((((((..(((((((..)))))))((((((.((..))))))))....((((((...)..)))))))))))))....	(((((((..((((........)))).(((((.......))))).....(((((.......))))))))))))....	(((((((..((((........)))).(((((.......))))).....(((((.......))))))))))))....	(((((((..((((........)))).(((((.......))))).....(((((.......))))))))))))....	(((((((<.((((><..{...))))(((((((.....)))))))...>((((((}..)..))))))))))))....
4WJ3	(((((((..((((........)))).(((((.......))))).....(((((.......))))))))))))....	(((((((..((((........)))).(((((.......))))).....(((((.......))))))))))))....	.(((((...((((........)))).....................(((((.......)))))).)))))).....	((((((((((((..(((((((((..))))))))))))(((....))).((((((...)..))))))))))))....	(((((((((((..((((((((((..)))))))))))))(((....))).((((((...)..)))))))))))))....	(((((((..((((........))))....((((..........))))((((((.......))))))))))))....	(((((((..((((........))))....((((..........))))((((((.......))))))))))))....	(((((((<.((((><..{[..)))).(((((.(...).)))))....>((((((}].)..)))))))))))))....	(((((((<.((((><...{..))))((((((.......))))))...>((((((.}.)..))))))))))))....
4X0A	((((((..((((........)))).(((((.......))))).....(((((.......)))))))))))...	((((((..((((........))))((((((.......))))).)...(((((.......)))))))))))...	((((((..((((........)))).(((((.(....))))))).....(((((.......)))))))))))...	((((((..(((((((..)))))))((((((.((..))))))))....((((((...)..)))))))))))...	(((((((.(((((((..)))))))))[[[[))))))..]]]](((((((((((...)..)))))...))))))	((((((..((((........)))).(((((.......))))).....(((((.......)))))))))))...	((((((..((((........)))).(((((.......))))).....(((((.......)))))))))))...	((((((..((((........)))).(((((.......))))).....(((((.......)))))))))))...	((((((<.((((><......))))(((((((.....)))))))...>((((((...)..)))))))))))...
4X0B	((((((((..((((........)))).(((((.......))))).....(((((.......)))))))))))))...	((((((((..((((........))))((((((.......))))).)...(((((.......))))))))))))))...	((((((((..((((........)))).(((((.(....))))))).....(((((.......)))))))))))))...	((((((((..(((((((..)))))))((((((.((..))))))))....((((((...)..)))))))))))))...	((((((((..(((((((..)))))))((((((.((..)))))))))....((((((...)..))))))))))))))...	((((((((..((((........)))).(((((.......))))).....(((((.......))))))))))))))...	((((((((..((((........)))).(((((.......))))).....(((((.......)))))))))))))...	((((((((..((((........)))).(((((.......))))).....(((((.......))))))))))))))...	((((((((<.((((><..{[..))))(((((((.....)))))))...>((((((}].)..)))))))))))))...
4X4O	((((((((((((.......)))))))))...)))	((((((((((((.......)))))))))...)))	((((((((((((.......)))))))))...)))	((((((((((((...)..)))))))))...)))	((((((((((((...)..)))))))))...)))	((((((((((((.......)))))))))...)))	((((((((((((.......)))))))))...)))	((.((((((((((....).))))))))).))....	((((((((((((.......)))))))))...)))
4X4P	((((((((((((.......)))))))))...))))..	((((((((((((.......)))))))))...))))..	((((((((((((.......)))))))))...))))..	((((((((((((...)..)))))))))...))))..	((((((((((((...)..)))))))))...)))..	((((((((((((.......)))))))))...)))..	((((((((((((.......)))))))))...)))..	((.((((((((((...)..))))))))).))....	((((((((((((.......)))))))))...)))..
4YCO	(((((((..((((........)))).((((((.....))))))).....(((((.......))))))))))))..	((((((((...((((((((.((.((((((((((...))))))).))).)).....)))))..)))))))))))..	((((((...((((........)))).(((((.(....))))).....(((((.......))))).))))))..	((((((((((((((((..))))))))((((((((...))))))))))))).(((((((....)))))))))))..	((((((((((((((((..))))))((((((((...)))))))))))))).(((((((....))))))))))..	(((((((..((((........)))).(((((((...)))))))).....(((((.......))))))))))))..	(((((((..((((........)))).(((((((...)))))))).....(((((.......))))))))))))..	(((((((<.((((>...<{..)))).(((((.......))))).....(((((>}.)..)))))))))))))..	(((((((<.((((><..{[..))))(((((((.....)))))))...>((((((}].)..))))))))))))..
4YVI	(((((((.(((((.......)))))(((((.......)))))....(((((.......)))))))))))).	((((((((((((((.......)))))).((((((.((((((((()))))))..))))))))))..)))))))).	.((((((....(....)......(((((((.......)))))))..(((((.......)))))))))))..	((((((((((((((((...)))))))((((((((..((((((((...))))))))))))))))))))))))).	.((((.(((((((((...))))))))))((((((.((((((.[[[[.[).))))))))))))))))..]]]]]	(((((((.(((((.......)))))(((((.......))))).....(((((.......)))))))))))).	(((((((.(((((.......)))))(((((.......)))))....(((((.......)))))))))))).	(((((((..((((.{<<...))))((((((.......))))))..}(((((>.>)..)))))))))))).	(((((((<.((((>...<..))))((((((.......))))))...((((((.>.)..)))))))))))).
4YVJ	(((((((.(((((.......)))))(((((.......)))))....(((((.......)))))))))))).	((((((((((((((.......)))))).((((((..((((((((()))))))...))))))))...)))))))).	.((((((....(....)......(((((((.......)))))))..(((((.......)))))))))))..	(((((((.(((((((...)))))))(((((.((..))))))).((((((((((..)))))))))))))))).	(((((((.(((((((...)))))))(((((.((..))))))).(((((((((..)))))))))))))))).	(((((((.(((((.......)))))(((((.......)))))....(((((.......)))))))))))).	(((((((.(((((.......)))))(((((.......)))))....(((((.......)))))))))))).	(((((((..((((...<...))))((((((.......))))))...((((((>..)..))))))))))))).	(((((((<.((((>...<..))))((((((.......))))))...((((((.>.)..)))))))))))).
4YVK	(((((((.(((((.......)))))(((((.......)))))....(((((.......)))))))))))).	((((((((((((((.......)))))).(((((..(((.((((()))...)).)))))))))...)))))))).	.((((((....(....)......(((((((.......)))))))..(((((.......)))))))))))..	(((((((.(((((((...)))))))(((((.((..))))))).((((((((((..)))))))))))))))).	(((((((.(((((((...)))))))(((((.((..))))))).(((((((((..)))))))))))))))).	((((((((((((((.......)))))))(((..(((((.((.........)).))))))...))).)))))).	((((((((((((((.......)))))))(((..(((((.((.........)).))))))...))).)))))).	(((((((..((((...<...))))((((((.......))))))...((((((>..)..))))))))))))).	(((((((<.((((>...<..))))((((((.....)))))))...((((((.>.)..)))))))))))).

PDB	MXfold2	CONTRAfold	Centroidfold	Mcfold Default	Mcfold Pseudoknotted	Linearfold C	Linearfold V	Ufold	SPOT-RNA
4YYE	((((((((((((........)))).((((.........)))).......((((((....)))))))))))))))..	((((((((((((........)))).((((.........)))).......((((....).)))))))))))..	(((((((((......................(((....)))..................)))))))))..	(((((((((((((((..))))))(((((((((((..)))))))))))((((((..)))))))))))))))..	(((((((((((((((..))))))((((((((((((..))))))))))))((((((..)))))))))))))))..	((((((((((..)))))))))))..	((((((((((..)))))))))))..	(((((((..((((........)))).((((.........))))......((((((.......)))))))))))))..	(((((((..(((((......)))))((((((.......))))))....((((((...)..)))))))))))..
4Z0C	..((......)).	....	....	.((((....))))	.((((....))))	....	....	....	....
4Z7L	((..........))	....	....	(((........)))	(((........)))	....	....	....	((..........))
4ZLD	(((((((...)))))))	(((((((...)))))))	.((((((...)))))).	(((((((...)))))))	(((((((...)))))))	....	....	(((((((...)))))))	(((((((...)))))))
4ZT0	((((.(((((.((.....((((....))))...........)).))..)))...)))).((((....))))..	.....(((..(((((((.((((....))))...)))))))).......))).........((((....))))..	.................((((....)))).((.........))............((((....))))..	((((((((((((((((((((((..))))))..)))))))))))))))))((((((((...))))))))....	((((((((((((((((((((((..)))))..)))))))))))))))))((((((((...))))))))....	.................((((....))))..	.................((((....))))..	......<<..((((((..((((....))))....))))))((((.()>>)).))....((((....))))..	..........((((((.((((((..))))))<<<)))))).((((..).)))......>>>...........
5A18	((((((((...((.......))..))))))))	((((((((...((.......))..))))))))	((((((((...((.......))..))))))))	((((((((((((((...)))))).))))))))	((((((((((((((...)))))).))))))))	((((((((...((.......))..))))))))	((((((((...((.......))..))))))))	((((((((((..(.....)..)).)))))))	(((((((((....(((..)).)).))))))))
5CCB	.(((((((..((((........)))).((((((.......)))))).....((((((.......))))))))))))....	.(((((((..((((.(....).)))).....(((((((.....)))))))((((((.......))))))))))))....	.(((((....((((........))))....................(((((.......)))))..))))).....	(((((((((.(((((((..))))))))))).((((((((...))))))))((((((...)..)))))..))))).)..	(((((((((.(((((((..))))))))).((((((((...))))))))((((((...)..)))))..))))).)..	.(((((((..((((........))))....((((((((.....))))))))(((((.....))))))))))))....	.(((((((..((((........))))....((((((((.....))))))))(((((.....))))))))))))....	.((((((...((((........)))).(((((.......)))))......((((.......))))).))))))....	((((((((<.((((><...{..))))((((((.....)))))))...>(((((.}.)..))))))))))))..).
5D6G	((((..(...(((((((((.((((.........))))).))))).......(.((......)).).))))))).))))	((((..((...((((((((.(((((.......)))))).)))).....((((........)))).))))))).))))	((((..(....((((((((.(((((.......)))))).)))).....((((........))))).)))).).))))	((((.((((((.(((((((((((((((...)..))))))))))))).(((((((.....))))))))))))))))))	((((.((((((.((((((((((((((...)..)))))))))))))).(((((((.....)))))))))))))))))))	((((.......((((((((.(((((.......))))).)))).....((((........)))).))))))..))))	((((.......((((((((.(((((.......))))).)))).....((((........))))).)))))..))))	((((....((<((((((((<((((((.......))))).))))))...{>((({((...).).)))))))>)))))	(((((....((((((((((..((((((.......)))))..)....................)).)))))))))))
5F5F	(((((.(......).)))))	.((((.(......).)))).	..(((..........)))..	((((((((((..))))))))))	((((((((((..))))))))))	....	....	(((((.(......).)))))	((((((((....))))))))
5F5H	(((((((......)))).)))	(((((((......)))).)))	(((((((......)))).)))	((((((((((..)))))).)))	((((((((((..)))))).)))	(((((((......)))).)))	(((((((......)))).)))	(((((((......)))).)))	(((((((......)))).)))
5F9F	((((((((((....))))))))))	((((((((((....))))))))))	((((((((((....))))))))))	(((((((((((..)))))))))))	(((((((((((..)))))))))))	((((((((((....))))))))))	((((((((((....))))))))))	((((((((((....))))))))))	((((((((((....))))))))))
5FJ4	(((...((((((..........))))))......)))	...((((.((....)).)))).............	.............((((....)))).........	((((((((((....))))))))..(((...)))))))	((((((((((....)))))))).(((...))))))	...((((.((....)).)))).............	...((((.((....)).)))).............	(((.......(.((((((....)))))).)..)))	(((..........((((((..))))))......)))
5FMZ	.((....))...	....	....	.((((...))))	.((((...))))	....	....	.(......)...	....
5HR6	(((((..((((.........))))((((((.......))))))...(((((.......))))))))))	(((((..((((.(.(()).))))((((((.......))))))...((((((....).)))))))))))	((((...(((..........)))((((((.......))))))...(((((.......))))).))))	((((((((...)))((((..))))((((((((...))))))))...((((((...)..))))))))))	((((((((...)))((((..))))((((((((...)))))))))...((((((...)..))))))))))	(((((..((((.........))))((((((.......))))))...(((((.......)))))))))))	(((((..((((.........))))((((((.......))))))...(((((.......))))))))))	(((((..((((.........))))((((((.......))))))...(((((.......))))))))))	(((((..((((.........))))((((((.......))))))...((((((...)..))))))))))

PDB	MXfold2	CONTRAfold	Centroidfold	Mcfold Default	Mcfold Pseudoknotted	Linearfold C	Linearfold V	Ufold	SPOT-RNA
5HR7	(((((..((((.........))))((((((.......))))))...(((((.......)))))))))))..	(((((..((((.(.(()).))))((((((.......))))))...(((((.......))))))))))..	.......(((...........)))((((((.......))))))...(((((.......))))))).......	((((((((...)))((((..))))((((((((...))))))))...((((((...)..))))))))))..	(((((..[[[[)))))((((((((((((((...)))))))))))).((((((..))))))))))))]]]]	(((((..((((.........))))((((((.......))))))...(((((.......)))))))))))..	(((((..((((.........))))(((((.......))))))...(((((.......))))))))))..	(((((..((((.........))))((((((.......))))))...((((.......))))))))))..	(((((..(((((.......)))))((((((.......))))))...((((((...)..))))))))))..
5ID6	....(((((.....)))))..	....(((((.....)))))..	.....((((.....))))...	((((((((...))))))))	((((((((...))))))))	....(((((.....)))))..	....(((((.....)))))..	....(((((.....)))))..	...((((((.....)))))).
5IEM	((((((((((.(((((..(((((((....))).)))))))..))...))))))))))	((((((((((...((((.(((((((....)).)))).)..)))...))))))))))	((((((((((........(((((((....))).))))..........))))))))))	((((((((((((((((((((((((((..)))).)))))..)))))))))))))))	((((((((((((((((((((((((((..)))).))))))..)))))))))))))))	((((((((((...(((..(((((((....))).))))....)))...)))))))))	((((((((((...(((..(((((((....))).))))....)))...)))))))))	((((((((((((.(((..((((((((..)))).)))))))...).).))))))))	((((((((((....((..((((((((..)))).))))....))....))))))))))
5IWA	...(((..((.(.(.((((((...)))))).).).(.(.((((((((((....(((......)))).....(((((((((((...)))))))))))((((((...((((...((((((((((...((((.(((((((.((((((...((((((....))))))....))))))...(((((.(((((....))))).))))..((((...)))).))))))).)))))))))))(((.((.(((..((((((((.......)))))))))))...)).)))..(((((((.........)))))))....(((((.(((.....)))))))).((((....))))......))))...))))((((...(((((....))))).))))....))))))).((((......((((....)))).....))))....(((((..((....((((.....))))......))...)))))....((((((((((..(((((((((.((....))))...)))))))))).)).))))))((((((.....)))))).....((((((((..((((((((((.((((((((((......((((((.....))))))....))))))))..)))))))))....((((((...((((((((...(((((((....(((........)))......)))))))...)(((.........))).)))))))..))))))......((((...((((....))))....)))...((((.........))))...))))))))))....((((((.(((.((((((((((...))))))))))..)))..[illegible]	..........(((..((((((...)))))).)))....(((((.(((.........))).)))))((.((((((((((((...))))))))))))).))....((.(((........((((((...((((.(((((((.((((((...((((((....))))))....))))))...(((((.(((((....))))).)))))..((((...)))).))))))).))))))))))))))).))((((()))))((((((........))))))............((.(((((((.........))))))).)).......(((.....((((((((.((((....))))......(.......((((((.((((((((....)))))..v...(((.....))))))..))))))..((((((((((.((.....(((((....)))))....))))))...)))))......).....(((.((((((.((..(((((..((((((((.((....)))))...)))))))))).)).)))))))))))... ..((((((((..((((((((..(((.((((((((.((((((((......((((((.....))))))....))))))))).).(((((.(...((((((((...((((((((...(((((((.((.(((.........)))..)).))))))))...)(((.........))).))))))))..))))))).))...))))))((((....)))).((........))..........))))))).))))))))))))..[illegible]	...........................(....)................................((((((((((.....))))))))))..........................((....))....((((((.((((((...((((((....))))))....))))))....((((.(((((....))))).))))...((((...)))).))))))..........................((((((........))))))..(.....)...(((((((...(((....))).(((((((....(((((.((........))))))).((((....))))...........................((((....))))......((.......))............((((....))))..............(((((........((((.....))))..........)))))........)))))))..((((((((((.........)))...))))))).((....)))))))))).......(((((((...((((((((.......((((((..(.((((((.(....).((((((.....))))))(....))))))))...).))))))......(((((....((((((.......((((((....(((.........))).....))))))............((....))..))))))...)))))).............(((....)))..[illegible]			..................(((((.(((..((((((((((((..((((...........(((((((.((((((((((.....))))))))))).))(((.((.(((........(((((((...((((.(((((((.((((((...((((((....))))))....))))))...(((((.(((((....))))).))))).((((...)))).))))))).))))))))))))))).)).)))..((((((((.......))))))))).))))))...)))).))).......(((..((((((((..((((((((((..((...((((.....((((.((((.(((.((.((((((...(((.(((.....))).))))))))))...))...))).)))).))))....)))).))..))))....))))))..(((((..((....((((.....))))......))...)))))........))))))).)))...)))))).((((((((...........((((((((...(((((((((.((((((((...((((((((.....((((((((((.(((((((((......((((((.......)))))))....))))))))).).)))))))).(((((((((...(((((((....((((((((....(((.........)))... ..))))))))....(((.........))).[illegible]	........(((((.......)))))............(((((.(((....(((......)))..))).)))))......		..................................((({{{{{{............AA....D...A...A(((((((((((...))))))))))))).....................{{{.................(((..((((((..((((((((..))))))))))...))))))...(((((.((((((..)))))).)))))..((((...))))..)))..(((.(.........[[)[)))......((((((((.....[[))))))))<<<<<<<<<<<<<<..(((((((.........)))))))).....(..........B.......)..((((....)))).................<<.......((((((..))))))).................[[(((((.>>...((((..<.))))).....))))....(((((........((((>....))))..........))))).........((((...((((((.......................))))))......................................<.<<.....((((((..((.(((((((((....<<((((((<<<<<<))))))BB.<)))))))))...))>)))))))..<<<<<<<.........<<.....(((((((........................))))))))....))))..((.....((((>>(((((...>>[illegible]

PDB	MXfold2	CONTRAfold	Centroidfold	Mcfold Default	Mcfold Pseudoknotted	Linearfold C	Linearfold V	Ufold	SPOT-RNA
5KH8	(((((......)))))......((((....)))).............	(((((......)))))......((((....)))).............	.....((((....))))......((((....))))..............	...((((((((....(((((..(((((..))))))))))))))))))	((((.[[[[[[[[))))((((..(((((..)))))))))]]]]]]]..	(((((......)))))......((((....))))..............	(((((......)))))......((((....))))..............	<<<<..((((((>>>>......((((....))))....))))))..	<<<<..((((((>>>>......(((((..)))))....))))))...
5KMZ	(((((.........)))))..........	(((((.........)))))..........	....	.......((((((((((..))))))))))	(((((..[[[[[[[))))).......]]]]]]]	....	....	<<<<..{((((.(((>>>>....))))}))))	(((((.........)))))..........
5KQE	((((((......((((((....))))))))))))	(((((((......((((((....))))))))))))	(((((((......((((((....))))))))))))	(((((((......(((((((..))))))))))))))	(((((((......(((((((..))))))))))))))	(((((((......((((((....))))))))))))))	(((((((......((((((....))))))))))))))	((((((.....(.(((((((..)))))))))))))))	(((((((......(((((((..))))))))))))))
5L1Z	((...((((......))))))	.....((((......))))..	.....((((......))))..	(((.((((((..)))))))))	(((.((((((..)))))))))	.....((((......))))..	.....((((......))))..	....	....((((((...).))))).
5LM7	.............(((((.....)))))	.............(((((.....)))))	.............(((((.....)))))	....((((..))))((((((...))))))	....((((..))))((((((...))))))	.............(((((.....)))))	.............(((((.....)))))	.............(((((.....)))))	.............(((((.....)))))
5LSN	((.((((((((((...)))))))))).))	((.((((((((((...)))))))))).))	((.(((((((((.....))))))))).))	((((((((((((...))))))))))))	((((((((((((...))))))))))))	((.(((((((((.....))))))))).))	((.(((((((((.....))))))))).))	((.((((((((((...))))))))).))	.(((((((((((.....))))))))))).
5M0I	(((((.((..((....)))))))))...	(((((.....((....))..)))))...	(((((.((..((....)))))))))...	(((((.((..(((..))))))))))...	(((((.((..(((..))))))))))...	....	....	(((((.((..((....)))))))))...	(((((.(..((((..))))))))))...
5N5C	((((((((...))))))))	((((((((...))))))))	..(((((.....)))))..	((((((((...))))))))	((((((((...))))))))	(((((((.....)))))))	(((((((.....)))))))	((((((((...))))))))	((((((((...))))))))
5NG6	(((((((((....)))))..))))	....(((((....)))))......	....(((((....)))))......	(((((((((....))))..)))))	(((((((((....))))..)))))	....(((((....)))))......	....(((((....)))))......	..(.(((((....))))).)....	(((((((((....)))))..))))
5TF6	(((((.((((........))))..))))).((((.....)))).........................	(((((....(........).....))))).((.((((.(((.....)))..)))).))..............	...(.........................((((.....))))..........................)..	.(((((((((((.(((.(((..)))))))((((((((((((...))))).)))))))))))))))))))))	.(((((((((((.(((.(((..))))))((((((((((((..)))))).)))))))))))))))))))	..............................((((.....))))..........................	..............................((((.....))))..........................	.(((..((((((((............<<.((((.(((>>...)))..)))).))))))..))..)))	.(((((((((((...................((((.....))))..................)))))))))))
5UDZ	(((((((.....)))))))....	(((((((.....)))))))....	((((((....)...)))))....	((((((((...))))))))....	.((((((.[[[[))))))..]]]]	((((((.......))))))....	((((((.......))))))....	(((((((.....)))))))....	((((((.......))))))....
5UF3	(((((((.((....)))))))))	(((((((.((....)))))))))	(((((((.((....)))))))))	((((((.(((....)))))))))	((((((.(((....))))))))))	((((((.(((....)))))))))	((((((.(((....)))))))))	(((((((.((....)))))))))	(((((((.(((..))))))))))
5UZT	(((((((.((..(((...))).)))))))))	(((((((.((((.(.....)))))))))))))	(((((((.((((........))))))))))))	((((((((((((((..))))).))))))))))	((((((((((((((..))))).)))))))))	(((((((.((((........))))))))))))	(((((((.((((........))))))))))))	(((((((.((....(...).)..))))))))	((((((((.((..........))))))))))
5V17	((((((.....(((((((((......))))))))))))))))	((((((.....(((((((((......))))))))))))))))	((((((.....(((((((((......))))))))))))))))	((((((.....((((((((((..))))))))))))))))	((((((.....((((((((((..))))))))))))))))	(((((((.....((((((((......)))))))))))))))	(((((((.....((((((((......)))))))))))))))	((((((.....(((((((((......))))))))))))))	((((((.....(((((((((......))))))))))))))))
5V6X	(((((((.((((.....))))((((((((.......)))))))..(((((.......)))))))))))))..	(((((((.((((.....))))((((((((.......)))))))..(((((.......)))))))))))))..	(((((((.((((.....))))((((((((.......)))))))..(((((.......)))))))))))))..	((((((((((((((((((((((..))).))))))))..))))))(((((...)..)))))).)))))))..	((((((((((((((((((((((..)))..))))))))..))))))(((((...)..))))).))))))..	(((((((.((((.....))))((((((((.......)))))))..(((((.......))))))))))))))..	(((((((.((((.....))))((((((((.......)))))))..(((((.......))))))))))))))..	(((((((.(...........).(((((.......))))))...(((((.......)))))))))))..	(((((((.((((.....))))((((((((.....))))))))..((((((...)..)))))))))))))..

PDB	MXfold2	CONTRAfold	Centroidfold	Mcfold Default	Mcfold Pseudoknotted	Linearfold C	Linearfold V	Ufold	SPOT-RNA
5VW1	..................((((....))))............................((((....))))....	..........(((((..((((....))))....)))))...((((()).))).......((((....))))...	..................((((....)))).............................((((....))))...	(((..)))((.(((((((((((..)))))))((((((((((((((..))))))))))))))))))))).)...	(((((((..(.((((((..)))))))[[[[[).)))))))...]]]]](((((((((((...))))))))).)))	..................((((....))))..	..................((((....))))..	............(((((.((((....))))...)))))(((((((()).)).)))).((((....))))...	..........((((((..(((((..)))))<<<<)))))).((((..).)))......>>>>..........
5W1H	......((((........))))......(((.......)))	...((.((((........)))).))...(((.......)))	......((((........))))..................	((((((((((((..))))))))(((((..)))))))))).	((((((((((((..))))))))(((((..)))))))))).	......((((........)))).................	......((((........)))).................	......((((........)))).....(((.......)))	......(((((......)))))..................
5WLH	.......((((........))))......(((.......)))	....((.((((........)))).))...(((.......)))	.......((((........)))).................	((((((((((((((..))))))))((((..)))))))))))	(((((((((((((..)))))))))((((..)))))))))))	.......((((........)))).................	.......((((........))))..................	....((.((((........)))).))...(((.......)))	.......(((((......))))).................
5WQ1	((((((((.....))))))))	((((((((.....))))))))	((((((((.....))))))))	(((((((((...)))))))))	(((((((((...)))))))))))	((((((((.....))))))))	((((((((.....))))))))	((((((((.....))))))))	((((((((.....))))))))
5WT1	((((..((((.........)))).((((((.......)))))).....(((((.......)))))))))	((((..((((...(..)..))))((((((.......))))).)...(((((.......))))))))	((((....................((((((.......))))).....(((((.......))))))))))	((((.(((((((((((((.(..))))))))))).))))))((((((((((((..)))))))))))))		.(((....((((.((((.......)))).)))).....)))....((((((..........)))))).	.(((....((((.((((.......)))).)))).....)))....((((((.........)))))).	((((.......((........)).(((((.......))))).....((((((.......)))))))))	((((<.((((>........))))((((((.......))))))....((((((...)..))))))))
5WT3	(((((((..((((.........)))).((((((.......)))))).....(((((.......)))))))))))	(((((((..((((..((()).))))(((((.......))))).)...(((((.......))))))))))))	(((((((..((((.........)))).(((((.......)))))......(((((.......)))))))))))			(((((((..((((.........)))).(((((.......))))).....(((((.......)))))))))))	(((((((..((((.........)))).(((((.......))))).....((((((....))))))))))))	(((((((..((((.........))).(((((.......))))).....(((((.......))))))))))))	(((((((<.((((>........))))((((((.......))))))....((((((...)..))))))))))))
5X6B	(((((((..((((.......)))).((((((....))))).....(((((.......)))))))))))))....	(((((((..((((((...)))))).((((((())).))))).....(((((.......))))))))))))....	(((((((..(((((.....))))).(((((.......))))).....(((((.......))))))))))))....	((((((((((((..))))))((((((((((..)).)))).)))))((((((...)..))))))))))))....		(((((((..((((((...)))))).((((((.......))))).....(((((.......))))))))))))....	(((((((..((((((...)))))).((((((.......))))).....(((((.......))))))))))))....	(((((((..((((.......)))).(((((.......))))).....(((((.......))))))))))))....	(((((((..(((((<..{.)))))(((((((.......))))))...>((((((.}.)..))))))))))))....
5XBL	.(((.(((((.((.....((((....))))...........)).)))))..))).....((((....))))(((((((...))))))).	..........(((((((.((((....))))...)))))))..(((..).)).......((((....))))(((((((...))))))).	..................((((....))))..............................((((....)))).((((((...))))))..	.(.((((((((((((((((((((..))))))..))))))..)))))))((((((((...))))))))(((((((...)))))))))	.(((((..[[[[[[[]))))))(((..))).(((((((((((((((..))))))))))))))))]]]]]](((((((...))))))).	..................((((....)))).....................................(((((((...))))))).	..................((((....))))..	..((.(....(((((((.((((....))))...)))))))<<).)()).....>>............(...((((((...))))))))	..........((((((.((((((..))))))...)))))).(((.....)))......((((..)))))((((((((.)))))))).
5Y58	(((((((..(((.......)))))))))).	((((((..((((.......)))))))))).	((((((..((((.......)))))))))).	((((((..((((((...)))))))))))).	((((((..((((((...)))))))))))).	((((((..((((.......)))))))))).	((((((..((((.......)))))))))).	(((((((..(((.......)))))))))).	((((((...((((.....)))).)))))).
6AAS	(((((((((((....)))))))))))	(((((((((((....)))))))))))	(((((((((((....)))))))))))	((((((((((((..))))))))))))	((((((((((((..))))))))))))	(((((((((((....)))))))))))	(((((((((((....)))))))))))	(((((((((((....)))))))))))	(((((((((((....)))))))))))
6B14	(((((((((.....(((...((.........(((.....)))(((((.(.....).)))))......))...)))))))))))	((((((((((..(((.......((.....(((((((.....)))))))....)).((((....))))))...))))))))))	(((((((((...................((((((((.....)))))))........((((....))))........)))))))))	((((((((.(((((((((((((((..)))))((((...))))))))))))))((((((.(((..)))))))))))))))))))	((((((((.((((((((((((((..)))))((((...)))))))))))))(((((((.(((..))))))))))))))))))	(((((((((.............((.....((((((((.....)))))))....)).((((....))))........)))))))))	(((.((.((...........)).)).)))(((((((.....)))))))........((((....)))).............	((((((((((.....................(((((((.....)))))))........((((....))))......))))))))))	((((((((((((((((((..(...).((((((((((.....)))))))..))...........))))))))).)))))))))

PDB	MXfold2	CONTRAfold	Centroidfold	Mcfold Default	Mcfold Pseudoknotted	Linearfold C	Linearfold V	Ufold	SPOT-RNA
6B3K	(((((((((..(((((((.....((((((......)))))))))))))).....((((....)))).......)))))))))	((((((((((.(((((((.....((((((..(())..)))))))))))))..(..((((....)))))......)))))))))	(((((((((.............((((((........))))))...........((((....))))........)))))))))	(((((((((.((((((((((((((((((..).)))))))))))))))))))((((((.(((..)))))))))))))))))))	(((((((((.(((((((((((((((((..).)))))))))))))))))((((((.(((..))))))))))))))))))))	(((((((((..(((((((.....((((((........))))))))))))).....((((....))))........)))))))))	...((((((..(((((((.....((((((........))))))))))))).....((((....))))........))))))	((((((((((.............<<<<<((((((..>>>>>))))))........((((....))))......))))))))))	(((((((((..((((((((....((((((<<<<<...))))))>>>>>...............))))))))...))))))))
6BHJ	..(((((((((((((((...))))))))))))))))	..(((((((((((((((...)))))))))))))))	..((((((((((((((...))))))))))))))	..((((((((((((((...))))))))))))))	..((((((((((((((...))))))))))))))	..((((((((((((((...))))))))))))))	..((((((((((((((...)))))))))))))))	..((((((((((((((...))))))))))))))	..((((((((((((((...)))))))))))))))
6CYT	((((.......)))..)	......((......)).	....	...((((((..))))))	...((((((..))))))	....	....	....	.....(((......)))
6D12	(((((((.....(((((....))))).....)))))))	(((((((((((.((((((())))))))))))).)))))))	(((((((.....(((((....)))))......)))))))	((((((((((((((((((..))))))))))))))))))	((((((((((((((((((..))))))))))))))))))	(((((((.....)))))))((((((.....)))))))	(((((((.....)))))))((((((.....)))))))	(((((((((((.((((((..))))))))))).)))))))	(((((((.....)))))))((((((.....)))))))
6DB8	(((((((((.......((.((.....)).))((((((........)))))..)))))))))	(((((((((.(.....(.(((((.....)))))(((........)))).).)))))))))	(((((((((.(....)....(((.....))).((((........))))...))))))))))	((((((((((((...(((((((...))))))))(((((((..))))))))))))))))))))	((((((((((((...((((((...)))))))(((((((..))))))))))))))))))))	(((((((((...........(((.....)))(((((........)))))..)))))))))	(((((((((...........(((.....)))(((((........)))))..)))))))))	(((((((((.........(((((.....)))))(((........)))....))))))))))	(((((((((..........((((.....))))((((........))))...)))))))))
6DTD	.((((....(((((((.....).))))))....))))	.((((....((((((........))))))....))))	..(((.....((((..........))))......))).	.((((((((((((((((((..))))))))))))))))))	.((((((((((((((((((..))))))))))))))))))	.((((.....(((((........)))))......))))	.((((.....(((((........)))))......))))	.((((..(.((((((........))))))).)..))))	.(((((((.((((((((.....).)))))).)))))))
6DU4	(((((((...............)))))))	(((((((...............)))))))	(((((((...............)))))))	((((((((((.(((..)))))))))))))	((((((((((.(((..)))))))))))))	(((((((...............)))))))	(((((((...............)))))))	(((((((.(...........).))))))))	(((((((.((..<))..>....)))))))
6DU5	((((..(((...)))....))))..	..(((((.............)))))	....	((((((((((...))))))))))..	((((((((((...)))))))))))..	..(((((.............)))))	..(((((.............)))))	..(((((.((...)....).)))))	..(((((.............)))))
6F4G	..((((((...........))))))	..((((((((....))...))))))	..((((((...........))))))	((((((((((((..)))))))))))).	(((((((((((((..)))))))))))))).	..((((((...........))))))	..((((((...........))))))	..(((((((((..)))...))))))	.<((((((((((..)))))..>)))))
6F4H	((((((..........))))))	((((((..........))))))	((((((..........))))))	((((((((((..))))))))))	((((((((((..))))))))))	((((((..........))))))	((((((..........))))))	((((((..........))))))	((((((..........))))))
6FQ3	(((((...)))))	(((((...)))))	((((.....))))	(((((...)))))	(((((...)))))	((((.....))))	((((.....))))	(((((...)))))	(((((...)))))
6FQL	(((((...)))))	(((((...)))))	.(((.....))).	(((((...)))))	(((((...)))))	....	....	(((((...)))))	((((((.))))))
6GBM	(((((((.........)))))))	(((((((.........)))))))	(((((((.........)))))))	(((((((.(((..))))))))))	(((((((.(((..))))))))))	(((((((.........)))))))	(((((((.........)))))))	(((((((.(......))))))))	(((((((.........)))))))
6HYK	(((((......((((....))))...)))))	(((((......((((....))))...)))))	(((((......((((....))))...)))))	(((((((..(.(((((..)))))))))))))	(((((((..(.(((((..)))))))))))))	(((((......((((....))))...)))))	(((((......((((....))))...)))))	(((((......(((((..)))))...)))))	(((((......(((((..)))))...)))))
6IV8	.(((((((((....))))))))))..........................	.((((((((((((())))))))))))))...........................	..((((((((....))))))))...............................	(((((((((((..))))))(.((((((((.((...))))))))))))))))	((((((((((.[[[[[))))))))..]]]]](((((((((..)))))))))	.(((((((((....))))))))))............................	.(((((((((....))))))))))..........................	.((((((((((((()))))))))))))..).........................	.((((((((((..)))))))))))..........................
6IV9	.(((((((((....)))))))))).........................	.((((((((((((()))))))))))))).........................	..((((((((....))))))))............................	...((((((((..))))))))(((((((((((...))))).))))))...	((((((((((.[[[[[))))))))..]]]]].((((((((..))))))))	.(((((((((....))))))))))........................	.(((((((((....))))))))))..........................	..(((((((((((()))))))))))).............................	.((((((((((.<)))))))))))........................>

PDB	MXfold2	CONTRAfold	Centroidfold	Mcfold Default	Mcfold Pseudoknotted	Linearfold C	Linearfold V	Ufold	SPOT-RNA
6JXM	(((((((..((((.......)))).((((((((....(((......))).....))))))))).....(((((.......))))))))))))....	(((((((..((((.......)))).(((((((((....(((......))).....))))))))).....(((((.......))))))))))))....	.........((((.......)))).(((((((((.....(.........)......)))))))))).....(((((.......)))))...........	(((((((((((((((...))))))))((((((((((((((((..)))))).))))))))))))((((((((((..))))))))).)))))))....	(((((((((((((((...))))))))((((((((((((((((..)))))).))))))))))))((((((((((..)))))))))).)))))))....	(((((((..((((.......)))).((((((((....(((......))).....)))))))))).....(((((.......)))))))))))....	.........((((.......)))).(((((((((....(((......))).....))))))))))...............	(((((((<.((((>{<<<.<)))).(((((((((...(((.......)))..)..)))))))))....}(((((>>>>...)))))))))))....	(((((((<.((((><..{..))))((((((((((....(((......))).....)))))))))...>((((((.}.)..)))))))))))....
6KYV	(((((((((....)))))))))	(((((((((....)))))))))	(((((((((....)))))))))	((((((((((..))))))))))	((((((((((..))))))))))	(((((((((....)))))))))	(((((((((....)))))))))	((((((((((..))))))))))	((((((((((..))))))))))
6MCE	(((((((..((((......)))))))))))	(((((((..((((......)))))))))))	(((((((..((((......))))))))))))	((((((((((((((..))))))))))))))	((((((((((((((..))))))))))))))	(((((((..((((......)))))))))))	(((((((..((((......)))))))))))	(((((((..((((......)))))))))))	(((((((..(((((...).))))))))))))
6MCF	((((((((((.(((((..((((((((....))).)))))))..))...))))))))))	((((((((((...((((.(((((((....))).)))).)..)))...))))))))))	((((((((((........(((((((....))).))))..........))))))))))	((((((((((((((((((((((((((..)))).))))))..))))))))))))))))	((((((((((((((((((((((((((..)))).))))))..)))))))))))))))))	((((((((((...(((..(((((((....))).))))....)))...))))))))))	((((((((((...(((..(((((((....))).))))....)))...))))))))))	((((((((((((.(.(..(((((((....))).))))).)...).).)))))))))	((((((((((.....((..((((((((..)))).))))....))....))))))))))
6MJ0	..((((.((((........(((((((((....)))))))))).((((((....))))))....((((.......)))((((((.....)))))).))))))))	..(((((.(((........(((((((((....))))))))).((((((....)))))).))((((.......))))(((((.....))))).....)))))	..((((..............((((((((...))))))))..((((((....)))))).....(((.......))).(((((.....))))).......))))			...................(((((((((....))))))))).((((((....)))))).. ..((((.......)))).......((((((.....))))))..	...................(((((((((....))))))))).((((((....)))))).....((((.......))))....	.......(<((.......)).).((((((<<<.)))))>>>..(((((((....))))))...((((.......))))(((((.....))))).>........	<<.....(((((>>...))))).....((((((....))))))(((((((..))))))))...(((((...)..))))<<<.....(((((>>>...)))))..
6MXQ	((((((.(((...(((.((.(((....)))))..)))...)))))))))	((((((.(((.(((((.......)))))...........)))))))))	((((((.(((...(((....(((....)))....)))...)))))))))	((((((.(((((((((((((((((..))))))))))))))))))))))))	((((((.(((((((((((((((((..)))))))))))))))))))))))	((((((.(((.(((((.......)))))............)))))))))	((((((.(((.(((((.......)))))............)))))))))	((((((.....(((((.......)))))(............)))))))	((((((.....(((((.......))))).............))))))
6NOA	((((((.(((...(((.((.(((..........)))))..)))...)))))))))	((((((.(((...(((.((..((..........))..))..)))...)))))))))	((((((..((..............................)).))))))	((((((.((((((((((((((((((((..))))))))))).))))))))))))))	((((((.((((((((((((((((((((..)))))))))))).)))))))))))))	((((((.(((....................................)))))))))	((((((.(((....................................)))))))))	((((((...........((.((...........)).))...........))))))	((((((.(((...................................)))))))))
6PK9	(((((((......)))))))	(((((((......)))))))	(((((((......)))))))	(((((((((..)))))))))	(((((((((..)))))))))	(((((((......)))))))	(((((((......)))))))	(((((((......)))))))	(((((((......)))))))
6SDY	((((((((((((((....))))))))))))))))	(((((((((((((((....))))))))))))))))))	(((((((((((((((....))))))))))))))))	(((((((((((((((((..))))))))))))))))	((((((((((((((((..))))))))))))))))	((((((((((((((....))))))))))))))	((((((((((((((....))))))))))))))	(((((((((((((((..))))))))))))))))	((((((((((((((((..))))))))))))))))
6SNJ	((((((((((.......)))))).))))	((((((((((.......)))))).))))	((((((((((.......)))))).))))	((((((((((((..)).)))))).))))	((((((((((((..)).)))))).))))	((((((((((.......)))))).))))	((((((((((.......)))))).))))	((((((((((.(....)))))))).))))	((((((((((((....).)))))).))))
6SY6	.(((...........(((....)))........)))	...............(((....)))...........	...............(((....)))...........	.((((...((((((((((((..))))))))))))))))	.((((...((((((((((((..)))))))))))))))))	...............(((....)))..........	...............(((....)))..........	.(((....(.((.(.((((..))))).)).)..)))	.(((...........((((..)))).......)))
6TQB	((((((((...))))))))	((((((((...))))))))	(((((((.....)))))))	((((((((...))))))))	((((((((...))))))))	((((((((...))))))))	((((((((...))))))))	((((((((...))))))))	(((((((.....)))))))
6U79	((((((((((((((.....))).)).))))))))))	((((((((((((((.(()))))).)).)))))))))))	(((((((((((((.......)).)).))))))))))	((((((((((.(((((((..)))))))))))))))))	((((((((((.(((((((..))))))))))))))))))	(((((((((((((((.....))).)).))))))))))	(((((((((((((((.....))).)).))))))))))	((((((((((((((.(()))))).)).))))))))))	((((((((((((...........)).))))))))))

PDB	MXfold2	CONTRAfold	Centroidfold	Mcfold Default	Mcfold Pseudoknotted	Linearfold C	Linearfold V	Ufold	SPOT-RNA
6U8D	.((((((((((..((((((....))))))((((((((....))))))).(((....)))))))))))))	.((((((((((..((((((....))))))(((((((....))))))).(((((()))))))))))))))	.((((((((((..((((((....))))))(((((((....))))))).(((....)))))))))))))	.((((((((((..((((((....))))))(((((((..)))))))).((((..))))))))))))))	.((((((((((..((((((....))))))(((((((..)))))))).((((..))))))))))))))	.((((((((((..((((((....))))))(((((((....))))))).(((....))))))))))))))	.((((((((((..((((((....))))))(((((((....))))))).(((....))))))))))))))	.((((((((((..(((((..<<<<)))))((((((....))))))).((..>>>>))))))))))))	.((((((((((..((((((....))))))(((((((..)))))))).((((..))))))))))))))
6VZC	((((((..((((....))))..))))))	((((((..((((....))))..))))))	((((((...(((....)))...))))))	((((((((((((((..))))))))))))))	((((((((((((((..)))))))))))))	((((((..((((....))))..))))))	((((((..((((....))))..))))))	(((((((.((((((..)))))).))))))	((((((..((((((..))))))..))))))
6W3M	(((((((((...((((((.....))))))...))))))))	(((((((((...((((((.....))))))...)))))))))	(((((((((...((((((.....))))))...)))))))))	((((((((((((((((((...))))))))))))))))))	(((((((((((((((((((...))))))))))))))))))	(((((((((...((((((.....))))))...)))))))))	(((((((((...((((((.....))))))...)))))))))	(((((((((...((((((.....))))))...)))))))))	(((((((((...(((((((...)))))))...)))))))))
6XH0	(((((...((((......)))))))))	(((((...((((......)))))))))	(((((...((((......)))))))))	(((((...(((((...).)))))))))	(((((...(((((...).)))))))))	(((((...((((......)))))))))	(((((...((((......)))))))))	(((((...((((......)))))))))	(((((...(((((...).)))))))))
6XWJ	(((((((((...)))))))))	(((((((((...)))))))))	((((((((.....))))))))	(((((((((...)))))))))	(((((((((...)))))))))	(((((((((...)))))))))	(((((((((...)))))))))	(((((((((...)))))))))	(((((((((...)))))))))
7K1Z	(((((((((..((((((((.((.((((((.......)))..)).))...)))))))))........((((.....)))........((....))...))))))))))	(((((((((..(((((((((((.(.((((.........))).).).))...)))))))))........((((.....)))((((........))))...)))))))))	(((((((((..((((((((......((((.......)))..........)))))))).......(((.....)))........(....)....)))))))))			(((((((((..((((((((.((.(((((.......)))..)).))...))))))))).........(((.....)))((((........))))...))))))))))	...........((((((((.((.((((((.......)))..)).))...))))))))........(((.....)))....	(((((((((.((((((((((.(..((((.......)))..)..))...))))))))........(((.....))).................)...)))))))))	(((((((((..((((((((....((.............))......))))))))........(((((...))))).................)))))))))
