# Supplementary material for: Comparative Study of Single-stranded Oligonucleotides Secondary Structure Prediction Tools
Source: BMC Bioinformatics. 2023 Nov 8;24:422. doi: 10.1186/s12859-023-05532-5 (PMC10634105; doi:10.1186/s12859-023-05532-5)

Additional File 9. Percentages of correctly predicted ($Apta_D$ = 0, blue bars), acceptably predicted ($Apta_D \leq 1.5$, light blue bars), and incorrectly predicted $Apta_D$ >1.5, red bars) ssDNA structures by the considered secondary structures prediction tools. Structures predicted as unfolded or unpredicted are represented with grey bars.

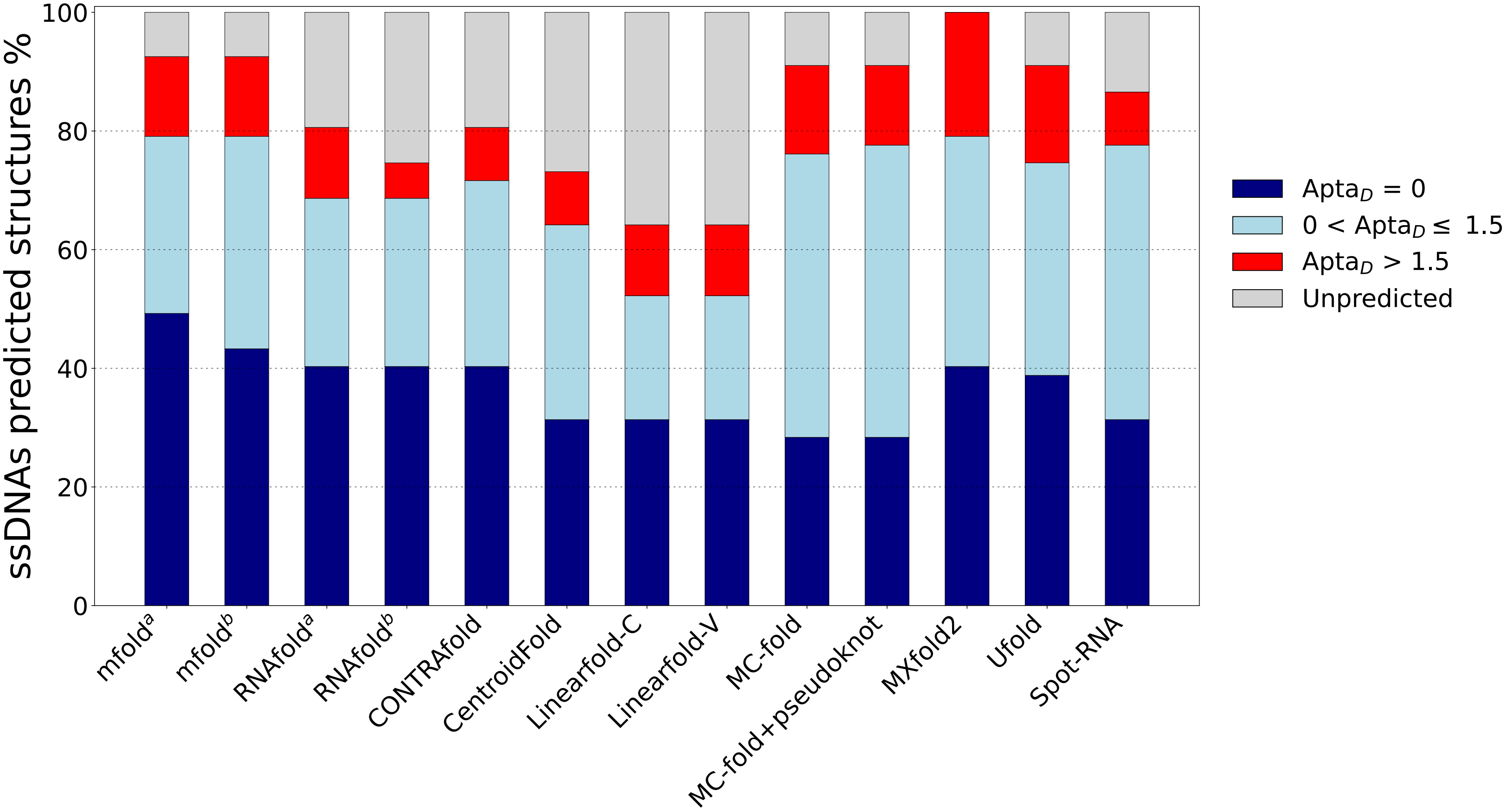

Supplement: Supplementary file 9 — Additional file 9. Percentages of correctly predicted ($Apta_D = 0$AptaD=0 , blue bars), acceptably predicted ($Apta_D \leq 1.5$AptaD≤1.5, light blue bars), and incorrectly predicted ($Apta_D > 1.5$AptaD>1.5, red bars) ssDNA structures by the considered secondary structures prediction tools. Structures predicted as unfolded or unpredicted are represented with grey bars. [file 12859_2023_5532_MOESM9_ESM.pdf]
