# Supplementary material for: Comparative Study of Single-stranded Oligonucleotides Secondary Structure Prediction Tools
Source: BMC Bioinformatics. 2023 Nov 8;24:422. doi: 10.1186/s12859-023-05532-5 (PMC10634105; doi:10.1186/s12859-023-05532-5)

Additional File 12. Percentages of correctly predicted (MCC = 1, blue bars), acceptably predicted (MCC ≥ 0.5, light blue bars), and incorrectly predicted MCC < 0.5, red bars) ssNA structures by the considered secondary structures prediction tools. Structures predicted as unfolded or unpredicted are represented with grey bars.

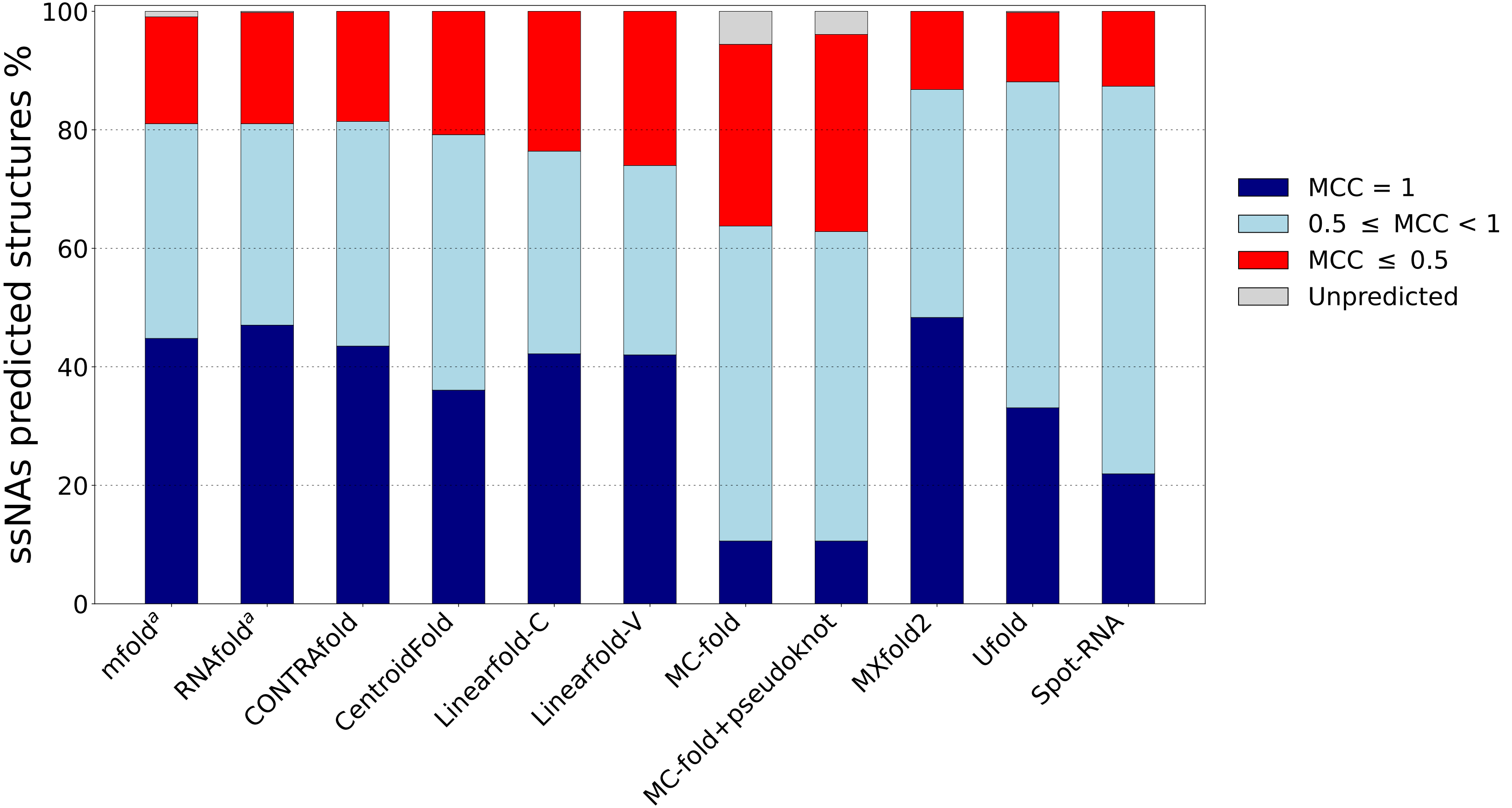

Supplement: Supplementary file 12 — Additional file 12. Percentages of correctly predicted (F1 score = 1, blue bars), acceptably predicted ($F1 \, score \geq 0.5$F1score≥0.5, light blue bars), and incorrectly predicted (F1 score < 0.5, red bars) ssNA structures by the considered secondary structure prediction tools. Structures predicted as unfolded or unpredicted are represented with grey bars. [file 12859_2023_5532_MOESM12_ESM.pdf]
