# Supplementary material for: Comparative Study of Single-stranded Oligonucleotides Secondary Structure Prediction Tools
Source: BMC Bioinformatics. 2023 Nov 8;24:422. doi: 10.1186/s12859-023-05532-5 (PMC10634105; doi:10.1186/s12859-023-05532-5)
Supplement: Supplementary file 13 — Additional file 13. Example of the difference in the classification of the predicted structures using AptaMat, F1 score and MCC as metrics. The experimental structure corresponds to the 2NC1 PDB code, the predicted structure are those obtained by MXfold2, MC-fold, and SPOT-RNA, respectively. [file 12859_2023_5532_MOESM13_ESM.pdf]

Additional File 13. Example of the difference in the classification of the predicted structures using AptaMat, F1 score and MCC score as metrics. The experimental structure corresponds to the 2NC1 PDB code, the predicted structures are those obtained by MXfold2, MC-fold, and SPOT-RNA, respectively.

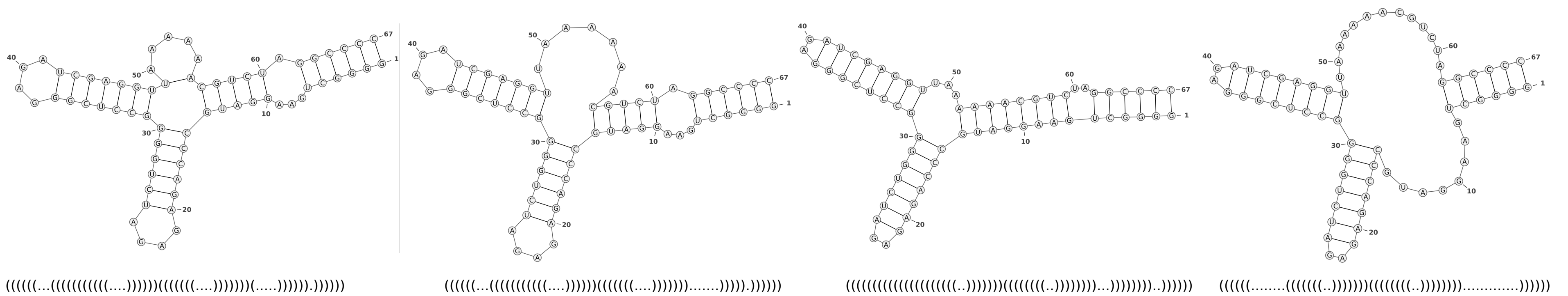

AptaMat	0.510	1.500	1.717
F1	0.980	0.703	0.826
MCC	0.954	-0.010	0.665
