# Supplementary material for: Comparative Study of Single-stranded Oligonucleotides Secondary Structure Prediction Tools
Source: BMC Bioinformatics. 2023 Nov 8;24:422. doi: 10.1186/s12859-023-05532-5 (PMC10634105; doi:10.1186/s12859-023-05532-5)

Additional File 14. Histogram of the $Apta_D$ obtained for the UFold predictions of the structures containing pseudoknots (the 4 predictions with $Apta_D > 5$ were discarded to facilitate the plot reading).

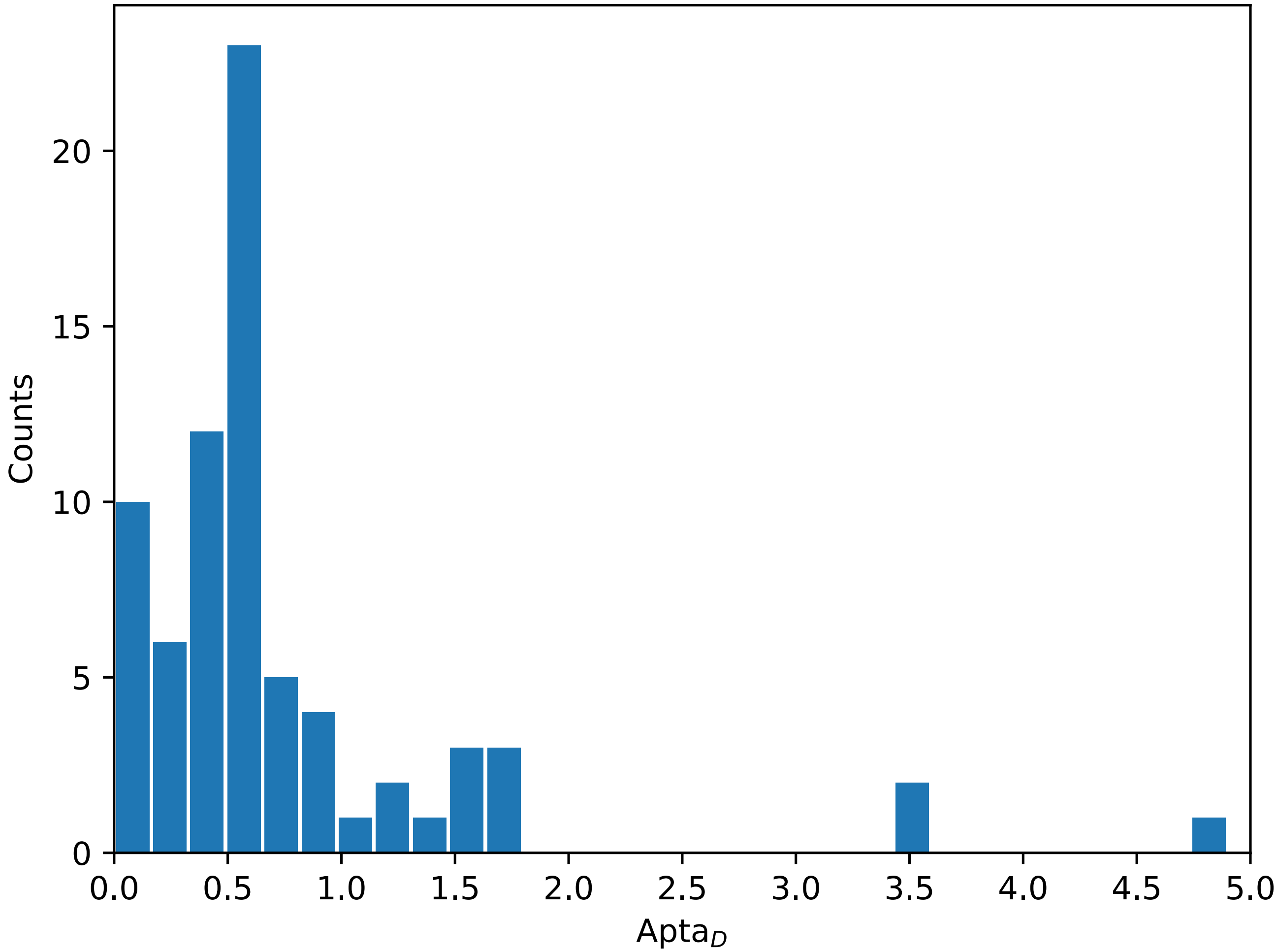

Supplement: Supplementary file 14 — Additional file 14. Histogram of the AptaD obtained for the UFold predictions of the structures containing pseudoknots (the 4 predictions with AptaD > 5 were discarded to facilitate the plot reading). [file 12859_2023_5532_MOESM14_ESM.pdf]
