# Supplementary material for: Comparative Study of Single-stranded Oligonucleotides Secondary Structure Prediction Tools
Source: BMC Bioinformatics. 2023 Nov 8;24:422. doi: 10.1186/s12859-023-05532-5 (PMC10634105; doi:10.1186/s12859-023-05532-5)
Supplement: Supplementary file 16 — Additional file 16. Example of pseudoknot-containing secondary structure predicted by UFold and SPOT-RNA and aligned to the experimental one (code PDB 3WC2). [file 12859_2023_5532_MOESM16_ESM.pdf]

Additional File 16. Example of pseudoknot-containing secondary structure predicted by UFold and SPOT-RNA and aligned to the experimental one (code PDB 3WC2).

```
3WC2
Ref     :  (((((((..((((.....[..)))).(((((.......))))).....(((((..]....))))))))))))..
UFold   :  (((((((..((((....[...)))).(((((.......))))).....(((((.].....)))))))))))).. AptaMat distance 0.091
SPOT-RNA: (((((((([.((((][......))))((((((.......))))))...]((((((...)..)))))))))))).. AptaMat distance 0.915
```
